# Proceedings of Réanimation 2018, the French Intensive Care Society International Congress

**DOI:** 10.1186/s13613-017-0345-7

**Published:** 2018-02-05

## Oral communications

### CO-01 Respiratory viral infection in ICU patients with respiratory failure: a snapshot during multiplex PCR era

#### Epaillard Nicolas^1^, Bige Naike^1^, Dumas Guillaume^1^, Ait Oufella Hafid^1^, Maury Eric^1^

##### ^1^Hôpital Saint-Antoine, Paris, France

###### **Correspondence:** Epaillard Nicolas - nicolas.epaillard@aphp.fr

*Annals of Intensive Care* 2018, **8(Suppl 1):**CO-01

**Introduction:** The role and incidence of viral infections in respiratory failure occurring in ICU patients is more and more investigated. The availability of multiplex PCR assay is actually frequently performed. The aim of this retrospective study was to assess presence of viral species using a multiplex PCR assay in adult patients admitted to ICU for respiratory failure during the November–April period.

**Patients and methods:** All adult patients admitted to ICU during the November–April period for respiratory failure were eligible. A respiratory sampling was performed according to patient severity—sputum, tracheal aspiration, or per fibroscopic broncho alveolar lavage (LBA) and was analyzed for conventional bacterial strain. In all the patients, a multiplex vial PCR assay was performed on pharyngeal aspirate or LBA fluid.

**Results:** During the study period 123 patients were analyzed (male 76, SAPS II: 49 ± 17 + age 69 ± 18). 70 of them had at least one respiratory comorbidity. Forty-nine patients required invasive mechanical ventilation. Shock defined by requirement of vasoconstrictor was recorded in 31 patients (23%). In ICU mortality was 23%. Multiplex PCR assay was positive in 23 patients (17%). Co infection by bacteria and virus was noted in 4 patients. Influenzae virus was found in 10 patients and VRS in nine. Four patients with a positive viral sample had a fatal outcome (Influenza virus was found in all these). Mechanical ventilation was required in 45% of patients who were VRS positive but all had a favourable outcome.

**Conclusion:** Positive viral sample is not infrequent in ICU patients with respiratory failure. VRS is in this study as frequently found than influenzae.

### CO-02 Low plasma citrulline concentration is associated with sepsis among critically ill patients

#### Piton Gaël^1^, Chaignat Claire^1^, Renaud Loïc^1^, Benoit Cypriani^1^, Hadrien Winiszewski^1^, Gilles Capellier^1^

##### ^1^CHU Besançon, Besançon, France

###### **Correspondence:** Piton Gaël - gpiton@chu-besancon.fr

*Annals of Intensive Care* 2018, **8(Suppl 1):**CO-02

**Introduction:** Plasma citrulline concentration, a biomarker of enterocyte function, is frequently low among critically ill patients. Plasma citrulline concentration might be decreased among critically ill patients presenting with sepsis, reflecting an alteration of small bowel function. We aimed to study the link between plasma citrulline concentration measured at ICU admission and variables related to the sepsis in critically ill patients.

**Patients and methods:** This was a prospective observationnal study performed in a University Hospital. Critically ill patients admitted to the ICU, aged 18 years or more, with ICU duration expected > = 48 h, without chronic renal failure or small bowel disease, were included. Plasma citrulline concentration, plasma I-FABP (biomarker of enterocyte damage), search for sepsis, results of bacterial sample, and usual clinical and biological variables were collected. We compared patients presenting with low plasma citrulline concentration (< = 10 µmol L), suspected of presenting enterocyte dysfunction, and patients with plasma citrulline concentration > 10 µmol L.

**Results:** Between July and December 2016, 109 patients, aged 64 years, 61% males, SOFA score 8 (6–12), were included in the study. Sepsis was found in 43 patients (40%), mainly of pulmonary (58%) and digestive (21%) origins. A plasma citrulline concentration ≤ 10 µmol L at ICU admission was associated with presence of Gram Negative or Candida infection, and higher plasma concentration of CRP and PCT (all p < 0.05). Plasma citrulline concentration was lower among septic patients than among patients without sepsis (12.0 µmol L [8.6–17.1] vs 20.5 µmol L (11.2–27.1) respectively, p = 0.004). Plasma citrulline concentration was not different among patients presenting with a sepsis of digestive origin and extra-digestive origin (7.8 [7.2–25.2] and 13.4 [9.8–16.3] respectively, p = 0.29). Plasma I-FABP was significantly higher among non-survivors (p = 0.005). Other factors associated with 28-day mortality were the age, plasma creatinine and lactate concentrations, and SOFA score.

**Conclusion:** This study confirms the link between sepsis and low plasma citrulline concentration in critically ill patients. The gut might be both the culprit, responsible for bacterial translocation, and the victim of sepsis. Indeed, the fact that plasma citrulline was also reduced during extra-digestive sepsis suggests the existence of septic enteropathy. I-FABP confirms its prognostic value among critically ill patients. The prognostic value of plasma citrulline in the critically ill requires further evaluation.

### CO-03 Point of care use of the multiplex PCR Unyvero Curetis for the rapid diagnosis of low respiratory infections: performances and practical consequences

#### Guerpillon Brice^1^, Negre Jean^1^, Bohe Julien^1^, Muszynska Monika^1^, Thiery Guillaume^1^, Allaouchiche Bernard^1^

##### ^1^Hospices civils de Lyon, Lyon, France

###### **Correspondence:** Guerpillon Brice - guerpillonbrice@gmail.com

*Annals of Intensive Care* 2018, **8(Suppl 1):**CO-03

**Introduction:** Lower respiratory tract infections are very common in intensive care units and require rapid microbiological diagnosis in order to optimize the clinical progression and minimize the ecological impact. It currently takes 2–3 days to obtain an antibiogram using conventional microbiological techniques, compared with just a few hours for some multiplex PCR methods.

This prospective observational study focuses on a technique currently available, the multiplex PCR (MxPCR) Unyvero^®^ system that targets the 20 bacteria most commonly implicated in these infections as well as 17 of the most widespread antibiotic resistance genes. Results can be obtained within 5 h.

The primary objective of this study was to demonstrate the diagnostic performance of this technique, and the secondary objective was to assess its practical impact.

**Patients and methods:** Between December 2016 and September 2017, point-of-care testing using MxPCR and conventional microbiological techniques were performed on respiratory specimens from patients with pulmonary infection in 2 intensive care units. Results, delay to obtain it, clinical and therapeutic datas were recorded.

**Results:** The main test results are presented in Table 1.

**Table 1:** Main tests results

	Number	Percentage	
*Positive samples using CM*	36		
*Positive samples using MxPCR*	43		
*Concordance between CM and MxPCR*			
Identification	65	76	
Resistance			
Total concordance	17	55	
Partial discordance	7	23	
Major discordance	7	23	
Not concerned	55		
*Impact of MxPCR*			
Beneficial	18	45 (39)	
Probably beneficial	21		
Futile	30	42 (36)	
Probably futile	6		
Detrimental	5	13 (11)	
Probably detrimental	6		
*Population deriving benefits from MxPCR*	39	45	Saving days
Accelerated de-escalation	24	28	2* (2–3)
Earlier weaning	11	13	5* (2–7)
Earlier appropriate escalation	7	8	2* (1–4)

A total of 86 out of 103 samples collected were fully analyzed. The results obtained for 17 samples could not be interpreted due to a handling error (1), technical failures (14) and the absence of conventional antibiograms (2). Samples collected were mostly tracheal aspirates (45). Pathogenic bacteria were detected more often using MxPCR than conventional bacteriology techniques (43 samples compared to 36). Concordance ratio between those two technics is described in Table 1. Antibiotic therapy initiated in the 48 h preceding sample did not increase the mismatching (p = 0.42). Antimicrobial agents administered before microbiological sampling did not alter the concordance between culture and PCRMx (p = 0.42). Despite some inconsistencies, particularly regarding the detection of nonpathogenic saprophytic resistance, clinical and or ecological benefits were observed in 42% of cases with this technique. The benefits were mainly ecological with de-escalation in 24 cases and weaning off antibiotics in 11 cases. The clinical benefits (adjustment of therapy for bacteria not targeted empirically) were more limited as only 7 patients were affected and adjustment was delayed by 2.4 days on average.

**Conclusion:** Thanks to this technique, the treatment of respiratory infections could have been optimized in 42% (39/86) of cases with a modest clinical gain (7/86) but a significant ecological gain (35/86).

### CO-04 Biomarkers to discriminate between infeted and non-infected patients at the time of sepsis criteria: the CAPTAIN multicenter cohort study

#### Misset Benoît^1^, Philippart François^2^, Parlato Mariana^3^, Rouquette Alexandra^4^, Moucadel Virginie^5^, Puchois Virginie^6^, Bedos Jean-Pierre^7^, Diehl Jean-Luc^8^, Hamzaoui Olfa^9^, Annane Djillali^10^, Journois Didier^8^, Ben Boutieb^11^, Myriam, Esteve Laurent^6^, Fitting Catherine^6^, Treluyer Jean-Marc^12^, Pachot Alexandre^13^, Adib-Conquy Minou^6^

##### ^1^CGU de Rouen, France; ^2^Hôpital Saint Joseph, Paris, France; ^3^Hôpital Pitié Salpêtrière, Paris, France; ^4^CHU de Montpellier, France; ^5^Biomérieux, Grenoble, France; ^6^Institut Pasteur, Paris, France; ^7^CH de Versailles, France; ^8^Hôpital Européen Georges Pompidou, Paris, France; ^9^CHU Paris-Sud, Paris, France; ^10^Hôpital Raymond Poincaré, Garches, France; ^11^Hôpital Cochin, Paris, France; ^12^Hôpital Necker, Paris, France; ^13^Biomerieux, Lyon, France

###### **Correspondence:** Misset Benoît - bmisset@wanadoo.fr

*Annals of Intensive Care* 2018, **8(Suppl 1):**CO-04

**Introduction:** Sepsis and non-septic Systemic Inflammatory Response Syndrome (SIRS) are two similar syndromes, which differ by their cause, sepsis being secondary to microbial infection. Microbiological tests are not enough to early affirm the bacterial or fungal origin of the syndrome. More than fifty biomarkers have been proposed for this purpose but none have been repeatedly validated in external populations. Our goal was to address the accuracy of the biomarkers already reported as being able to efficiently discriminate between sepsis and non-septic SIRS.

**Patients and methods:** Prospective observational multicenter cohort of 363 consecutive patients with criteria of SIRS who were included when the attending physician considered they required antimicrobial therapy. We collected blood at inclusion to measure 53 biomarkers, including 29 plasma compounds, 10 whole blood RNA and 14 leukocyte surface markers. Patients were classified as sepsis or non-septic SIRS a posteriori, blindly to the biomarker results. Performance of each marker was assessed by AU-ROC curve. Multivariate analysis used a lasso regression (Tibshirani 1996; Musoro 2014), including multiple imputation for missing values and bootstrap validations, adapted for high numbers of variables (biomarkers).

**Results:** Eighty-four patients had exclusion criteria. Ninety-one patients had non-septic SIRS and 188 had sepsis. Median [IQ] SAPS 2 score was 55 [50–61] in both groups (p = 0.81). Time from ICU admission to inclusion was 1 [0–1] day. 69.8% of infections were pulmonary, most frequent strains were *E. coli* (23%), *S. aureus* (15%) and *P. aeruginosa* (13%). Causes of non septic SIRS were circulatory (37%), inflammatory (25%), hypoxemia (22%) and neurological (16%). Only 8 biomarkers had a ROC-AUC over 0.6 with a 95%IC over 0.5. In multivariate lasso analyses, only CRP and HLA-DRA circulating RNA were repeatedly associated with sepsis, and no model performed better than CRP alone (ROC-AUC 0.76 [0.68–0.84]).

**Discussion:** Strengths of this series are (1) the similar severity of SIRS and sepsis groups, (2) a multicenter design and (3) the use of a multivariate method adapted to the high number of independent variables.

**Conclusion:** In this prospective cohort, among 53 previously published biomarkers, CRP was found to better discriminate between sepsis and non septic SIRS than the other biomarkers. However, its performance was insufficient to be useful for routine use. HLA-DR circulating RNA was the only independent marker predicting infection. Multivariate models did not improve the performance of CRP alone.

### CO-05 Procalcitonin in necrotizing soft tissue infection: an interesting prognostic marker and a potentially useful marker to guide the antimicrobial therapy duration

#### Pouly Olivier^1^, Parmentier-Decrucq Erika^1^, Duburcq Thibault^1^, Thieffry Camille^1^, Mathieu Daniel^1^, Poissy Julien^1^

##### ^1^CHRU Lille, France

###### **Correspondence:** Pouly Olivier - olivier.pouly@hotmail.fr

*Annals of Intensive Care* 2018, **8(Suppl 1):**CO-05

**Introduction:** Procalcitonine (PCT) is a useful biomarker to reduce antibiotic exposure in intensive care units (ICU), especially in respiratory tract infections. Nevertheless, its usefulness for necrotizing soft tissue infections (NSTI) has not yet been reported. We aimed to assess PCT as a prognostic biomarker and a marker to guide duration of antimicrobial therapy in NSTI.

**Patients and methods:** We retrospectively included all the patients hospitalised for NSTI between January 1st 2013 and 31 December 2015 in an ICU department of a teaching hospital. Initial PCT value and kinetics, demographic and clinical data were reported. We compared the duration of the antibiotic course with: 1) the IDSA guidelines that recommend to use the decrease of fever and the need for surgical debridement and, 2) a theoretical duration if a PCT-based algorithm has been applied.

**Results:** We included 196 patients. Median LRINEC score was 6 [5–8.5] and median SOFA score was 6 [4–10]. At D28, mortality was 19.4%, significantly linked with initial PCT value (28.6 ng mL [3.5–58.7] in dead patients versus 3.1 ng mL [0.7–11.8] in survival ones (p = 10^–4^). There was no decrease in PCT kinetic for 12.2% of the patients who survived versus 62.5% among those who died at D28 (p < 10^–5^). The duration of the antimicrobial therapy was 12.5 days [10–17]. Stopping antimicrobial therapy if PCT levels was inferior to 0.5 ng mL or decreased of more than 80% from the initial value would have reduced the antibiotic treatment by 6.5 days [5–8] (p = 10^–5^). It would have been possible wherever the localisation of the infection. But 17.9% of patients could not have had a PCT guided antibiotic duration because of an initial PCT value < 0.5 ng mL, especially in the cervical cellulitis group. Antimicrobial therapy would have been reduced by 4 days if IDSA guidelines had been applied [5–13] (p = 10^–5^). The reduction of the antibiotic therapy was superior with the PCT algorithm compared to IDSA guidelines (p = 5.10^–4^).

**Conclusion:** Procalcitonin could be a good prognostic marker as well as a relevant tool to reduce the antibiotic therapy duration in the severe NSTI. We will nevertheless have to prospectively determine the impact of such a practice on patient prognosis, including mortality and need for reintervention.

### CO-06 Health related quality of life trajectories of patients after acute illness

#### Benammi Sarah^1^, A. El Khattate^1^, M. Bizrane^1^, N. Madani^1^, Belayachi Jihane^1^, R. Abouqal^1^

##### ^1^Ibn Sina University Hospital, Rabat, Morocco

###### **Correspondence:** Benammi Sarah - sara-benammi@hotmail.fr

*Annals of Intensive Care* 2018, **8(Suppl 1):**CO-06

**Introduction:** This study aimed to identify and describe a set of longitudinal HRQoL trajectories then determine factors associated with trajectory class membership of patients after acute illness.

**Patients and methods:** This was a prospective cohort study conducted in an acute medical unit of University Hospital, between June–September 2014. Patients aged ≥ 17 years admitted to AMU were included. Demographic, medical history, clinical and paraclinical characteristics were recorded at admission. EQ5D index, EQ-VAS and survival status of patients were collected at admission, 3, 6 and 18 months of follow-up. Latent class growth analysis (LCGA) was applied to identify classes of HRQoL trajectories, while association between baseline covariate and class membership were identified using polynominals logistic regression. Statistical analysis was carried out in STATA version 14.

**Results:** Two hundred fifty-one patients were included. The mean age was 55.6 ± 18.9 years, women were 54.6%. In-hospital and 540 days follow-up mortality were respectively 11.6 and 34.3%. For 229 patients included in LCGA, three trajectory classes where identified for EQ5D Index + stably low (16.2%), stably moderate (30.6%), and high initially increasing (53.2%). The three trajectory classes of EQ-VAS were low increasing, moderate initially increasing and high initially increasing with, respectively, 29.7, 48.5 and 21.8% of the patients. Concerning EQ5D index, comparing to high initially increasing trajectory, factors associated to—(a) stably low trajectory membership were-age ≥ 70 years (OR 9.3; CI 1.7–49; p = 0.008), intensive care unit transit (OR 5.1; CI 0.01–10; p = 0.01) and low hemoglobinemia (OR 0.8; CI 0.05–1.1; p = 0.05) + (b) stably moderate trajectory membership were—comorbidity (OR 2.8 + CI 0.01–6.4 + p = 0.01) and low hemoglobinemia (OR 0.8; CI 0.001–0.9; p = 0.001). Concerning EQ-VAS, comparing to high initially increasing trajectory, factors associated to—(a) low increasing trajectory membership were—female gender (OR 5; CI 1.7–10; p = 0.006), Km hospital-residence (OR 0.9; CI 0.9–0.9; p = 0.01) and comorbidity (OR 9.6; CI 3.1–29; p < 0.001), (b) moderate initially increasing trajectory membership was comorbidity (OR 9.5; CI 3.7–24; p < 0.001).

**Conclusion:** Three HRQoL trajectories were identified. Aged patients with low hemoglobinemia and who transited through ICU had the worst EQ5D index. Females, with comorbidities, living far from hospital perceived an amelioration of their, previously low, EQ-VAS.

### CO-07 Post-traumatic stress disorder after intensive care unit discharge: prevalence and impact on quality of life

#### Pottier Véronique^1^

##### ^1^Hôpital de la Côte de Nacre, Caen, France

###### **Correspondence:** Pottier Véronique - veroniquepottier@icloud.com

*Annals of Intensive Care* 2018, **8(Suppl 1):**CO-07

**Introduction:** Hospitalization in ICU is a period of severe stress that can be experienced as a real trauma leading to post traumatic stress disorder (PTSD) after hospital discharge. The objective of this study is to determine the prevalence of PTSD and its impact on the quality of life at 3 months, 6 months and 1 year after ICU discharge.

**Patients and methods:** A before and after prospective, observational, non-interventional study assessing the impact of a nurse implemented sedation and analgesia algorithm on complications of critical illness and on outcome of patients after their ICU stay, was conducted in surgical ICU, between November 2014 and April 2017. During the “Before” period, sedation and analgesia was managed by the physician, while during the “After” period, it was managed by the nurses. Survivors were followed up by a phone survey at 3 months, 6 months and 1 year after ICU discharge.

**Results:** 1156 intubated and mechanically ventilated patients were admitted during the study period. Among the 145 included patients, 47 died and 35 were lost or disapproved the follow-up before the 3rd month. Only 62 patients accepted follow-up at 3 months (42.8%), 55 at 6 months (37.9%) and 42 at 1 year (29%). The presence of a new hospitalization after ICU discharge varies from 24.2 to 29.1%, between 3 months and 1 year, and the use of psychotropic drugs from 25.5 to 29%. The prevalence of PTSD (PTSS-10 score ≥ 35) was 12.9% at 3 months, 10.9% at 6 months and 16.7% at 1 year. ICU Stay has a moderate impact on quality of life at 3 months, 6 months and 1 year (62 < WHOQOL-BREF score < 96). Approximately 10% of patients have a severe impact on their quality of life (WHOQOL-BREF > 61) at ICU discharge, up to 1 year. The PTSS-10 score at 3 months is significantly higher in the “Before” period. There is no difference at 6 months and 1 year, nor significant difference on quality of life between the 2 periods.

**Conclusion:** Improving the quality of care in ICU should also take into account the possible deleterious consequences of ICU stay. Follow-up is paramount in patients at risk, and increased efforts to improve quality of life after ICU discharge are needed.

### CO-08 An eye tracking adaptation of the Montreal-Toulouse aphasia test (MT-86) for intensive care patients to evaluate comprehension

#### Charuel Tessa^1^, Papazian Antoine^1^, Persico Lucille^1^, Aubrey Aurelie^1^, Barougier Anouck^1^, Ehrmann Stephan^1^, Bodet-Contentin Laetitia^1^

##### ^1^CHU de Tours, France

###### **Correspondence:** Charuel Tessa - t.charuel@yahoo.fr

*Annals of Intensive Care* 2018, **8(Suppl 1):**CO-08

**Introduction:** In intensive-care units, intubated patients, who can’t speak and patients unable to designate because of physical weakness are strongly limited for communicating with relatives and caregivers, despite cognitive function which may be preserved. There is a lack of validated tools to assess oral comprehension, adapted to those patients. Outside of the critical care setting the Montreal-Toulouse aphasia test (MT-86) is a validated tool to assess language and communication capabilities. It includes an evaluation of the lexical and syntaxic comprehension. This preliminary study aimed to adapt the oral MT-86 to overcome physical limitations of intensive-care unit patients using eye-tracking technology.

**Patients and methods:** A simplified version of the comprehension tasks from the MT-86, assessing the receptive lexicon and syntax was used. 47 item slides (2 or 4 pictures) were digitalized and integrated into an 8 min automated protocol, presented on a computer screen. The patient’s ocular fixation on pictures, when instructed to do so (human voice), was recorded by an eye-tracking device (see Fig. 1, patient instructed to “look at the button”). For patients regaining capability, the MT-86 paper version was performed.

**Fig. 1:**
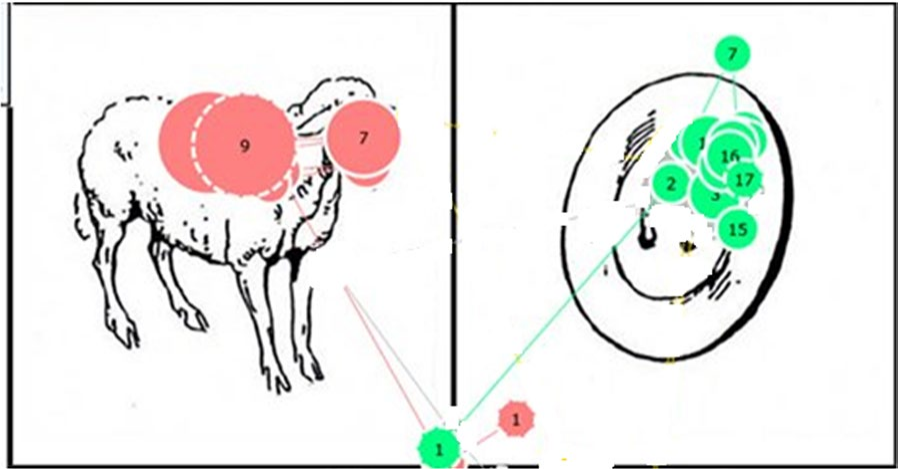
The figure presents 2 patients’ ocular fixation (each color corresponds to one patient). Green patient answered successfully and the total fixation duration was the most important for the area of interest “button”. Pink patient ocular fixation answered not successfully

**Results:** 11 control subjects (21–70 years old, 2 men 9 women) were used for device development which enabled to defining the response validation criterion after 3.5 s of fixation on the instructed picture. 19 non-intubated patients (12 women 7 men, 24–84 years old) were tested with both versions, and 4 intubated patients with the eye-tracking version. No side effect was noted. 4 types of responses were observed: response validation, sporadic fixation time (no choice), shortened fixation time (low awareness), no fixation. Two patients interrupted the test in both versions, at the same point, demonstrating the same resource cost from both modalities. The Fig. 1 presents 2 patients’ ocular fixation (each color corresponds to one patient). Green patient answered successfully and the total fixation duration was the most important for the area of interest “button”. Pink patient ocular fixation answered not successfully (phonological distractor).

**Conclusion:** This study shows feasibility of passing the MT-86 using eyetracking technology among critically ill patients. The tool appeared easy to use thanks to its brevity and automation. Further development may provide intensive care unit healthcare workers with a new device to assess comprehension and attention capacities of patients. This study may provide a new evaluation tool for patients among whom classical tests cannot be conducted.

### CO-09 Impact of obstructive sleep apnea syndrome on ICU patient prognosis: analysis of a French ICU cohort

#### Bailly Sebastien^1^, Seiller Alexandre^2^, Ruckly Stéphane^3^, Galerneau Louis Marie^1^, Terzi Nicolas^1^, Schwebel Carole^1^, Neuville Mathilde^4^, Tamisier Renaud^1^, Mourvillier Bruno^4^, Pépin Jean Louis^1^, Timsit Jean-François^4^

##### ^1^CHU de Grenoble, Grenoble, France; ^2^INSERM, Paris, France; ^3^INSERM, Paris, France; ^4^CHU Bichat, Paris, France

###### **Correspondence:** Bailly Sebastien - sbailly@chu-grenoble.fr

*Annals of Intensive Care* 2018, **8(Suppl 1):**CO-09

**Introduction:** Patients admitted in ICU have frequently several chronic diseases, including obstructive sleep apnea syndrome (OSAS). To date, OSAS was not robustly considered as a determinant of ICU patient prognosis. The objective of the study was to assess the impact of OSAS (known at admission) in patient prognosis of ICU patients.

**Patients and methods:** Data were retrospectively collected between 2006 and 2013 from two centers of a French national prospective database. A nested exposed-unexposed design was used. The OSAS status was checked for exposed patients using the hospitalization files. Adult patients without therapeutic limitation and staying alive in ICU at least one day were included. OSAS patients were matched (1 − n, n > 2) with non-exposed patients on body mass index, age and presence of chronic obstructive pulmonary disease (COPD). The sample size was computed to reach a power upper than 90% to show a one-day increase of ICU stay (± 4 days). The quality of matching was assessed using the standardized mean differences. The following outcomes were considered: length of stay alive in ICU, ICU mortality, in-hospital mortality, ventilator-associated pneumonia (VAP).

**Results:** From 5146 patients admitted in both ICUs and included in the study, 288 had a OSAS at ICU admission (5.6%). OSAS patients were mainly male (71 vs 60%), were older (64 years ± 12 vs 59 ± 17), had a higher BMI (29 ± 11 kg m^−2^ vs 22 ± 4 kg m^−2^), have a more frequent COPD status (19.4 vs 8.2% p < 0.01) and cardiovascular diseases (35.7 vs 20.5%), had a higher SOFA score at ICU admission (6.6 ± 4 vs 5.2 ± 4.4). After matching (289 OSAS exposed vs. 571 OSAS non-exposed patients), there was no significant impact of OSAS on the length of stay alive in ICU (adjusted RR: 1.09–95% confidence interval: [0.88; 1.36], p = 0.42). Sensitivity analyses showed no differences in the results. Using logistic conditional regression models, the impact of OSAS on ICU mortality, in-hospital mortality and VAP were not different between OSAS and unexposed patients. Limitation—under-diagnosed OSAS for non-exposed patients and the presence of unmeasured confounders in the matching process are major limitations of the study.

**Conclusion:** In a large cohort of ICU patients, obstructive sleep apnea syndrome do not impact patient’s prognosis and outcome.

### CO-10 Crush induction traceability in an ICU dedicated Patient Data Management System (PDMS), 1 year 21 ICU retrospective analysis

#### Goutorbe Philippe^1^, Cungi Pierrejulien^1^, Dupont Herve^2^, Payen Jean-François^3^, Radjou Aguila^4^, Choukroun Gerald^5^, Charpentier Julien^6^, Crova Philippe^7^, Schwebel Carole^3^, Lanceleur Anthony^8^, Slama Michel^2^, Luyt^9^, Charles-Edouard, Cordier Pierre-Yves^10^, Boutonnet Matthieu^11^, Pessey François^12^, Tran-Van David^13^, Patrigeon René-Gilles^14^, Ben Salah Adel^15^

##### ^1^Hôpital Sainte Anne, Toulon, France; ^2^CHU Amiens, Amiens, France; ^3^CHU Grenoble, Grenoble, France; ^4^CH René Dubos, Cergy Pontoise, France; ^5^CH Corbeil, Corbeil-Essonnes, France; ^6^CHU Cochin, Paris, France; ^7^CH Bourgoin-Jallieu, Bourgoin-Jallieu, France; ^8^Hopital Foch, Paris, France; ^9^CHU Pitié Salpetrière, Paris, France; ^10^HIA Laveran, Marseille, France; ^11^HIA Percy, Clamart, France; ^12^HIA Clermont Tonnerre, Brest, France; ^13^HIA Robert Picqué, Bordeaux, France; ^14^CH Auxerre, Auxerre, France; ^15^CH Chartres, Chartres, France

###### **Correspondence:** Goutorbe Philippe - philippegoutorbe@me.com

*Annals of Intensive Care* 2018, **8(Suppl 1):**CO-10

**Introduction:** Physicians in charge of critically ill patients deal with very high amount of data. Sources of data are as various as biology, bacteriology, monitoring, therapeutic or clinical inputs. ICU dedicated PDMS provides a quick scalable synthesis of this information. Besides computerized physician order entry (CPOE) seems to reduce medications errors in intensive care unit. It enhances traceability of continuous infusion, planed drugs or any items part of the care plan. But in some emergency cases, orally given orders may not be captured in the Medical file. We studied crush induction traceability in Centricity Critical Care (CCC), ICU dedicated solution, from GE Healthcare.

**Patients and methods:** We performed a retrospective anonymous database analyze of 2016 in 21 French ICU using CCC. We checked: the number of endotracheal intubations done in ICU (intubation 20 min or later after admission), the number of induction doses of rapid onset neuromuscular blockade agents (rocuronium or suxamethonium) at the same time (sampling only non-continuous perfusion). For each ICU, we asked if nurses were allowed to capture medications on behalf of physicians in the CPOE.

**Results:** During 2016, 19,736 patients were admitted, mean age was 60.68 mean SAPS II was 40.64 and length of stay was 6.69 days. We identified 5516 ICU intubations and found 909 rocuronium and 829 suxamethonium induction doses. The overall traceability ratio of induction was 31.51%. The traceability greatly varies between different ICU: individual ratio range from 1.32 to 83%. Third quartile was 51.55%. Traceability was better in ICU’s where nurses can capture medications in CPOE 48.7 versus 22.49% p < 0.02 (Fig. 1).

**Fig. 1:**
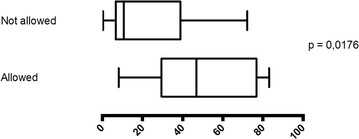
Traceability of prescriptions according to the nurse transcription right

**Conclusion:** Our study may suffer from 2 opposites bias: replacement of tracheal tube without anesthesia or use of bolus of rocuronium for another reason than intubation. Nevertheless, induction drugs seem to be greatly under written in the PDMS. Where nurses support, traceability is better. CCC allowed such database analyzes. Physician in charge of ICU should periodically, among others, check this indicator. Collaborative work with PDMS providers should keep enhancing traceability and safety. In our case, GE agreed in future software releases to add on top of “prescription” a “nurse note option” and when a nurse is adding a device such as endotracheal tube, tracheostomy or gastrostomy to insert a question: did you perform an anesthesia? Clinical PDMS databases should be great sources of knowledge and improvement areas only in the case of serious data collection.

### CO-11 Acute Respiratory Failure of patients with small-vessel vasculitis admitted to intensive care units: a multicenter retrospective study

#### Gibelin Aude^1^, Dumas Guillaume^2^, Valade Sandrine^3^, Pineton de Chambrun Marc^4^, Bagate François^5^, Neuville Mathilde^6^, Schneider Francis^7^, Baboi Loredana^8^, Groh Matthieu^2^, Raphalen Jean Herlé^9^, Guerin Claude^8^, Maury^10^, Eric, Timsit Jean François^6^, De Prost Nicolas^5^, Luyt Charles-Edouard^4^, Chiche Jean-Daniel^3^, Hertig Alexandre^1^, Parrot Antoine^1^, Azoulay Elie^2^, Fartoukh Muriel^1^

##### ^1^Hôpital Tenon, Paris, France; ^2^Hôpital Saint Louis, Paris, France; ^3^Hôpital Cochin, Paris, France; ^4^Hôpital Pitié Salpêtrière, Paris, France; ^5^Hôpital Henri Mondor, Paris, France; ^6^Hôpital Bichat, Paris, France; ^7^CHU de Strasbourg, France; ^8^Hôpital de la Croix Rousse, Lyon, France; ^9^Hôpital Necker, Paris, France; ^10^Hôpital Saint Antoine, Paris, France

###### **Correspondence:** Gibelin Aude - aude.gibelin@gmail.com

*Annals of Intensive Care* 2018, **8(Suppl 1):**CO-11

**Introduction:** The acute respiratory failure (ARF) of patients with known or de novo small-vessel vasculitis (SVV) admitted to the intensive care unit (ICU) may be due to immune or non-immune causes. The prognosis is poor. Early identification of an underlying immune disorder is essential to initiate appropriate treatment. The aim of the study was to determine the incidence, describe the clinical presentation and assess the prognosis of immune ARF related to SVV.

**Patients and methods:** This retrospective multicenter study was conducted from January 2007 to June 2017 in 10 ICUs in France. Patients were identified from computerized registers using either keys words research or the International Classification of Diseases, Tenth Revision (CIM10). Inclusion criteria were (1) admission to the ICU for ARF, and (2) known or de novo granulomatosis with polyangiitis, eosinophilic granulomatosis with polyangiitis, microscopic polyangiitis, or anti-glomerular basement membrane antibody disease. Demographics, characteristics of ICU admission, treatments and outcomes were extracted from medical records.

**Results:** During the study period, 130 patients (64 [51–75.5] years old + 63% male) were eligible. The SAPSII and SOFA scores were 39 [28–54] and 6 [4–8], respectively. A mixed cause of ARF was diagnosed in 22 patients (16.9%), which was secondary classified as a primary immune cause in 16 patients, and a primary non immune cause in 6 patients. Altogether, 67 patients (51.5%) had an immune ARF (n = 67), and 63 (48.5%) a non-immune ARF. Five etiologies accounted for the immune ARF group—diffuse alveolar hemorrhage (n = 47), pulmonary or bronchial granulomatosis (n = 13), acute exacerbation of interstitial lung disease (n = 11), asthma (n = 7), myocarditis (n = 4) and others (n = 2). In univariable analysis, the most relevant factors associated with immune ARF, as compared with non-immune ARF were: absence of chronic renal insufficiency (6 vs 40%; p < 0.0002) and dialysis (1 vs 25%; p < 0.0003), a short time between SVV diagnosis and ARF (0 [0–0] vs 48 months [5–132]; p < 0.0001) and the presence of specific extra respiratory symptoms (72 vs 27%; p < 0.0001). There was no major difference in prognosis between immune and non immune ARF patients (Table 1).

**Table 1:** Characteristics of 130 patients with known or de novo SVV (small-vessel vasculitis) admitted to the intensive care unit for Acute Respiratory Failure (ARF)

	All ARF (n = 130)	Immune ARF (n = 67)	Non immune ARF (n = 63)	p
*Demographics*				
Age, year	64 [51–75.5]	59 [44–72]	68 [59–76]	0.004
Male gender, n (%)	82 (63)	38 (57)	44 (70)	0.15
Cardiovascular disease^a^, n (%)	77 (59)	30 (45)	47 (75)	0.0007
Respiratory disease^b^, n (%)	60 (46)	18 (27)	42 (67)	< 0.0001
Immunosuppression^c^, n (%)	62 (48)	11 (16)	51 (81)	< 0.0001
Chronic renal disease, n (%)	29 (22)	4 (6)	25 (40)	< 0.0002
Chronic dialysis, n (%)	17 (13)	1 (1)	16 (25)	< 0.0003
*Small Vessels Vasculitis (SVV)*				
Granulomatosis with Polyangiitis (Wegener’s), n (%)	53 (41)	27 (40)	26 (41)	1
Microscopic Polyangiitis, n (%)	41 (32)	25 (37)	16 (25)	0.19
Eosinophilic Granulomatosis with Polyangiitis (Churg-Strauss vasculitis), n (%)	23 (18)	8 (12)	15 (24)	0.11
Goodpasture, n (%)	11 (8)	7 (10)	4 (6)	0.53
Others, n (%)	2 (2)	0 (0)	2 (3)	0.23
Time from SVV diagnosis to ICU admission, months	3 [0–60]	0 [0–0]	48 [5–132]	< 0.0001
*BVAS* (Birmingham Vasculitis Activity Score)	13 [0–21]	21 [15–25]	0 [0–6]	< 0.0001
*FFS* (Five factor score)	1 [0–2]	1 [0–2]	0 [0–1]	0.0002
*Reasons to ICU admission in addition to ARF, n (%)*				
Shock	22 (17)	7 (10)	15 (24)	0.06
Neurological	20 (15)	7 (10)	13 (21)	0.14
Cardiac arrest	6 (5)	3 (4)	3 (5)	Ns
*Time (days) between first respiratory symptoms and ICU admission, n (%)*				
<3 days	50 (38)	15 (22)	35 (56)	< 0.0001
3–10 days	31 (24)	18 (27)	13 (21)	0.42
>10 days	49 (38)	34 (51)	15 (24)	0.002
*Extra respiratory symptoms* ^*d*^ *on ICU admission, n (%)*	111 (85)	60 (90)	51 (81)	0.22
*Specific extra respiratory symptoms* ^*e*^ *on ICU admission, n (%)*	65 (50)	48 (72)	17 (27)	< 0.0001
*Severity criteria on ICU admission*				
SAPS II	39 [28–54]	37 [22–48]	45 [30–58]	0.009
SOFA	6 [4–8]	6 [3–8]	6 [4–9]	0.44

**Conclusion:** Half of the patients with a known or de novo SVV admitted to the ICU for ARF have an immune cause of ARF. Readily identified predictive factors of an immune cause could allow the introduction of an early and targeted treatment.

### CO-12 Anxiety in patients receiving non-invasive ventilation for acute respiratory failure: prevalence, risk factors and prognostic impact

#### Dangers Laurence^1^, Laforêt Jean Pierre^1^, Kouatchet Achille^1^, Jaber Samir^2^, Meziani Ferhat^3^, Perbet Sébastien^4^, Azoulay Elie^5^, Demoule Alexandre^5^

##### ^1^Hôpital Pitié-Salpêtrière, Paris, France; ^2^CHU de Montpellier, France; ^3^CHU de Strasbourg, France; ^4^CHU de Clermont-Ferrand, France; ^5^Hôpital Saint Louis, Paris, France

###### **Correspondence:** Dangers Laurence - laudangers@gmail.com

*Annals of Intensive Care* 2018, **8(Suppl 1):**CO-12

**Introduction:** Non-invasive ventilation (NIV) is a cornerstone therapy of acute respiratory failure (ARF) and is increasingly used in the intensive care unit (ICU). A recent survey suggests that up to 37% of patients report a certain level of anxiety related to NIV sessions. However, few is known on the actual prevalence, severity, risk factor and prognosis impact of anxiety in patients receiving NIV for an ARF. Objectives—to determine the prevalence and intensity of anxiety in patients receiving NIV for ARF, to identify the factors associated with anxiety, to assess the impact of anxiety on the risk of NIV failure, outcome, quality of life and post—ICU burden.

**Patients and methods:** Second analysis of a prospective observational cohort study in patients receiving NIV for ARF in 54 ICUs in France and Belgium, in 2010–2011. Anxiety was quantified using a graded ordinal scale from 0 (no anxiety) to 4 (very strong anxiety). A level of anxiety of 0 or 1 defined “mild or no anxiety” and a level of anxiety of 2, 3 or 4 defined a “moderate to severe anxiety”.

**Results:** Of the 419 patients included, 280 (67%) had mild or no anxiety and 139 (33%) had moderate to severe anxiety. Moderate to severe anxiety was independently associated with de novo ARF (OR 2.14; 95% CI 1.31–3.49; p = 0.002), male gender (OR 1.60; 95% CI 1.01–2.53; p = 0.047), dyspnea (OR 2.03; IC 95% 1.29–3.20; p = 0.002) and NIV intolerance (OR 4.9; 95% CI 3.03–7.93; p < 0.001). Factors of NIV failure were moderate to severe anxiety (OR 1.78; 95% CI 1.07–2.95; p = 0.027), SAPS 2 (OR 1.07; IC 95% 1.05–1.09) and de novo ARF (OR 3.30; IC 95% 1.97–5.55). In patients with moderate to severe anxiety, there was a trend toward a higher in-hospital (24 vs. 16%; p = 0.052) and day-90 mortality (29 vs. 20%; p = 0.05). Anxiety was not associated with higher length of stay, higher post ICU burden and altered quality of life.

**Conclusion:** Among patients receiving NIV for ARF, anxiety is frequent and potentially severe. Moderate to severe anxiety is associated with NIV failure and a trend toward higher mortality. Future studies should evaluate the benefit strategy aiming at improving anxiety management in ICU patients.

### CO-13 Initial ECCO2R experience in the great Paris area: rate of utilization and safety preliminary data

#### Augy Jean-Loup^1^, Aissaoui Nadia^1^, Richard Christian^2^, Maury Eric^3^, Fartoukh Muriel^4^, Mekontso-Dessap Armand^5^, Paulet Rémi^6^, Anguel Nadia^2^, Blayau Clarisse^4^, Cohen Yves^7^, Chiche Jean-Daniel^8^, Gaudry^9^, Stephane, Voicu Sebastian^10^, Demoule Alexandre^2^, Combes Alain^2^, Charpentier Emannuel^11^, Haghighat Suzanne^12^, Panczer Manuelle^12^, Diehl Jean-Luc^1^, Megarbane Bruno^10^

##### ^1^Hôpital Européen Georges-Pompidou, Paris, France; ^2^Hôpital de Bicêtre, Paris, France; ^3^Hôpital Saint Antoine, Paris, France; ^4^Hôpital Tenon, APHP, Paris, France; ^5^Hôpitaux Universitaires Henri Mondor, Paris, France; ^6^Centre Hospitalier Longjumeau, Paris, France; ^7^Hôpital Avicenne, Paris, France; ^8^Hôpital Cochin, Paris, France; ^9^Hôpital Louis Mourier, Paris, France; ^10^Hôpital Lariboisière, Paris, France; ^11^Office du Transfert de Technologie et des Partenariats Industriels, Paris, France; ^12^Agence Générale des Equipements et Produits de Santé, Paris, France

###### **Correspondence:** Augy Jean-Loup - augyjeanloup@hotmail.com

*Annals of Intensive Care* 2018, **8(Suppl 1):**CO-13

**Introduction:** Veno-venous extracorporeal CO_2_ removal (ECCO2R) is a promising new therapeutic option in the critical care setting. We conducted a prospective observational study of the use of ECCO2R in selected voluntary centers during 2 years aiming to assess the prevalence of the ECCO2R use mainly among COPD and ARDS patients.

**Patients and methods:** Two medical devices: Hemolung (Alung Technologies, Pittsburgh, USA) and iLA Activve (Xenios Novalung, Heilbronn, Germany) were selected after literature and medico-economic evaluations. A specific medical and nurses training was provided in each center. Data were collected on a dedicated form and were centralized by the coordinating center. Primary outcome was the number of patients treated per month and per center during the 2-years study period. Secondary outcomes were ICU and hospital-mortality and adverse events complications related to device use.

**Results:** We present preliminary results from 47 patients recruited in 6 centers (29 men, 18 women, mean age 66.9 yrs ± 11.3 yrs). The utilization rate was of 0.33 ± 0.32 patient month center. Thirty-nine patients were under invasive and 8 under noninvasive mechanical ventilation. Hemolung was used in 40 patients (65% in jugular site, cannula size: 15.5 F) and iLA Activve in 7 (71% in femoral site, cannula size: 24 F). Main indications were COPD AE (n = 22) and ARDS (n = 18). Eighteen patients were treated as a part of a clinical trial and 29 were treated as decided by the physician in charge according to current practice. Mean duration of ECCO2R was 5.4 ± 3.0 days. Twenty-five ECCO2R treatments were discontinued because of clinical condition improvement, 10 because of complications, 7 because of death and 5 for futility. Twenty hemolysis (either biological-free Hb > 100 µmol L or clinical), 15 hemorrhagic complications, 4 thrombosis, 1 cannula disinsertion, and 1 local hematoma occurred. Results according to the used medical devices are presented in Table 1. Twenty-one deaths occurred during ICU stay, 27 during the hospitalization, 3 of which in relation with ECCO2R.

**Table 1:** Results according to the ECCO2R medical device

Medical device	Hemolung (n = 40)	iLA Activve (n = 7)
Age (yrs): mean ± SD	66.7 ± 11.9	68.2 ± 7.6
Sex (men): n (%)	26 (65%)	3 (42%)
Indications		
COPD: n (%)	19 (47.5%)	3 (42%)
FEV1%th.: mean ± SD	43.8 ± 20.5	missing data
ARDS: n (%)	14 (35%)	4 (58%)
PaO_2_/FiO_2_: mean ± SD	157 ± 62	139 ± 45
Severe asthma: n (%)	4 (10%)	0 (0%)
Other: n (%)	4 (10%)	0 (0%)
Ending cause: n (%)		
Success	22 (55%)	3 (42%)
Adverse events	9 (22.5%)	2 (29%)
Death	5 (12.5%)	2 (29%)
Futility	4 (10%)	0 (0%)
Adverse events n (%)	26 (65%)	3 (42%)
Hemolysis	20	0
Clinical hemolysis	10	0
Bleeding	14	1
Thrombosis	2	2
Infection	0	1
Treatment of adverse events n (%)		
Surgery	1 (2.5%)	0 (0%)
Interventional radiology	2 (5%)	0 (0%)
Transfusion	9 (22.5%)	0 (0%)
In-ICU mortality: n (%)	18 (45%)	3 (42%)
In relation with ECCO_2_R	3 (7.5%)	0 (0%)

*COPD* chronic obstructive pulmonary disease, *FEV1* forced expiratory volume in 1 s, *ARDS* acute respiratory distress syndrome, *ECCO*_*2*_*R* extracorporeal CO_2_ removal

**Conclusion:** Our data indicate a preferential use of veno-venous ECCO2R devices in very severe (as illustrated by the overall high mortality) COPD and ARDS patients; with a lower than expected rate of utilization. Safety remains a major concern, indicating the need for further technological improvements as well of for optimization of anticoagulation regimen. Ultimately, RCTs will help to delineate clinical indications in these 2 main settings (COPD and ARDS).

### CO-14 Impact of an electronic monitoring device on maintaining a semirecumbent position in mechanically ventilated patients

#### Bouadma Lila^1^, Guillaume Sit^1^, Couffignal Camille^1^, Toni Alfaiate^1^, Arnaud Foucrier^1^, Mathilde Neuville^1^, Romain Sonneville^1^, Mourvillier Bruno^1^, Timsit Jean-François^1^

##### ^1^Hôpital Bichat, Paris, France

###### **Correspondence:** Bouadma Lila - lila.bouadma@bch.aphp.fr

*Annals of Intensive Care* 2018, **8(Suppl 1):**CO-14

**Introduction:** Maintaining a semirecumbent position (SRP) with a backrest elevation (BE) of 45° is not feasible for patients on mechanical ventilation (MV). Therefore, the effect of a SRP on the development of ventilator-associated pneumonia is uncertain. Our objective was to determine the efficacy of a simple electronic monitoring device to maintain a SRP during MV.

**Patients and methods:** We conducted a prospective, randomized, crossover study in two ICUs. BE of the patients were continuously assessed during a sequence of two consecutive 24 h-periods. Each period was either a control day = CD or a prototype day = PD. During the two periods, the targeted BE was between 40° and 45°. During the CD, BE was managed according to an internal procedure published previously. During the PD, an electronic device developed by our institution was added to the bed. The device was able to monitor continuously the BE and to alarm if the patient is not in the proper position. Adults patient were eligible if they were intubated with an expected duration of MV > 48 h and not eligible if they had a contraindication to SRP or a SAPS II score > 65. The time spent in a SRP defined by a BE between 40° and 50° was compared first in each crossover period by a Wilcoxon Rank-Sum test and then by a linear mixed effect model.

**Results:** One-hundred-five patients were randomized and a complete set of data were available for 103 patients: 72 men + age 59 ± 16 years, SAPS II score 48 ± 14 + acute respiratory failure in 28% and coma in 27%. BE was recorded for 833 ± 435 min during the CD and 1211 ± 333 min during the PD. During the two periods, patients had a median proportion of time spent in SRP about 30% higher in PD than in CD (IQR [25–75%]). In period 1 and period 2, patients had a higher median proportion of time spent in SRP in a PD than in a CD (93% [86–96%] vs 59% [34–79%], p < 0.0001) and (95% [78–97%] vs 62% [31–92%], p < 0.005) respectively. No period effect was identified. No adjustment covariate was significantly associated with the proportion of time spent in the SRP.

**Conclusion:** In ICUs with a high proportion of patients in a SRP (about 60%), a simple removable, washable, easily sanitized electronic monitoring device is able to raise this proportion at about 95%. Prospective studies are required to assess its clinical impact.

### CO-15 Assessment of proportional assist ventilation in patients exhibiting refractory ineffective triggering during pressure support ventilation

#### Haudebourg Anne-Fleur^1^, Maraffi Tommaso^1^, De Prost Nicolas, Razazi Keyvan, Mekontso Dessap Armand^1^, Carteaux Guillaume^1^

##### ^1^Hôpital Henri Mondor, Paris, France

###### **Correspondence:** Haudebourg Anne-Fleur - annefleur.haudebourg@gmail.com

*Annals of Intensive Care* 2018, **8(Suppl 1):**CO-15

**Introduction:** Ineffective triggering is frequent during pressure support ventilation (PSV). Its occurrence is favored by dynamic hyperinflation that may arise when increasing the pressure support level (PSL). Decreasing the PSL however fails to suppress ineffective triggering in a subgroup of patients that are therefore exposed to refractory ineffective triggering. Proportional assist ventilation with load-adjustable gain factors (PAV +) decreases the incidence of ineffective triggering in unselected patients but its effect on refractory asynchrony during PSV is unknown. The main aim of our study was to assess the effect of PAV + in patients exhibiting refractory ineffective triggering during PSV.

**Patients and methods:** Refractory ineffective triggering was defined as persisting ineffective triggering at the lowest tolerated PSL. Patients with refractory ineffective triggering during PSV were included. Flow, airway and esophageal pressures were continuously recorded during 10 min under PSV with the minimal tolerated PSL, and then under PAV + with the gain adjusted to target a muscle pressure between 5 and 10 cm H_2_O. Primary endpoint was the comparison of asynchrony index between PSV and PAV + . Continuous data are reported as median [25^th^–75th percentiles].

**Results:** Seven patients with refractory ineffective triggering were included so far. The median lowest tolerated PSL was 16 cm H_2_O [12–18], with a PEEP level of 8 cm H_2_O [5.5–8] and a PaO_2_ FiO_2_ ratio of 209 mmHg [178–225]. The median gain during PAV + was 70% [63–73]. The asynchrony index was significantly lower with PAV + than PSV (3% [2–8] vs. 24% [13–48] respectively, p = 0.02). Moreover, the asynchrony index decreased in every patient with PAV + (Fig. 1). Noticeably, the tidal volume was already protective in PSV and decreased even more during PAV + (5.71 mL kg [4.6–9.3] vs. 5.71 mL [1.8–7.1] respectively, p = 0.03); and the neural respiratory rate was high in both modes (41 cycles min [26–51] in PSV vs. 40[28–44] in PAV + , p = 0.61). Total esophageal Pressure–Time Product (PTPes) did not significantly differ between the two modes but the PTPes proportion that was wasted in ineffective efforts decreased with PAV + (1% [1–2] vs. 18% [7–37], p = 0.03).

**Fig. 1:**
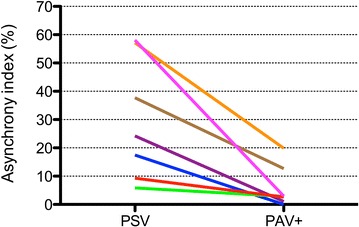
Asynchrony index during PSV and PAV+

**Conclusion:** Our preliminary data suggest that: (1) PAV + reduces the incidence of refractory ineffective triggering; (2) patients exposed to refractory ineffective triggering during PSV seem characterized by rapid shallow breathing despite high ventilatory support, questioning the tolerance of both ventilatory modes. Results with further inclusions will be presented.

### CO-16 Should we perform an immediate coronary angiogram in all patients after cardiac arrest?

#### Bougouin Wulfran^1^, Dumas Florence^1^, Karam Nicole^1^, Maupain Carole^1^, Marijon Eloi^1^, Lamhaut Lionel, Jost Daniel^1^, Geri Guillaume^1^, Beganton Frankie^1^, Varenne Olivier^1^, Spaulding Christian^1^, Jouven^1^, Xavier, Cariou Alain^1^

##### ^1^Hôpital Cochin, Paris, France

###### **Correspondence:** Bougouin Wulfran - wulfran.bougouin@gmail.com

*Annals of Intensive Care* 2018, **8(Suppl 1):**CO-16

**Introduction:** An immediate coronary angiogram (CAG) may be associated with better outcome after out-of-hospital cardiac arrest (OHCA) in neurologically preserved patients but could be futile in other cases. We aimed to assess the relationship between an immediate invasive strategy and survival after an out-of-hospital cardiac arrest (OHCA) of presumed cardiac cause, according to prognosis evaluated on hospital arrival.

**Patients and methods:** From May 2011 to May 2015, we collected data for all patients admitted in hospital after OHCA in Paris and suburbs (France). Risk of in-hospital death was retrospectively calculated using the validated Cardiac Arrest Hospital Prognosis (CAHP) score. Independent predictors of survival at discharge (including immediate CAG) were assessed in multivariate logistic regression in each of the 3 pre-defined subgroups of CAHP score: low (< 150 points), medium (150–200 points) and high (> 200 points) risk for in-hospital death.

**Results:** 1410 patients were included and overall survival rate at hospital discharge was 32%. Distribution in the low, medium and high-risk CAHP subgroups was 667 (47%), 469 (33%) and 274 patients (20%) respectively. The rate of early CAG was 86, 66 and 47% in the low, medium and high-risk subgroups, respectively (p < 0.001). Early invasive strategy was independently associated with better survival in low-risk patients (OR 2.3, 95% CI 1.4–3.9, p = 0.001), but not in medium (p = 0.55) and high-risk (p = 0.43) patients. Sensitivity analysis found consistent results.

**Conclusion:** In cardiac arrest patients, our results suggest that the potential benefit of an early coronary angiogram (and subsequent revascularization) is restricted to those with a preserved neurological prognosis at hospital arrival.

### CO-17 Unexpected cardiac arrest in the ICU: intermediate results of an ongoing multicenter study (ACIR)

#### Leloup Maxime^1^, Langlois Alice^1^, Briatte Isabelle^1^, Faucher Eric^1^, Bedoussac Emilie^1^, Lesieur Olivier^1^, Acir Study Group^1^

##### ^1^Groupe hospitalier La Rochelle Ré Aunis, La Rochelle, France

###### **Correspondence:** Leloup Maxime - leloup.mx@gmail.com

*Annals of Intensive Care* 2018, **8(Suppl 1):**CO-17

**Introduction:** To our knowledge, no study mapped the epidemiology of unexpected cardiac arrest (i.e. with resuscitation attempt) in French ICUs. The current research primarily aims at describing the demographics, management, and prognosis of patients concerned.

**Patients and methods:** “ACIR” (in French: Arrêt Cardiaque Inattendu en Réanimation) is a prospective multicenter study that started on January 1st 2017, and is expected to last 1 year. All victims of cardiac arrest during ICU stay are included, whereas patients presenting with cardiac arrest at the time of admission are not. By convention, any resuscitation attempt (chest compression, adrenaline and or electric shock) defines the “unexpected cardiac arrest”. The data collected comprise medical history, circumstances of happening, ongoing treatments, resuscitation maneuvers, and outcome). A 6-month follow up period is planned for survivors.

**Results:** Forty-four centers (17 University and 27 general hospitals) are participating in the study, including 8 medical, 7 surgical and 29 medical surgical ICUs. Among those, nearly one-third have implemented a specific protocol regarding the management of cardiac arrest. Around half undergo regular resuscitation trainings, and among them 80% do so at least annually. Following unexpected cardiac arrest, most of ICU staffs set up post hoc debriefings (always 32%, often 20%, sometimes 41% or never 7%). On two-thirds of the investigation period, 2842 patients were victim of cardiac arrest in the participating units. Of those, 2426 did not endure any resuscitation attempt: 1687 were previously attributed a “do-not-resuscitate” order, 589 were unresponsive to maximal therapy and 150 for other reasons. Four hundred and sixteen patients (69% men, 68 ± 13 years, mean SAPS2 72 ± 25) were included in the “unexpected cardiac arrest” chart. Half of the events occurred within the first 24 h from admission. Return of spontaneous circulation was achieved in 271 (66%) patients (67% men, 68 ± 13 years, mean SAPS2 70 ± 25), and among them 69 (25%) were discharged alive from hospital (62% men, 64 ± 15 years, mean SAPS2 64 ± 24).

**Conclusion:** According to the Utstein style, our intermediate data are within the range reported in relevant literature. Although initial resuscitation was successful in two-thirds of cases, only 16.5% of victims of unexpected cardiac arrest in the ICU were discharged alive from the hospital. Among other concerns, this study aims at determining among participating centers what specific interventions are likely to enhance prognosis and quality of life for cardiac arrest patients in the ICU.

### CO-18 Venous-to-arterial carbon dioxide difference (cv-art CO_2_ gap): prognosis factor of septic shock patients according to central venous oxygen saturation (ScvO_2_)

#### Ronflé Romain^1^, Lefebvre Laurent^2^, Garrigues Bernard^2^ Lehingue Samuel^1^, Duclos Gary^1^, Papazian Laurent^1^, Leone Marc^1^

##### ^1^Hôpital de La Timone, Marseille, France; ^2^CH d’Aix En Provence, France

###### **Correspondence:** Ronflé Romain - romainronfle@hotmail.fr

*Annals of Intensive Care* 2018, **8(Suppl 1):**CO-18

**Introduction:** Venous-to-arterial carbon dioxide difference (cv-art CO_2_ gap) is a marker of tissue perfusion. Despite being in the security zone, high central venous oxygen saturation (ScvO_2_) is associated with poor outcome in patients with septic shock. The aim of this study is to assess the ability of the cv-art CO_2_ gap to predict clinical worsening in patients with septic shock, according to ScvO_2_.

**Patients and methods:** We performed a multicentric prospective study in 3 ICUs. All consecutive patients with septic shock were included during the study period. Patients were assigned into three groups according to ScvO_2_. Clinical worsening was defined as an increase of SOFA score ≥ 1 two days after admission ∆SOFA ≥ 1). Patients characteristics and severity scores were collected during the first 3 days of stay. To assess the ability of the cv-art CO_2_ gap to predict a clinical worsening, a ROC curve was produced for the event ∆SOFA ≥ 1. Associations between cv-art CO_2_ gap and 28-day mortality along with length-of-stay (LOS) were explored using linear regression and correlations.

**Results:** Fifty-six patients were included. 28-day mortality was 23% with an admission mean SOFA score of 9 ± 3. On admission, mean ScvO_2_, blood lactate and cv-art CO_2_ gap were 74 ± 13%, 3.4 ± 2.8 mmol L and 6 ± 4.5 mmHg, respectively. Considering all patients, cv-art CO_2_ gap failed to predict ≥ 5.7 in the [70–80%] ScvO_2_ group could predict clinical worsening (Se = 67%, Sp = 75%, PPV = 57%, NPV = 82%, AUC = 0.7). In addition, every increase of 1 mmHg in cv-art CO_2_ gap might extend the LOS by 1.8 day (95% CI [0.2–3.4], p = 0.025). Finally, 28-day mortality of patients with mean cv-art CO_2_ gap > 6 mmHg within 72 h was 39% compared with 16% for patients with cv-art CO_2_ gap > 6 mmHg (p = 0.056).

**Table:** Table Hermodynamic and biological characteristics of studied patients according to ΔSOFA

Variable	Cohort (n = 52)	ΔSOFA ≥ 1 (n = 13)	ΔSOFA < 1 (n = 39)	*p*
IGS2 score	54 ± 18	64 ± 14	53 ± 17	0.06
Admission SOFA score	9 ± 3	9 ± 4	9 ± 2	0.54
28 days mortality n (%)	12 (23)	8 (67)	4 (33)	0.001
Mean MAP 72 h (mmHg)	77 ± 9	72 ± 8	80 ± 9	0.016
Mean Lactate 72 h (mmol/l)	3.2 ± 2.3	4.5 ± 3.1	2.5 ± 1.7	0.008
Mean ScvO_2_ 72 h (%)	74 ± 8	74 ± 8	73 ± 7	0.5
Mean cv-art CO_2_ gap 72 h (mmHg)	5.6 ± 2.1	5.9 ± 1.3	5.5 ± 2.3	0.23
∆ Lactate H0–H6 (mmol/l)	0.15 ± 1.2	− 0.07 ± 1.1	− 0.17 ± 1.2	0.87
Mean diuresis 24 h (ml)	1600 ± 1400	1111 ± 1647	1800 ± 1273	0.01
Mean fluid 24 h (ml)	3935 ± 3237	4091 ± 4176	3880 ± 2915	0.85
Mean fluid balance 24 h (ml)	3566 ± 2860	5498 ± 4237	3039 ± 2157	0.04
Mean ICC 24 h (l/min/m^2^)	3.34 ± 0.94	3.4 ± 0.8	3.4 ± 1	0.9
Mean admission EVLW (ml/Kg)	11 ± 4	10.9 ± 3.4	11.5 ± 4.6	0.84
Mean PaO_2_ 72 h (mmHg)	96 ± 20	98 ± 23	94 ± 19	0.72
Mean PaCO_2_ 72 h (mmHg)	41 ± 10	42 ± 5	40 ± 11	0.07
Mean PaO_2_/FiO_2_ 24 h (mmHg)	191 ± 73	181 ± 67	195 ± 75	0.54
PaO_2_/FiO_2_ < 150 (mmHg) n (%)	14 (29)	3 (22%)	11 (78%)	0.73
Mean noradrenaline 72 h (μg/kg/min)	0.44 ± 0.56	0.85 ± 0.95	0.28 ± 0.20	0.0001
Precharge dependance n (%)	35 (71)	7 (20)	28 (80)	0.28

**Conclusion:** cv-art CO_2_ gap is a parameter to assess the inadequacy of circulating blood flow in response to metabolic demand. cv-art CO_2_ gap could help clinicians to identify septic shock patients with organ dysfunction despite normalization of ScvO_2_.

### CO-19 Reversible microvascular endothelial dysfunction during correction of ketoacidosis diabetes

#### Joffre Jérémie^1^, Bourcier Simon^1^, Hariri Geoffroy^1^, Miaihle Arnaud Felix^1^, Bige Naike^1^, Dumas Guillaume^1^, Guidet Bertrand^1^, Maury Eric^1^, Ait Oufella Hafid^1^

##### ^1^Hopital Saint Antoine, Paris, France

###### **Correspondence:** Joffre Jérémie - jeremie.joffre@aphp.fr

*Annals of Intensive Care* 2018, **8(Suppl 1):**CO-19

**Introduction:** Context—Metabolic acidosis is commonly observed in critically ill patients. Experimental studies suggested that acidosis by itself could impair vascular function but such hemodynamic effect has been poorly investigated in human. Objectives: To assess the relationship between metabolic acidosis severity and endothelial microvascular function.

**Patients and methods:** Design, settings and patients: Prospective monocenter study. All adult diabetes patients admitted in our medical ICU for ketoacidosis (pH < 7.35) were included. Endothelial microvascular response to acetylcholine iontophoresis was measured at admission (H0) and after correction of metabolic acidosis (H24).

**Results:** Thirty-nine patients with diabetes ketoacidosis were included, 68% (n = 18) were male, with a median age of 43 [31–57] years. At admission, endothelial function significantly correlated with acidosis severity, the deeper acidosis was the worst endothelial activity (R = 0.53, p < 0.001). Endothelial response was strongly depressed below a pH at 7.20 (AUC 1779 [740–3079] versus 12,944 [4874–21,596], p < 0.0001). At H24, after rehydration and insulin infusion, clinical and biological disorders were fully corrected. After acidosis correction, endothelial response improved more in patients with baseline deep acidosis (pH < 7.20) when compared to patients with mild acidosis (AUC + 453 [213–1470] versus + 121 [79–312]  %, p < 0.01).
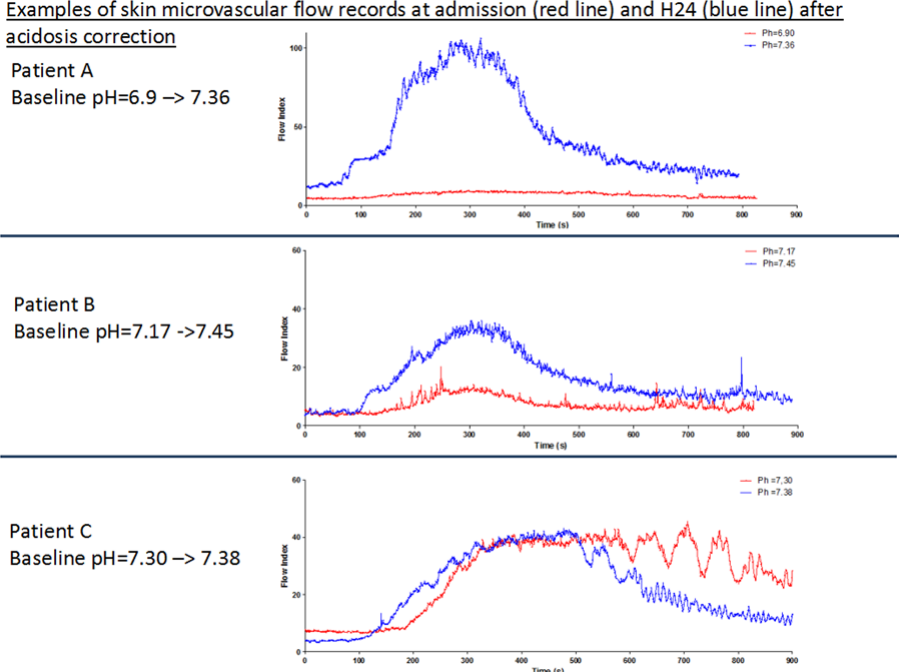


**Conclusion:** We documented an endothelial dysfunction during metabolic acidosis in critically ill patients with diabetic ketoacidosis. Endothelial dysfunction recovered after acidosis correction.

### CO-20 Intra-abdominal hypertension is responsible to false negatives to the passive leg raising test

#### Beurton Alexandra^1^, Teboul Jean-Louis^1^, Girotto Valentina^1^, Galarza Laura^1^, Richard Christian^1^, Monnet Xavier^1^

##### ^1^Le Kremlin-Bicêtre, Paris, France

###### **Correspondence:** Beurton Alexandra - alex.beurton@gmail.com

*Annals of Intensive Care* 2018, **8(Suppl 1):**CO-20

**Introduction:** The passive leg raising (PLR) test mimics a volume challenge and is based on the passive transfer of some venous blood from the legs toward the cardiac cavities when moving a patient from the semi-recumbent to a passive leg raised position. Nevertheless, intra-abdominal hypertension (IAH) may increase the resistance to venous return through the inferior vena cava and may impede the PLR-induced increase in cardiac preload. We tested the accuracy of the PLR test to predict fluid responsiveness in case of IAH.

**Patients and methods:** In patients with an intra-abdominal pressure (IAP) > 12 mmHg, we measured the changes in cardiac index (PiCCO2 device) induced by a PLR test and by a 500-mL saline volume expansion. The IAP (bladder pressure) was measured at different study steps.

**Results:** Twenty-nine patients were included, 20 being fluid responders (fluid-induced increase in cardiac index 15%) and 9 fluid non-responders. The IAP at baseline was 20 ± 6 mmHg. It significantly decreased during the PLR test in fluid responders (by 34 ± 13%) and in fluid non-responders (by 30 ± 16%). In fluid responders, cardiac index increased by 7 ± 9% during PLR and by 22 ± 6% during volume expansion. The PLR test was negative (PLR-induced increase in cardiac index < 10%) in 15 patients (false negatives) and positive in 5 patients (true positives). In fluid non-responders, cardiac index increased by 6 ± 6% during PLR and 6 ± 3% during VE. The PLR test was negative in all but one of them was positive (false positive). The sensitivity and specificity of the PLR test to detect fluid responsiveness were 40% (95% confidence interval 19–64) and 89% (52–100), respectively. The area under the receiver operating characteristic curve was 0.58 ± 0.11.

**Conclusion:** Intra-abdominal hypertension is responsible for false negatives to the PLR test. The PLR test significantly reduces IAP.

### CO-21 A prospective observational study reporting for acute mesenteric ischemia the results of the first 18 months of a dedicated intestinal stroke center for acute mesenteric ischemia

#### Nuzzo Alexandre^1^, Huguet Audrey^1^, Weiss Emmanuel^1^, Tran Dinh Alexy^2^, Maggiori Léon^1^, Iserentant Jules^1^, Pellenc Quentin^2^, Roussel Arnaud^2^, Sibert Annie^1^, De Blic Romain^2^, Billiauws Lore^1^, Ronot^1^, Maxime, Montravers Philippe^1^, Panis Yves^1^, Vilgrain Valerie^1^, Bouhnik Yoram^1^, Castier Yves Herve^2^, Paugam Burtz Catherine^1^, Corcos Olivier^1^

##### ^1^Hôpital de Beaujon, Clichy, France; ^2^Hôpital Bichat, Paris, France

###### **Correspondence:** Nuzzo Alexandre - al.nuzzo@gmail.com

*Annals of Intensive Care* 2018, **8(Suppl 1):**CO-21

**Introduction:** Acute mesenteric ischemia (AMI) has high mortality and intestinal resection rates. An intestinal stroke center based on a multimodal and multidisciplinary management focusing on intestinal viability was created in our center in January 2016. We aimed to study AMI patients and outcomes.

**Patients and methods:** Single-center, observational and prospective study was carried out in our intestinal stroke center. AMI was defined as an acute intestinal injury related to a splanchno-mesenteric insufficiency and without alternative diagnosis. All AMI patients underwent a computed tomography angiography. AMI were classified in occlusive (atheroma, thrombosis, embolisms etc.) and non-occlusive (splanchnomesenteric hypoperfusion, vasoconstrictive medications), arterial and venous. Patients with post-AMI short bowel syndrome (SBS), chronic mesenteric ischemia, aorta dissection anevrism and left ischemic colitis were not included. Outcomes studied were mortality at 3 months and at the end of follow-up, intestinal resection, SBS and parenteral nutrition requirement. Predictive factors of intestinal resection and mortality were studied in uni- and multivariate analyses. Quantitative data were reported as medians (range). A p value < 0.05 was considered to be significant.

**Results:** In 124 included patients (female 38%, 66.1 (26.3–95.6) yo) AMI was occlusive, non-occlusive, arterial, venous in 89, 11, 81 and 18.5%, respectively. Main etiologies of occlusive AMI were atheroma (39.5%), thrombosis (35%), embolism (9.7%). Occlusion concerned one, two or three of the digestive arteries in 54, 23 or 13%, respectively and superior mesenteric artery in 89.1% of arterial AMI. Chronic mesenteric ischemia preceded AMI in 22%. A surgical (n = 23) and or radiological (n = 36) revascularization was performed in 56.4% of arterial AMI. Intestinal resection was necessary in 31.4%. At end of follow-up of 290 (0–614) days, 22 (18%) patients had a SBS and 16 (13%) required parenteral nutrition. Mortality at 3 months and at the end of follow-up were 11.3 and 16.9%, respectively (Figure). In multivariate analysis a stay in ICU was the only factor associated with resection (p < 0.001, OR 7.9 (2.9–21.4)) and mortality (p = 0.02, OR 4.4 (1.2–15.4)).
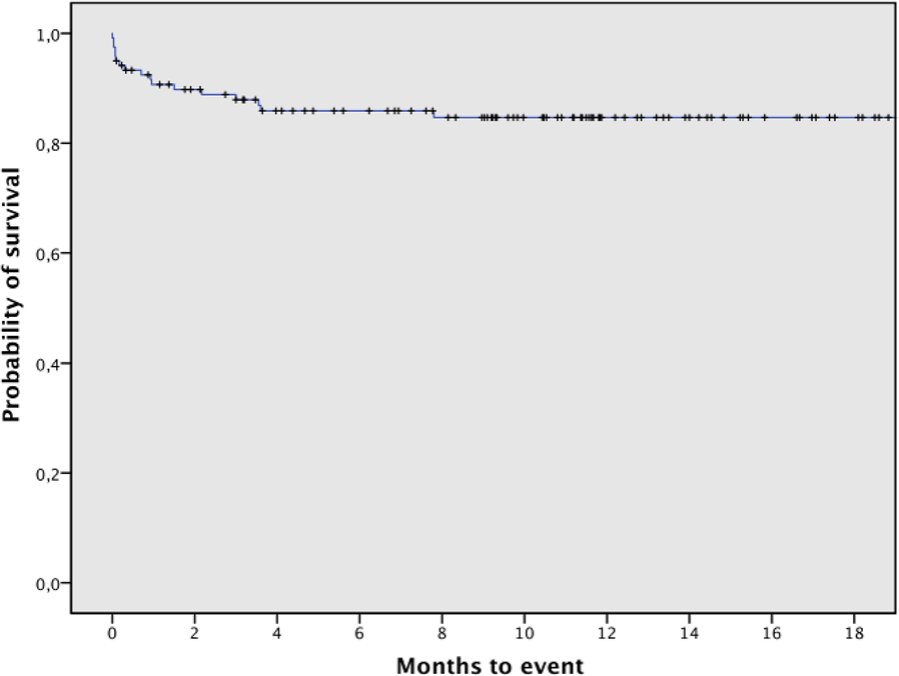


**Conclusion:** AMI patients treated in the intestinal stroke center have a survival and small bowel preservation rates of more than 80 and 70%, respectively. Such structure could serve as a model for multicentric studies and to improve at a large territorial scale the prognosis and the knowledge of AMI.

### CO-22 Incidence of intravascular catheter-related infections among patients undergoing prone positioning for Acute Respiratory Distress Syndrome: an exposed non-exposed study

#### Jacquot Audrey^1^, Novy Emmanuel^1^, Louis Guillaume^3^

##### ^1^CHU Nancy, France; ^2^CHR Metz Thionville, Ars-Laquenexy, France

###### **Correspondence:** Jacquot Audrey - audreyjacquo@hotmail.com

*Annals of Intensive Care* 2018, **8(Suppl 1):**CO-22

**Introduction:** The use of prone positioning (PP) for patients presenting moderate to severe Acute Respiratory Distress Syndrome (ARDS) has increased since the PROSEVA trial. Adverse events associated with PP have been well described such as pressure sores. But although patients with moderate to severe ARDS had high mortality, had prolonged length of stay and were particularly exposed to central venous catheters (CVC), there were no data reporting intravascular catheter-related infections (ICRI). In this study, we evaluated the incidence of CVC colonization in patients exposed to PP for ARDS.

**Patients and methods:** In this retrospective bicentric observational study we compared two groups of adults, assigned 1:1. The “exposed” group was composed of patients treated with PP for moderate to severe ARDS. The “non-exposed” group was composed of non-ARDS patients matched with: centre, year, sex, age, APACHE II score and length of ICU stay. ICRI incidence rate (1/1000 catheter-days) and preventive bundles were similar between the two centres, as well as the practice for PP session. The primary outcome was the incidence of catheter-related colonization in each group. The secondary outcome was the incidence of a composite criterion which evaluated an overall infectious risk associating colonization and/or bloodstream catheter-related infection and/or ICRI.

**Results:** Between January 1st, 2014 and December 31st, 2015, 173 patients were eligible for the “exposed” group: we matched 101 patients with 101 non-exposed patients. ARDS patients were mainly composed of direct ARDS (Pneumonia), median length of mechanical ventilation was 20 days, the average number of PP session was 2 and its mortality rate was 30%. “Non-exposed” group included a majority of cardiogenic shock. The number and length of catheter did not differ significantly between groups. The incidence of CVC colonization was 23.8% in the exposed group and 8.91% in the non-exposed group (p = 0.007) (Fig. 1). The odds ratio for PP was 3.8 (p = 0.01). The incidence of composite criterion was significantly higher in the exposed than in the non-exposed group (34% versus 19% p = 0.02).

**Fig. 1:**
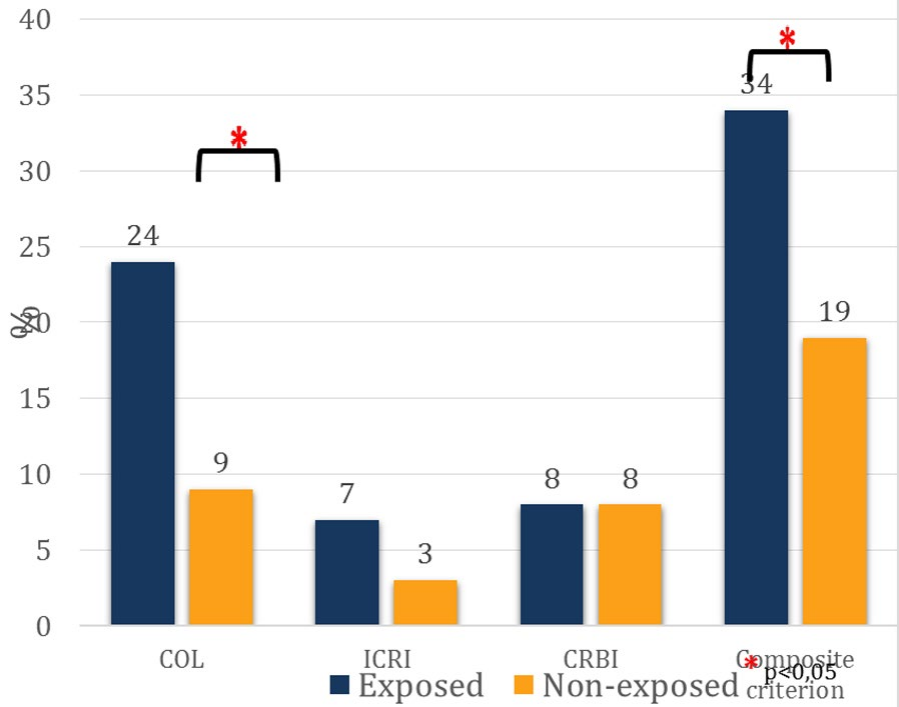
Incidence of colonization (COL), intravascular catheter-related infection (ICRI), catheter-related bloodstream infection (CRBI) and composite criterion (COL ± ICRI ± CRBI)

**Conclusion:** This is the first study to assess the incidence of ICRI in ARDS patients undergoing prone positioning. In our study, PP was associated with a higher rate of colonization. The overall catheter-related infectious risk was affected by the PP. We probably identified a population at high risk of ICRI who may benefit from additional preventive measures.

### CO-23 Low versus standard-blood-flow reperfusion strategy in experimental ischemic refractory cardiac arrest treated with Extra Corporeal Life Support

#### Levy Bruno^1^, Luo Yun^1^, Fritz Caroline^1^, Hammache Nefissa^1^, Kimmoun Antoine^1^, Grandmougin Daniel^1^, Orlowski Sophie^1^, Albuisson Eliane^1^, Tran N’Guyen^1^

##### ^1^CHRU NANCY, Vandoeuvre-Les-Nancy, France

###### **Correspondence:** Levy Bruno - blevy@sfr.fr

*Annals of Intensive Care* 2018, **8(Suppl 1):**CO-23

**Introduction:** This study was designed to assess the effect of two extracorporeal life support (ECLS) blood-flow strategy in an experimental model of E-CPR in the first six hours of resuscitation on macrocirculatory and microcirculatory parameters, lactate clearance and cytokine storm.

**Patients and methods:** Cardiac arrest was induced in 18 pigs by surgical ligature of the left coronary artery. ECLS was initiated after 40 min of cardiopulmonary resuscitation and the ECLS blood flow was set on 30–35 ml kg^−1^ min^−1^ (low-blood-flow group, LBF) versus 65–70 ml kg^−1^ min^−1^ (standard-blood-flow group, SBF) according to the randomized group. Continuous systemic blood pressure and carotid blood flow were continuously monitored. Blood gas analysis and lactate were measured at baseline H0, H3 and H6. Sublingual microcirculatory was assessed by sidestream dark field (SDF) technology and the following parameters were assessed: total and perfused vessels density (TVD, PVD), the proportion of perfused vessels (PPV) and microvascular flow index (MFI). Leg tissue oxygenation (StiO2) was monitored by a Near-Infrared Spectrometer (NIRS) device. Cytokine inflammatory was measured by enzyme-linked immunosorbent assay (ELISA).

**Results:** There was no differences between groups at baseline and at ECLS initiation (H0). Lactate and sublingual microvascular parameters were significantly impaired after the low-flow period. MAP target (65 mmHg) was reach in each randomized group. Total infused norepinephrine and total infused fluid were similar between the two groups. A significant difference was observed in the six hours evolution concerning carotid blood flow (LBF vs SBF at H6–19 [5–34.45] vs 67.81 [43.5–82] %, p < 0.05). Lactate clearance at H6 was inferior in the LBF compared with the SBF (6.67 [− 10.46 to 18.78] vs 44.72 [19.54–69.07] %, p < 0.05). Concerning the microvascular parameters, the LBF had lower PVD, PPV and MFI at H3 compared with the SBF but no significant difference observed at H6. TNF: was lower (361 [73–778] vs 1164 [177–1848] ng ml^−1^, p < 0.05) in the LBF at the end of the experiment.

**Conclusion:** In an experimental porcine model of refractory cardiac arrest treated by ECLS, a low-blood-flow strategy during the first six hours of resuscitation was associated with a decreased cerebral blood flow, lactate clearance and microcirculatory parameters despite a lower inflammatory response.

### CO-24 Hemodynamic efficacy of high permeability hemodialysis in post-cardiac arrest shock: results of the HYPERDIA randomized control trial

#### Geri Guillaume^1^, Grimaldi David^1^, Seguin Thierry^1^, Lamhaut Lionel^1^, Marin Nathalie^1^, Chiche Jean-Daniel^1^, Pène Frédéric^1^, Bouglé Adrien^1^, Daviaud Fabrice^1^, Morichau-Beauchant Tristan^1^, Arnaout Michel^1^, Champigneulle^1^, Benoit, Bougouin Wulfran^1^, Zafrani Lara^1^, Bourcier Simon^1^, Nguyen Yen-Lan^1^, Charpentier Julien^1^, Mira Jean-Paul^1^, Coste Joel^1^, Cariou Alain^1^

##### ^1^Hôpital Cochin, Paris, France

###### **Correspondence:** Geri Guillaume - guillaume.geri@aphp.fr

*Annals of Intensive Care* 2018, **8(Suppl 1):**CO-24

**Introduction:** After resuscitation of cardiac arrest (CA), an acute circulatory failure occurs in about 50% of cases, which shares many characteristics with septic shock. Most frequently, supportive treatments are unable to control this shock that may provoke multiple organ failure and death. We evaluated whether an early plasma removal of inflammatory mediators using high permeability hemodialysis (HPHD) in addition to conventional treatments could improve hemodynamic status of this patients.

**Patients and methods:** We performed a randomized open-label trial. Successfully resuscitated comatose CA patients who had a post-resuscitation shock (defined as requirement of norepinephrine or epinephrine infusion > 1 mg h) were included. The experimental group received 2 separated sessions of HPHD during the first 48 h following ICU admission. The control group received continuous veno-venous hemofiltration (CVVH) if needed. Non-parametric tests were used to compare the two groups. The primary outcome was the duration of the shock expressed by the length of catecholamine infusion. Number of vasopressors-free days, 6-h repeated measures of blood pressure, daily fluid balance and mortality (ICU and day-28) have been evaluated as well.

**Results:** 35 patients were included: 17 (median age 68.4, 59% male) in the HDHP group and 18 (median age 66.3, 83% male) in the control group. Baseline characteristics did not differ between the two groups. Ventricular fibrillation was the first recorded rhythm in 23.5 and 44.4% in the HDHP and control group, respectively (p = 0.289). Day-28 mortality rate was 64.7 and 72.2% in the HDHP and control group, respectively (p = 0.72). Probability of vasopressors discontinuation over time was similar in the two groups (Figure, p for logrank test = 0.67). Number of catecholamine-free days was 25.1 [0, 26.5] and 24.5 [0, 26.2] in the HDHP and control group, respectively (p = 0.65). No difference was observed regarding the daily-dose of vasopressors and the 6-h recorded systolic mean diastolic arterial pressure. No difference in terms of fluid balance was observed either.
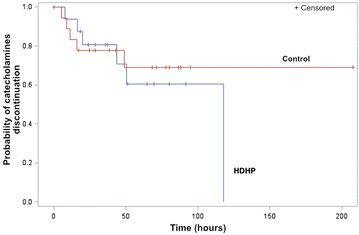


**Conclusion:** In post-cardiac patients with acute circulatory failure, HPHD did not reduced the duration of shock and had no effect on hemodynamic status. Registration: NCT00780299 the study was completely funded by the French Ministry of Health. Baxter provided the Septex membranes that were used in the HPHD group.

### CO-25 Usefulness of the Unyvero system to decrease broad-spectrum antibiotics consumption in patients with ventilator-associated pneumonia

#### Luyt Charles-Edouard^1^, Nicolas Bréchot^1^, Hékimian Guillaume^1^, Aubry Alexandra^1^, Lafeuille Emilie^1^, Schmidt Matthieu^1^, Franchineau Guillaume^1^, Besset Sébastien^1^, Nieszkowska Ania^1^, Bourcier Simon^1^, Coutrot Maxime^1^, Combes^1^, Alain

##### ^1^Hôpital de la Pitié-Salpêtrière, Paris, France

###### **Correspondence:** Luyt Charles-Edouard - charles-edouard.luyt@aphp.fr

*Annals of Intensive Care* 2018, **8(Suppl 1):**CO-25

**Introduction:** Reducing the use of broad-spectrum antibiotics in the ICU is a key issue. The P55 pneumonia cartridge of the multiplex-PCR Unyvero (Curetis) system allows identification of 19 bacteria and 1 fungi among the most frequently responsible for ventilator-associated pneumonia (VAP), and 19 of their resistance markers directly in clinical specimens. We aimed to evaluate the concordance between this technique and the conventional microbiological methods (CMM) for the diagnosis of VAP, assuming that it could support a decrease in broad-spectrum antibiotics consumption.

**Patients and methods:** Prospective, observational, single centre study. All consecutive patients with suspected VAP and a positive direct examination of broncho-alveolar lavage fluid (i.e. intracellular bacteria on direct examination) were included. Fresh BAL fluid was submitted to CMM and tested with the Unyvero system. We compared the results given by the 2 techniques, CMM being considered as the reference.

**Results:** Forty-four patients (median age 55 [43–63] yrs, median SAPS II score 54 [36–68]) were included. Microorganisms responsible for VAP were predominantly *P. aeruginosa* (Pa, n = 19) and Enterobacteriaceae (n = 21). 13 (30%) patients had polymicrobial VAP. The Unyvero system correctly identified pathogens in 35 (80%) patients + whereas no bacteria were detected for 4 patients, and a bacteria different that the one identified by CMM for 5 patients (Table 1). The Unyvero system failed to identify the resistance mechanism in 19 (43%) patients, either by default (n = 12) or excess (n = 7). These failures were mainly observed for Pa (n = 14, 74% of failures). In non-Pa VAP, the system failed to detect penicillinase in 2 patients with Enterobacteriaceae, and ESBL in 2 patients with E. coli VAP. Excluding Pa VAP, and assuming that the Unyvero system had been used for choosing initial empirical treatment, it could have saved a median of 2 [2–3] days of broad-spectrum antibiotics in 24 patients, but with a potential inappropriate initial antimicrobial treatment in 2 patients (2 with ESBL not detected).

**Table 1:** Discordance between the conventional cultures of BAL fluid and Unyvero system

	Organism identified by	
	Conventional microbiological methods	Unyvero
Patient 1	*E. aerogenes*, *K. pneumoniae*	*K. pneumoniae*
Patient 2	*K. pneumoniae*	No pathogen
Patient 3	*E. faecium*	No pathogen (*E. faecium* not on the panel)
Patient 4	*E. aerogenes*, *P. mirabilis*	*K. oxytoca*
Patient 5	*K. oxytoca*	No pathogen
Patient 6	*Kluyvera ascorbata*	*E. cloacae*
Patient 7	*K. pneumoniae*	*E. coli*
Patient 8	*S. aureus*, *A. baumannii*	*A. baumannii*
Patient 9	Polymicrobial culture	No pathogen

**Conclusion:** A strategy based on direct examination of BAL fluid followed by the P55 cartridge when the former is positive allows rapid identification of pathogen in 80% of patients with VAP. Excluding Pa VAP, mechanism of resistance could be correctly detected in 83% of VAP patients. Interventional studies are warranted to test the usefulness of this technique in an antimicrobial stewardship program. Acknowledgment: Curetis GmbH provided the P55 cartridges.

### CO-26 Hemophagocytic lymphohistiocytosis in adult ICU patients: an epidemiological and clinical study

#### Sellami Walid^1^, Rafrafi Emel^2^, Ben Mrad Ines^2^, Hajjej Zied^2^, Bousselmi Radhouene^2^, Yengui Olfa^2^, Sammoud Walid^2^, Gharssallah Hedi^2^, Labbene Iheb^2^, Ferjani Mustapha^2^

##### ^1^Montefleury, Tunis, Tunisia; ^2^Hôpital Militaire de Tunis, Tunis, Tunisia

###### **Correspondence:** Sellami Walid - drsellamiwalid@yahoo.fr

*Annals of Intensive Care* 2018, **8(Suppl 1):**CO-26

**Introduction:** Hemophagocytic lymphohistiocytosis (HLH) is a rare yet life-threatening condition characterized by an inappropriate activation of lymphocytes and or histyocytes leading to an abnormal phagocytosis of blood cells. Prognosis and outcomes mainly depend on the precocity of diagnosis and specific treatment implementation. A few studies were interested in HLH occuring in ICU patients. The Purpose of the study was to describe epidemiological, clinical, paraclinical and therapeutic characteristics of HLH in ICU patients.

**Patients and methods:** It was a retrospective, descriptive and longitudinal study including 30 cases of HLH, assessed during a 5 years period in the intensive care unit. We included all patients who had evidence of hemophagocytosis in bone marrow smears (realized when HLH was suspected) and a H-score superior to 169.

**Results:** The mean age of our patients was 48.9 ± 17.6 years [17–80] with a female preavalence. Hypertension and diabetes were the most frequent comorbidities. Immunodepression was present in 5 patients. Shock and neurological disorders were the main causes of admission in ICU. Mean APACHE II and SOFA (admission) scores were respectively 22.6 and 8.2. Fever was the most common clinical presentation in SAM. The most common biological disorder was bicytopenia (anemia and thrombopenia). The mean H-score was 209.7 p. Hemodynamic and respiratory distress were the prevalent organ failures. Corticoïds and immunoglobuline were given respectively to 14 and 13 patients. Etoposid was taken by one patient. Infections were the largely predominant etiology of HLH with a clear prevalence of multidrug resistant bacterial infections. Mortality at day 28 was 40%. Septic shock was the leading cause of death. SOFA and APACHE II scores were the only predictive factors of mortality (p = 0.015 and p = 0.042).

**Conclusion:** Management a patient with HLH is challenging because of its rarity, its variable presentation and its association with a panel of disorders. A multidisciplinary approach is mandatory to determine the best therapeutic option for the patient.

### CO-27 Effects of red blood cell transfusion on global oxygenation in anemic critically ill patients

#### Themelin Nicolas^1^ Biston Patrick^1^, Massart Jacqueline^1^, Piagnerelli Michael^1^

##### ^1^CHU de Charleroi, Belgium

###### **Correspondence:** Themelin Nicolas - nicolas.themelin@ulb.ac.be

*Annals of Intensive Care* 2018, **8(Suppl 1):**CO-27

**Introduction:** Anemia and RBC transfusion as treatment are common in ICU patients. Neverthless, RBC transfusions are associated with increased morbidity and mortality. Restrictive strategy policy, based on a haemoglobin [Hb] level of 7 g dL, is the guideline to transfuse most of ICU patients. The aim of RBC transfusion is to avoid tissue hypoxia by improving oxygen delivery (DO2) and therefore oxygen consumption (VO2). This could suggest to combine [Hb] and some parameters reflecting the sytemic DO2 VO2 balance to decide to transfuse. This study aims to analyse the effects of RBC transfusion on the systemic oxygenation assessed by the (central) venous oxygen saturation (S(c)VO2), the lactate level, the venous-to-arterial carbon dioxyde pressure gradient (Pv-aCO2) and the ratio between cardiac index and O_2_ extraction (IC EO2), and to assess their usefulness in the transfusion decision.

**Patients and methods:** During 9 months, all adult patients transfused in the ICU of CHU-Charleroi Marie Curie were included except patients with active bleeding or without jugular or subclavicular catheter. The systemic oxygenation parameters have been measured before and after transfusion. Patients a priori have been grouped according to their initial S(c)VO2 (< or > 70%), to their cardiac (< or > 50% of a left ventricular ejection fraction) and septic status. The results are expressed in median and interquartile ranges. Comparisons were made by a Wilcoxon test.

**Results:** 86 RBC transfusions were made on 53 patients. For all patients, mean arterial pressure, [Hb] and S(c)VO2 increased significantly after transfusion ([Hb]: 7.4 [7–7.8] to 8.3 [7.7–8.8] g dL; MAP: 79 [69–90] to 82 [74–92] mmHg and S(c)VO2: 66 [60–73] to 69 [63–75] %; for all p < 0.001). Only patients with an initial S(c)VO2 < 70% improved it after transfusion (62 [58–65] to 66 [62–71] %; p < 0.001). The Pv-aCO2 was significantly higher in patients with an initial S(c)VO2 < 70% (8 [5–10] mmHg) but did not change after transfusion as [lactate] and IC EO2. These results are maintained whatever the cardiac or septic status.

**Conclusion:** A S(c)VO2 < 70% in anemic ICU patients compared to other systemic oxygenation parameters, seems to be useful to assess the transfusion requirement as indicator of a DO2 VO2 imbalance. It should be interesting to combine it with the [Hb] in the decision to transfuse anemic critically ill patient.

### CO-28 Elderly patients with cancer in the ICU

#### Sirjacques Camille^1^, Ameye Lieveke^1^, Berghmans Thierry^1^, Paesmans Marianne^1^, Sculier Jean-Paul^1^, Meert Anne-Pascale^1^

##### ^1^Institut Jules Bordet, Bruxelles, Belgium

###### **Correspondence:** Sirjacques Camille - Camille.Sirjacques@ulb.ac.be

*Annals of Intensive Care* 2018, **8(Suppl 1):**CO-28

**Introduction:** There is very little data on the survival of elderly patients with cancer requiring admission to intensive care unit (ICU). The aim of the study is to evaluate in our department prognostic factors for hospital mortality and survival after hospital discharge for patients aged more than 65 years.

**Patients and methods:** This is a retrospective study including all patients aged more than 65 with a solid or hematological tumor admitted for acute complication in an oncological ICU over a 3 years period. In case of multiple admissions only the 1st admission was used for the statistical analyses.

**Results:** We recorded 311 admissions for 270 patients over the 3 years period. The main reasons for admission were cardiovascular (22%), respiratory (17%) and hemodynamic (13%). ICU and in-hospital mortality rates were respectively 10% (95% CI 6–13%) and 22% (95% CI 17–27%). Median survival after discharge from hospital was 7.8 months (95% CI 5.7–11.3). The identified prognostic factors for higher hospital mortality were non invasive ventilation use (OR 9.8, 95% CI 3.3–28.5), invasive mechanical ventilation use (OR 8.6, 95% CI 3.4–21.9) and the existence of a therapeutic limitation in the first 24 h (OR 3.1, 95% CI 1.5–6.6) (assessment of all covariates was restricted to the period of 24 h following ICU admission). After hospital discharge, prognostic factors for higher risk of death were Charlson’s score bigger than 8 (HR 2.73, 95% CI 1.93–3.86), SAPS II score bigger than 37 (HR 1.50, 95% CI 1.08–2.09), administration of amines in the first 24 h (HR 2.13, 95% CI 1.03–4.38) and the existence of a life-sustaining therapeutic limitation in the first 24 h (HR 2.35, 95% CI 48–3.73). A total of 77% of patients were able to benefit from an antineoplastic treatment after hospital discharge.

**Conclusion:** 78% of oncologic patients aged more than 65 years were discharged alive from hospitalization after an ICU stay and the large majority were still able to benefit from cancer treatment. Life-sustaining therapeutic limitation is directly related to hospital mortality and post-discharge survival.

### CO-29 Clinical features and outcomes of central nervous system (CNS) infections in critically ill immunocompromised patients

#### Kerhuel Lionel^1^, Mariotte Eric^1^, Zafrani Lara^1^, Ghrenassia Etienne^1^, Ardisson Fanny^1^, Ekpe Kenneth^1^, Darmon Mickael^1^, Azoulay Elie^1^

##### ^1^Hôpital Avicenne, Paris, France

###### **Correspondence:** Kerhuel Lionel - lionel.kerhuel@aphp.fr

*Annals of Intensive Care* 2018, **8(Suppl 1):**CO-29

**Introduction:** Studies assessing outcomes of CNS infections in critically ill immunocompromised patients are scarce. We sought to describe clinical features and outcomes in this population.

**Patients and methods:** We conducted a retrospective observational study over a 13-year period (01, 2004–08, 2017). All patients admitted to the ICU, presenting with meningitis, encephalitis or brain abscess were included. All patients with the following diseases were considered: hematologic malignancy, solid tumor, solid organ transplantation, autoimmune disease and splenectomy. HIV patients were not included. Baseline characteristics and variables related to ICU admission, treatments, ICU outcomes and neurological status (Rankin scale 6 months after ICU discharge) were collected.

**Results:** Among the 182 patients admitted to our ICU for CNS infection, 64 were included. HIV patients (N = 45) and non-immunocompromised patients (N = 73) were not included. Among the 64 patients, 40 (63%) had hematological malignancies (in whom 6 received an allogeneic stem cell transplant), 8 (13%) solid tumors, 9 (14%) autoimmune diseases, 7 (11%) renal transplantations and 3 (5%) splenectomies. Thirty-four (53%) were male, with a median age of 55 [47; 64] years and a Performance Status of 1 [0; 2]. The main reason for ICU admission was mental confusion or coma (77%). Lumbar puncture was performed in 57 (89%) patients. CNS CT was performed in 51 patients and was abnormal in 14 (27%). Brain MRI was performed in 40 patients and was abnormal in 34 (85%). Meningitis accounted for 48% (n = 31), in whom 74% were microbiologically documented (Streptococcus pneumonia, Streptococcus spp. and Gram negative bacilli in 8, 3 and 3 cases, respectively). Encephalitis accounted for 31% (n = 20) of diagnoses, in whom 80% were microbiologically documented (tuberculosis, Herpes Simplex Virus and Human Herpes Virus 6 in 5, 3 and 3 cases, respectively). Brain abscess accounted for 20% (n = 13) of diagnoses, in whom 69% were microbiologically documented (Aspergillus and Nocardia in 4 and 3 cases, respectively). Median ICU length of stay was 7 [3; 18] days. Mechanical ventilation was performed in 35 (56%) patients. Vasopressors and renal replacement therapy were implemented in 25 (40%) and 2 (11%) patients respectively. ICU mortality was 32% (n = 20), and 25 (48%) patients were alive 6 months after ICU discharge with a good neurological status (Rankin < 4).

**Conclusion:** This study sheds light on clinical features and outcomes in immuncompromised patients with CNS infections. Determinants of mortality and comparative data across the main groups of CNS infections are being prepared.

### CO-30 Cardiac involvement in patients with severe thrombotic thrombocytopenic purpura (TTP)

#### Fourmont Aude-Marie^1^, Zafrani Lara^2^, Mariotte Eric^3^, Galicier Lionel^3^, Merceron Sybille^4^, Bertinchamp Remi^4^, Lemiale Virginie^3^, Darmon Michael^3^, Veyradier Agnes^3^, Azoulay Elie^3^

##### ^1^10, Paris, France; ^2^HU Est-Parisien, 10, Paris, France; ^3^Hôpital Saint Louis, 10, Paris, France; ^4^CH de Versailles, 10, Paris, France

###### **Correspondence:** Fourmont Aude-Marie - am.fourmont@gmail.com

*Annals of Intensive Care* 2018, **8(Suppl 1):**CO-30

**Introduction:** Cardiac involvement in patients with TTP is associated with high mortality. It might be undermined by the lack of operational definition and established diagnostic workup for cardiac TTP. The objectives of this study were to describe cardiac involvement in TTP patients and to assess prognostic value of each clinical sign.

**Patients and methods:** In a single center study, all adult patients admitted to our ICU between 2007 and 2017 with confirmed TTP (ADAMTS13 < 10%) were included and standardized diagnostic workup was performed. Cardiac signs included chest pain, changes in electrocardiogram, increase troponin level, new-onset changes in cardiac echography, cardiogenic shock, cardiac arrest.

**Results:** Among the 98 TTP patients (67 women, 65% non-Caucasian, age 43(32–53)), 28 had a cardiovascular comorbidity and 12 were HIV-infected. Half the TTP were idiopathic, 25 autoimmune, 5 drug-related and 4 surrounded pregnancies. At initial clinical evaluation organ involvement was present in all patients, including neurological (N = 77), cardiac (N = 76), renal (N = 50), or digestive (N = 32). All patients received urgent plasma therapy with a median number of plasma exchanges of 11(8–18) and all but 3 received steroids, including 28 who received bolus. 52 patients required a rescue therapy (49 rituximab, 21 vincristine, 4 cyclophopshamide, and 6 splenectomy). 68 patients required antihypertensive therapy, 24 mechanical ventilation, 18 vasopressors and 15 renal-replacement therapy. Cardiac involvement was observed in 89 (91%) patients. Namely, 23 patients exerted chest pain, 57 electrocardiogram changes, 70 (71%) increased troponin, and 6 cardiac arrest. Troponin was the only feature of cardiac involvement in 24 patients. In 13 patients, echocardiography identified focal or global hypokinesia. Anticoagulants were prescribed in 86 patients (58 prophylactic and 28 curative) and 88 patients received low dose aspirin. Hospital mortality was 9.4%. All the 9 patients without cardiac involvement survived. Cardiac involvement was associated with prolonged ICU stay (6 (4–7) vs. 8 (6–14), p = 0.04). By univariable analysis, factors associated with mortality included age, platelet count, status epilepticus, troponin level, cardiac arrest. Of striking finding, cardiac involvement of TTP was not associated with mortality or with adverse TTP outcome.

**Conclusion:** In patients with severe TTP, cardiac involvement is present in 90% of patients. In this cohort of patients receiving a second-line TTP therapy in half the cases, aspirin in 90% and anticoagulants in 88%, cardiac involvement was not associated with mortality suggesting that aggressive TTP management translates into improved outcomes.

### CO-42 Use of alternatives to carbapenems for the treatment of EBSL Gram-negative bacilli severe infections in the ICU

#### Besset Sébastien^1^, Hékimian Guillaume^1^, Bréchot Nicolas^1^, Franchineau Guillaume^1^, Coutrot Maxime^1^, Bourcier Simon^1^, Nieszkowska Ania^1^, Schmidt Matthieu^1^, Combes Alain^1^, Luyt Charles-Edouard^1^

##### Hôpital Pitié-Salpêtrière, Paris, France

###### **Correspondence:** Besset Sébastien - sebastien.besset@aphp.fr

*Annals of Intensive Care* 2018, **8(Suppl 1):**CO-42

**Introduction:** The use of alternatives to carbapenems to treat patients with extended-spectrum beta lactamase-producing Gram negative bacilli (ESBL-GNB) infections remains controversial. Their use in patients with severe infections in the ICU has been poorly studied. The aim of this study was to compare the outcome of ICU patients having received carbapenems to those having received a carbapenem-sparing agent (CSA).

**Patients and methods:** The charts of patients with ESBL-GNB infection hospitalized in our ICU between 2015 and 2017 were retrospectively reviewed. Patients treated with betalactam betalactam inhibitor (BL BLI), cefepime or quinolones were considered has having received an alternative to carbapenems (CSA). Patients having received such a CSA were compared to those having received a carbapenems. Primary outcome was treatment failure at day 28, defined as ESBL-GNB infection recurrence (relapse with same pathogen) or death, whichever first occurred.

**Results:** 66 patients with ESBL–GNB infection were included. Source of infection was the lung for most of them. Characteristics of patients are displayed on Table 1. Their median SAPS II and SOFA scores were 59 [44–71] and 12 [7–15], respectively, and 38 (58%) were on septic shock. 40 patients received a carbapenem empirically, among whom 18 were switched to a CSA agent when antibiogram was available (CSA-definite group), whereas carbapenems were pursued in the 22 others (carbapenem-only group), mainly because pathogens were resistant to others antibiotics. Among the 26 patients having received a non-carbapenem agent as empirical treatment, pathogen was susceptible to this agent in 9 and they pursued the same treatment (CSA-only group), whereas 17 were switched to a carbapenem (pathogens resistant to empirical treatment, carbapenem-definite group). Treatment failure were not different among these 4 groups (Table 1). Globally, 27 patients received a CSA as their definite treatment (CSA-definite and CSA-only groups), whereas 39 received a carbapenems (carbapenems-only and carbapenems-definite groups). Whereas duration of antimicrobial treatment was similar (8 [6–10] days vs. 9 [7–10] days, respectively, p = NS), treatment failure rate was not higher in the former, as compared to those having received a carbapenems (29 vs. 71%, respectively, p = 0.049).

**Table 1:** Characteristics and outcomes of patients according to their antimicrobial regimen

	All patients	Carbapenem-only	Carbapenem-definite	CSA-only	CSA-definite	p
	N = 66	N = 22	N = 17	N = 9	N = 18	
Age, years	57 [49–63]	54.5 [45–62]	61 [55–64]	61 [51–74]	55 [34–61]	NS
Female sex	46 (70)	8 (36)	3 (18)	1 (11)	8 (44)	NS
SAPS II score	58.5 [44–71]	58.5 [41–69]	58 [45.5–72.5]	66 [43.5–79]	54.5 [47–70]	NS
SOFA score	11.5 [7–15]	9 [6–15]	13 [7–16]	13 [4.75–14.25]	11.5 [7–16]	NS
Septic shock	38 (58)	10 (56)	4 (57)	10 (59)	14 (58)	NS
Immunodepression	21 (33)	7 (39)	1 (17)	7 (41)	6 (26)	NS
Site of infection						0.02
Lung	51 (77)	17 (77)	16 (94)	7 (78)	11(61)	
Primary bacteremia	12 (18)	4 (18)	1 (6)	0	7 (39)	
Other	4 (6)	1 (5)	0	2 (22)	0	
Treatment duration, days	8 [6–10]	7 [6–10]	9 [7–10]	7 [6–9]	8 [7–11]	NS
Treatment failure (mortality or relapse)	34 (52)	15 (68)	9 (53)	4 (44)	6 (33)	NS

Results are expressed as median [IQR] or n (%)

**Conclusion:** Treatment of patients with ESBL-GNB severe infection in the ICU with a CSA seems to be safe when the pathogen is susceptible to this CSA. However, MIC should be first determined before de-escalating to a CSA. Larger studies are needed.

### CO-43 Is augmented renal clearance responsible for subtherapeutic ß-lactam antibiotic concentrations and therapeutic failure in critically ill patients? A prospective observational study

#### Petit Laurent^1^, Carrié Cédric^1^, Cottenceau Vincent^1^, Lafitte Mélanie^1^, D’Houdain Nicolas^1^, Breilh Dominique^1^, Sztark François^1^

##### ^1^CHU PELLEGRIN, Bordeaux, France

###### **Correspondence:** Petit Laurent - laurent.petit@chu-bordeaux.fr

*Annals of Intensive Care* 2018, **8(Suppl 1):**CO-43

**Introduction:** This study aimed to assess whether augmented renal clearance (ARC) impacts negatively on beta-lactam antibiotic unbound concentrations and clinical outcome in critically ill patients.

**Patients and methods:** Over a 9-month period, all critically ill patients treated by beta-lactam antibiotics for a microbiologically documented infection without renal impairment were eligible. During the first 3 days of antimicrobial therapy, every patient underwent 24-h creatinine clearance (CrCL) measurements and therapeutic drug monitoring. The main outcome investigated in this study was the rate of therapeutic failure, defined as an impaired clinical response with a need for escalating antibiotics during treatment and or within 15 days after end-of-treatment. The secondary outcome was the rate of pharmacokinetic pharmacodynamics (PK PD) target non-attainment defined by at least one sample under four times MIC of the known pathogen (subexposure < 4.MIC).

**Results:** Over the study period, 96 patients were included. The rate of therapeutic failure was 16%. Mean CrCL values over the first three days of antimicrobial therapy were significantly higher in patients with therapeutic failure (203 ± 63 vs. 163 ± 68 ml min, p = 0.04). Subexposure < 4.MIC was independently associated with therapeutic failure with an OR at 5.8 (1.3–25.4), p = 0.02. Patients with mean CrCL > 200 ml min presented higher risk of subexposure < 4.MIC with an OR at 5.1 (1.8–14.4), p = 0.002. The percentages of samplings which attained the PK PD targets for various CrCL are presented Fig. 1.

**Fig. 1:**
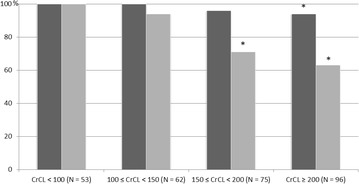
Percentages of samplings which attained the PK PD targets for various CrCL

**Conclusion:** Our results suggested that (1) mean CrCL values > 200 ml min were associated with higher rates of sub-exposure < 4.MIC and (2) sub-exposure < 4.MIC was associated with higher rates of therapeutic failure. This study emphasizes the need of therapeutic drug monitoring in patients with ARC, especially when targeting less susceptible pathogens or surgical infections with limited penetration of antimicrobial agents. Whether those patients should benefit for increased dosing regimens should be evaluated by randomized controlled studies.

### CO-44 Impact of renal replacement therapy strategies on beta-lactamine plasma concentrations: the BETAKIKI study: an ancillary study of a randomized controlled trial

#### Roux Damien^1^, Benichou Nicolas^1^, Hajage David^1^, Martin-Lefèvre Laurent^2^, De Prost Nicolas^3^, Lerolle Nicolas^4^, Maizel Julien^5^, Boulet Eric^6^, Mayaux Julien^7^, Mégarbane Bruno^8^, Mahjoub Khaoula^9^, Carpentier^10^, Dorothée, Nseir Saad^11^, Tubach Florence^1^, Ricard Jean-Damien^11^, Dreyfuss Didier^7^, Gaudry Stéphane^1^

##### ^1^Hôpital Louis Mourier, Colombes, France; ^2^CHU de La Roche-sur-Yon, France; ^3^Hôpital Henri Mondor, Créteil, France; ^4^CHU d’Angers, Angers, France; ^5^CHU Amiens, Amiens, France; ^6^Hôpital René Dubos, Pontoise, France; ^7^Hôpital Pitié-Salpêtrière, Paris, France; ^8^Hôpital Lariboisière, Paris, France; ^9^Hôpital Delafontaine, Saint-Denis, France; ^10^CHU de Rouen, Rouen, France; ^11^CHU de Lille, France

###### **Correspondence:** Roux Damien - damien.droux@gmail.com

*Annals of Intensive Care* 2018, **8(Suppl 1):**CO-44

**Introduction:** Critically-ill patients often receive antimicrobials. Early adequate antibiotic administration, usually including beta-lactams, improves prognosis of septic patients. However, beta-lactam pharmacokinetic as well as effect of renal replacement therapy (RRT) on beta-lactam concentrations have been hardly explored in ICU patients with acute kidney injury (AKI). We aimed to determine factors associated with potential suboptimal beta-lactam concentration in critically-ill patients with AKI treated either with an early or a delayed RRT strategy.

**Patients and methods:** Ancillary study in a subset of patients with severe AKI (KDIGO3), receiving a beta-lactam antibiotic, in a trial comparing two RRT initiation strategies. In this trial, patients from 31 intensive care units were randomly assigned to either an early (immediate RRT) or a delayed (late or no RRT) RRT initiation strategy. Beta-lactam residual concentrations were sampled at 24 and 48 h after inclusion. The appropriate concentration was defined as a trough of at least 4 times the minimal inhibitory concentration (clinical breakpoint of EUCAST). The primary outcome was an adequate plasma concentration of the beta-lactam during the first 2 days.

**Results:** Among the 233 patients included in the 11 centers participating to this ancillary study, a beta-lactam trough concentration was evaluated in 112 subjects, 53 in the early and 59 in the delayed groups. Ninety patients (80.4%) had an adequate beta-lactam dosage. RRT initiation strategy had no impact on beta-lactam concentration (p = 0.78). Among the 83 septic shock patients (74% of the sampled patients), 73 (88%) had a correct antibiotic concentration. In contrast, only 10 of the 16 patients without definite sepsis (62.5%) had a correct dosage. Factors associated with an adequate beta-lactam trough concentration in univariate analysis were admission for a septic shock (p = 0.002), a higher plasma creatinine level (p = 0.024), a higher mean arterial pressure (p = 0.018) and a lower serum bicarbonate level (p = 0.045) at randomization. A higher SOFA score was associated with an adequate beta-lactam concentration near to statistical significance (p = 0.053). Multivariate analysis will be presented.

**Conclusion:** In the context of severe AKI, beta-lactam concentration reached a sufficient level in 88% of septic shock patients. Interestingly, RRT initiation strategy was not associated with beta-lactam trough concentration. Early RRT did not affect trough concentration of beta-lactam. We may hypothesize that physicians were highly vigilant and adapted antibiotic administration adequately in these patients.

### CO-45 Predictors of insufficient Amikacin peak concentration in critically ill patients on extracorporeal membrane oxygenation

#### Touchard Cyril^1^, Aubry Alexandra^2^, Bréchot Nicolas^1^, Lebreton Guillaume^2^, Besset Sebastien^2^, Franchineau Guillaume^2^, Hekimian Guillaume^2^, Nieszkowska Ania^2^, Leprince Pascal^2^, Luyt Charles-Edouard^2^, Combes Alain^2^, Schmidt^2^, Matthieu^2^

##### ^1^Clinique du Parc, Aix-en-Provence, France; ^2^Hôpital Pitié-Salpêtrière, Paris, France; ^3^Clinique Saint George, Paris, France

###### **Correspondence:** Touchard Cyril - cyriltouchard@hotmail.fr

*Annals of Intensive Care* 2018, **8(Suppl 1):**CO-45

**Introduction:** Amikacin infusion requires to target a peak serum concentration (C_max_) 8–10 times the minimal inhibitory concentration, corresponding to a C_max_ at 60–80 mg L^−1^ for the least susceptible bacteria. Recent study reported that 33% of critically ill patients do not attain this target with a 25 mg kg dose (1). Membrane sequestration, alteration of the volume of distribution and lack of data in this population make drugs pharmacokinetics (PK) on ECMO challenging. Our study aimed to assess the prevalence of insufficient Amikacin C_max_ in critically ill patients on ECMO and to identify relative risk factors.

**Patients and methods:** Prospective, observational, monocentric study of adult patients on venoarterial (VA) or venovenous (VV) ECMO receiving a loading dose of Amikacin for suspected Gram-negative infections. Intravenous Amikacin was administered with a loading dose of 25 mg kg of total body weight and C_max_ was measured 30 min after the end of the infusion. Independent predicators of C_max_ < 60 mg L^−1^ after the first Amikacin infusion were identified by mixed model multivariate analysis.

**Results:** From January 2015 to February 2016, 106 patients (median SAPS 2 (interquartile range) 68(47–81); age 55(45–62) years) under VA-ECMO (68%) or VV-ECMO (32%) were included. At inclusion, the SOFA score was 15 (12–18) and 54 (51%) patients were on renal replacement therapy. Overall ICU mortality was 54%. C_max_ was < 60 mg L^−1^ in 41 (39%) of the patients. Independent risk factors of amikacin under-dosing were body mass index (BMI) < 22 kg m^−2^ (Odds Ratio (OR) 6.38, 95% confidence interval 95%CI 1.8–22.8, p = 0.043) and a positive 24 h fluid balance (OR per 500 mL increment: 1.28, 95%CI 1.05–1.65, p = 0.041) (Fig. 1). Our results were comparable to those observed in patients treated with Amikacin without ECMO (1).

**Fig. 1:**
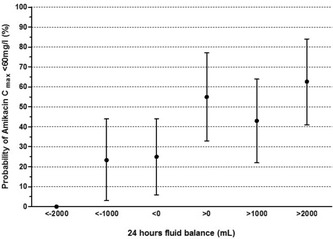
24-h fluid balance and probability of Amikacin C_max_ < 60 mg L^−1^ in ECMO-treated patients. Error bars indicate 95% confidence interval

**Conclusion:** This large prospective study suggests that the prevalence and associated risk factors of Amikacin under-dosing are similar in critically-ill patients with or without ECMO. The use of a 30 mg kg dose in low BMI patients and in those with a positive 24-h fluid balance on ECMO is strongly encouraged to obtain adequate therapeutic targets and prevent therapeutic failure.

**Reference**

1. de Montmollin E, Bouadma L, Gault N, Chemam S, et al. Predictors of insufficient amikacin peak concentration in critically ill patients receiving a 25 mg kg^−1^ total body weight regimen. Intensive Care Med. 2014.

### CO-46 BLInD: Beta-Lactams Induced Delirium? A pilot feasibility study

#### Van Grunderbeeck Nicolas^1^, Klapka-Petit Elisa^1^, Alexandrzak Perrine^1^, Temime Johanna^1^, Pouly Olivier^1^, Bignon Anne^2^, Boulo Marie^2^, Mallat Jihad^1^, Hennart Benjamin^2^, Thevenin Didier^1^

##### ^1^CHU de Lens, France; ^2^CHRU Lille, Lille, France

###### **Correspondence:** Van Grunderbeeck Nicolas - nicovgdb9@orange.fr

*Annals of Intensive Care* 2018, **8(Suppl 1):**CO-46

**Introduction:** Delirium is frequent in the ICU and has impact on morbidity and mortality. Broad-spectrum beta-lactams (BL) are daily used and may have neurological side effects, notably in cases of overdosing. We aimed to assess impact of BL overdosing on confusion through a validated method, the CAM ICU (Confusion Assessment Method for the ICU) as a primary endpoint, and to look for pharmacokinetic factors associated with respect of the BL therapeutic index as a secondary objective.

**Patients and methods:** Prospective observational study–pilot study in two centers. Patients treated with broad spectrum BL for more than 48 h to treat sepsis or septic shock in the ICU could be included. Delirium was assessed through CAM-ICU, and determined group of inclusion: delirium patients or control group. Exclusion citeria were RASS inferior or equal to: 3, imipenem, and other obvious causes of neurologic failure (stroke, poisoning, previous neurological disorders). BL concentrations were measured at peak and trough for intermittent and extended perfusion, and at plateau for continuous perfusion, by high performance chromatography coupled with mass spectrometry. Data were expressed as median [25–75%, interquartile range]. Statistical were performed by Mann–Whitney *U* test and Pearson test. Analysis referred to delirium factors and betalactams’ concentrations, and PK factors (obesity, renal failure, hypoalbuminemia) and overdosing.

**Results:** Fifty patients were included (29 with delirium, 21 controls), at day 4 for controls and day 5 for patients with confusion. Delirium patients were more severely ill SOFA 5 [3; 9] versus 3[1; 5] (p = 0.05); with higher RASS 1[0; 2] versus 0[0; 0] (p = 0.05). They presented with 41% BL overdosing versus 19% in controls (p = 0.09); with 41% of BL in therapeutic index: 71% in controls (p = 0.04). Obesity and renal failure were not associated with BL overdosing but there was a trend with hypoalbuminemia (p = 0.09).

**Discussion:** Trend in association of BL overdosing with delirium corresponds to previous studies, and would need a larger scale study to be confirmed. Severity differences in groups would need changes in inclusion criteria to obtain homogeneous groups. A possible association of BL underdosing with poor evolution of infection and organ failures would need more precise evaluation. Hypoalbuminemia could have an impact on BL overdosing.

**Conclusion:** Delirium was not associated with BL overdosing but with therapeutic index. A high variability of BL concentrations warrants therapeutic drug monitoring. A larger scale study should include changes in design.

### CO-47 Feasibility and safety of low-flow extracorporeal CO_2_ removal with a renal replacement platform to enhance lung protective ventilation in patients with mild to moderate ARDS

#### Schmidt Matthieu^1^, Jaber Samir^1^, Constantin Jean-Michel^1^, Capellier Gilles^1^, Zogheib Elie^1^, Combes Alain^1^

##### ^1^Hopital Pitié Salpetrière, Paris, France

###### **Correspondence:** Schmidt Matthieu - matthieuschmidt@yahoo.fr

*Annals of Intensive Care* 2018, **8(Suppl 1):**CO-47

**Introduction:** Extracorporeal carbon dioxide removal (ECCO2R) might allow ultraprotective mechanical ventilation with lower tidal volume (VT) (< 6 mL kg ideal body weight), plateau pressure (Pplat) (< 30 cm H_2_O), driving pressure, and respiratory rate (RR) to reduce ventilator induced lung injury (VILI). The aim of this study was to assess the feasibility and safety of ECCO2R with a renal replacement platform (RRT) to permit ultra-protective ventilation in patients with mild to moderate acute respiratory distress syndrome (ARDS).

**Patients and methods:** Twenty patients with mild (n = 8) or moderate ARDS were included. VT was gradually reduced from 6 to 5, 4.5 and 4 mL kg^−1^ and PEEP adjusted to reach 23 > Pplat > 25 cm H_2_O. Stand-alone ECCO2R (no hemofilter associated on the RRT platform) was initiated when arterial PaCO2 increased by more than 20%. Ventilation parameters (VT, RR, PEEP), respiratory compliance, driving pressure, arterial blood gases, and ECCO2R system operational characteristics (blood flow, sweep gas flow, and CO_2_ removal rate) were collected during a minimum of 24 h of ultra-protective ventilation. Complications, mortality at day 28, need for adjuvant therapies and data on weaning from both mechanical ventilation and ECCO2R were also collected.

**Results:** While VT was reduced from 6 to 4 mL kg^−1^ and Pplat kept below 25 cm H_2_O, PEEP was significantly increased from 13.4 ± 3.6 at baseline to 15.0 ± 3.4 cm H_2_O at VT = 4 mL kg^−1^. As a result, the driving pressure was significantly reduced to 7.9 ± 3.2 cm H_2_O at VT = 4 mL kg^−1^ (p < 0.05) (Fig. 1). No significant differences in RR, PaO2 FiO2 ratio, respiratory system compliance were observed after Vt reduction. Mean extracorporeal blood, sweep gas flow and CO_2_ removal were 421 ± 40 mL min^−1^, 10 ± 0.3 L min^−1^ and 51 mL min^−1^, respectively. Mean treatment duration was 31 ± 22 h. Main side effects related to ECCO2R were membrane clotting which occurred in 7 patients after 19 ± 9 h.

**Fig. 1:**
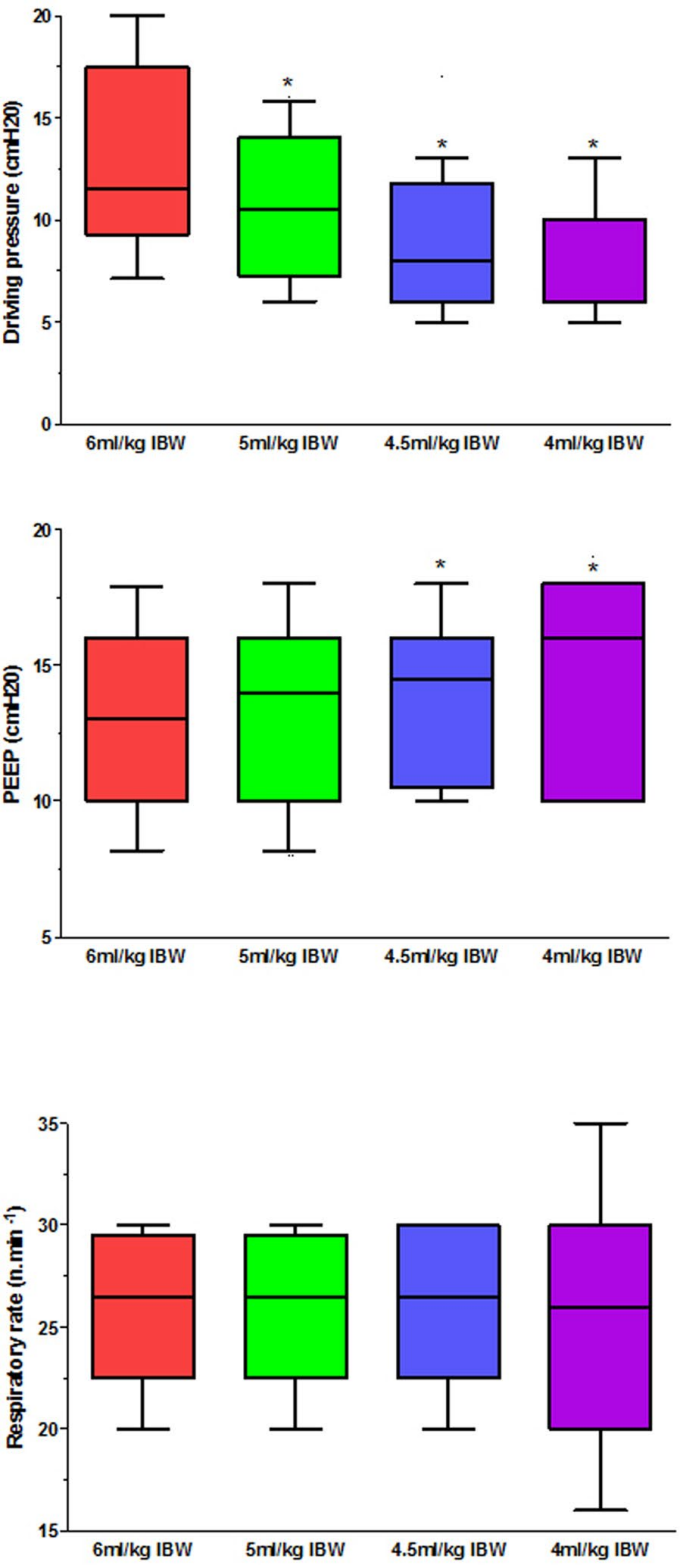
Low-flow extracorporeal CO2 removal

**Conclusion:** A low-flow ECCO2R device driven by a RRT platform efficiently removed CO2 while allowing ultra-protective mechanical ventilation settings in patients with mild to moderate ARDS (ClinicalTrials.gov identifier: NCT02606240).

### CO-48 Afterload burden on the right ventricle is enhanced when ARDS is associated with hypercapnic acidosis

#### Morimont Philippe^1^, Habran Simon^1^, Desaive Thomas^1^, Janssen Nathalie^1^, Amand Theophile^1^, Blaffart Francine^1^, Dauby Pierre^1^, Kolh Philippe^1^, Defraigne Jean-Olivier^1^, Lambermont Bernard^1^

##### ^1^CHU de Liège, Liège, Belgium

###### **Correspondence:** Morimont Philippe - ph.morimont@chu.ulg.ac.be

*Annals of Intensive Care* 2018, **8(Suppl 1):**CO-48

**Introduction:** Protective lung ventilation (PLV) is recommended in patients with acute respiratory distress syndrome (ARDS) to minimize additional injuries to the lung. However, increased right ventricular (RV) afterload resulting from ARDS could be enhanced by hypercapnic acidosis resulting from ventilation at lower tidal volume. Relative contribution of these factors (ARDS and PLV) in RV afterload is not clearly established. The aim of this study was to compare RV afterload in ARDS combined with PLV to RV afterload in PLV alone.

**Patients and methods:** This study was performed in an experimental model of severe hypercapnic acidosis performed in 2 series of 6 pigs. In both groups, respiratory tidal volume was decreased by 60%. In the first group (ARDS group), an ARDS (obtained by repeated bronchoalveolar lavage) was performed before reducing ventilation, while in the second group (control group), hypercapnic acidosis was resulting from low tidal volume ventilation alone.

**Results:** In both groups, systolic pulmonary artery pressure (PAPs) significantly increased during PLV. This increase was significantly higher in ARDS group than in control group (Fig. 1). Severe hypercapnic acidosis occurred in both groups: PaCO2 increased from 41.7 ± 3.6 to 78.6 ± 8.1 (p < 0.01) and arterial pH decreased from 7.44 ± 0.05 to 7.13 ± 0.04 (p < 0.01) in ARDS group while PaCO2 increased from 37.7 ± 9.4 to 93.6 ± 6.4 (p < 0.01) and arterial pH decreased from 7.48 ± 0.05 to 7.11 ± 0.04 (p < 0.01) in control group. PaO2 significantly decreased in ARDS group (178 ± 42 to 54 ± 12.3 mmHg, p < 0.01) but did not significantly changed in control group.

**Fig. 1:**
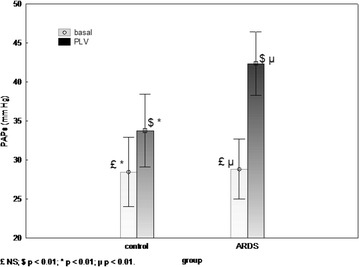
Systolic pulmonary artery pressure (PAPs) during PLV. ARDS and control group

**Conclusion:** Isolated hypercapnic acidosis resulting from PLV was clearly responsible for increased RV afterload and this effect was significantly enhanced in ARDS. Pulmonary vasoconstriction resulting from hypercapnic acidosis is strongly enhanced by factors like hypoxia, endothelial injuries or inflammatory mediators in ARDS. Extracorporeal CO_2_ removal could be the solution to limit afterload burden on the right ventricle when PLV is achieved during ARDS.

### CO-49 Effect of prone positioning on transpulmonary driving-pressure in ARDS patients: a pilot study

#### Persichini Romain^1^, Jozwiak Mathieu^2^, Teboul Jean Louis^2^, Richard Christian^2^, Monnet Xavier^2^

##### ^1^CHU de La Réunion, Saint Denis, France; ^2^Le Kremlin-Bicêtre, Saint Denis, France

###### **Correspondence:** Persichini Romain - romain.persichini@hotmail.fr

*Annals of Intensive Care* 2018, **8(Suppl 1):**CO-49

**Introduction:** Prone positioning has been shown to improve mortality in acute respiratory distress syndrome (ARDS) patients. The respiratory system driving-pressure (DPRS) and the transpulmonary driving-pressure (DPL), measured with esophageal manometry, have been shown to be strongly correlated with mortality. The aim of this study was to investigate the evolution of the DPL during prone positioning and its relationship with evolution of oxygenation in ARDS patients.

**Patients and methods:** Ten patients with ARDS equipped with esophageal manometry were enrolled. DPRS, DPL and chest wall driving-pressure (DPCW) were measured before and 1 h after prone positioning. Respiratory system, pulmonary and chest wall elastance (ERS, EL, ECW) were calculated at the same time. Finally, we studied the correlation between these respiratory variables and oxygenation indicators. Patients were classified as responders to prone positioning if the change in the ratio of arterial oxygen partial pressure oxygen inspired fraction (Delta.PaO2/FiO2) induced by the manoeuvre was larger than the median value observed in the group.

**Results:** In the whole population, median value of Delta.PaO2/FiO2 was 53.5 mmHg, and 5 patients were classified as responders and 5 as non-responders. In responders, DPL significantly decreased from 8.8 ± 4.1 cm H_2_O to 5.9 ± 5.4 cm H_2_O (p = 0.02) and EL decreased from 21.9 ± 5.1 cm H_2_O L to 14.9 ± 6.5 cm H_2_O L (p = 0.02) after prone positioning. Other respiratory variables did not change. In non-responders, respiratory variables did not change. Between responders and non-responders, there was no significant difference between baseline respiratory variables. After prone positioning, Delta.PaO2/FiO2 was not related to baseline respiratory parameters. On the contrary Delta.PaO2/FiO2 induced by prone positioning was strongly correlated with changes in DPL (r = − 0.70, p = 0.02) and changes in EL (r = − 0.69, p = 0.03). We did not find any correlation between Delta.PaO2/FiO2 and changes in DPCW or changes in ECW. The correlation between Delta.PaO2/FiO2 and changes in DPRS (r = − 0.56, p = 0.09) and changes in ERS (r = − 0.55, p = 0.10) did not reach significance.

**Conclusion:** In patients who respond to prone positioning by the highest improvement in oxygenation, DPL significantly decrease after prone positioning. The changes in DPL and the changes in EL play a major role in the improvement in oxygenation induced by prone positioning whereas the changes in DPCW and ECW do not.

### CO-50 A prospective international observational prevalence study on prone positioning ARDS patients: the APRONET (Ards PROne position NETwork) study

#### Guerin Claude^1^, Beuret Pascal^2^, Constantin Jean-Michel^3^, Bellani Giacomo^4^, Baboi Loredana^5^, Mercat Alain^6^, Chretien Jean Marie^6^, François Guy^8^, Ayzac Louis^9^

##### ^1^Hôpital de la Croix Rousse, Lyon, France; ^2^CH Roanne, France; ^3^CHU Clermont-Ferrand, France; ^4^Monza, Italie; ^5^Hôpital de la Croix Rousse, Lyon, France; ^6^CHU Angers, France; ^8^Bruxelles, Belgique; ^9^CHU de Lyon, France

###### **Correspondence:** Guerin Claude - claude.guerin@chu-lyon.fr

*Annals of Intensive Care* 2018, **8(Suppl 1):**CO-50

**Introduction:** Whereas prone positioning (PP) has been shown to improve patient survival in moderate to severe ARDS patients, its rate of use was 7.4% in Lung Safe study. However, Lung Safe study was not specifically focused on PP. Therefore, present study aimed to determine prevalence of use of PP in ARDS patients (primary end-point), physiologic effects of and reasons for not using PP (secondary end-points).

**Patients and methods:** The APRONET study was a prospective international one-day prevalence study performed 4 times in April, July, October 2016 and January 2017. At each study day, investigators had to screen every patient staying in ICU from 0 to 24 h and to fill electronic CRF. For patients with ARDS (defined from the Berlin definition criteria) at each study day oxygenation and ventilator settings were recorded. For those receiving PP these variables were recorded before and at the end of PP session. The reasons for not proning were also collected. Values are presented as median (1st–3rd quartiles). Prevalence rates of PP were compared by using Chi square for trend and groups were compared with nonparametric tests.

**Results:** In the study period 6723 patients were screened in 141 ICUs from 21 countries, of who 735 with ARDS were analyzed. Over the four study days, one-hundred and two ARDS patients received at least one proning session (13.2%). The prevalence of prone positioning in ARDS patients was not significantly different between study days: 13.8% (33 240), 12.6% (18 143), 15.3% (24 157) and 13.8% (27 195) (p = 0.83, Chi square for trend). Over the four study days merged, the rate of PP use was 5.9% (11 187), 10.3% (41 399), 32.9% (49 149) in mild, moderate and severe ARDS, respectively (p = 0.0001). The duration of the first PP session was 18 [16–23] hours. Between supine before PP and end PP, PaO2 FIO2 ratio significantly increased from 101 [76–136] to 171 [118–220] mmHg (p = 0.0001), driving pressure significantly decreased from 14 [11–17] to 13 [10–16] cm H_2_O (p = 0.001). The main reason for not proning was not severe enough hypoxemia (472 734 reasons for not proning 633 ARDS patients, 64.3%).

**Conclusion:** Present study found a higher rate of PP use in severe ARDS patients than in the Lung Safe study, which could reflect change in practice or ICU selection bias.

### CO-51 Treatments and outcomes of severe hypoxemic patients in French speaking ICUs: a subgroup analysis from the SPECTRUM study

#### Hraiech Sami^1^, Grimaldi David^2^, Boissier Florence^3^, Barbar Saber^4^, Brouard Florence^5^, Da Silva Daniel^5^, Ehrmann Stéphane^7^, Hamzaoui Olfa^7^, Kimoune Antoine^7^, Lacherade Jean-Claude^9^, Lascarrou Jean-Baptiste^10^, Michel^11^, Philippe, Piton Gaël^12^, Youssoufa Atika^13^, Muller Grégoire^13^, Aissaoui Nadia^14^, Cerc Cerc^15^

##### ^1^Hôpital Nord, Marseille, France; ^2^Bruxelles, Belgique; ^3^CHU de Poitiers, France; ^4^CHU de Nîmes, France; ^5^CHU de Perigueux, France; ^6^CHU de Tours, France; ^7^CHU Paris-Sud, Paris, France; ^9^CHU de La Roche-Sur-Yon, France; ^10^CHU de Nantes, France; ^11^CHU de Pontoise, France; ^12^CHU de Besançon, France; ^13^CHU de Marseille, France; ^14^Hôpital Européen Georges Pompidou, Paris, France; ^15^(on Behalf Of The Srlf Trial Group), France

###### **Correspondence:** Hraiech Sami - sami.hraiech@ap-hm.fr

*Annals of Intensive Care* 2018, **8(Suppl 1):**CO-51

**Introduction:** Although acute respiratory distress syndrome (ARDS) has been largely focused on, few data are available concerning hypoxemia independently of its cause in intensive care unit (ICU) patients. A recent prevalence-point-day (PPD) evaluated the patterns and outcomes of hypoxemia in French speaking ICUs. Here, we describe the main etiologies, management and outcomes of the patients of this cohort presenting with severe hypoxemia.

**Patients and methods:** A PPD was conducted among 117 French speaking ICUs during spring 2016. Hypoxemia was defined by a PaO2 FiO2 ratio below 300. We analyzed the data from patients with severe hypoxemia (i.e. with a PaO2 FiO2 ratio < 100) and compared their characteristics (causes of hypoxemia, ventilatory and non-ventilatory management) and outcomes to the patients with mild or moderate hypoxemia.

**Results:** Among the 859 hypoxemic patients the day of the study, 74 (9%) had severe hypoxemia. The main cause of hypoxemia was pneumonia and this diagnosis was more frequent than in mild and moderate hypoxemia. Whereas bilateral radiologic infiltrates were present in 56 (84.9%) patients, ARDS was diagnosed by physicians in only 30 (40.5%) of them. Invasive mechanical ventilation (MV) was used in 55 (74.3%) patients. High flow oxygen was administered in 11 (14.9%) of them and 8 (10.8%) were under non-invasive ventilation (NIV) the day of the study. Median Vt was 6.1 (4.8–6.6) ml kg of IBW. Positive end-expiratory pressure (PEEP) was higher than in mild and moderate hypoxemic patients (10 (8–12) vs. 5 (5–8) and 7 (5–10) cm H_2_O respectively, p < 0.001). Median plateau pressure was 25.5 (23–29.5) and was higher than in mild and moderate groups. Median driving pressure was 13 (10–19) cm H_2_O with no difference when compared to other groups. Neuromuscular blocking agents were administered in 28 (51.9%) patients, inhaled nitric oxide (iNO) in 7 (13%) patients and only 6 patients (11.1%) were on prone positioning. Fourteen (18.9%) patients were under extracorporeal membrane oxygenation (ECMO). ICU mortality was higher in severe hypoxemic patients as compared to mild and moderate (50.7 vs. 21.3 and 28.5% respectively, p < 0.001). ICU length of stay in ICU survivors was not statistically different between groups.

**Conclusion:** Severe hypoxemia, independently from ARDS, worsens the prognosis of ICU patients. Even though ARDS might be underdiagnosed, a protective ventilation was respected in severe hypoxemic patients.

### CO-57 Determinants of ICU-acquired infections in septic shock in the current era

#### Llitjos Jean-François^1^, Gassama Aïcha^1^, Jamme Matthieu^1^, Charpentier Julien^1^, Cariou Alain^1^, Chiche Jean-Daniel^1^, Mira Jean-Paul^1^, Pene Frederic^1^

##### ^1^Hôpital Cochin, Paris, France

###### **Correspondence:** Llitjos Jean-François - jllitjos@gmail.com

*Annals of Intensive Care* 2018, **8(Suppl 1):**CO-57

**Introduction:** Major changes in septic shock management raise the questions of the relevance of the classical risk factors of nosocomial infections in the current era and the link with the primary infectious insult. We herein investigated the risk factors and the outcomes of ICU-acquired infections in a recent cohort of septic shock patients.

**Patients and methods:** This was a 9-year (2008–2016) monocenter retrospective study. All adult patients diagnosed for septic shock within the first 48 h were included. Septic shock was defined as a microbiologically proven or clinically suspected infection, associated with acute circulatory failure requiring vasopressors. Patients who survived the first three days were eligible for assessment of the risk of the first ICU-acquired infections. The diagnosis of nosocomial infections were based on current international guidelines. Patients were classified according to the development of pulmonary or non-pulmonary ICU-acquired infections. The determinants of ICU-acquired infections were addressed in a multivariate logistic regression analysis.

**Results:** 938 patients were admitted for septic shock. 788 patients remained alive in the ICU after the first three days and could then be evaluated for the risk of ICU-acquired infections. Hence, 554 patients remained free of secondary infections, 138 patients first developed an episode of nosocomial pneumonia and 96 patients first developed an episode of non-pulmonary infection. The mortality rates of patients with ICU-acquired pneumonia, non-pulmonary ICU-acquired infections and without secondary infections were 49, 49 and 14%, respectively (p = 0.0001). In multivariate analysis, the development of ICU-acquired pneumonia was independently associated with male gender (OR 2.23, CI 95% [1.28–3.48], p = 0.003), renal replacement therapy (OR 2.15, CI 95% [1.35–3.42], p = 0.001), platelet transfusion (OR 2.38, CI 95% [1.4–4.04], p = 0.001) and a primary pulmonary infection (OR 8.06, CI 95% [2.69–24.12], p < 0.001). The development of non-pulmonary infections was independently associated with renal replacement therapy (OR 4.6, CI 95% [2.7–7.85], p < 0.001), fresh frozen plasma transfusion (OR 2.49, CI 95% [1.42–4.37], p = 0.001), healthcare-associated septic shock (OR 1.8, CI 95% [1.1–2.91], p = 0.01).

**Conclusion:** ICU-acquired pneumonia occurs preferentially in patients with septic shock of pulmonary origin. In addition, we identified the transfusion of blood products as a risk factor for pulmonary and non-pulmonary nosocomial infections.

### CO-58 Albumin infusion had protective endothelial effects in septic shock patients

#### Hariri Geoffroy^1^, Joffre Jérémie^1^, Mialhe Arnaud-Felix^1^, Bigé Naïke^1^, Dumas Guillaume^1^, Deryckere Stéphanie^1,^ Baudel Jean-Luc^1^, Maury Eric^1^, Guidet Bertrand^1^, Aït-Oufella Hafid^1^

##### ^1^Hôpital Saint-Antoine, Paris, France

###### **Correspondence:** Hariri Geoffroy -geoffroyhariri@hotmail.com

*Annals of Intensive Care* 2018, **8(Suppl 1):**CO-58

**Introduction:** Human serum albumin is used for the restoration of blood volume, emergency treatment of septic shock patients. Several experimental studies suggested that albumin could have additional protective effects on the vascular wall and more specifically on endothelial functions. However, the in vivo effect of albumin in human endothelium remains unknown. The aim of this study is to assess the effect of albumin or saline infusion on skin endothelial function in septic shock patients requiring volume expansion.

**Patients and methods:** We performed a prospective randomized monocentric study in an 18-bed medical intensive care unit. All patients with septic shock who required fluid administration were included between H6 and H24 after vasopressor starting. Patients were randomized to receive either 500 ml of saline solution 0.9% or 100 ml of albumin 20%. Norepinephrine dose was not modified 1 h before and during the procedure. Endothelium-dependant vasodilatation in the skin circulation was assessed by iontophoresis of acetylcholine before and after fluid administration. The improvement of skin blood flow in response to acetylcholine after fluid administration was compared between groups. For each patient, age, sex, SAPS II, site of infection, global hemodynamic parameters and clinical microcirculatory parameters were recorded. Results are expressed as mean ± SD. Qualitative data were compared using Chi-2 or Fisher’s exact test while quantitative data comparisons used Student t Test or Mann–Whitney as appropriate.

**Results:** Twenty-two patients were included (12 women, age: 79 ± 17, SAPS II: 42 ± 12). Twelve patients received saline and 10 received albumin. Apart from age, no statistical difference was found between groups regarding demographic characteristics and baseline hemodynamic parameters. Norepinephrine dose and mean volume of infused fluid before inclusion was not different between groups (Table 1). Before fluid replacement, endothelial response to acetylcholine iontophoresis was not different between groups (AUC 3514 vs 3378; p = 0.16). Volume expansion induced a slight increase of systolic arterial pressure, significantly higher in the albumin group (7 vs 2%; p = 0.02) with no difference regarding cardiac output variations between groups. Next, we compared the variations of endothelium response to iontophoresis before and after fluid infusion. The improvement of endothelial response after acetylcholine challenge was significantly higher in the albumin group (196 vs 44%, p = 0.01).

**Table 1:** Characteristics of patients

Characteristics of patients	NacL (n = 12)	Albumine (n = 10)	p
Age (year)	84 ± 13	73 ± 17	0.05
Male gender, (%)	44	55	NS
Comorbidities (%)			
Diabetes	17	10	NS
HTA	67	30	NS
Vasular disease	33	20	NS
IRC	0	10	NS
Cirrhosis	0	0	NS
Primary site of infection (%)			
Lung	25	40	NS
Abdomen	25	20	NS
Urinary tract	33	20	NS
Other	17	20	NS
SAPSII	51 ± 10	38 ± 12	NS
Norepinephrine (μg/kg/min)	0.8 ± 0.55	0.25 ± 0.71	NS
Fluid infused (ml)	2000 ± 821	2000 ± 773	NS

**Conclusion:** In the early stage of septic shock resuscitation, we showed that albumin infusion had protective endothelial effects. This result has to be confirmed in a larger cohort.

### CO-59 Early identification of sepsis-associated encephalopathy with EEG is not associated with short-term cognitive dysfunction

#### Maenhout Christelle^1^, Ferlini Lorenzo^1^, Crippa Ilaria Alice^1^, Taccone Fabio^1^, Créteur Jacques^1^, Peigneux Philippe^1^, Gaspard Nicolas^1^

##### ^1^ULB - Hôpital Erasme, Bruxelles, Belgium

###### **Correspondence:** Maenhout Christelle - christelle.maenhout@gmail.com

*Annals of Intensive Care* 2018, **8(Suppl 1):**CO-59

**Introduction:** Encephalopathy occurs in 75% of patients with sepsis and is associated with short-term mortality and long-term cognitive disability among survivors. It is currently unclear what is the gold standard to detect SAE, how it can be prevented, and how evolution to long-term disability can be prevented. The aims of this study were to determine which clinical scales are altered after sepsis and if acute encephalopathy is associated with short-term cognitive impairment.

**Patients and methods:** We prospectively included adult patients with sepsis between January 2016 and September 2017. Exclusion criteria were encephalopathy from another etiology, psychiatric disorder, recent sepsis or cardiac surgery. We assessed consciousness twice daily (with the Confusion Assessment Method for the ICU [CAM-ICU], Glasgow Coma Scale [GCS], Full Outline of UnResponsiveness [FOUR], Coma Recovery Scale-Revised [CRS-R], and Reaction Level Scale 85 [RLS85]). All patients received continuous EEG monitoring for up to 7 days after admission. We assessed encephalopathy using a modified Synek scale. After discharge from ICU, we assessed cognitive functions (with the Montreal Cognitive Assessment [MOCA], the Frontal Assessment Battery [FAB], and the Language Screening Test [LAST]). A healthy control group of 18 adults was used for comparison.

**Results:** We enrolled 38 patients, all with EEG demonstrated encephalopathy. Clinical tools identified encephalopathy in 57 (42% + positive CAM-ICU), 147 (72% + GCS < 15) + 113 (55% + FOUR < 16) + 140 (69% + CRS-R < ) and 139 assessments (68% + RLS85 > 1). We found significant correlations between clinical and EEG assessment (GCS-Eye [R2 = .35], GCS-Verbal [R2 = .43], GCS-Motor [R2 = .375], FOUR-Eye [R2 = .363], FOUR-Motor [R2 = .344], FOUR-Brainstem [R2 = .183], FOUR-Respiration [R2 = .38], CRS-R [R2 = .432], and RLS85 [R2 = .418] + all p < .001). We found no correlation between cognitive scores at hospital discharge and the severity of EEG-defined encephalopathy during the 7 days of ICU or during the first 48 h after admission. However, sepsis survivors’ scores were lower than controls’ (p < .001) (Table 1).

**Table 1:** Cognitive scores for Septic group vs Control group and for SAE and non-SAE groups

	Septic group (*n* = 26)	Control group (*n* = 18)	Septic versus CT group
	M ± *SD*	M ± *SD*	*p*
MoCA	23.54 ± *4.92*	29.11 ± *1.18*	.000
BREF	14.62 ± *3.38*	17.83 ± .*383*	.000
LAST	14.73 ± *.72*	15 ± *.0*	.007

	SAE group (*n* = 14)	Non-SAE group (*n* = 8)	SAE *vs* Non-SAE
	M ± *SD*	M ± *SD*	*p*
MoCA	22.21 ± *5.6*	24.88 ± *4.7*	.271
BREF	13.36 ± *4.01*	15.88 ± *1.64*	.053
LAST	14.66 ± *0.62*	14.75 ± *0.7*	.293

**Conclusion:** In this study, EEG was more sensitive than clinical tools to detect SAE but clinical scales correlated with the EEG grade. Encephalopathy was not associated with short-term cognitive function. Further study and a larger cohort are needed to determine which early EEG features can identify patients who will develop short-term and long-term cognitive dysfunction.

### CO-60 Cerebral autoregulation during septic shock

#### Crippa Ilaria Alice^1^, Vincent Jean-Louis^1^, Creteur Jacques^1^, Subirà Carles^2^, Taccone Fabio^1^

##### ^1^Université Libre de Bruxelles, Belgium; ^2^Althaia Xarxa Assistencial Universitària, Manresa, Spain

###### **Correspondence:** Crippa Ilaria Alice - ilaria.alice.crippa@gmail.com

*Annals of Intensive Care* 2018, **8(Suppl 1):**CO-60

**Introduction:** Sepsis associated encephalopathy (SAE) is associated with increased morbidity and mortality. Its pathophysiology is incompletely elucidated but a possible impairment of cerebral autoregulation (CAR) could result in brain hypoperfusion and neuronal damage (1, 2). We studied CAR in septic patients to test the hypothesis that its alteration is associated with SAE.

**Patients and methods:** We studied 96 adult patients with sepsis (July 2015–August 2017). Exclusion criteria were—intracranial disease; arrhythmias; extracorporeal membrane oxygenation; and any supra-aortic arteriopathy. Transcranial Doppler (DWL, Germany) was performed, insonating the left middle cerebral artery (LMCA) with a 2 MHz probe. LMCA blood flow velocity (FV) and arterial blood pressure (BP) signals were simultaneously recorded; Pearson´s correlation coefficient between BP and FV (Mxa) was calculated using MATLAB (MathWorks, USA). Impaired CAR was defined as Mxa > 0.3 (3). All data are reported as median [IQR], n (%).

**Results:** Age was 62 [52–71] years + APACHE II score was 21 [15–26] + 70 96 patients were treated with norepinephrine (0.15 [0–0.5] mcg min). Site of infection was mainly abdominal (45%) or pulmonary (28%). ICU length of stay was 7[4–12] days. Seventy-two patients (75%) were alive at ICU discharge. Mxa was 0.27 [0.02–0.62]. CAR was impaired in 46 patients (48%) overall. SAE was diagnosed in 54 patients (56%). CAR was altered in 31 54 (57%) patients with SAE and 15 41 (36%) patients without SAE (p = 0.06). In univariate analysis (Table 1), Mxa and history of arteriopathy were different in patients with and without SAE + the differences persisted in a multivariate analysis (p < 0.05).

**Table 1:** Unvivariate analysis

	SAE (n = 54)	No SAE (n = 41)	p value
Mxa	0.43 [0.19 to 0.63]	0.20 [− 0.14 to 0.52]	0.02
Arteriopathy	16 (30%)	3 (7%)	< 0.01

**Conclusion:** CAR was altered in nearly half of the patients with sepsis. Mxa was higher in patients with than without SAE.

### CO-61 Bedside assessment of resistance to corticotherapy in adults with sepsis

#### Sivanandamoorthy Sivanthiny^1^, Heming Nicholas^1^, Meng Paris^1^, Maxime Virginie^1,^ Chevret Sylvie^2^, Annane Djillali^1^

##### ^1^Hôpital Raymond Poincare, Garches, France; ^2^Hôpital Saint-Louis, Paris, France

###### **Correspondence:** Sivanandamoorthy Sivanthiny - sivanthiny.sivanandamoorthy@aphp.fr

*Annals of Intensive Care* 2018, **8(Suppl 1):**CO-61

**Introduction:** There is growing evidence that corticotherapy improves survival from septic shock. This observational study aimed at evaluating at bedside resistance to corticosteroids in adults with sepsis.

**Patients and methods:** Participants—ICU adults with septic shock or without sepsis admitted to the Raymond Poincaré university hospital. We also evaluated 10 healthy controls. Intervention—resistance to corticosteroids was assessed using a skin test. 10 µl of dermocorticoid cream (class III, Betamethasone) was applied on a 3 cm^2^ surface of the skin. At 24 h, two independent physicians scored the blanching of the skin from 0 to 4—0- no blanching + 1- < 50% of surface + 2- 50 to 75% of surface + 3- 75 to 100% of surface, and 4- blanching beyond application area. Cohen’s kappa was used to measure concordance. A mean score of < 2 indicated corticoresistance and a score of 4 indicating normal sensitivity to corticosteroids. We also performed a 250 µg ACTH test.

**Results:** We enrolled 110 patients, 47 patients with septic shock (22 males, SAPSII 34 [6–114]) and 63 patients without sepsis (28 males, SAPSII 30 [6–100]). Overall, 81 95 (85%) with two measurements patients had concordant evaluation of score by the two physicians + while 14 had a difference of 1-point in scores, resulting in a kappa of 0.78 (95% CI 0.65–0.91). In patients with septic shock, 32 (78%) have corticoresistance, i.e. a mean score < 2, 8 (20%) a score of 2 or 3, and 1 (2%) has normal sensitivity to corticosteroids. In non-septic critically ill, 30 (56%) have corticoresistance, 20 (37%) a mean score of 2–3, and 4 (7%) have normal sensitivity to corticosteroids. Hence, as compared to non-septic patients, patients with septic shock were more likely to have corticoresistance (p = 0.04).

**Discussion:** Topic application of corticosteroids on the skin results in activation of glucocorticoid receptors present within the vessels. Subsequently, activation of lipocortin 1 may inhibit the activity of phospholipase A2, regulator of prostaglandins, leucotrienes and platelet activating factor. Then, the coupling of alpha adrenoreceptors to their agonists is potentiated, increasing vessels smooth muscles sensitivity to catecholamines. The subsequent local vasocontriction is reflected by skin blanching. Thus, the observed lack of skin blanching in septic patients may reflect altered coupling between gluocorticoids and glucocorticoids receptors.

**Conclusion:** Roughly one out of two adults with septic shock may develop a resistance to corticosteroids as assessed by a skin blanching test in response to betamethasone.

### CO-62 Short term antibiotics prevents early VAP in patients treated with mild therapeutic hypothermia after cardiac arrest

#### Daix Thomas^1^, Cariou Alain^2^, Clere-Jehl Raphaël^3^, Dequin Pierre-François^4^, Guitton Christophe^5^, Deye Nicolas^6^, Plantefève Gaëtan^7^, Quenot Jean-Pierre^8^, Desachy Arnaud^9^, Kamel Toufik^10^, Bedon-Carte Sandrine^11^, Diehl Jean-Luc^12^, Nicolas Chudeau^13^, Karam Elias^14^, Renon-Caron Françoise^1^, Vignon Philippe^1^, Le Gouge Amélie^4^, François Bruno^1^

##### ^1^CHU Dupuytren, Limoges, France; ^2^HU Paris-Centre, Paris, France; ^3^Hôpital civil, Strasbourg, France; ^4^CHU Bretonneau, Tours, France; ^5^CHU de Nantes, Nantes, France; ^6^CHU Lariboisière, Paris, France; ^7^CH Victor Dupouy, Argenteuil, France; ^8^CHU de Dijon, France; ^9^CH d’Angoulême, France; ^10^CHR Orléans, Orléans, France; ^11^CH de Périgueux, France; ^12^HU Paris-Ouest, Paris, France; ^13^CHU Le Mans, France; ^14^CHU de Brive La Gaillarde, France

###### **Correspondence:** Daix Thomas - thomas.daix@wanadoo.fr

*Annals of Intensive Care* 2018, **8(Suppl 1):**CO-62

**Introduction:** Mild therapeutic hypothermia, currently recommended in the management of cardiac arrests with shockable rhythm could promote infectious complications and especially ventilator-associated pneumonia (VAP) (Mongardon et al. Crit Care Med 2011). Despite high incidence of VAP and retrospective trials suggesting a benefit of short-term (48 h) antibiotics in this setting (Davies et al. Resuscitation 2013), systematic use of antibiotic prophylaxis is not recommended in patients treated with mild therapeutic hypothermia after cardiac arrest. The primary objective was to demonstrate that systematic short-term antibiotic prophylaxis with amoxicillin-clavulanic acid can reduce incidence of early VAP (< 7 days) in patients treated with mild therapeutic hypothermia after out-of-hospital cardiac arrest. Secondary objectives were its impact on incidence of late VAP and on Day 28 mortality.

**Patients and methods:** Multicenter two parallel-group double-blinded randomized trial. Adult patients hospitalized in ICU, mechanically ventilated after out-of-hospital resuscitated cardiac arrest related to initial shockable rhythm and treated with mild therapeutic hypothermia were eligible. Exclusion criteria were pregnancy, need for extracorporeal life support, ongoing antibiotic therapy or pneumonia, known chronic colonization with multiresistant bacteria, known allergy to beta-lactam antibiotics and moribund patients. Patients received either intravenous injection of amoxicillin-clavulanic acid (1 g 200 mg) or placebo three times a day for 2 days. The primary endpoint was the onset of early VAP. All suspected pulmonary infections were adjudicated by a blinded independent committee.

**Results:** Out of 198 patients included, 196 were finally analyzed, 99 in treatment group and 97 in placebo group (mean age 60.5 ± 14.4 years, sex ratio = 4, SOFA score 8.7 ± 3.1). Characteristics of cardiac arrest were similar in both groups (no flow = 3.5 ± 4.8 min vs 3.8 ± 4.0 min, low-flow = 21.8 ± 13.7 min vs 18.2 ± 10.1 min). 51 early VAP were confirmed, 19 in treatment group vs 32 in placebo group, with an incidence of 19.2 vs 33.0%, respectively (HR = 0.546 + IC 95% = [0.315 + 0.946], p = 0.031) (Fig. 1). The procedure did not affect occurrence of late VAP (> 7 days), respectively 4 vs 5. Day 28 mortality was similar in both arms (41.4 vs 37.5%, p = 0.58) and no adverse event was related to study treatment.

**Fig. 1:**
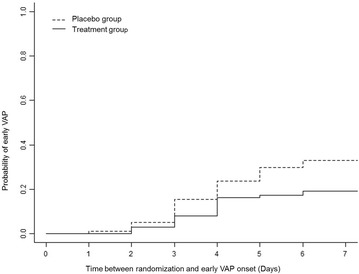
Time between randomization and early VAP onset

**Conclusion:** Short-term antibiotic prophylaxis with amoxicillin-clavulanic acid significantly decreases incidence of early VAP in patients treated with mild therapeutic hypothermia after out-of-hospital cardiac arrest related to shockable rhythm.

### CO-63 Treatments and outcomes of hypoxemia in immunosuppressed patients compare to immunocompetent patients in French speaking ICU: a sub-study from the SPECTRUM study

#### Grimaldi David^1^, Hraiech Sami^2^, Boissier Florence^3^, Lacherade Jean Claude^4^, Muller Gregoire^5^, Michel Philippe^6^, Lascarrou Jean Baptiste^7^, Piton Gaël^8^, Barbar Saber^9^, Youssoufa Atika^8^, Brouard Florence^11^, Ehrmann Stephan^12^, Aissaoui Nadia^13^

##### ^1^Hôpital Erasme, Bruxelles, Belgium; ^2^Hôpital Nord, Marseille, France; ^3^CHU de Poitier, France; ^4^CHU de La Roche-sur-Yon, France; ^5^Hôpital de la Source, Orléans, France; ^6^; ^7^CHU de Nantes, France; ^8^CHU de Besançon, France; ^9^CHU de Nîmes, France; ^10^CH de Périgueux, France; ^11^CHU de Tours, France; ^12^Hôpital Européen Georges Pompidou

###### **Correspondence:** Grimaldi David - david_grimaldi2001@yahoo.fr

*Annals of Intensive Care* 2018, **8(Suppl 1):**CO-63

**Introduction:** Immunosuppressed (IS) patients are prone to develop respiratory failure and to need ventilatory support. Invasive ventilation shared a grim prognosis in the past and non-invasive ventilation had been recommended in these patients, however NIV efficacy has been recently challenged and the advent of high flow oxygen therapy had brought even more complexity in the management of such patients. Using the data from a recent point-prevalence-day of hypoxemia in ICU, we compare the frequency, management and outcomes of hypoxemia in IS and immuncompetent (IC) patients.

**Patients and methods:** The SPECTRUM study was conducted in 117 French-speaking ICUs in 7 countries during spring 2016. IS was retained in case of malignant hemopathy, HIV positivity, immunosuppressive drugs, recent chemotherapy, neutrophil count < 0.5 G L. Hypoxemia was defined as a PaO_2_ FiO_2_ ratio > 300 and separate into severe (> 100), moderate (> 200) and mild (> 300). We focused on the causes of hypoxemia, the ventilatory management and the outcome.

**Results:** Among the 1604 patients included, 187 (12%) were IS out of whom 99 (53%) were hypoxemic, proportion similar to the IC patients. Mean age and IGS-2 of hypoxemic patients were similar in IS and IC patients. Hypoxemia was mild in 46 (46%), moderate in 43 (43%) and severe in 10 (10%) IS patients with a similar distribution compared to hypoxemic IC patients. The causes of hypoxemia were also similar pneumonia being the leading cause. 24 (24%) hypoxemic IS patients fulfilled the Berlin criteria for ARDS in a similar proportion to IC patients. Respiratory support used in hypoxemic IS patients was ambient air in 3, low flow oxygen in 24, high flow in 11, NIV in 5 and invasive ventilation in 56 patients, with a different distribution from the IC patients (more patients on high flow therapy and less invasively ventilated). The day of the study, thoracic CT scan and echocardiography were performed in a similar proportion in IS and IC patients whereas broncho-alveolar lavage was more frequently performed in IS patients (14 vs 6%, p < 0.001). Finally, as expected, ICU mortality was higher in hypoxemic IS patients (38 vs 25%, p < 0.01).

**Conclusion:** Immunosuppression in the ICU seems not to be associated with hypoxemia, severity of hypoxemia or ARDS. Oxygenation management is slightly different from immunocompetent patients with more frequent use of high flow therapy.

### CO-64 Can a flow rate of 3 L/kg/min, compared to 2 L/kg/min, reduce the risk of failure during the initial management of acute viral bronchiolitis with high flow nasal cannulae: a randomized controlled trial (TRAMONTANE 2 study)

#### Milesi Christophe^1^, Pierre Anne Florence^2^, Deho Anne^3^, Pouyau Robin^4^, Liet Jean-Michel^5^, Guillot Camille^6^, Guilbert Anne Sophie^7^, Rambaud Jerome^8^, Millet Anne^9^, Afanetti Mickael^10^, Guichoux Julie^11^, Genuini Mathieu^12^, Mansir Thierry^13^, Bergounioux Jean^14^, Michel Fabrice^15^, Marcoux Marie Odile^16^, Baleine Julien^1^, Durand Sabine^1^, Douillard Aymeric^1^, Cambonie Gilles^1^

##### ^1^CHU de Montpellier, Montpellier, France; ^2^Hopital Kremlin Bicetre, Paris, France; ^3^Hopital Robert Debré, Paris, France; ^4^Hopital de Lyon, Lyon, France; ^5^Hopital de Nantes, Nantes, France; ^6^Hopital Jeanne de FLandre, Lilles, France; ^7^CHU de Strasbourg, France; ^8^HU Est parisien, Paris, France; ^9^CHU de Grenoble, Grenoble, France; ^10^CHU Lenval, Nice, France; ^11^CHU de Bordeaux, France; ^12^HU Necker, Paris, France; ^13^CH de Pau, France; ^14^Hôpital Raymond Poincaré, Paris, France; ^15^Hôpital de la Timone, Marseille, France; ^16^CHU TLSE, Toulouse, France

###### **Correspondence:** Milesi Christophe - c-milesi@chu-montpellier.fr

*Annals of Intensive Care* 2018, **8(Suppl 1):**CO-64

**Introduction:** HFNC is currently proposed as first-line respiratory support in moderate to severe acute viral bronchiolitis (AVB) in infants. However, the flow setting remains empiric, 2 L/kg/min being used by most teams. Considering the failure rate observed with this device, as high as 50% in some studies, we hypothesized that a higher flow rate may improve this issue. The purpose of the present study was to compare the failure rates with two flow regimen-2 L/kg/min versus 3 L/kg/min.

**Patients and methods:** A randomized controlled study was performed in 16 French Pediatric Intensive Care Units (PICUs). Infants younger than 6 months-old with moderate to severe AVB, defined by Wood-modified Clinical Asthma score (mWCAs) > 3, were randomly allocated to HFNC treatment with a flow rate of 2 L/kg/min or 3 L/kg/min for 48 h. The primary endpoint was the percentage of failure, defined as the occurrence of one or more of the following—increase in mWCAs or RR, increase in discomfort (EDIN score), and severe apnea episodes.

**Results:** 287 infants with mean (SD) age and weight of 47 (58) days and 4460 (1130) g were included from November 2016 to March 2017. At baseline, RR was 58 (16) rpm, mWCAs 4.5(1), FiO2 32 (13) %, PCO2 59 (13) mmHg, pH 7.26 (0.1). 142 were included in the 2 L/kg/min group and 145 in the 3 L/kg/min group. No difference was observed between groups for baseline characteristics. Failure rate was not different between groups—38.7 vs 39.3% + p = 0.92. No center effect was observed for failure. Discomfort was more frequent in the 3 L kg min group—7 vs 17% + p = 0.006. The length of stay was shorter in the 2 L kg min group—5.3 (2.8) vs 6.4 (5) days + p = 0.048. Intubation occurred in 4 142 patients in the 2 L/kg/min group vs 10 145 patients in the 3 L kg min group (p = 0.12).

**Conclusion:** HFNC with a flow rate of 3 L/kg/min did not reduce the risk of failure compared to 2 L/kg/min at the initial respiratory management of AVB in young infants.

### CO-65 Comparison of epinephrine and norepinephrine for the treatment of cardiogenic shock following acute myocardial infarction. OPTIMA CC study

#### Levy Bruno^1^, Meziani Ferhat^1^, Leone Marc^2^, Guiot Philippe^3^, Quenot Jean-Pierre^4^, Louis Guillaume^5^, Legras Annick^6^, Duarte Kevin^7^, Vignon Philippe^8^

##### ^1^CHRU de Strasbourg, France; ^2^Hôpital Nord, Marseille, France; ^3^Hôpital Emile Muller, Mulhouse, France; ^4^CHRU de Dijon, France; ^5^CHR Metz, France; ^6^CHRU Bretonneau, Tours, France; ^7^CHU de Nancy, France; ^8^CHU de Limoges, France

###### **Correspondence:** Levy Bruno - blevy@sfr.fr

*Annals of Intensive Care* 2018, **8(Suppl 1):**CO-65

**Introduction:** Despite the frequent use of vasopressors which are administered in 90% of patients in cardiogenic shock (CS), there is only limited evidence from randomized trials comparing vasopressor in CS. Hence, the OPTIMA CC study was designed to compare epinephrine and norepinephrine in cardiogenic shock following myocardial infarction.

**Patients and methods:** Multicenter, double-blind, randomized trial in 8 french ICU. Cardiogenic shock patients due to myocardial infarction treated by PCI were randomized to receive epinephrine or norepinephrine to maintain MAP at 70 mmHg. Dobutamine was introduced at the physician discretion according to a combination of parameters—echocardiographic parameters, cardiac index, lactate clearance, SVO2 and Swan-Ganz derived parameters.

**Results:** 56/57 patients were ventilated (98%). There were no differences in the duration nor in the maximal dose or cumulated dose of epinephrine or norepinephrine. Dobutamine was used in 18/27 (67%) in the epinephrine group and in 20/30 (67%) in the norepinephrine group. There were no differences in the duration, in the maximal or cumulated dose. Arterial pressure evolution was similar. Heart rate increased significantly in epinephrine group and did not change in norepinephrine group. Cardiac index and cardiac power index increased significantly more in the epinephrine group than in the norepinephrine group. Cardiac double product, a surrogate of myocardial oxygen consumption increased in epinephrine group and did not change in norepinephrine group. Epinephrine use was associated with a lactic acidosis from H2 to H24 while arterial pH increased and lactate level decreased in norepinephrine groupEpinephrine was significantly associated with an higher incidence of refractory shock—10/27 (37%) versus 2/30 (7%) p = 0.008). The incidence of arrhythmia was identical (epinephrine—41% versus norepinephrine—33%, p = 0.59). ECMO was used in 3/27 (11%) in the epinephrine group and in 1 30 (3%) in the norepinephrine group (p = 0.34) Mortality was 11/30 (37%) in the norepinephrine group and 14/27 (52%) in the epinephrine group (p = 0.25) Epinephrine use was associated with a trend to an increased risk of death (p = 0.08) and an increased risk of death plus ECMO (p = 0.031) at 7 days. There was a trend for an increased risk of death plus ECMO at J28 (p = 0.064).

**Conclusion:** In patients with cardiogenic shock following myocardial infarction, epinephrine use was associated with a lactic acidosis, an higher incidence of refractory shock and an increased risk of death plus ECMO at J7.

### CO-66 WITHDRAWN

### CO-67 High dose immunoglobulins in toxic shock syndrome in children: a pilot randomized controlled study (IGHN study)

#### Javouhey Etienne^1^, Leteurtre Stéphane^2^, Tissières Pierre^3^, Joram Nicolas^4^, Wroblewski Isabelle^5^, Ginhoux Tiphanie^6^, Dauger Stéphane^7^, Kassai Behrouz^8^

##### ^1^Hôpital Mère Enfant, Bron, France; ^2^Hôpital Jeanne de Flandre, Lille, France; ^3^Hôpital du Kremlin-Bicêtre, Le Kremlin-Bicêtre, France; ^4^CHU Nantes, Nantes, France; ^5^CHU Grenoble, La Tronche, France; ^6^Hospices Civils de Lyon, Bron, France; ^7^Hôpital Robert Debré, Paris, France; ^8^Hospices Civils de Lyon, Bron, France

###### **Correspondence:** Javouhey Etienne - etienne.javouhey@chu-lyon.fr

*Annals of Intensive Care* 2018, **8(Suppl 1):**CO-67

**Introduction:** Superantigen toxins synthesized by S. aureus or by S. pyogenes are responsible for toxic shock syndromes (TSS) which lethality can reach 28%. High dose intravenous immunoglobulins (IVIG), able to neutralize these toxins, are frequently used even tough evidence of its efficacy is not supported by randomized controlled study (RCT). Moreover, IVIG are expensive and possibly harmful. Before conducting a RCT, a pilot study was first designed to assess the feasibility in the context of pediatric critical care.

**Patients and methods:** A double blinded RCT was performed comparing 2 g kg of IVIG to isovolumic 4% albumin perfusion within the first 12 h of TSS in children aged between 1 month to 17 years. A priori criteria to determine the feasibility were defined as a rate of inclusion among eligible patients > 50%, a rate of protocol’s deviations < 30% (treatment delivery, non-respect of blinding, premature stop), and by the practical and financial aspects of the protocol. Secondary objectives were to assess the efficacy of IVIG on organ dysfunction (using Pelod-2 score), on mortality at day 60 and their safety. The study was promoted by the Hospices Civils of Lyon, approved by the CPP Sud-Est and registered at clinical trial (NCT02219165). Inform consent from both parents was required before randomization. This study was funded by CSL-Behring company.

**Results:** During the 30 months study period, 21 patients were included in 9 centers. The inclusion rate was of 71% (5 parent’s refusals, 3 parents were absent at admission). Two patients were wrongly included (pneumococcal shocks), one patient didn’t receive the treatment because he was transferred for ECMO in a non-investigator center, three patients were treated after 12H, and in two patients one bottle of treatment was missing. The blinding was well respected. Missing data on the Pelod2 score and mortality was lower than 10%, and no premature stop was reported. The eCRF completion was judged easy by investigators. The inclusion of children within the first 12 h was judged challenging. The treatment delivery had to be improved, requiring the help of research assistants. Seven serious and one severe adverse events were registered, all patients recovered and no death was reported.

**Conclusion:** This pilot study suggested that a RCT is feasible. It provides crucial information to improve the recruitment, the respect of the protocol and the correct measure of organ failure. However, inclusion of international centers is necessary to attain the sample size required.

### CO-68 Indirect calorimetry as a tool to assess the work of breathing in critically ill children

#### Mortamet Guillaume^1^, Nardi Nicolas^2^, Essouri Sandrine^2^, Jouvet Philippe^2^, Emeriaud Guillaume^2^

##### ^1^CHU Grenoble, Grenoble, France; ^2^CHU Sainte-Justine, Montréal, Canada

###### **Correspondence:** Mortamet Guillaume - mortam@hotmail.fr

*Annals of Intensive Care* 2018, **8(Suppl 1):**CO-68

**Introduction:** Indirect calorimetry is a non invasive tool to measure oxygen consumption (VO_2_) and resting energy expenditure at bedside. The aim of the present study was to assess the validity of the indirect calorimetry-based method for the work of breathing assessment when compared to esophageal pressure (Pes) measurement and Electrical Activity of the Diaphragm (EAdi) during a spontaneous breathing trial in continuous positive airway pressure.

**Patients and methods:** A prospective single center study. All intubated and mechanically ventilated children > 1 months and < 18 years old, hospitalized in the pediatric intensive care unit were eligible. Patients considered as ready to extubate were included. Simultaneous recordings of VO2, Pes and EAdi were performed during 3 steps: before, during and after the spontaneous breathing test in continuous positive airway pressure.

**Results:** Twenty patients, median 5.5 months, were included. Half of the patients were admitted for a respiratory reason. Predicted resting energy expenditure was overestimated as compared to measured resting energy expenditure (51 [47–55] vs 23 [21–29] kcal kg day, p < 0.001). Spontaneous breathing test was associated with an increase in Esophageal Pressure–Time Product from 23 [5–89] to 83 [24–110] cm H_2_O s min. The same trend was observed in respiratory drive, assessed by EAdi which increased from 7.5 [3.2–16.3] to 15.9 [5.2–22.1]. Oxygen consumption obtained by IC was higher during spontaneous breathing test as compared to conventional ventilation (3.8 [3.0–5.2] vs 3.6 [3.1–4.6] ml kg min) but non significantly. Changes in work of breathing as assessed by VO_2_ was poorly correlated with measurements from Pes and EAdi whereas we found a moderate correlation between Pes and EAdi values. Spontaneous breathing test and extubation were successful in 20 (100%) and 18 (90%) patients, respectively.
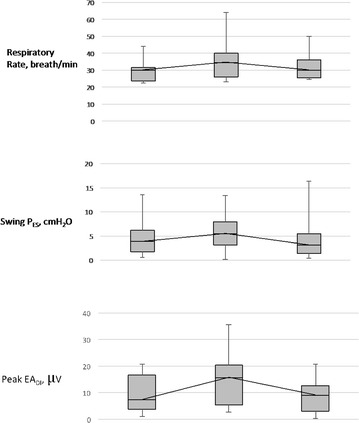


**Conclusion:** During weaning from mechanical ventilation, spontaneous breathing test in continuous positive airway pressure induced an increase in work of breathing, both in respiratory drive, as measured by EAdi and in respiratory mechanics, as measured by Pes. Oxygen consumption measured by Indirect Calorimetry does not seem to be a reliable tool to assess work of breathing in mechanically ventilated children.

### CO-69 Determinants of continued breastfeeding during hospitalization for bronchiolitis in infants less than 6 months of age (Bronchilact II)

#### Ben Gheriba Khalil^1^, Grimaud Marion^1^, Heilbronner Claire^1^, Roy Emeline^1^, Hadchouel Alice^1^, Renolleau Sylvain^1^, Rigourd Virginie^1^

##### ^1^Hôpital Necker Enfants Malades, Paris, France

###### **Correspondence:** Ben Gheriba Khalil - bg.khalil@gmail.com

*Annals of Intensive Care* 2018, **8(Suppl 1):**CO-69

**Introduction:** During the winter season 2015–2016 we had evaluated breastfeeding disruption after hospitalization for bronchiolitis in our hospital in infants under 6 month (n = 84). We observed 51% of mothers whose breastfeeding was stopped of modified. Clinical severity had no impact on breastfeeding but 63% of mothers stated that lack of support and advice was the first cause of breastfeeding disturbance. We conducted this second phase to evaluate the potential impact of actions to promote breastfeeding on unwanted weaning during hospitalization for bronchiolitis.

**Patients and methods:** This is a cross sectional study during two epidemic seasons of bronchiolitis in a tertiary care hospital. All patients aged 6 months or younger hospitalized with acute bronchiolitis and receiving at least partial breastfeeding were eligible for the study. Patients discharged at home whose parents accepted to be contacted by phone were included. A bundle of actions to promote breastfeeding in patients with bronchiolitis was implemented (posters, flyers, staff training, equipment with breast pumps) between the two epidemic seasons. The data was extracted from the charts and from a phone survey two weeks after discharge to evaluate breastfeeding in eligible patients in our hospital. Phase I (before action) had included 84 patients hospitalized between December 2015 and March 2016 in all wards hosting patients with bronchiolitis. Phase II (after action) included 50 patients hospitalized from October 2016 to December 2016. The data from phase II was compared with data from phase I.

**Results:** Fifty patients could be included during the second step of the study, with a mean age of 37 days. Breastfeeding was exclusive for 78% of mothers (vs 77% in phase I). The median length of stay was 6 days (vs 3 days in phase I). Twenty-one (42%) patients spent time in PICU vs. 32% in phase I, 3 21 needed intubation, 18 21 received non invasive ventilation for a median length of 4 days (vs. 3 days in phase I). The number of patients needing nutritional support was 35 50 (70%) during phase II vs. 44 84(52%) during phase I. After implementation of our actions, 40 50 (81%) mothers kept breastfeeding as before (vs. 49% in the previous epidemic season, p < 0.05), 4 mothers (8%) stopped, 4 (8%) switched to partial breastfeeding and 2 (4%) reduced without stopping.

**Conclusion:** Bronchiolitis is a high risk event for breastfeeding disruption but staff training and correct advices and support for mothers during hospitalization seems to diminish that risk.

### CO-70 Benefits of using a high temporal resolution database in the automatic real-time pediatric ARDS screening

#### Nardi Nicolas^1^, Sauthier Michael^1^, Brossier David^2^, Eltaani Redha^1^, Emeriaud Guillaume^1^, Jouvet Philippe^1^

##### ^1^CHU Sainte Justine Montréal, Canada; ^2^CHU de Caen, France

###### **Correspondence:** Nardi Nicolas - nicolas.nardi@hotmail.fr

*Annals of Intensive Care* 2018, **8(Suppl 1):**CO-70

**Introduction:** Pediatric acute respiratory distress syndrome (PARDS) is frequent in pediatric intensive care units (PICU), potentially lethal and the diagnosis is often missed or delayed (PALICC 2015). In PICU, data are usually recorded between 5 to 60 min which leads to only a minority of the arterial partial pressure of oxygen (PaO2) that are usable to calculate a valid oxygenation index (OI). If not available, PaO2 should be replaced by the SpO2 if < 98% to calculate the oxygen saturation index (OSI). Using a high temporal resolution (HTR) database that records data every 5–30 s, we aim to develop a relevant clinical algorithm of mass data aggregation to improve PARDS screening with the automatic OI and OSI calculation.

**Patients and methods:** All the patients admitted to our Pediatric ICU between May 2015 and August 2017 were included. The HTR and the electronic medical records (EMR) were queried through Structured Query Language (SQL) following these steps—(1) Data selection (2) Extraction to a linear format (3) Date and time synchronization (4) Data pivoting (5) Aggregation through a 10-min moving average (6) Hypoxemia calculation. Statistical analysis included proportions, correlations and Bland–Altman analysis.

**Results:** Between May 2015 and August 2017, 1793 patients (2210 stays) were admitted to the PICU. Approximately 46 million rows were retrieved from the databases including 19,189 PaO2 values. The algorithm was able to calculate 11,320 (59% of the PaO2) OI and 5204 OSI. The comparison between OI and OSI showed that 97.4% of the results were between the limits of agreements (− 17.2 + 10.6), a bias of − 3.3 and a correlation R2 = 0.643. The comparison between the OIs from the HTR and EMR databases showed that 94.6% of the results were between the limits of agreements (− 5.47 + 5.39), a bias of − 0.04 and R2 = 0.904.

**Conclusion:** Using a mass data aggregation algorithm on a HTR database allows more PaO2 to be used to calculate an OI than the usual EMR. The OI results differ slightly between the HTR and the EMR. The accuracy is probably in favor of the HTR because of the shorter time-lapse between the OI parameters. The OSI is possibly a biased OI surrogate and should be interpreted with caution. Our next step will be to measure the impact of the algorithm on the PARDS real-time diagnosis and PARDS severity categories.

### CO-71 Are beta-lactam concentrations adequate in severe sepsis and septic shock in children?

#### Chosidow Anaïs^1^, Benaboud Sihem^1^, Beranger Agathe^1^, Zheng Yi^1^, Moulin Florence^1^, Dupic Laurent^1^, Renolleau Sylvain^1^, Tréluyer Jean-Marc^1^, Oualha Mehdi^1^

##### ^1^Hôpital Necker Enfants-Malades, Paris, France

###### **Correspondence:** Chosidow Anaïs - anaischosidow@gmail.com

*Annals of Intensive Care* 2018, **8(Suppl 1):**CO-71

**Introduction:** Early administration of appropriate antibiotic therapy with adequate concentration is the cornerstone of the severe sepsis and septic shock’s treatment. Adult studies showed alteration of distribution and elimination which can lead to insufficient drug concentration in septic patients. In children, studies are lacking and antibiotic dosing may be suboptimal. We aim to describe the plasma concentration of the most used beta-lactam in critically ill children, to describe the rate of patients with suboptimal exposure and associating clinical and biological factors.

**Patients and methods:** This was a prospective, single center, observational study designed in 32 beds Pediatric Intensive Care Unit (PICU) and high dependency care at the Necker Hospital (Paris, France) from January 2016 to May 2017. Were included, children with severe sepsis or septic shock, aged less than 18 years and weighing more than 2.5 kg, and receiving one or more of the following antibiotics—amoxicillin, cefotaxime, cefazolin, ceftazidime, piperacillin-tazobactam, meropenem and imipenem for suspected or proven infection. Beta-lactam plasma concentrations were analysed using High Performance Liquid Chromatography.

**Results:** We enrolled 37 children (severe sepsis, n = 22 (59.5%) + septic shock, n = 15 (40.5%)) with a median age of 19 months (4–64. Bacteria were identified in 26 patients (70.3%). A total of 132 blood samples were analysed at a median of 2 days (1–12) following the onset of sepsis. Twenty-four patients (64.8%) had insufficient concentration (cefotaxime 7 14 (43%) + piperacillin-tazobactam, 10 13 (77%) + amoxicillin 6 7 (86%) + meropenem 3 6 (50%), cefazoline 1 4 (25%), imipenem 0 2 (0%) + ceftazidime 0 1 (0%)). Insufficient concentrations were associated with early measurements (< 72 h from the sepsis’ onset) (p = 0.035) and creatinine clearance increase (p = 0.01). Adequate concentrations were associated with small age (p = 0.048).
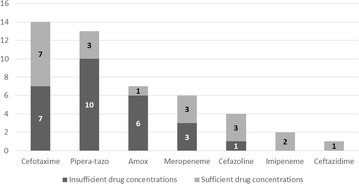


**Conclusion:** In conclusion, current standard beta-lactam dosing in children with severe sepsis or septic shock could be inadequate to reach the target concentrations. That could lead to the risk of clinical and bacteriological failures as well as the emergence of bacterial resistance. Further pharmacokinetic studies are mandatory to improve antibiotic therapy in this vulnerable population.

### CO-72 Predicting intradialytic hemodynamic instability in critically ill patients: derivation and validation of a bedside tissue hypoperfusion score

#### Bigé Naïke^1^, Dang Julien^1^, Attias Philippe^1^, Deryckere Stéphanie^1^, Joffre Jérémie^1^, Dubée Vincent^1^, Dumas Guillaume^1^, Preda Gabriel^1,^ Bourcier Simon^1^, Pichereau Claire^1^, Guidet Bertrand^1^, Maury Eric^1^, Ait-Oufella Hafid^1^

##### ^1^Hôpital Saint-Antoine - Service de Réanimation Médicale, Paris, France

###### **Correspondence:** Bigé Naïke - naikebige@gmail.com

*Annals of Intensive Care* 2018, **8(Suppl 1):**CO-72

**Introduction:** Intermittent hemodialysis is a key support therapy in ICU. Despite protocol-based optimization, intradialytic hemodynamic instability (IHI) remains a common complication and could account for mortality and delayed renal recovery. The identification of patients at high risk for IHI is crucial but remains poorly explored. Our objective was to test whether tissue perfusion parameters assessed at the bedside (mottling, index capillary refill time (iCRT), and lactate) predict IHI and to develop and to validate a predictive score of IHI.

**Patients and methods:** Prospective observational study in a 18-bed medical ICU in a tertiary university hospital including hemodialysis sessions performed for acute kidney injury. Exclusion criteria were patients with dark skin and dialysis performed in extreme emergency. Mean arterial pressure (MAP), mottling score, iCRT, and lactate were recorded just before starting hemodialysis. First episode of IHI requiring therapeutic intervention was recorded 60, 120, and 240 min after hemodialysis starting.

**Results:** Ninety-six hemodialysis sessions performed in 43 patients were recorded. Half of the patients received vasopressors (n = 43, 45%). IHI occurred in 22 (23%) sessions and was more frequent among patients receiving vasopressors (42 vs 7%, p < 0.0001). Mottling were more frequent (59 vs 26%, p = 0.005), lactate levels higher (2.8 [1.4–6.9] vs 1.1 [0.8–1.5] mmol L, p < 0.0001) and iCRT longer (3.3 [1.6–4.6] vs 1.1 [0.8–2.0] s, p < 0.0001) before sessions with IHI compared to sessions without, independently of MAP (p < 0.0001). The incidence of IHI increased with the number of tissue perfusion alterations (3, 11, 41, and 55% for 0, 1, 2, and 3 alterations, respectively, p < 0.0001). A tissue hypoperfusion score, defined as iCRT (seconds) + lactate level (mmol L) + 1 if mottling presence was predictive of IHI independently of MAP (OR 1.18 [1.05–1.32], p < 0.0001) with an AUROC of 86 [77–94] %. A threshold of 4.1 predicted IHI with a sensitivity of 82 [60–95]  %, and a specificity of 82 [72–90] %. The accuracy of this score was validated in a second prospective cohort including 115 hemodialysis sessions performed in 45 patients.
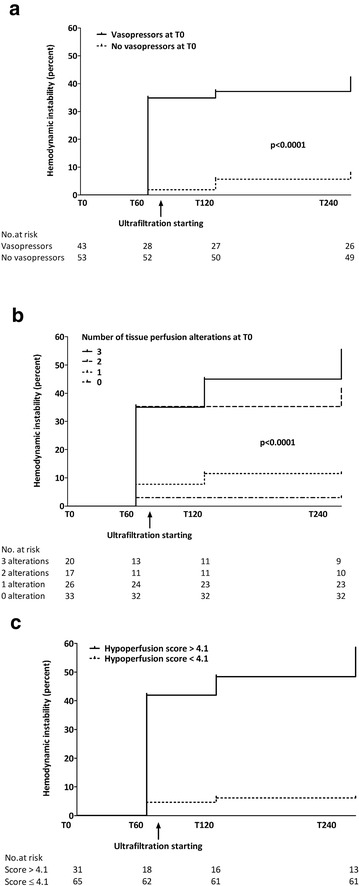


**Conclusion:** The incidence of IHI increases with the number of tissue perfusion alterations independently of MAP. At the bedside, a combined tissue hypoperfusion score including mottling score, iCRT and lactate level is helpful to identify patients at high risk of IHI.

### CO-73 Long term renal recovery in out-of-hospital cardiac arrest survivors

#### Ait Hamou Zakaria^1^, Jamme Matthieu^1^, Ben Hadj Salem Omar^1^, Dumas Florence^1^, Guillemet Lucie^1^, Bougouin Wulfran^1^, Pène Frédéric^1^, Mira Jean-Paul^1^, Cariou Alain^1^, Geri Guillaume^1^

##### ^1^Hôpital Cochin, Paris, France

###### **Correspondence:** Ait Hamou Zakaria - aithamou-zakaria@hotmail.fr

*Annals of Intensive Care* 2018, **8(Suppl 1):**CO-73

**Introduction:** Epidemiological data suggest an increased risk of long-term chronic kidney disease after acute kidney injury (AKI). In survivors of out-of-hospital cardiac arrest (OHCA), AKI is frequent and is associated with numerous factors of definitive renal injury. We made the hypothesis that AKI after OHCA was a strong risk factor of long-term chronic kidney disease (CKD). We aimed to evaluate renal outcome of OHCA survivors according the occurrence of AKI in ICU.

**Patients and methods:** We used the cohort of consecutive OHCA patients admitted between 2007 and 2012 in a tertiary medical ICU previously described (Geri et al. ICM. 2015). AKI was defined by Kidney Disease Improving Global Outcomes (KDIGO) criteria. Long-term creatinine level was the last blood creatinine assessment we were able to retrieve. The main outcome was the occurrence of CKD, defined by an estimated glomerular filtration rate (eGFR) lower than 60 mL min 1.73 m^2^ according to the MDRD equation. Long-term mortality was evaluated as well. Factors associated with CKD occurrence were evaluated by competing risk survival analysis (Fine Gray and Cox cause specific models providing sub-hazard ratio (sHR) and Cox sub-hazard (CSH)).

**Results:** Among the 246 OHCA patients who were discharged alive, we were able to retrieve the outcome of 133 patients (median age 55 [iqr 46, 68], 75.2% of male) who were included in the analysis. During a median follow-up time of 1.8 [0.8–2.5] years, CKD occurred in 17 (12.7%) patients and 24 (18%) patients died. A previous history of arterial hypertension (sHR = 3.28[1.15 + 9.39], p = 0.027 + CSH = 4.83 [1.57 + 14.9], p = 0.006), AKI during ICU stay (sHR = 3.72[1.40 + 9.84], p = 0.008 + CSH = 5.41[1.79 + 16.3], p = 0.003) and an age higher than 55 (sHR = 6.13[1.55 + 24.3], p = 0.009 + CSH = 2.16[1.72 + 43.8], p = 0.006) were independently associated with CKD occurrence. AKI was not associated with long-term mortality (sHR = 0.73 [0.27 + 1.99], p = 0.55 + CSH = 0.75 [0.28 + 2.01], p = 0.57).
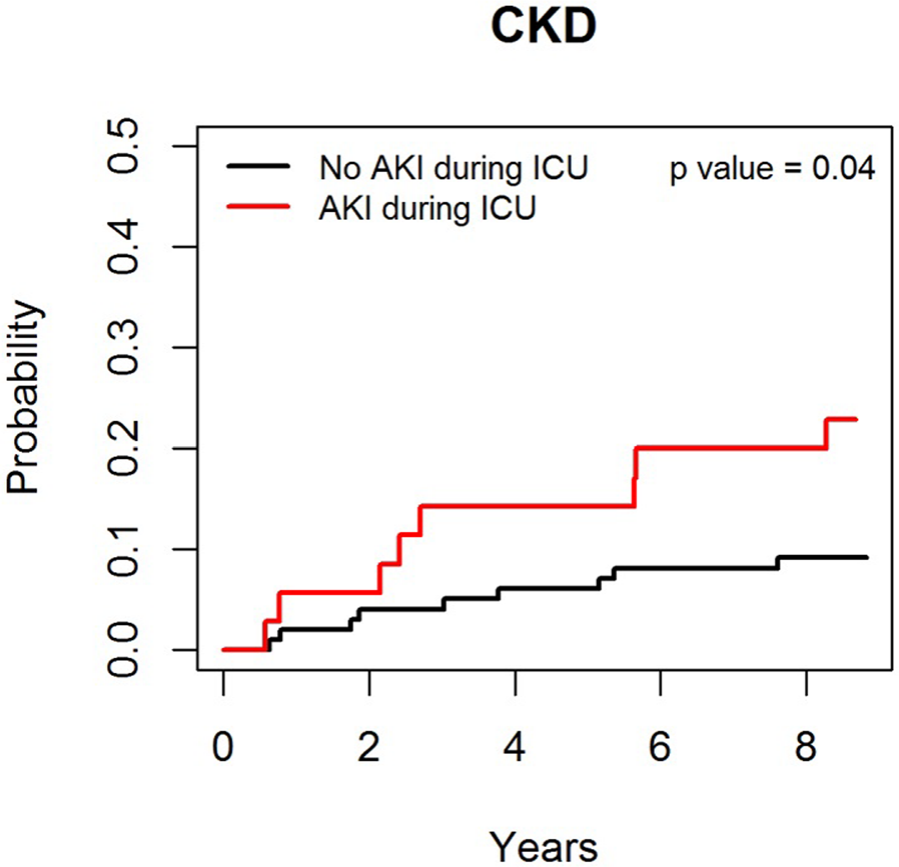


**Conclusion:** In OHCA survivors resuscitated from an OHCA, CKD was a frequent long-term complication. AKI during ICU stay was a strong determinant of long-term CKD occurrence.

### CO-74 Trends in acute kidney injury requiring dialysis in metropolitan France between 2009 and 2014

#### Garnier Fanny^1^, Couchoud Cécile^2^, Boudemaghe Thierry^1^, Landais Paul^3^, Moranne Olivier

##### ^1^CHU Carémeau, Nîmes, France; ^2^Agence de la Biomédecine, Saint Denis La Plaine, France; ^3^Laboratoire de Biostatistique, Montpellier, France

###### **Correspondence:** Garnier Fanny - garnier.fanny26@gmail.com

*Annals of Intensive Care* 2018, **8(Suppl 1):**CO-74

**Introduction:** Acute kidney injury requiring dialysis (AKID) is associated with high mortality. In several countries, epidemiology of AKID is lacking. Objectives of the study was to assess changes in standardized incidence, patients’ characteristics and hospital mortality in France.

**Patients and methods:** The study was conducted using the French database on hospitalizations (Program of Medicalization of Information Systems). It focused on adults hospitalized in metropolitan France between 2009 and 2014 and diagnosed with AKID according to the codes of Common Classification of Medical Procedures. We described and statistically compared crude and standardized incidence of AKID on gender and age, as well as comorbidity and mortality rates, principal diagnosis and dialysis modality. We also analyzed variables associated with mortality using logistic regression.

**Results:** Crude incidence of AKID increased from 459per million people (pmp) in 2009 (16,500 cases) to 512pmp (25,142 cases) in 2014. Standardized incidence increased from 475[468–482] in 2009 to 524 pmp[517–532] in 2013, an annual increase of 2%. AKID’s incidence was twofold higher for men. Patients’ median age was 68 years. Standardized incidence of AKID increased by 18, 8, 15 and 50% for the 60–69, 70–79, 80–89 and over 90 age groups respectively. The most common comorbidities were cardiocerebrovascular (67.3%), pulmonary (42%), chronic kidney disease (CKD) (32.5%), diabetes (27.9%), all of which increased significantly (excluding CKD). The five most frequent principal diagnoses were heart failure (17.7%), sepsis (13.4%), acute kidney injury (12.7%), digestive diseases (11.6%) and shock (8.7%), with significant increase in heart failure and digestive diseases. Number of stays in intensive care units increased from 80.3 to 83.9%. Continuous dialysis was the most widely used dialysis modality, with a utilization rate up from 56.9 to 61.8%. Intra-hospital mortality was stable at 47%. Factors associated with higher mortality were: advanced age, intensive care admission (OR 3.33), introduction to dialysis exceeding 8 days (OR 1.46), cardiocerebrovascular comorbidities (OR 1.12), pulmonary comorbidities (OR 1.39), cancer (OR 1.33), hepatic comorbidities (OR 1.85). Diagnoses associated with higher mortality, compared with CKD, were: shock (OR 5.99), respiratory diseases (OR 4.81), sepsis (OR 4.44), digestive diseases (OR 4.10) and heart failure (OR 3.33).

**Conclusion:** It is the first study in France showing a significant increase in AKID’s standardized incidence among adults and a significant change in patients’ characteristics.

### CO-75 Prolonged heparin-free hemodialysis sessions with calcium-free citrate-containing dialysate in critically ill patients with a moderate to high risk of bleeding

#### Faguer Stanislas^1^, Saint-Cricq Morgane^1^, Lavayssiere Laurence^1^, Setbon Nicolas^1^, Nogier Marie-Béatrice^1^, Kamar Nassim^1^, Cointault Olivier^1^

##### ^1^Hôpital Rangueil, Toulouse, France

###### **Correspondence:** Faguer Stanislas - stanislas.faguer@gmail.com

*Annals of Intensive Care* 2018, **8(Suppl 1):**CO-75

**Introduction:** Many critically ill patients have a moderate to high risk of bleeding but they also require prolonged intermittent dialysis to ensure a negative water balance without hemodynamic adverse events. Thus, a heparin-free easy-to-use anticoagulation within the dialysis circuit is needed but, to date, usual protocols (iterative saline flushes, heparin grafted membranes) lead to 20–50% of premature clotting and sessions that last greater than 240 min are rarely achievable. We assessed the safety and efficiency of heparin-free regional citrate anticoagulation of the dialysis circuit using a calcium-free citrate-containing dialysate, with calcium reinjected according to ionic dialysance (an online measure of the instantaneous clearance of small molecules available in most of dialyzers).

**Patients and methods:** We prospectively reported the clotting events that occurred during all the heparin-free dialysis sessions that were performed with a regional anticoagulation based on calcium-zero citrate-containing dialysate (Citrasate, Hemotech, France) between January 2016 and August 2017 in a 28-beds ICU.

**Results:** A total of 186 dialysis sessions were performed in 70 patients (mechanical ventilation n = 93 + norepinephrine n = 20). Median duration of dialysis was 240 min (IQR, 240–300 + maximum 360 min), and median ultrafiltration volume was 2 L (IQR 1.3–2.6). When assessed, urea and Beta2-microglobulin reduction rates were 64.5% ± 0.4% and 48% ± 0.13%, respectively. Postfilter ionized calcium was 0.35 ± 0.17 and 0.38 ± 0.14 mmol L at 1 and 3 h, respectively, within the extracorporeal circuit. A major clotting event that led to premature termination of the session occurred in only 5186 sessions (2.7%). In these five cases, major catheter dysfunction occurred before clotting within the circuit. Prefilter ionized calcium remained within narrow ranges (before after change +0.07 ± 0.006 mmol L), and total-to-ionized calcium ratio, a surrogate marker for citratemia, was unchanged and always below 2.5. In 85 sessions, no ionized calcium measurement was required.

**Conclusion:** Dialysis anticoagulation with calcium-free citrate containing dialysate is an easy-to-use, efficient, and inexpensive form of heparin-free regional anticoagulation. Calcium reinjection according to ionic dialysance allows prolonged hemodialysis sessions in critically ill patients without the need to systemically monitor ionized calcium. Sessions can be safely extended according to the hemodynamic tolerance to ensure an adequate dose of dialysis and a negative water balance, a major point in patients with severe AKI.

### CO-76 Renal replacement therapy initiation strategies for critically ill patients with acute on chronic renal failure: a post hoc analysis of the AKIKI trial

#### Gaudry Stéphane^1^, Verney Charles^1^, Hajage David^2^, Martin-Lefèvre Laurent^3^, Pons Bertrand^4^, Boulet Eric^5^, Boyer Alexandre^6^, Chevrel Guillaume^7^, Lerolle Nicolas^8^, Carpentier Dorothee^9^, De Prost Nicolas^10^, Lautrette Alexandre^11^, Bretagnol Anne^12^, Mayaux Julien^2^, Nseir Saad^13^, Schortgen Frederique^10^, Tubach Florence^14^, Ricard Jean-Damien^1^, Dreyfuss Didier^1^

##### ^1^Hôpital Louis Mourier, Colombes, France; ^2^Hôpital Pitié-Salpêtrière, Paris, France; ^3^CHU de la Roche sur Yon, France; ^4^CHU DE Pointe à Pitre, France; ^5^CHU de Pontoise, France; ^6^CHU de Bordeaux, France; ^7^CHU de Vienne, France; ^8^CHU d’Angers, France; ^9^CHU de Rouen, France; ^10^HU Henri Mondor, Paris, France; ^11^CHU de Clermont-Ferrand, France; ^12^CHU d’Orléans, France; ^13^CHU de Lille, France; ^14^Hôpital Bichat, Paris, France

###### **Correspondence:** Gaudry Stéphane - stephanegaudry@gmail.com

*Annals of Intensive Care* 2018, **8(Suppl 1):**CO-76

**Introduction:** Acute kidney injury (AKI) is frequent and associated with poor intensive care unit (ICU) outcome in critically ill patients. Superimposed AKI on chronic kidney disease (CKD) is associated with long-term risk of death and end-stage renal disease. We aimed to investigate the effect of an early compared to a delayed RRT initiation strategy in patients with severe AKI on CKD.

**Patients and methods:** Post-hoc analysis of the AKIKI trial. In this multicenter pragmatic RCT, patients with severe AKI (stage 3 of KDIGO classification) were randomly assigned to either an early or a delayed RRT initiation strategy. The subgroup of patients with CKD was a priori defined as a preexisting creatinine clearance < 60 ml min in stable condition. The primary endpoint was 60-day mortality. Secondary endpoints included—number of patients who actually received RRT, RRT-free days, mechanical ventilation-free days and vasopressor-free days, ICU and hospital length of stay, time to renal function recovery, day-28 and day-60 dependence on RRT.

**Results:** Of the 619 patients, 60 (10%) had CKD. We found significant treatment effect heterogeneity according to the CKD status (test for interaction p = 0.006). Patients with CKD had an increased risk of death (HR 2.50 (95% CI 1.23–5.00, p = 0.009) with the early strategy (Fig. 1). Delayed strategy allowed avoidance of RRT in 45% of patients with CKD. Mechanical ventilation-free days, ICU and hospital length of stay, dependence on RRT at day-28 and day-60 did not significantly differ between RRT strategies. The number of vasopressor-free days was significantly higher in patients with CKD assigned to the delayed strategy (5.5 [0–20] vs 22.0 [3–26], p = 0.02). We found a trend for an earlier renal function recovery with the delayed RRT strategy but this difference did not reach significance (p = 0.06).

**Fig. 1:**
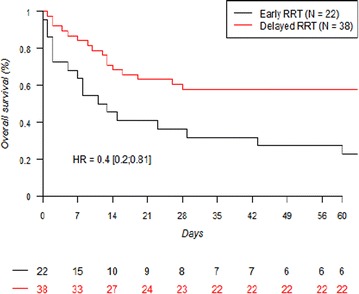
60-day survival in patients with early- and delayed-RRT

**Conclusion:** Early renal replacement therapy initiation strategy was associated with a significant increase in 60-day mortality in the subgroup of patients with CKD. Delaying RRT initiation may benefit patients with severe AKI on CKD.

### CO-82 Background EEG reactivity and neurologic outcome after cardiac arrest in the Parisian registry

#### Benghanem Sarah^1^, Paul Marine^1^, Charpentier Julien^1^, Rouhani Said^1^, Ben Hadj Salem Omar^1^, Guillemet Lucie^1^, Llitjos Jean Francois^1^, Legriel Stephane^1^, Pene Frederic^1^, Chiche Jean Daniel^1^, Mira Jean-Paul^1^, Dumas Florence^1^, Cariou Alain^1^

##### ^1^Hôpital Cochin, Paris, France

###### **Correspondence:** Benghanem Sarah - benghanem.sarah89@gmail.com

*Annals of Intensive Care* 2018, **8(Suppl 1):**CO-82

**Introduction:** Brain injury is the first cause of death after cardiac arrest (CA) and multimodal neuroprognostication is a cornerstone of post-resuscitation care. Among the different usable information provide by electroencephalogram (EEG), the aim of this study was to evaluate the predictive value of EEG reactivity regarding neurological outcome at discharge.

**Patients and methods:** Using our prospective registry of successfully resuscitated patients admitted to a cardiac arrest center between January 2007 and 2016, we studied all consecutive comatose patients still alive at 48 h and in whom at least one EEG was performed during coma. In addition to usual clinical findings, we collected EEG (patterns and reactivity, status epilepticus) and somatosensory evoked potentials characteristics. The EEG reactivity was evaluated by a blinded neurophysiologist and was defined as a reproducible change of the tracing (in amplitude or frequency) provoked by an auditory and a nociceptive standardized stimulation. We evaluated the predictive values of persistent lack EEG reactivity and other indicators regarding their respective ability to predict a favorable or unfavorable outcome. Recovery of a level 1 or 2 on the Cerebral Performance Category (CPC) scale at discharge was considered as a favorable outcome, as opposed to recovery of a CPC level 3–5 (unfavorable outcome).

**Results:** We included 428 patients who were mostly male (71%), with median age of 63 years. CA occurred in a public place in 32% of cases, and it was witnessed in 89% of cases. Bystander CPR was initiated in 61% patients and the initial cardiac rhythm was shockable in 49% patients. Median time to EEG was 3 days (1–4) and 43% of patients were still sedated during the examination. A favorable neurologic outcome was observed in 85 patients (20%). An EEG reactivity was present in 78 85 patients (92%) with favorable outcome and in 117 343 patients (34%) with unfavorable outcome. The positive predictive value (PPV) of a persistent EEG reactivity for prediction of favorable outcome was 40% (IC 95% 33–47). By contrast, the PPV of lost EEG reactivity for prediction of unfavorable outcome was 97% (IC 95% 94–99) with a false positive rate (FRP) of 2.8% (1.2–4.4).

**Table:** Prognostic value of clinical and EEG parameters for poor outcome

	Sensitivity (IC 95%)	Specificity (IC 95%)	NPV (IC 95%)	PPV (IC 95%)	FPR (IC 95%)
Absent pupillary reflex	40.82 (35.6–46.2)	78.82 (68.6–86.9)	24.81 (19.8–30.4)	88.61 (82.6–93.1)	11.4 (8.4–14.4)
Absent motor Glasgow	74.92 (70–79.4)	37.65 (23.4–48.8)	27.12 (19.4–36.1)	82.90 (78.2–86.9)	17.1 (13.5–20.7)
Areactive first EEG background	63.66 (58.2–68.9)	92.86 (85.1–97.3)	40 (33.1–47.2)	97.16 (93.9–98.9)	2.8 (1.2–4.4)
Bilaterally absent N20 on SSEP	54.21 (46.9–61.4)	100 (79.4–100)	15.53 (9.2–24)	100 (96.5–100)	0 (0–1.8)
Burst suppression	7.87 (5.3–11.3)	100 (95.8–100)	21.20 (12.3–25.5)	100 (87.2–100)	0 (0–0.9)

*EEG* electroencephalogram, *SSEP* short-latency somatosensory evoked potentials, *PPV* positive predictive value, *NPV* negative predictive value, *FPR* false positive rate

**Conclusion:** In this population of post-cardiac arrest patients, the presence of EEG reactivity was poorly predictive of a favorable neurologic outcome. The absence of reactivity was highly predictive of unfavorable outcome. In combination with other indicators, searching for EEG reactivity may have important implications in the neuroprognostication process.

### CO-83 Morbidity and mortality of crystalloids vs. colloids in surgery: a subgroup analysis of a randomised trial

#### Heming Nicholas^1^, Lamothe Laure^1^, Jaber Samir^2^, Trouillet Jean Louis^3^, Martin Claude^3^, Chevret Sylvie^3^, Annane Djillali^1^

##### ^1^Hôpital Raymond Poincaré, Garches, France; ^2^CHU de Montpellier, France; ^3^Hôpital de la Salpetrière, Paris, France

###### **Correspondence:** Heming Nicholas - nicholas.heming@aphp.fr

*Annals of Intensive Care* 2018, **8(Suppl 1):**CO-83

**Introduction:** The safest resuscitation fluid for surgical patients remains uncertain. We sought to compare the safety and efficacy of either crystalloids or colloids in surgical critically ill patients.

**Patients and methods:** Subgroup analysis of the multicenter randomized trial. All critically ill surgical patients were included in the current analysis. 741 patients were randomized to receive crystalloids or colloids. Patients were stratified as suffering from hypovolemia due to sepsis, trauma or other causes. Primary outcome measure was death by day 28. Secondary outcome measure included death by day 90.

**Results:** Crystalloids (n = 356) or colloids (n = 385) were administered for all fluid interventions other than fluid maintenance throughout the ICU stay. 484 (65.3%) patients were male, median [IQR] age was 66 [52 + 76] years. Surgery was unscheduled in 543 (73.3%) cases. Mortality by day 28 did not significantly differ for crystalloids 84 (23.6%) vs. colloids 100 (26%) (Adjusted odds ratio 0.86 [IC 95%, 0.61 to 1.21] + p = .49). Mortality by day 28 did not significantly differ among all three predefined strata (p = .52). Death by day 90 (111 (31.2%) vs. 122 (31.7%) (Adjusted odds ratio 0.97 [IC 95%, 0.70 to 1.33] + p = .86) did not significantly differ between groups.

**Conclusion:** This subgroup analyses of a randomized controlled trial, found no survival benefit when comparing crystalloids to colloids in critically ill surgical patients.

### CO-84 Transient or persistent fluid responders: toward a new definition of fluid responsiveness? The FC-Rev study

#### Roger Claire^1^, Zieleskiewicz Laurent^2^, Demattei Christophe^1^, Lakhal Karim^3^, Piton Gaël^4^, Constantin Jean-Michel^6^, Chabanne Russell^6^, Faure Jean Sébastien^6^, Majhoub Yazine^7^, Desmeulles Isabelle^8^, Quintard Hervé^9^, Lefrant Jean Yves^1^, Muller Laurent^1^

##### ^1^CHU de Nîmes, France; ^2^CHU de Marseille, France; ^3^CHU de Nantes, France; ^4^CHU de Besancon, France; ^6^CHU de Clermont Ferrand, France; ^7^CHU d’Amiens, France; ^8^CHU de Caen, France; ^9^CHU de Nice, France

###### **Correspondence:** Roger Claire - claire.roger@chu-nimes.fr

*Annals of Intensive Care* 2018, **8(Suppl 1):**CO-84

**Introduction:** Goal of a fluid challenge (FC) is in fine to increase the stroke volume (SV) or the cardiac index (CI) when an episode of hypovolemia or a preload dependence status are suspected. FC is one of the most common practices in ICUs, however, the way to assess the response to FC is not standardized. The present study aimed to evaluate whether the trans-thoracic echocardiographic (TTE) assessment of the response to FC immediately at the end of the infusion or delayed 20 min later could affect the results of the FC.

**Patients and methods:** Prospective, observational, multicentre study including all ICU patients in septic shock requiring a FC. Were excluded patients with—arrhythmias, poor echogenicity and severe mitral or aortic regurgitation. FC was performed administering 500 mL of crystalloids over 10 min. Fluid responsiveness was defined as a > 15% increase in stroke volume (SV). The following echocardiographic parameters were recorded—E wave, A wave, E A ratio, velocity–time integral (VTI), Ea wave and Sa wave. MAP, HR and TTE variables were collected at baseline (T0), at the end of fluid challenge (T10) and 10 (T20) and 20 min (T30) after the end of fluid challenge. Quantitative data are expressed as mean and standard deviation (SD) or median and interquartile (IQR), according to their distribution. Qualitative data are expressed as absolute number and frequency (%).

**Results:** From May 20th 2014 to January 7th 2016, a total of 143 patients were enrolled in 11 French ICUs (mean age—64 ± 14 years, median IGS II—53 [43–63], median SOFA score—10 [8–12]). Among the 76 143 (53%) patients responders to FC at T10, 37 patients were transient responders (TR), i.e. became non-responders at T30 (49%, 95% CI = [37–60]) and 39 (51%, 95% CI = [38–62])) patients were persistent responders (PR), i.e. remained responders at T30. Among the 67 non-responders (NR) at T10, 4 became responders at T30, (6%, 95% CI = [1.9–15.3]). In the subgroup analysis, no statistical difference in haemodynamic and echocardiographic parameters was found between non-responders, transient responders and persistent responders (Fig. 1).

**Fig. 1:**
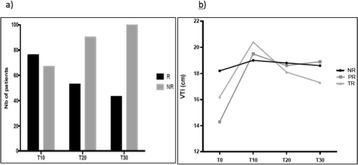
**a** Proposition of R and NR at the end (T10), 10 min (T20) and 20 min (T30) after the FC. **b** VTI time course during FC (T0 to T10) and during the 20 min after the FC (T10 to T30). *R* responders, *NR* non-responders, *PR* persistent responders, *TR* transient responders

**Conclusion:** The present study shows that, after a 15% VTI increase at the end of the FC, VTI returns to baseline at 30 min in half of the responders. Blood volume status (normo or hypovolemia) before initiating the fluid infusion could explain the transient or persistent response to FC observed in septic patients.

### CO-85 Mottling score is a strong predictor of Day-14 mortality in sepsis patients independently of catecholamine dosing and other tissue hypoperfusion parameters

#### Dumas Guillaume^1^, Joffre Jérémie^1^, Hariri Geoffroy^1^, Bigé Naike^1^, Baudel Jean-Luc^1^, Razach Abdallah Idriss^1^, Chevret Sylvie^2^, Guidet Bertrand^1^, Ait Oufella Hafid^1^

##### ^1^Hôpital Saint-Antoine, Paris, France; ^2^Hôpital Saint Louis, Paris, France

###### **Correspondence:** Dumas Guillaume - dumas.guillaume1@hotmail.fr

*Annals of Intensive Care* 2018, **8(Suppl 1):**CO-85

**Introduction:** Sepsis is a frequent critical condition. Mottling score, an hypoperfusion parameter, is well correlated with outcome. However, uncertainties persist regarding its value not only as a marker of patient severity but also as an independent predictor of mortality and treatment efficacy.

**Patients and methods:** We performed a post hoc analysis of four published prospective studies including sepsis patients with or without shock. We analyzed the relationship between the mottling score (from 0 to 5) and Day-14 mortality according to other prognosis covariates such as catecholamine dosing, urine output and plasma lactate levels. First, factors associated with outcome were determined by multivariate analysis. Second, mottling score-by-covariate interaction was studied to better understand its effect on mortality. Finally, effect of mottling score variation at different time point (H0–H6–H12–H24) was assessed.

**Results:** 274 patients were included. SAPSII at admission was 54 [41.2–70.0]. Six hours after ICU admission, 210 (76.6%) patients received catecholamine (norepinephrine (73.2%)), median dose of 0.3 [0.05–0.8] µg Kg min. At H6, the mottling score distribution was—grade 0 in 126 patients (48.6%), grade 1 in 36 (13.9%), grade 2 in 29 (11.2%), grade 3 in 29 (11.2%), grade 4 in 14 (5.4%) and grade 5 in 25 (9.7%). Day-14 mortality rate was 37.2%. Factors associated with Day-14 mortality were—mottling score (OR 2.16 [1.63–2.86]), lactate level > 5 mmol l (OR 3.74 [1.35–10.35]), urine output < 0.5 ml Kg h (OR 2.5 [1.09–5.7]) and SAPSII (OR 1.04 [1.02–1.06]). The predictive value of mottling score was not affected by catecholamine dosing (p = 0.45). OR of mottling score ranged from 2.38 [1.37–4.12] for patients without catecholamine to 3.84 [1.98–7.43] for patients receiving > 0.8 μg/kg/kg min. We did not find any significant interaction between mottling score prognosis value and urine output (p = 0.62) or lactate concentration (p = 0.09). A decrease of mottling score between the first 6 h of resuscitation was associated with outcome improvement (adjusted on SAPSII, p = 0.001).

**Conclusion:** Our results support the high prognostic value of mottling score for 14-day mortality, independently of catecholamine dosing and other hypoperfusion parameters. Mottling score variation during resuscitation is a strong predictor of mortality.

### CO-86 Microcirculation in refractory cardiogenic shock on veno-arterial extracorporeal membrane oxygenation

#### Chommeloux Juliette^1^, Monteiro Santiago^1^, Franchineau Guillaume^1^, Lebreton Guillaume^1^, Bréchot Nicolas^1^, Hekimian Guillaume^1^, Leprince Pascal^1^, Luyt Charles-Edouard^1^, Combes Alain^1^, Schmidt Matthieu^1^

##### ^1^APHP, Paris, France

###### **Correspondence:** Chommeloux Juliette - juliette.chommeloux@gmail.com

*Annals of Intensive Care* 2018, **8(Suppl 1):**CO-86

**Introduction:** Numerous studies have identified a relationship between microcirculation disorders and poor outcomes in cardiogenic shock patients. Despite the increasing interest for Veno-Arterial Extra Corporeal Membrane Oxygenation (VA-ECMO), data on microcirculation in this context are scarce. Thus, we aimed to describe the evolution of microcirculation and its relationship with the outcomes of patients on VA-ECMO for refractory cardiogenic shock.

**Patients and methods:** Consecutive patients with refractory cardiogenic shock (cardiac arrest excluded) who required VA-ECMO were included. Microcirculation evaluation by video microscopy, global haemodynamic and Doppler echocardiography variables were obtained before and 2, 4, 12, 24 and 48 h after ECMO initiation. Patients who survived had additional evaluations six hours before and after VA-ECMO removal.

**Results:** Fourteen patients (median age 58 (interquartile range 56–62) years + SOFA score 14 (12–18) were included. Myocardial infarction (33%) was the main cause for shock. Mortality on VA-ECMO was 57%, whereas ECMO was successfully weaned in 6 (33%) patients. Proportion of Perfused Vessel (PPV), Perfused Vessel Density (PVD), Micro Flow Index (MFI) and Heterogeneity Index (HI) were severely impaired before ECMO. Re-establishing high and stable peripheral blood flow with VA-ECMO led to a rapid decrease in heart rate and vasoactive inotropic support and significantly improved all microcirculation parameters within 12 h. Total vessel density and PVD, measured before and after ECMO initiation, were better in patients successfully weaned from ECMO (p < 0.05) (Fig. 1).

**Fig. 1:**
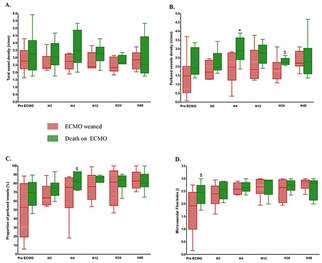
Horizontal line inside the box plot is the median; lower and upper box limis are 25th percentile to 78th percentile. T-bars represent the 10th and 90th percentiles. *p < 0.05; $p < 0.1

**Conclusion:** Cardiovascular support with ECMO-VA rapidly improved macro and microcirculation in refractory cardiogenic shock patients. Total vessel density and perfused vessel density were significantly better in survivors 12 h after ECMO initiation and might therefore help to predict outcomes. Further studies are now needed to better define the utility of this technology in larger groups of VA-ECMO patients.

### CO-93 WITHDRAWN

### CO-94 WITHDRAWN

### CO-95 WITHDRAWN

### CO-96 WITHDRAWN

### CO-97 Diagnostic, management and outcome of thyroid storm in intensive care unit: a retrospective multicenter study

#### Bourcier Simon^1^, Coutrot Maxime^2^, Sonneville Romain^3^, De Montmollin Etienne^1^, Persichini Romain^3^, Aubron Cécile^4^, Carreira Serge^5^, Nseir Saad^7^, Barbier François^8^, Razazi Keyva^9^, Hraiech Sami^9^, Terzi Nicolas^10^, Delmas Clément^11^, Peigne Vincent^12^, Lautrette Alexandre^13^, Aissaoui Nadia^14^, Kimmoun Antoine^15^, Demoule Alexandre^1^, Maizel Julien^16^, Schnell David^17^, Ferré Alexis^18^, Azoulay Elie^19^, Reuter Danielle^20^, Combes Alain^1^, Schmidt Matthieu^1^

##### ^1^Hôpital Pitié-Salpêtrière, Paris, France; ^2^Hôpital Bichat, Paris, France; ^3^CHU de La Réunion de Saint-Denis, France; ^4^CHRU de Brest, France; ^5^Hôpital Saint-Camille, Bry-Sur-Marne, France; ^6^CHRU de Lille, France; ^7^CHR d’Orléans, France; ^8^CHU Henri Mondor, Créteil, France; ^9^Hôpital Nord, Marseille, France; ^10^CHU de, France; ^11^CHU Toulouse, France; ^12^CH Métropole de Savoie, Chambéry, France; ^13^CHU Gabriel Montpied, Clermont-Ferrand, France; ^14^Hôpital Européen Georges Pompidou, Paris, France; ^15^CHU Nancy, VandŒuvre-Les-Nancy, France; ^16^HU d’Amiens Picardie, France; ^17^CH d’Angoulême, France; ^18^CH de Versailles - Le Chesnay, France; ^19^CHU Saint-Louis, Paris, France; ^20^CH Sud Francilien, Corbeil-Essonnes, France

###### **Correspondence:** Bourcier Simon - simon_bourcier@hotmail.com

*Annals of Intensive Care* 2018, **8(Suppl 1):**CO-97

**Introduction:** Thyroid storm is a rare but life-threatening disease related to thyrotoxicosis. It can lead to multiple organ failure including cardiovascular disorders or neurological impairment. To date, data on this disease in ICU patients are scarce and limited to case reports. We therefore aimed to describe clinical presentation, outcomes and management of thyroid storm in ICU patients.

**Patients and methods:** Local diagnoses coding database (from January 2000 to July 2017) from 22 French ICU were interrogated for main and secondary diagnoses codes including thyrotoxicosis based on the International Classification of Disease 10th revision. Thereafter two investigators reviewed all the medical records selected. Inclusion criteria were thyroid storm based on the diagnostic criteria of the Japan Thyroid Association (T. Satoh, Endocrine Journal 2016). It combines thyrotoxicosis with elevated levels of free triiodothyronine (FT3) or free thyroxine (FT4) with at least two of the following symptoms—central nervous system manifestation, fever, tachycardia > 130 bpm, congestive heart failure, or total bilirubin level more than 50 micromol/l. Clinical presentation, therapy used, and outcome were recorded.

**Results:** Sixty-two patients (median age 57 years (interquartile range 44–67) + SAPS II 45 (32–66) were included. Thyroid storm was the first manifestation of thyrotoxicosis in 18 (29%) patients. Graves’ disease (27%), amiodarone induced thyroiditis (26%), autoimmune thyroiditis (11%), and toxic multinodular goitre (10%) were the main causes of hyperthyroidism. Amiodarone, thyroid hormone toxicity, antithyroid drugs withdrawal or infectious trigger were identified in 59 (95%) patients. Organ support including mechanical ventilation, catecholamine infusion, renal replacement therapy and veno-arterial ECMO were used in 34, 31, 11, and 12 patients, respectively. Main thyroid storm treatments included antithyroid drugs (81%), betablockers (69%), corticosteroids (45%), and plasmapheresis (13%). Lastly, ICU-mortality was 16%, with multiple organ failure responsible of death in all patients.

**Conclusion:** Although its incidence appears low, ICU physicians should be aware of the multiple clinical features of thyroid storm. Our preliminary data reported various specific therapeutic management of this potentially fatal disease.

### CO-98 Early differentiation of Shiga toxin-associated hemolytic uremic syndrome in critically ill adults with thrombotic microangiopathies—a case control study

#### Joseph Adrien^1^, Rafat Cédric^1^, Zafrani Lara^1^, Mariani Patricia^4^, Veyradier Agnès^4^, Hertig Alexandre^1^, Rondeau Eric^1^, Mariotte Eric^4^, Azoulay Elie^4^

##### ^1^Hôpital Tenon, Paris, France; ^2^CHU Robert Debré, Paris, France; ^3^Hôpital Lariboisière, Paris, France; ^4^Hôpital Saint Louis, Paris, France

###### **Correspondence:** Joseph Adrien - adrien.joseph@hotmail.fr

*Annals of Intensive Care* 2018, **8(Suppl 1):**CO-98

**Introduction:** Thrombotic microangiopathies (TMAs) are severe diseases which often require admission in an intensive care unit (ICU). Prompt initiation of targeted therapies is required for atypical hemolytic uremic syndrome (aHUS) and thrombotic thrombocytopenic purpura (TTP), but no specific therapy is consensual for Shiga toxin-associated hemolytic uremic syndrome (STEC-HUS). Thus, rapid differentiation of STEC-HUS is mandatory to tailor the initial treatment. Furthermore, apart from large outbreaks, characteristic features of this syndrome in adults have not been described.

**Patients and methods:** In this study, we retrospectively compared the characteristics of STEC-HUS, aHUS and TTP patients at admission in two expert ICUs. Patient were included if they presented with the triad of mechanical hemolytic anemia, thrombocytopenia and organ damage, and TMAs were classified using international criteria. Other causes than STEC-HUS, aHUS and TTP were excluded.

**Results:** Amongst 236 TMAs admitted between September 2003 and January 2017, 12 STEC-HUS, 21 aHUS and 91 TTP were included. STEC-HUS patients were older (64) than aHUS (36, p = 0.02) and TTP patients (43, p < 0.01). They presented with more frequent digestive symptoms (92 versus 62 and 37% for aHUS and TTP, p = 0.01 and < 0.01), but bloody diarrhea was rare (17%) and non-statistically different from other TMAs. Confusion was more frequent in STEC-HUS (33%) than aHUS patients (5%, p = 0.05). Biologically, STEC-HUS patients displayed elevated fibrinogen levels (5.1 vs 3.2 and 3.6 for a HUS and TTP, both p < 0.01) and severe renal failure. Forty-two percent required renal replacement therapy and 83% were treated with plasma exchange before the distinction from other TMAs could be made. Only 1 (8%) STEC-HUS patient died in the ICU (Fig. 1).

**Fig. 1:**
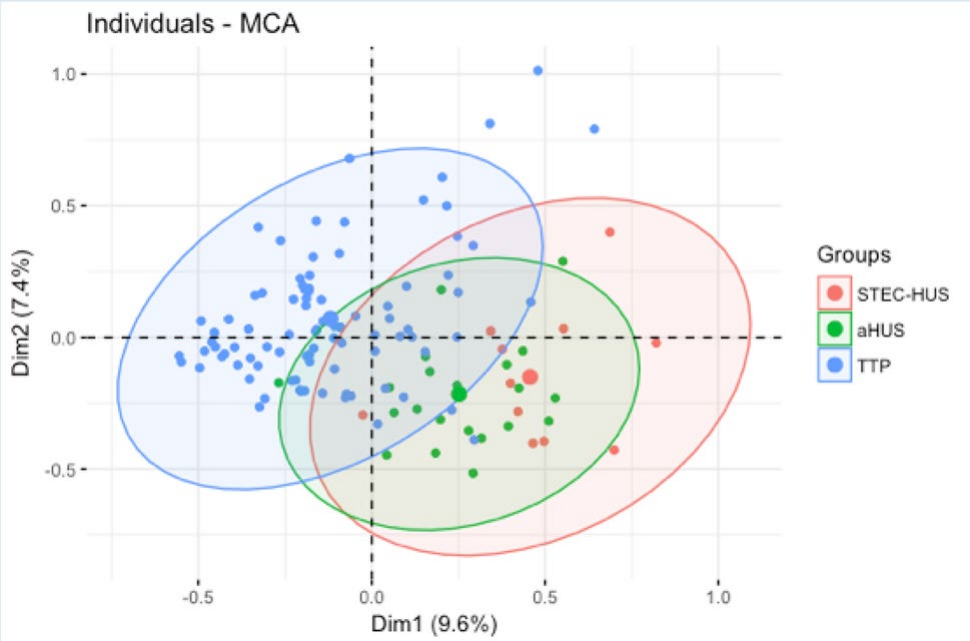
Multiple correspondence analysis of STEC-HUS, aHUS and TTP individuals

**Conclusion:** Characteristics supposed to identify STEC-HUS are largely shared with other TMAs. In particular, the differential diagnosis between aHUS and STEC-HUS appears to be more difficult than the stereotypical description derived from pediatric studies.

### CO-99 Severe hyperglycemia in 1000 ICU patients: a higher mortality rate and a higher incidence of diabetes in a long-term follow-up study

#### Ben Hamou Adrien^1^, Robriquet Laurent^1^, Kipnis Eric^1^, Voisin Benoit^1^, Preau Sebastien^1^, Tamion Fabienne^2^, Vanbaelinghem Clement^3^, Thellier Damien^4^, Du Cheyron Damien^5^, Bignon Anne^1^, Poissy Julien Jaillette^1^, Emmanuelle, Jourdain Merce^1^

##### ^1^CHRU LILLE, Lille, France; ^2^CHU ROUEN, Rouen Cedex, France; ^3^CH ROUBAIX, Roubaix, France; ^4^CH TOURCOING, Tourcoing, France; ^5^CHU CAEN, Caean, Francezz

###### **Correspondence:** Ben Hamou Adrien - adrien.benhamou@hotmail.fr

*Annals of Intensive Care* 2018, **8(Suppl 1):**CO-99

**Introduction:** Stress hyperglycemia frequently occurs in critically ill patients independently of preexisting diabetes. Some studies suggest an association with poor prognosis. The aim was to evaluate 28 and 90-day mortality after admission to the intensive care unit and the incidence of diabetes during follow-up at 6 and 12 months in patients who presented a severe hyperglycemia in their first 3 days in the ICU (glycemia > 200 mg dL, > 2 samples).

**Patients and methods:** We conducted a prospective multicentric study between April 2012 and August 2016 in French ICUs. Patient’ characteristics, medical history, blood tests, aetiology of ICU admission and organ dysfunctions were collected. Patients were divided into 4 groups—diabetic patients with severe hyperglycemia (HD), non-diabetic with severe hyperglycemia (HND), diabetic without severe hyperglycemia (NHD) and non-diabetic without severe hyperglycemia (NHND). Kaplan–Meier estimator was used to analyze survival adjusted to disease severity (Logistic Organ Dysfunction, Simplified Acute Physiology Score, Knaus, McCabe scores) and Kaplan–Meier survival curves were compared by Cox proportional hazards test. Fasting glycaemia above 126 mg dL was used to diagnose diabetes at 6 or 12 months.

Adrien BEN HAMOU The Kaplan–Meier survival curve Abstract SRLF 2018.

**Results:** 991 out of the 1000 enrolled patients were analyzed (62% men). Median age was 61 years old, median Body Mass Index was 27 kg m^2^ + 236 patients (24%) were diabetic. Among the 991 patients, 413 (42%) presented severe hyperglycemia and had a significant increase in mortality at day 28 (HR = 1.87, IC 95% [1.36–2.56], p = 0.0001), and day 90 (HR = 1.72, IC 95% [1.33–2.22], p < 0.0001), independently of the cause of death. Severe hyperglycemia was associated with increased mortality risk at days 28, when patients were admitted for shock, except septic shock, (HR = 1.70, IC 95% [1.01–2.87], p = 0.04) but not when admitted for coma, sepsis or cardiac arrest. Mortality rate was significantly higher in patients with severe hyperglycemia compared to those without, regardless of preexisting diabetes (HND HD vs. NHND NHD groups + p < 0.05). Patients with severe hyperglycemia had a higher incidence of type 2 diabetes at 6 (16 vs. 8% + p = 0.01) and 12 months (14 vs. 9% + p = 0.08) compared to those who did not.

**Conclusion:** Severe hyperglycemia occurring in the first 3 days of ICU admission was associated with higher mortality rate and an increased risk of diabetes in the following months regardless of preexisting diabetes.

### CO-100 Relation between level of 25OH-vitamine D at ICU patient admission and outcomes

#### Khaldi Amina^1^, Prevedello Danielle^1^, Mickael Gardette^1^, Autphenne Myriam^1^, Preiser Jean Charles^1^

##### ^1^Hopital ERASME, Bruxelles, Belgium

###### **Correspondence:** Khaldi Amina - amina.khaldi@erasme.ulb.ac.be

*Annals of Intensive Care* 2018, **8(Suppl 1):**CO-100

**Introduction:** Vitamin D deficiency is frequent in Northwestern countries and could represent a modifiable risk factor for critically ill patients, in relation with its pleiotropic effects (1). Some studies reported an association between 25OH vitamin D (25OH) deficiency, chronic health status and ICU- and hospital-related outcomes. However, a large supplementation study have not been found to improve outcome of patients with moderate 25OH deficiency (< 20 ng ml) (2). The aim of the study is to analyze the relationship between the severity of 25OH deficiency at ICU admission, severity of illness and outcomes and ultimately to identify subgroups of patients in whom the likelihood of benefit of supplementation is larger.

**Patients and methods:** Consecutive patients admitted over a 4-month period who stayed at least 48 h in a medical surgical 32-bed ICU were included. In these patients, demographic data, Charlson co-morbidity score, severity scores (SAPS 3 and SOFA) and 25-OH (chemiluminescence, DiaSorin) were collected at admission. ICU and hospital length of stay (LOS) and mortality were recorded. Correlations were searched between 25OH and the different scores, and vital outcomes by non-parametric tests for continuous value and by cross dynamic board for categorical variables.

**Results:** A total of 185 patients have been analyzed. The mean (SD) age was 66 (15) years, and 50% were men, most often admitted for a medical reason (57.8%), Charlson score 2.5 ± 2.9, SAPS III 56.2 ± 20.5, SOFA 5.5 ± 4.5, ICU (LOS) 8.2 ± 9.2 days, Hosp LOS 28.4 ± 27.5 days, ICU mortality 17.8%, hospital mortality 23.8%. The mean 25OH at admission was 16.9 ± 11.5 ng ml. 25OH deficiency (> 20 ng ml) in 69.7%, severe deficiency (> 12 ng ml) in 47.5%, and a moderate deficiency (12–20 ng ml) in 22.2%. However, there was no correlation between vitamin D level at ICU admission, severity of illness, mortality and LOS (Fig. 1).

**Fig. 1:**
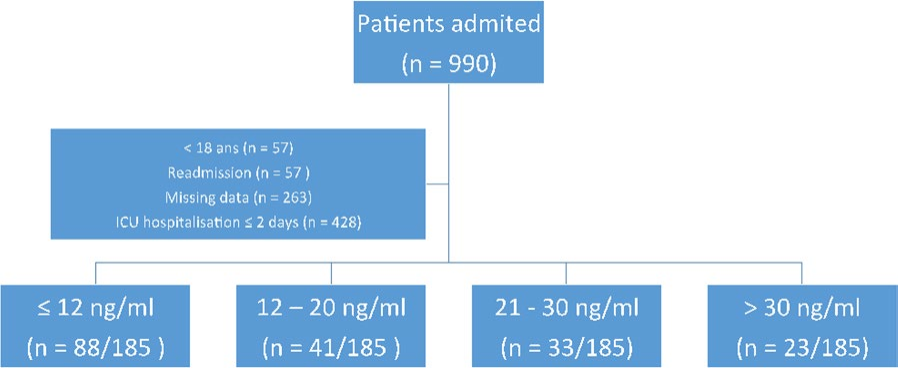
Flow chart of the study

**Conclusion:** These findings confirm the high incidence of severe 25OH deficiency and a possible relation with a higher mortality in the subset of long-stayers. It reflects a marker of poorer health. Specific subsets of patients could benefit from vitamin D supplementation.

### CO-101 Diagnosis, management and outcome of severe hypothyroidism in ICU: an observational multicenter study

#### Bourcier Simon^1^, Coutrot Maxime^1^, Van Grunderbeeck Nicolas^2^, Azoulay Elie^3^, Hraiech Sami^4^, Aissaoui Nadia^5^, Delmas Clément^6^, Ferré Alexis^7^, Messika Jonathan^8^, Terzi Nicolas^9^, Nseir Saad^10^, Carreira Serge^11^, Persichini Romain^12^, De Montmollin Etienne^13^, Reuter Danielle^14^, Contou Damien^15^, Lautrette Alexandre^16^, Bigé Naïke^17^, Lascarrou Jean-Baptiste^18^, Sztrymf Benjamin^19^, Combes Alain^1^, Schmidt Matthieu^1^

##### ^1^Hôpital Pitié-Salpêtrière, Paris, France; ^2^CH de Lens, Lens, France; ^3^CHU Saint-Louis, Paris, France; ^4^CHU de Marseille - Hôpital Nord, Marseille, France; ^5^Hôpital Européen Georges Pompidou, Paris, France; ^6^CHU Toulouse - Hôpital Rangueil, Toulouse, France; ^7^CH de Versailles, Le Chesnay, France; ^8^Hôpital Louis Mourier, Colombes, France; ^9^CHU de Grenoble - Hôpital A. Michallon, Grenoble, France; ^10^CHRU de Lille - Hôpital Roger Salengro, Paris, France; ^11^Hôpital Saint-Camille, Bry -Sur-Marne, France; ^12^CHU La Réunion - Hôpital Felix Guyon, Saint-Denis, France; ^13^CH de Saint-Denis - Hôpital Delafontaine, Saint-Denis, France; ^14^CH Sud Francilien, Corbeil-Essonnes, France; ^15^CH d’Argenteuil, Argenteuil, France; ^16^CHU Gabriel Montpied, Clermont-Ferrand, France; ^17^CHU Saint-Antoine, Paris, France; ^18^CHU de Nantes, Nantes, France; ^19^CHU Antoine-Béclère, Clamart, France

###### **Correspondence:** Bourcier Simon - simon_bourcier@hotmail.com

*Annals of Intensive Care* 2018, **8(Suppl 1):**CO-101

**Introduction:** Severe hypothyroidism manifestations including myxedema coma and tamponnade frequently lead to intensive care unit (ICU) admission. To date, data on this severe disease in ICU are scarce and limited to case reports. We aimed to describe clinical presentation, management and outcomes of severe hypothyroidism admitted in ICU.

**Patients and methods:** Local diagnoses coding database (from January 2000 to July 2017) from 15 French ICU were interrogated for main and secondary related to hypothyroidism and myxedema coma in the International Classification of Disease 10th revision. Two investigators therefore reviewed all medical records selected. Inclusion criteria were severe patients whom severe clinical hypothyroidism manifestations were the main reason for ICU admission. Clinical presentation, therapy used, and outcome were recorded.

**Results:** We report the preliminary data of 40 patients (80% female + median age 70 years (65–76) + SAPSII 58 (41–74)). Hypothyroidism was unknown before ICU admission in 68% patients. Median SOFA score at ICU admission was 6 (4–10). Myxedema coma, circulatory failure, respiratory failure, and severe hypothermia were respectively the main admission reason in 60, 18, 10, and 5% patients. A precipitating factor such as drugs thyroid toxicity, thyroid hormone withdrawal or infection was found out in only 13 (32%) patients. Main causes of hypothyroidism were thyroiditis and thyroidectomy. Thirty-two (80%) patients had alteration of consciousness with a median Glasgow score at 10 (6–13). In addition, median heart rate at ICU admission was 50 (35–65) bpm while hypothermia < 35 °C was noted in 24 (60%) patients. Median TSH level at admission was 78 (43–135) mUI l, T3 and T4 levels respectively 0 (0–0.45) pmol l and 2 (0–5.07) pmol l. Rhabdomyolysis was frequent with median CPK level 539 (192–1500) UI l. Organ support including mechanical ventilation, catecholamine infusion and, renal replacement therapy were respectively used in 58, 45, and 20% patients. Lastly, 70% patients received oral levothyroxine whereas the intravenous form was used in others. Overall ICU-mortality was 22%.

**Conclusion:** Our preliminary data showed that severe manifestations of hypothyroidism leading to ICU admission represent de novo hypothyroidism in two-thirds of patients, leading to a high mortality.

### CO-102 Central nervous system infections management in the ED: a race against time!

#### Le Borgne Pierrick^1^, Perriguey Arnaud^1^, Dascalu Elena^1^, Ugé Sarah^1^, Kauffmann Philippe^1^, Kam Claire^1^, Quoirin Etienne^1^, Bilbault Pascal^1^

##### ^1^CHU Strasbourg, Strasbourg, France

###### **Correspondence:** Le Borgne Pierrick - pierrick_med@yahoo.fr

*Annals of Intensive Care* 2018, **8(Suppl 1):**CO-102

**Introduction:** When it comes to infections of the central nervous system (CNS), the greatest challenge in the Emergency Department (ED) is to identify patients that have a rare life-threatening diagnosis. Alone or in combination, fever, headache, altered mental status encompass a broad differential diagnosis. Antibiotics or antiviral therapy should be given as soon as possible, ideally after both blood and cerebrospinal fluid (CSF) have been obtained. Early treatment is associated with a lower mortality.

**Patients and methods:** We present here, a four-year (2012–2015) retrospective and monocentric study. During the period of the study, we included all adult patients with the diagnosis of CNS infection (positive CSF culture). We collected and analyzed all clinical, biological, imaging, treatments and evolution datas during the stay. A total of 67 patients with CNS infection have been included for statistical analysis. We analyzed a second group (n = 25) with suspected CNS infection (negative CSF) as a control group.

**Results:** In the study population, mean age was 43 ± 21.5 years old and the sex-ratio was 1.2. There were no difference between the two groups in terms of clinical signs except for more altered mental status in the control group (p = 0.02). All patients of the study (n = 92) benefited of lumbar puncture (LP) in the ED with an average time of 381 ± 370 min after admission. This delay was the same between the two groups (p = 0.76) but was significantly higher in the encephalitis subgroup (n = 13, p = 0.03). Patients who had imaging (CT or MRI) during the ED stay had more likely a delay in LP realization (450 vs 193 min, p = 0.0005). Patients where the CNS infection diagnosis was firstly evoke by the triage nurse had LP more quickly (p = 0.03). The median door to-antibiotic-time was 339 min (IQR 198–534) with no difference between the two groups of the study (p = 0.7). 94% of all patients were hospitalized for an average length of stay of 11.7 ± 15.5 days and 10% of them were admitted in the ICU. The in-hospital mortality was 14% in the study population.

**Conclusion:** Our study highlights the poor adherence to guidelines. The management of CNS infections remains slow and heterogeneous. Management practices, such as the timing of LP realization or antibiotics in relation to the return of CT scan or CSF results are the most important factors associated with antibiotic timeliness.

### CO-103 Viral acute exacerbations of chronic obstructive pulmonary disease (COPD)

#### Elabbadi Alexandre^1^, Gounane Chérifa^1^, Gibelin Aude^1^, Taconet Clémentine^1^, Voiriot Guillaume^1^, Fartoukh Muriel^1^

##### ^1^Hôpital Tenon, Paris, France

###### **Correspondence:** Elabbadi Alexandre - alexandre.elabbadi@gmail.com

*Annals of Intensive Care* 2018, **8(Suppl 1):**CO-103

**Introduction:** There are numerous causes of acute exacerbations of COPD (AECOPD), the most common of which are bronchial and or pulmonary infections. Viral etiologies may account for 30% of AECOPD, but this rate is likely underestimated because of the limited performance of the conventional diagnostic tests. Multiplex molecular diagnostic tests may identify several pathogens including viruses and bacteria, from a single respiratory tract sample, with high sensitivity. Using these tests, respiratory viruses are identified in 30 to 60% of cases, according to the series. The objective of this work was to describe the microbial epidemiology, the management and the outcome of patients admitted to the intensive care unit (ICU) with moderate to severe AECOPD, in the era of multiplex testing.

**Patients and methods:** A prospective non interventional multicenter study conducted in two university-teaching hospitals. In addition to the usual samplings, a nasopharyngeal swab was performed for multiplex polymerase chain reaction (PCR), using respiratory panels FilmArray Biomérieux (17 viruses and 3 bacteria) or ePlex automaton (22 viruses and 4 bacteria) depending on the center.

**Results:** The preliminary results involve the 37 patients (29 males + 70 years (55–87)) included in Tenon hospital over a 6-month period. The mean FEV1 was 37% (21–60) median 35% [31–44]. Drug therapies included anticholinergics (n = 21 + 62%) and beta-2-mimetics (n = 24 + 71%), inhaled (n = 16 + 47%) or oral (n = 2 + 6%) steroids, and azithromycin (n = 5 + 23%). A respiratory virus was identified in 11 patients (30%), alone or in combination with a bacterium (n = 6). A bacterial pathogen was identified alone 9 times (21%). Therapeutic interventions did not differ depending on whether a virus was detected or not-exposure to antibiotics (10 ± 1.8 vs. 12.5 ± 2 d + p = 0.4), administration of oseltamivir (5/6 vs. 8/18 + p = 0.39), steroids (1/7 vs. 3/15 + p = 0.12) and mechanical ventilation (11/11 vs. 20/26 + p = .08). The ICU length of stay (5.8 ± 0.8 vs. 7.8 ± 1.1 d + p = 0.29) was similar. The ICU and 90d-mortality rates were 5.4 and 13.5%, respectively.

**Conclusion:** Respiratory viruses are frequently involved in moderate to severe AECOPD. The respiratory multiplex PCR should be performed in this setting and the results should be taken into account to more adequately use the anti-microbial treatments.

### CO-104 Community-acquired co-infection in severe imported falciparum malaria in adults

#### Roujansky Ariane^1^, Missaoui Lamia^1^, Wolff Michel^2^, Matheron Sophie^2^, Roy Carine^2^, Biard Lucie^3^, Bruneel Fabrice^3^

##### ^1^Paris, France; ^2^CHU Bichat, Paris, France; ^3^CHU de Versailles, France

###### **Correspondence:** Roujansky Ariane - ariane.roujansky@gmail.com

*Annals of Intensive Care* 2018, **8(Suppl 1):**CO-104

**Introduction:** Co-infections during severe malaria have been widely studied among children in the endemic setting and there is evidence they result in worse outcome. Little is known about the epidemiology and impact of these co-infections (community-acquired or nosocomial) in the course of imported malaria in adults. We sought to describe community-acquired co-infections in severe imported malaria (SIM) in adults and to identify associated factors.

**Patients and methods:** We conducted a multicenter observational study in 52 French intensive care units (ICU), derived from a retrospective (2000–2006) and a prospective (2007–2010) study. We identified patients admitted for severe imported falciparum malaria according to 2000 World Health Organization (WHO) criteria adapted for imported malaria. Community-acquired co-infection was defined as an infection diagnosed within the first 48 h following ICU admission. Clinical and demographic data were collected from medical charts using standardized case-report forms. Factors associated with community acquired co-infection were identified by univariate and multivariate analysis.

**Results:** Between 2000 and 2010, 555 patients were admitted for SIM. Mean age was 44 years, and males represented 69% of our population. All patients were treated by intravenous quinine. Among them 119 patients (21%) presented with at least one episode of co-infection, including 42 (35%) community-acquired episodes and 77 (65%) of nosocomial origin. Overall in-hospital mortality was 9% with no significant difference between patients with or without co-infections at admission. Among community-acquired co-infections (n = 42), pulmonary infection (43%), bacteraemia (31%), and urinary tract infection (12%) were the most common + *Escherichia coli*, *Streptococcus pneumoniae*, *Pseudomonas aeruginosa* and non-pneumoniae streptococci were predominant (16, 12, 10 and 10% of bacterial isolates respectively). By multivariate analysis, 3 variables at ICU admission were independently associated with community-acquired co-infection—male sex, respiratory distress (WHO modified criteria—requirement for noninvasive and or endotracheal mechanical ventilation, or spontaneous breathing with PaO2 < 60 mmHg (if FiO2 0.21), and or respiratory rate > 32 min), and acidosis (WHO criteria—pH < 7.35 or serum bicarbonate < 15 mmol L) (Table 1).

**Table 1:** Multivariate analysis of factors associated with community-acquired co-infection

Variables	OR [95% CI]	p
Gender: M/F (reference = F)	3.41 [1.75–6.78]	< 0.001
Respiratory distress (WHO modified criteria)	2.96 [1.42–6.08]	0.003
Acidosis (WHO criteria)	2.33 [1.04–5.07]	0.034

**Conclusion:** Our study shows that in a large population of adults admitted to ICU for SIM, 7.5% have a community-acquired co-infection, mainly bacteraemias and pulmonary infections. These results emphasize the need to search for co-infection in these patients and make therapeutic adaptations accordingly. Male sex, respiratory distress and acidosis at admission may help to identify patients most at risk.

### CO-105 Severe leptospirosis in metropolitan France, a retrospective multicentre study

#### Miailhe Arnaud-Félix^1^, Le Thuaut Aurélie^1^, Lascarrou Jean-Baptiste^1^, Bourhy Pascale^2^, Grall Maximilien^3^, Henry-Lagarrigue Mathieu^4^, Mercier Emmanuelle^5^, Maamar Adel^7^, Sedillot Nicolas^8^, Goursaud Suzanne^8^, Reignier Jean^1^

##### ^1^CHU de Nantes, France; ^2^Institut Pasteur, Paris, France; ^3^CHU de Rouen, France; ^4^CHU de La Roche-Sur-Yon, France; ^5^CHU de Tours, France; ^6^CHU de Rennes, France; ^7^CHU de Bourg En Bresse, France; ^8^CHU de Caen, France

###### **Correspondence:** Miailhe Arnaud-Félix - flexmiailhe@hotmail.com

*Annals of Intensive Care* 2018, **8(Suppl 1):**CO-105

**Introduction:** Leptospirosis is a worldwide zoonosis, and is more common in tropical area. Epidemiological data reported increased incidence during the past 10 years in Metropolitan France. Most of the cases are asymptomatic or sparsely symptomatic, but some patients develop severe leptospirosis, requiring hospitalisation in intensive care units (ICU). Only few studies exist on severe leptospirosis and data on severe leptospirosis in Europe are very scarce. We performed a retrospective multicentre study in metropolitan France in order to identify the characteristics, treatments and prognostic factors of severe leptospirosis in this context.

**Patients and methods:** LeptoRea was a retrospective multicentre study performed in 73 French ICUs. Each unit was requested to include all the patients admitted in ICU, between 2012 and September 2016, over than 18 years old, and with leptospirosis diagnosed with at least one positive test (PCR, MAT, ELISA). For each patient, a case report form was completed and hospitals report was sent to the study coordinator (AFM). A descriptive analysis was performed to assess the patients characteristics, treatments received and mortality rate. A univariate analysis was performed to identify factors associated with mortality.

**Results:** Of the 73 participating ICUs, 26 (35.6%) did not admit any patient with leptospirosis during the study period. 147 patients were included. 90% were male. Median age (years) and IGS2 [95% CI] were 54 [38 + 65] and 39 [28 + 56], respectively. Median delay between the onset of symptoms and ICU admission was 5 [4 + 6] days. Risk factors for leptospirosis and pre-existing comorbidities are detailed in the Table 1. Median SOFA score on day 1 [95% CI] was 10 [8 + 4]. 52 (35%) patients had ARDS and 23 (16%) had intra-alveolar haemorrhage. During their ICU stay, 85 patients (58%) were treated with vasoactive support, 52 (35%) with mechanical ventilation and 50 (34%) patients received renal replacement therapy. Median ICU and hospital length of stay were 4 [2 + 10] and 11 [7 + 17] days. ICU mortality was 8.9%.

**Table 1:** Pre-existing comorbidities

*Preexisting comorbidities n (%)*	
Tobacco	45 (31.3)
Alcohol	26 (17.8)
Cancer	2 (1.4)
Diabete mellitus	7 (4.8)
Liver disease	5 (3.4)
Cardiac disease	0
Pulmonary disease	0
Chronic renal failure	1 (0.7)
*Risk factors n (%)*	
None	8 (6)
Contact with rats	23 (17.3)
Contact with other rodents	17 (12.8)
Contact with dogs	20 (15)
Contact with water	91 (67.9)
Job related	18 (12.7)
Previous travel	17 (12.3)

**Conclusion:** This study is the first large multicenter study on severe leptospirosis in Metropolitan France and the world’s largest series on leptospirosis in ICU. Our data show that severe leptospirosis is uncommon in ICUs of Metropolitan France. Despite high IGS2 and SOFA score, mortality rate was lower than expected and previously reported.

### CO-106 Functional outcomes in adult patients with herpes simplex encephalitis admitted to the ICU: the HERPETICS multicenter study

#### Jaquet Pierre^1^, De Montmollin Etienne^2^, Dupuis Claire^1^, Sazio Charline^3^, Conrad Marie^4^, Susset Vincent^5^, Demeret Sophie^6^, Argaud Laurent^7^, Barbier François^8^, Sarton Benjamine^9^, Chabanne Russel^10^, Daubin Delphine^11^, Brulé Noelle^12^, El Kalioubi Ahmed^13^, Alves Mikael^14^, Tadie Jean-Marc^15^, Bouadma Lila^1^, Timsit Jean-François^1^, Sonneville Romain^1^, Encephalitica Study Group^16^

##### ^1^Hopital Bichat, Paris, France; ^2^Hopital Delafontaine, Paris, France; ^3^CHU de Bordeaux, France; ^4^CHU de Nancy, France; ^5^CHU de Chambery, France; ^6^Hôpital Pitié-Salpêtrière, Paris, France; ^7^Hôpital Edouard Herriot, Lyon, France; ^8^CHU d’Orléans, France; ^9^CHU de Toulouse, France; ^10^CHU de Clermond-Ferrand, France; ^11^CHU de Montpellier, France; ^12^CHU de Nantes, France; ^13^CHU de Lille, France; ^14^CHU de Poissy, France; ^15^CHU de Rennes, France; ^16^Paris, France

###### **Correspondence:** Jaquet Pierre - pierre.jaquet@yahoo.fr

*Annals of Intensive Care* 2018, **8(Suppl 1):**CO-106

**Introduction:** Herpes simplex encephalitis (HSE) remains the most common infectious cause of encephalitis in Europe and is associated with a poor prognosis. Little is known about management and functional outcomes of severe HSE cases requiring ICU admission. This study aimed to identify factors associated with poor neurological outcome in HSE patients admitted to the ICU.

**Patients and methods:** We conducted a large retrospective multicenter study on consecutive patients diagnosed with HSE in 46 ICUs in France, between January 2006 and December 2016. Patients were included if they met the International Encephalitis criteria for confirmed HSE, i.e. an acute encephalitis syndrome and a positive cerebrospinal fluid PCR for Herpes simplex virus. Multivariate logistic regression analysis was used to identify factors associated with poor outcome at 90 days, defined by a score of 3–6 (indicating severe disability or death) on the modified Rankin Scale (mRS). Data are presented as number (percentage) or median [interquartile range].

**Results:** Overall, 227 patients (age 64 [55–73] years) with confirmed HSE (HSV-1 infection—181 (95.8%) patients) treated with intravenous acyclovir were included. SAPS2 score was 43 [32–56] and 43 (18.9%) were immunocompromised. At ICU admission, the score on the GCS was 9 [6–12], body temperature was 38.7 [38–39.2] °C, seizures and status epilepticus were present in 78 (34.4%) and 22 (9.7%) patients, respectively. Invasive mechanical ventilation was required in 148 (65.2%) patients, and 33 (14.6%) received norepinephrine. Acyclovir was initiated 0 days [-1–0] after ICU admission at a dose of 10 [10–11.9]mg/kg/8 h for 21 [20–21] days. ICU and hospital mortality were 14.5% (n = 32) and 18.6% (n = 40), respectively. At 90 days, 164 (72.2%) patients had a poor outcome (including 43 (18.9%) deaths). Multivariate logistic regression analysis identified older age, immunocompromised status, body temperature > 38.5 °C, invasive mechanical ventilation at admission and MRI brain lesions > 3 lobes to be independently associated with poor outcome. By contrast, direct admission to the ICU (versus initial ward admission) and early brain MRI had a protective effect (Table 1). Status epilepticus, hemodynamic, renal, coagulation or hepatic failures had no impact on outcome.

**Table 1:** Multivariate analysis of factors associated with a poor outcome

Characteristic	Odds Ratio [95% CI]	p value
Age, per 1-year increment	1.03 [1.01–1.05]	0.007
Chronic alcoholism	3.30 [0.99–10.95]	0.051
Immunocompromised status	2.67 [1–7.1]	0.049
Score on the GCS		
< 8	0.59 [0.16–2.19]	0.483
8–12	0.68 [0.22–2.1]	0.768
14–15	1	0.727
Body temperature > 38.5 °C	2.76 [1.34–5.65]	0.006
Mechanical ventilation	3.22 [1.36–7.64]	0.008
Focal deficit	2.12 [0.87–5.13]	0.097
MRI brain lesions > 3 lobes	4.60 [1.69–12.57]	0.003
Early brain mri	0.28 [0.13–0.61]	0.001
Direct admission to icu	0.33 [0.16–0.7]	0.004

**Conclusion:** In adults with HSE requiring ICU admission, only 28% patients had functional independence at 90 days (mRS 0–2). Immediate ICU admission, early brain MRI and elevated body temperature represent modifiable factors that may impact patients’ outcome.

## Flash Communications

### F-01 Prophylactic non invasive ventilation after extubation in severe brain injured patients

#### Zarrouki Youssef^1^, Ait Souabni Sara^2^, Ziadi Amra^2^, Samkaoui M Abdenasser^2^

##### ^1^CHU Mohammed VI, Marrakech, Morocco

###### **Correspondence:** Zarrouki Youssef - zarroukiyoussef@hotmail.fr

*Annals of Intensive Care* 2018, **8(Suppl 1):**F-01

**Introduction:** Prophylactic non-invasive ventilation (NIV) is a well established method for prevention of post-extubation acute respiratory failure in hypercapnic patients. However, its role in the post-extubation period, in traumatic brain injury patients, is uncertain. Especially, because of effects of the brain injury, on respiration and airway control. We perform a study to assess the impact of prophylactic NIV after extubation among patients with severe traumatic brain injury.

**Patients and methods:** Over a period of 1 year, adult patients with isolated severe traumatic brain injury, who were under invasive mechanical ventilation for more than 48 h were eligible for inclusion in the study. They were randomized, after decision of extubation, to receive conventional therapy or conventional therapy associated with NIV. Conventional therapy consisted of oxygen delivery by facial mask, semi-recumbent position, mucus suctioning and nebulization therapy. The main objective of the study is to assess the impact on reintubation rate. Extubation succes was defined by the absence of need for reintubation within the 7 days. The secondary objective is to evaluate the effect on ICU length of stay after extubation.

**Results:** Fifty-three patients ranging in age from 17 to 61 years, were included in the study. They have no history of respiratory disease. 26 patients to receive NIV and 27 to receive conventional therapy. NIV was delivered with mean levels of inspiratory positive-airway pressure of 11.1 ± 1.8 cm H_2_O and expiratory positive-airway pressure of 5 ± 1.00 cm H_2_O for a mean period of 19.85 ± 10 h for 6 to 11 h day. Reintubation rates among NIV group (34.61%) and conventional group (44.44%) were similar (p = 0.39). The duration of intensive care unit stay after extubation (8.5 ± 2.55 vs 9.35 ± 1.72 days, p = 0.83) in both groups were comparable.

**Conclusion:** NIV is safe to use in severe brain injured patients soon after extubation, although clear benefit is not documented in this study. In order to establish a potential benefit of NIV in this type of patients, it is probably necessary to study it in the subgroup of hypercapnic patients in the post-extubation period.

### F-02 Non-invasive ventilation in acute hypoxemic respiratory failure

#### Cayrol Elsa^1^, Guesdon Charlotte^2^, Vargas Frédéric^1^, Georget Aurore^3^, Bui Hoang-Nam^1^, Sazio Charline^1^, Gruson Didier^1^, Boyer Alexandre^1^, Clouzeau Benjamin^1^, Pillet Odile^1^, Hilbert Gilles^1^

##### ^1^CHU Bordeaux, Bordeaux, France; ^2^CH Pau, Pau, France; ^3^Université de Bordeaux (ISPED), Bordeaux, France

###### **Correspondence:** Cayrol Elsa - elsa.cayrol@free.fr

*Annals of Intensive Care* 2018, **8(Suppl 1):**F-02

**Introduction:** The clinical benefit of non-invasive ventilation (NIV) in patients with acute hypoxemic respiratory failure (ARF) is being called into question. Indeed, in a multicenter randomized trial recently conducted in hypoxemic ARF patients (Pa02 Fi02 < 300), intubation rate in the NIV group was 50% and intensive care unit (ICU) mortality rate was 25%, numbers higher than in the standard-oxygen group (1). An excessive tidal volume under NIV is a hypothesis to explain these bad outcomes (2). Our experience does not seem to support these data. Therefore we wanted to—investigate the rate of NIV success in hypoxemic ARF and global in-ICU mortality. Estimate the average expired tidal volume and identify predictive factors of NIV failure.

**Patients and methods:** Observational cohort study conducted in a medical ICU, including all patients treated with NIV for hypoxemic ARF over a 3 years period.

**Results:** During the study period, 57 patients were included (mean age 57.9 ± 15.1 years, median SAPS II 43 (29–54), median Pa02 Fi02 151 (111–186)) and 23 had acute respiratory distress syndrome (ARDS). Intubation rate was 28.1% and in-ICU mortality was 14%. Patients with a Pa02 Fi02 ratio < 150 during the first NIV session had a mortality rate five times higher than those with a Pa02 Fi02 ≥ 150 (37.5 vs 7.3% p = 0.047). More than 2000 h of NIV were analysed. Median expired tidal volume was 10 ml/kg of predicted body weight (PBT) (8–11 ml/kg PBT), whether the patients were intubated (NIF failure) or not (NIV success). In multivariate analysis, only the presence of ARDS was independently associated with NIV failure.

**Conclusion:** With a success rate of 71.9% and a mortality rate of 14% it seems legitimate not to exclude VNI of the management of patients with hypoxemic ARF. Moreover, the hypothesis of a deleterious effect related to an excessive tidal volume has not been confirmed in our study.

### F-03 Non-invasive ventilation following unplanned extubation: Is it a suitable option?

#### Kudela Agathe^1^, Prat Dominique^1^, Jacobs Frédéric^1^, Millerux Maude^1^, Moneger Guy^1^, Dumenil Anne Sylyvie^1^, Demars Nadege^1^, Trouiller Pierre^1^, Sztrymf Benjamin^1^

##### ^1^Hôpital antoine Béclère, Clamart, France

###### **Correspondence:** Kudela Agathe - kudela.agathe@gmail.com

*Annals of Intensive Care* 2018, **8(Suppl 1):**F-03

**Introduction:** Unplanned extubation (UE) occurs in up to 33% of ICU patients. Reintubation is required in 28.5 to 74.7% because of respiratory failure, or other hemodynamic, neurologic or laryngeal matter. Very few data is available regarding the effect of non-invasive ventilation (NIV) performed after UE.

**Patients and methods:** Retrospective analysis of prospectively collected data on a 4 years period. A NIV local procedure, based on current guidelines, is available in the investigating centre, but UE is not mentioned. The decision to perform NIV in this setting was only based on the attending physician’s judgment. All patients experiencing an UE were included. We registered demographic data, patient’s characteristics and evolution after UE according to the occurrence of NIV. Prophylactic NIV denoted NIV performed after extubation without sign of respiratory failure, whereas curative NIV was a mean to treat post extubation respiratory failure.

**Results:** 106 UE occurred during the study period. Age was 59.4 [41.1–74.4] years, SAPS II 45 [36–54]. Reintubation was needed in 30 cases, within 1.8 [0.33–21.5] hours after UE, mostly for acute respiratory failure (n = 25). The death was associated with the need for reintubation (10 30 vs 0 76, p < 0.0001). NIV was performed in 21 cases. In all but 8 patients, NIV was used to treat an acute respiratory failure, the remaining indications being described as preventive of reintubation without evidence of respiratory failure. Respiratory failure as an indication for NIV was more often associated with reintubation (10/13 vs. 1/8, p = 0.008). NIV was also associated with death (5/21 vs. 5/85, p = 0.02). As a comparison, during the same period, 536 patients ventilated at least 48 h had a planned extubation (age 67 [54–80] years, SAPS II 49 [35–62]). Regarding NIV in this subgroup, data were available in 438 patients. NIV was used 141 times, with prophylactic NIV being more frequent (n = 118) as compared to rescue NIV (n = 23). Reintubation rates were similar as compared to UE patients for prophylactic NIV (16 118 vs. 1 8, p = 0.94) and rescue NIV (9 23 vs. 10 13, p = 0.26).

**Conclusion:** Though limited by its design, our study seems to show a similar efficacy of NIV following UE as compared to planned extubation, with a safety concern for rescue NIV and a potential interest for “prophylactic” NIV. Further data is warranted.

### F-04 Predictive factors of noninvasive ventilation failure during acute respiratory failure due to bronchiectasis exacerbation

#### Lahmar Manel^1^, Tilouche Nejla^1^, Mnif Karama^1^, Sikali Habiba^1^, Gharbi Rim^1^, Fekih Hassen Mohamed^1^, Elatrous Souheil^1^

##### ^1^EPS Taher Sfar, Mahdia, Tunisia

###### **Correspondence:** Lahmar Manel - firassmal4@gmail.com

*Annals of Intensive Care* 2018, **8(Suppl 1):**F-04

**Introduction:** Noninvasive ventilation (NIV) is the gold standard in the treatment of COPD exacerbation. NIV in acute respiratory failure (ARF) due to bronchiectasis exacerbation remain less recommended. Objectives—to evaluate the utility and factors predicting failure of NIV during bronchiectasis exacerbation.

**Patients and methods:** we conducted a retrospective study between 2001 and 2016 in the Medical intensive care unit in the hospital of Taher Sfar in Mahdia. All patients with bronchiectasis exacerbation and requiring NIV were enrolled. Only the first episode of exacerbation was recorded and analyzed. At admission we collected the following parameters—demographic details, etiology of exacerbation, comorbidities, the SAPSII score, arterial blood gases, respiratory, hemodynamic and neurologic parameters, NIV failure, nosocomial infection, duration of NIV, length of stay and mortality.

**Results:** 4900 patients were hospitalized in our medical intensive and 89 patients met the inclusion criteria. Bronchiectasis was associated with other pulmonary diseases in 16 patients. Bronchitis was the most common etiology of exacerbation (56%). three patients pseumononas aeruginosa in sputum cultures. NIV was successful in 71% of patients. The mortality was 19%. Length of stay was 12 ± 10 daysThe NIV failure group was older 67 ± 14 vs 59 ± 18, had a lower pH (7.25 ± 0.96 vs 7.31 ± 0.068) and elevated SPAS II score (33 ± 15 vs 24 ± 10). Factors associated with NIV failure were the SAPSII score (OR 2, IC 95% (1.5–3.02), p = 0.038) and the pH at admission (OR 6, IC 95% (2.04–8.06), p = 0.012). Nosocomial infection (54 vs 5%) and mortality (30 vs 2%) were significantly lower in NIV success group. However, Duration of NIV and length of stay were similar between the two groups.

**Conclusion:** NIV in bronchiectasis exacerbation reduce mortality and nosocomial infections. The rate of NIV failure was high. The SAPSII score and pH were the predictive factors of NIV failure.

### F-05 Evaluation of the risk of hyperoxia-induced hypercapnia in obese cardiac surgery patients: crossover comparison of two saturation targets and two oxygen titration modes

#### Denault Marie-Hélène^1^, Ruel Carolanne^1^, Simon Mathieu^1^, Bouchard Pierre-Alexandre^1^, Lellouche François^1^

##### ^1^Institut universitaire de cardiologie et de pneumologie, Québec, Canada

###### **Correspondence:** Denault Marie-Hélène - marie-helene.denault.1@ulaval.ca

*Annals of Intensive Care* 2018, **8(Suppl 1):**F-05

**Introduction:** Noxious effects of hyperoxia have been described for more than 60 years. Recent studies on stable obesity-hypoventilation syndrome (OHS) patients have risen concern about hyperoxia-induced hypercapnia in this population, but many questions remain unanswered. How important is this risk in acute care? Is there a risk in non-OHS obese patients as well? This study aims to evaluate the risk of hyperoxia-induced hypercapnia in post-op obese cardiac surgery patients.

**Patients and methods:** 30 obese patients (BMI > 30) having coronary artery bypass grafting (CABG) are currently being recruited. Informed written consent is obtained before inclusion. With a crossover design, we are comparing two oxygenation strategies immediately after extubation, in terms of their effect on arterial partial pressure of carbon dioxide (PaCO2)—a peripheral oxygen saturation (SpO2) target of > 95% achieved with manual titration (control period) and a SpO2 target of 90% achieved with automatic titration by a closed-loop system (FreeO2 period). Every patient is delivered oxygen according to both strategies for 30 min each, in a randomized order. FreeO2 (Oxynov, Québec, Canada) is a closed-loop oxygen delivery system which titrates oxygen flow according to the patient’s real-time SpO2 and a target set by the physician. SpO2, oxygen flow and heart rate are continuously recorded by FreeO2 during both periods. Hemodynamic parameters are checked at 10 min intervals and arterial blood gases are collected at the end of each period. The primary outcome is the change in PaCO2 between periods.

**Results:** 17 patients have been included so far and data for 16 patients are presented here. 13 are men, mean age is 60 ± 8 years and mean BMI is 34 ± 4 kg m^2^. 9 patients are responders, as shown by an elevation in PaCO2 in control period compared to FreeO2 period, and 7 patients are non-responders. Two distinct oxygenation profiles are being studied (see Fig. 1). The mean SpO2 for FreeO2 period is 91.1 ± 1.3% while it is 97.2 ± 1.4% for control period. Mean oxygen flow is 0.7 ± 0.7 L min for FreeO2 period (equivalent to < 24% FiO2) while mean FiO2 is 51 ± 10% for control period.

**Fig. 1:**
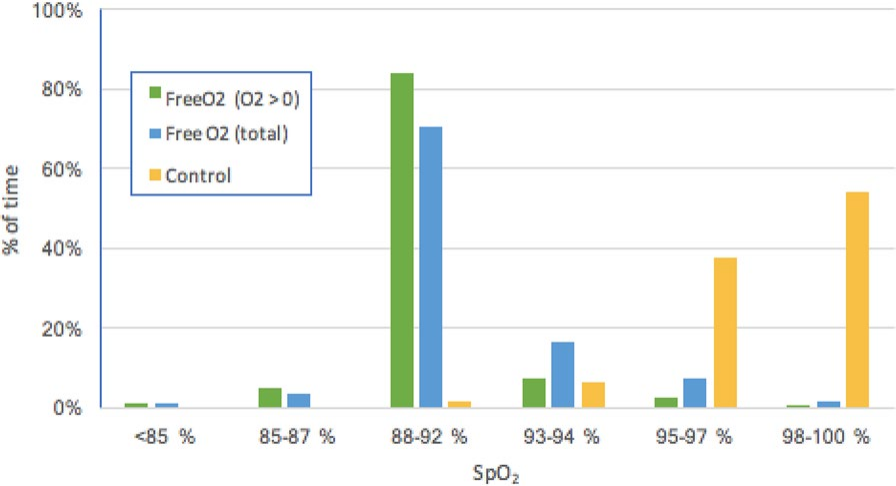
Percentage of time in different saturation ranges per period

**Conclusion:** FreeO2 is helpful in maintaining oxygenation within target. High SpO2 values may cause an elevation in PaCO2 in obese patients after CABG, but more analyses are required for this outcome. Characteristics of responders and non-responders will be analyzed after study completion.

### F-06 Automated patient-ventilator asynchrony monitoring in the ICU (CURVEX)

#### L’Her Erwan^1^, Hourmant Baptiste^2^, N’Guyen Quang-Thang^3^, Pateau Victoire^2^, Lellouche François^4^

##### ^1^LATIM INSERM UMR 1101, Brest, France; ^2^CHUY de la Cavale Blanche, Brest, France; ^3^Oxynov, Plouzané, France; ^4^Centre de Recherche de l’IUCPQ, Québec, Canada

###### **Correspondence:** L’Her Erwan - erwan.lher@chu-brest.fr

*Annals of Intensive Care* 2018, **8(Suppl 1):**F-06

**Introduction:** Optimal patient-ventilator synchronisation improves patients’ comfort and avoids unnecessary WOB. Asynchronisms are currently diagnosed by direct bedside ventilatory curves analysis, which is yet operator dependent and time-consuming, or by invasive methods including esophageal pressure or diaphragmatic electromyogram measurements. The main purpose of this study was to assess the relevance of Curvex as a noninvasive diagnostic and classification tool for asynchronism management.

**Patients and methods:** This project is based on a prospective physiological tracing data-warehousing program (Rea STOC, clinicaltrials.gov # NCT02893462) that aims to record 1500 consecutive ICU patients, over 3-years. All consecutive patients were recorded for a 2-hours period during 24-h following ICU admission. All measurements were recorded with the patient laying supine, with a 30° bed angulation. Raw ventilatory pressure and flow curves were transferred to a centralized server using a dedicated network. The physician in charge of the study was informed of the online analysis on a routine basis. Physiological recordings were associated with metadata collection. Asynchronisms detection is based on a non-parametric hypothesis testing (Random Distortion Testing), that requires no prior information on the signal distribution. Beside Asynchrony Index monitoring (AI), five asynchronism’s types were qualified—ineffective efforts (IE), short cycles (SC), multiple cycles (MC), prolonged inspiration (PI) and premature cycling (PC).

**Results:** 72 patients were included (age 61.2 ± 13.8 yr + 52 male 20 female + SAPS II 53.6 ± 2.4), of whom 15 with chronic respiratory diseases. Reason for admission was respiratory distress (47%), hemodynamic failure (29%), and neurological failure (22%). 92% patients where under invasive mechanical ventilation (12.8 ± 18.7 days) and 32% under sedation. Asynchronies were frequent (less 10% patients with AI < 10%) and mean AI was 34 ± 18%. IE were the most frequent asynchronisms (39.7 ± 28.6%). Concordance between visual and automatic evaluation was 97.2%. An attempt to reduce AI according to a standardized protocol was performed in 56/72 patients. Significant AI reduction (≥ 10%) was observed for only 10 patients (mean AI reduction = 15.5 ± 3.9%). PC were reduced for 28/56, SC for 27/56, PI for 25/56, MC for 25/56, and IE for 20/56 patients. No correlation was depicted between AI and patients status.

**Conclusion:** Asynchronies are frequent during MV and can be efficiently and noninvasively monitored using Curvex. The impact of asynchronism on patients’ prognosis and the availability of correction measures require further investigation.

### F-07 Early mechanical ventilation in patients with Guillain-Barré syndrome at high risk of respiratory failure: a randomized, controlled study

#### Melone Marie-Anne^1^, Mompoint Dominique^2^, Aboab Jérôme^2^, Meng Paris^2^, Clair Bernard^2^, Salomon Jérôme^2^, Sharshar Tarek^2^, Orlikowski David^2^, Annane Djillali^2^

##### ^1^Rouen, France; ^2^Hôpital Raymond Poincaré

###### **Correspondence:** Melone Marie-Anne - m_a.melone@icloud.com

*Annals of Intensive Care* 2018, **8(Suppl 1):**F-07

**Introduction:** About 30% of patients with Guillain-Barré syndrome (GBS) need mechanical ventilation (MV), of whom roughly 75% may develop pneumonia, in particular those with swallowing impairment. This trial aimed at assessing the benefit of early mechanical ventilation (EMV) in the prevention of onset of pneumonia in GBS patients.

**Patients and methods:** Design—this was a single centre, open-label, randomized controlled trial performed on two parallel groups. Patients—50 ICU adults admitted for GBS and risk factors for endotracheal intubation, i.e. time from onset to admission of < 7, inability to lift the head, and forced vital capacity of < 60%. Intervention—patients were randomized to receive EMV via face mask or endotracheal intubation owing to the presence or absence of impaired swallowing (experimental arm), or to receive conventional care that may include invasive mechanical ventilation whenever acute respiratory failure occurred (control arm). Outcomes—the primary outcome was the time to onset of pneumonia up to ICU discharge (or 90 days, pending of which occurred first). Secondary outcomes included the total number of episodes of pneumonia, time to and length of mechanical ventilation, length of hospital stay, mortality and any serious adverse events.

**Results:** Twenty-five patients were randomized in each group. In EMV, 44% required invasive mechanical ventilation. There was no evidence for clinically relevant differences in baseline characteristics between groups (Vital capacity—47% [40 + 54], PaCO2—5.3 kPa [4.6 + 5.6], PaO2—11.8 kPa [9.6 + 12.3]). 48% of patient in experimental group and 52% in control group had swallowing impairment. There was no significant difference between groups for the time to pneumonia (p = 0.50, Gray test). There were 16/25 (64%) and 15/25 (60%) of patients that presented at least one episode of pneumonia (p = 1.00), in the experimental and control groups, respectively. During follow-up, 16/25 (64%) patients in the control group were eventually mechanically ventilated (all invasively) and 25/25 (100%) in the experimental arm (p < 000.1). The time on ventilator was non-significantly shorter in the experimental arm (14 [7 +29] versus 21.5 [17.3 +35.5], p = 0.10). There were no significant differences between groups for length of hospital stay, neurological scores, the proportion of patients who needed tracheostomy, in-hospital death, or serious adverse events.

**Conclusion:** In Guillain-Barré syndrome patients at high risk of respiratory failure, early mechanical ventilation did not prevent the onset of pneumonia.

### F-08 Background and training of French Intensive Care fellows: a national survey

#### Messika Jonathan^1^, Helms Julie^2^, Vieillard-Baron Antoine^3^, Guidet Bertrand^4^, Ricard Jean-Damien^1^

##### ^1^Hôpital Louis Mourier, Colombes, France; ^2^CHU de Strasbourg, France; ^3^Hôpital Ambroise paré, Boulogne, France; ^4^Hôpital Saint Antoine, Paris, France

###### **Correspondence:** Messika Jonathan - jonathan.messika@aphp.fr

*Annals of Intensive Care* 2018, **8(Suppl 1):**F-08

**Introduction:** French hospital fellows’ missions are threefold—teaching, research and care. Although some diplomas are welcome (Master 2, for instance), prerequisites to become a fellow are limited to graduation in a specialized medical degree, and a medical thesis. Hence, fellows in the ICU may have a very varied background. We therefore decided to perform a survey of the scientific, clinical and educational training of intensive care medicine fellows, at the dawn of a new degree in intensive care medicine, and analyse heterogeneity causes.

**Patients and methods:** We conducted a national survey among the current and former fellows (2014–2017) of French medical and mixed critical care units, with an electronic questionnaire.

**Results:** One hundred and fifty-seven current and former fellows were contacted, among whom 123 (78%) responded (age—33 years [32–35], men 60.2%)—56.9% completed a complementary degree in intensive care, while 37% were on their way to graduate. Their background speciality was anaesthesiology (47.2%), nephrology (13%), cardiology and pneumology (12.2% each), internal medicine (11.4%), or another medical speciality (4%). One hundred and eighteen respondents (87.8%) had at least one university diploma. Seventy-one respondents had graduated with a Master 2 degree and 16 of them had a Ph.D. while 29 anticipated its completion. Medical education training was mainly provided by the Universities, when the fellows entered their position (n = 38 + 30.9%). The scientific production of the respondents was valorised by at least one presentation in a congress (83.7%) or by a scientific article published or to be published as first author (51.2%). There were heterogeneities—more Master 2 degree holders in Ile-de-France and older fellows were more trained in pedagogy.

**Discussion:** The increasing demand for fellow positions and the stability of their number calls for a clear definition of criteria for position attribution. Although a Master 2 degree is usually required to perform experimental research, no counterpart is necessary for clinical research.

**Conclusion:** This survey shows a diverse, but solid scientific and clinical training of French critical care fellows, attested by a high rate of Master degree and Ph.D. This inventory must be addressed in other medical specialities.

### F-09 Is micro-convex better than standard linear vascular probe for echo guided internal jugular and axillary venous puncture: a randomized controlled study on an inanimate manikin

#### Maury Eric^1^, Bonsey Michael^1^, Bouys Lucas^1^, Joffre Jéremie^1^, Dumas Guillaume^1^, Baudel Jean-Luc^1^, Ait Oufella Hafid^1^, Bigé Naïke^1^

##### ^1^Hôpital Saint-Antoine, Paris, France

###### **Correspondence:** Maury Eric - eric.maury@aphp.fr

*Annals of Intensive Care* 2018, **8(Suppl 1):**F-09

**Introduction:** International guidelines recommend ultrasound (US) guidance for central venous catheter (CVC) insertion. However, evidence is lacking for several aspects of guidance such as probe shape or whether the needle has to be in plane (IP) or out-of-plane (OOP). We assessed these issues in a randomized trial.

**Patients and methods:** 53 Health Care Workers [mean age—27 [22–31], male (28), senior (n = 18), resident (n = 13), student (n = 22), having previously inserted > 10 US guided CVC (n = 29)] were proposed to take part to a pilot study. Naive HCWs received a training (course and hands-on). Right Internal Jugular Vein (RIJV) and Right Axillary Vein (RAV) with either linear probe (LP) [HFL38, with the M-Turbo^®^, Sonosite, Bothewell, MA], or micro-convex probe (MCP) [GE8C, with a Vivid 7^®^, General Electric, 78457 Velizy, France] using IP and OOP approaches were performed on an inanimate manikin (Blue Phantom II, CAE Healthcare St. Louis, MO). Procedure was stopped at 180 s. The order of the 8 punctures—site probe approach—was randomized. Success at first pass, number of attempts (needle passes), success, times between skin contact and needle skin penetration and between needle skin penetration and liquid back flow in the syringe were recorded. Qualitative and quantitative values are expressed as number (percentage), and median (range), and were compared using the Wilcoxon matched pairs test and the Fisher exact test, respectively.

**Results:** For IJV puncture, first attempt success rate was more than 80% and was neither influenced by probe shape nor approach (Table 1). Conversely for RAV puncture, using LP with IP approach was more frequently successful at first attempt (45 vs 34%, p = 0.2). Time elapsed between needle skin penetration and liquid back flow was shorter for RAV puncture using IP approach (16 s vs 34 s, p = 0.03). Time elapsed between probe appliance on skin and liquid back flow was significantly shorter with the linear probe for IJV whatever the approach and for RAV using IP approach. RAV puncture was more frequently impossible with MCP (10 vs 3%, p = 0.08). Arterial puncture occurred more frequently with MCP (7 vs 3%, p = 0.08). LP use and IP approach were associated with more free event puncture (93 vs 87%, p = 0.03). Analysis according to previous experience disclosed similar trends.

**Table 1:** Characteristics of 424 venous punctures performed by 53 HCWs using US guidance

	Characteristics of 424 venous punctures performed by 53 HCWs using US guidance							
	Right Internal Jugular Vein				Right Axillary Vein			
	LP/IP	MCP/IP	LP/OOP	MCP/OOP	LP/IP	MCP/IP	LP/OOP	MCP/OOP
Success 1st pass (%)	85	89	83	83	45	34	43	47
Success 2nd pass (%)	11	9	8	8	38	28	28	21
Success 3rd pass (%)	4	2	8	8	9	25	19	19
Puncture/liquid back flow	3	5	4	4	16	34*	16	14
Skin contact/liquid backflow	21	30*	13	20*	48	74*	39	42
Failure (%)	0	0	0	0	6	14	0	8
Arterial puncture (%)	0	4	2	0	6	16	16	16
Puncture free event (%)	100	96	98	100	95	74	84	76*^$^

Puncture/liquid back flow: time (median) elapsed between skin needle penetration and liquid back flow in syringe (s)

Skin contact/liquid backflow: time (median) elapsed between probe appliance on the skin and liquid back flow in syringe (s)

Puncture free event: successful venous puncture without arterial puncture

*HCW* health care worker, *LP* linear probe, *MCP* micro-convex probe, *IP* in plane, *OOP* out of plane

*p < 0.05 between LP and MCP, ^$^p < 0.05 between IP and OOP

**Conclusion:** This study suggests no benefit of MCP over LP for CVC insertion and supports IP approach for axillary puncture.

### F-10 Validation of heart rate variability monitoring in high fidelity cardiac arrest simulation training in PRESAGE simulation center

#### Satre Buisson Lea^1^, De Jonckheere Julien^1^, Duburcq-Gury Emilie^1^, Jourdain Merce^1^

##### ^1^CHU de Lille, France

###### **Correspondence:** Satre Buisson Lea - leasatrebuisson@gmail.com

*Annals of Intensive Care* 2018, **8(Suppl 1):**F-10

**Introduction:** Simulation training is an effective teaching tool, preeminently in high-risk situations such as advanced cardiac life support (ACLS). Sudden cardiac arrest (CA) in clinical conditions or high fidelity simulation (HFS) represents a highly stressful medical situation. Stress measurement and its correlation to technical and non-technical performances could be a tool to improve teaching methods. Heart rate variability (HRV) is mediated by the autonomic nervous system and a lower HRV reflects parasympathetic inhibition therefore it could reflects stress. The aim of this study was to validate the feasibility of objective stress measurement by heart HRV monitoring during CA in HFS. Analyzing the relation between ANI, self-reported stress and performance was the second objective.

**Patients and methods:** An open monocentric prospective study was conducted from December 2015 to December 2016 in Lille’s medical simulation center PRESAGE. Intensive care residents from Lille Medical University played team leader in a CA scenario. Objective stress was measured from recordings of three leads electrocardiogram portable device (PhysioDoloris^®^ monitor, MDoloris Lille, France). HRV was obtained by analyzing time interval between successive R waves and transformed in the Analgesia Nociception Index (ANI). ANI gives both a qualitative and quantitative measurement of HRV. Self-reported stress was assessed with a visual analog scale (VAS). Results were expressed in numbers (%), mean (± Standard Derivation). Correlations were tested using the non-parametric Spearman rank test (Rho). p < 0.05 was considered significant.

**Results:** Sixty-four intensive care residents were monitored for HRV in simulation training. 36 signals (56.3%) were fully interpretable. Team leader’s self-reported pre-HFS stress VAS was 43.3 (± 22.4) and maximal stress VAS was 58.7 (± 21.7). Team leaders’ maximal heart rate was 157 bpm (± 20). Minimal ANI, reflecting intense stress was 33.7 (± 9.9). Objective and subjective stress of each team leader is shown in Fig. 1. There was a significant negative linear correlation between minimal ANI and maximal HR (Rho = − 0.52, p = 0.001). There was no significant correlation between self-reported stress VAS (neither pre HFS or maximal stress) and minimal ANI.

**Fig. 1:**
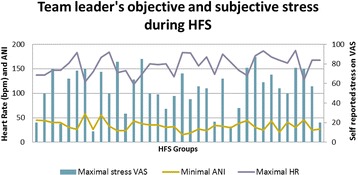
Team leader’s objective (heart rate and ANI) and subjective stress (declared VAS stress)

**Conclusion:** HRV monitoring is a feasible method to evaluate continuous physiological stress for team leaders in highly stressful simulation-teaching. Upgrading signal connection by Bluetooth 4.0 or Wi-fi could improve the method. Focusing on specific stressful time points might improve stress assessment and its correlation with performance.

### F-11 Simulation based-training: perception of ICU students and residents of the faculty of Monastir

#### Ouanes Islem^1^, Sik-Ali Habiba^1^, Ouanes-Besbes Lamia^1^, Fekih-Hassen Mohamed^1^, Hammouda Zaineb^1^, Dachraoui Fahmi^1^, Elatrous Souheil^1^, Abroug Fekri^1^

##### ^1^CHU F. Bourguiba, Monastir, Tunisie

###### **Correspondence:** Ouanes Islem - ouanes.islem@gmail.com

*Annals of Intensive Care* 2018, **8(Suppl 1):**F-11

**Introduction:** Simulation training has become available in Health sciences faculties and proposed in many specialties. Intensive Care is one of the fields of development of simulation based training. The aim of the present study was to report the experience of the Faculty of Medicine of Monastir Simulation Center in training medical students and residents in Intensive Care and to compare their respective perceptions.

**Patients and methods:** This was a descriptive study including students (5th year of the medical curriculum) and residents who received training during the last academic year (2016–2017), in the simulation center during their ICU traineeship. Simulation training was based on high-fidelity mannequins for students and seminars with high fidelity and procedural simulation training for residents. Three sessions per group were organized for students and a total of five sessions for residents. We collected participant characteristics and used Likert scale (from 1 to 5) to assess participant satisfaction, simulation fidelity, impact on clinical practice, stress level and instructor behaviors. Chi 2 test was used to compare students’ and residents’ perception of the simulation based-training.

**Results:** During the study period 91 students (of the 269 students’ whole promotion) and 88 residents actively participated at least in one of the simulation-based training sessions. Median students’ and residents’ ages were respectively 24 years (24–24) and 29 years (27–30). Participants were mainly females in 78 and 73.8% respectively in students and residents. Prior to the study period all the students received simulation-based training, whereas 48.9% of residents assisted for the first time in a simulation based training. For all participants, satisfaction (levels 4 and 5 of the Likert scale) was reported in 87.2%, simulation fidelity in 60.3%, possible impact on clinical practice in 77.1%, 40.8% perceived training as stressful and 85.5% noted favorably instructors’ behaviors. Compared to ICU residents, students were significantly less satisfied (80.2 vs 94.3% + p = 0.005), perceived training more stressful (59.3 vs 21.6% + p < 0.001) and noted less favorably instructors’ behaviors (79.1 vs 92%; p = 0.014), whereas the other items were comparable (Fig. 1).

**Fig. 1:**
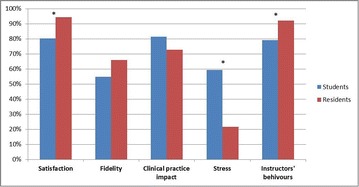
Comparison of perceptions between students and residents

**Conclusion:** We observed globally a favorable appreciation of ICU students and residents of the simulation based training differences in perceptions’ items could be mainly explained by different previous experiences with the real life in the hospital of the two study groups.

### F-12 Use of social media by Tunisian intensive care residents

#### Dachraoui Fahmi^1^, Nouira Wiem^1^, Tlili Mariem^1^, Hammouda Zaineb^1^, Bouker Nouha^1^, Hraiech Kmar^1^, Boukadida Sana^1^, Ouni Amal^1^, Zorgati Hend^1^, Ouanes Islem^1^, Ouanes Besbes Lamia^1^, Abroug Fekri^1^

##### ^1^CHU Fatouma Bourguiba, Monastir, Tunisia

###### **Correspondence:** Dachraoui Fahmi - dachraoui.fahmi@gmail.com

*Annals of Intensive Care* 2018, **8(Suppl 1):**F-12

**Introduction:** Social media (SM) can be used to improve or enhance professional networking and education, organizational promotion, patient care, patient education, and public health programs. Little data are available about perceptions among young medical practitioners although unconstrained online social activity is the norm for them. The aim of the present study is to describe the actual use and perception of the role of SM by Tunisian residents in intensive care medicine.

**Patients and methods:** This was a prospective observational study involving intensive care residents at the university hospitals of Sousse, Monastir and Mahdia (Tunisia). Residents were invited to participate and answer an anonymous questionnaire about social media in clinical medicine, accessibility to the internet, their personal and academic use of SM and their perception of the use of SM. The questionnaire comprised 22 different types of questions—Likert style, multiple choices, yes no, ranking and short and open response questions.

**Results:** Overall, 52 residents (36 females, age = 27.5 ± 3.9 years) responded to the questionnaire (66%) + 84.6 and 65.5% of them had smartphones and laptops, respectively. Sixty-five percent had mobile Internet access or a home WIFI network, while 13.5% said they had access to the Internet only through their faculty or hospital where they were exercising. Facebook and YouTube were the most frequently daily used SM for both personal and academic use, 94–32.7 and 61.6–34.6% respectively. In contrast, 94.2% said they had never used Twitter. The daily use of medical applications on Smartphones and weekly e-learning platforms were reported by 65.4 and 63.5% of residents respectively. Eighty-eight percent of the residents thought that YouTube should play a key role in their training. The use of SM was supposed to improve the collaboration between residents and with their supervisors respectively in 88.4 and 86.5%. Nearly half of the residents (42.3%) were opposed to communication with patients and or their families by SM. Respondents in the survey suggested that medical schools should use SM to keep them informed on training cycles, and that they should adopt a policy that would govern the use of SM in 94.2 and 88, 6% respectively.

**Conclusion:** Facebook and YouTube are the most used social media by Tunisian residents in intensive care medicine. They remain however, little contributors in their training course.

### F-13 Performance of the electronic referential ANTIBIOGARDE^®^ in initial antibiotic prescription in a French ICU

#### Allary Chloé^1^, Mentec Hervé^1^

##### ^1^Hôpital Victor Dupouy, Argenteuil, France

###### **Correspondence:** Allary Chloé - chloe.allary@gmail.com

*Annals of Intensive Care* 2018, **8(Suppl 1):**F-13

**Introduction:** Hospitals are encouraged to edit local antibiotic therapy guidelines. ANTIBIOGARDE^®^ is an electronic antibiotic prescription referential developed by a multidisciplinary team of French physicians, regularly updated, and locally customizable, which has been purchased by more than 170 French hospitals. We compared adequacy of initial antibiotic prescription by ICU clinicians, ANTIBIOGARDE^®^ proposal and national or international guidelines.

**Patients and methods:** Between January and June 2016, initial antibiotic prescriptions in an ICU were retrospectively analyzed when microbiologically documented. ANTIBIOGARDE^®^ and guidelines proposals were simulated, considering data available at the time of initial prescription. Adequacy was defined when all bacteria responsible for infection were sensitive to at least one prescribed proposed antibiotic. National guidelines were used when published after 2011. Otherwise, most recent international guidelines were used.

**Results:** 120 initial prescriptions were analyzed (45 monotherapy) in 97 patients (median age 63y, median SAPS II 53, median SOFA on prescription 7, ICU mortality 41%, 17% immunocompromised). Main sources of infection were lung (n = 75) and intra-abdominal (n = 23). Leading isolated bacteria were Enterobacteriaceae (n = 67, antibiotic resistance in 14), Streptococci (n = 55), non-fermenting Gram negative bacilli (n = 27, antibiotic resistance in 12) and Staphylococci (n = 25, resistance to methicillin in 5). In the 120 clinical settings analyzed, there was a proposal by ANTIOGARDE^®^ in 97 (81%) and a guideline available in 102 (85%) (p = 0.39). Initial antibiotic regimen prescribed by clinicians was adequate in 107 120 settings (89%, 95% CI [82–94%]). Considering only settings with a proposal, ANTIBIOGARDE^®^ was adequate in 90 97 (93%, 95% CI [86–96%]) (p = 0.36 vs clinicians), and guidelines in 102 102 (100%, 95% CI [96–100%] (p < 0.001 vs clinicians and p < 0.001 vs ANTIBIOGARDE^®^). Considering all settings and no proposal as inadequacy, ANTIBIOGARDE^®^ was adequate in 90 120 (75%, 95% CI [67–82%]) (p = 0.004 vs clinicians), and guidelines in 102 120 (85%, 95% CI [78–90%] (p = 0.34 vs clinicians and p = 0.05 vs ANTIBIOGARDE^®^). The antibiotic regimen prescribed by clinicians had a narrower spectrum against Gram negative bacilli than ANTIBIOGARDE^®^ and guidelines proposals in 70 and 68% of cases respectively. Proportions of prescriptions, ANTIBIOGARDE^®^ proposal and guidelines proposal including antibiotics active against methicillin resistant staphylococci were 12, 25 and 37% respectively (p < 0.00005).

**Conclusion:** ANTIBIOGARDE^®^ electronic referential could allow even non expert ICU physicians to adequately (93%) prescribe initial antibiotic therapy in most clinical settings (81%).

### F-14 Learning and performances of the face-to-face intubation technique in difficult intubation situation on a high-fidelity manikin—Macintosh laryngoscope versus Airtraq videolaryngoscope

#### Tabatchnik Xavier^1^, Descoins Médéric^1,^ Winer Arnaud^1^

##### ^1^Hôpital Gabriel Martin, Saint Paul, Reunion

###### **Correspondence:** Tabatchnik Xavier - xavier.tabatchnik@gmail.com

*Annals of Intensive Care* 2018, **8(Suppl 1):**F-14

**Introduction:** Intubation is plagued with a high morbimortality, especially in emergency situations. It is now acknowledged that a seated position allows for optimized preoxygenation (1). However, there is no guideline concerning the patient’s position for intubation. The patient is most often laid in a supine position, leading to a higher risk of aspiration (2). Face-to-face intubation in sitting position (FtFi) would allow for an easier intubation and a lower morbidity. We focused on learning the FtFi technique using the Macintosh laryngoscope and the Airtraq videolaryngoscope in simulated difficult intubation situation and comparing the performance of the FtFi with the classic technique.

**Patients and methods:** The participants would intubate a high-fidelity manikin (SimMan3G, Leardal, Norway) configured with a tongue edema (Cormack 2b-3). For each trial, time to intubate (TTI), success and complication rate, intubation difficulty and glottis exposure were noted. In classic position, three trials were performed with the Airtraq followed by the laryngoscope in order to obtain baseline parameters. In FtFi, at least 30 intubations were performed by each participant for each device. The utilization order was randomized.

**Results:** Thirty physicians, with an experience of at least 200 intubations each, were included. Figure shows the learning curves of the FtFi based on the evolution of the TTI measured for the Airtraq and the laryngoscope. In classic position, the mean TTI with the Airtraq was 30.5 ± 19.1 s versus 27.3 ± 16.7 s with the laryngoscope (p = ns). In FtFi, once the technique mastered, the TTI was 14 ± 2.9 s with the Airtraq versus 17.5 ± 2.4 s with the laryngoscope (p < 0.05). Success rate, TTI, complication rate, intubation difficulty and glottis exposure were better using FtFi versus classic intubation (p < 0.05). These parameters were even better with the Airtraq than with the laryngoscope (p < 0.05).
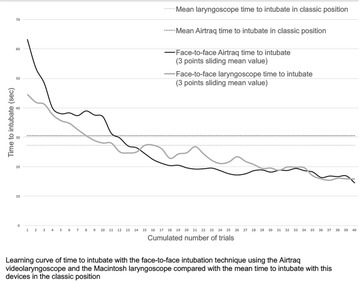


**Discussion:** The learning profile of FtFi is different between the Airtraq and the laryngoscope. It could be due to the participants’ lesser familiarity with the Airtraq. The better performances in FtFi could be due to better ergonomics allowing easier glottis exposure and learning (3).

**Conclusion:** Face-to-face intubation in sitting position is easy to learn. It provides better performances and fewer complications than the classic intubation technique which might result in a lower morbidity. The Airtraq provides even better results than Macintosh laryngoscope. All participants recommend their colleagues to be trained in face-to-face intubation.

### F-15 Non invasive ventilation use for Acute Chest Syndrome in French speaking Pediatric Intensive Care Units, online survey

#### Heilbronner Claire^1^, Grimaud Marion^1^, Oualha Mehdi^1^, Sommet Julie^1^, Rambaud Jerome^1^, Brousse Valentine^1^, Allali Slimane^1^, Renolleau Sylvain^1^

##### ^1^Hopital Necker Enfants Malades, Paris, France

###### **Correspondence:** Heilbronner Claire - claire.heilbronner@aphp.fr

*Annals of Intensive Care* 2018, **8(Suppl 1):**F-15

**Introduction:** Acute chest syndrome (ACS) is the leading reason of admission in Pediatric Intensive Care unit (PICU) for patients with Sickle Cell Disease (SCD). Non invasive ventilation (NIV) is nowadays unavoidable for the management of respiratory failure in PICU. Few studies assessed NIV’s impact on ACS outcome. We sought to investigate the clinical practice and particularly the use of NIV for ACS management in PICU or high dependency care unit (HDU) in France and French speaking countries.

**Patients and methods:** Participants were sought via e-mail through membership in the French Group for Paediatric Intensive Care Emergencies (GFRUP) to complete a 78-questions online survey for children admitted to PICU during the 1 year (2015). Demographics, clinical data and management’s description of ACS were collected.

**Results:** Seventeen PICUs or HDU with a total of 145 and 95 beds, respectively answered to the survey from June 2016, 10th to November 2016, 10th. Fifteen centres (88%) had a local transfusion center and 14 (82%) had a local blood centre and 14 (82%) were in connection with a SCD reference centre. During the 1 year 2015, 360 SCD patients were managed (median 7 SCD per centre, range 0–127) of which 137 (38%) for an ACS (median 4ACS per centre, range 0–34). Median length of PICU stay for ACS was 5 days, range 2–11 days. Three centres (totalling 62 beds) in the Parisian agglomeration hosted 146 SCD patients (40% of all SCD patients reported) with a total of 83 ACS (65% of ACS reported). Simple blood transfusion was performed in 73 patients with an ACS (53%). Sixteen patients (12%) received exchange transfusion. For patients who observed an ACS—bronchodilatators nebulisations were administrated in 47 (34%) of them, incentive spirometry was performed by 94 patients and 64 (47%) received standard oxygen therapy. Among non-invasive respiratory support, NIV with bilevel pressure was the most frequent (n = 68, 50%) before CPAP (n = 23, 17%) and high flow oxygen (n = 21, 15%). The proportion of patients on NIV was up to 71% in the centres hosting more ACS patients.

**Conclusion:** Despite the absence of evidence from randomized controlled trials NIV is nowadays commonly used in PICU and HDU for SCD patients with ACS, especially in centres taking in charge a high number of SCD patients. Future physiological studies and randomized controlled trials might help to choose between the different ventilatory support options for ACS.

### F-16 Transfusion practices in children with pediatric acute respiratory distress syndrome: a retrospective cohort study

#### Emeriaud Guillaume^1^, Paquin Marie-Eve^1^, Shemyakina Ilona^1^, De Cloedt Lise^1^, Tucci Marisa^1^, Lacroix Jacques^1^, Jouvet Philippe^1^

##### ^1^CHU Sainte Justine, Université de Montréal, Montréal, Canada

###### **Correspondence:** Emeriaud Guillaume - guillaume.emeriaud@umontreal.ca

*Annals of Intensive Care* 2018, **8(Suppl 1):**F-16

**Introduction:** Restrictive transfusion practices are increasingly used in pediatric intensive care unit (PICU), but evidence is lacking for deciding transfusion in children with severe acute respiratory distress syndrome (PARDS). This study aims to describe the red blood cells (RBC) transfusion practices during PARDS, to identify the main transfusion determinants in this population, and to describe the evolution of the patients after the transfusion.

**Patients and methods:** Patients with PARDS were prospectively screened in the PICU of a university affiliated pediatric hospital, from June 2015 to October 2016. Patients aged between 3 days and 18 years with a PARDS lasting at least 6 h were included. Baseline patient characteristics, PARDS data (for a period of 72 h), RBC transfusion characteristics, and main outcome data in the PICU were retrospectively collected in the electronic medical records. PARDS severity was classified based on PALICC definition. Results are reported as median (25^th^–75th percentile), and comparisons were made using Mann–Whitney test and Friedman test.

**Results:** 27 patients aged 36 (16–79) months were included. No transfusion was given in children with mild ARDS. At least one RBC transfusion was given during the PARDS period in 3 6 patients with moderate PARDS, and 7/15 patients with severe PARDS. At baseline, transfused patients had higher PRISM III score (10 (8–13) vs 4 (2–6), p < 0.05) and higher Oxygen Saturation Index (OSI 22 (13–27) vs 9 (6–18), p < 0.01). In transfused patients, the pre-transfusion hemoglobin was 8.1 (7.5–9.3) g dL in moderate PARDS and 7.5 (6.9–7.3) g dL in severe PARDS. The evolution of hemoglobin, OSI, ScvO2 and lactate after the transfusion is reported in the Table 1.

**Table 1:** Evolution of outcomes after transfusion

	H-6 to H0 (pre-transfusion)	H0 (transfusion) to H6	H6 to H24
Hemoglobin (g/dL) (n = 10)	7.8 ± 2.9	7.9 ± 2.5	9.5 ± 1.0
Oxygenation Saturation Index (n = 10)	21 (19–24)	15 (11–22)	21 (18–25)
ScvO_2_ (%) (n = 6)	72 (64–74)	64 (56–75)	57 (52–72)
Lactate (mmoL/L) (n = 5)	1.0 (0.7–1.3)	1.4 (1.0–1.6)	1.5 (1.4–1.8)

**Conclusion:** In our PICU, a relatively restrictive policy of RBC transfusion was observed even in patients with severe PARDS. Decision to transfuse seemed associated with the general severity status of the patient and with the hemoglobin level. Further studies are needed to explore the generalizability of these findings, and to investigate the impact of transfusion on oxygen transport consumption balance in pediatric acute respiratory distress.

### F-17 Impact of pediatric acute respiratory distress syndrome severity on adherence to PALICC guidelines for low tidal volume ventilation

#### Nardi Nicolas^1^, Sauthier Michael^1^, Proulx Francois^1^, Mortamet Guillaume^1^, Roumeliotis Nadia^1^, Jouvet Philippe^1^, Emeriaud Guillaume^1^

##### ^1^CHU Sainte Justine, Université de Montréal, Montréal, Canada

###### **Correspondence:** Nardi Nicolas - nicolas.nardi@hotmail.fr

*Annals of Intensive Care* 2018, **8(Suppl 1):**F-17

**Introduction:** Pediatric acute respiratory distress syndrome (PARDS) is associated with a high mortality rate of 30%. Lung protective strategies in ARDS advocate using low tidal volume and high positive end expiratory pressure (PEEP) while limiting the inspiratory plateau pressure in the recent international consensus conference on PARDS (PALICC)^1^. Our objectives were to evaluate the adherence to the guidelines for lung protective ventilation proposed by the PALICC and determine whether variations in tidal volume were related to the severity of oxygenation impairment.

**Patients and methods:** All patients, admitted to our tertiary pediatric ICU from January 2013 to May 2015, who received conventional ventilation for severe PARDS using PALICC definition and who had data to calculate ideal body weight (IBW) were retrospectively included. We excluded children with pulmonary bronchodysplasia and those admitted after cardiac surgery. Expiratory tidal volume (VTe), PEEP, peak inspiratory pressure, mean airway pressure, FiO2 and PaO2 were collected from electronic medical record (ICCA, Philips), using SQL Server 2008 (Microsoft, USA) + data were collected for 10 days following onset of conventional mechanical ventilation. Adherence proportions were analyzed with the non-parametric Cochran’s Q test, using cut-off values of VTe ≤ 8.0 mL kg. Mixed model analysis was performed to test the hypothesis that variations in tidal volume were related to the severity of oxygenation impairment.

**Results:** Twenty-one patients reached inclusion exclusion criteria, with median age of 5 years (interquartile [IQR] + 2.5–12 years) and a median weight of 17 kg ([IQR] + 12–30). Mortality rate was 38% (8/21) with a median duration of mechanical ventilation of 10 days ([IQR] + 7–32 days). On 1780 measurements of respiratory parameters, 72% of VTe measurements were ≤ 8.0 mL kg with median VTe of 6.7 mL kg ([IQR] + 5.6–8.3). During the first 10 days of conventional mechanical ventilation (159 patient-days), there were a significant variations in the daily rates of adherence to low tidal volume ventilation (p < 0.001) and a significant decrease in VTe with the highest oxygenation index (p = 0.02).

**Conclusion:** In our centre, adherence to PALICC guidelines for low tidal volume ventilation in PARDS is not perfect. However the adherence to protective ventilation strategy was higher in the most severe patients, in line with the recommendations. We plan to test if the implementation of a clinical decision support system can improve the adherence to guidelines at the bedside.

### F-18 Treatment level for children with polyhandicap admitted in ermergency in pediatric intensive care units in Paris

#### Tencer Jérémie^1^, Billette de Villemeur Thierry^2^, Leger Pierre-Louis^2^

##### ^1^Hôpital Necker, Paris, France; ^2^Hôpital Trousseau, Paris, France

###### **Correspondence:** Tencer Jérémie - jeremie.tencer@gmail.com

*Annals of Intensive Care* 2018, **8(Suppl 1):**F-18

**Introduction:** Children with polyhandicap are a complex medical and ethical situation. There are few studies about their admission in intensive care units, despite them having a strong risk of acute diseases. Our objetive was to describe the hospitalization of children with polyhandicap in intensive pediatric care units.

**Patients and methods:** This is a retrospective study in 4 pediatric intensive care units in Paris. Children with polyhandicap hospitalized in emergency from January 1st 2013 to December 31st 2015 were included. We looked at epidemiological datas, medical supports and medications at admission and treatment level (respiratory, circulatory, neurologic, invasive procedures), and limitations of treatments.

**Results:** 96 patients were included, representing 143 hospitalizations. At admission average age was 11 ± 5 years, mean number of medical support per patient was 1 ± 0.6 and mean number of neurological medication per patient was 2.5 ± 1.8. The main admission criteria were—respiratory failures (59%), neurological failures (20%) and circulatory failures (10%). Respiratory support was needed in 56%, circulatory support in 31% and nutritional support in 20% of patients. During these hospitalizations 25 consultation meetings have been held. Forty-two percent of patients did not have to be submitted to any invasive procedures. Overall mortality was 16%.

**Conclusion:** children with polyhandicap admitted in intensive care units are at school age and are mostly having respiratory or neurological failures. There is a strong rate of rehospitalisation. There are few therapeutic limitations that have been found. Anticipating a crisis is a major point to provide the best care for these children.

### F-19 Piperacillin dosing regimen optimization in critically ill children according to their renal function

#### Béranger Agathe^1^, Benaboud Sihem^2^, Urien Saïk^3^, Moulin Florence^4^, Bille Emmanuelle^4^, Genuini Mathieu^4^, Lesage Fabrice^4^, Hirt Déborah^5^, Renolleau Sylvain^4^, Tréluyer Jean-Marc^4^, Oualha Mehdi^2^

##### ^1^Montrouge, France; ^2^Université Paris Descartes, Paris, France; ^3^Hôpital Tarnier, Université Paris Descartes, Paris, France; ^4^Hôpital Necker, Université Paris Descartes, Paris, France; ^5^Hôpital Cochin, Paris Descartes University, Paris, France

###### **Correspondence:** Béranger Agathe - agathe.beranger@gmail.com

*Annals of Intensive Care* 2018, **8(Suppl 1):**F-19

**Introduction:** Pharmacokinetic parameters are altered in critically ill patients. For instance, in adult patients, it has been well demonstrated that augmented renal clearance results in subtherapeutic antibiotic concentrations. Our objectives were to build a pediatric population pharmacokinetic model for Piperacillin, in order to optimize individual dosing regimen.

**Patients and methods:** All children admitted in pediatric intensive care unit, aged less than 18 years, weighing more than 2.5 kg, and receiving intermittent Piperacillin infusions were included. Piperacillin was quantified by high performance liquid chromatography. Pharmacokinetics were described using the non-linear mixed effect modelling software MONOLIX. Monte Carlo simulations were used to optimize dosing regimen, in order to maintain plasma concentration above the minimum inhibitory concentration (16 mg L^−1^ for Pseudomonas aeruginosa) throughout the dosing interval (100% fT > MIC).

**Results:** We included 50 children with a median (range) post natal age of 27.2 (1.1–222.9) months, median (range) body weight of 11.9 (2.7–50) kg, median (range) PELOD-2 score of 4 (0–16) and median (range) estimated creatinine clearance of 142 (29–675) mL.min^-1^.m^-2^. A one compartment model with first-order elimination adequately described the data. Median (range) values for Piperacillin clearance and volume of distribution were respectively 3 (0.71–10) L h^−1^ and 3.8 (0.72–25.8) L. Body weight (allometric relationship), estimated creatinine clearance and PELOD-2 severity score were the covariates explaining the estimated between subject variability. A third of the cohort attained the target, according to our dosing regimen and to the European guidelines. To reach the target and according to the simulated dosing regimens, children with acute kidney injury should receive intermittent infusion every 6 h, administered on 30 min. Those with augmented renal clearance should receive a continuous infusion.
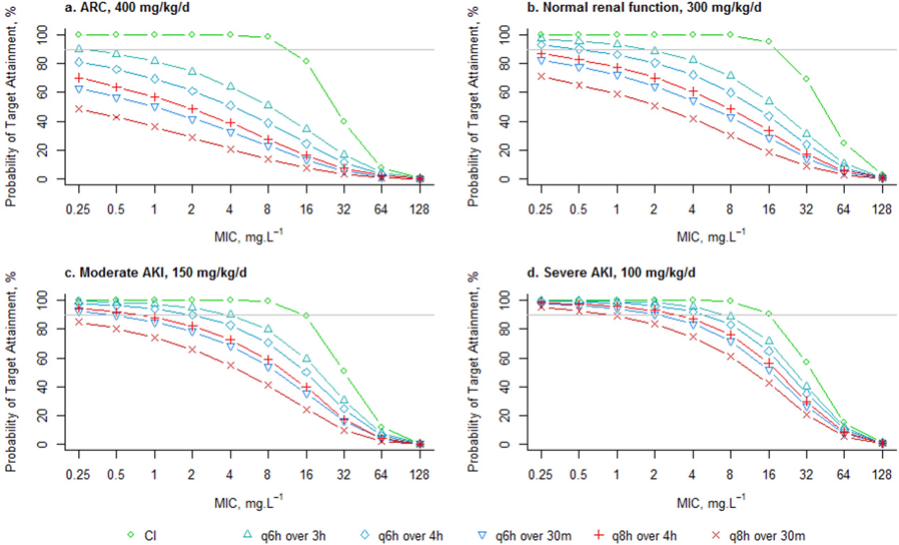


**Conclusion:** To reach the target, standard intermittent Piperacillin dosing regimen in critically ill children is not appropriate. In addition to body weight, dosing regimens should take into account the creatinine clearance. Continuous infusion is adequate for children with augmented renal clearance. Piperacillin individualized dosing regimens and therapeutic drug monitoring are mandatory in pediatric intensive care unit.

### F-20 Comparison between the NIPE^®^ index (Neonatal Infant Parasympathetic Evaluation) and the DAN scale for pain assessment during acute procedural pain in preterm infants

#### Ros Barbara^1^, Tandonnet Olivier^1^, Cramaregeas Sophie^1^, Duchateau Josselin^1^, Brissaud Olivier^1^, Nolent Paul^1^, Renesme Laurent^1^

##### ^1^CHU de Bordeaux, Bordeaux, France

###### **Correspondence:** Ros Barbara - rosbarbara33@gmail.com

*Annals of Intensive Care* 2018, **8(Suppl 1):**F-20

**Introduction:** All data support the need for early recognition, evaluation of pain in the NICU. Multiparametric analysis including physiological parameters could be useful to have a more objective evaluation of pain in the NICU compared to scales built on external-evaluation. The Newborn Infant Parasympathetic Evaluation (NIPE^®^) was developed to assess pain in newborns and infant, from preterm to the age of 2 years.

**Patients and methods:** We conducted a monocentric, prospective study to compare the instantaneous NIPE^®^ index value (NIPEi^®^) to the DAN scale during acute procedural pain (PICC line insertion) in preterm infants (under 37 GW). The operators and the nurse were blinded to the continuous recording of NIPEi^®^ during the entire procedure. DAN scale was assessed every 5 min by a third person, trained to this scale and blinded to NIPEi^®^. A direct correlation assessment between the DAN scale and the NIPEi^®^ was performed by calculating the Pearson’s linear correlation coefficient. The differences between the NIPEi^®^ of non-painful (DAN < 3) and painful (DAN ≥ 3) infants were estimated by the Wilcoxon–Mann–Whitney test. The usefulness of NIPEi^®^ as a new tool for pain assessment in neonates was estimated by the corresponding ROC curve. Our study was approved by our local ethic institutional review board.

**Results:** Thirty-five preterm infants were included, NIPEi^®^ data were incomplete in 3 infants. Fifty percent of newborns were born before 31 GW, and 66% had non-invasive respiratory support (continuous positive airway pressure CPAP). At the time of the procedure, newborns had a median post-natal age of 3 days and a median weight of 1180 grams. There was a moderate correlation between the NIPEi^®^ index and the DAN scores (r = 0.605 + p < 0.001). The median NIPEi^®^ index was 59 for non-painful events vs. 43 for painful events, p < 0.001. The area under the ROC curve was 0.92. For a threshold of NIPEi^®^ < 50, the sensitivity was 91.3%, the specificity was 83%. Positive likelihood ratio was 5.36 and the negative likelihood ratio was 0.10 (Fig. 1).

**Conclusion:** We showed a correlation between the DAN scale and the NIPEi^®^ index for pain assessment in preterm infants. The NIPE^®^ monitor could be a useful and non-invasive tool for pain assessment in neonates. Further studies are needed to confirm our results and to define more precisely the place of such monitors for pain evaluation in daily clinical practice in the NICU.

**Fig. 1:**
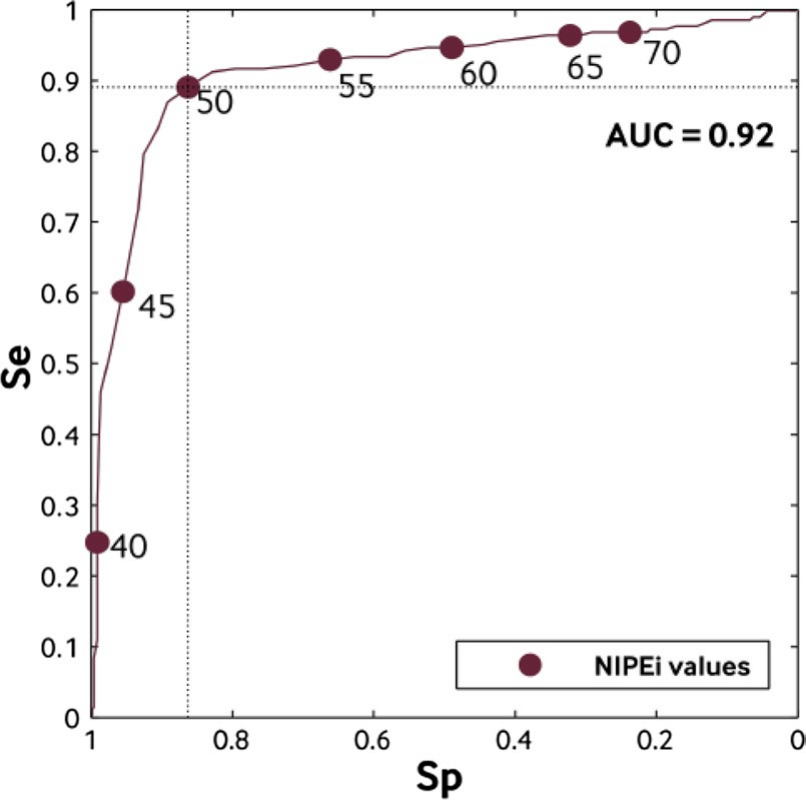
NIPEi ROC curve. *NIPEi* instantaneous Newborn Parasympathetic Evaluation index, *Sp* Specificity, *Se* Sensibility. Area under the curve (AUC): 0.92

### F-21 Prospective evaluation of acute pituitary hormone dysfunction following severe Traumatic Brain Injury (endocTC study)

#### Javouhey Etienne^1^, Courtil-Teyssedre Sonia^1^, Cour-Andlauer Fleur^1^, Wroblewski Isabelle^2^, Desrumeaux Amélie^2^, Ginhoux Tiphanie^1^, Dupuis Clémentine^2^, Bernoux Delphine^1^, Kassai Behrouz^1^, Nicolino Marc^1^, Faure Patrice^2^, Plotton Ingrid^1^

##### ^1^Hospices Civils de Lyon, Bron, France; ^2^CHU Grenoble, La Tronche, France

###### **Correspondence:** Javouhey Etienne - etienne.javouhey@chu-lyon.fr

*Annals of Intensive Care* 2018, **8(Suppl 1):**F-21

**Introduction:** The aim was to identify factors associated with the occurrence of acute pituitary hormone dysfunction in children with moderate to severe TBI and to describe the impact of this dysfunction on the stability of the children.

**Patients and methods:** Prospective bicenter study including all children aged between 1 month to 17 years, admitted to PICU for a moderate-severe TBI and with an expected stay > 2 days. Setting—Pediatric intensive care units of Grenoble and Lyon, from 2010 to 2017. Endocrine explorations at the second morning following admission and 24 h before discharge were performed—cortisol 24 h cycle with free cortisol and ACTH dosages every 4 h (or 6 h if no central line) + free 24H urinary cortisol + TSH and T4L, 24H urinary LH and FSH, blood level of testosterone or estradiol for children aged > 10 years, and IGF1. Patients were classified as having cortisol insufficiency if all the cortisol dosages were < 200 nmol l and all ACTH were < 12 pg l. TSH deficiency was defined as T4 l < 11.5 pmol l and TSH < 4.2 mUI l. Gonadotropin defciency was defined as urinary LH < 0.14 UI 24 h and urinary FSH < 1.52 UI 24 h for males + urinary LH < 0.018 UI 24 h and urinary FSH < 2.24 UI 24H in female. Patients with deficiency (ACTH and any deficiency) were compared to those without deficiency in terms of hemodynamic instability, respiratory instability, neurological and infectious complications For continuous variables means and 95% confidence interval were calculated and compared by *t* student test. Chi-2 test was used to compare proportions.

**Results:** Among the 110 patients evaluated, 12 had ACTH deficiency, and 23 had at least one acute pituitary dysfunction. Comparison of patients who presented ACTH deficiency with those who were not deficient found no differences in terms of patients characteristics, cause of TBI, level of severity and level of injury. Paitents with ACTH deficiency required more frequently fluid bolus at day2 (67 vs 34%, p = 0.03). All the markers of severity were higher and the need of vasoactive drugs were more frequent but the differences were not statistically significant. Table 1 shows comparison between patients with at least one pituitary hormone deficiency to those without deficiency. The same result was found. Glycemia levels were lower in the group with deficiency.

**Table 1:** Comparison of moderate-severe TBI children with at least one pituitary hormon deficiency to those without deficiency

	Population	Any Pituitary hormon deficiency	No deficiency	p
Number (%)	110	23 (21%)	87 (79%)	
Age (years)	7.8 (2.1–12.5)	9.4 (7.2–11.7)	7.38 (6.2–8.5)	Ns
Male	73 (66%)	19 (83%)	54 (62%)	Ns
Body weight (kg)	30 (14–45)	37.4 (28.4–46.4)	28.1 (24.1–32)	Ns
Cause of TBI				Ns
Road Traffic Accident	56 (51%)	12 (52%)	44 (51%)	
Inflicted	12 (11%)	0	12 (14%)	
Fall	33 (30%)	10 (44%)	23 (26%)	
Blunt I	7 (6%)	1 (4%)	6 (7%)	
Penetrating	2 (2%)	0	2 (2%)	
GCS	7 (5–9)	7 (5–8)	7 (6–7)	Ns
Pupillary dilatation	25 (23%)	3/22 (14%)	22/85 (26%)	Ns
ISS	26 (17–33)	29 (24–33)	25 (23–27)	Ns
PIM2	9.2 (3.9–8.7)	10.2 (3.4–16.9)	9 (6.6–11.3)	Ns
PELOD	16.8 (11–22)	17.8 (12.2–23.5)	16.6 (14.4–18.9)	Ns
Intracranial hypertension	53 (49%)	13 (57%)	40/86 (47%)	Ns
Neurosurgery	32 (29%)	4 (17%)	28/86 (33%)	Ns
IV Fluid Bolus				
Day 1	71 (65%)	16 (70%)	55/86 (64%)	Ns
Day 2	41 (38%)	15 (65%)	26/86 (30%)	0.002
Day3	19 (18%)	7 (30%)	12/85 (14%)	Ns
Vasoactive drugs				
Day 1	34 (31%)	9 (39%)	25/86 (29%)	Ns
Day 2	20 (18%)	6 (26%)	14/86 (16%)	Ns
Day 3	17 (16%)	5 (22%)	12/85 (14%)	Ns
Hemodynamic instability (until PICU discharge)				
	3 (3%)	0/22	3/82 (4%)	Ns
PICU stay	12.4 (4–12)	9.9 (6.6–13.3)	13 (6.3–19.8)	Ns
POPC (PICU discharge)	3 (2–4)	3 (2–3)	2 (2–3)	Ns
Death (PICU)	3 (2.7%)	0	3	

**Conclusion:** We didn’t find any predictive factors of pituitary hormone deficiency in children with moderate-severe TBI justifying a systematic screening of those patients.

### F-22 Can we satisfy parents of patients in intensive care unit ?

#### Fathallah Ines^1^, Tobich Mariem^1^, Habacha Sahar^1^, Ben El Arbi Rim^1^, Ben Abderahim Amina^1^, Sakis Dorra^1^, Kalel Hela^1^, Boujelben Mohamed^1^, Selami Ali^1^, Kouraichi Nadia^1^

##### ^1^Hôpital Régional de Ben Arous, Ben Arous, Tunisia

###### **Correspondence:** Fathallah Ines - ines822004@yahoo.fr

*Annals of Intensive Care* 2018, **8(Suppl 1):**F-22

**Introduction:** Most intensive care unit (ICU) patients cannot make decisions themselves. Familiy members are actively involved in the care process as surrogate decision-makers and judges of care quality. However, family satisfaction with care is complex and is not clearly defined. The aim of this study is to evaluate the different procedures (reception book and staff education for aid and relationship) used in a new ICU to improve the family care.

**Patients and methods:** We included in our study patients who had spent more than 48 h in our department. A questionnairy, adapted to our population, was performed by our staff and validated by the Hygiene and Quality Care departement. We proceded by phone calls, 10 months after the inauguration of our ICU.

**Results:** Sixty-five questionnaires were included (Fig. 1). The average of age was 52 ± 20 with a sex ratio of 2. The average of the simplified acute physiology score (SAPSII) was 36 ± 17. The median stay was 7 days [4–16] with a total mortality rate of 25%. Mostly, we interrogated first-degree parents (n = 52). Only three families recieved reception book at admission. Visit in patient room was autorised only for 27% (n = 17) of family members. Only four persons said they were disturbed in visit hours for architectural reasons (tight space). Disponibility was found excellent in 40% (n = 24) of cases for medical staff, 45% (n = 26) for paramedicals. Informations provided by physicians were clear in 71.5% (n = 53) of cases. Fifteen of the family members (23%) asked psychology support. Only 15% of parents felt involved in the management care decisions. Most parents (87%) opted for making all dicisions related to doctors. Patients management was judged excellent in 74% (n = 48) of cases. There was no difference according to satisfaction between families of dead patients and families of surviving ones.

**Fig. 1:**
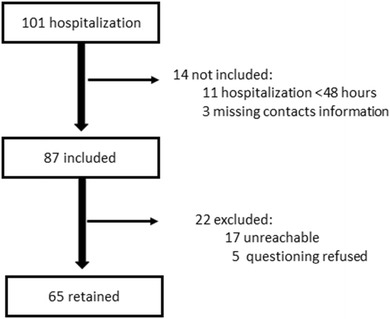
Questionnaire results

**Conclusion:** To optimise patients care and families satisfaction specific psychological support is necessary.

### F-23 Predictors of 1 year mortality rate for AE COPD ICU-admitted patients

#### Ben Saida Imen^1^, Zarrougui Wafa^1^, Ennouri Emna^1^, Fraj Nesrine^1^, Limam Manel^1^, Ayachi Jihene^1^, Sma Nesrine^1^, Khedher Ahmed^1^, Azouzi Abdelbaki^1^, Boussarsar Mohamed^1^

##### ^1^Hôpital Universitaire Farhat Hached, Sousse, Tunisia

###### **Correspondence:** Ben Saida Imen - imen.bensaida@yahoo.com

*Annals of Intensive Care* 2018, **8(Suppl 1):**F-23

**Introduction:** Acute exacerbations of COPD (AE COPD) is one of the most common causes of admission in the intensive care unit (ICU). The prognosis of COPD requiring ICU seems to be poor with high in-hospital and long-term mortality. Predictors of post ICU mortality for those patients are still not well defined. This study was carried out to define the predictors of 1 year mortality in patients admitted to the ICU with AE COPD.

**Patients and methods:** A prospective cohort study was performed in a Tunisian medical ICU between 2014 and 2016. A follow-up was performed on ICU survivors admitted for AE COPD during 1-year using phone interviews. Univariate and multivariate analyses were used to predict 1 year mortality. Stepwise backward elimination was performed to identify independent factors, with retention of predictors with p < 0.2.

**Results:** Among 119 patients admitted for AE COPD in ICU during the study period, 76(63.8%) patients were discharged alive. The mean age was 67.24 ± 10.9 years. 85% were male. Exacerbations were mainly caused respectively by—tracheobronchitis, 49(81.7%), community acquired pneumonia, 5(8.3%), lung edema, 4(6%) and bronchial hyperresponsiveness, 2(3.3%). 36(60%) required intubation, and the median duration of mechanical ventilation (MV) was 2 days with extremes ranging from 0 to 21 days. 30(39.5%) required inotropic agents. The most common ICU adverse events were respectively acute renal failure 20(26.3%), cardiac arrhythmia 21(35%), nosocomial infection in 9(15%) and difficulty in weaning 10(13.2%). The in-hospital mortality rate for the study group was 36%. The follow up was performed for 60(79%). The mortality rate at 1 year was 33.33%. Univariate analysis identified three factors associated with 1 year fatal outcome—invasive mechanical ventilation (p = 0.0015), WHO performance status (p = 0.0001) and decline in functional status (p = 0.000). Multivariate logistic regression analysis showed only two independent variables associated with long term mortality—SAPS II (OR, 1.14 + 95% CI [1.04–1.25] + p = 0.004) and performance status at discharge (OR, 3.11 + 95% CI [1.55–6.23] + p = 0.001).

**Conclusion:** In the present study, severity of illness and physiological reserve at discharge were identified as independent predictors of long term mortality after ICU discharge in AE COPD. Therefore, caregivers should allocate adequate strategies for the prevention and control of COPD patients.

### F-24 Long-term outcome according to ventilation mode in ICU survivals from AE COPD

#### Ben Saida Imen^1^, Zarrougui Wafa^1^, Ennouri Emna^1^, Sma Nesrine^1^, Limam Manel^1^, Fraj Nesrine^1^, Ayachi Jihene^1^, Khedher Ahmed^1^, Azouzi Abdelbaki^1^, Boussarsar Mohamed^1^

##### ^1^Hôpital Universitaire Farhat Hached, Sousse, Tunisia

###### **Correspondence:** Ben Saida Imen - imen.bensaida@yahoo.com

*Annals of Intensive Care* 2018, **8(Suppl 1):**F-24

**Introduction:** Randomized controlled trials have confirmed the evidence and helped to define when and where NIV should be the first line treatment of acute exacerbation of COPD (AE COPD). Its effectiveness in preventing endotracheal intubation and reducing intensive care mortality is undeniable. Few studies have evaluated the relationship between ventilation modalities (noninvasive ventilation [NIV] versus invasive mechanical ventilation [IMV] and long-term survival in COPD patients treated in the intensive care unit (ICU). The aim of this study was to investigate one-year survival stratified by mechanical ventilation modality in COPD patients treated in the ICU.

**Patients and methods:** An observational prospective cohort study including all patients admitted to a Tunisian Tertiary ICU for AE COPD and discharged alive between January 2014 and December 2015. Patients were followed up via phone calls during 1 year after discharge. Characteristics on admission and outcomes after discharge were analyzed stratified by ventilation modality NIV vs IMV. The overall survival was analyzed on the basis of the Kaplan–Meier curves.

**Results:** During the predetermined period of data collection, the follow-up involved 60 patients. 24 patients were treated by NIV (group1) and 36 patients needed IMV (group2). There was no difference between the 2 groups in age (p = 0.69), severity of COPD (p = 0.39), physiological reserve at discharge (p = 0.14) and ICU readmission (p = 1). Short term outcomes were not different between the 2 groups—1-month readmission (16.7 vs 25% respectively in NIV and IMV, p = 0.52) and 1-month mortality (4.2 vs 19.4%, p = 0.18). However, one-year Mortality rate was lower in NIV group (16.7 vs 44.4%, p = 0.02). Long-term survival analyzed with the Kaplan–Meier method was significantly greater in the NIV group (p = 0.016) (Fig. 1).

**Fig. 1:**
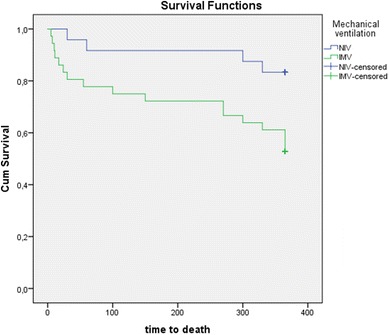
One-year long-term survivals according to ventilation mode (NIV, blue line vs IMV, green line) in ICU survivals from AE/COPD

**Conclusion:** In the present study while the characteristic of AE COPD patients’ prior to ICU admission and at discharge were similar, long term survival was higher in the NIV managed patients. Invasive mechanical ventilation seems to be a negative salient feature in the natural course of the COPD. Avoiding IMV by early treatment with NIV in AE COPD needing critical care is associated with better long-term survival.

### F-25 A predictive triage model for ICU discharge to identify patients at risk of 30-day post ICU mortality

#### Ben Saida Imen^1^, Ennouri Emna^1^, Zarrougui Wafa^1^, Ayachi Jihene^1^, Limam Manel^1^, Fraj Nesrine^1^, Sma Nesrine^1^, Khedher Ahmed^1^, Azouzi Abdelbaki^1^, Boussarsar Mohamed^1^

##### ^1^Hôpital Farhat Hached, Sousse, Tunisia

###### **Correspondence:** Ben Saida Imen - imen.bensaida@yahoo.com

*Annals of Intensive Care* 2018, **8(Suppl 1):**F-25

**Introduction:** The major challenge of intensivists is to reduce intensive care mortality rate. However, less attention is paid to outcomes beyond ICU discharge. A predictive triage model may influence decision making at ICU discharge and hence help caregivers to decrease short term post ICU mortality. The aim of this study was to develop a predictive triage model for discharge to identify patients at risk of 30 days post ICU mortality.

**Patients and methods:** An observational prospective cohort study was performed in a Tunisian medical ICU between January 2014 and December 2015. The study included all survivors after ICU admission. The mortality was assessed by telephone interviews at thirty days after discharge. Univariate and multivariate analysis were performed to identify candidate variables for the model.

**Results:** Among 573 patients admitted during the study period, 215 were included. The 30 days mortality rate was 16%. In univariate analysis, predictors of 30-days mortality of ICU survivors were Charlson comorbidity index (p = 0.001), baseline functional impairment ≥ 3 (p = 0.003), SAPS II ≥ 30 (p = 0.0001), Tachycardia (Heart rate ≥ 90b min) at discharge (p = 0.011), performance status ≥ 3 (p = 0.0001) and decline in functional status (p = 0.0001). Multivariate regression analysis identified the following independent risk factors, SAPS II ≥ 30 (OR, 3.258 + 95% CI [1.1–9.6] + p < 0.032), tachycardia at discharge (OR, 3.024 + 95% CI [1.01–9.11] + p < 0.049), decline in functional status (OR, 15.868 + 95% CI [15.18–48.56] + p < 0.000) and performance status ≥ 3 (OR, 6.57 + 95% CI [2.03–21.25] + p < 0.002). Based on these results, the following variables were used to develop the model—SAPSII ≥ 30, tachycardia, decline in functional status and performance status ≥ 3. These four items were weighted by their respective OR SAPSII ≥ 30, 3 points + tachycardia, 3 points + decline in functional status, 16 points + performance status ≥ 3, 6 points. The area under the ROC curve value of this predictive model obtained from the development cohort was 0.914 (95% CI [0.86–0.96]), a level deemed to be good discrimination (Fig. 1).

**Fig. 1:**
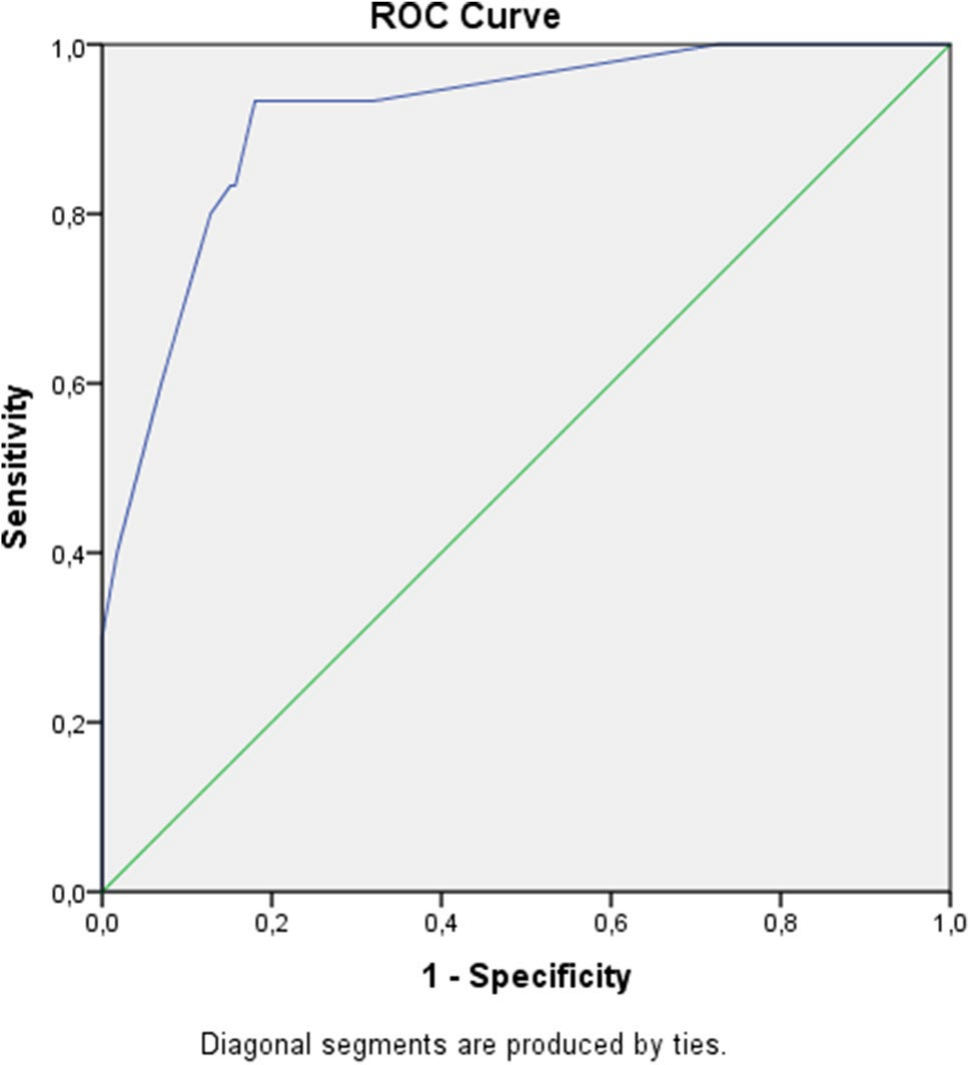
Receiver operator characteristic curve of ICU discharge triage model to identify patients at risk of 30-day post ICU mortality. (AUC, 0.914; 95% CI [0.86–0.96])

**Conclusion:** This discharge triage model is a simple, didactic, not time-consuming, costless and minimally invasive tool. It may afford the opportunity to critical care practitioners to identify patients at risk of early post-ICU mortality.

### F-26 Child morbidity and mortality in an adult polyvalent intensive care unit

#### Merhabene Takoua^1^, Ben Slimène Abdelaziz^1^, Jamoussi Amira^1^, Ayed Samia^1^, Ben Khelil Jalila^1^, Besbes Mohamed^1^

##### ^1^Hôpital Abderrahman Mami, Ariana, Tunisia

###### **Correspondence:** Merhabene Takoua - takouamg@yahoo.fr

*Annals of Intensive Care* 2018, **8(Suppl 1):**F-26

**Introduction:** Background—intensive care physicians rarely have to manage children less than 18 years of age. In Tunisia, lack of pediatric intensive care units (ICU) requires their hospitalization in adult units. Purpose—To assess the effectiveness of child care in a polyvalent adult ICU and determine the relevant mortality risk factors.

**Patients and methods:** Methods—Retrospective study including children less than 18 years old at admission in the adult polyvalent intensive care unit of Abderrahman Mami Hospital in Ariana between January 1st 2008 and August 31th 2017. Data were analyzed using Student test or logistic regression as appropriate.

**Results:** Out of 4994 admissions, 80 patients (incidence = 1.6 1000 admission) were enrolled according to established criteria. 10.1% were asthmatic, 3.9% were diabetic, 2.6% have a neuromuscular disease and 83.4% have no previous comorbidities. Mean age was 14.9 ± 3 years [5–18]. Sex ratio was 1.42. Acute respiratory failure (69.7%) complicating community acquired pneumonia, neurologic disorders (15.6%) and acute poisoning (12.8%) were the main causes of admission. References from our emergency room represent 53.7% of cases, wards of our hospital 19.1%, pediatric units 5.2% and onco-hematologic units 5.2%. Mean PRISM during the first 24 h was 6 ± 6.6. Septic shock was observed in 3.9% of cases, 36.6% were invasively ventilated, 22.5% were non-invasively ventilated, 8.75% had a combination of invasive and non-invasive ventilation (NIV) and 41.2% did not receive ventilatory support. 20% of patient developed complications during their hospital stay—it was predominately nosocomial infection (16.6%) complicated by septic shock in 22.5%, acute renal failure (13.8%) and barotrauma (5.6%). Average ICU stay was 6.28 ± 9.72 days with a mortality rate of 21.3%. Multivariate analysis showed that independent factors predictors of mortality were—organ dysfunction at admission (acute respiratory failure, coma and hemodynamic failure), use of mechanical ventilation, occurrence of complications during hospital stay and PRISM ≥ 18.32.

**Conclusion:** Pediatric mortality remains high in our adult intensive care unit. Presence of organ failure at admission and occurrence of complications were the major factors for worse prognosis.

### F-27 Current knowledge of the ICU healthcare providers on the post-intensive care syndrome

#### Prevedello Danielle^1^, Devroey Marianne^1^, Maetens Yves^1^, Preiser Jean-Charles^1^

##### ^1^Hôpital Erasme, Bruxelles, Belgique

###### **Correspondence:** Prevedello Danielle - danielle.prevedello@ulb.ac.be

*Annals of Intensive Care* 2018, **8(Suppl 1):**F-27

**Introduction:** Post-intensive care syndrome (PICS) has been recently described as a combination of physical, cognitive and mental impairments appearing during a stay in an intensive care unit (ICU). The prevention and detection of PICS require the participation of each category of healthcare workers. However, the level of knowledge is unknown. We sought to assess the awareness among our ICU staff in preparation for a follow-up consultation.

**Patients and methods:** The study used a short multiple-choice survey filled on a voluntary basis. All members of the staff were asked to fill the questionnaire over a one-week period. The assessment was composed by seven structured questions which aimed measure basic knowledge of post-intensive care syndrome and general strategies to diagnose that syndrome and the tests used.

**Results:** Fifth five workers (30% of the staff) of the department of intensive care answered the questionnaires (67% nurses, 7% physiotherapists, 20% physicians). The estimated ranges of prevalence of psychological problems were very low (0–25%) for 3.63%, low (25–50%) for 25.45%, intermediate (50–75%) for 50.9%, and high (75–100%) for 18.18%. The ability to return at work was thought as very low, low, intermediate and high for 49.09, 43.63, 5.45 and 0% of the respondents, respectively. The other results are displayed in the Table 1.

**Table 1:** PICS current knowledge

	Positive answers (%)
Existence of PICS	22
Knowledge of IPREA	10.9
Knowledge of ICU memory tool	12.7
Knowledge of HADS	18.2
Knowledge of score MRC	20
Knowledge of sit to stand test	16.4
Knowledge of Katz score	49.1

**Discussion:** In spite of the sample size and of the voluntary character of this survey, these data indicate an ample room for improvement of the awareness of PICS in our ICU. The knowledge of the specific tests used for the assessment of PICS is mostly determined by the professional background. Hence, the implementation of a follow-up clinic is challenging as it requires educational training. Large similar projects have been successful after intensive training and awareness campaigns.

**Conclusion:** Knowledge of PICS is poor in the absence of training and education. Post-ICU follow-up clinics require skill and motivation from a large variety of ICU healthcare workers.

### F-28 Evaluation of death certificates drafted in emergency and intensive care departments

#### Wiem Ben Amar^1^, Zribi Malek^1^, Ennouri Hassen^1^, Karray Narjess^1^, Bardaa Sami^1^, Hammami Zouheir^1^, Maatoug Samir^1^

##### ^1^Hôpital Habib Bourguiba, Sfax, Tunisia

###### **Correspondence:** Wiem Ben Amar - wiembenamar@yahoo.fr

*Annals of Intensive Care* 2018, **8(Suppl 1):**F-28

**Introduction:** Drafting a death certificate (DC) is a procedure considered as a part of doctor’s daily practice, especially in emergency and intensive care departments. This certificate represents a civil, social, epidemiological and medico-legal act. It can engage the liability of the certifying doctor. The objectives of our study were to examine the content of DC drafted in emergency and intensive care departments, assess the quality of writing, and analyze drafting errors.

**Patients and methods:** A prospective study extended over a period of 12 months from January to December 2015, including all DC emanating from emergency and intensive care departments and received in the forensic department of Habib Bourguiba hospital in Sfax.

**Results:** During the study period, 120 DC meeting the inclusion criteria were collected. Although confidential, the medical part of the DC was sealed by the doctor in onlyone third of cases. In the administrative section, nine socio-demographic parameters were studied. In 10% of the cases, less than four of the nine criteria were found. In the section concerning the certifying doctor data, 7 parameters were screened. 67.1% of the certifying doctors met at least six criteria. The most frequently missing parameter in this section was the identity of the person to whom the certificate was issued. The identity of the doctor was not mentioned in 10% of the cases. Forensic data (4 items) was complete in over three quarters of the certificates. Nevertheless, in 26.7% of cases, the medicolegal obstacle to burial box was left empty (8.3%) or not ticked even if judicial investigation was required (18.3%). The section on causes of death was the source of almost all of the drafting errors. We have classified these errors into six major ones, according the classifications reported in the literature. The percentage of certificate without faults was 13%. The most common major error was insufficient cause of death found in 40.4% of cases followed by incorrect sequence of causes of death (28.8%), medicolegal obstacle to burial not ticked although required (26.6%), several causes of death mentioned simultaneously (16.7%), unacceptable cause of death (10.8%) and mechanism of death mentioned instead of the cause of death (5.8%).

**Conclusion:** Our study showed that the quality of drafting of DC suffered from several insufficiencies, which encourages us to provide more effort in training doctors and to review the current official model of DC.

### F-29 Validation of a delocalized measurement method of lactate for septic patients

#### Jouffroy Romain^1^, Leguillier Teddy^1^, Boisson Marie^1^, Boussaroque Agathe^1^, Nivet Antoine Valérie^1^, Beaudeux Jean Louis^1^, Vivien Benoit^1^

##### ^1^Hôpital Necker Enfants Malades, Paris, France

###### **Correspondence:** Jouffroy Romain - romain.jouffroy@gmail.com

*Annals of Intensive Care* 2018, **8(Suppl 1):**F-29

**Introduction:** Septic shock is defined as a sepsis with hyperlactaemia greater than 2 mM after correction of hypovolemia requiring vasopressors to maintain MBP > 65 mmHg [1]. It can be observed in pre-hospital emergency medicine (PHEM). The use of a reliable portable device for measuring lactate in PHEM would allow a better evaluation of septic patient facilitating their orientation towards intensive care unit (ICU) or emergency department (ED). This portable delocalized biology device must be validated against the laboratory reference method (NFEN ISO 22870) [2]. The aim of this study was to clarify the validity of a delocalized measure of lactatemia.

**Patients and methods:** We performed a prospective study including 47 patients admitted into ICU for septic shock (CPP number 2015-08-03 SC). Lactate was measured in parallel on 2 samples—one capillary with the portable device (Lactate StatStrip Xpress, Nova Biomedical) and the other venous on a centrifuge tube for plasma analysis (Architect C16000 Abbott Diagnostics). We evaluated the analytical performance (coefficients of variation (CV) for repeatability and reproducibility evaluated at 2 levels of quality control (QC)—1.6 and 3.6 mM) and then the concordance between lactate levels measured by the devices and lactate levels measured by laboratory analyzer.

**Results:** At the QC concentrations tested, the CVs were in agreement with the limits set by the French Society of Clinical Biology—CV < 3% for repeatability and < 5% for reproducibility. An excellent correlation was observed between the 2 measurements—correlation coefficient R2 = 0.98, slope = 0.95 and ordered at the origin = 0.1. The latter suggested a low positive bias of the device not confirmed by Bland–Altmann graph analysis and graph of the differences.

**Conclusion:** We verified the analytical performance of the device and showed an excellent correlation with the laboratory measurement. The delocalized measure can be used in PHEM in patients with suspected sepsis syndrome. This measure should allow a more accurate and early assessment of their severity in order to improve triage and hospital orientation between ED and ICU.

### F-30 Association between hyperoxia and mortality for patients treated by Extra Corporeal Life Support after Out Hospital Cardiac Arrest

#### Halter Maryline^1^, Jouffroy Romain^1^, Saade Anastasia^1^, Philippe Pascal^1^, Carli Pierre^1^, Vivien Benoit^1^

##### ^1^Hôpital Necker Enfants Malades, Paris, France

###### **Correspondence:** Halter Maryline - haltermaryline@orange.fr

*Annals of Intensive Care* 2018, **8(Suppl 1):**F-30

**Introduction:** We examine whether values of PaO2 taken immediately after starting Extra Corporeal Pulmonary Resuscitation (eCPR) inserted in a intensive care unit (ICU) is associated with death in patients treated by eCPR after refractory Out Hospital Cardiac Arrest (OHCA).

**Patients and methods:** We performed a retrospective observational study. Patients were divided into 3 groups defined on oxygen partial arterial pressure (PaO2) on the first arterial blood gas analysis after starting eCPR. Hyperoxia was defined as PaO2 greater than 300 mmHg + hypoxia as a PaO2 of less than 60 mmHg and normoxia, not classified as hyperoxia or hypoxia. The main outcome was mortality at day 28 (D28).

**Results:** Sixty-six consecutive patients, 77% male, with a mean age of 51 ± 14 years, were admitted to the ICU after refractory OHCA requiring eCPR. Aetiologies of OHCA were mainly due to acute coronary syndrome (67%), hypertrophic cardiomyopathy (8%) and cardiotoxic overdose (8%). Mortality at D28 reached 61%. Mean PaO2 at admission was 227 ± 124 mmHg. We observed an association between D28 mortality and PaO2—OR 1.010 [1.005–1.015], (p = 0.0005). Area Under Curve (AUC) for PaO2 after starting eCPR was 0.77 [0.65–0.89]. In a model controlling for potential confounders (age, no flow and lo flow duration, lactate level, pH and carbon dioxide partial pressure), hyperoxia exposure had an odds ratio for D28 mortality of 3.92 (95% CI 1.14–16.50) (p = 0.04) whereas in normoxia had an odds ratio for D28 mortality of 0.25 (95% CI 0.06–0.91) (p = 0.04).
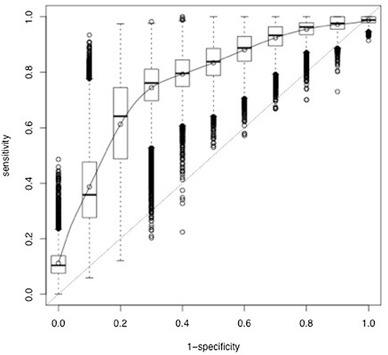


**Conclusion:** There is an association between mortality at D28 and hyperoxia in patients admitted in ICU for refractory OHCA requiring eCPR. These data underline the potential toxicity of high dose of oxygen and suggest that control of oxygen administration in such patients is an important part of the treatment. A value of PaO2 between 100 and 300 mmHg after starting eCPR seems to be a target during treatment of OHCA treated by eCPR.

### F-31 Validation of qSOFA score in the Emergency Department (ED): a prospective study

#### Lafon Thomas^1^, Vallejo Christine^1^, Zmiri Camilia^1^, Organista Alexandre^1^, Coroller Louis^1^, Baisse Arthur^1^, Herafa Isabelle^1^, Vignon Philippe^1^, François Bruno^1^

##### ^1^CHU Dupuytren, Limoges, France

###### **Correspondence:** Lafon Thomas - thomas.lafon@chu-limoges.fr

*Annals of Intensive Care* 2018, **8(Suppl 1):**F-31

**Introduction:** Sepsis has been defined as a dysregulated host response to infection leading to life-threatening organ dysfunction (Singer M et al., JAMA 2016). A qSOFA score relying on 3 simple clinical criteria (respiratory rate, mental status and systolic blood pressure) has been proposed to better identify septic patients with associated higher mortality outside the intensive care unit (Seymour CW et al., JAMA 2016). The study aim was to evaluate the ability of qSOFA to predict the development of organ failure and increased 28-day mortality in patients admitted for suspected sepsis in the Emergency Department (ED).

**Patients and methods:** Prospective study conducted over a period of 6 months comparing the prevalence of organ failure and 28-day mortality according to the value of qSOFA at admission to the ED between group A (qSOFA > = 2) and group B (qSOFA < 2). As part of routine care, an electronic sepsis form was specifically created to identify prospectively and exhaustively all eligible patients on-line. For the purpose of the study, sepsis diagnosis was independently validated off-line by an adjudication committee which included three physicians who reviewed clinical, biological and microbiological data. For each patient, demographic data, source of infection, qSOFA and SOFA score, biological data and 28-day mortality were recorded.

**Results:** From November 2016 to April 2017, of the 476 patients identified, 374 patients were included after adjudication and 77 patients (20.5%) had a positive qSOFA score and constituted group A (46 men + median age = 77 years [23–95]). Tachypnea, confusion and low blood pressure were noted respectively in 82, 71 and 70% of group A patients. In group A, organ failure occurred in 64 patients (83.1%), 30 of them exhibiting septic shock (39%). Median SOFA score was 4 [2–12] and median lactate level at baseline was 3.31 [0.5–27.13]. Seventy-six patients of group A (99%) were hospitalized, of whom 25 were admitted to the intensive care unit (32.5%), and 28-day mortality reached 46.7%. In group B, only 32 patients developed an organ failure (10.7%) and 28-day mortality was 4.3% (Table 1).

**Table 1:** Comparison of qSOFA groups

	Group A qOFA ≥ 2 points (n = 77)	Group B qSOFA < 2 points (n = 297)	*p*
Organ failure	64 (83.1)	32 (10.7)	< 0.01
ICU admission	25 (32.4)	13 (4.3)	= 0.05
Death on day 28	36 (46.7)	13 (4.3)	< 0.01

**Conclusion:** The present study confirmed that the qSOFA score is a reliable and practical tool to predict the development of organ failure and higher 28-day mortality in patients with suspected sepsis in the ED.

### F-32 Limits of CT scan criteria and intravascular contrast extravasation to define pelvic angioembolization need: a specific assessment on the risk of false-positive

#### Ramin Séverin^1^, Charbit Jonathan^1^, Hermida Margaux^1^, Deras Pauline^1^, Capdevila Xavier^1^

##### ^1^CHU de Montpellier, France

###### **Correspondence:** Ramin Séverin - severin.ramin@gmail.com

*Annals of Intensive Care* 2018, **8(Suppl 1):**F-32

**Introduction:** Intravascular contrast extravasation (ICE) on CT imaging is commonly considered as the marker of pelvic active arterial bleeding, which justifies immediate performing of an pelvic angiography and trans-arterial angioembolization (TAE). However, ICEs lack of specificity in detecting arterial injuries especially in cases of bones or venous bleeding. The objective of present study was to determine the predictive performance of different ICEs characteristics for the need for Trans-arterial embolization (TAE) and the risk factors for false positive for arterial bleeding.

**Patients and methods:** A retrospective study was performed in our trauma center between 2010 and 2015. All severe trauma patients with pelvic fracture were included. Pelvic ICE characteristics on CT scan were specifically studied—arterial (aS2ICE), portal surface (pS2ICE) and extension (exS2ICE), anatomical relationships (ICE contact with complex bone fractures, direct relation between ICE and large retroperitoneal Hematoma. The overall predictive performance of ICE surfaces for pelvic TAE use was analyzed thanks ROC curves. The analysis then focused on the study of risk factors for false positives.

**Results:** Among 311 severe trauma patients with pelvic ring fracture (mean age 42 ± 19, mean ISS 27 ± 19), 94 (30%) had at least one pelvic ICE on initial CT scan. Patients requiring pelvic TAE had aS2ICE and pS2ICE significantly larger than others (p = 0.001 and p = 0.035, respectively). The global ability of ICE surfaces was either modest (aS2ICE AUC 0.76 [CI 95% 0.64–0.90] + p = 0.011) or non-significant (pS2ICE and exS2ICE) to predict pelvic TAE. The high sensitivity threshold was defined as an aS2ICE greater than 20 mm^2^. Using this threshold, 76% of patients were false-positives. Risk factors of false-positives were—admission low systolic blood pressure (OR 6.7 [CI 95% 1.2–36.7]) and low transfusion needs (OR 15.0 [CI 95% 1.7–133.6]), the extravasation in contact with complex bone fracture (OR 7.8 [CI 95% 1.4–59.7]) or the absence of direct relation between the extravasation and large retroperitoneal hematoma (OR 7.6 [CI 95% 1.4–42.3]).
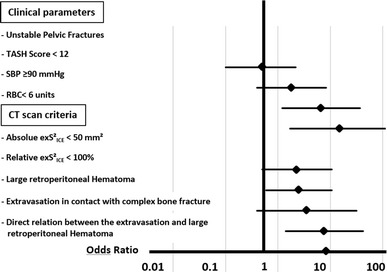


**Conclusion:** A significant pelvic ICE during arterial phase does not guarantee the need for pelvic TAE. Three-quarter of patients with an aS2ICE greater than 20 mm^2^ did not experienced pelvic TAE. TAE may be performed in cases of direct relation between ICE and large retroperitoneal hematoma or an ICE without contact with complex bone fracture.

### F-33 Role of mitochondrial Cyclophilin-D in cardiac resuscitability and survival after asphyxial cardiac arrest in mice

#### Jahandiez Vincent^1^, Cour Martin^1^, Pillot Bruno^2^, Gallo-Bona Noelle^2^, Ovize Michel^2^, Argaud Laurent^1^

##### ^1^Hôpital Edopuard Herriot, Lyon, France; ^2^INSERM-U1060, Lyon, France

###### **Correspondence:** Jahandiez Vincent - vincent.jahandiez01@chu-lyon.fr

*Annals of Intensive Care* 2018, **8(Suppl 1):**F-33

**Introduction:** Opening of the mitochondrial permeability transition pore (PTP), triggered by Cyclophilin-D (CypD) binding under stress conditions, plays a key role in ischemia–reperfusion injury. We sought to determine, using transgenic mice, whether CypD deletion (CypD −) would improve resuscitability and survival after experimental cardiac arrest (CA). Additionally, we compared the protective effects of CypD deficiency with that of targeted temperature management (TTM).

**Patients and methods:** Anesthetized mice underwent a 5 min asphyxial CA followed by resuscitation (cardiac massage, resumption of ventilation, epinephrine). Four groups of animals were studied—Sham, Control (Ctrl), CypD-CA using mice lacking CypD (knockout mice), and TTM-CA with fast hypothermia induced by external cooling at reperfusion (33 °C for 1 h). Two hours after CA, the following measurements were carried out (n = 6–9 group)—echocardiography, cellular damage markers (including S100B protein and troponin Ic) and mPTP opening in mitochondria isolated from brain and heart. Additional mice (n = 7–12 group) were included in the same 4 groups for survival follow-up (24 h and 7 days).

**Results:** Characteristics of CA were similar among groups. Rate of restoration of spontaneous circulation (ROSC) was significantly higher in CypD—and TTM groups compared to controls (p < 0.05). Time to ROSC was shorter in CypD—versus TTM and Ctrl (p < 0.05). Genetic loss of CypD and TTM prevented to a similar extent CA-induced myocardial dysfunction, increase in blood levels of both S100B protein and troponin Ic (p < 0.05 versus Ctrl). CA resulted in a significant increase in PTP opening only in mitochondria isolated from brain (p < 0.05 versus Sham). CypD deletion as well as TTM limited CA-induced PTP opening in brain (p < 0.05 versus Ctrl). Short-term survival (24 h) was significantly improved in the CypD—and TTM groups when compared to controls (p < 0.05). However, only therapeutic hypothermia improved survival at day 7 (p < 0.05 versus Ctrl).

**Conclusion:** In our murine CA model, genetic loss of CypD increased resuscitability and short-term survival but, unlike therapeutic hypothermia, failed to improve 7-day survival.

### F-34 Predicting coma outcome using resting-state fMRI and machine learning

#### Pugin Deborah^1^, Hofmeister Jeremy^1^, Van de Ville Dimitri^1^, Gasche Yvan^1^, Vulliemoz Serge^1^, Haller Sven^1^

##### ^1^HUG de Genève, Switzerland

###### **Correspondence:** Pugin Deborah - Deborah.pugin@hcuge.ch

*Annals of Intensive Care* 2018, **8(Suppl 1):**F-34

**Introduction:** Early prediction of neurological outcome of post-anoxic comatose patients after cardiac arrest (CA) is challenging. Prognosis of comatose patient relies on multimodal testing—clinical examination, electrophysiological testing and structural neuroimaging (mainly diffusion MRI). This prognostication is accurate for predicting poor outcome (i.e. death) but not sensitive for identifying patients with good outcome (i.e. consciousness recovery). Resting state functional MRI (rs-fMRI) is a powerful tool for mapping functional connectivity, especially in patients with low collaboration. Several studies showed that rs-fMRI can differentiate states of consciousness in chronically brain-damaged patients. A recent study also showed that functional neuroimaging can early detect signs of consciousness in patient with acute traumatic brain injury. However, rs-fMRI has not been assessed for the early prognostication of post-anoxic comatose patient.

**Patients and methods:** We assessed whole-brain function connectivity (FC) of 17 post-anoxic comatose patients early after CA using rs-fMRI. Nine patients ultimately recovered consciousness (good outcome) while eight died (poor outcome). We estimated FC for each patient following a procedure previously described. We statistically compared whole-brain FC between good and poor outcome group, to assess which brain connections differed between them. Then, we trained a machine-learning classifier (a Support Vector Machine, SVM) to automatically predict coma outcome (good poor) based on whole-brain FC of comatose patients. Finally, we compared this outcome prognostication based on functional MRI to those using standard structural diffusion MRI.

**Results:** Good and poor coma outcome groups were similar in terms of demographics, except for time to ROSC. Good outcome group showed significant increase in whole-brain FC between most cortical brain regions + with the strongest changes occurring within and between occipital and parietal, temporal and frontal regions (Fig. 1). Using whole-brain FC and a SVM classifier to predict coma outcome yielded to an overall prediction accuracy of 94.4%(AUC 0.94). Interestingly, automatic outcome prognostication using functional neuroimaging achieved better results that structural neuroimaging methods like DWI (accuracy 70.6%).

**Fig. 1:**
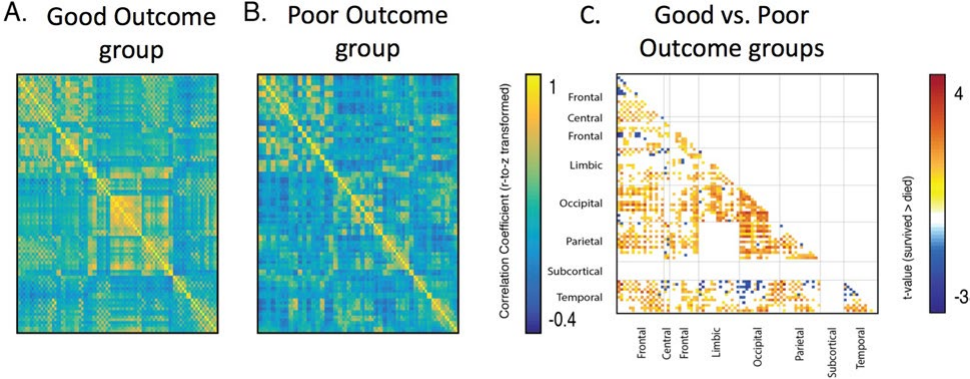
Outcomes of post-anoxic comatose patients early after CA, using rs-fMRI

**Conclusion:** We used rs-fMRI to predict coma outcome in a cohort of post-anoxic comatose patients early after CA. We deliberately chose to include only patients with indeterminate prognosis after standard multimodal testing, to assess the contribution of rs-fMRI in the early prognostication of coma outcome. We found that automatic prediction based on functional neuroimaging yielded much better results than current DWI methods, notably for identifying patients who recovered consciousness.

### F-35 Neurological Pupil index for early neuroprognostication

#### Miroz John-Paul^1^, Solari Daria^1^, Eckert Philippe^1^, Oddo Mauro^1^, Ben-Hamouda Nawfel^1^

##### ^1^Centre Hospitalier Universitaire Vaudois (CHUV), Lausanne, Switzerland

###### **Correspondence:** Miroz John-Paul - John-Paul.Miroz@chuv.ch

*Annals of Intensive Care* 2018, **8(Suppl 1):**F-35

**Introduction:** ECMO is increasingly used in critical care. Neurologic complications are frequent however there are no quantitative tools for outcome prediction in this setting. The aim of the study is to examine the value of quantitative pupillometry to predict neurologic outcome in patients on veno-arterial ECMO (VA-ECMO).

**Patients and methods:** Preliminary cohort analysis (June 2016-August 2017) of consecutive patients with VA-ECMO, in whom Npi^®^-200 pupillometer (Neuroptics^®^, USA) was repeatedly performed (12–72 h) to measure  % pupillary light response (qPLR) and Neurologic Pupil index (NPi). Mortality was assessed at 3 months. Using simplified 4-channel EEG (Masimo SedLine^®^, USA) depth of sedation (using PSI—Patient State Index and SR— % suppression ratio on EEG) was recorded in parallel.

**Results:** A total of 28 patients (mean age 56 years [23–81] + mean APACHE II 28 [12–47], mean SOFA 14 [9–18]) were analyzed—14 patients (50%) had refractory cardiogenic shock and 14 had refractory cardiac arrest. Mortality at 3 months was 78%. NPi < 3 and qPLR < 11% were associated with 100% mortality. NPi = 0 (n = 7) was associated with highly suppressed EEG (SR 64–100%) and early death within 96 h. Among survivors (n = 5) NPi was > 4 on all measurements.

**Conclusion:** Automated infrared pupillometry, and particularly the quantitative Neurological Pupil index, may be useful for early neuroprognostication in VA-ECMO patients. Further study is undergoing at our center to confirm these preliminary data in a larger cohort.

### F-36 Elaboration of a consensual endpoint to evaluate antimicrobial treatment efficacy in future HAP VAP clinical trials

#### Weiss Emmanuel^1^, Ewig Santiago^2^, Zahar Jean-Ralph^1^, Adler Jeff^3^, Asehnoune Karim^1^, Bassetti Matteo^4^, Bonten Marc^5^, Chastre Jean^1^, De Waele Jan^6^, Dimopoulos George^7^, Eggimann^8^, Philippe, Engelhardt Marc^8^, Kollef Marin^3^, Lipman Jeffrey^9^, Luna Carlos^10^, Martin Loeches Ignacio^11^, Pagani Leonardo^4^, Palmer Lucy^3^, Papazian Laurent^1^, Poulakou Garyphallia^7^, Prokocimer Philippe^3^, Rello Jordi^12^, Rex John^12^

##### ^1^Hôpital Beaujon, Clichy, France; ^2^Hôpital de Bonn, Germany; ^3^Hôpital Saint Joseph, Ohio, USA; ^4^Hôpital Santa Maria Misericordia, Udine, Italy; ^5^Hôpital de Utrecht, Netherlands; ^6^Hôpital de Ghent, Belgium; ^7^Université d’Athènes, Grèece; ^8^CHUV Lausanne, Switzerland; ^9^Université du Queensland, Australia; ^10^Université de Buenos Aires, Argentina; ^11^HôpitaI Saint James, Dublin, Ireland; ^12^Hôpital Vall d’Hebron, Barcelone, Spain

###### **Correspondence:** Weiss Emmanuel - manuweiss@yahoo.fr

*Annals of Intensive Care* 2018, **8(Suppl 1):**F-36

**Introduction:** Hospital-acquired (HAP) and ventilator-associated pneumonia (VAP) are often selected for randomized clinical trials (RCTs) aiming at new drug approval. A recent systematic review (Weiss et al. Crit Care 2017) reported a significant heterogeneity in endpoints used in RCTs comparing treatment of severe pneumonia that may influence their ability to demonstrate differences between studied drugs. Clinical cure was the most frequently used endpoint but its definition was highly variable. These results are not surprising as far as even guidance from regulatory agencies on how to evaluate HAP VAP treatments differ. The aim of this work was to reach a consensus on the most appropriate endpoint to consider in future clinical trials evaluating the efficacy of antimicrobial treatment for HAP VAP, using Delphi method.

**Patients and methods:** Twenty-six international experts from intensive care, infectious disease and from the industry were consulted using Delphi method (four successive questionnaires) from January 2016 to January 2017. More than 70% of similar answers to a question were necessary to reach a consensus.

**Results:** According to 60% the experts, clinical cure was the most desirable primary outcome among those found in the literature but two other endpoints were highly rated—all-cause mortality and mechanical ventilation (MV)-free days. Consequently, 88% of the panelists agreed to use a composite endpoints and even a hierarchical composite endpoint to combine these items together in which clinical cure and MV-free days would be assessed at day 28 and clinical cure at day 7 after end of therapy. For VAP, mortality was considered as the most clinically significant item by 75% of the experts, followed by MV-free days and finally clinical cure (Fig. 1). For HAP, a dual composite endpoint that only included all-cause mortality and clinical cure was chosen (Fig. 1). Among the various elements of clinical cure definition found in the literature, only three were retained by the experts—resolution at end of therapy of signs and symptoms present at enrolment, no further antimicrobial treatment needed and resolution or lack of progression of radiological signs of pneumonia. Finally, we found a consensus on the signs and symptoms that should trigger the suspicion of pneumonia—worsening of gaz exchange, purulent tracheal secretions, hypotension and or vasopressor requirements and fever or hypothermia.

**Fig. 1:**
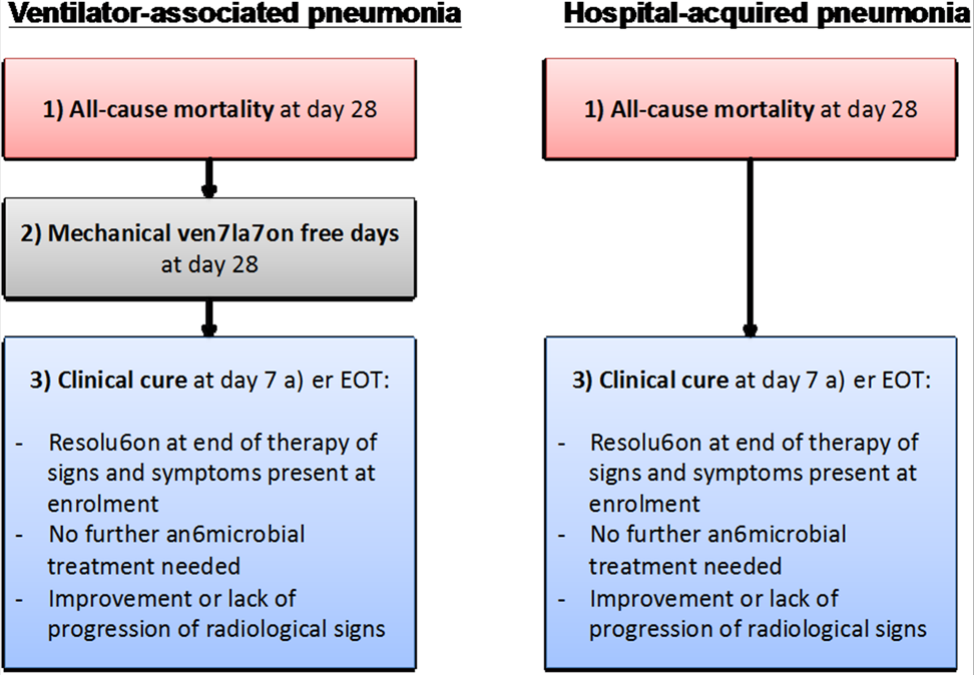
Clinically significant endpoints of hospital-acquired (HAP) and ventilator-associated pneumonia (VAP)

**Conclusion:** We provide here two consensual endpoints (for VAP and HAP) that would help addressing the efficacy of antimicrobial molecules for HAP VAP treatment in future clinical trials.

### F-37 Ventilator associated pneumonia due to Stenotrophomonas maltophilia: risk factors and outcome

#### Merceron Sybille^1^, Ibn Saied Wafa^1^, Schwebel Carole^1^, Le Monnier Alban^1^, Garrouste-Orgeas Maité^1^, Marcotte Guillaume^1^, Ruckly Stéphane^1^, Souweine Bertrand^1^, Darmon Michael^1^, De Montmollin Etienne^1^, Mourvillier Bruno^1^, Reignier Jean^1^, Papazian Laurent^1^, Siami Shidasp^1^, Azoulay Elie^1^, Bédos Jean-Pierre^1^, Timsit Jean-François^1^

##### ^1^Hôpital Le Chesnay, Versailles, France

###### **Correspondence:** Merceron Sybille - sybille07@gmail.com

*Annals of Intensive Care* 2018, **8(Suppl 1):**F-37

**Introduction:** Stenotrophomonas maltophilia (SM) is a Gram negative bacteria rarely involved in ICU acquired infections. Empirical treatment is challenging, as SM is resistant for almost all clinically-relevant antibiotics. SM ventilator-associated pneumonia (SM-VAP) have been associated with frequent inadequate antimicrobial therapy and increased ICU stay and mortality. Identifying risk factors for SM-VAP could be useful.

**Patients and methods:** We conducted a retrospective analysis of prospectively collected data in a multicenter database in France. VAP was determined as microbiologically documented pneumonia occurring after at least two days of mechanical ventilation. All patients admitted from 2000 to 2015 presenting at least one episode of VAP were included. SM-VAP were compared with VAP due to other micro-organisms (VAP-other). To assess risk factors and day-30 (D30) mortality, SM-VAP were matched (1 − n) with VAP-other based on center, time from ICU admission to VAP occurrence, and SAPS II score.

**Results:** Of the 20 968 patients included, 1 659 presented at least one episode of VAP. Among them, 102 (6.2%) suffered from SM-VAP. Patients with SM-VAP were men (61.3%), aged 62 [53–76] with SAPS II score 50 [40–65] and 20% were immunocompromised. Median length of stay in ICU was longer in SM-VAP (Table). 93 SM-VAP were matched with 380 control patients. In univariate analysis, risk factors for SM-VAP were—male gender, chronic heart failure, respiratory, cardiovascular and coagulation SOFA scores two days before VAP, median number of antibiotics used, percentage of time with antibiotics before VAP, parenteral nutrition, dialysis, catecholamine use and exposure to Ureido-carboxypenicillin, Ciprofloxacin, Tazobactam or Imipenem-Meropenem during the week before VAP (Table). Patients with SM-VAP were less likely to receive initial adequate therapy (56 vs 70%, OR 0.53, p = 0.01). There was no statistical difference for ICU or D30 mortality. D60 mortality was higher for SM-VAP (Table). In multivariate analysis, exposure to Imipenem-Meropenem during the week before VAP, respiratory and coagulation SOFA scores two days before VAP were independent risk factors for SM-VAP.

- **SAPSII**: Simplified Acute Physiology Score; **SOFA**: SOFA (Sequential Organ Failure Assessment); **SOFA Resp**: SOFA respiratory score;
- **SOFA coag**: SOFA coagulation score; **SOFA cardio: SOFA** cardiovascular score

**Table:**
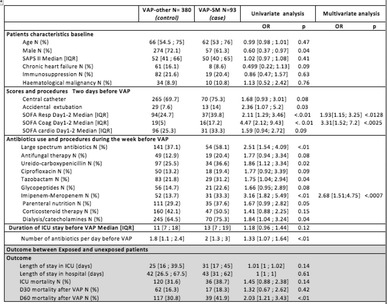

**Conclusion:** SM-VAP represented 6.2% of VAP. We observed no differences in patients characteristics between the groups. Imipenem-Meropenem use during the week before VAP was the most important risk factor for SM-VAP. The higher risk of inadequate initial therapy with SM-VAP had no impact on D30 mortality but D60 mortality was significantly higher.

### F-38 Personal feedback with ultraviolet inspection cabinets for undergraduate students education to hand hygiene and nosocomial infection prevention

#### Lehingue Samuel^1^, Forel Jean-Marie^1^, Cassir Nadim^1^, Papazian Laurent^1^

##### ^1^Hôpitaux de Marseille, Marseille, France

###### **Correspondence:** Lehingue Samuel - samlehingue@gmail.com

*Annals of Intensive Care* 2018, **8(Suppl 1):**F-38

**Introduction:** Education of undergraduate students is key to improve Hand hygiene (HH) behavioral changes amongst doctors [1.2]. Our aim was to evaluate personal feedback using Ultraviolet (UV) light inspection cabinets in a 2 years program. Our hypothesis was that its use for Alcohol hand rub (AHR) application on first year would increase complete AHR application on 2nd year.

**Patients and methods:** This was a simple blind randomized trial comparing HH training with personal feedback using UV cabinet to a control group. On first year, students had access to a theoretical formation then were convened by groups for a demonstration of the correct execution of world health organization’s (WHO) procedure [3]. Before HH training, each group underwent a cluster randomization. In the control group, the student hand rubbed under visual supervision and advises of a trainer. In the intervention group after the same visual assessment, completeness of AHR hand application was recorded under UV light and shown to the student. He was given free access to the UV cabinet to repeat the technique, until perfect application complete under UV light. An enhancement with a scenario-based learning was proposed to both groups. On second year, every student were asked to hand rub with the fluorescent AHR. A supervisor blinded to the group of randomization assessed the quality of the HH procedure visually, the completeness of hand application under UV light and compliance with the WHO’s opportunities for HH during the simulation.

**Results:** After randomization 140 students were included in the intervention group and 102 in the control group. On second year, the rate of complete application of the AHR under UV was increased in the intervention group as compared with the control group (60% versus 30.4% p < 0.001) (Fig. 1) despite that visual assessment of HH procedures was similar between the two groups. In a logistic regression model including gender, intercurrent HH formation, intercurrent UV cabinet use, surgical unit traineeship and report of regular use of AHR, the hazard ratio for the intervention was 3.837 (IC 2.086–7.058). The rate of perfect compliance with the HH opportunities in the intervention group was increased (58.1% versus 42.4% p < 0.018) and the effect persisted in the logistic regression.

**Fig. 1:**
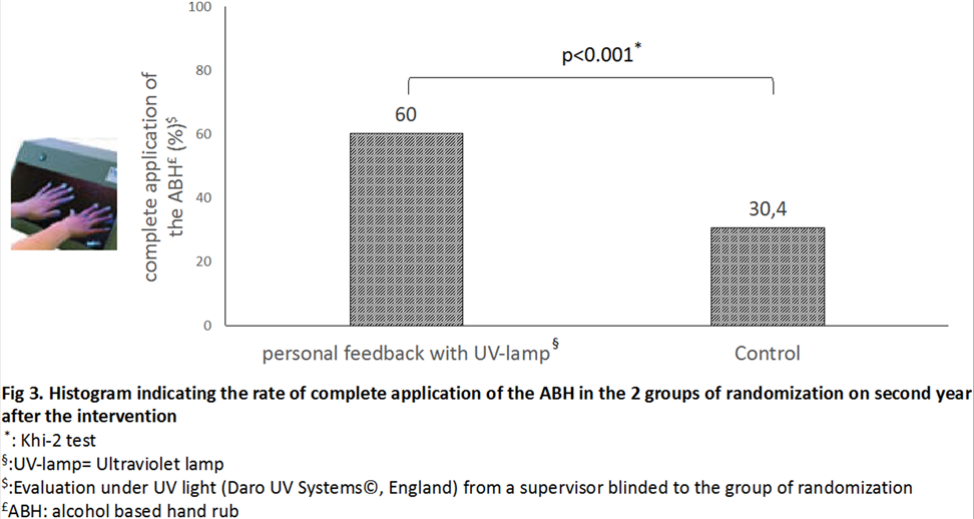
Histogram indicating the complete application of the ABH in the 2 groups

**Conclusion:** UV cabinets for undergraduate students’ HH education improve the technique and the compliance with HH opportunities. Included in a multifaceted education program, it must be considered a key tool for training.

### F-39 Bacterial colonization of healthcare workers’ mobile phones in an intensive care unit (ICU) effects of disinfection

#### Missri Louai^1^, Smiljkovski Daniel^1^, Prigent Gwénolé^2^, Lesenne Aude^2^, Obadia Thomas^3^, Joumaa Mohsen^1^, Chelha Riad^1^, Chalumeau-Lemoine Ludivine^1^, Obadia Edouard^1^, Galbois Arnaud^1^

##### ^1^Hôpital Privé Claude Galien, Quincy-Sous-Sénart, France; ^2^Cerballiance Paris sud, laboratoire de Bactériologie, Wissous, France; ^3^Institut Pasteur, Paris, France

###### **Correspondence:** Missri Louai - louai.missri@gmail.com

*Annals of Intensive Care* 2018, **8(Suppl 1):**F-39

**Introduction:** Bacterial colonization of environmental surfaces or medical devices can lead to nosocomial infections, especially in ICU. Mobile phones are commonly used by healthcare workers. However, very few studies assessed bacterial colonization of healthcare workers’ mobile phones in ICU and the impact of disinfection.

**Patients and methods:** We conducted a prospective monocentric study in a 15-bed medical ICU with 54 healthcare workers. Swabs were used to assess bacterial colonization of healthcare workers’ mobile phones before and after 5 min of disinfection with bactericidal Anios© wipes. Results were compared to those observed in administrative staff. Swabs were incubated at 37 °C for 24 h on different types of agar. Bacterial identification was conducted by mass spectrometry (MALDI-TOF) combined with the VITEK2 automated antibiotic device for the search for antibiotic-resistant strains.

**Results:** We report in this abstract preliminary results after inclusion of 47 healthcare workers in ICU and 27 in administrative staff. All healthcare workers participating in the study reported keeping their mobile phone during their shift. Only 5 47 (10.6%) declared to wash it monthly. All of the ICU’s mobile phones were colonized by bacterial skin flora, 53.2% by environmental flora and 43% were colonized by pathogens (coagulase negative staphylococcus excluded). Only one MRSA was identified and no ESBL was detected. Results were similar in administrative staff’ mobile phones (Table). No variable (healthcare workers, profession, mobile phone brand, use of cover case, routine disinfection…) was associated with pathogens colonization. Following disinfection, bacterial colonization decreased—105 (20–315) UFC mL vs. 1000 (295–1000) UFC mL (p < 0.001). After disinfection, only 5 (10.6%) mobile phones were sterilized but 75% (15 20) of pathogens colonized mobile phones became pathogen-free.

**Table:** 

Variable	ICU healthcare workers’ mobile phones (n = 47)	Administrative staff’ mobile phones (n = 27)	*p* value
Bacterial colonization, n (%)	47 (100)	27 (100)	1
Skin flora	47 (100)	27 (100)	1
Oropharyngeal flora	4 (8.5)	2 (7.4)	1
Digestive flora	3 (6.4)	2 (7.4)	1
Environmental flora	25 (53.2)	17 (63.0)	0.47
Pathogens colonization (CNS excluded), n (%)	20 (42.6)	11 (40.7)	1
*Staphylococcus aureus*	9 (19.1)	5 (18.5)	1
*Digestive flora*	4 (8.5)	2 (7.4)	1
*K. oxytoca*	1 (2.1)	0	
*E. cloacae*	1 (2.1)	1 (3.7)	
*Leclercia*	0	1 (3.7)	
*E. fecalis*	1 (2.1)	0	
*E. faecium*	1 (2.1)	0	
*Oropharyngeal flora*	5 (10.6)	2 (7.4)	1
*Moraxella* sp.	1 (2.1)	1 (3.7)	
*Raoultella ornithinolytica*	1 (2.1)	0	
*Haemophilus parainfluenzae*	1 (2.1)	0	
*Rothia*	1 (2.1)	0	
*Streptococcus salivarius*	1 (2.1)	0	
*Aerococcus viridans*	0	1 (3.7)	
*Bacillus cereus*	6 (12.8)	3 (11.1)	1
MRSA, n (%)	1 (2.1)	0	1
ESBL, n (%)	0	0	

**Conclusion:** Colonization of mobile phones is similar in healthcare workers in ICU and in administrative staff. In our ICU, all mobile phones are colonized by skin flora and 43% with pathogens. Our assessed disinfection protocol appeared insufficient. Intensivists should be aware of these results to help prevent nosocomial infections.

### F-40 Absence of extended-spectrum beta-lactamases-producing Enterobacteriaceae fecal carriage cross-transmission in a medical ICU

#### Prevel Renaud^1^, Cockenpot Thibault^1^, M’Zali Fatima^1^, Andre Catherine^1^, Boyer Alexandre^1^, Dubois Veronique^1^, Gruson Didier^1^

##### ^1^Hôpital Pellegrin, Bordeaux, France

###### **Correspondence:** Prevel Renaud - renaud.prevel@hotmail.fr

*Annals of Intensive Care* 2018, **8(Suppl 1):**F-40

**Introduction:** Extended-spectrum beta-lactamases-producing Enterobacteriaceae (ESBL-E) are disseminating worldwide. ESBL-E fecal carriage is known to increase the risk of ESBL-E infections and so untailored empirical probabilistic antibiotherapy and mortality. The aim of this work was to study the mechanisms of dissemination of ESBL-E fecal carriage in an intensive care unit (ICU).

**Patients and methods:** We screened every in-patient at admission and every 7 days in a two units of medical ICU (25 beds) for 6 months according to current practice. Every ESBL-E found was characterized by ESBL genes PCR amplification and sequencing and the clonal dissemination was assessed by Pulsed-Field Gel Electrophoresis (PFGE).

**Results:** Over that 6-month period, among the 608 screened patients, 57 (9.4%) were found to be colonized by ESBL-E. Among these 57 patients, 7 (1.2%) acquired the ESBL-E fecal carriage during their stay in the ICU. No cross-transmission of ESBL-E occurred (as assessed by PFGE analysis) even for ICU-acquired fecal carriage. ESBL genes were found to be mostly from the CTX-M group mainly CTX-M group 1 (36 57) enhancing the community origin of the ESBL-E fecal carriage even in ICU. ESBL-E caused infections occurred in 5 57 patients always consisting in ventilator-associated pneumonia caused by the colonizing strain.

**Conclusion:** Hygiene isolation procedures seem to be well applied in our units as we do not have any ESBL-E fecal carriage cross transmission case. But still cases of ICU-acquired ESBL-E fecal carriage occurred suggesting an endogenous origin in a non-outbreak situation. Therefore, the mechanisms of ESBL-E expansion in the community and of endogenous ESBL-E acquisition in ICU are still to investigate.

### F-41 Bloodstream infections in adults undergoing extracorporeal membrane oxygenation: epidemiology and risk factors

#### Perrin Caroline^1^, Conil Jean-Marie^1^, Georges Bernard^1^, Seguin Thierry^1^, Crognier Laure^1^, Fourcade Olivier^1^, Bounes Fanny^1^, Minville Vincent^1^, Silva Stein^1^, Delmas Clément^1^

##### ^1^CHU de Toulouse, France

###### **Correspondence:** Perrin Caroline - perrincaroline@hotmail.fr

*Annals of Intensive Care* 2018, **8(Suppl 1):**F-41

**Introduction:** Detection of nosocomial infections (NIs) occurring during extracorporeal membrane oxygenation (ECMO) is difficult and their related side effects appear to contribute to the high mortality rates (40–50%) of these patients. The most common ones are ventilator-associated pneumonia and bloodstream infections (BSI). Thus we empirically developed a standardized protocol with systematic blood cultures on days 2, 6, 10 and 15. The objectives of this study were to analyze BSI incidence, distribution of microorganisms, risk factors in adult undergoing ECMO support and to evaluate the efficiency of our protocol.

**Patients and methods:** We prospectively included all adult patients who underwent veinoarterial (VA-ECMO) or veinovenous extracorporeal membrane oxygenation (VV-ECMO) between May 2014 and March 2016. Anamnestic data and blood culture results using the protocol and additional blood samples were analyzed.

**Results:** Among the 81 patients who underwent ECMO support for more than 48 h, 10 patients in the VA-ECMO group (n = 10 56 + 17.9%) and 3 patients in the VV-ECMO group (n = 3 25 + 12%) developed 18 BSI, corresponding to a global rate of 22.4 BSI per 1000 ECMO days. Microorganisms associated with these infections were most frequently gram-negative bacilli. Only the duration of ECMO was significantly correlated with BSI occurrence, with a median duration of 6.5 days for “patients without BSI” and of 14 days for “patients with BSI” (p = 0.0170). The efficiency of the protocol in diagnosing BSI was roughly 5% (5 96) for the VA-ECMO group and around 6% (3 49) for the VV-ECMO group.

**Conclusion:** Probability of developing BSI in adults undergoing ECMO increases with ECMO duration. Systematic blood cultures do not appear to be an effective way of improving BSI detection.

### F-42 Epidemiology of bacteriological analyses in adult patients supported by veinoarterial extracorporeal membrane oxygenation

#### Perrin Caroline^1^, Conil Jean-Marie^1^, Georges Bernard^1^, Marcheix Bertrand^1^, Seguin Thierry^1^, Ruiz Stéphanie^1^, Fourcade Olivier^1^, Bounes Fanny^1^, Minville Vincent^1^, Silva Stein^1^, Delmas Clément^1^

##### ^1^CHU de Toulouse, France

###### **Correspondence:** Perrin Caroline - perrin.ca@chu-toulouse.fr

*Annals of Intensive Care* 2018, **8(Suppl 1):**F-42

**Introduction:** Nosocomial infections (NIs) occurrence during veno-arterial extracorporeal membrane oxygenation (VA-ECMO) support and their complications appear to contribute to the high mortality rates (40–60%) of these patients. The most common ones are ventilator-associated pneumonia and bloodstream infections (BSI). In clinical practice, detection of NIs is difficult due to confounding factors caused by the device. The objectives of our study were to analyze BSI and positive tracheal aspirate (TA) culture incidences, distribution of microorganisms and risk factors in adult undergoing veinoarterial ECMO (VA-ECMO).

**Patients and methods:** We prospectively and consecutively included all adult patients who underwent VA-ECMO between May 2014 and March 2016 in our intensive care unit. Anamnestic data, blood culture and TA culture were analyzed. Primary endpoint was the occurrence of a BSI or a positive TA culture more than 24 h after ECMO initiation and within 48 h after ECMO discontinuation.

**Results:** Among the 56 patients who underwent ECMO support for more than 48 h, the BSI prevalence was 27.4 cases per 1000 ECMO days and microorganisms associated were most frequently gram-negative bacilli. As for positive TA cultures, microorganisms associated were oropharyngeal germs and gram-negative bacilli. Two risk factors were associated with nosocomial bacteria occurrence in TA cultures—prior antibiotics and duration of mechanical ventilation more than 5 days. We demonstrated a link between “positive TA culture” and “positive blood culture” and we showed a protective effect of using an antibioprophylaxis on “positive TA culture” and “global positive cultures” development.

**Table:** 

	No antibioprophylaxis	Antibioprophylaxis	p
Positive blood culture no/yes	29 (80.6%)/7 (19.4%)	16 (80%)/4 (20%)	0.9604
Positive tracheal aspiration culture no/yes	4 (11.1%)/32 (88.9%)	12 (60%)/8 (40%)	0.00037*
Positive tracheal aspiration culture (> 10^6^ UFC/ml) no/yes	19 (52.8%)/17 (47.2%)	17 (85%)/3 (15%)	0.02068*
Global positive culture no/yes	16 (44.4%)/20 (55.6%)	15 (75%)/5 (25%)	0.0484*

**Conclusion:** Use of an antibioprophylaxis at ECMO implantation is associated with a decrease of global positive cultures development.

### F-43 Management of status epilepticus in the intensive care unit: evaluation of the current pharmacological practice and risk of drug–drug interactions

#### Alexandre Destere^1^, Vallée Estelle^1^, Mouly Stéphane^1^, Linares-Lloret Celia^1^, Megarbane Bruno^1^

##### ^1^Hôpital Lariboisière, Paris-Diderot University, Paris, France

###### **Correspondence:** Alexandre Destere - alexandre.destere@aphp.fr

*Annals of Intensive Care* 2018, **8(Suppl 1):**F-43

**Introduction:** The majority of recommended drugs to treat status epilepticus (SE) patients have a limited safety margin with potential risk of drug–drug interactions. Cytochrome P450 (CYP) induction may result in insufficient dosage and subsequent therapeutic inefficiency of the patients’ co-treatments including the anticonvulsive drugs administered to treat the SE. Our objective was to describe the anticonvulsive drugs administered to SE patients and determine the resulting theoretical risk of drug–drug interactions with the patients’ treatments.

**Patients and methods:** Patients admitted during 1 year (Jan–Dec 2016) in our University Hospital intensive care unit (ICU) were selected using the Program of Medicalization of the Information system (PMSI). The usual demographic, clinical, and outcome data were collected with a special focus at the patients’ treatments taken before ICU admission and treatments administered during the ICU stay including the anticonvulsive drugs. We used the table of drug–drug interactions of the Geneva University Hospitals (1) and the DrugBank website (2) to determine the risks of drug–drug interactions.

**Results:** Sixty-one patients (74% males 26% females + age—56 years [16 + 92] (median [percentiles 25, 75]) were included in this study. Patients were chronic alcoholics (41%) and drug users (16%). The following anticonvulsive drugs were administered—levetiracetam (N = 46, 75%), fosphenytoine (N = 40, 66%), clonazepam (N = 37, 61%), clobazam (N = 28, 46%), midazolam (N = 22, 36%), valproic acid (N = 10, 16%), carbamazepine (N = 7, 12%) and phenobarbital (N = 7, 12%). Among the 46 61 patients (77%) treated with a CYP inducer, 26 patients (63%) received at least one drug with liver metabolism of which plasma concentrations might have been altered by such potential drug–drug interactions, thus representing 43% of the SE patients admitted to the ICU. Drugs at risk of pharmacokinetic alterations were cardiovascular, metabolic and psychotropic drugs.

**Conclusion:** Drugs with potential risk of drug–drug interactions used to treat SE patients in the ICU are relatively frequent. Such drug–drug interactions are at risk of altering the plasma concentrations of the anticonvulsive drugs as well as several other patients’ co-treatments, highlighting the usefulness of pharmaceutical advice at the bedside when choosing the anticonvulsive drugs to administer.

**References**

1. http://www.hug-1-ge.ch/sites/interhug/files/structures/pharmacologie_et_toxicologie_cliniques/a5_cytochromes_6_2.pdf
2. https://www.drugbank.ca

### F-44 Delirium in the intensive care unit (ICU): perception of healthcare professionals

#### Fahmi Dachraoui^1^, Hraiech Kmar^1^, Mariem Tlili^1^, Nouira Wiem^1^, Bouker Nouha^1^, Hend Zorgati^1^, Sana Boukadida^1^, Amal Ouni^1^, Hammouda Zaineb^1^, Islem Ouanes^1^, Lamia Ouanes Besbes^1^, Abroug Fekri^1^

##### ^1^CHU Fatouma Bourguiba, Monastir, Tunisia

###### **Correspondence:** Fahmi Dachraoui - dachraoui.fahmi@gmail.com

*Annals of Intensive Care* 2018, **8(Suppl 1):**F-44

**Introduction:** Delirium in the ICU is often under-diagnosed despite its related burden and impact on patients’ morbidity, mortality and prolongation of hospital length of stay. The aim of this study was to assess the medical and paramedical community beliefs and practices regarding delirium in Tunisian ICUs.

**Patients and methods:** Between August 1st and 31/2017, healthcare professionals working at the ICUs of university hospitals of Monastir and Mahdia (Tunisia) were asked to participate in the survey by completing a questionnaire anonymously (that specified participants’ characteristics (age, gender, function, years of experience in ICU) and their knowledge and perception of delirium in ICU. The questionnaire consisted in 10 questions of different types: Likert style (: widespread scale in psychometric questionnaires in which the respondent expresses his or her degree of agreement or disagreement with an assertion), multiple choice, ranking and yes/no).

**Results:** During the study period, 96 respondents out of 163 (60% female, nurses: 52%), aged between 20–30 years in 70%, responded to the questionnaire. Healthcare professionals experience in the ICU was < 1 year in 39.6%; 1–5 years in 32.3%, and > 5 years in 28.1%. Participants asserted that the “most characteristic signs of delirium” were: insomnia (25%); confusion (22%); agitation (21%) and aggressiveness (20%). Three-quarters of participants said they did not systematically search for signs of delirium in their patients. 33% thought that delirium was “an insignificant problem” or that “it was not a problem”. Only one and three participants respectively, said they attended a conference and read an article about delirium in ICU the last year. Half of the respondents felt that the most appropriate treatment for a patient with delirium was restraint. Nearly one-third of participants thought that delirium was an under-diagnosed entity and only 6% felt that it was associated with long-term neuropsychological deficits. Factors considered to be determinant in the occurrence of delirium were ARDS, shock, age, mechanical ventilation, postoperative status in 70, 66, 63, 55 and 17%, respectively.

**Conclusion:** Most Tunisian healthcare professionals consider delirium as a common, underdiagnosed, and serious problem in the ICU. Yet, few participants actually monitor this condition.

### F-45 The influence of sedation choice on the delirium occurrence in critically ill poisoned patients: a randomized controlled trial

#### Khzouri Takoua^1^, Mrad Aymen^1^, Foudhaili Nasreddine^1^, Barghouth Manel^1^, Fatnassi Meriem^1^, Bachrouch Mayssa^1^, Mahdhaoui Soumaya^1^, Ben Hamida Samia^1^, Brahmi Nozha^1^

##### ^1^Tunis, Tunisia

###### **Correspondence:** Khzouri Takoua - takoua_kh2@yahoo.fr

*Annals of Intensive Care* 2018, **8(Suppl 1):**F-45

**Introduction:** Delirium is a common manifestation of acute brain dysfunction in critically ill patients. It is associated with a healthcare cost increase, and extension of the hospital stay length. The present study aimed to explore influence of patient characteristics and analgesic-sedation on delirium incidence and to analyze its risk factors.

**Patients and methods:** It is a prospective single blind randomized controlled trial, started on the first July 2017 in a 12-bed toxicological Intensive Care Unit, including all mechanically ventilated patients requiring sedation who were admitted for acute poisoning. They were randomly divided into two groups G1 et G2 receiving respectevily Propofol-Remifentanil and Midazolam—Remifentanil. Delirium assessment scores were judged not adapted to our population and we retained the diagnosis of delirium on arguments inspired from diagnostic and statistical manual of mental disorders fourth edition (DSM-IV).

**Results:** Until the 15th September 2017, 35 patients were included, with 22 patients in G1 and 13 in G2. The two groups were comparable in terms of epidemiological characteristics. Delirium was developed in 9 patients (26%) (n = 5 in G1 and n = 4 in G2) with an average duration of 18 ± 13 h [6–48] with no difference between the 2 groups (13 ± 10 h for G1–24 ± 16 h for G2, p = 0.268). Compared to those without delirium, no differences were found in the patient characteristics among these two groups with regard to sex, age, psychiatric history and severity of illness (APACHE II, IGS II score) and even with regard to hypnotic choice (5 vs 4 p = 0.599). Delirium was associated to prolonged duration of mechanical ventilation (69 ± 73 h vs 39 ± 69 h, p = 0.280) and length of ICU stay (91.51 h vs 54.05 h, p = 0.132) without significant differences. Delirious patients had more hypotension (p = 0.008), and received more atropine (0.026). Multiple logistic regression analysis identified atropine (OR 2.333, 5%Cl 0.992–5.489, p = 0.026) as an independent risk factor for delirium.

**Conclusion:** The diagnosis and prevention of ICU delirium are subjects of multiple ongoing investigations. We carried out this study to detect the risk factors of delirium in order to prevent it. It is important to note that our results are influenced by the studied population and are only preliminary. We rely on the study pursuit and the sample enlargement to better inform us as well on risk factors as protective.

### F-46 Predictive factors for complications in patients with severe alcohol withdrawal syndrome (Delirium tremens) requiring large dosages of benzodiazepines in ICU: observational retrospective study

#### Mateos François^1^, Haler Renaud^1^, Letheulle Julien^1^, Fillatre Pierre^1^, Barbarot Nicolas^1^, Legay François^1^, Debarre Matthieu^1^, Lhommet Claire^1^, Godard Aurelie^1^, Bousser Jérôme^1^, Tadie Jean Marc^2^, Courte Anne^1^

##### ^1^Centre hospitalier de Saint Brieuc, Saint Brieuc, France; ^2^CHU de RENNES, Rennes, France

###### **Correspondence:** Mateos François - francois.mateos@gmail.com

*Annals of Intensive Care* 2018, **8(Suppl 1):**F-46

**Introduction:** Background: Severe alcohol withdrawal syndrome is a common cause of hospital admission. Delirium tremens is a potentially fatal complication of alcohol withdrawal. In severe delirium, very large dosages of benzodiazepines can be required despite well described side effects, such as coma and hypoxic cardiac arrest, although there is no recommendations for standardized treatments. Objective -The aim of this study was to describe outcomes and risk factors for complications in patients with severe alcohol withdrawal syndrome treated in intensive care unit with continous infusion of benzodiazepine (BZD).

**Patients and methods:** We retrospectively reviewed the medical records of all patients hospitalized for alcohol withdrawal syndrome between 2006 and 2016. Only those who received continous-infusion of BZD, associated with close clinical monitoring and the evaluation of RASS and Cushman scores, without systematic recourse to mechanical ventilation, were included.

**Results:** We studied 104 patients hospitalized in ICU for severe alcohol withdrawal syndrome. The mean age (SD) was 48.7 ± 8.7 years, mean ICU admission SAPS (simplified acute physiology score) II score was 20 ± 6.1. All of them have received continous infusion of midazolam, with a median maximum perfusion velocity of 8 mg h (interquartile range, (5, 12)). The median duration of treatement was 2 days (interquartile range, (1, 3)). Thirteen patients (12%) developed pneumonia, and or required intubation, and 8 (8%) have had seizures. No cardiac arrest and death was observed. ICU length of stay (LOS) was 3 days (2, 5) (median, interquartile range). Patients who requiried intubation and or developed pneumonia, received substantially more BZD (median total dose, 428 mg of midazolam vs. 147 mg in the non-complicated group + p < 0.001), and their ICU LOS was higher (median, 5 days vs. 3 days + p < 0.001). Endotracheal intubation and or development of pneumonia were associated with a higher maximum perfusion velocity of midazolam (> 8 mg h) (OR 18.83, IC 95% (2.11–168.15), p = 0.009). Previous episodes of delirium tremens before ICU admission were associated with higher complications such as mechanical ventilation and or pneumonia (OR 11.29, IC 95% (1.45–87.60), p = 0.02).

**Conclusion:** In severe delirium, very large dosages of benzodiazepines can be used without systematic mechanical ventilation with a low incidence of complications.

### F-47 Impact of daylight exposure on delirium in ICU patients receiving invasive mechanical ventilation—the Delirium and dayLIGHT exposure (D-LIGHT) study

#### Smonig Roland^1^, Magalhaes Eric^1^, Andremont Olivier^1^, Essardy Fatiha^1^, Mourvillier Bruno^1^, Lebut Jordane^1^, Dupuis Claire^1^, Neuville Mathilde^1^, Lermuzeaux Mathilde^1^, Bouadma Lila^1^, Timsit Jean-Francois^1^, Sonneville Romain^1^

##### ^1^Hôpital Bichat, Paris, France

###### **Correspondence:** Smonig Roland - r.smonig@gmail.com

*Annals of Intensive Care* 2018, **8(Suppl 1):**F-47

**Introduction:** Delirium is frequent in intensive care unit (ICU) patients and is associated with increased mortality, increased hospital stay, increased cost and long term cognitive impairment in survivors. Numerous pharmacological and non-pharmacological strategies have been investigated for delirium treatment without success. Therefore delirium prevention strategies are recommended by current critical care practice guidelines. Among the potentially modifiable risk factors for delirium, the impact of daylight exposure on delirium incidence and or duration has not been studied. The objective of this study was to investigate whether daylight exposition would reduce delirium burden in critically ill patients.

**Patients and methods:** We conducted a prospective study in a 27-bed medical intensive care unit (ICU) over a 1-year period (January 2016–January 2017). All consecutive adult patients receiving invasive mechanical ventilation (MV) for 2 days or more were eligible for the study. Patients were assigned to a room with windows allowing daylight exposure (“Light” group) or without window (“Dark” group), depending on bed availability. Delirium was evaluated with the Intensive Care Delirium Screening Checklist (ICDSC) for a maximum period of 28 days. Delirium was defined by a ICDSC score ≥ 4 for two consecutive days. Agitation was defined by a RASS > or = +2. The primary endpoint was cumulative incidence of delirium. Data are presented as median (interquartile range) or number (percentage).

**Results:** A total of 195 patients were included (age—50 [50 + 69] years, SAPS2—51 [36 + 64], SOFA score—9 [7 + 11], medical admission—69%). Of them, 110 patients were admitted to a “Light” group and 85 to a “Dark” group. Incidence of known risk factors for delirium was similar in the two groups. Delirium occurred in 65 (64%) patients in the “Light” group and in 55 (71%) patients in the “Dark” group (p = 0.28). The duration of delirium was 3 [1 + 7] days. Patients in the “Light” group received significantly less neuroleptics to treat agitation than patients in the “Dark” group (13 vs. 25%, p = 0.04). This protective association persisted after adjustment for confounders in multivariate analysis (Odds ratio = 0.40 + [0.17 + 0.90] + p = 0.03).
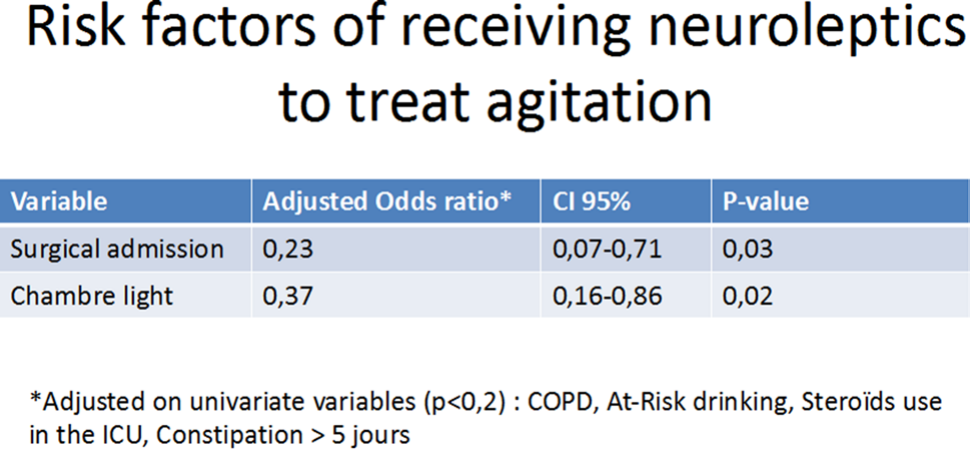


**Conclusion:** Daylight exposure does not impact on delirium burden in ICU mechanically ventilated patients. However, daylight exposure is independently associated with a reduced prescription of neuroleptics to treat agitation.

### F-48 Multicenter retrospective study of early in-ICU anesthetic drugs’ management in convulsive status epilepticus

#### Zeidan Sinéad^1^, Rohaut Benjamin^1^, Outin Hervé^2^, Bolgert Francis^1^, Navarro Vincent^1^, Demeret Sophie^1^

##### ^1^Pantin, France; ^2^CHI de Poissy, France

###### **Correspondence:** Zeidan Sinéad - sineadzeidan@gmail.com

*Annals of Intensive Care* 2018, **8(Suppl 1):**F-48

**Introduction:** Patients with convulsive status epilepticus (CSE) frequently require mechanical ventilation (MV), either for general anesthesia in case of refractory generalized CSE, or for airway protection. Guidelines for the management of refractory generalized CSE currently recommend general anesthesia for 24–48 h, followed by gradual withdrawal. Our objective is to evaluate the incidence of refractory generalized CSE among patients who required MV during pre-hospital management of status epilepticus, and to describe the management of general anesthesia in intensive care unit (ICU).

**Patients and methods:** This ongoing multicenter retrospective observational study is conducted in 4 French ICUs. All patients admitted in ICU under mechanical ventilation between 01-01-2014 and 12-31-2016 with disease-code “Status Epilepticus” are included. Exclusion criteria are—age < 18 years, post anoxic SE, acute traumatic brain injury, initiation of MV in ICU, transfer from another ICU, inclusion in a therapeutic trial on SE, non-convulsive SE. Collected data include reason for MV, antiepileptic treatment, dosage and duration of general anesthesia, mode of EEG monitoring. Outcomes are—relapse of SE, MV duration, in-ICU length of stay and mortality.

**Results:** Among the 211 medical files reviewed, 77 met the inclusion criteria and were analyzed, and 134 were excluded. A minority of patients (18.2%) had a refractory generalized CSE, most patients (64.9%) had a non-refractory generalized CSE + the others had mostly partial CSE. The main reason for intubation was coma (n = 45, 58.4%). The duration of general anesthesia was not significantly different in refractory CSE patients compared to non-refractory CSE patients (p = 0.18). Data regarding main outcomes are summarized below-.
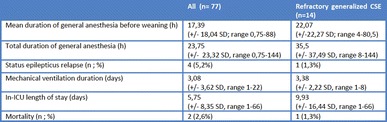


**Conclusion:** These preliminary data suggest that the majority of the patients admitted in ICU under MV for CSE do not have a refractory status. Indication of MV is mainly coma without persistent convulsions. The mean duration of general anesthesia before withdrawal is < 24 h, and thus in discrepancy with guidelines, but does not seem associated with a frequent relapse of SE. If this low rate of RSE for patients admitted in ICU and the safety of rapid withdrawal of GA are confirmed, the recommended 24–48 h duration of general anesthesia in ICU could be challenged.

### F-49 Brain imaging findings in patients with wake-up delay after induced coma

#### Nèji Henda^1^, Affes Mariem^1^, Attia Monia^1^, Marhbène Takoua^1^, Baccouche Ines^1^, Hantous-Zannad Saoussen^1^, Ben Miled-M’Rad Khaoula^1^

##### ^1^Hôpital Abderrahmen Mami, Ariana, Tunisia

###### **Correspondence:** Nèji Henda - hendaneji@gmail.com

*Annals of Intensive Care* 2018, **8(Suppl 1):**F-49

**Introduction:** Induced coma in intensive care patients protect them against pain and neurologic disorders. However, a few of them may present a delayed wake-up when the sedation is interrupted. The aim of this work is to assess brain imaging findings in patients with this condition.

**Patients and methods:** Retrospective review of imaging data of 27 patients (21 males and 6 females), aged between 32 and 84 years, admitted in intensive care unit (ICU) between June 2015 and September 2017, who had sedation or general anesthesia and presented a delayed wake-up. They were explored either by MRI (n = 11) or computed tomography (CT) (n = 16). Patients with traumatic lesions were excluded.

**Results:** Patients were admitted in the ICU because of chronic obstructive pulmonary disease exacerbation (n = 7), infectious pneumonia or pleural effusion (n = 6), acute respiratory failure (n = 5), heart disease (n = 2). Two patients underwent general anesthesia. Septic shock and circulatory collapse occurred in 3 and 5 patients respectively. MRI and CT showed lesions that may explain the wake-up delay in 6 of 11 and 4 of 16 patients, respectively. Brain anomalies included anoxic lesions (n = 4) with basal ganglia involvement (n = 3), ischemic or hemorrhagic strokes (n = 4), hepatic encephalopathy (n = 1) and herpetic encephalitis (n = 1).

**Conclusion:** Brain imaging techniques help diagnosing causes of delayed wake-up after induced coma. Anoxic lesions and strokes are mostly behind this condition. MRI is more accurate than CT.

### F-50 Confirming gastric tube placement in ICU: effectiveness of gastric ultrasonography

#### Abily Julien^1^, Derville Sébastien^1^, Lemaitre Caroline^1^, Lagache Laurie^1^, Grangé Steven^1^, Carpentier Dorothée^1^, Girault Christophe^1^, Le Bouar Gurvan^1^, Grall Maximilien^1^, Misset Benoit^1^, Tamion Fabienne^1^, Béduneau Gaëtan^1^

##### ^1^CHU de Rouen, France

###### **Correspondence:** Abily Julien - julien.abily@chu-rouen.fr

*Annals of Intensive Care* 2018, **8(Suppl 1):**F-50

**Introduction:** Gastric tubes are common in intensive care units used for enteral feeding, administration of drugs or aspiration of the digestive tract. These tubes offer an excellent tolerance but malposition may have serious consequences that can lead to patient’s death. The actualy gold method to confirm their correct placement is chest X-ray. We report a study which evaluate the performance of gastric ultrasonography for the validation of the good positioning of the gastric tube.

**Patients and methods:** We carried out a prospective, monocentric study in a Medical Intensive Care Units. For each included patient, we compared the results of a gastric ultrasonography to the interpretation of a chest X-ray.

**Results:** One hundred and thirteen gastric ultrasonographies were performed from July 2016 to May 2017. In 87 cases, ultrasonography concluded that the gastric tube was correctly positioned, confirmed by chest X-ray. In 24 cases, ultrasonography did not visualize the tube in gastric area. Among these 24 cases, only 4 malpositions were detected by the chest X-ray. The sensitivity and specificity of gastric ultrasonography were 0.81 [0.72 + 0.87] and 1 [0.51 + 1]. Positive and negative predictive values were 1 and 0.17, respectively. The ultrasonography was performed 54 min [50.3 + 57.6] after the gastric tube placement while the chest X-ray was interpreted 211 min [170.9 + 251.7] after this same placement (p < 0.0001).

**Conclusion:** Our results suggest a good performance of gastric ultrasonography to check the positioning of the gastric tube. This result must be interpreted with caution because of a low power of the study. We planned a multi-center study to confirm our results.

### F-51 Neutrophil-to-lymphocyte ratio improves prediction of mortality in cirrhotic patients hospitalized more than 3 days in intensive care

#### Giabicani Mikhael^1^, Lemaitre Caroline^1^, Grangé Steven^1^, Carpentier Dorothée^1^, Beduneau Gaetan^1^, Girault Christophe^1^, Misset Benoit^1^, Piton Gaël^1^, Paugam-Burtz Catherine^1^, Weiss Emmanuel^1^, Tamion Fabienne^1^

##### ^1^CHU Beaujon, APHP, Clichy, France

###### **Correspondence:** Giabicani Mikhael - mikhael.giabicani@aphp.Fr

*Annals of Intensive Care* 2018, **8(Suppl 1):**F-51

**Introduction:** Prognosis of cirrhotic patients hospitalized in intensive care unit (ICU) remains poor. In many ICUs, cirrhotic patients are widely admitted and revalued after receiving optimal treatments. Little is known about risk factors involved in the evaluation of the prognosis at day 3, except the persistence of organ failure. This susceptibility to organ failure would be related to an alteration of the regulation mechanisms of the systemic inflammatory response. The blood neutrophil-to-lymphocyte ratio (NLR) is an inflammation biomarker reported to predict clinical outcome in unselected critically ill patients and in patients with stable liver cirrhosis, but has never been studied in critically ill cirrhotic patients. The aim of this study was to evaluate the blood NLR as parameter to predict mortality of cirrhotic patients hospitalized > 3 days in ICU.

**Patients and methods:** Retrospective monocentric study including consecutively cirrhotic patients hospitalized in a medical ICU from 2010 to 2016. For each patient, clinical and biological data at admission and day 3 were collected. NLR at admission (“NLRD0”), at day 3 (“NLRD3”) and its variation between admission and D3 (“delta NLR”) were calculated. Statistical analysis used appropriate non parametric tests and Cox regression for survival analysis. The ability of the variables to discriminate survivors from non-survivors was determined using ROC curves and a Net Reclassification Index (NRI).

**Results:** 140 patients (median Child–Pugh score = 9 [7–11], median MELD score = 25 [19–30]) were hospitalized more than 3 days in ICU. The major causes for ICU admission were sepsis (56.4%), gastrointestinal bleeding (15%) or respiratory failure (6.4%). Patients were followed up for 38.5d [13–339]. 74 (53%) patients died—38 (27%) in ICU, 21 (15%) after ICU discharge and 15 (11%) after hospital discharge. In univariate analysis, factors significantly associated with mortality were—at D3, NLR, MELD and SOFA scores + and between D0 and D3—delta NLR, delta SOFA and delta MELD. Predictors of death in multivariate analysis are shown in Table 1. Area under delta NLR ROC curve was 0.74 (CI = 0.69–0.79). NRI revealed that delta NLR was more efficient than delta SOFA (NRI = 8.7%) to identify patients with a 85% mortality risk at least.

**Table:**
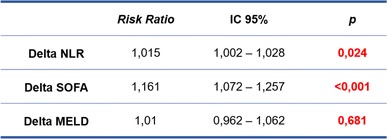

**Conclusion:** NLR is a novel inflammation index known to predict poor clinical outcomes. Delta NLR is an independent predictor of mortality in critically ill cirrhotic patients and could be more effective than delta SOFA in predicting hospital mortality in these patients.

### F-52 Severe liver dysfunction acute liver failure related to exertional heatstroke: outcomes, histological features and role of liver transplantation

#### Ichai Philippe^1^, Camus Christophe^1^, Boutonnet Mathieu^2^, Laurent-Bellue Astrid^1^, Moreau David^3^, Boudon Marc^1^, Aigle Luc^3^, Peron Jean Marie^4^, Gregoire Emilie^5^, Ichai Carole^6^, Quinart Alice^7^, Cousty^8^, Julien, Saliba Faouzi^1^, André Stéphane^1^, Coilly Audrey^1^, Antonini Teresa^1^, Samuel Didier^1^

##### ^1^Hôpital Pontchaillou, Villejuif, France; ^2^Hôpital des Armées, Clamart, France; ^3^France; ^4^CHU de Toulouse, France; ^5^Hôpital de la Timone, Marseille, France; ^6^CHU Pasteur, Nice, France; ^7^CHU de Bordeaux, France; ^8^CHU Saint Pierre, La Réunion, France

###### **Correspondence:** Ichai Philippe - philippe.ichai@aphp.fr

*Annals of Intensive Care* 2018, **8(Suppl 1):**F-52

**Introduction:** Severe acute liver injury and failure (sALI ALF) is a grave complication of exertional heatstroke (EH). Liver transplantation (LT) may be a therapeutic option, but the criteria for, and timing of, transplantation have not been clearly established. The aim of this study was to define the profile of patients who require transplantation in this context.

**Patients and methods:** This was a multicentre, retrospective study of patients admitted with a diagnosis of exertional heatstroke-related sALI ALF with a prothrombin time (PT) lower than 50%, with or without hepatic encephalopathy.

**Results:** 24 male patients (median age—27.5 years) with ALI ALF related to exertional heatstroke were studied + nine of them (37.5%) were listed for emergency LT. The latter differed from those who were not listed with respect to their more severe liver failure after D1, a clear deterioration in their PT and ALT values between D0 and D3, and more marked organ dysfunction. Four of these nine patients were subsequently transplanted. At the time of LT, all had PT levels lower than 10%, a marked rise in bilirubin levels and required support for at least one organ (or x organs were involved). Histological findings on the explanted livers demonstrated massive or sub-massive necrosis and little potential for effective mitosis with a mitonecrotic appearance. The 15 unlisted patients (62.5%) were still alive 6 months later and had not experienced any after-effects.

**Conclusion:** Survival without liver transplantation in patients with heatstroke-related ALI ALF reaches 83.5%. The indication for liver transplantation is based on an evolving dynamic. The lack of any signs of an improvement in liver function at or after D3, in patients presenting with other organ dysfunctions or failure, means that liver transplantation should be envisaged. The peculiar histological features observed on all the explanted livers, and the aspect of abortive mitoses in hepatocytes could be attributed to the effects of heatstroke.

### F-53 Should we use Prometheus for fulminant hepatitis ?

#### Roubin Johanna^1^, Kelway Charlotte^2^, Nafati Cyril^2^, Reydellet Laurent^2^, Blasco Valery^2^, Harti Karim^2^, Cungi Pierre Julien^2^, Albanese Jacques^2^

##### ^1^Hôpital de la Timone, Marseille, France; ^2^CHU de Toulon, France

###### **Correspondence:** Roubin Johanna - roubinjohanna@gmail.com

*Annals of Intensive Care* 2018, **8(Suppl 1):**F-53

**Introduction:** Without any treatment, patients with fulminant hepatitis die in 70 to 90% of cases. The Prometheus system allows to purify toxin bounded to albumin through adorption. Up to now, there a little data concerning its use during fulminant hepatitis. The aim of this study was to evaluate the Prometheus system on mortality and the need for liver transplant among patients with fulminant hepatitis.

**Patients and methods:** We conducted a retrospective observational study in a single polyvalent ICU of a teaching hospital. From December 2011 to December 2015 every patient admitted for fulminant hepatitis was included. We defined two groups—Prometheus group versus standard medical care group. Exclusion criteria were the contraindications for liver transplant.

**Results:** 45 patients were admitted for fulminant hepatitis during the study period, 21 patients were excluded. 12 patients had Prometheus and 12 had standard medical treatment. The average age was 40 (18–61), the mean SAPSII was 42 (15–99). On admission, the mean PT was 18.5% (8–42), the mean total bilirubin was 157 umol l. Paracetamol poisoning was the principal etiology with 45% of the patients—66% in the Prometheus group versus 25% in the standard group (p = 0.106). The hepatic encephalopathy grade was significantly higher in the Prometheus group—3 versus 0.5 in the standard group (p = 0.029). There was no difference between the two groups concerning mortality on day 28 (p = 1) or day 90 (p = 0.59). There was no difference concerning the length of stay in intensive care unit or in hospital between the two groups. 13 patients (37.1%) were transplanted. There was a statistical difference between the two groups concerning liver transplantation (p = 0.041)—4 transplant (40%) in the Prometheus group versus 9 transplant (75%) in the standard medical care group. There was a significant improve of encephalopathy after the Prometheus session (p = 0.002).
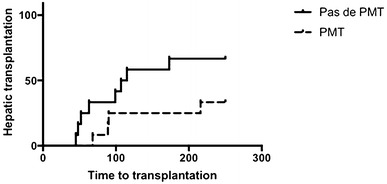


**Discussion:** Our study does not show any difference on mortality in fulminant hepatitis by using Prometheus. We found a statistical difference concerning the need for transplant which is significantly diminished after Prometheus. Prometheus may be a bridge to recovery. It is difficult to conclude because our study is retrospective and with only 24 patients. Also, there is a trend to have a majority on paracetamol poisoning in the Prometheus group.

**Conclusion:** Prometheus does not change the mortality in fulminant hepatitis. Prometheus may be a bridge to recovery with less need for liver transplantation.

### F-54 Extra corporeal liver therapy in the intensive care: a 12 years MARS^®^ experience from a French center

#### Monet Clément^1^, De Jong Audrey^1^, Prades Albert^1^, Chanques Gerald^1^, Conseil Matthieu^1^, Belafia Fouad^1^, Jaber Samir^1^

##### ^1^Hôpital Saint Eloi, Montpellier, France

###### **Correspondence:** Monet Clément - clemen.t@hotmail.fr

*Annals of Intensive Care* 2018, **8(Suppl 1):**F-54

**Introduction:** The Molecular Adsorbent Recirculating System (MARS^®^) is a device based on an albumin-enriched dialysis, allowing removal of albumin-bound toxins that accumulate during acute liver failure. Data on MARS^®^ are scarce and little is known on efficiency and tolerance. MARS^®^ therapy has been used in our ICU since 2005. We report here our experience with a focus on efficacy as well as tolerance.

**Patients and methods:** From March 2005 to August 2017 all patients who have undergone MARS^®^ therapy in our ICU were included consecutively and prospectively in the cohort. MARS^®^ therapy performed using a double lumen dialysis catheter in the femoral or jugular vein. We used the monitor MARS^®^ 1 TC (Teraklin) coupled with the dialysis machine Prismaflex^®^ (Gambro). The albumin dialysate circuit consisted of 500 ml of 20% human albumin and was regenerated by an anion-exchange column and an uncoated charcoal column (diaMARS^®^ IE250, diaMARS^®^ AC250).

**Results:** Ninety patients were included for 300 sessions. The mean duration was 7 h 38 min (± 1 h 43 min). The population treated consisted of 5 groups—acute-on-chronic liver failure (AoCLF), acute liver failure (ALF), post-surgery liver failure (post transplantation, post hepatectomy), refractory pruritus and drug intoxication (Fig. 1). Regarding biological efficacy—total bilirubin was lowered in AoCLF and post-surgery groups (p < .001), also in the ALF group although not significatively. MELD score was lowered in the AoCLF and ALF group (p < .001). However clinical variables (Glasgow score and encephalopathy) didn’t improve significatively. In the refractory pruritus group, pruritus decreased in 20 out of 25 patients (p < .001). Bile acid levels decreased to 34.8% of its mean baseline level (p < .001). In the drug intoxication group improvement of the Richmond Agitation-Sedation Scale (RASS) from deeply sedated (RASS < = − 3) to minimal sedation (RASS > = − 2) was obtained in 5 out of 6 patients. Out of 300 sessions, catheter-related adverse effects were low (1.3%), thrombocytopenia was the main adverse effect (26.7%).

**Fig. 1:**
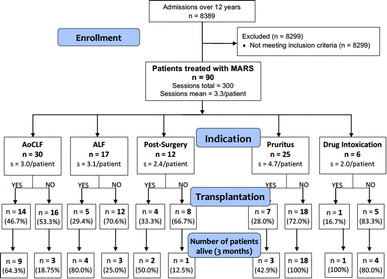
Admissions over 12 years

**Conclusion:** We report our MARS^®^ experience with the largest cohort of patients referred from a single hospital. We showed biological efficacy in all 5 indications, although clinical efficacy was uneven. MARS^®^ therapy in patients with refractory pruritus yielded promising results. Tolerance was good and the main adverse effect was thrombocytopenia. Global transplantation-free survival was low in patients with liver failure, reinforcing the need for a liver transplantation center when using MARS^®^.

### F-55 Digestive complications in veno-arterial ECMO-assisted patients after cardiac surgery

#### Margetis Dimitri^1^, Dujardin Olivier^1^, Bernard Remy^1^, Bouglé Adrien^1^, Amour Julien^1^

##### ^1^Pitié-Salpetriere, Paris, France

###### **Correspondence:** Margetis Dimitri - dimargetis@free.fr

*Annals of Intensive Care* 2018, **8(Suppl 1):**F-55

**Introduction:** Postoperative low cardiac output syndrome occurs in 4–26% after cardiac surgery. In case of refractory cardiogenic shock, veno-arterial extra corporeal membrane oxygenation (VA-ECMO) is used when pharmacological support fails to restore adequate perfusion. There is little evidence about severe digestive complications in this specific population. The aim this study was to investigate the incidence and the risk factors of gastrointestinal bleeding and mesenteric ischemia in patients assisted by VA-ECMO after cardiac surgery.

**Patients and methods:** Retrospective, observational study in a university hospital cardiac surgery department. All VA ECMO-assisted patients admitted in intensive care unit after cardiac surgery in a two-year period were included. Our study has been approved by ethic committee CPP Ile de France 5.

**Results:** At all, 160 patients have been included. Digestive complications occurred in 53 patients (33.1%). We observed 30 (18.7%) gastrointestinal bleeding. No risk factor has been identified. Bleeding was associated with prolonged assistance by VA-ECMO, mechanical ventilation, hospital length of stay, use of vasoactive drugs and transfusion. Mesenteric ischemia occurred in 32 patients (20%). In multivariate analysis, dialysis was the only significant risk factor, OR 6.35 [IC95 1.76–22.9] (p = 0.005). Heart transplant seems to be protective, OR 0.11 + [IC95 0.01–0.90 (p = 0.04). Attributed mortality increases consistently from 43 to 84.4% (p < 0.0001).

**Conclusion:** Severe digestive complications occur in 1 3 of VA-ECMO assisted-patients after cardiac surgery. The mortality increases by 95% in this population. Renal replacement therapy is identified as risk factor of ischemic complications.

### F-56 Colonoscopy in the intensive care unit: What indication for what profitability? A bicenter retrospective study

#### Messika Jonathan^1^, Issoufaly Tazime^1^, Duboc Henri^1^, Sztrymf Benjamin^2^, Prat Dominique^2^, Roux Damien^1^, Gaudry Stéphane^1^, Dreyfuss Didier^1^, Coffin Benoit^1^, Ricard Jean-Damien^1^

##### ^1^Hôpital Louis Mourier, Colombes, France; ^2^Hôpital Antoine Béclère, Clamart, France

###### **Correspondence:** Messika Jonathan - jonathan.messika@aphp.fr

*Annals of Intensive Care* 2018, **8(Suppl 1):**F-56

**Introduction:** Colonoscopy is crucial for the management of lower gastro-intestinal disorders, but its profitability is discussed in critically ill patients, mainly because of the complexity of colonic preparation. As the profitability of colonoscopy in Intermediate or Intensive Care Units (CICU) has been scarcely reported (1), we investigated its indications and usefulness.

**Patients and methods:** Retrospective bicenter observational study (2004–2015). Main endpoint: diagnostic profitability of CICU in unselected critically ill patients. Profitability was a priori defined as “high” if CICU led to adapt ongoing therapies; allowed an endoscopic intervention; or participated in the decision to limit therapeutic effort. Secondary endpoints: describe the quality of CICU and its preparation; determine its position in diagnosis strategy; describe its morbidity.

**Results:** One hundred and eleven CICU were performed in 84 patients (male sex n = 50 + 60%, age 72y [63–84] + SAPSII 73 [62–84]) within 3 days [1–10] after admission. Gastro-intestinal bleeding or haemorrhagic shock was the cause of admission in 35 patients (42%). On the day of CICU, 37 patients (44%) had shock. CICU indications were—haemorrhage (n = 77) with 35% of acute anaemia (n = 39) + suspected colitis (ischemic [n = 19, 17%], infectious [n = 17, 15%], toxic [n = 5, 5%], inflammatory [n = 1, 1%]) + investigation of a Gram negative bacilli sepsis (n = 7, 6%) + sigmoid volvulus (n = 4, 4%) + and cancer diagnosis (n = 4 + 4%). CICU profitability was deemed high in 50% (n = 56), with an endoscopic intervention performed in 32% (n = 35). The CICU lead to antimicrobial adaptation (n = 9), emergent surgery (n = 10), or to limit therapeutics effort (n = 2). In 23 cases (21%) the CICU was considered normal. Patients’ preparation was rated as good in 27% (n = 30) + and the colonoscopy was complete in 33 (30%). The CICU was mainly performed as a 2nd (58%) or 3rd (40%) investigation after an abdominal CT-scan or an upper digestive endoscopy (respectively performed in first instance in 69 and 55%). Three CICU were complicated by 3 hemodynamic and 2 respiratory failures, none were fatal.

**Discussion:** In our series of unselected critically ill patients, CICU were mainly performed to investigate lower gastro-intestinal bleeding. Despite a low rate of good preparation, CICU is safe and its profitability is high in the majority of cases.

**Conclusion:** Although performed in poor conditions, CICU seems useful in the diagnostic and the therapeutic management of critically ill patients, and not only in gastro-intestinal bleeding. (1) Church, Surgical endoscopy 2014.

### F-57 Correlation between non-invasive pain assessment using the analgesia nociception index and Behavioral Pain Scale in critically ill patients: a prospective observational study

#### Masse Juliette^1^, Dejonckheere Julien^1^, Broucqsault-Dedrie Céline^1^, Houard Marion^1^, Nseir Saad^1^

##### ^1^CHU de Lille, France

###### **Correspondence:** Masse Juliette - juliette.masse2911@gmail.com

*Annals of Intensive Care* 2018, **8(Suppl 1):**F-57

**Introduction:** Accurate pain assessment is associated with better outcomes in intensive care unit (ICU) patients. Specific scales for non-communicative patients have been developed and validated but their routine use still remains inaccurate and subjective. Analgesia Nociception Index (ANI) is based on high-frequency heart rate variability. This study objective was to assess the correlation between the Behavioral Pain Scale (BPS) and ANI during care procedures in deeply sedated patients.

**Patients and methods:** We conduced a French multicentric prospective observational study with blinded continuous recording of ANI during 8 h with spotting of care procedures in patients with RASS less or equal to − 2. We compared pain assessment using BPS and ANI before (t1) and during (t2) each care procedure. The cares analyzed included prick glycaemia, turning, catheter insertion, dressing change and others. A behavioral pain reactivity (BPR) was defined by a BPS elevation of at least 1 point. We analyzed minimal ANI values and its variations with calculation of DeltaANI (ANIt1– ANIt2). Because of the analysis of several cares per patient we used a Bonferroni’s correction in comparison of BPR and no BPR groups with a significant p value < 0.025 for this comparison. For others analyses the p value considered as significant was p < 0.05. Correlation between ANI and BPS was analyzed using a Spearman correlation rank test.

**Results:** Nighty two cares were analyzed in 46 patients. A BPR was noted for 29 cares (32%). ANI before care procedure was comparable in BPR and no BPR groups (p = 0.19). The most frequent care was turning in the BPR group (55%) and prick glycaemia (40%) in the no BPR group. Compared to the period before care, minimal ANI decreased significantly during care procedure in BPR group (median (IQR) 53 [35–82] vs 43 [28–50] + p < 0.001), whereas it did not reach statistical significance in the no BPR group (49 [35–58] vs 44 [31–60] + p = 0.049). DeltaANI was higher in the BPR group than in the no BPR group (8 [2–33] vs 3 [− 4 to 13] + p = 0.009). A low correlation was noted between DeltaANI and BPS during care (r = 0.211, p = 0.044).

**Conclusion:** ANI decreases significantly during procedures where BPS raises and not during procedures with constant BPS. A correlation was founded between BPS and DeltaANI. Thus, ANI monitoring could be a helpful tool for daily assessment of pain in sedated critically ill patients.

### F-58 Assessment of virtual reality as an adjunct therapy in pain management in burn patient

#### Mokline Amel^1^, Gharsallah Lazheri^1^, Rahmeni Imen^1^, Chaouch Nadia^1^, Messadi Amen Allah^1^

##### ^1^CHU Ben Arous, Tunis, Tunisia

###### **Correspondence:** Mokline Amel - dr.amelmokline@gmail.com

*Annals of Intensive Care* 2018, **8(Suppl 1):**F-58

**Introduction:** The pain associated with burn was one of the most painful injuries to treat. Pain was induced by therapeutic acts such as wound debridement, dressing and other painful procedures. Burn pain caused changes in neurophysiology and pharmacokinetics that may make standard pharmacologic analgesic therapy less effective than usual.virtual reality has been explored as an adjunct therapy for the management of acute pain for a number of conditions. In our study, we attempt to assess the impact of virtual reality on management of burn pain during dressing changes.

**Patients and methods:** Before the therapeutic procedure (dressing changes), the concept of virtual reality therapy was explained to the patient (technology and equipment used). The video used was snow mountain. During the act, pain was assessed until the end of the procedure. The assessment of pain was based on visual analog scale (vas). For pain intensity, the scale was most commonly anchored by “no pain” (score of 0) and “very intense pain” (score of 10).

**Results:** During the study period, 20 patients were included. The mean age was 32 ± 17 years. 75% of our patients were adults aged over 20 years. They were 17 men and 3 women. the average burned surface area was 30 ± 13%. Pain was evaluated before the start of the therapeutic procedure. The mean initial pain severity score was 8.85 ± 0.74 (range 8 to 10). The pain assessment after virtual reality condition showed a significant decrease in the intensity of pain (p < 0.01). The mean pain decreased from 8.85 to 4.6 ± 0.84 with extremes ranging from 1 to 4.

**Conclusion:** Our study supports the use of virtual reality, simple non-invasive, as an adjunct therapy in the management of pain associated with dressing changes in burn patients.

### F-59 Evaluation of patient comfort during abdominal drain removal using hypno-analgesia in intensive care unit

#### Amar Yaël^1^, Alves Sylvie^1^, Le Bec Caroline^1^, Puechberty Christelle^1^, Rousseau Isabelle^1^, Blot François^1^

##### ^1^Institut Gustave Roussy, Villejuif, France

###### **Correspondence:** Amar Yaël - yamar80@gmail.com

*Annals of Intensive Care* 2018, **8(Suppl 1):**F-59

**Introduction:** Hypno-analgesia (HA) is used in the operating room and for complex pain. Before implementation of HA in our intensive care unit (ICU), most protocols for algogenic procedures included intravenous or epidural morphine and Nitrous Oxid. Since 2016, many caregivers have been trained, HA has been implemented and patient comfort is evaluated using 1) a specific analogic scale of comfort (0 to 10) before and after the procedure + 2) at the end of the procedure, a score of patient and caregiver comfort using a five item questionnaire (5 to 20 points).

**Patients and methods:** This pilot prospective study compares HA versus the standard protocol in the removal of abdominal drains after digestive surgery. The main objective was to evaluate the patient comfort before after the procedure using a scale of comfort + the secondary objectives were to test the patient and caregiver comfort scores and evaluate in the impact on consumption of analgesic. Between May 2016 and September 2017, two groups were obtained, according whether the procedure was performed by HA-trained or non-HA-trained professionals (depending on caregivers availability in the unit). The number of subjects required to compare scales of comfort before vs. after drain removal was 66, using a nonparametric Wilcoxon–Mann–Whitney test.

**Results:** Eighty-eight patients were analyzed. The mean note in the comfort scale remained unchanged after vs. before drain removal in patients without HA (n = 43, +0.04 points, ± 2.8), while it increased in patients with HA (n = 45, +2.07, ± 2.7 + p = 0.001). Using our specific five item comfort score, patients and caregivers had a comparable level of satisfaction in HA and non-HA groups (Patients 17.4 20 and 16.3 20 + caregivers—19 20 in both). A trend was observed in reduction of the consumption of morphine and Nitrous Oxid with HA, without altering their comfort.

**Discussion:** Despite its limitations (mainly, its open non-randomized design), this study suggests that—HA may be used for algogenic procedures and is willingly adopted in ICU by patients and professionals + specific scales scores, adapted for HA, may be useful to assess the effectiveness + finally, HA seems to be at least as efficient as classical procedures and could reduce the use of analgesic drugs.

**Conclusion:** HA adds value to patients and to all caregivers. Prospective randomized studies are needed to valid the comfort scores we proposed, and to prove that HA reduces the consumption of analgesic drugs.

### F-60 The effects of standardized musical intervention (Music Care© type) during painful cares on vigils patients in intensive care unit

#### Guilbaut Victoria^1^, Gouteix Eliane^2^, Koubi Claude^2^, Fosse Jean Philippe^2^

##### ^1^Annecy Le Vieux, France; ^2^Hôpital Les Sources, Nice, France

###### **Correspondence:** Guilbaut Victoria - victoria.guilbaut@hotmail.fr

*Annals of Intensive Care* 2018, **8(Suppl 1):**F-60

**Introduction:** Pain has long been a focus of concern for doctors and caregivers. In intensive care unit, the inability to verbalize discomfort and pain are major stressors for patients. Music therapy has demonstrated in many international studies its effect on the blood pressure and on the respiratory frequency. In this context, we conducted a study to evaluate the effects of standardized musical intervention on pain during painful cares in vigils patients hospitalized in critical care.

**Patients and methods:** Design—We conduct a prospective, observational, randomised, single blind, mono center study. 140 painful cares were studied and then distributed in two groups (n = 70 with music, n = 70 without music). The patients were equiped with a Bose© helmet, and had or not music therapy during the care. Our main criteria was the pain, it has been evaluated by a numeric scale before and after the painful care. We also estimated anxiety with the COVI’s heteroevaluation scale before and after the car. We also noticed if the care were stopped because of the pain, then we used a semi quantative numeric scale in order to estimate the feeling of the caregiver and the patient on the session.

**Results:** Concerning pain, there is no significant difference between the two groups (p > 0.005). However, in the music group, pain decreased by 35% after the care (p < 0.001). Anxiety was way lower in the music group than in the group without music (p < 0.001). We also noticed a decrease of 50% of the anxiety in the music group. The patients and the caregivers’ feeling were the same in the two groups, with no significant difference (p > 0.05). On the other hand, caregivers tended to underestimate the difficulty of the session in comparison with the patients’ (p < 0.01) in both groups.

**Conclusion:** Music therapy did not improve the pain in a significant way, in the music group versus the group without but allowed a decrease of 35% of the pain after the care. Nevertheless, music reduced by two patients’anxiety.

### F-61 Impact of a nurse implemented sedation and analgesia algorithm on complications of critical illness in surgical intensive care unit

#### Pottier Véronique^1^

##### ^1^Hôpital Côte de Nacre, Caen, France

###### **Correspondence:** Pottier Véronique - veroniquepottier@icloud.com

*Annals of Intensive Care* 2018, **8(Suppl 1):**F-61

**Introduction:** Sedation and analgesia is one of the basic themes in ICU as complications associated with excessive sedation negatively impact the morbidity and mortality of patients. The objective of this study is to show that the nurse implementation of a sedation and analgesia algorithm is beneficial to the patient in terms of sedative drugs reduction and thus overall decrease in duration of mechanical ventilation (MV) and the morbidity and mortality which is associated with it, without altering patient comfort and tolerance of the environment.

**Patients and methods:** A before and after prospective, observational, non-interventional study was conducted in surgical ICU in Caen University Hospital, between November 2014 and April 2017. Mechanically ventilated patients under sedation predicted to last 48 h or more were included. During the “Before” period, sedation and analgesia was managed by the physician, while during the “After” period, it was managed by the nurses according to the protocol.

**Results:** 1156 intubated and mechanically ventilated patients were admitted during the study period. Among the 145 eligible patients, 100 were included during “Before” period and 45 during “After” period. The duration of MV after inclusion was significantly shorter in group “After” (10.5 [7 + 18] vs 8 [5 + 11.5] days, p = 0.042), as the duration of target RASS (-2 à 0) was significantly longer (0 [0 + 2] vs 1 [0 + 3] day, p = 0.038), the duration of RASS < − 2 significantly shorter (6.5 [4 + 12] vs 3 [2 + 5] days, p < 0.001), dose of sedative drugs was significantly decreased (1330 [517.5 + 2543.8] vs 315 [15 + 720] mg, p < 0.001 for hypnotics and 1803 [1097.5 + 4290] vs 900 [450 + 1680] µg, p < 0.001 for opioids, respectively), and sedation cost (25.1 [12.7 + 67.4] vs 12.2 [7.3 + 34] euros, p = 0.004). The patients experienced less of ventilator-acquired pneumonia (VAP) and delirium during the “After” period (55 vs 24.4%, p = 0.004, and 41 vs 26.7%, p = 0.015, respectively).

**Conclusion:** The nurse implementation of a sedation and analgesia algorithm was associated with a trend towards reduction in duration of MV, ICU and hospital length of stay. Moreover, prevalence of VAP and delirium was reduced, in correlation to the significant decrease in sedative drugs. This type of algorithm is necessary to reduce morbidity and mortality associated with MV.

### F-62 Remifentanil for analgesia in central venous catheter insertion: a randomized, controlled trial

#### Pichon Xavier^1^, Vardon Bounes Fanny^1^, Ducos Guillaume^1^, Ruiz Jean^1^, Samier Caroline^1^, Silva Stein^1^, Sommet Agnès^1^, Fourcade Olivier^1^, Conil Jean-Marie^1^, Minville Vincent^1^

##### ^1^CHU de Toulouse, France

###### **Correspondence:** Pichon Xavier - pichon.x@chu-toulouse.fr

*Annals of Intensive Care* 2018, **8(Suppl 1):**F-62

**Introduction:** Central venous catheter insertion is a common practice for anesthetists and intensivsts. This invasive procedure generates pain and anxiety for patients. We aim to demonstrate that remifentanil improves the analgesia during scheduled central venous catheter insertion in mindful patients.

**Patients and methods:** A prospective, randomized, double-blind, controlled study in patients requiring central venous access. Patients were randomly assigned to receive 3 ng ml^−1^ remifentanil target controlled infusion (TCI) and local anesthesia (LA) with lidocaine or placebo and LA. All patients were monitored in intensive care or post-intervention care unit and systematically received oxygen. Patients were asked to assess verbal numeric rating pain scale (VNRPS) during the procedure. The primary outcome was the maximal VNRPS. Secondary outcomes were pain at each step, anxiety, patient satisfaction, operator ease and side effects.

**Results:** Ninety patients were included (45 in each group). All patients were analyzed. Remifentanil significantly reduced maximal pain—VNRPS 20 (95% confidence interval [CI] 16–40) vs 50 (95% CI 40–60) in the placebo group p = 0.0009 (Table 1). We did not observe any adverse event during this study, and there were no significant difference between the 2 groups regarding side effects.

**Table 1:** data during procedure

	Group		*p* value
	Placebo (n = 45)	Remifentanil (n = 45)	
Maximal VNRPS	50 (40–60)	20 (16–40)	.0009*
VNRPS at punction	30 (20–30)	20 (2–20)	.0746
VNRPS at dilatation	40 (25–40)	10 (0–20)	.0005*
VNRPS at fixation	30 (20–40)	10 (0–20)	.0153*
N. of punctions	1 (1–2)	1 (1–1)	.3446
Duration (mn)	17 (14–22)	15 (14–19)	.3389
Dose (µg/kg/mn)	0 (NA)	0.135 (0.12–0.15)	NA
Total dose (µg)	0 (NA)	136 (120–164.6)	NA

Data are expressed as median (95% confident interval)

*VNRPS* Verbal Numeric Rating Pain Scale

**Conclusion:** TCI remifentanil is a safe procedure to reduce pain during central venous catheter insertion in awake patients. Trial Registration—clinicaltrials.gov Identifier—02206022, REMIDOLCATH.

### F-63 Comparison of two sedation regimens during targeted temperature management after cardiac arrest

#### Paul Marine^1^, Bougouin Wulfran^1^, Cariou Alain^1^, Dumas Florence^1^, Geri Guillaume^1^, Guillemet Lucie^1^, Champigneulle Benoit^2^, Ben Hadj Salem Omar^1^, Legriel Stéphane^3^, Chiche Jean-Daniel^1^, Charpentier Julien^1^, Mira Jean-Paul^1^, Sandroni Claudio^4^

##### ^1^Hôpital Cochin, Paris, France; ^2^Hôpital Européen Georges Pompidou, Paris, France; ^3^Hôpital Mignot, Paris, France; ^4^Catholic University School of Medicine, Rome, Italy

###### **Correspondence:** Paul Marine - marine.1604@hotmail.fr

*Annals of Intensive Care* 2018, **8(Suppl 1):**F-63

**Introduction:** Although guidelines on post-resuscitation care recommend the use of short-acting agents for sedation during targeted temperature management (TTM) after cardiac arrest (CA), the potential interests of this strategy have not been clinically demonstrated.

**Patients and methods:** Before-after study. We compared two sedation regimens (propofol-remifentanil, period P2 vs midazolam-fentanyl, period P1) among comatose TTM-treated CA survivors. Management protocol, apart from sedation and neuromuscular blockers use, did not change between the two periods. Baseline severity was assessed with Cardiac-Arrest-Hospital-Prognosis (CAHP) score. Time to awakening was measured starting from discontinuation of sedation at the end of rewarming. Awakening was defined as delayed when it occurred after more than 48 h.

**Results:** 460 patients (326 in P1, 134 in P2) were included. CAHP score in P2 and P1 did not significantly differ (p = 0.93). Sixty percent of patients awoke in both periods (194 326 vs 81 134, p = 0.85). Median time to awakening was 2.5 (IQR 1–9) hours in P2 vs. 17 (IQR 7–60) hours in P1. Awakening was delayed in 6% in P2 vs. 29% of patients in P1 (p < 0.001). After adjustment, P2 was associated with significantly lower odds of delayed awakening (OR 0.08, 95% CI 0.03–0.2 + p < 0.001). Patients in P2 had significantly more ventilator-free days, and lower catecholamine-free days between admission and day 28. Survival and favorable neurologic outcome at discharge did not differ across periods.

Time course for awakening according to sedation period.
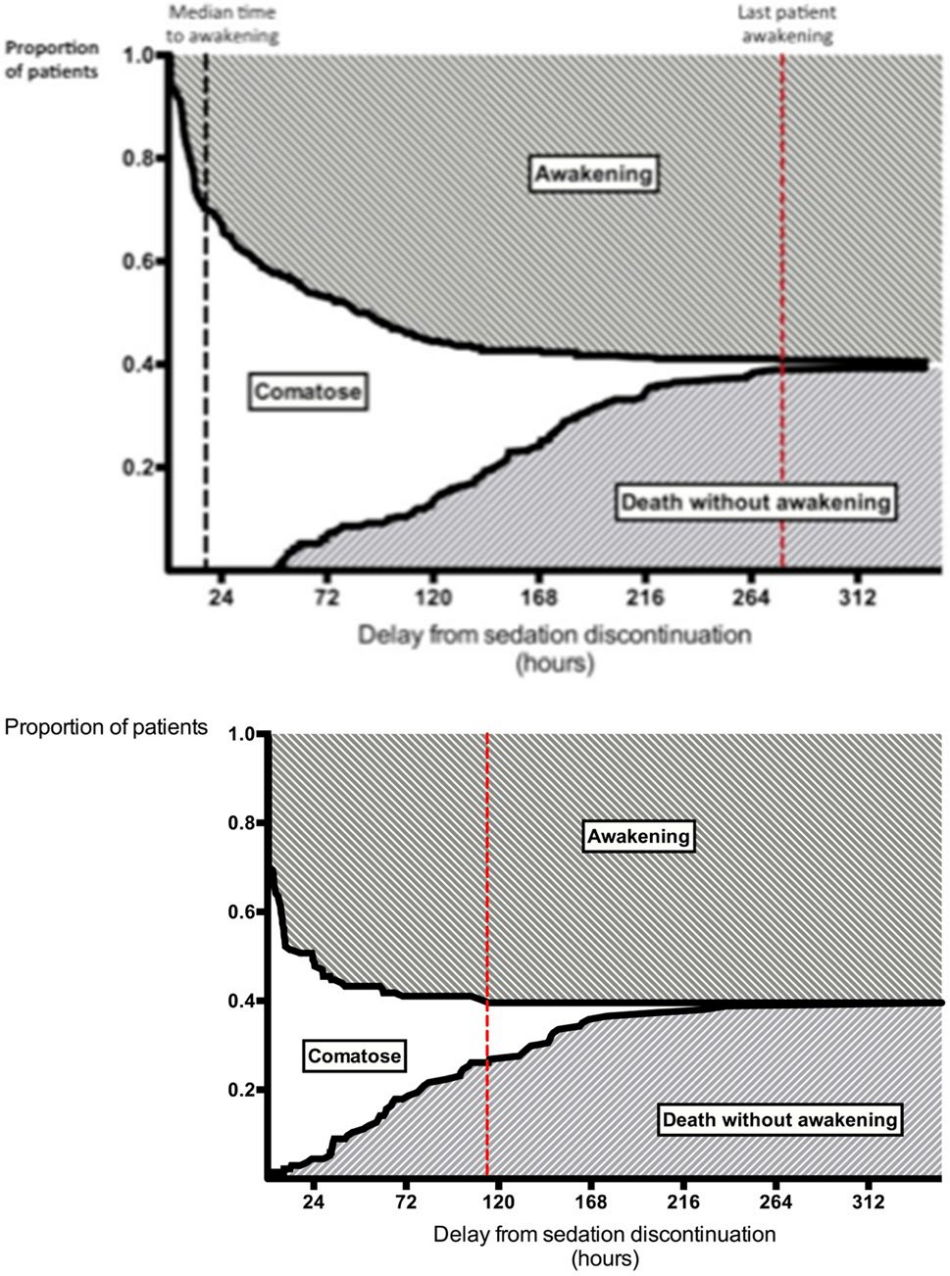


These figures report the time course of patients included after discontinuation of sedation. At each time point, we report in the upper part, proportion of patients awoken after discontinuation of sedation, in the lower part, patients who died without awakening, and in the middle part (in white), patients still comatose. Red dots indicate, for each period, the last patient awakening (after 12 days in P1 and 5 days in P2).

Black dots indicate the median time to awakening (too early to appear for period 2).

**Conclusion:** During TTM following resuscitation from CA, sedation with propofol-remifentanil compared with midazolam-fentanyl was associated with an earlier awakening, and an increase in ventilator-free days.

### F-64 Conformity of drug administration and central venous access in intensive care unit: a prospective evaluation

#### Perez Justine^1^, Lotito Adrien^1^, Jean Emmanuel^1^, Ouvrier Maryse^1^, Escudier Etienne^1^, Levrat Albrice^1^, Sirodot Michel^1^, Guerin Franck^1^

##### ^1^CH de Metz Tessy, France

###### **Correspondence:** Perez Justine - justine.perez03@orange.fr

*Annals of Intensive Care* 2018, **8(Suppl 1):**F-64

**Introduction:** The new recommendations of the French society of anesthesia-intensive care (SFAR) on perfusion and medication errors were revised in 2016 to promote proper use relating to drug administration with medical devices. To advance that of inquiry, practices of our intensive care unit (ICU) were assessed in order to improve drug administration by central venous catheter (CVC).

**Patients and methods:** Prospective evaluation by pharmacist resident and technical nurse during seven weeks, using a standard evaluation tool, in a 16 bed ICU. Drug recommendations and SFAR documents from 2016 were used as referential of conformity. The following parameters were evaluated—1 Central venous lines mounting, 2 Drug administration and identification with a focus on narrow therapeutic index (NTI) drugs.

**Results:** 50 patients with CVC were analyzed between June and July 2017. 19 entered directly in the ICU. 23 were hospitalized for surgical reasons. 42 had triple-lumen CVC. Regarding the first parameter, no conformity was found due to lack of line identification (100%) or anti-return valve well positioned (96%). Perfusion ramp position was above heart level in 10%, infusion tubing had contact with floor in 6%, and absence of plug on non-used lines is found in 8% of cases. Regarding second parameter, non-conformities were due mostly to syringe label—absence of drug’s concentration (72%), preparator identification (99%), patient identification (99.7%), drug identification (3.5%, all concerning propofol), date and time of medication preparation (67%), lack of color code of labels (100%). Regarding NTI, 70% were not administrated according to the recommendations—absence of dedicated line (63%), absence of administration on the nearest insertion site of the catheter (67%).

**Conclusion:** The evaluation highlight some improvement axis such as complete identification on syringes, sensitizing of ICU healthcare team, or homogenization of CVC perfusion system. It calls for a second evaluation round after implementation of improvements.

### F-65 Impact of a sedation protocol in patients treated with invasive mechanical ventilation: a retrospective before-after study

#### Piriou Pierre-Guillaume^1^, Brule Noelle^1^, Delbove Agathe^1^, Lascarrou Jean-Baptiste^1^, Martin Maelle^1^, Pontis Emmanuel^1^, Lemeur Anthony^1^, Garret Charlotte^1^, Reignier Jean^1^

##### ^1^CHU de Nantes, Nantes, France

###### **Correspondence:** Piriou Pierre-Guillaume - pgpiriou@wanadoo.fr

*Annals of Intensive Care* 2018, **8(Suppl 1):**F-65

**Introduction:** Sedation is a corner stone of the care of patients receiving mechanical ventilation in the ICU. Sedation was associated with increased comfort and adherence to care, but also with increased morbidity, including delirium, increased duration of mechanical ventilation and length of ICU stay. Previous studies reported beneficial impact of reduced doses of sedative drugs and careful monitoring of patients comfort and consciousness. Our goal was to assess the impact of the introduction of a nurses-dedicated sedation protocol in our ICU.

**Patients and methods:** This monocentre retrospective before-after study included all the patients admitted in our ICU, over two three-month periods, from July 2015 and January 2016, treated with invasive mechanical ventilation for more than 24 h and older than 18yrs. After the first period, all physicians and nurses were trained to a new sedation management protocol. Analysis was performed to assess the prescription and application of the protocol, its impact on the use of sedative drugs, ICU length of stay, and duration of mechanical ventilation. Major complications were also recorded.

**Results:** 134 patients were included—78 before and 56 after the protocol implementation. Patients in both groups had similar baseline characteristics (Men 67 vs. 50%, p = 0.08 + mean age 56 ± 18 vs. 60 ± 16 years, p = 0.1 + weight 73.2 ± 20.6 vs. 70.2 ± 15.9 kg p = 0.61 + IGS2 44 ± 17 vs. 44 ± 19, p = 0.99 + medical admission 91 vs. 88%, p = 0.51). Recordings of RASS and BPS did not differ between groups (9 ± 4 vs 10 ± 9, p = 048 + 19 ± 25 vs, 16 ± 11, p = 0.25). The duration of sedation was significantly shorter after introduction of protocol (4.1 ± 4.7 vs 1.9 ± 1.0, p < 0.0001), as was the duration of mechanical ventilation (9.3 ± 13.8 vs 4.1 ± 3.2, p = 0.001) and ICU length of stay (12.3 ± 15.8 vs 7.3 ± 5.3, p = 0.028). There was no difference in major ICU complications, nor in mortality between groups (25 and 30%).

**Conclusion:** Although the implantation of a sedation protocol did not translate in increased recording of RASS and BPS scores, it was associated with improved outcomes. Our data suggest that, more than the protocol by itself, beneficial effects reported after the implementation of a sedation protocol may be ascribed to increased awareness of the care givers and thus better management of sedation.

### F-66 Assessing the impact of specialized clinical pharmacists on patient care in the critical care unit: a systematic review

#### Goyer Isabelle^1^, Villarbu Mathilde^1^, Jokic Mikael^1^, Brossier David^1^

##### ^1^CHU de Caen, Caen, France

###### **Correspondence:** Goyer Isabelle - goyer-i@chu-caen.fr

*Annals of Intensive Care* 2018, **8(Suppl 1):**F-66

**Introduction:** Critically ill patients often necessitate multiple high-risk supportive pharmacologic treatments and exhibit complex and rapidly changing pharmacokinetic parameters. Their constantly evolving medical condition warrants continuous reevaluation and adjustments in pharmacotherapy. Multiple studies have shown the impact of pharmacist’s interventions on the reduction of medication prescription errors and extrapolated hypothetic cost savings. Critical care societies around the world recommend full time specialized clinical pharmacy services in the intensive care unit (ICU). Nonetheless, the full impact of specialized clinical pharmacy in the critical care unit should be appreciated in terms of meaningful direct patient outcomes such as length of ICU stay, length of mechanical ventilation (MV), risk of MV associated pneumonia or other nosocomial infections, risk of delirium, thromboembolic events or mortality and direct actual expenditure savings.

**Patients and methods:** Literature review (PubMed, google scholar and cross-referencing of selected articles) with selected Mesh terms (Intensive Care Units [Mesh] OR Critical Illness [Mesh] OR Resuscitation [Mesh]) AND (Pharmacists [Mesh] OR Pharmacy [Mesh]) done on August 28th, 2017. Original research studies written in English or French evaluating the impact of an ICU clinical pharmacist’s care on clinical endpoints were selected. All available articles were included without time limit, oldest article dating back to December 1991.

**Results:** The literature search yielded 157 references to which additional relevant references were added by cross-referencing selected articles. 26 studies evaluating the impact of clinical pharmacists on patient outcomes in the critical care setting were selected for this analysis. One hundred percent of the studies reported positive measurable clinical outcomes of the presence of a clinical pharmacist in critical care setting. Eighty-eight percent of the studies were done in the USA and 31% were prospective trials (n = 1348 patients). Six studies evaluated actual monetary expenditure and 100% of them concluded in cost-effectiveness of critical care clinical pharmacy services.

**Conclusion:** ICU clinical pharmacists do not only intercept medication prescription errors once they happen and decrease related hypothetic institutional costs. Their full time presence and interdisciplinary team inclusion in the ICU globally improves patient care and translates into tangible benefits in terms of patient outcomes and direct monetary savings. We can no longer ignore international recommendations warranting full time specialized clinical pharmacy services in critical care units to ensure patient care efficiency, safety and cost-effectiveness. Critical care clinical pharmacy services should be considered as an essential element of a standard ICU.

### F-67 Impact of implementing a computer-based prescription system and moving in new buildings on healthcare workload—the case for acute respiratory failure (ARF) and sepsis

#### Dachraoui Fahmi^1^, Tlili Mariem^1^, Bouker Nouha^1^, Hraiech Kmar^1^, Nouira Wiem^1^, Ouni Amal^1^, Zorgati Hend^1^, Boukadida Sana^1^, Hammouda Zaineb^1^, Ouanes Islem^1^, Ouanes Besbes Lamia^1^, Abroug Fekri^1^

##### ^1^Chu Fatouma Bourguiba, Monastir, Tunisia

###### **Correspondence:** Dachraoui Fahmi - dachraoui.fahmi@gmail.com

*Annals of Intensive Care* 2018, **8(Suppl 1):**F-67

**Introduction:** Workload affects the quality of care and the prognosis of critically ills patients. Measuring workload in intensive care units (ICU) has thus become essential for allowing a better matching between the activities required and the management of resources. In March 2016, the medical ICU of the university hospital of Monastir (Tunisia) moved into new buildings (more space and beds, computer-based prescriptions and monitoring, etc.). The aim of the present study is to compare the level of workload before and after the change of the ICU buildings.

**Patients and methods:** During the two study periods (period 1- July–September 2015 and period 2- July–September 2017) adult patients consecutively admitted, for more than 24 h, in the medical ICU for ARF and or sepsis were included in the analysis. Data collected were the demographic characteristics (age, sex, Body Mass Index (BMI), comorbidities, Simplified Acute Physiology Score (SAPS) III), the nursing workload measured using the Therapeutic Intervention Scoring System (TISS-28) and hospital survival.

**Results:** Thirty-six patients (22 male) were included in the study (14 during Period 1 and 22 during the second period). The medians of age, SAPS III and BMI were respectively 65 (IQR = 21) years, 46 (IQR = 12) and 27.7 (IQR = 9.4). The main comorbidities were hypertension, COPD and neurological disease respectively in 36, 25 and 20%. The demographic characteristics were similar during the two periods. Nurse workload was characterized by m TISS-28 = 18 (IQR = 7) and Time of nurse’s care of 190 min (IQR = 74). These two workload indicators were significantly higher during the second period (Table 1). During the second period, “standard monitoring” and “frequent dressing changes” (> 3 time day) were the activities with significant increase from, respectively 81 to 91% (p < 0.0001) and from 41 to 88% (p < 0.0001).

**Table 1:** Comparison of workload indicators in two ICU locations

	Period 1	Period 2	*p*
TISS-28: points (IQR)	15 (5.1)	19.7 (5.8)	< 0.0001
Time of nurse’s care : mn (IQR)	159 (54)	209 (61)	< 0.0001

**Conclusion:** The relocation of our ICU in in new buildings was associated with a significant increase of the nurse workload with regard to patients with ARF and or sepsis.

### F-68 Creating a high resolution electronic database in the pediatric intensive care unit: validation phase

#### Mathieu Audrey^1^, Brossier David^2^, Sauthier Michael^1^, El Taani Redha^1^, Goyer Isabelle^1^, Emeriaud Guillaume^1^, Jouvet Philippe^1^

##### ^1^CHU Sainte Justine, Montréal, Québec, Canada; ^2^CHU de Caen, France

###### **Correspondence:** Mathieu Audrey - audreymathieu22@icloud.com

*Annals of Intensive Care* 2018, **8(Suppl 1):**F-68

**Introduction:** The HSJ-PICU database is a high resolution electronic database automatically collected in the pediatric intensive care unit (PICU) of the Montreal Sainte Justine hospital (HSJ). Every 5 to 30 s, this database collects all physiological, therapeutic and clinical data from medical devices at bedside of all children admitted in the PICU. To ensure that these data are accurately collected in the HSJ-PICU database, we conducted a real time data validation study.

**Patients and methods:** The prospective validation study was performed from June 23rd to July 11th 2017. Every day, each monitor (IntelliVue MP60 and MP70, Koninklijke Philips Electronics) of all hospitalized children in the PICU was video recorded during 30 s. The videotaped data were compared to the simultaneously collected data in the HSJ PICU database using a Bland–Altman analysis.

**Results:** Data from 95 patients’ days were recorded. Overall 3279 data were video recorded and compared to the corresponding data collected in the HSJ-PICU database (1104 heart rates, 1079 respiratory rates, 975 pulse oximetries, 43 central venous pressures, 12 end tidal PCO2, 66 mean arterial pressures). Bland–Altman analysis showed excellent accuracy and precision between recorded and collected data for all tested variables within clinically significant pre-defined limits of agreement. However, 82 (2.5%) data were missing and a delay was observed between videotaped and collected times. This delay was less than 28 s and remained stable through all data for each patient. We identified that the missing data were due to a limit in the number of data being processed in the database at the same time and the delay between data presentation and data collection in the database was due to different server time settings. Both technical issues were corrected.

**Conclusion:** Our study identified two issues in the data collection process that slightly limited the accuracy of our high resolution electronic database. We recommend the performance of such validation study before using a high resolution database for clinical or research purposes.

### F-69 Association between bodyweight variation and survival and other adverse events in critically ill patients with shock: a multicenter cohort study of the OUTCOMEREA network

#### Dupuis Claire^1^, Gros Antoine^1^, Ruckly Stéphane^1^, Lautrette Alexandre^1^, Garrouste-Orgeas Maité^1^, Gainnier Marc^1^, Forel Jean-Marie^1^, Marcotte Guillaume^1^, Azoulay Elie^1^, Cohen Yves^1^, Schwebel Carole^1^, Argaud Laurent^1^, De Montmollin Etienne^1^, Siami Shidasp^1^, Goldgran-Toledano Dany^1^, Darmon Michael^1^, Timsit Jean-François^1^

##### ^1^Hôpital André Mignot, Paris, France

###### **Correspondence:** Dupuis Claire - cdup83@gmail.com

*Annals of Intensive Care* 2018, **8(Suppl 1):**F-69

**Introduction:** Fluid overload, and also its variations, is known to jeopardize the outcome of ICU patients. However, fluid balance remains difficult to assess accurately. In that context, our study aims to assess the prognostic value of body weight variations (BWV) from Day 3 to Day 7 on the 30-day mortality, length of stay (LOS) and the occurrence of ventilator-associated pneumonia (VAP) and bedsore in critically ill patients with shock.

**Patients and methods:** Adult patients admitted in ICU with shock between 2002 and 2012, and requiring mechanical ventilation during the first 48 h, were extracted from a prospective multicenter cohort for a retrospective analysis. BWV was defined as the difference between the body weight of the day of interest and the body weight on admission. Case mix, severity on admission, and outcomes were collected. Fine and Gray sub-distribution survival models were used, with ICU discharge as competing event, adjusted on comorbidity and illness severity at admission at each landmark, from Day 3 to Day 7. The impact of BWV on ICU stay duration was estimated through a multivariate negative binomial regression model.

**Results:** The median age and SAPS 2 score of the 2 374 included patients were 67 (IQR, 55–77) years and 53 (IQR, 41–65), respectively. The BWV increased from 0.4 kg (IQR, 0–4.8) on Day 3 to 3 kg (IQR, − 0.4 to 8.2) on Day 7. The 30 day in-hospital mortality, the ICU occurrence of bedsore and VAP were 27, 3 and 19.6%, respectively. Four categories of BWV were defined according to BWV interquartiles: weight loss, stable weight, moderate and severe weight gain. Categories of BWV were independently associated with death on Day 5 and Day 6 (Day 5 : sHR 1.27; 95% 0.99–1.63 p = 0.06; Day 6: sHR 1.43; 95% CI 1.08–1.89, p = 0.01) (Fig. 1). A weight loss tended to be associated with increased occurrence of bedsore, and weight gain with increased occurrence of VAP. The extent of BWV increased the duration of ICU stay independently of other severity factors.

**Fig. 1:**
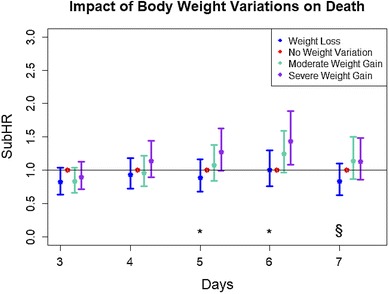
Impact of body weight variations on death

**Discussion:** BWV may be another clinically relevant tool to assess the risk of death, mostly after Day 4. The increased risk of bedsore in case of weight loss deserved to be confirmed.

**Conclusion:** Body weight should be daily monitored for better prognostication. BWV-based restrictive strategies should be further evaluated.

### F-70 The clinical effectiveness of multi-layer silicone dressings in preventing ICU acquired pressure ulcers: a randomised controlled trial

#### Santamaria Nick^1^

##### ^1^University de Melbourne, Melbourne, Australia

###### **Correspondence:** Santamaria Nick - n.santamaria@unimelb.edu.au

*Annals of Intensive Care* 2018, **8(Suppl 1):**F-70

**Introduction:** The development of pressure ulcers (PU) in critically ill ICU patients result in additional morbidity and may contribute to mortality in some cases. The minimisation of ICU acquired PU remain an international challenge. This paper describes Australian research that used multi-layer soft silicone sacral and heel dressings to prevent PU in critically ill patients.

**Patients and methods:** A total of 440 critically ill patients were enrolled into an 18-month randomised controlled trial in one of Melbourne’s trauma centres. Patients were randomised on admission to the Emergency Department and either had standard PU prevention or standard care plus the application of prophylactic sacral and heel dressings. Patients were observed daily for PU development for the duration of their ICU stay.

**Results:** Patients in the dressing group has significantly reduced incidence rate of PU development compared to patients receiving standard PU prevention alone (3.1 vs 13.1%, p < 0.03). Patients in the dressings group had a relative risk reduction of 80% and a 10% absolute risk reduction for developing a PU regardless of their critical illness. Results indicate the number needed to treat to prevent one PU was 10. Additionally, we calculated the cost–benefit of this intervention and found the patients treated with prophylactic dressings cost 3.6 time less than the standard care group for wound care.

**Discussion:** The use of prophylactic dressings to prevent PU at our hospital have proved to be very effective in ICU and subsequent studies have confirmed our results. It appears that the main mechanism of PU protection provided by these dressings is the reduction of pressure and shear forces leading to tissue distortion and cell death rather than the previously accepted ischaemic model of PU development. Our current policy is now to use these dressings on all patients with a high risk of developing PU.

**Conclusion:** The use of prophylactic multi-layer silicone dressings to prevent PU in critically ill patients is effective but it does not replace standard PU prevention methods. The use of these dressings sould be considered complimentary to best practice in PU prevention.

### F-71 Iatrogenic events in intensive care unit: incidence, risk factors and impact on outcome

#### Ayed Samia^1^, Merhebene Takoua^1^, Jamoussi Amira^1^, Ben Khelil Jalila^1^, Besbes Mohamed^1^

##### ^1^Hôpital Memmi, Ariana, Tunisia

###### **Correspondence:** Ayed Samia - samia.ayed@yahoo.fr

*Annals of Intensive Care* 2018, **8(Suppl 1):**F-71

**Introduction:** Iatrogenic events (IEs) are defined as harm resulting from medical intervention and health care, and not explained by underlying disease. Mortality is reported to be as high as 13.6% in cohorts of hospitalized patients experiencing IE. Both length of stay and cost of hospitalization are increased by IEs occurrence. We perform this study to determine the incidence, risk factors, and impact on outcome of IEs in intensive care unit (ICU).

**Patients and methods:** All patients admitted more than 24 h to the 22-bed ICU of a teaching hospital were prospectively screened. Patients were monitored daily for adverse clinical occurrences. Time and data about each IE were collected and they were considered as preventable or life-threatening events. For each patient, the followings were recorded—basic demographic data, indication for admission, severity scores on admission (SAPSII and APACHEII), need and duration of mechanical ventilation (MV), length of stay (LOS) in ICU, intensive care work load score (OMEGA), global mortality and IEs related mortality.

**Results:** During the 6 months period, 167 patients were included and 65 (38.9%) were judged to have developed an IE while hospitalized. We recorded 282 IEs over 2004 days in ICU so a density incidence of 14 IE for 100 patient-day. IEs were considered preventable in 34% of cases and life-threatening in 22% of cases. IEs occurred in a mean delay of 6 ± 6 days. Global mortality rate was 36.5% and IEs related mortality rate was 13.7%. Patients with IEs were significantly severe on admission, with a longer duration of MV and LOS in ICU. OMEGA score was significantly higher. Multivariate analysis showed that OMEGA score was the independent risk factor of IEs occurrence (OR 1.031 IC 95% [1.015–1.047], p < 10^–3^). Dead patients were significantly severe on admission and experienced more IEs than survivors. OMEGA score, duration of MV and LOS were significantly higher. In multivariate analysis, IEs and life-threatening IEs were independent factors of mortality (OR 6.171 IC 95% [3.087–12.339], p < 10^–3^ and OR 21.482 IC 95% [5.077–90.889], p < 10^–3^ respectively).

**Conclusion:** IEs in ICU are common and frequent but one-third is preventable. Work load ICU score is the independent risk factor of their occurrence. IEs impact largely the outcome especially the life-threatening ones. Efforts must be focused on preventing programs to reduce IEs and improve the outcome.

### F-72 Determinants of initial hyperlactatemia and early clearance of lactate in septic shock

#### Llitjos Jean-François^1^, De Roquetaillade Charles^1^, Jamme Matthieu^1^, Charpentier Julien^1^, Cariou Alain^1^, Chiche Jean-Daniel^1^, Mira Jean-Paul^1^, Pene Frederic^1^

##### ^1^Hôpital Cochin, Paris, France

###### **Correspondence:** Llitjos Jean-François - jllitjos@gmail.com

*Annals of Intensive Care* 2018, **8(Suppl 1):**F-72

**Introduction:** Based on the recent SEPSIS-3 definitions, septic shock is defined by the combination of vasopressor requirement and serum lactate level > 2 mmol/L. However hyperlactatemia and lactate kinetics may result from both increased production and impaired clearance in the critically ill, and may therefore not only rely on the severity of circulatory failure. We herein addressed the determinants of hyperlactatemia (> 2 mmol/L) and the factors likely to impact on early lactate clearance in septic shock.

**Patients and methods:** This was a 9-year (2008–2016) monocentric retrospective study. All adult patients diagnosed for septic shock within the first 48 h were included. Septic shock was defined as a microbiologically proven or clinically suspected infection, associated with acute circulatory failure requiring vasopressors. The first lactate value (L1) was measured at the time of ICU admission. Hyperlactatemia was defined as a first lactate level > 2 mmol/L. The second value (L2) was measured within 12 h following the first measurement. Lactate clearance was calculated as (L1-L2) L1 time between L1 and L2 measurements) and expressed in mmol hour. Parameters associated with initial hyperlactatemia and lactate clearance were investigated using multivariate logistic regression analysis.

**Results:** Among 938 patients admitted for septic shock, 438 (46%) patients exhibited hyperlactatemia > 2 mmol/L at ICU admission. Patients with initial hyperlactatemia had a higher in-ICU mortality rate (41.3% versus 32.6%, p = 0.007). In multivariate analysis, hyperlactatemia was independently associated with bacteremia (OR 1.64, CI 95% [1.19–2.27], p = 0.002) and prior beta-2 adrenergic agonist therapy (OR 0.32, CI 95% [0.15–0.67], p = 0.003). In multivariate analysis adjusted with severity, early lactate clearance (i.e. decrease in lactate levels within the first 12 h) was independently associated with the following variables—leucopenia at admission (OR 0.53, CI 95% [0.32–0.89], p = 0.016), initial lactate value (OR 1.28, CI 95% [1.20–1.36], p < 0.001), arterial oxygen content (OR 1.05, CI 95% [1.01–1.10], p = 0.009), concurrent fluid balance (per liter) (OR 0.91, CI 95% [0.85–0.97], p = 0.005).

**Conclusion:** Optimisation of the arterial oxygen content to improve tissue oxygenation represents a major therapeutic goal in septic shock. Whether the amount of fluid infusion may have harmful effects on lactate clearance by itself or is a surrogate marker of severity deserves further investigation.

### F-73 Neutrophil function is not defective after cardiopulmonary bypass

#### Lesouhaitier Mathieu^1^, Gregoire Murielle^1^, Uhel Fabrice^1^, Gacouin Arnaud^1^, Le Tulzo Yves^1^, Flecher Erwan^1^, Tarte Karin^1^, Tadie Jean-Marc^1^

##### ^1^CHU Rennes, Rennes, France

###### **Correspondence:** Lesouhaitier Mathieu - mathieu.lesouhaitier@gmail.com

*Annals of Intensive Care* 2018, **8(Suppl 1):**F-73

**Introduction:** Cardiac surgery with cardiopulmonary bypass (CPB) induces immunosuppression which has considerable implications for patients. CPB induces a significant increase in circulating neutrophils. Neutrophil activation, associated with production of antibacterial peptides, reactive oxygen species (ROS), cytokines, and other inflammatory mediators, as well as release of DNA into the extracellular milieu (Neutrophil extracellular traps (NETs)), plays a central role in innate host defense and modulation of inflammation. However, it has been shown that, in septic shock or systemic inflammation as major surgery, immature circulating neutrophils can induce immunosuppression and increase the risk of secondary infections. Staphylococcus aureus (SA) is one of the most commonly encountered bacterial pathogen responsible for poststernotomy mediastinitis, and neutrophils alterations may favor postoperative infections. The main objectives of this study were to evaluate the direct effects of CBP on neutrophils functions and to study the impact of different strains of SA on neutrophils bactericidal functions.

**Patients and methods:** Blood samples were collected before and 24 h after cardiac surgery with CPB and bone marrow samples were harvested directly after sternotomy, before initiation of CPB, and at the end of CPB, before sternal closure. Septic patients were included as controls. Circulating neutrophils analysis was performed using flow cytometry. We also studied NETosis, ROS production and bactericidal activity in isolated neutrophils before and after surgery using two strains of SA—one responsible of postoperative mediastinitis and one isolated from nasal carriage.

**Results:** Blood cell count with differential demonstrated a significant increase in neutrophils 24 h after surgery. Flow cytometry analysis of blood samples indicated neutrophils were matures with a significant increase in degranulation marker (CD66b). Neutrophils life span was also increased after CBP. Flow cytometry analysis of bone marrow samples showed no difference in cell composition and maturation before and after CBP. The neutrophil production of ROS was significantly higher after CBP. However, CBP did not impact NETs formation, phagocytosis and bactericidial function. Moreover, there was no difference regarding the phagocytosis and the bactericidial activity when exposed to the two strain of SA. As expected, immature neutrophils count was significantly increased in septic patients compared to cardiac surgery patients.

**Conclusion:** These results indicate that CBP promotes the recruitment of matures neutrophils via a demargination process. CBP does not induce neutrophil dysfunction. Neutrophils should not be targeted to decrease postoperative infection after CPB.

### F-74 A study of the correlation between Protein Tyrosin Phosphatase-1B gene expression and organ failure during septic shock

#### Beuzelin Marion^1^, Grangé Steven^1^, Clavier Thomas^1^, Girault Christophe^1^, Beduneau Gaëtan^1^, Carpentier Dorothée^1^, Artaud-Macari Elise^1^, Hobeika Sinad^1^, Lemaitre Caroline^1^, Enguerrand Pauline^1^, Tamion Fabienne^1^

##### ^1^CHU Charles Nicolle, Rouen, France

###### **Correspondence:** Beuzelin Marion - marion.beuzelin@gmail.com

*Annals of Intensive Care* 2018, **8(Suppl 1):**F-74

**Introduction:** Protein Tyrosine Phosphatase 1B (PTP1B) is a negative regulator of both NO production and insulin signaling and has been shown to be an aggravating factor in septic shock. Stress hyperglycemia frequently occurs in critically ill patients and is associated with poor outcome. Experimental studies on transgenic mice have shown that PTP1B deletion resulted in a reduced insulin resistance and in a better survival during experimental model of sepsis. The main objective was to study the correlation between the PTP1B gene expression and organ failure (through the delta SOFA score between day 1 and day 5) or insulin resistance.

**Patients and methods:** Preliminary monocentric study including patients admitted for septic shock. Patient’s characteristics, medical history, blood tests, etiology of ICU admission and number of organ dysfunction were collected and the blood samples were taken between D1 and D5 to analyse PTP1B ARN’s expression.

**Results:** Forty-seven patients were included. No link was found between PTP1B expression and the delta SOFA score between day 1 and 5 (Spearman correlation: − 0.088 [− 0.40 + 0.41]; p = 0.60). Level of PTP1B gene significantly decreased during the 5 first days (1.4 [0.90–1.70] versus 0.81 [0.71–1.00], p < 0.0001). Patients with higher expression of PTP1B gene did not show more insulin resistance. The PTP1B gene expression was linked to the cumulated doses of Norepinephrine given at day 1 (Spearman correlation—0.33 [0.036 + 0.58]; p = 0.025) and during the 5 first days (Spearman correlation—0.32 [0.028 + 0.56] + p = 0.028), and to the lactate level at day 1 (Spearman correlation—0.47 [0.20 + 0.88] p = 0.0012).

**Conclusion:** No correlation was found between PTP1B gene expression and organ failure (through the day 1 and day 5 delta SOFA score) or insulin resistance in septic shock patients. PTP1B gene expression seems to be linked to septic endothelial dysfunction and further studies are necessary to explain more the role of PTP1B expression in sepsis

### F-75 Characterization of the safety and pharmacokinetic profile of a novel TREM-1 pathway regulating peptide (MOTREM) during a phase I, randomised, placebo controlled, first in human clinical trial

#### Salcedo Margarita^1^, Cuvier Valerie^1^, Lorch Ulrike^2^, Witte Stephan^1^, Olivier Aurélie^1^, Gibot Sebastien^1^, Delor Isabelle^1^, Garaud Jean-Jacques^1^, Derive Marc^1^

##### ^1^INOTREM SA, Paris, France; ^2^Richmond Pharmacology, London, UK

###### **Correspondence:** Salcedo Margarita - msm@inotrem.com

*Annals of Intensive Care* 2018, **8(Suppl 1):**F-75

**Introduction:** MOTREM is the clinically formulated synthetic peptide (LR12) that is currently being developed for the treatment of sepsis and for the prevention of sequelae after acute myocardial infarction. Preclinical experiments have demonstrated that LR12 is able to modulate inflammatory responses and prevent their deleterious consequences in various animal models at a pharmacologically active dose of 1 mg kg h. Its pharmacological action occurs via the Triggering Receptor Expressed on Myeloid Cells-1 (TREM-1) signalling pathway. MOTREM was safe and well tolerated in GLP toxicology studies.

**Patients and methods:** Twenty-seven healthy male volunteers have been included in this clinical trial. The product was administered by continuous intravenous infusion (CIV). A single ascending dose design with 8 dose levels was used. Cohorts 1 and 2 received a 15-min single dose of MOTREM (1 and 10 mg and one and two volunteers respectively). Then, cohorts 3 to 8 received either a 15-min loading dose (from 0.5 mg kg to 5 mg kg) followed by 7.75-hours maintenance dose (from 0.03 mg kg h to 6 mg kg h) of MOTREM or a matching placebo (3–1 ratio). All volunteers were carefully monitored. Before escalation to the next dose level, safety and PK data of the previous dose level were reviewed by a safety review committee. Since immune system is at rest in normal individuals and thus TREM-1 pathway is not activated, no pharmacodynamics parameters were analyzed. The main objectives of this trial was then to study the safety and pharmacokinetic profile of MOTREM.

**Results:** No product related changes in vital signs, clinical nor laboratory parameters were observed. No product-related adverse events were reported. The PK of MOTREM was linear; The main clearance was estimated at 463L/h/70 kg which is higher than the hepatic blood flow in human (i.e., 90L/h/70 kg) and is therefore indicative of an extensive enzymatic metabolism in blood + effective half-life was calculated to be about 3 min.

**Conclusion:** MOTREM was found to be safe and well tolerated up to the highest dose tested (5mg/kg for a 15-min loading dose and 6 mg kg h for a 7.75-hours maintenance dose). Safety and pharmacokinetics of MOTREM is currently being studied in septic shock patients in a phase IIa randomised, double-blind, two-stage, placebo controlled, international, multicenter clinical trial (www.clinicaltrials.gov NCT03158948).

### F-76 Targeted endothelial Trem-1 deletion protects mice during septic shock

#### Jolly Lucie^1^, Carrasco Kévin^1^, Derive Marc^1^, Boufenzer Amir^1^, Gibot Sébastien^2^

##### ^1^INOTREM, Vandoeuvres Les Nancy, France; ^2^CHU Nancy, Nancy, France

###### **Correspondence:** Jolly Lucie - lj@inotrem.com

*Annals of Intensive Care* 2018, **8(Suppl 1):**F-76

**Introduction:** TREM-1 (Triggering Receptor Expressed on Myeloid cells-1) is an immunoreceptor expressed on neutrophils and monocytes macrophages whose role is to amplify the inflammatory response driven by Toll-Like Receptors engagement. The pharmacological inhibition of TREM-1 confers protection in several pre-clinical models of acute inflammation. In this study, we aimed to decipher the role of TREM-1 on the endothelium.

**Patients and methods:** We evaluated the expression of Trem-1 in vessels and isolated endothelial cells by flow cytometry, qRT-PCR and confocal microscopy. We generated an endothelium-conditional Trem-1 ko mice and submitted them to polymicrobial sepsis through CLP. Organs and blood were harvested at different time points and analyzed for cellular content, cytokine chemokine concentrations, and vasoreactivity. Survival was monitored for 1 week.

**Results:** Trem-1 was expressed in aorta and pulmonary vessels from animals, and inducible after LPS stimulation or during sepsis. These results were confirmed in human pulmonary microvascular endothelial cells. The pharmacological inhibition of TREM-1, using the synthetic inhibitory peptide LR12, decreased the LPS-induced Trem-1 expression. Sepsis induced a profound vascular hyporeactivity in WT animals, both in terms of contractility and endothelium-dependent relaxation. Although contractility was still impaired in EndoTREM-1—mice, vasorelaxation was completely restored. Soluble TREM-1 concentrations, a marker of TREM-1 activation, were markedly increased in the plasma, the peritoneal lavage fluid and the lungs from WT septic mice compared to control. In EndoTREM-1—mice, sTREM-1 level was reduced. Plasma concentrations of soluble VCAM-1 and IL-6 were also reduced in EndoTREM-1—animals. We observed an accumulation of neutrophils and inflammatory Ly6Chigh monocytes in the lung of WT septic mice. This accumulation was dampened in EndoTrem-1—mice. By contrast, endothelial Trem-1 deletion favored the accumulation of reparative cells (Ly6Clow monocytes). Finally, survival was clearly improved in the EndoTrem-1—group as compared to the WT group.

**Conclusion:** We reported that TREM-1 is expressed and inducible in endothelial cells and plays a direct role in vascular inflammation and dysfunction. The targeted deletion of endothelial Trem-1 conferred protection during septic shock in modulating inflammatory cells mobilization and activation, restoring vasoreactivity and improving survival. The effect of TREM-1 on vascular tone, while impressive, deserves further investigations including the design of endothelium specific TREM-1 inhibitors.

### F-77 Thiamine status and lactate concentration in sepsis

#### Heming Nicholas^1^, Salah Amor^1^, Meng Paris^1^, Sivanandamoorthy Sivanthiny^1^, Annane Djillali^1^

##### ^1^Hôpital Raymond Poincaré, Garches, France

###### **Correspondence:** Heming Nicholas - nicholas.heming@aphp.fr

*Annals of Intensive Care* 2018, **8(Suppl 1):**F-77

**Introduction:** Thiamine is an essential co-factor for aerobic metabolism. Both sepsis and thiamine deficiency are associated with lactic acidosis and hypotension. We assessed in a single center observational study the relationship between thiamine and lactate concentrations as well as clinical outcomes in a cohort of sepsis patients.

**Patients and methods:** 28 patients, median age 60 [44–77.3] years, of which 15 (53.6%) were male, SAPS II 40 [27–50.5]. 14 patients suffered from pneumonia, 6 from intra-abdominal sepsis. We measured serum levels of total and free thiamine, thiamine mono di and triphosphate (TMP, TDP and TTP respectively), as well as the erythrocyte transketolase activity and arterial lactate at the time of admission. We also recorded the vital status at the end of the ICU stay.

**Results:** 50% of our subjects exhibited particularly low levels of free thiamine (< 7 nmol/L). There was no correlation between free (r = − 0.4; p = 0.85), or total (r = − 0.19; p = 0.34) thiamine concentration and lactate levels. There was no correlation between TMP (r = 0.02; p = 0.91), TDP (r = − 0.19; p = 0.34), TTP (r = − 0.15; p = 0.46) and lactate levels in the whole population. No correlation was found between the concentration of thiamine derivatives and arterial lactate levels in the subgroup of patients exhibiting the highest levels of lactate (> 2 and > 4 mmol/L). Total thiamine and TDP concentration at the time of admission were significantly higher in ICU survivors than in non-survivors (p = 0.031 and p = 0.028).

**Conclusion:** During sepsis, we did not find any correlation between thiamine and lactate concentration. Lower thiamine diphosphate concentration may be associated with ICU-mortality.

### F-78 Impact of sodium lactate perfusion in fluid balance and capillary leak in a rat model of endotoxin shock

#### Boyer Déborah^1^, Besnier Emmanuel^1^, Duburcq Thibault^2^, Favory Raphael^2^, Henry Jean-Paul^1^, Richard Vincent^1^, Tamion Fabienne^1^

##### ^1^Rouen, France; ^2^Lille, France

###### **Correspondence:** Boyer Déborah - deborah_bo@hotmail.com

*Annals of Intensive Care* 2018, **8(Suppl 1):**F-78

**Introduction:** A positive fluid balance in sepsis is a determining factor for mortality. In previous experimental studies, sodium lactate has been shown to improve hemodynamic and avoid fluid overload (1). To understand these beneficial effects, we investigated the impact of sodium lactate on capillary leakage, in comparaison to saline on capillary leak in a rat model.

**Patients and methods:** The sixteen sedated, mechanically ventilated rats were challenged with intravenous infusion of E.coli lipopolysaccharide (10 mg/kg). Two groups of eight animals were randomised to receive a continous perfusion (5 mL/kg/h) of sodium lactate 11.2% (treatment group) or 0.9% NaCl (control group). In order to inject the same caloric load in the two groups, a 4.35 mL/kg/h of either water of 10% dextrose solution were perfused. Mean arterial pressure, heart rate, urine ouput were measured over a 210 min period. An echocardiography was then performed and Evans Blue (1%, 30 mg/kg) was intravenously injected 30 min before sacrifice. Organs were withdrawn and organs wet dry ratio and Evans blue dye extravasation were measured.

**Results:** Fluid balance, organs wet dry ratio and Evans blue dye extravasation were not significantly improved in sodium lactate group. Hemodynamics parameters were not significantly enhanced after sodium lactate infusion.

**Discussion:** Previously, lactate administration has improves renal perfusion. In our study, the volume of urine output was decreased in the 2 groups reflecting the severity of our model. and the vascular filling (9.35 ml/kg/h) higher than in the literature could impact our results.

**Conclusion:** In our endotoxinic model, capillary leakage was not improved by sodium lactate infusion. Further studies are under way to evaluate the impact of lactate sodium in a less severe model (LPS 5 mg/kg), with a decreased vascular filling and in a polymicrobial model of sepsis by caecal ligature and puncture.

**Reference**

1. Duburcq T, Durand A, Dessein A-F, Vamecq J, Vienne J-C, Dobbelaere D, et al. Comparison of fluid balance and hemodynamic and metabolic effects of sodium lactate versus sodium bicarbonate versus 0.9% NaCl in porcine endotoxic shock: a randomized, open-label, controlled study. Crit Care. 2017 + 21(1).

### F-79 Photoplethysmographic determination of the respiratory rate in acutely ill patients

#### L’Her Erwan^1^, Bodenes Laetitia^1^, N’Guyen Quang-Thang^2^, Lellouche François^3^

##### ^1^CHU de la Cavale Blanche, Brest, France; ^2^Oxynov France, Plouzané, France; ^3^Centre de Recherche de l’IUCPQ, Québec, Canada

###### **Correspondence:** L’Her Erwan - erwan.lher@chu-brest.fr

*Annals of Intensive Care* 2018, **8(Suppl 1):**F-79

**Introduction:** Respiratory rate (RR) is among the first vital signs to change in deteriorating patients. Intermittent RR measurement via visual observation is routine and it is usually estimated by visual counts of the chest wall movements. This approach has several inherent limitations that diminish its clinical applicability. The pulse oximetry photoplethysmographic (PPG) signal monitoring is routine since the early 1980’. The PPG signal includes respiratory synchronous components, seen as frequency modulation of the heart rate, amplitude modulation of the cardiac pulse, and respiratory induced intensity variations in the PPG baseline. Several studies have attempted to extract RR from PPG recordings, however their majority have been undertaken in controlled settings, on small groups of healthy subjects, and or have included waveforms without artefacts. The main purpose of our study is to assess the validity of a new RR extraction algorithm.

**Patients and methods:** This study is derived from a datawharehousing project (ReaSTOC study, ClinicalTrials.gov identifier—NCT02893462), including all consecutive patients admitted to our ICU. Physiological tracings were recorded from the standard monitoring system (Intelliview MP70 Philips), using a dedicated network and extraction software (Synapse v1, LTSI INSERM U1099) that enables photoplethysmographic recordings at a native resolution of 125 Hz. All consecutive patients were recorded for a 2-hours period during the first 24-hours following admission. Physiological recordings were associated with metadata collection. The proposed method for RR estimation relies on analyzing frequency components of a PPG signal via its spectrum, and enhancing respiration-induced modulations by advanced statistical signal processing techniques.

**Results:** 202 patients were analyzed (140 male + age 60 ± 11 yr., SAPSII 51.5 ± 15.7). 26.2% had a history of chronic respiratory disorder + They were admitted for acute respiratory failure (41.6%), hemodynamic failure (28.2%), neurological failure (18.3%). 128 patients were under invasive mechanical ventilation (63.4%) and 72 patients were spontaneously breathing (35.6%). Due to a bad PPG signal and or artefacts, RR calculation was not possible in 9 patients (4.5%). Correlation of the algorithm RR values and RR gold standard are provided within the Fig. 1. Error was null and bias was low (− 3.3 and + 3.5 b min).

**Fig. 1:**
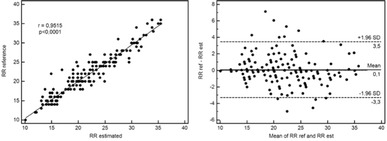
Results from photoplethysmographic determination of the respiratory rate (RR)

**Conclusion:** The proposed method for RR estimation is accurate and has a low error and bias, either during invasive mechanical ventilation and in spontaneously breathing patients. The versatility of pulse oximetry monitoring in various types of clinical settings offers new issues for RR monitoring.

### F-80 Muscular pressure under NAVA using the PEI index during weaning after acute respiratory failure

#### Maxime Denis^1^, Benjamin Repusseau^1^, Ouattara Alexandre^1^, Roze Hadrien^1^

##### ^1^CHU de Bordeaux, Bordeaux, France

###### **Correspondence:** Maxime Denis - maxime_denis@me.com

*Annals of Intensive Care* 2018, **8(Suppl 1):**F-80

**Introduction:** With NAVA assist is proportional and synchronized to diaphragmatic electrical activity (EAdi). EAdi represent the diaphragmatic neural drive. We titrate NAVA according the EAdi max recorded during a failed spontaneous breathing trial (SBT). After a failed SBT NAVA is started and we increase NAVA level in order to reduce the respiratory load until EAdi = 40% EAdi max. (1) Recently, the pressure Electricy index—Pmus Eadi index (PEI) has been described. (2) The purpose of this study was to assess muscular pressure (Pmus) using PEI with our NAVA protocol.

**Patients and methods:** Observational study, patients recovering from pneumonitis and acute respiratory failure. SBT was Pressure Support Ventilation with 7cmH20 of assist and no PEP. PEI was calculated under NAVA and during SBT from airway pressure drop during end-expiratory occlusions, muscular pressure (Pmus) was estimated from PEI (2). Another index, patient ventilator contribution index (PVBC) was also measured using the inspiratory peak of EAdi and Vt (inspiratory) during assisted and non-assisted breaths. We calculated PVBC-squared because it has been shown that it is more correlated to Pmus Ptot.

**Results:** Results are summarized in the Table. No patient had respiratory distress under NAVA. NAVA VT was higher than SBT VT—6.2 [4.7–6.7] vs 5.4 [4.8–6.7] ml.kg-1 PBW, p = 0.02. After the failed SBT the reduction of 40% of EAdi with NAVA reduced Pmus by 53%. Pmus, EAdi and Paw-PEP were not correlated and PEI during SBT was 22% higher than under NAVA, p = 0.013. The ratio Pmus (Pmus +Paw-PEP) under NAVA was 33% and PVBC-squared was 31%.

**Table:**
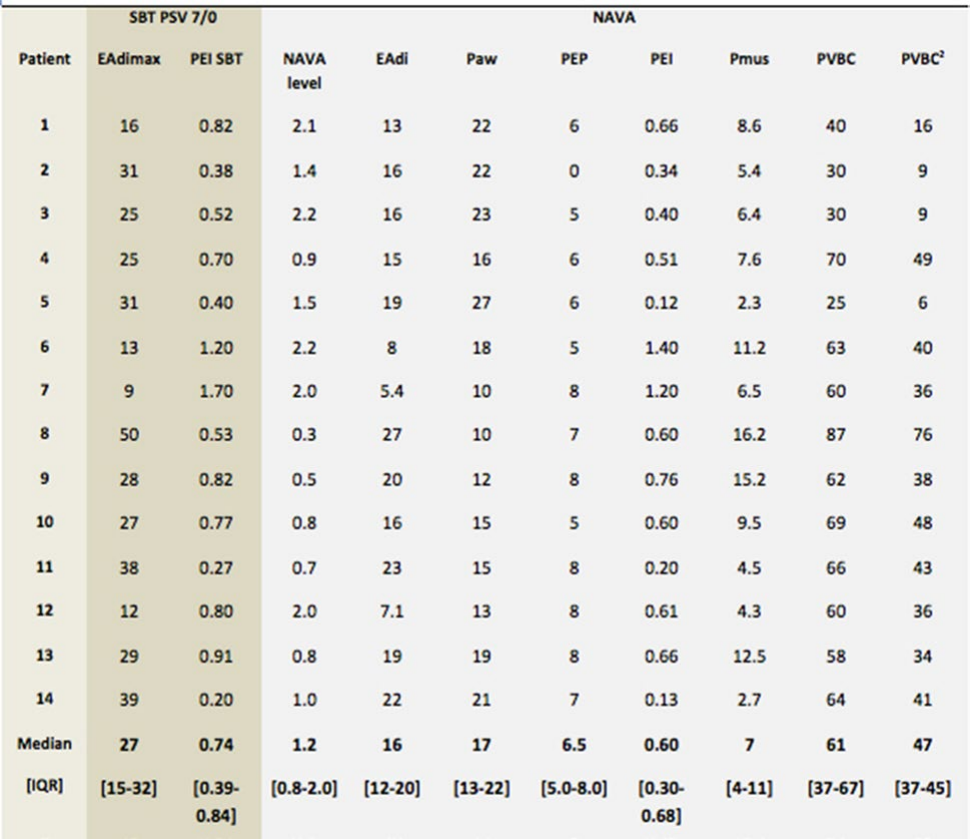

**Conclusion:** The reduction of 40% of the diaphragmatic neural drive according to our protocol reduced significantly the respiratory load within acceptable range of Pmus. Moreover PEI can increase when the load is too high, probably by accessory muscles recruitment.

**References**

1. Intensive Care Med. 2011 + 37–1087–94.
2. Crit Care Med. 2013 + 41–1483–91.

### F-81 What is the best patient position for intubation in intensive care unit: an ancillary study of the MACMAN trial

#### Lascarrou Jean-Baptiste^1^, Bailly Arthur^2^, Le Thuaut Aurelie^3^, Champigneulle Benoit^4^, Toufik Kamel^5^, Mercier Emmanuelle^6^, Ricard Jean-Damien^7^, Lemiale Virginie^8^, Colin Gwenhael^2^, Martin Maelle^3^, Helms Julie^9^, Reignier Jean^3^

##### ^1^CHU de Nantes, France; ^2^CHD Vendee, Nantes, France; ^3^CHU Nantes, France; ^4^HEGP, France; ^5^CHR Orleans, France; ^6^CHU Tours, France; ^7^Hopital Colombes, France; ^8^Hopital Saint Louis, France; ^9^CHU Strasbourg, Strasbourg, France

###### **Correspondence:** Lascarrou Jean-Baptiste - jeanbaptiste.lascarrou@chu-nantes.fr

*Annals of Intensive Care* 2018, **8(Suppl 1):**F-81

**Introduction:** In ICU, intubation is a high risk procedure associated with high morbidity. Despite procedure’s improvement with systematic application of fluid loading, early use of vasopressors and checklist use, morbidity remains high. First pass success is strongly correlated with adverse event occurrence. A recent study by Semler et al. concluded than “sniffing” position is better than “ramped” position to increase first pass success even the primary outcome prespecified—pulse pressure saturation was not different between the two groups. We conducted a post hoc analysis of the randomized clinical trial MacGrath Mac video laryngoscope or Macintosh laryngoscope for intubation in the intensive care unit (MACMAN) to determine the best position for intubation in the ICU.

**Patients and methods:** MACMAN was a multicentre, open-label, randomized controlled superiority trial. Consecutive patients requiring intubation were randomly allocated to either the McGRATH MAC videolaryngoscope or the Macintosh laryngoscope, with stratification by centre and operator experience. An only inclusion criterion was—“Patients must be admitted to an ICU and require mechanical ventilation through an endotracheal tube”. Patients were excluded if—contraindication to orotracheal intubation (e.g., unstable spinal lesion); insufficient time to include and randomize the patient (e.g., because of cardiac arrest); age < 18 years; pregnant or breastfeeding woman + correctional facility inmate; patient under guardianship + patient without health insurance; refusal of the patient or next of kin to participate in the study; previous enrolment in a clinical randomized trial with intubation as the primary end point (including previous inclusion in the present trial). Post-hoc analysis was performed to assess association between patient position (sniffing or supine) and first pass success. Between-groups baseline difference was adjusted for baseline covariates significantly associated with the group membership (p < 0.2).

**Results:**

**Table:** 

	OR [IC 95%]	AUC	Se	Sp
Failure of first pass	1.18 [0.43–3.22] p = 0.75	0.52 [0.40–0.63]	33.3 [13.3–59.0]	70.2 [64.9–75.1]
Bougie use at first pass	1.41 [0.31–6.47] p = 0.66	0.51 [0.44–0.59]	11.1 [1.4–34.7]	91.9 [88.4–94.6]
Duration of intubation > 3 min	1.01 [0.37–2.76] p = 0.99	0.50 [0.39–0.62]	33.3 [13.3–59.0]	66.9 [61.5–71.9]
DASH1-A	32.51 [4.27–247.39] p = 0.001	0.80 [0.74–0.86]	94.4 [72.7–99.9]	65.7 [60.3–70.8]

**Conclusion:** In the MACMAN study, sniffing position is not associate with better intubation metric success as compare to supine position. Further studies are warranted to determine the best patient position for intubation in the ICU.

### F-82 Automated oxygen titration (FreeO2) during acute exacerbation of COPD

#### Lellouche Francois^1^, Bouchard Pierre-Alexandre^1^, Morissette Martin^2^, L’Her Erwan^3^

##### ^1^IUCPQ, Québec, Canada; ^2^Oxynov, Québec, Canada; ^3^CHU la Cavale Blanche, Brest, France

###### **Correspondence:** Lellouche Francois - francois.lellouche@criucpq.ulaval.ca

*Annals of Intensive Care* 2018, **8(Suppl 1):**F-82

**Introduction:** During acute exacerbation of COPD oxygen should be titrated to avoid both hypoxemia and hyperoxia. The recommendations are not followed and automated oxygen titration may be useful in this population. The aim of this study was to evaluate a new device developed to automatically titrate oxygen based on SpO2 target (FreeO2, Oxynov, Canada) and to compare oxygenation parameters with usual administration (manual flowmeter).

**Patients and methods:** The study is an observational monocentric study. We prospectively included patients hospitalized for acute exacerbation of COPD receiving oxygen. Written informed consent was obtained from all patient. In the first part of the study, we evaluated oxygen flowrate and SpO2 during 30 min at baseline based on management of the physicians in charge. The oxygenation parameters were compared with automated titration (FreeO2 during 1 h). In the second part of the study, oxygen was delivered with FreeO2 until oxygen weaning or a maximum of 72 h. We evaluated the oxygenation parameters during prolonged utilization, the duration of oxygen administration, a new Bluetooth SpO2 connection compared to wire SpO2 connection (evaluated by Visual Analog Scale 0–10).

**Results:** We present preliminary data of 23 COPD patients (sex ratio M F = 9 14). Mean age (± SD) was 72 ± 11 years, mean FEV1 (± SD) was 0.9 ± 0.3L. Oxygenation data in both parts of the study are displayed in the Table 1. Time in the SpO2 target was significantly increased with FreeO2 in comparison with manual titration and oxygen flowrate was reduced by half. In the second part of the study, the  % of time in the SpO2 target with automated oxygen titration was above 80% and time with hypoxemia and with hyperoxia were low. In 11 patients, we compared comfort with wire SpO2 connection to Bluetooth wireless SpO2 connection. The comfort was significantly increased with wireless connection (7.0 ± 2.9 vs. 9.7 ± 0.6, p < 0.001). Duration of oxygen administration after inclusion (2.3 ± 4.3 days) and hospital length of stay after inclusion (5.7 ± 5.3 days).

**Table:**
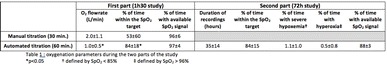

**Conclusion:** Automated oxygen titration maintains the patients within predetermined SpO2 target more than 80% of the time and reduces oxygen flowrate in comparison with manual oxygen titration. The second part of the study demonstrates the feasibility to use automated oxygen titration during several days with similar outcomes as previously reported in similar population. There are several limitations of the study and additional evaluations of this device are required.

### F-83 Continuous control of arterial oxygenation in mechanical ventilated patients

#### Mechati Malika^1^

##### ^1^CH Sainte Musse, Toulon, France

###### **Correspondence:** Mechati Malika - malikamechati@hotmail.fr

*Annals of Intensive Care* 2018, **8(Suppl 1):**F-83

**Introduction:** Hyperoxemia occurs up to 50% of mechanical ventilation days in the ICU [1] and is associated with increased mortality as compared to patients ventilated in normoxemia [2]. INTELLiVENT-ASV is a full closed loop ventilation mode adjusting automatically oxygenation’s settings FiO2 and PEEP according to SpO2 for passive and spontaneously breathing mechanically ventilated patients. This post hoc analysis of a monocentric randomized controlled parallel group study compared frequency of hyperoxemia (PaO2 > 120 mmHg and or SpO2 > 96%) and hypoxemia (PaO2 < 60 mmHg and or SpO2 < 90%) and the percentage of ventilation time with SpO2 > 96% and the percentage of ventilation time with SpO2 < 88% between INTELLiVENT-ASV and conventional ventilation mode in mechanically ventilated ICU patients.

**Patients and methods:** The randomized controlled trial was performed in the general ICU of Hôpital Sainte Musse, Toulon, France. Eligible participants were adult aged 18 or over, invasively ventilated for less than 24 h at the time of inclusion with an expected duration of mechanical ventilation of more than 48 h. Exclusion criteria were broncho-pleural fistula, ventilation drive disorder and moribund patients. Patients were allocated to INTELLiVENT-ASV group or to conventional ventilation group (volume assist control and pressure support modes) using blocked randomization. The post hoc analysis was performed by the comparison of all arterial blood gases (ABG) performed during the study period—the number of ABG with hyperoxemia and hypoxemia, the median PaO2 and SpO2 for these arterial blood gases and FiO2 associated were compared according to group.

**Results:** 60 patients were included, 30 patients in each group. The total number od ABG was 333 (mode conventional) vs 316 (mode INTELLiVENT-ASV) (p = ns). The number of ABG with PaO2 > 120 mmHg was respectively 54 versus 33 (p = 0.032) with SaO2 > 96% was 203 vs 153 (p = 0.001) with PaO2 < 60 mmHg was 57 vs 56 (p = 0.121) + with Sao2 < 90% was 88 vs 86 (p = 0.951). The percentage of time of ventilation spent with Spo2 > 96% was 32% vs 29 (p = 0.438), and with Sao2 < 88% was 0.6 vs 0.5 (p = 0.615).
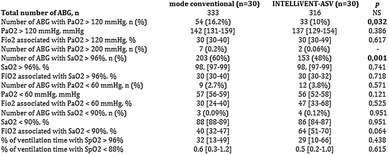


**Conclusion:** The continuous control of oxygenation settings provided by INTELLiVENT-ASV decreases significantly the number of blood gas with hyperoxemia as compared to manual oxygenation setting without increasing the risk of hypoxemia.

**References**

1. Suzuki et al. J Crit Care 28(5)—647–654.
2. Girardis et al. JAMA 316(15)—1583 to 1589.

### F-84 The Mechanical Ventilation: Respiratory Distress Observation Scale (MV-RDOS) as a surrogate of self-reported dyspnea in intubated patients

#### Decavèle Maxens^1^, Gay Frédérick^1^, Persichini Romain^1^, Morélot-Panzini Capucine^1^, Similowski Thomas^1^, Demoule Alexandre^1^

##### ^1^Hôpital Pitié-Salpêtrière, Paris, France

###### **Correspondence:** Decavèle Maxens - maxencesar@hotmail.fr

*Annals of Intensive Care* 2018, **8(Suppl 1):**F-84

**Introduction:** In invasively mechanically ventilated patient, dyspnea is frequent and severe. Relying on self-report, its measurement remains challenging in patients unable to communicate. A 5-item observation scale, namely the intensive care—respiratory distress observation scale (IC-RDOS), has been proposed as a surrogate of dyspnea-visual analogic scale (D-VAS) self-report in intensive care unit (ICU) patients [1]. However this scale has been validated among non-intubated patients and included one item “supplemental oxygen” not thoroughly adapted for intubated population. We sought to develop a dyspnea observation scale more suitable for intubated patients and to evaluate its performance to detect dyspnea.

**Patients and methods:** Ancillary analysis of data prospectively collected from 220 ICU communicative patients enrolled for the validation of the IC-RDOS. Factorial principal component analysis was first performed to select variables that mostly contributed to the principal axes, among a set of 21 observable variables with possible clinical relevance. To identify the best correlation between these variables and D-VAS, were performed an iterative partial least square regression process (PLS).

**Results:** Iterative PLS procedure identified five variables, of which the combination and weighting allowed optimal correlation with D-VAS (r = 0.61; 95% CI 0.50 to 0.72; *p* value < 0.0001), which constitute the IC-RDOS [1]. In a first step, we removed “supplemental oxygen”, not relevant in intubated patients. We obtained a 4-items IC-RDOS (r = 0.47, 95% CI [0.32 + 0.60], p < 0.0001) of which a value of 2.2 predicted a D-VAS > 3 with 48.1% sensitivity and a 93.9% specificity (areas under receiver operating characteristic curves (AUC = 0.763; 95% CI [0.679 + 0.848]). In a second step, to improve these performances, we added the item “respiratory rate”. Further iterations, led to a new 5-item optimized correlate of D-VAS (r = 0.47, 95% CI [0.36 + 0.57], p < 0.0001) with the algorithm described in Fig. 1b, which constitute the mechanical ventilation—respiratory distress observation scale (MV-RDOS). The Fig. 1a shows the AUC for the 4-items IC-RDOS and the MV-RDOS according to the D-VAS thresholds. A MV-RDOS of 2.3 predicted a D-VAS > 3 with 72% sensitivity and a 74% specificity (AUC = 0.766, 95% CI [0.679 + 0.852]).

**Fig. 1:**
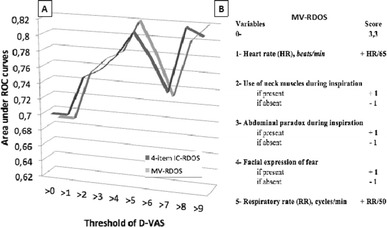
**a** Comparison of the areas under the receiver operating characteristic (ROC) curves between the 4-items intensive care—respiratory distress observation scale (IC-RDOS 4, former IC-RDOS [1] without respiratory rate) and the mechanical ventilation—respiratory distress observation scale (MV-RDOS) according to the dyspnea visual analog scale (D-VAS) thresholds. **b** Calculation of the MV-RDOS

**Conclusion:** These results suggest that MV-RDOS could be of value to identify dyspnea in intubated ICU patients.

**Reference**

1. Persichini R, Gay F, Schmidt M, Mayaux J, Demoule A, Morélot-Panzini C, Similowski T. Diagnostic Accuracy of Respiratory Distress Observation Scales as Surrogates of Dyspnea Self-report in Intensive Care Unit Patients. Anesthesiology. 2015 Oct; 123(4):830–7.

### F-85 Lung ultrasound in the diagnosis of Ventilator-associated pneumonia in intensive care medicine. Study LUVAP

#### Rezig Sofiane^1^, Marc Julien^1^, Brault Clement^1^, Zerbib Yoann^1^, Caplin Cecile^1^, Mercier Romain^1^, Verrier Nathalie^1^, Richecoeur Jack^1^, Slama Michel^1^, Maizel Julien^1^

##### ^1^CHU d’Amiens, France

###### **Correspondence:** Rezig Sofiane - zinou42@hotmail.com

*Annals of Intensive Care* 2018, **8(Suppl 1):**F-85

**Introduction:** Lung ultrasound (LUS) has emerged in different clinical settings, such as in intensive care medicine (ICM). Early diagnosis of ventilator-associated pneumonia (VAP) remains a challenge to the intensivist. However, scientific evidence is little available on whether LUS reliably improves the diagnosis of VAP. The aim of this prospective study was to assess whether LUS could be an alternative to pulmonary computerized Tomography (CT) for assessing diagnosis of VAP in ICM.

**Patients and methods:** Twenty-one patients ventilated for duration more than 5 days suspected of VAP were included. LUS was performed by a well-trained operator who was blinded of the VAP diagnosis. The diagnostic gold standard of VAP was on the basis of pulmonary CT and positive culture pulmonary. All clinical criteria for the diagnosis were collected the same day of LUS and pulmonary CT. The ultrasound exam included anterior, lateral and posterior views from both sides of the chest with superior and inferior views. We classed patient in 4 groups according diagnosis of VAP with pulmonary CT (VAP + or VAP-) and LUS (LUS + or LUS-). LUS characteristics of VAP diagnosis included 9 profils—asymetric line B (profil A B), without sliding (profil B’), sub pleural consolidation (profil C), consolidation with punctiforme bronchogram (PB), linear air bronchograms (LB) or dynamic bronchograms (LBD), posteror lateral alveolar pleural suffusion (PLAPS), pleural effusion pathological (PEP), shred sign (SS).

**Results:** Of 21 patients prospectively included, 17 (81%) were diagnosed VAP + . Of the 17 patients VAP + , 16 were VAP + LUS + (95%) and 1 was VAP + LUS—(5%). The positive rates for LUS + of the profil A B, profil B’, profil C, PB, LB, LBD, PLAPS, PEP and SS were respectively 34, 23, 14, 34, 25, 32, 21, 9, 45%. The overall accuracy, sensitivity and negative predictive value of LUS for VAP diagnosis were 85.8, 95.7 and 80.4% respectively. Specificity and positive predictive value of LUS for VAP diagnosis were 82 and 95.3% respectively. The area-under-the-ROC curve of the LUS for VAP diagnosis was 0.89 (95% IC 0.76–0.92), p = 0.0055.
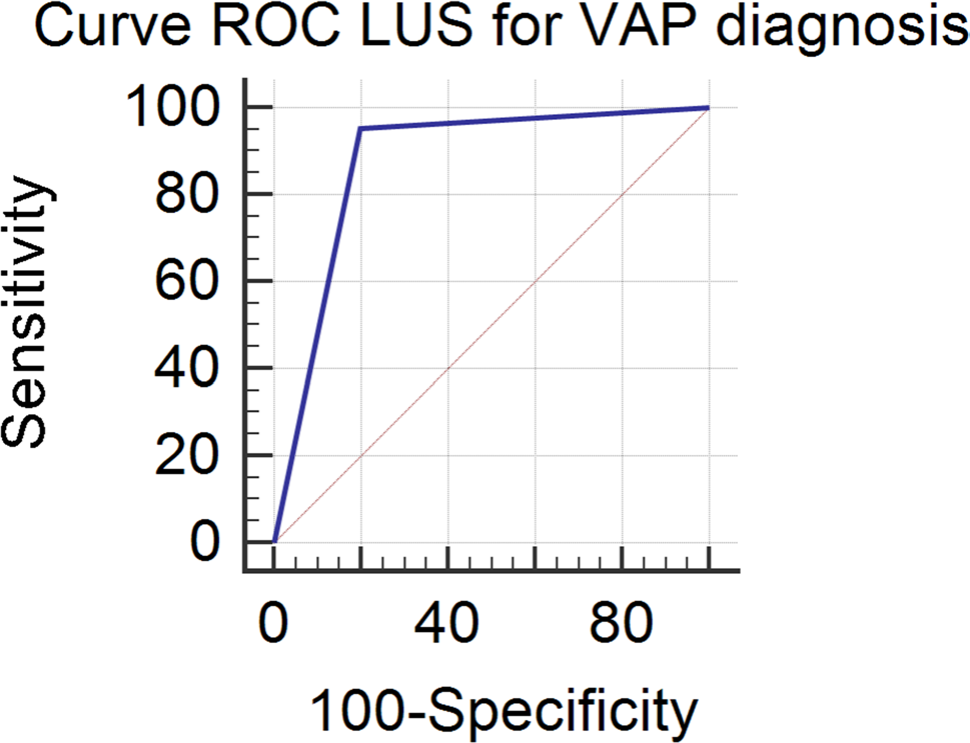


**Conclusion:** LUS is a sensitive diagnostic tool with which to identify VAP in critical ill patient. We suggest that LUS is a complementary tool to pulmonary CT in the diagnosis of VAP and that the follow up of VAP by LUS can reduce the need for accessing of pulmonary CT.

### F-86 Impact of onset time of acute kidney injury on outcomes in patients under veno-venous ECMO support: a prospective observational study

#### Zapetskaia Tatiana^1^, Conil Jean Marie^2^, Georges Bernard^2^, Bounes Fanny^2^, Seguin Thierry^2^, Crognier Laure^2^, Brouchet Laurent^1^, Silva Stein^2^, Minville Vincent^2^, Delmas Clement^2^

##### ^1^CHU de Tours, France; ^2^CHU de Toulouse, France

###### **Correspondence:** Zapetskaia Tatiana - zapetskaia@hotmail.fr

*Annals of Intensive Care* 2018, **8(Suppl 1):**F-86

**Introduction:** VV-ECMO is increasingly used for critically ill patients, such as those with life-threatening but reversible respiratory failure. In this context, acute kidney injury (AKI) is a major and frequent complication which seems associated with poor outcome. As far as risk factors for occurrence of AKI in patients under VV-ECMO, are poorly studied, we aimed to identify characteristics associated with severe AKI stage 3 KDIGO and the impact on 3 months’ outcome.

**Patients and methods:** We conducted a prospective, observational cohort study in our tertiary university hospital ICU (Toulouse, France) from May 2009 to April 2016. All adult patients undergoing VV-ECMO were included. Clinical and biological data were collected prior to ECMO implantation and during the hospitalization.

**Results:** Among 60 included patients, AKI occurred in 45 patients (75%)—31 patients (51%) developed AKI stage 3 and 26 patients (43%) required CRRT. Most of patients developed AKI stage 3 prior to ECMO insertion (41.4%). Instable hemodynamics, lower pH, higher lactate level, higher SOFA and modified SOFA scores, prior cardiopulmonary resuscitation or hemorrhagic shock were associated with AKI stage 3 occurrence. The rate of successful weaning from mechanical ventilation was initiatively lower in patients with AKI stage 3 (24 vs 68%; p = 0.0009). 23 (85%) patients had normal renal function at discharge. The 90-day mortality rate was significantly higher in patients with AKI stage 3 (72 vs 32%, p = 0.0023). Kaplan–Meier curves differed significantly for patients with AKI stage 3 versus patients without AKI (p = 0.012) (Fig. 1a) and for patients with AKI stage 3 prior to ECMO insertion versus patients without AKI (p = 0.004) (Fig. 1b). Multivariate logistic regression suggests, that AKI stage 3 prior to ECMO insertion was an independent risk for 90-days mortality.

**Fig. 1:**
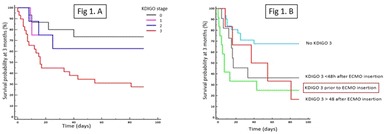
Impact of onset time of acute kidney injury on outcomes in patients

**Conclusion:** Incidence of severe AKI is correlated with global severity at admission and prior to ECMO insertion. In our cohort, AKI stage 3 KDIGO prior to ECMO insertion was negatively associated with survival.

### F-87 Vascular Access for Renal Replacement Therapy Among 459 Critically Ill Patients A pragmatic analysis of the randomized AKIKI trial

#### Gaudry Stéphane^1^, Benichou Nicolas^1^, Lebbah Saïd^2^, Hajage David^2^, Martin-Lefèvre Laurent^2^, Pons Bertrand^2^, Boulet Eric^2^, Boyer Alexandre^2^, Chevrel Guillaume^2^, Lerolle Nicolas^2^, Carpentier Dorothée^2^, De Prost Nicolas^2^, Lautrette Alexandre^2^, Anne Bretagnol^2^, Mayaux Julien^2^, Saad Nseir^2^, Frederique Schortgen^2^, Tubach Florence^2^, Ricard Jean-Damien^1^, Dreyfuss Didier^1^

##### ^1^Hôpital Louis Mourier, Colombes, France; ^2^Hôpital Henri Mondor, Paris, France

###### **Correspondence:** Gaudry Stéphane - stephanegaudry@gmail.com

*Annals of Intensive Care* 2018, **8(Suppl 1):**F-87

**Introduction:** Vascular access for acute renal replacement therapy (RRT) is routine question in the intensive care unit. Randomized trials comparing jugular and femoral sites have shown similar rate of nosocomial events and catheter dysfunction. Recent prospective observational data on RRT catheters use are scarce. We analyzed, in a large population of critically ill patients enrolled on a randomized trial on RRT initiation strategies, RRT catheter site, cause of catheters changes and complications according to insertion site. The advantage of this method is that it gives a pragmatic view of the real clinical situation.

**Patients and methods:** Ancillary study of the AKIKI trial, an open pragmatic randomized controlled trial published in 2016, in which patients with severe acute kidney injury were randomly assigned to either an early or a delayed RRT initiation strategy. The present study involved all patients who underwent at least one RRT session. Number of RRT catheters, insertion sites, factors potentially associated with the choice of insertion site, duration of catheter use, reason for catheter replacement, and complications were prospectively collected.

**Results:** Among the 619 patients included in AKIKI, 462 received RRT at least once and 459 patients were finally included in the analysis (3 missing data), leading to a total of 598 RRT catheters. Femoral site was chosen preferentially (n = 319, 53%), followed by jugular site (n = 256, 43%) and subclavian site (n = 23, 4%). Investigating center was the sole factor significantly associated with the choice of insertion site in multivariate analysis (p = 0.01). Higher weight did not affect choice of insertion site. Mean duration of catheter use was 6.6 (+-5.0) days without difference according to site. Catheter dysfunction was the main reason for replacement (n = 61, 49%). Suspicion of infection led to replacement of many catheters (n = 40, 32%) but was actually seldom proven (n = 4, 3%). No mechanical complication (pneumothorax or hemothorax) occurred.

**Conclusion:** Femoral site was preferentially used in this observational study of RRT catheters in 31 French intensive care units. The choice of insertion site depended on investigating center habits. A high incidence of catheter infection suspicions that were eventually ruled out led to many undue replacement.

### F-88 Model based meta-analysis of vancomycin during renal replacement therapy: application to vancomycin pharmacokinetic during SLED in ICU patients

#### Claisse Guillaume^1^, Ollier Edouard^2^, Maillard Nicolas^1^, Mariat Christophe^1^

##### ^1^Saint-Priest En Jarez, France; ^2^INSERM, U1059, Saint-Priest En Jarez, France

###### **Correspondence:** Claisse Guillaume - claisse.guillaum@gmail.com

*Annals of Intensive Care* 2018, **8(Suppl 1):**F-88

**Introduction:** Sustained low-efficiency dialysis (SLED) has become a frequent renal replacement therapy (RRT) technique used in instable critically ill patients hospitalised in ICU. However, little is known about the effect of SLED on antibiotics clearance. Despite recent new antibiotics, vancomycin remains an important drug used to treat cocci-gram positive severe infections in ICU. Many studies have reported data regarding clearance of vancomycin during continuous and or intermittent RRT. However, only few data are available regarding vancomycin pharmacokinetic (PK) on SLED. Here, we realised a meta-analysis of vancomycin clearance during continuous and intermittent RRT to model vancomycin PK during SLED.

**Patients and methods:** We conducted a systematic review of published article on PK of vancomycin during RRT with high flux filters. The literature search was conducted from the PubMed database from its inception to August 2017. Studies were included if they were providing an estimation of RRT vancomycin clearance. A model based meta-analysis of RRT vancomycin clearance in function of RRT settings (blood, dialysate and ultrafiltration flow rate) was performed using a non-linear mixed effect model. To guide the choice of the dosing regiment, we used previously published population PK data to simulate the PK of vancomycin in ICU patient undergoing SLED. Vancomycin clearance was splited in two parts—(1) the residual body clearance and (2) the clearance due to SLED obtain from the model constructed by meta-analysis.

**Results:** A total of 28 articles were included in the study corresponding to 69 measurements of RRT vancomycin clearance (47 with Haemodialisys, 14 with Haemofiltration, 8 with Haemodiafiltration). The model described adequately the measurements with no apparent bias between observed and predicted clearance. Based on population PK simulations, following a 1.75 g vancomycin bolus (25 mg kg for a 70 kg patient), time to pass below a threshold of 15 µg l was estimated to 5.87 h [2.83–12.7] (mean, 90%CI) for patient undergoing SLED (started 1 h after first administration) and to 19.21 h [4.37–120 +] for whose who not. At the end of SLED, only 4.5% of patients would have a vancomycin concentration above 15 µg L whereas 76% of them would be above this threshold with a second bolus administrated 6 h after the first one (Fig. 1).

**Fig. 1:**
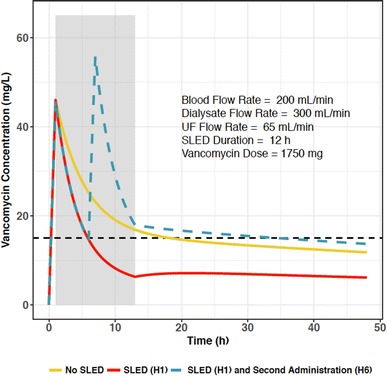
Vancomycin PK during SLED

**Conclusion:** Vancomycin is clearly eliminated by SLED and an administration of a standard unique dose (25 mg kg) may not allowed to stay above the therapeutic threshold.

### F-89 Relative blood volume monitoring predicts the variation of mean arterial pressure during long standing dialysis in ICU

#### Chraka Hayate^1^, Claisse Guillaume^1^, Alamartine Eric^1^, Mariat Christophe^1^, Maillard Nicolas^1^

##### ^1^CHU Saint etienne, Saint Etienne, France

###### **Correspondence:** Chraka Hayate - hayate.chraka@hotmail.fr

*Annals of Intensive Care* 2018, **8(Suppl 1):**F-89

**Introduction:** Long standing dialysis (SLED or CRRT) allows a better hemodynamic tolerance as well as a greater performance to achieve a negative fluid balance in intensive care unit. Dialysis alter hemodynamics mainly by short term variation of blood volume. In this study we took advantage of a continuous monitoring of blood volume during dialysis session to decipher the relationship between the variation of relative blood volume (RBV) with mean arterial pressure (MAP).

**Patients and methods:** This study is observational prospective, including all prolonged (> 8 h) dialysis sessions in Saint Etienne nephrology intensive care unit between January and June 2017. Exclusion criteria were ongoing blood transfusion and blood volume controled ultrafiltration. Medical records were compiled along with cardiac ultrasonography at the beginning when available. The statistical analysis was perfomed in two parts. The first part studied the performances of the first hour deltaRBV (defined by RBV before minus RBV after 1 h of dialysis) to predict a drop of MAP below 65 mmHg (hypotension). This analysis excluded sessions with hypotension and intervention during the first hour. The second study was the modelization of the relationship between deltaRBV and deltaMAP for every hour of dialysis without any intervention on blood pressure. Both analyses were performed using mixed effects linear and generalized models.

**Results:** A total of 82 sessions on 23 different patients were performed during the period. The characteristics of patients were as follows—sex ratio at 1, age (SD) 64.3 (9.0), weight 83.3 kg (17.2), SAPSII score 48.3 (13.6). 20 patients on 23 were taken in charge for fluid overload. In the first set of analyses (per sessions), 27 sessions were excluded for intervention in the first hour. The adjusted DeltaRBV did not predict hypotension during the session (generalized mixed effect model, session and patients set as random effects, estimate 0.14, p = 0.71). In the second set of analyses (per hour without any intervention), 535 h were analyzed. Adjusted DeltaRBV correlated strongly and inversly with deltaMAP (linear mixed effect model, random effects were sessions, patients and hour order in the session, estimate 1.42, p < 0.0001).

**Conclusion:** In our mostly fluid overloaded patients, the drop of RBV correlated with an increase of MAP.

### F-90 Renal and immunological outcomes in kidney-transplant recipients admitted in intensive care units

#### Guinault Damien^1^, Lavayssiere Laurence^1^, Nogier Marie-Béatrice^1^, Cointault Olivier^1^, Del Bello Arnaud^1^, Esposito Laure^1^, Hebral Anne-Laure^1^, Kamar Nassim^1^, Faguer Stanislas^2^

##### ^1^CHU de Toulouse, Toulouse, France, ^2^Hôpital Rangueil, 1 avenue Jean Poulhes, Toulouse, France

###### **Correspondence:** Guinault Damien - guinault.d@chu-toulouse.fr

*Annals of Intensive Care* 2018, **8(Suppl 1):**F-90

**Introduction:** Kidney transplant recipients (KTR) are at risk of ICU admission because of prolonged immunosuppressive therapy and a higher risk of cardiovascular events, severe infections or drug-related toxicities. Several retrospectives studies reported the short-term outcome of KTR admitted to the ICU, but data concerning the risk of chronic kidney disease and anti-HLA immunization are scarce.

**Patients and methods:** In this retrospective study, we addressed the in-hospital and long-term mortalities of the 200 KTR admitted in a French ICU (10 beds) between January 2010 and June 2016. Predictive factors for death, long-term renal function and HLA immunization were identified.

**Results:** The main causes for admission were acute respiratory failure (27.5%), sepsis (26.5%), post-operative period (peritonitis, hemorrhage + 23%). At the admission, mean age, SAPS2 and SOFA score were 58 ± 13 years, 52 ± 19 and 6.7 ± 3.3, respectively. Renal replacement therapy, mechanical ventilation and vasopressors were required in 103 (51.5%), 107 (53.5%) and 97 (48.5%) patients. Immunosuppressive regimen was modified in 155 patients (76.1% + steroids increase 68%, calcineurin inhibitors or antimetabolites withdrawal 25 and 37%, respectively). In-hospital mortality was 20% (30.1 and 38.2% at months 12 and 36). By multivariate analysis, EBV blood proliferation in the 6 months preceding the admission in the ICU, and the SAPS2 gravity score at admission independently predicted the in-hospital and long-term mortalities. Among the 113 patients alive at month 6 after the admission in the ICU and with available data, 34 (30.1%) and 51 (45.1%) progressed to a more severe CKD stage at months 1 and 6, respectively. Both, the severity of the AKI and the preexisting CKD predicted the risk of progression of the CKD. Last, de novo anti-HLA immunization at month 6 was identified in 18 119 patients (15.1%, donor specific antibodies 11 18 (61.1%)) and was significantly associated with the occurrence of acute transplant rejection (p = 0.002). In five patients who developed anti-HLA antibodies, RBC transfusion during the ICU stay was the only immunological trigger identified.

**Discussion:** Outcome of KTR is closed to the general population admitted in ICU and better than other immunocompromised patient, like patients from oncohematology.

**Conclusion:** Worsening of the renal function and HLA immunization are frequent and may impact mid to long-term prognosis because of the high risk of transplant rejection, end-stage renal disease and further transplantation contraindication.

### F-91 Frequency and prognostic impact of acute kidney injury in patients with pulmonary embolism, data from the RIETE registry—results of a retrospective study performed on a prospective multicenter multinational database

#### Murgier Martin^1^, Bertoletti Laurent^1^, Darmon Michael^1^, Reina Valle^2^, Del Toro Jorge^2^, Llamas Pilar^4^, Monreal Bosch Manuel^1^

##### ^1^Saint-Priest-En-Jarez, France; ^2^Hospital Sierrallana, Santender, Spain; ^3^Hospital General Universitario Gregorio Marañón, Madrid, Spain; ^4^Hospital Universitario Fundación Jiménez Díaz, Madrid, Spain

###### **Correspondence:** Murgier Martin - murgiermartin@gmail.com

*Annals of Intensive Care* 2018, **8(Suppl 1):**F-91

**Introduction:** Acute kidney injury (AKI) is associated with a poor prognosis. Although pulmonary embolism (PE) may promote AKI through renal congestion or hemodynamic instability, its frequency as its impact on the prognosis of patients with acute PE have been poorly studied.

**Patients and methods:** Using data from the Registro Informatizado de la Enfermedad TromboEmbolica venosa (RIETE) registry, we assessed the frequency of AKI at baseline, and its influence on the 30-day mortality rate of patients with objectively confirmed PE. AKI was defined according to the “Kidney Disease—Improving Global Outcomes” definition. We used multivariate analysis to assess whether or not the presence of AKI independently influenced the risk for 30-day death.

**Results:** The study included 21,131 patients with acute PE, of whom 6222 (29.5%) had AKI at baseline. Of these, 4385 patients (21%) were in stage 1, 1385 (6.5%) in stage 2 and 452 (2%) in stage 3. The proportion of patients with high-risk PE in those with no AKI, AKI stage 1, AKI stage 2 and AKI stage 3 was—2.8, 5.3, 8.8 and 12%, respectively (p < 0.001). After 30 days, 1236 patients (5.85%) had died. Overall mortality was—4% in patients with no AKI, 8.4% in AKI stage 1, 14% in AKI stage 2, 17% in AKI stage 3, all p < 0.001). On multivariable analysis, AKI was independently associated with an increased risk of death at 30 days (odds ratio = 1.25 + 95% CI 1.02–1.54), after adjusting for the initial severity of PE, age > 65 years, chronic heart failure or chronic lung disease, cancer, anemia and liver cirrhosis.

**Conclusion:** One in every 3–4 patients with acute PE had AKI. Moreover AKI was an independent predictor of poor outcome in PE patients. This study suggests that PE (and its severity) should be considered as a risk factor for AKI and AKI may deserve to be evaluated as a prognostic factor in patients with acute PE.

### F-92 Severe metabolic acidosis after out-of-hospital cardiac arrest: risk factors and relationship with outcome

#### Jamme Matthieu^1^, Ben Hadj Salem Omar^1^, Guillemet Lucie^1^, Dupland Pierre^1^, Bougouin Wulfran^1^, Charpentier Julien^1^, Mira Jean-Paul^1^, Pène Frédéric^1^, Dumas Florence^1^, Cariou Alain^1^, Géri Guillaume^2^

##### ^1^Hôpital Cochin (APHP), Paris, France; ^2^Hôpital Ambroise Paré (APHP), Boulogne Billancourt, France

###### **Correspondence:** Jamme Matthieu - mathieu.jamme@aphp.fr

*Annals of Intensive Care* 2018, **8(Suppl 1):**F-92

**Introduction:** Metabolic acidosis is frequently observed as a consequence of global ischemia–reperfusion after out-of-hospital cardiac arrest (OHCA). We aimed to identify risk factors and assessing the impact of metabolic acidosis on outcome after OHCA.

**Patients and methods:** We included all consecutive OHCA patients admitted between 2007 and 2012. Using admission data, metabolic acidosis was defined by a positive base deficit and was categorized by quartiles. Main outcome was survival at ICU discharge. Factors associated with acidosis severity and with main outcome were evaluated by linear and logistic regression, respectively.

**Results:** 826 patients (68.3% male, median age 61 years) were included in the analysis. Median base deficit was 8.8 [5.3, 13.2] mEq/l. Male gender (p = 0.002), resuscitation duration (p < 0.001), initial shockable rhythm (p < 0.001) and post-resuscitation shock (p < 0.001) were associated with a deeper acidosis. ICU mortality rate increased across base deficit quartiles (39.1, 59.2, 76.3 and 88.3%, p for trend < 0.001) and base deficit was independently associated with ICU mortality (p < 0.001). The proportion of CPC 1 patients among ICU survivors was similar across base deficit quartiles (72.8, 67.1, 70.5 and 62.5%, p = 0.21) and 11.7% of patients with a base deficit higher than 13.2 mEq l survived to ICU discharge with a good neurological recovery.
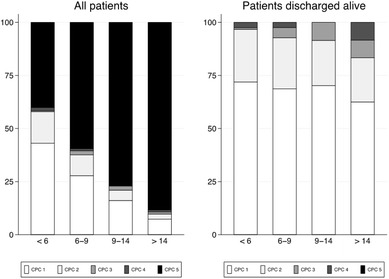


**Conclusion:** Severe metabolic acidosis is frequent in OHCA patients and is associated with poorer outcome, in particular due to refractory shock. However, we observed that about 10% of patients with a very severe metabolic acidosis survived to ICU discharge with a good neurological recovery.

### F-93 Influence of socio-economic status on initial severity and prognosis of patients admitted to intensive care: the IVOIRE study

#### Quenot Jean-Pierre^1^, Meziani Ferhat^2^, Meunier-Beillard Nicolas^1^, Capellier Gilles^3^, Bollaert Pierre-Edouard^4^, Louis Guillaume^5^, Dargent Auguste^1^, Levy Bruno^4^, Bein Christophe^6^, Ecarnot Fiona^3^, Rigaud Jean-Philippe^7^, Helms^2^, Julie, Fournel Isabelle^1^

##### ^1^Centre Hospitalier Universitaire, Dijon, France; ^2^Centre Hospitalier Universitaire, Strasbourg, France; ^3^Centre Hospitalier Universitaire, Besançon, France; ^4^Centre Hospitalier Universitaire, Nancy, France; ^5^Centre Hospitalier, Metz, France; ^6^Centre Hospitalier, Vesoul, France; ^7^Centre Hospitalier, Dieppe, France

###### **Correspondence:** Quenot Jean-Pierre - jean-pierre.quenot@chu-dijon.fr

*Annals of Intensive Care* 2018, **8(Suppl 1):**F-93

**Introduction:** Precarious socio-economic status can directly influence health, need for hospitalisation and mortality, according to a previous study performed in 22 European countries. Similar findings have been reported from Anglo-Saxon countries in the setting of intensive care. Due to the different structure of the healthcare system in France, we aimed to investigate whether socio-economic status influences initial severity of disease and 3 months mortality in patients admitted to intensive care in France.

**Patients and methods:** Prospective, multicentre, cohort study including 1396 adult patients admitted to one of 8 participating intensive care units (ICUs) between 2012 and 2016, and presenting failure of one or more major organs. Patients were considered to have a precarious socio-economic status if they presented at least one criterion of social vulnerability or a high EPICES deprivation score.

**Results:** Data on social vulnerability were available for 1281 patients, of whom 54.3% were considered to be socially vulnerable. Compared to non-vulnerable patients, socially vulnerable patients were younger (64.8 vs 67.2 years, p = 0.006), more frequently had chronic disease (18.0 vs 12.0%, p = 0.003 respectively for congestive heart failure and 26.8%vs 18.1%, p = 0.0002 for chronic respiratory disease), had higher levels of physical dependency (9.2 vs 4.7%, p = 0.002), and were more often classed as having long-term health conditions (67.1 vs 62.1%, p < 0.0001). Conversely, non-vulnerable patients had greater severity of disease at admission to the ICU than those classed as vulnerable, both in terms of SAPS II and SOFA scores (respectively 53.16 vs 50.09 (p = 0.001) and 8.35 vs 7.77(p = 0.002)). Findings were similar after adjusting for major confounders (adjusted odds ratio (OR) 0.62, 95% confidence interval (CI) [0.48–0.81], p = 0.0005). Mortality at 3 months was not significantly different between socially vulnerable patients and those not considered vulnerable, respectively 34.6 vs 36.10% (p = 0.62), even after adjustment for initial severity.

**Conclusion:** Despite less severe disease at admission to the ICU among patients considered socially vulnerable, 3-month mortality did not differ significantly between those who were socially vulnerable and those who were not. These findings suggest that the French healthcare system provides good protection for the most disadvantaged members of society, particularly when they are admitted to the ICU.

### F-94 Anonymous and continuous evaluation of the satisfaction of the critically ill patients’ proxies: the OpinionFamily tool

#### Labbe Vincent^1^, Marie Cécile De Crémiers^1^, Taconet Clémentine^1^, Pham Tài^1^, Djibre Michel^1^, Blayau Clarisse^1^, Voiriot Guillaume^1^, Garnier Marc^1^, Fulgencio Jean-Pierre^1^, Gounane Cherifa^1^, Gibelin Aude^1^, Flechel Anita^1^, Fartoukh Muriel^1^

##### ^1^Hôpital Tenon, Paris, France

###### **Correspondence:** Labbe Vincent - vincent.labbe@aphp.fr

*Annals of Intensive Care* 2018, **8(Suppl 1):**F-94

**Introduction:** An approach of the quality of care may involve assessing the patients’ satisfaction. However, the extended caregiver—patient and family relationship, specific to the critically ill patients, may also require to assess the proxies’ satisfaction. The OpinionFamily tool was developed to assess the satisfaction of the critically ill patients’ proxies, in an anonymous and continuous fashion.

**Patients and methods:** We conducted a study in the ICU of Tenon hospital (Paris, France) between Mars and August 2017. The OpinionFamily questionnaire, built with 6 categories (4 items each), aimed to measure the proxies’ satisfaction regarding their perception of the quality of care. All the proxies were invited to express voluntarily and anonymously his her degree of agreement as a response to a statement by the selection of the corresponding stars (strongly disagree—1 star, disagree—2 stars, neither agree nor disagree—3 stars, agree—4 stars, strongly agree—5 stars) using a secure touch screen disposed 24 7 in the waiting room of the ICU.

**Results:** Altogether, 456 patients were hospitalised during the study period, and 148 proxies completed the questionnaire. All the responders spoke French. Only 12 responders (9%) answered more than one time. Of the responders, 105 (71%) were the referring person, 47 (32%) were children and 26 (17%) were spouses. During the study period, 50 (34%), 59 (40%), and 38 (26%) responders had visited their relative 1 to 3 times, 4 to 10 times, and more than 10 times, respectively. The different categories assessed by the OpinionFamily tool were related to «the family and the patient» (Fig. 1A), «the family and the environment» (Fig. 1B), and «the family and the caregivers–availability, trust, support, and information» (Fig. 1C). The corresponding levels of satisfaction (responses of at least 4 stars) were respectively 67, 78, 65, 77, 78, and 79%. Some items were associated with a poor satisfaction (participation to the care, identification and availability of the caregivers).

**Fig. 1:**
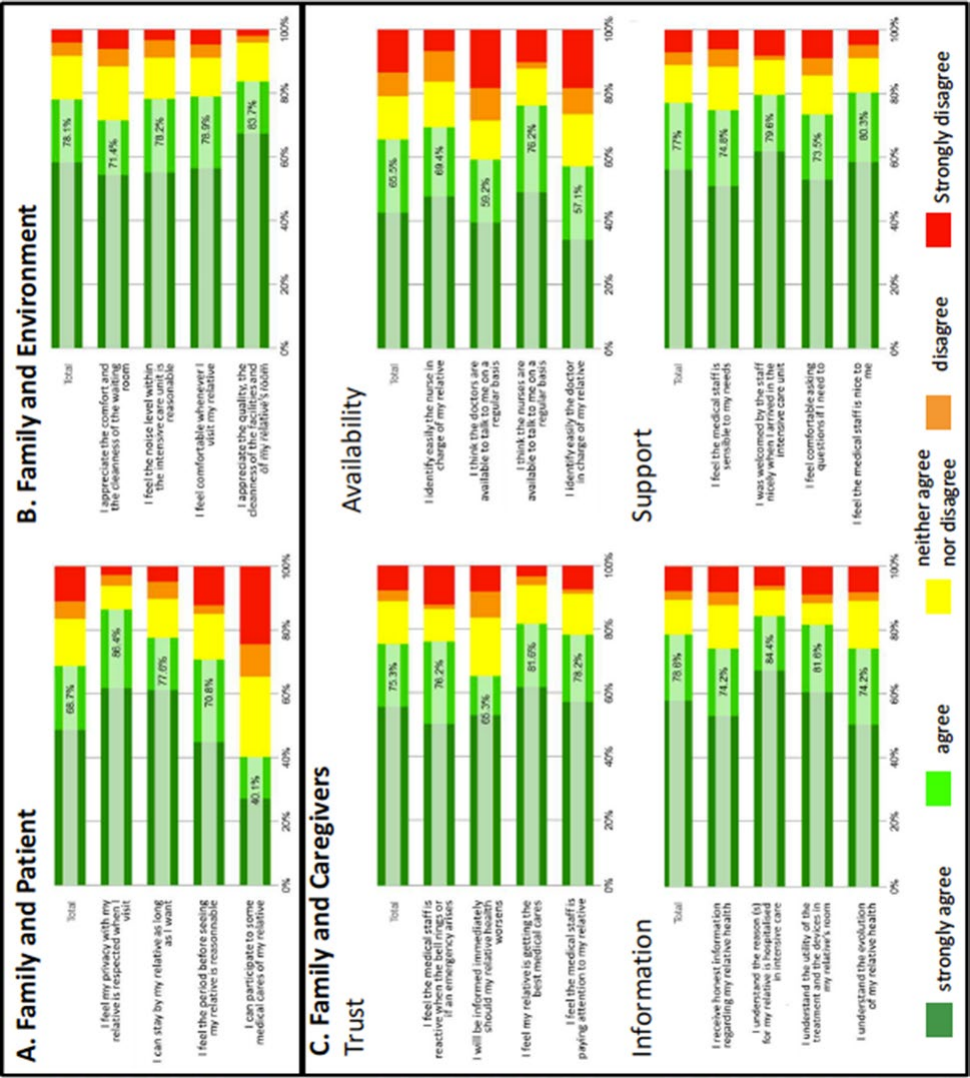
Questionnaire results

**Conclusion:** The implementation of the OpinionFamily tool allowed a continuous evaluation of the satisfaction of the critically ill patients’ proxies. A systematic implementation of this tool in the ICUs may be useful to the caregivers for a better understanding of the needs of the proxies. In addition, this tool may allow rapid changes in ICU organizations and behaviours to improve the proxies’ satisfaction, which may ultimately, improve the care of patients.

### F-95 Ethical decisions in patients with severe acute respiratory failure: determining factors, time of decision and impact on outcome: the eDecide project—an ancillary analysis of LungSafe study

#### Berleur Marie^1^, Pham Tai^2^, Ricard Jean-Damien^1^, Uchida Masatoshi^3^, Thomson Taylor^4^, Messika Jonathan^1^, Dreyfuss Didier^1^, Roux Damien^1^

##### ^1^Hôpital Louis Mourier, Colombes, France; ^2^Hôpital St Michael, Toronto, Canada; ^3^Dokkyo Medical University, JAPAN; ^4^Boston, USA

###### **Correspondence:** Berleur Marie - m.berleur@gmail.com

*Annals of Intensive Care* 2018, **8(Suppl 1):**F-95

**Introduction:** Withholding and withdrawing life-sustaining therapy (LST) are complex ethical decisions made in many critically ill patients. Many factors influence end-of-life decisions (EOL). We describe EOL decisions in patients with acute respiratory failure and their impact on patients’ prognosis.

**Patients and methods:** An international observational study included all patients with acute respiratory distress over a 1-month period. 459 ICU in 50 countries were involved. Demographic, clinical and biological data were compared between patients with and without decision of LST limitation. We also compared surviving patients after LST limitation decision to those who eventually died.

**Results:** Among the 4041 patients, mortality was 37.2%. A decision of LST limitation was reported in 948 patients (23.4%). In univariate analysis, patients with LST limitation decision were older and more frequently hospitalized for a medical condition, had a lower body weight, a higher SOFA score, and presented active neoplasia immunosuppression or chronic liver failure more frequently (p < 0.001 for all). Patients admitted after trauma, drug overdose or pulmonary contusion were less subject to have an LST limitation decision (p < 0.001). In contrast, patients with non-cardiogenic shock were more subject to these decisions (p = 0.018). EOL decisions were less frequent in lower-middle income countries as compared to high and middle-high income countries (p < 0.001). Multivariate analysis will be presented. Among 948 patients with an LST limitation decision, 148 survived (15.6%). Mortality was higher in this group than in the whole study population (p = 0.001). In univariate analysis, death after decision of LST limitation was associated with admission for a medical condition (p = 0.02), severe ARDS, higher inspiratory pressure, non-cardiogenic shock, higher SOFA score with or without respiratory component and chronic liver failure (p < = 0.001 for all). On the contrary, admission for trauma was associated with survival (p = 0.001). Regarding the 1503 patients who died during their hospital stay, 703 did not receive a decision of LST limitation (46.8%). Decision of LST limitation was more frequent in older patients (p < 0.001) and in high-income countries.

**Conclusion:** Decisions of LST limitation are frequent in the ICU, and are associated with increased age and medical severity. However, a significant percentage of these patients survived. Interestingly, almost half of the patients who eventually died during their hospital stay had not been subject of a decision of LST limitation.

### F-96 Evaluation of the decision-making process leading to a decision not to readmit a patient to the intensive care unit during a same hospital stay

#### Guido Olivia^1^, Meunier-Beillard Nicolas^1^, Meziani Ferhat^2^, Delmas Emmanuel^1^, Dargent Auguste^1^, Rigaud Jean-Philippe^3^, Ecarnot Fiona^4^, Fournel Isabelle^1^, Quenot Jean-Pierre^1^

##### ^1^CHU de Dijon, France; ^2^CHRU de Strasbourg, France; ^3^Centre Hospitalier, Dieppe, France; ^4^Centre Hospitalier Universitaire, Besançon, France

###### **Correspondence:** Guido Olivia - olivia_guido@hotmail.fr

*Annals of Intensive Care* 2018, **8(Suppl 1):**F-96

**Introduction:** The risk–benefit ratio of (re-)admission to the intensive care unit (ICU) has been widely discussed in the literature. However, the ethics of non-readmission during a single hospital stay have not been widely addressed. A decision not to re-admit a patient to the ICU could be seen as a limitation of therapy, thus falling within the scope of the law dated 22 April 2005, by denying the patient access to potentially-available healthcare resources. In this context, we aimed to—(1) investigate whether decisions not to re-admit patients to the ICU are taken in accordance with French legislation + and (2) identify the characteristics of patients concerned by this type of decision.

**Patients and methods:** This study was based on data from the prospective, multicentre IVOIRE cohort (Influence of socio-economic vulnerability on initial severity and prognosis of patients admitted to the ICU + PHRC-IR 2012). We identified patients included in two large regional university hospitals in the East of France for whom a decision not to re-admit was taken during a single hospital stay. The decision-making process was evaluated based on a questionnaire comprising 13 items developed by a sociologist from semi-directive interviews with clinicians.

**Results:** Among 857 patients discharged from the ICU alive, a decision not to re-admit to the ICU during a same hospital stay was noted in the medical file of 63 patients (7.3%). This decision was primarily made on the day of discharge (42.6%), and those involved in the decision included—the family, an outside consultant, and the patient themselves in 54, 31.7 and 15.9% of cases respectively. The decision was justified in medical terms in 92.1% of cases, and the main reasons cited were—(1) therapeutic impasse (52.4%) + (2) comorbidities (44.4%) + (3) degree of dependence of the patient (34.9%). Patients concerned by decisions of this type were generally older (75 vs 65.6 years, p < 0.0001), with more comorbidities (median 4 vs 2, p = 0.001), greater loss of dependence according to Katz’s activities of daily living (5 vs 6, p < 0.0001), and longer duration of life-sustaining therapies (7.5 vs 5 days, p = 0.028).

**Conclusion:** Although the profile of the patients identified in this study likely justified the decision not to re-admit the patient to the ICU, there is room for improvement in the decision-making process.

### F-97 Evolution between 1993 and 2013 of 4 triage criteria for admission to a French ICU

#### Mentec Hervé^1^, Mouhtatine Youssef^1^

##### ^1^CH Victor Dupouy, Argenteuil, France

###### **Correspondence:** Mentec Hervé - herve.mentec@ch-argenteuil.fr

*Annals of Intensive Care* 2018, **8(Suppl 1):**F-97

**Introduction:** Nowadays, opposite to ancient times, the feeling of caring only for patients accumulating frailty factors wins ICU physicians. We assessed the evolution of the prevalence of 4 patients’ characteristics, deemed frailty markers, on ICU admission over 20 years.

**Patients and methods:** Retrospective study of reports of all patients admitted to the same ICU in 1993 and 2013. During that period, the medical team evolved only slowly + therefore the same medical philosophy prevailed. We recorded the prevalence of 3 characteristics on 2 levels—age > = 80 years with or without cognitive impairment, smoking with or without COPD, alcoholism with or without cirrhosis, and of a forth characteristic—metastatic cancer or malignant hemopathy (MCMH). We recorded SAPS II, vital supports, withholding and withdrawing of cares and ICU mortality.

**Results:** 589 patients were admitted in 1993, 597 in 2013. Median age was 59.8 years in 1993, 66.3 years in 2013 (p < 0.0001). The proportion of patients > = 80 years increased between 1993 and 2013 (10.2 to 17.3%, p = 0.0004). The prevalence of cognitive impairment also increased (0.4 to 4.0%, p < 0.0001). The prevalence of smoking decreased (36.2 to 32.3%, p = 0.001) and prevalences of COPD (15.6 to 19.3%, p = 0.63), alcoholism (26.0 to 29.5%, p = 0.80), cirrhosis (5.9 to 7.0%, p = 0.92) and MCMH (11.0 to 11.4%, p = 0.68) remained stable. Median number of the 4 characteristics per patient was similar in 1993 and 2013 (1[0–2] and 1[0–1], p = 0.86). Median SAPS II was higher in 2013 (45.0) compared to 1993 (32.1) (p = 0.0001). Compared to 1993, vital supports and withholding or withdrawing of cares were more frequent in 2013. ICU mortality was higher in 2013 (29%) than in 1993 (20%) (p = 0.001). ICU mortality increased with accumulation of the 4 characteristics. In multivariate analysis, factors positively associated with ICU mortality were age > = 80 years, alcoholism-cirrhosis combination, SAPS II, catecholamine infusion and mechanical ventilation, whereas admission for drug intoxication had a protective effect.

**Conclusion:** The intensivists’ feeling that patients admitted to ICU changed over time is confirmed. However, among the 4 characteristics we studied, only age > = 80 years and cognitive impairment were more prevalent in 2013 and only age > = 80 years and alcoholism-cirrhosis combination were independently associated with ICU mortality.

### F-98 Non-therapeutic ventilation for organ donation. National survey of practice

#### Si Larbi Anne-Gaëlle^1^, Soummer Alexis^1^, Lapergue Bertrand^1^, Gaudin Virginie^1^, Bonnin France^1^, Cerf Charles^1^

##### ^1^Hôpital Foch, Suresnes, France

###### **Correspondence:** Si Larbi Anne-Gaëlle - agsl17@club-internet.fr

*Annals of Intensive Care* 2018, **8(Suppl 1):**F-98

**Introduction:** Most of organ donors are brain dead patients. In some cases, patients are identified as potential donors before brain death and will undergo intubation and mechanical ventilation for the sole purpose of awaiting brain death. The aim of this study is to evaluate the practices of professionals in charge of potential donors.

**Patients and methods:** In 2014, a questionnaire was sent by e-mail to 1800 French health professionals working in departments in charge of potential donors (ICU, neurology, emergency room), 183 responded with a response rate of 10%. Qualitative variables are expressed in percent and compared by Chi2 test.

**Results:** 169 questionnaires are usable. 117 respondents (69%) are ICU physicians (ICUp), 36 (21%) are non-ICU physicians (non-ICUp) and 16 (10%) are non-medical caregivers. Among the non-ICUp, 19 (53%) are neurologists, 7 (19%) are emergency physicians and 8 (22%) work in another department. 47% work in a university hospital and 57% in Paris area. Experience of the physicians—112 ICUp (97%) have already pursued mechanical ventilation with the sole purpose of waiting for brain death, and 68% of all physicians have already intubated a patient for this sole purpose (Table 1). In this case, the issue of organ donation was addressed to the relatives before intubation by 42% of ICUp and 68% of non ICUp (p = 0.03). 29% of participants never addressed organ donation before the brain death. For the 71% who have done so at least once, organ harvesting never happened in 20% of cases. Legitimacy and difficulties (Table 1)—21% of respondents felt that when a decision of treatment withdrawal or withholding is taken, the patient should not go to ICU for any reason and 29% think that these patients should be allowed to die “quietly”. The prospect of an extubation if brain death does not occur or in case of organ donation refusal is a problem for 24% of ICUp and 38% of non ICUp (p = 0.08). 88% of ICUp and 93% of non ICUp think they would need to receive training.

**Table 1:** Questionnaire results

Practices and difficulties	ICU physicians	Non ICU physicians
*Already continued MV in the sole purpose of OD?*	112 (97%)	
If yes, time of information to relatives?		
When they seemed able to understand	60 (54%)	–
After decision of WLST	43 (38%)	
Only if relatives asked for WLST	29 (26%)	
*Already intubated in the sole purpose of OD?*	78 (70%)	22 (63%)
If no, why?		
Situation never occurred or do not think of it	19 (56%)	10 (77%)
This practice does not seem ethical	11 (32%)	0
If yes, time of information to relatives		
Before intubation	33 (42%)	15 (68%) *
At the time of brain death	16 (20%)	1 (5%)
*Addressing organ donation before death*		
May leave false hopes for relatives	48 (44%)	16 (53%)
Is a problem	30 (28%)	8 (27%)
Is difficult	49 (42%)	21 (58%)
If it is difficult, what kind of difficulties		
Relation with relatives	36 (73%)	17 (81%)
Ethical problem	20 (41%)	7 (33%)
Personal and emotional problem	10 (20%)	5 (24%)

*MV* mechanical ventilation, *OD* organ donation, *WLST* withdrawal of life sustaining treatments

*p < 0.05 with Chi2 test

**Conclusion:** This study shows that pursuing mechanical ventilation for the sole purpose of awaiting brain death and organ harvesting is a common practice, and that intubating a patient for this purpose alone is done in most of cases but could still be more generalized. On the other hand, information to the relatives should be improved.

### F-99 Effect of low tidal volume on absolute humidity delivered by heated humidifiers in ICU ventilators: a bench study

#### Guerin Claude^1^, Moro Barbara^1^, Baboi Loredana^1^, Yonis Hodane^1^, Lissonde Floriane^1^, Aublanc Mylène^1^, Louf-Durier Aurore^1^, Riad Zakaria^1^, Richard Jean-Christophe^1^

##### ^1^Hôpital de la Croix Rousse, Lyon, France

###### **Correspondence:** Guerin Claude - claude.guerin@chu-lyon.fr

*Annals of Intensive Care* 2018, **8(Suppl 1):**F-99

**Introduction:** In ARDS patients under ECMO common ventilator strategy aims at resting the lung by lowering tidal volume (VT). We were wondering whether such low VT can impact the efficacy of heated humidifiers (HH) in ICU ventilators to achieve the recommended target of 33 mg L^−1^ absolute humidity (AH).

**Patients and methods:** Pneumatic test lung with 20 ml cm H_2_O compliance and 20 cm H_2_O L s resistance was attached to any of 5 ICU ventilators (V 500 (Drager), Carescape R 860 (GE Healthcare), Servo U (Maquet), G5 (Nihon-Kohden) and PB980 (Medtronic)) equipped with Fisher-Paykel MR 850 HH and double wired limb ventilator circuit. PEEP was set to 12 cm H_2_O and FIO2 0.21. HH was set in manual mode and targeted 37 °C into the chamber and 40 °C at the Y piece. AH was assessed by using the psychrometric method and probes temperature continuously monitored. HH was run during 45 min at VT 500 ml then 100 ml, at respiratory rate (RR) 30 breaths min. Once probes temperatures leveled off at VT 100 ml, 10 cycles were counted then 120 ml VT was delivered til next plateau temperature and so on by 20 ml-increment in VT up to 280 ml. Then VT was lowered to 100 ml and RR to 15 breaths min. At the time of plateau temperature same VT increment as above was performed. Experiment was performed by using adult (RT 380 EVAQUA Fisher Paykel) and repeated with neonate (RT 266 EVAQUA Fisher-Paykel) ventilator circuit. The relationship of VT to AH were analyzed by using linear model for each circuit-RR combination.

**Results:** With adult RR15, adult RR30, neonate RR15 and neonate RR30 the median intercept and slope of the relation ship of VT to AH were 28 and 0.017 °C ml, 31 and 0.017, 25 and 0.023, 28 and 0.019, respectively. There were significant différences across ICU ventilators from this mean effect (Fig. 1).

**Fig. 1:**
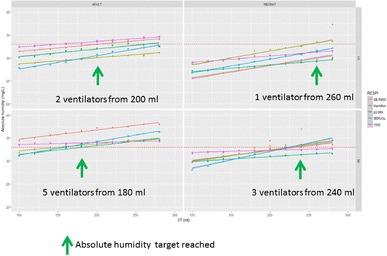
Differences across ICU ventilators

**Conclusion:** AH increased with increasing VT with differences across ventilators and circuit RR combinations. Recommended 33 mg L^−1^ AH was never reached in some ventilatorsBest setting is adult circuit and RR 30 min for reaching 33 mg L^−1^ AH.

### F-100 Continuous prolonged monitoring with EIT in supine and prone position: feasibility and first results

#### Mezidi Mehdi^1^, Riad Zakaria^1^, Yonis Hodane^1^, Louf-Durier Aurore^1^, Tapponnier Romain^1^, Lissonde Floriane^1^, Aublanc Mylène^1^, Baboi Loredana^1^, Richard Jean-Christophe^1^, Guérin Claude^1^

##### ^1^Hospices Civils de Lyon, France

###### **Correspondence:** Mezidi Mehdi - mezidi.mehdi@gmail.com

*Annals of Intensive Care* 2018, **8(Suppl 1):**F-100

**Introduction:** Electric impedance tomography (EIT) is a non-invasive tool allowing a continuous monitoring of regional lung ventilation. Prone position (PP) for long sessions in association with low tidal volume and cisatracurium infusion has been shown survival benefit in moderate to severe ARDS patients. The goal of the present study was to report on the feasibility of continuous EIT monitoring and to describe time course of EIT-derived indexes during a 16-hour PP session.

**Patients and methods:** This is a secondary study of patients included in a prospective interventional physiologic study performed in patients with moderate to severe ARDS (PaO2 FIO2 < 150 mmHg), intubated, sedated and paralyzed, requiring PP. The study was evaluating 2 different PEEP strategies. Notably, PEEP and other settings was kept constant during the last 14 h of the 16 h PP session. EIT was performed with Swisstom BB2 device. Patients were assessed in SP at 30° and in PP at 0°or 15° from the horizontal plane. EIT belt was placed around the chest according to manufacturer’s specifications. To prevent pressure sores in the PP dressing was placed between EIT belt and mattress and around the EIT wire. We analyzed the the center of ventilation (CoV), which describes the well-ventilated areas of the lung with 0% for ventilation occurring only in the ventral region and 100% for a ventilation only in the dorsal region. We report the time course of CoV in supine position (SP) 30 min before PP, 30 min after the beginning of PP (H0.5) and every hour (H1, H2… H16) till PP end. Results are expressed in median [1st-3rd quartiles] and count (percentage).

**Results:** Continuous EIT was recorded in 19 patients of who 15 patients (79%) had usable records and 10 (67%) enough time points for present purpose. One patient presented an erythema on the sternum despite the protective dressing. In the SP, CoV was greater than 50% in every patient indicating that the ventilation took place predominantly in the dorsal (spine) lung regions. With PP, CoV did not vary significantly (p = 0.53) between SP and H0.5. However, some patients experienced clinically significant drop or rise of CoV (Fig. 1). CoV reached a plateau after 5 [3–6] h.

**Fig. 1:**
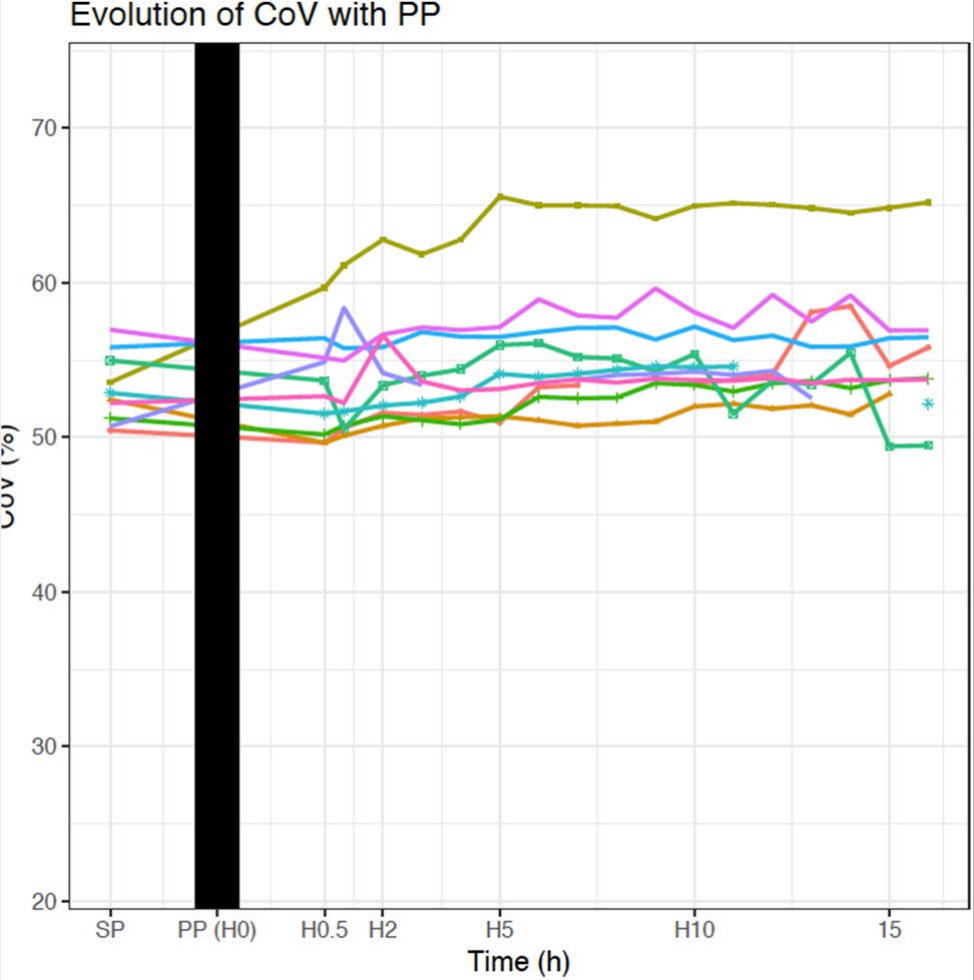
Evolution of CoV with PP

**Conclusion:** Continuous and prolonged EIT monitoring is feasible and safe in PP. Main issue is to keep a stable signal. The ventilation redistribution may take a long time for some patients.

### F-101 How long should the patient be left in prone position?

#### Jochmans Sebastien^1^, Mazerand Sandie^1^, Sy Oumar^1^, Chelly Jonathan^1^, Vong Ly Van^1^, Ellrodt Olivier^1^, Mercier Des Rochettes Emmanuelle^1^, Abdallah Razach^1^, Rolin Nathalie^1^, Serbource-Goguel Jean^1^, Vinsonneau Christophe^2^, Monchi Mehran^1^

##### ^1^GH Sud Ile-de-France, Hôpital de Melun, Melun, France; ^2^CH Bethune, Melun, France

###### **Correspondence:** Jochmans Sebastien - sebastien.jochmans@ch-melun.fr

*Annals of Intensive Care* 2018, **8(Suppl 1):**F-101

**Introduction:** The optimal duration of a prone position session (PP) is not yet known.

**Patients and methods:** All ARDS patients treated with PP were included. We recorded before the session and every 30 min (during the session) up to 24 h—dynamic compliance (Cdyn), PetCO2, dead space (Vd Vt) and phase 3 slope of volumetric capnography. We evaluated for each session whether there was a positive response for each parameter—decrease in PetCO2, Phase 3 slope, Vd Vt and increase in Cdyn.

**Results:** 57 patients and 114 sessions were analysed. PaO2 FiO2 was 124 [87–163] with PEEP 16 [14–18], RR 25 [20–30] and VT 6.5 mL kg IBW [6.1–7.3]. The effect of PP on the monitored parameters varies significantly between each patient but also between each session for the same patient. In positive responders, the effect continues statistically for 7 to 20 h depending on the parameter studied—10.5 h for Vd Vt, 19.5 for Phase 3 slope, 7 for PetCO2 and 9 for Cdyn.
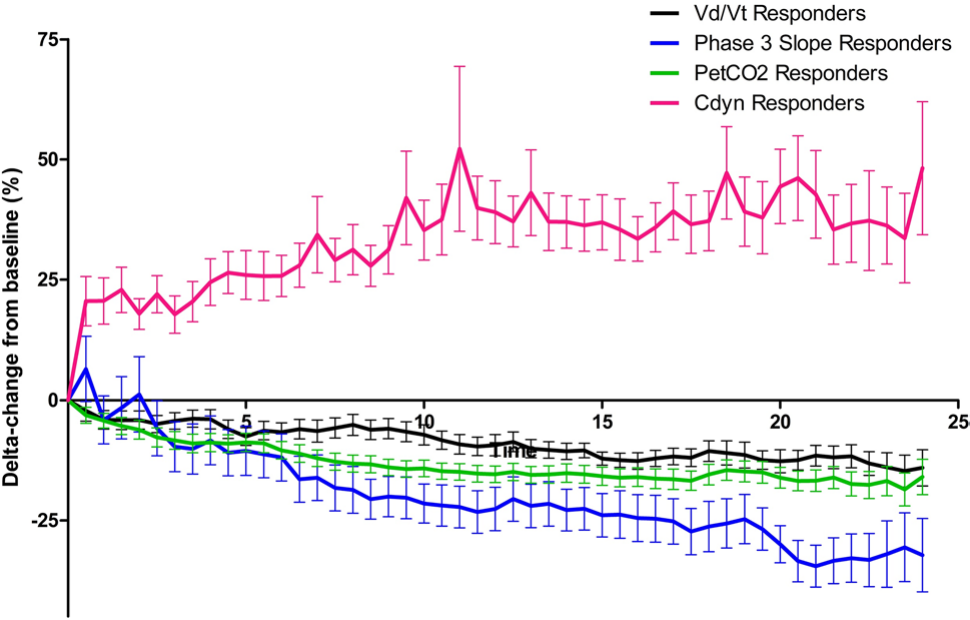


**Conclusion:** The maximum effect of prone positioning on selected parameters seems to be obtained after 20 h of therapy.

### F-102 Respiratory mechanic, transpulmonary pressure and oxygenation during PEEP titration with the Express protocol in ARDS patients

#### Bergez Marie^1^, Tranvan David^1^, Saghi Tahar^2^, Gentile Ariane^1^, Ouattara Alexandre^3^, Labadie Philippe^4^, Fontaine Bruno^1^, Roze Hadrien^4^

##### ^1^Hopital des Armées Robert Piquet, Bordeaux, France; ^2^Polyclinique Bordeaux nord, Bordeaux, France; ^3^SAR2, CHU de Bordeaux, Bordeaux, France; ^4^CHU de Bordeaux, France

###### **Correspondence:** Bergez Marie - bergez.marie@gmail.com

*Annals of Intensive Care* 2018, **8(Suppl 1):**F-102

**Introduction:** Some protocols use individual respiratory mechanics to titrate PEEP in ARDS (1, 2). Respiratory mechanics with transpulmonary pressure and oxygenation during individual PEEP titration using the Express protocol in ARDS patients is unknown. This study measured and compared transpulmonary pressures with the Express protocol.

**Patients and methods:** Prospective data collection. Oesophageal pressure (Pes) was measured with Nutrivent™ catheter. Patients under myorelaxant received randomly 2 methods of PEEP titration, alternatively based on plateau pressure (Express group) or PEEP 5–8 cm H_2_O (Mini distension group). We used the following equations– Relative inspiratory transpulmonary pressure (PL rel) = Plateau pressure x (lung elastance respiratory system elastance). Transpulmonary expiratory Pressure (Ptp expi) = End expiratory airway pressure—End expiratory oesophageal pressure − 5 cm H_2_O (2)—Driving Pressure (DP) = plateau pressure—total PEEP—Transpulmonary Driving Pressure (DPtp) = DP—deltaPoeso—Respiratory system elastance (ERS) = DP VT.—Lung elastance (EL) = DPtp VT—PEEP volume = expired VT from PEEP to ZEEP-VT.

**Results:** 17 patients were included in 2016. With the Express protocol, PEEP increased from 7[5–8] to 15[12–16] cm H_2_O p < 0.0001 + PaO2 FiO2 increased from 87[70–121] to 94[84–185], p = 0.02, and DP remained unchanged 13[10–15] vs 13[11–16] cm H_2_O. With Express protocol Plateau pressure, relative transpulmonary inspiratory pressure (PLrel) and expiratory transpulmonary pressures were 28 [28–30], 20 [18–23.5] and 6 [4–10] cm H_2_O, respectively. Transpulmonary driving pressure (DPtp) was 13 [11–16.5] cm H_2_O and was highly correlated to DP (R2 = 0.97, p < 0.001), whereas lung (EL) and chest wall elastance (ECW) were 25[20–34] and 9.4[7.0–11.0] cm H_2_O litre, respectively. Patients with PLrel below 20cmH20 had higher ECW and lower DPtp. PEEP volume was significantly lower in the group with PLrel below 20cmH20 with 360[272–571] vs 621[438–856] ml, respectively, p = 0.04. PEEP corresponding Ptp expi matched FiO2 according to the Talmor protocol (2) for 3 patients.

**Conclusion:** Those results indicate the effect of the Express protocol on different index derived from oesophageal pressure at the bedside in ARDS patients. It shows that individual PEEP level were different from those proposed by the Talmor protocol. Further protocols based on inspiratory relative transpulmonary pressure in patients with higher chest wall elastance are required. 1. JAMA 2008 + 299–646. 2. N Engl J Med 2008 + 359–2095–2104.

### F-103 Neutrophil extracellular traps in the lungs of patients with pneumonia and the acute respiratory distress syndrome

#### De Prost Nicolas^1^, Bendib Inès^1^, De Chaisemartin Luc^1^, Granger Vanessa^1^, Schlemmer Frédéric^1^, Maitre Bernard^1^, Carteaux Guillaume^1^, Razazi Keyvan^1^, Mekontso Dessap Armand^1^, Martin Sylvie^1^

##### ^1^Hôpital Henri Mondor Créteil, Créteil, France

###### **Correspondence:** De Prost Nicolas - nicolas.de-prost@aphp.fr

*Annals of Intensive Care* 2018, **8(Suppl 1):**F-103

**Introduction:** The acute respiratory distress syndrome (ARDS) is characterized by lung infiltration with activated neutrophils. Neutrophil extracellular traps (NETs) are antimicrobial structures released by neutrophils. NETs have also been associated with tissue damage in experimental models of acute lung injury. Whether NETs are involved in the pathogenesis of human ARDS and could be a potential therapeutic target is unknown. We aimed to quantify alveolar NETs production in patients with pneumonia and ARDS and assess its relationship with outcomes.

**Patients and methods:** Prospective monocentric study. Patients admitted in the ICU in 2014 2015 with pneumonia and moderate severe ARDS were included. Immunosuppressed patients were excluded. NETs (DNA-myeloperoxidase) levels were measured by ELISA in broncho-alveolar lavage (BAL) fluid and serum samples of ARDS patients and in those of control patients (n = 4). Patients with higher and lower BAL fluid NETs levels were compared using the median as a cutoff value.

**Results:** Thirty-five patients with bacterial (n = 18), viral (n = 11) or non-microbiologically documented (n = 6) pneumonia and ARDS were included. NETs levels were significantly higher in BAL fluid than in blood of ARDS but not of control patients (Fig. 1). Patients with higher BAL NETs levels did not differ from others regarding baseline characteristics (age—55 ± 17 vs 52 ± 17 years, p = 0.63 + SAPS II—42 ± 18 vs 39 ± 14, p = 0.66 + SOFA—8 ± 4 vs 8 ± 3, p = 0.99). PaO2 FiO2 ratios did not differ upon admission (130 [69–186] vs 99 [81–160] mmHg, p = 0.65) but were higher in patients with higher BAL NETs levels on the day BAL was performed (242 [149–350] vs 142 [103–193] mmHg, p = 0.006) together with a trend towards higher blood neutrophil counts (13.6 ± 7.4 vs 9.3 ± 6.6 × 103 mm3, p = 0.09). Patients with higher BAL NETs levels did not have a significantly different hospital mortality (17.6 vs 16.7%, p > 0.99) but exhibited a shorter mechanical ventilation duration in survivors (6 [3–13] vs 11 [6–24] days, p = 0.026) and a trend towards more ventilator-free days at day 28 (22 [14–25] vs 14 [0–21] days, p = 0.066).

**Fig. 1:**
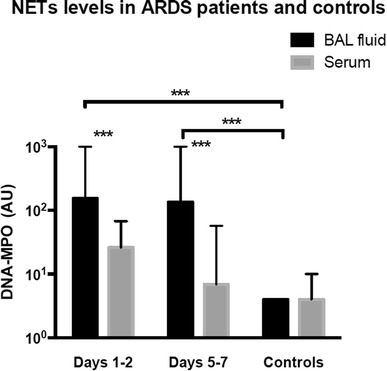
NETs levels in ARDS patients and controls

**Conclusion:** In this cohort of patients with pneumonia and ARDS, we observed a lung production of NETs-MPO. Patients with a higher lung production of NETs showed shorter MV duration than others. Our results do not support targeting NETs for adjuvant therapy in ARDS patients with pneumonia.

### F-104 PaO2 FiO2 ratio estimation from SpO2 FiO2 rati: a planned companion study of the SPECTRUM study

#### Michel Philippe^1^, Boissier Florence^2^, Aissaoui Nadia^3^, Barbar Saber Davide^4^, Brouard Florence^5^, Grimaldi David^6^, Hraiech Sami^7^, Lacherade Jean-Claude^8^, Lascarrou Jean-Baptiste^9^, Muller Grégoire^10^, Piton Gaël^11^, Youssoufa Atik Cerc (on Behalf Of The Srlf Trial Group)^7,12^

##### ^1^CH de Pontoise, France; ^2^CHU Poitiers, Poitiers, France; ^3^Hôpital Européen George Popidou, France; ^4^CHU de Nîmes, France; ^5^CH de Périgueux, France; ^6^Hôpital Erasme, Bruxelles, Belgique; ^7^Hôpital Nord, Marseille, France; ^8^CHD Vendée, La Roche-Sur-Yon, France; ^9^CHU de Nantes, France; ^10^Hôpital de la Source, Orléans, France; ^11^CHU de Besançon, France; ^12^Srlf Trial Group, France

###### **Correspondence:** Michel Philippe - philippemich@gmail.com

*Annals of Intensive Care* 2018, **8(Suppl 1):**F-104

**Introduction:** The ratio of arterial oxygen partial pressure to fractional inspired oxygen (PaO2 FiO2 or P F) is daily used to assess patients’ evolution under ventilatory support. Some studies reported the reliability of percutaneous oxygen saturation (SpO2) to appreciate PaO2 easy to get on bedside. Thus two equations have been proposed—Rice equation and Ellis equation. However, no large prospective study assessed the reliability of such equations to estimate the P F at the bedside in real conditions. Using the SPECTRUM (Severe hyPoxemia—prevalEnCe, TReatment and oUtcoMe) study, we aimed to evaluate the reliability of SpO2 obtained by Rice and Ellis equation.

**Patients and methods:** This study is a planned companion of SPECTRUM study, a recent prevalence-point-day conducted by the SRLF Trial group in 117 french-speaking ICU aiming to report the patterns and outcomes of hypoxemic patients (defined by P F < 300 mmHg). We included in the analysis all patients under mechanical ventilation with SpO2 < 98% (according to limit of the Rice study). SpO2 and FiO2 were measured simultaneously to arterial blood gas were drawn.

**Results:** Among 1656 Patients of the SPECTRUM study, 421 were on mechanical ventilation and had undergone arterial blood gas with simultaneously recorded SpO2 and FiO2. Of note, P F was < 100 mmHg for 132 + between 100 and 200 for 191 + and between 200 and 300 for 29. Pairwise correlations of truth P F with estimated P F was good (Rice—Spearman’s rho = 0.84, p < 0.001–Ellis—rho = .85 p < 0.001). Bland–Altmann test showed an important variability of results (P F vs Rice (Figure)—0.02 ± 57.8–P F vs Ellis—14.3 ± 45.5). The variability decreased with lower P F. Caution may be used to interpret our results because we did not reported the quality of SpO2 signal at the bedside.

**Conclusion:** Regarding the variability of the results, whatever the used equation, caution may be used to predict the P F by the SpO2 FiO2 ratio in patients under mechanical ventilation.

### F-105 Transpulmonary pressures measurements as a mean to optimize mechanical ventilation in a unique series of patients combining ARDS and very severe morbid obesity

#### Vimpere Damien^1^, Aissaoui Nadia^2^, Couteau-Chardon Amélie^3^, Lancelot Aymeric^3^, Commereuc Morganne^3^, Guerot Emmanuel^3^, Diehl Jean-Luc^4^

##### ^1^Hopital Georges Pompidou, Paris, France; ^2^Centre de Recherche Cardiovasculaire, Paris, France; ^3^Hôpital Européen Georges Pompidou, AP-HP, Paris, France; ^4^Université Paris Descartes, Paris, France

###### **Correspondence:** Vimpere Damien - damien.vimpere@aphp.fr

*Annals of Intensive Care* 2018, **8(Suppl 1):**F-105

**Introduction:** Morbid obesity and ARDS both affect respiratory mechanics mainly through their respective impacts on chest wall and lung elastances. We present a unique series of patients combining very severe morbid obesity and moderate to severe Acute Respiratory Distress Syndrome (ARDS). We describe the use of trans-pulmonary pressures (TPP) measurements for optimization of external PEEP setting.

**Patients and methods:** The monocentric observational study was performed in 8 morbidly obese patients admitted for moderate to severe ARDS. We performed an incremental PEEP trial (5 cm H2O steps) with TPP measurement (NutriVent probe, Sidam, Italy) in a semi-recumbent position as previously described. A decremental PEEP trial after a recruitment maneuver was not performed since the safety of such a maneuver in this specific population is largely unknown. We defined two ways for determination of external PEEP setting—(1) PEEP necessary to obtain a positive expiratory TPP and (2) PEEP necessary to obtain a plateau pressure between 28 and 30 cm H_2_O (maximal alveolar recruitment Express strategy). Data are expressed as numbers (%) and medians (interquartile range). Statistical analysis was made using the XLSTAT software.

**Results:** We enrolled during 5 years 8 morbidly obese patients (BMI 63 (IR 59–69)) admitted for a moderate to severe ARDS. Clinical characteristics are displayed in Table 1. The Express strategy indicated a PEEP setting of 15 cm H_2_O (IR 15–20) whereas TPP-guided PEEP was 30 cm H_2_O (IR 25–32), p = 0.03. Driving pressure was higher in the Express strategy PEEP setting (10.7 cm H20 (IR 9–12)) than in the TPP-guided PEEP (10.2 cm H20 (IR 9.5–13)), p = 0.92. TPP-guided PEEP setting was higher than indicated by the Express strategy in all but one patient. One patient suffered from transient hypotension when external PEEP was set at 25 cm H_2_O, while no patient displayed an inspiratory TPP higher than 25 cm H_2_O. Additional data will be provided during the meeting—pressure–volume curve at ZEEP (6 patients), CRF measurements (4 patients) and ABG and capnometry values at each PEEP level (3 patients).

**Table:**
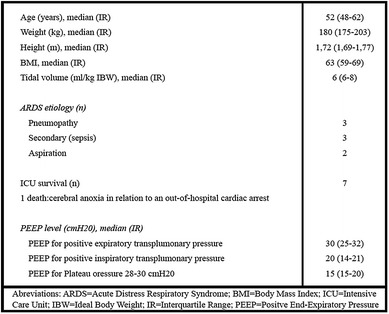

**Conclusion:** In our ARDS patients with extremely severe obesity, an incremental PEEP trial with TPP measurements appeared to be safe and indicated a PEEP setting significantly higher than for the commonly-used ARDS strategies. Such an approach deserves further comparisons with other modalities of monitoring, such as CRF measurements, EIT studies, etc.

### F-106 Severe poisoning by cardiotoxic drugs and circulatory assistance: 5-year experience at French university hospital

#### Tardif Elsa^1^, Conil Jean-Marie^1^, Georges Bernard^1^, Marcheix Bertrand^1^, Crognier Laure^1^, Bounes Fanny^1^, Delmas Clement^1^

##### ^1^CHU Rangueil, Toulouse, France

###### **Correspondence:** Tardif Elsa - tardif.elsa@gmail.com

*Annals of Intensive Care* 2018, **8(Suppl 1):**F-106

**Introduction:** Toxicity from cardiac drugs is associated with a large number of fatalities, significant morbidity and healthcare consequences. Severity of these poisonings can be explained by a refractory cardiogenic shock not responding to optimal conventional treatment. Criteria of circulatory assistance indications remain unclear. The aim of the study was to describe and to compare patients intoxicated by cardiotoxic drug treated with or without veno-arterial ExtraCorporeal Membrane Oxygenation (VA ECMO).

**Patients and methods:** Retrospective cohort study conducted at French university hospital. All patients intoxicated with cardiotoxic drugs between January 2012 and March 2017 were included. Patients were divided into 2 groups—with and without VA ECMO.

**Results:** Among the 105 patients included in the study, 18 patients were treated with VA ECMO (17%) and 87 patients with conventional therapies. ECMO was respectively employed for refractory shock and cardiac arrest in 13 and 5 cases, all patient required vasopressor support. In-hospital mortality was 10.5% and was significantly higher in the ECMO group (44.4%). Beta-blockers with membrane stabilizing activity and non-dihydropyridine calcium channel blockers poisoning were the most commonly reported in the ECMO group. Mean time from hospital admission to initiation of ECMO was 11 h [1–53] and the average ECMO duration was 2.6 days [1–8]. No serious adverse reaction was reported during this period.

**Table:** 

	Conventional treatment n = 87	ECMO V-A n = 18	*p* value
*Clinical data at admission*			
Glasgow Coma Scale, score	15 [14 to 15]	14 [6 to 15]	0.13
Temperature, degree	36.3 [36 to 36.5]	36.1 [35.2 to 36.8]	0.54
Mean arterial pressure, mmHg	70 [67 to 74]	51 [45 to 63.6]	*0.006*
Heart rate, beats/min	70 [63 to 78]	70 [59 to 91]	0.93
PaO2/FiO2 ratio	392,9 [361 to 433]	238,8 [93.8 to 429]	*0.02*
*Paraclinical exams at admission*			
Minimal Left Ventricular Ejection Fraction, %	55 [50 to 55]	20 [10 to 48]	*< 0.0001*
Sinus rhythm, n (%)	77 (90.6)	10 (55.6)	*0.001*
QRS, ms	80 [80 to 80]	120 [98 to 200]	*0.02*
Corrected QT interval, ms	441 [425 to 458]	495 [436 to 555]	*0.03*
PR, ms	180 [180 to 200]	120 [110 to 200]	0.06
*Biological data at admission*			
pH	7.385 [7.38 to 7.41]	7.36 [7.24 to 7.43]	0.14
Lactate levels, mmol/l	2.05 [1.4 to 2.54]	4.3 [1.8 to 7.99]	*0.006*
Bicarbonate, mmol/l	22.65 [21.3 to 23.5]	20.15 [16 to 23.2]	0.19
Base excess, mEq/l	− 2.8 [(− 3.6) to (− 1.4)]	− 6 [(− 9.3) to (− 0.6)]	0.19
pCO2, mmHg	36.75 [35 to 39]	40.1 [35.5 to 50]	0.09
Troponin, ng/l	7 [7 to 7.1]	7 [3.5 to 25.3]	0.77
Blood glucose level, g/l	1.2 [1.1 to 1.3]	1.5 [1.16 to 2.4]	*0.05*
Serum sodium levels, mmol/l	140 [139 to 141]	142.5 [139.4 to 146]	*0.02*
Serum potassium levels, mmol/l	3.9 [3.8 to 4]	3.35 [2.9 to 4.1]	*0.01*
Creatinine, µmol/l	78.5 [70.4 to 87]	89.5 [63.8 to 151.6]	0.45
Prothrombin time, %	91 [88 to 94]	80 [64 to 88.8]	*0*.*01*
Factor V, %	109 [71 to 125]	58.5 [38.7 to 72.8]	*0*.*007*
SGOT, UI/l	23 [21 to 25]	30.5 [21 to 65.2]	*0*.*05*
SGPT, UI/l	23 [18 to 25]	28 [17 to 71]	0.07
*Biological data at day 1*			
pH	7.4 [7.38 to 7.4]	7.4 [7.38 to 7.48]	0.39
Lactate levels, mmol/l	1.3 [1 to 1.47]	2.1 [1.6 to 4]	*0.02*
Bicarbonate, mmol/l	23.2 [22 to 24]	19.7 [17.1 to 21.8]	*0.006*
Base excess, mEq/l	− 1.7 [(− 1.9) to (− 0.8)]	− 3.9 [(− 8.2) to (− 0.4)]	*0.03*
pCO2, mmHg	37.4 [35 to 40]	29.3 [28 to 33.7]	*0.0001*
Serum sodium level, mmol/l	139 [139 to 141]	145 [140 to 150]	*0.0007*
SGOT, UI/l	30 [22.3 to 45]	93 [49.6 to 1063]	*0.0005*
SGPT, UI/l	26 [19 to 35.6]	77 [36 to 722.9]	*0.003*
Prothrombin time, %	74.5 [68 to 79.5]	58 [43.6 to 68.7]	*0.001*
Factor V, %	78 [58.4 to 95.3]	46 [19.4 to 59.3]	*0.002*
*Treatment in intensive care unit*			
Norepinephrine, n (%)	31 (35.6)	16 (89)	*<* *0.0001*
Dobutamine, n (%)	10 (12)	6 (33)	*0.03*
Epinephrine, n (%)	13 (15)	14 (78)	*<* *0.0001*
Isoprenaline, n (%)	14 (16)	7 (38.9)	0.05
Crystalloids/24 h, ml	2000 [1500 to 2198]	3605 [2500 to 5000]	*0.0001*
Renal replacement therapy, n (%)	2 (2.3)	7 (38.9)	*<* *0.0001*
*Death, n (%)*	3 (3.4)	8 (44.4)	*<* *0.0001*

Results expressed in median value ± confidence interval

**Conclusion:** Refractory cardiogenic shock following cardiotoxic drug poisoning requiring circulatory assistance is associated with significant mortality. Even if its use seems justified by the literature, the implantation criteria must be specified and this after an optimal conventional treatment to prevent multiple organ failure.

### F-107 Outcome of poisoned patients with cardiovascular failure requiring massive catecholamine doses: back from irreversibility?

#### Megarbane Bruno^1^, Bouchet Amandine^1^, Peron Nicolas^1^, Nuzzo Alexandre^1^, Kerdjana Lamia^1^, Malissin Isabelle^1^, Voicu Sebastian^1^, Deye Nicolas^1^

##### ^1^Hôpital Lariboisière, Paris, France

###### **Correspondence:** Megarbane Bruno - bruno.megarbane@lrb.aphp.fr

*Annals of Intensive Care* 2018, **8(Suppl 1):**F-107

**Introduction:** Maximal dosage thresholds for catecholamine infusion were associated with almost 100%-fatality rate in patients presenting cardiovascular failure of non-toxic causes and mainly in case of sepsis. In contrast, little is known regarding such thresholds in poisoning while survivors were reported despite receiving massive catecholamine doses. Our objectives were to test the validity in poisoning of the thresholds determined in non-poisoned patients and evaluate the survival rate, the catecholamine-attributed complications and the predictive factors of death in the poisoned patients receiving massive catecholamine doses.

**Patients and methods:** We performed a retrospective study including all poisoned patients admitted to our intensive care unit (ICU) during a 13-year period (2003–2016) and who received massive catecholamine doses (i.e. epinephrine > 5 mg h + norepinephrine > 10 mg h and or isoprenaline > 5 mg h) to treat cardiovascular failure. The usual demographic, clinical and outcome data were collected. Univariate analysis was performed with Mann–Whitney and Chi-2 tests.

**Results:** Two hundred forty-six poisoned patients (representing 25% of the poisoned patients admitted in the ICU and receiving catecholamine infusion during this period) were included in the study. The patients (56% females 44% males, age—47 years [36 + 57] (median [percentiles 25 + 75]) were poisoned in relation to self—(88%) or accidental overdose (12%). One-hundred and seventeen patients (48%) survived. The involved toxicants were calcium-channel inhibitors (), beta-blockers (), sodium-channel blocker (), vasodilatators (). The mortality rate was significantly correlated with the epinephrine (p = 0.0001) and norepinephrine (p < 0.0002) infusion rate. The maximal infusion rates associated with survival were 50 mg h epinephrine, 140 mg h norepinephrine and 25 mg h isoprenaline. Catecholamine-attributed complications (11%) were mesenteric () and limb ischemia (). Based on univariate analysis, the other predictive factors of death were the onset of cardiovascular arrest (p < 0.0001), the female sex (p = 0.03), decreased Glasgow coma score (p = 0.0001), the cardiogenic shock (p = 0.004), ECMO requirement (p = 0.003), mechanical ventilation (p = 0.01), lactate elevation (p < 0.0001), plasma creatinine (p = 0.009), transaminases (p < 0.0001) and CPK (p < 0.0001) as well as prothrombine time (p < 0.0001), platelets (p < 0.0001), arterial pH (p < 0.0001) and PaO2 FiO2 ratio (p = 0.008).

**Conclusion:** Surviving to drug-related cardiovascular failure requiring major catecholamine doses overwhelming the usual thresholds associated with irreversibility (i.e. fatality rates of 100%) in non-toxic situations.

### F-108 Assessment of inferior vena cava size as a static parameter to predict fluid responsiveness in ventilated patients with circulatory failure

#### Repessé Xavier^1^, Evrard Bruno^2^, Charron Cyril^2^, Gonzalez Céline^2^, Jacob Christophe^2^, Bouferrache Koceila^2^, Maizel Julien^2^, Prat Gwenaël^2^, Goudelin Marine^2^, Daix Thomas^2^, François Bruno^2^, SlamaMichel^2^, Vieillard-Baron Antoine^2^, Géri Guillaume^2^, Vignon Philippe^2^

##### ^1^CHU Ambroise Paré, Boulogne-Billancourt, France; ^2^HU Dupuytren, Limoges, France

###### **Correspondence:** Repessé Xavier - xavier.repesse@aphp.fr

*Annals of Intensive Care* 2018, **8(Suppl 1):**F-108

**Introduction:** As a static parameter, end-expiratory IVC diameter (IVCEE) has been disregarded to accurately predict fluid responsiveness (FR) in small study samples. Nevertheless, IVCEE is easy and rapid to measure in most critically ill patients by the frontline intensivist with competence in basic critical care echocardiography (CCE).

**Patients and methods:** Ancillary study of a multicenter cohort of ventilated ICU patients who were prospectively assessed using CCE for an acute circulatory failure of any origin in 5 ICUs. In each patient, the following parameters were measured—IVCEE in the subcostal longitudinal view of the vessel, left ventricular (LV) stroke volume using the Doppler method before and after a 90°-PLR, and bladder pressure as a surrogate of intra-abdominal pressure (IAP). FR was defined by an increase of LV stroke volume > 10% after 1 min of PLR, when compared to baseline.

**Results:** Among the 540 patients of the initial cohort, IVCEE was not available in 117 patients (22%) due to a poor subcostal view, and finally 423 patients were included in the present study (median age—65 years [56–76] + 281 men + median SOFA—10 [8–12]), 172 of them exhibiting FR (58%). Acute circulatory failure was related to sepsis in 237 patients (56%) and ICU mortality reached 41%. To predict FR, the optimal IVCEE threshold values were lower than 8 (n = 16 patients), 10 (n = 31) and 13 mm (n = 62) to reach a 95, 90 and 85% specificity, respectively. Conversely, optimal IVCEE threshold values were higher than 28 (n = 17), 27(n = 28) and 25 mm (n = 61) to reach a 95, 90 and 80% specificity in predicting the absence of FR. In multivariable analysis, IVCEE was independently associated with FR and interacted significantly with IAP. A strong and adjusted relationship between IVCEE and FR in the subgroup of patients with an IAP < 12 cm H_2_O was observed while this relationship was significantly less pronounced in patients with IAP > = 12 cm H_2_O (Fig. 1).

**Fig. 1:**
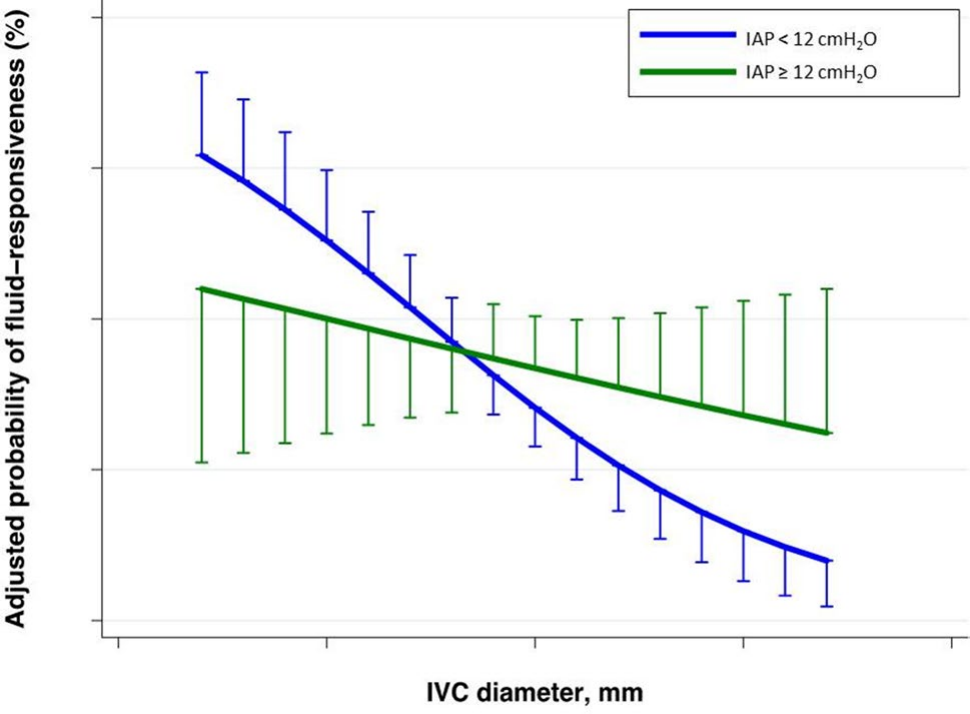
IVC size as a static parameter to predict fluid responsiveness in ventilated patients with circulatory failure

**Conclusion:** Measurement of IVCEE is moderately feasible in ventilated patients with acute circulatory failure of various origins and appears as a specific indicator of FR in the presence of markedly low or high values, although many patients are in a large “grey” zone. This static index should be used with caution in patients with elevated IAP.

### F-109 Vasopressor cumulative dose requirement and risk of early death during septic shock: an analysis from the EPISS cohort

#### Dargent Auguste^1^, Nguyen Maxime^1^, Fournel Isabelle^1^, Bourredjem Abderrahmane^1^, Charles Pierre-Emmanuel^1^, Quenot Jean-Pierre^1^

##### ^1^CHU Dijon, Dijon, France

###### **Correspondence:** Dargent Auguste - auguste.dargent@chu-dijon.fr

*Annals of Intensive Care* 2018, **8(Suppl 1):**F-109

**Introduction:** Septic shock is the primary cause of death in intensive care units, with about 20% of patients dying in the first 3 days in a state of multi-organ failure. To design future trials focused on early mortality, we require knowledge of early indicators that can detect patients at high risk of early death from refractory septic shock. It was recently demonstrated that vasopressor dependency and refractory shock could be predicted by a MAP-phenylephrine dose–response curve. The aim of this study was to assess whether the cumulative dose of vasopressors (CDV) is a predictor of early death (within 72 h) attributable to septic shock (EDASS).

**Patients and methods:** This substudy of the EPISS trial was based on 370 patients admitted to a French ICU for septic shock between 2009 and 2011. CDV was calculated as the cumulative dose of epinephrine + norepinephrine. EDASS was defined as defined as fulfillment of the following criteria—death within the first 72 h following vasopressor initiation, ongoing vasopressor therapy at time of death, exclusion of a cause of death other than sepsis. The area under the receiving operating characteristic (ROC) curve was calculated for the CDV at 6, 12, 24, 36, and 48 h after vasopressor initiation. A strategy in two steps was designed in order to assess the feasibility of real-time, early detection of EDASS risk with CDV, based on selected times and thresholds.

**Results:** Among the 370 patients included, 51 (14%) died within the first 72 h with 40 (11%) EDASS. The CDV of patients in the EDASS group was significantly higher (p < 0.0001) at all-time points after the introduction of catecholamines than among those without EDASS, as early as 6 h from catecholamine initiation (Fig. 1). A strategy in two steps (CDV > 800 µg kg at 6 h and or CDV > 2600 µg kg at 24 h) was able to predict EDASS with sensitivity of 45%, specificity 97%, positive predictive value 78% and negative predictive value 94%. Overall, this two-step strategy identified 18 high-risk patients at 6 h, of whom 15 presented EDASS.

**Fig. 1:**
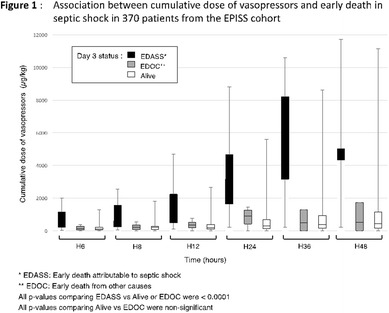
Association between cumulative dose of vasopressors and early death in septic shock

**Conclusion:** Overall, our results confirm that early death directly attributable to septic shock could be effectively predicted by the CDV in the first hours of treatment. These results will help to select patients eligible for innovative therapies aimed at improving early mortality in septic shock.

### F-110 CO2 interpretation during cardiopulmonary resuscitation: a bench and clinical study

#### Savary Dominique^1^, Drouet Adrien^2^, Grieco Domenico Luca^3^, Rigollot Marceau^4^, Badat Bilal^4^, Delisle Stephane^4^, Charbonney Emmanuel^5^, Ouellet Paul^5^, Ricard Cecile^2^, Mancebo Jordi^5^, Mercat Alain^6^, Brochard^7^, Laurent, Richard Jean-Christophe^2^

##### ^1^CH Annecy Genevois, Metz-Tessy, France; ^2^CHU Metz-Tessy, France; ^3^Université du Sacré coeur, Rome, Italy; ^4^Air Liquide, Paris, France; ^5^Université de Montréal, Canada; ^6^CHU d’Angers, France; ^7^Hôpital Saint Michael, Toronto, Canada

###### **Correspondence:** Savary Dominique - dsavary@ch-annecygenevois.fr

*Annals of Intensive Care* 2018, **8(Suppl 1):**F-110

**Introduction:** In patients with cardiac arrest, end-tidal CO2 (EtCO2) has been proposed to monitor the efficacy of cardiopulmonary resuscitation (CPR) but uncertainty persists on its interpretation. We hypothesized that exhaled CO2 may also by affected by occurrence of “lung airways” collapse previously noticed during CPR. Because this closure may possibly also limit oxygenation + analysis of the entire exhaled CO2 time waveform—may give information of high clinical value to manage CPR. We report preliminary results from a clinical and bench study aimed at describing the pattern of the capnogram during CPR.

**Patients and methods:** Clinical study: Capnograms were recorded prospectively during CPR in patients with out-of-hospital cardiac arrest and enrolled in a French registry and later analysed. EtCO2 was recorded at each breath and we calculated a coefficient of transmission (CTCO2) describing the CO2 swings induced by CPR: ratio of the change in exhaled CO2 value induced by compression to the EtCO2 value.

Bench study: A constant CO2 flow, simulating CO2 production, was added in the aveoli in a bench model including a simulation of airway closure. Same methodology was applied in a Thiel cadaver model (ethic committee approval CER-14–201–08–03.17) from the Anatomy Department of a University of Canada. We obtained CO2/time waveforms on the lung model and in cadavers similar to patients’ recordings under different ventilator settings.

**Results:** The capnograms from 54 patients were analysed age 71[57–79] years, female 13 (24%), survivors after hospital admission 7 (13%). Qualitative assessment exhibited mainly 3 patterns that correlated with CTCO2. CTCO2 was independent from EtCO2 (Fig. 1). In bench and cadaver models, PEEP increase resulted in significant CTCO2 increase.

**Fig. 1:**
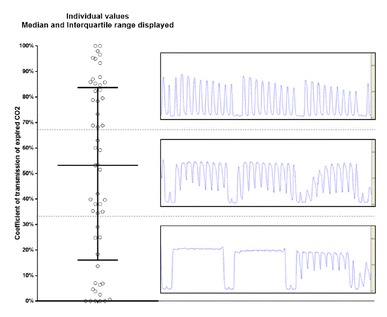
CO2 interpretation during cardiopulmonary resuscitation

**Discussion:** These observations suggest that CO2 pattern during CPR depends not only on circulation but also on the status of airways opening. Examination of the CO2 pattern and measuring CTCO2 could permit to adjust ventilator settings during CPR without impairing circulation.

**Conclusion:** During CPR, the capnogram provides information reflecting airway patency, and unrelated to EtCO2. This can contribute to assess tidal ventilation generated by chest compressions and diffusion of oxygen to the alveoli.

### F-111 Extracorporeal life support for refractory out-of-hospital cardiac arrest: outcome analysis according to a modified institutional protocol

#### Lavigne Flavie^1^, Pozzi Matteo^2^, Armoiry Xavier^3^, Jacquet Lagreze Matthias^3^, Schweizer Remi^3^, Portran Philippe^3^, Fellahi Jean-Luc^3^

##### ^1^Bron, France; ^2^Hôpital Louis Pradel, Bron, France; ^3^University of Warwick, ROYAUME UNI

###### **Correspondence:** Lavigne Flavie - flavielavigne@gmail.com

*Annals of Intensive Care* 2018, **8(Suppl 1):**F-111

**Introduction:** Refractory out-of-hospital cardiac arrest (OHCA) is a catastrophic condition with a dismal survival under conventional cardiopulmonary resuscitation (CPR). In this setting extracorporeal life support (ECLS) could be suggested as a rescue therapeutic option. However, the results of ECLS for refractory OHCA are disappointing. Moreover, the underlying cardiac rhythm is a matter of debate as previous reports found shockable rhythms as independent predictors of survival. The aim of this report was to analyse the outcomes of ECLS for refractory OHCA in our single-center experience according to the modification of our institutional protocol.

**Patients and methods:** Patients who received an ECLS for witnessed, refractory, OHCA from January 2010 to December 2016 were included in this analysis. The criteria for ECLS implantation were—(1) no-flow time ≤ 5 min + (2) low-flow time ≤ 75 min (≤ 100 min from January 2010 to July 2012) + (3) end-tidal carbon dioxide (ETCO2) ≥ 10 mmHg. Patients were divided into 2 groups according to the study period and cardiac rhythm at ECLS implantation—(1) Period 1 = from January 2010 to December 2014, shockable and non-shockable rhythms + (2) Period 2 = from January 2015 to December 2016, only shockable rhythms. We excluded from our analysis patients—(1) referred for ECLS support for refractory cardiogenic shock + (2) experiencing severe hypothermia (body temperature < 32 °C) before CPR + (3) with pre-existing, known major comorbidities compromising short-term (< 1 year) life expectancy + (4) aged < 18 years.

**Results:** Over the 7-year study period, we used ECLS in 81 patients with refractory OHCA. Baseline characteristics, cardiac arrest etiology, no-flow and low-flow duration were comparable between the 2 groups (Table 1). The survival to hospital discharge was not statistically different between the 2 groups (Period 1 = 8.8% [n = 6 patients] vs. Period 2 = 7.7% [n = 1 patient], p = 0.69). In the Period 1 group, all the survivors to hospital discharge (n = 6 patients) presented a shockable rhythm at ECLS implantation.

**Table 1:** Baseline characteristics and outcome

	Period 1 N = 68	Period 2 N = 13	*p*
Age, years	44 (40)	40 (11)	0.26
Male sex, n (%)	50 (73)	12 (92)	0.13
No-flow time, min	3 (4)	2 (3)	0.70
Low-flow time, min	83 (22)	80 (22)	0.70
EtCO2, mmHg	20 (11)	29 (19)	0.05
NSH-R, n (%)	50 (73)	1 (8)	< 0.001
SH-R, n (%)	18 (26)	12 (92)	< 0.001
Cardiac arrest etiology, n (%)	27 (40)	5 (38)	0.60
Acute coronary syndrome	5 (7)	0 (0)	
Aortic dissection	4 (6)	0 (0)	
Pulmonary embolism	4 (6)	0 (0)	
Pre-existing cardiomyopathy	9 (13)	1 (8)	
Various	19 (28)	5 (38)	
Unknown			
Renal replacement therapy, n %)	16 (24)	5 (38)	0.22
Survival to discharge, n (%)	6 (9)	1 (8)	0.69
ECLS duration, days	2 (3)	2 (2)	0.70
ICU stay, days	7 (23)	7 (17)	0.98
Total hospital, days	10 (32)	10 (21)	0.95

Data are expressed as counts (percentages) or mean (standard deviation)

EtCO2, end tidal of carbon dioxide; NSH-R, non shockable rhythm; SH-R, shockable rhythm; ECLS, extracorporeal life support; ICU, intensive care unit

**Conclusion:** The introduction of a restrictive protocol based on the underlying cardiac rhythm in the setting of refractory OHCA has dramatically reduced the number of ECLS implantation. Nonetheless, the survival to hospital discharge is still poor even for refractory OHCA with a shockable rhythm. Further investigations are needed in order to best define those patients who could benefit from this potential life-saving procedure.

### F-112 Decreased prevalence, but persisting hazard of cardiogenic shock at the acute stage of myocardial infarction: a 20-year perspective

#### Aissaoui Nadia^1^, Puymirat Etienne^2^, Delmas Clément^3^, Bonello Laurent^4^, Henry Patrick^5^, Bonnefoy Eric^6^, Schiele François^7^, Ferrieres Jean^3^, Simon Tabassome^8^, Danchin Nicolas^2^

##### ^1^Hôpital Européen Georges Pompidou, Paris, France; ^2^Université Paris-Descartes, Paris, France; ^3^CHU Rangueil, Toulouse, France; ^4^Hôpital Nord, Marseille, France; ^5^Hôpital Lariboisière, Paris, France; ^6^Hôpital cardiologique Louis Pradel, Lyon, France; ^7^CHU Jean Minjoz, Besancon, France; ^8^Hôpital Saint Antoine, Paris, France

###### **Correspondence:** Aissaoui Nadia - nadia.aissaoui@aphp.fr

*Annals of Intensive Care* 2018, **8(Suppl 1):**F-112

**Introduction:** Cardiogenic shock (CS) is one of the most severe complications of acute myocardial infarction (AMI). Little information is available on trends in prevalence, management and outcomes in recent years.

**Patients and methods:** We used data from the FAST-MI programme, consisting of 5 one-month nationwide French surveys of patients admitted for AMI in intensive care units of cardiology, carried out 5 years apart since 1995, to assess the characteristics of CS. Data on timing of CS were recorded since 2000.

**Results:** From 1995 to 2015, prevalence of CS consistently decreased from 6.9% in 1995 to 2.8% in 2015. Prevalence decreased both for CS on admission (from 2.7% in 2000 to 1.1% in 2015) and CS developing at a later stage (from 4.4 to 1.7%). In CS patients, baseline demographic characteristics did not change markedly over time (mean age 74 years in 1995, 72 years in 2015 + female sex 44% in 1995, 28% in 2015, p = 0.11). Diabetes mellitus remained unchanged while hypertension increased from 52 to 66% and hypercholesterolemia from 24 to 42%. History of AMI (28 to 15%), stroke (13 to 4%) and heart failure (20 to 6%) decreased over time. In contrast, cardiac arrest increased from 9% in 2005 to 16% in 2015. In parallel, invasive ventilation increased from 16 to 40% and circulatory assist devices from 0.4 to 9%. Use of PCI increased from 20% in 1995 to 74% in 2015, while Coronary Artery Bypass Graft Surgery during the initial hospital stay increased from 2 to 4.8%. Six-month mortality was 73% in 1995, decreased to 62% in 2005 and remained as high as 58% in 2015. At each time point, mortality was lower in patients who underwent PCI + trends in 6-month mortality showed little difference from 1995 to 2015 in patients without PCI (76 to 74%), and only a slight decrease in those undergoing PCI (60 to 53%). Using Cox multivariate analysis both the period (2015 vs 1995 HR 0.65, 95% CI 0.47–0.91) and in-hospital PCI (HR 0.66, 95% CI 0.53–0.81) were independent predictors of death.

**Conclusion:** Prevalence of CS in AMI patients has notably decreased over the past 20 years. Mortality of CS patients in the most recent period remains high, and the gain in mortality over time is correlated with a greater use of PCI.

### F-113 ECLS induces changes in immune cells that may promote nosocomial infection

#### Frerou Aurelien^1^, Lesouhaitier Mathieu^1^, Gregoire Murielle^1^, Uhel Fabrice^1^, Gacouin Arnaud^1^, Maamar Adel^1^, Le Tulzo Yves^1^, Flecher Erwan^1^, Tarte Karin^1^, Tadie Jean-Marc^1^

##### ^1^CHU de Rennes, France

###### **Correspondence:** Frerou Aurelien - aurelien.frerou@gmail.com

*Annals of Intensive Care* 2018, **8(Suppl 1):**F-113

**Introduction:** Although major advances in management of Extra Corporeal Life Support (ECLS) patients have been made in the last decade, the short-term mortality remains important. The high incidence of nosocomial infections during ECLS largely contributes to this poor outcome. Cardiopulmonary bypass (CBP) during cardiac surgery induces a systemic inflammatory response associated with an immune dysregulation and a significant pulmonary dysfunction which has been well characterized. Surprisingly, there are only a few data available on immunological changes induced by ECLS. We believe that ECLS leads to immune dysfunction that could expose patients to nosocomial infections.

**Patients and methods:** A two-phase study was lead. First we analyzed blood cell count with differential (including lymphocyte, neutrophils and monocyte counts) in all patients who received ECLS in our institution from 2014 to 2017 within the first week following ECLS initiation. Secondly, Monocytes, granulocytes, dendritic cells and lymphocytes function were assessed at day 0, day 1 and day 3 using flow cytometry and functional tests in patients receiving ECLS and compared to patients with cardiogenic shock without ECLS.

**Results:** Among 159 patients with ELCS we found an early and persistent lymphopenia and a late neutrophilia (found to be associated with poor outcome in critically ill patients). Compared to control (n = 7), we found in patients who received ECLS (n = 6) a significant increase in immature granulocytes (104.107 ± 47.106 on day one versus 95.105 ± 96 ± 105, p = 0.01) and lymphocytes apoptosis. ECLS induced changes in Myeloid derived suppressors cells proportion (3.69% ± 3.5 on day three versus 0.58% ± 0.49 before ECLS, p = 0.05), which has been recently associated with a higher incidence of nosocomial infections and seems to be major actors of sepsis-induced immune suppression. Complement component 5a receptor (C5aR) from the neutrophil cell surface, was also decreased after ECLS initiation (ratio of mean fluorescence index 0.73 ± 0.22 on day one, p = 0.03) which is a sign of complement-induced neutrophil dysfunction in septic patients.

**Conclusion:** ECLS induces quantitative and qualitative leukocytes dysfunctions that can lead to a greater susceptibility to nosocomial infections which contribute to the poor outcome observed in several studies.

### F-114 Interest of microbiological documentation in aspiration pneumonia in a cardiac arrest population admitted in an intensive care unit—a retrospective monocentric study

#### Diehl Jean-Luc^1^, Vimpere Damien^1^, Guerot Emmanuel^1^, Couteau-Chardon Amélie^1^, Lancelot Aymeric^1^, Commereuc Morganne^1^, Aissaoui Nadia^1^

##### ^1^Hôpital Européen Georges Pompidou, Paris, France

###### **Correspondence:** Diehl Jean-Luc - jean-luc.diehl@aphp.fr

*Annals of Intensive Care* 2018, **8(Suppl 1):**F-114

**Introduction:** Aspiration pneumonia is a common complication of cardiac arrest. Although its real incidence remains undetermined, probabilist antibiotherapy is frequently or even systematically prescribed in these cases. We assessed the incidence of out-of-hospital cardiac arrest-related aspiration pneumonia and the impact of a microbiological documentation in regard to antibiotherapy course.

**Patients and methods:** All patients admitted for out-of-hospital cardiac arrest from 1 1 2014 to 1 8 2017 were studied. In our ICU, aspiration pneumonia is suspected when a clinical syndrome (fever, per resuscitation constatation) and or chest radiography infiltrates were present. In case of suspected aspiration pneumonia, a microbiological documentation was performed before initiation of probabilist treatment with amoxicillin-clavulanate. We retrospectively defined if patients have aspiration pneumonia using the following criteria—per resuscitation constatation, chest radiography infiltrates, fever. The number of microbiological documentation leading to an antibiotherapy modification was recorded as well as pathogens types. Data are expressed as numbers (%) and medians (interquartile range). Statistical analysis was made as appropriate using the XLSTAT software.

**Results:** 176 patients were studied. Clinical characteristics are displayed in Table 1. 80 (45) received a probabilist antibiotherapy and 79 (45) were retrospectively considered with aspiration pneumonitia. Results of microbiological documentation were 94 (53) positive microbiological sample and 68 (38) with a positive threshold whose 6 (3) were considered colonized (i.e. no clinico-radiological sign). On the entire positive culture sample, 51 (54) were positive with oropharyngeal flora as unique pathogen, 14 (15) with Streptococcus pneumonia, 11 (12) with Haemophilus influenza, 10 (11) with Staphylococcus aureus (no meticilin-resistant) and 3 (3) with Pseudomonas aeruginosa. No anaerobic pathogens were isolated. In 18 cases (20) the documentation lead to a modification of the probabilist antibiotherapy—10 downgrade, 4 upgrade, 3 arrest and 1 introduction. Pathogens associated with modifications are oropharyngeal flora (arrest), Staphyloccus aureus (downgrade) and Pseudomonas aeruginosa (upgrade). Haemophilus influenzae is equally associated with downgrade and upgrade.

**Table:**
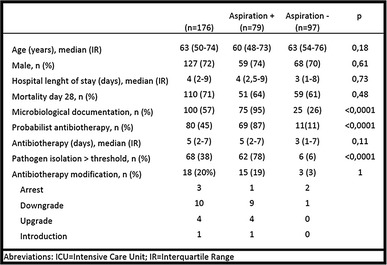

**Conclusion:** Aspiration pneumonia occurs in less than half of patients with out-of-hospital cardiac arrest. Microbiological documentation leads to antibiotherapy modification in almost a quarter of treated patients. These results don’t support systematic undocumented antibiotics prescription.

### F-115 Association between augmented renal clearance, pharmacological dosing and clinical outcomes in patients receiving piperacillin-tazobactam administered by continuous infusion

#### Petit Laurent^1^, Carrié Cédric^1^, Sauvage Noemie^1^, D’Houdain Nicolas^1^, Cottenceau Vincent^1^, Lafitte Mélanie^1^, Legeron Rachel^1^, Breilh Dominique^1^, Sztark François^1^

##### ^1^CHU BORDEAUX, Bordeaux, France

###### **Correspondence:** Petit Laurent - laurent.petit@chu-bordeaux.fr

*Annals of Intensive Care* 2018, **8(Suppl 1):**F-115

**Introduction:** This study aimed to assess whether augmented renal clearance (ARC) impacts negatively on piperacillin-tazobactam pharmacokinetic pharmacodynamics (PK PD) target attainment in critically ill patients receiving 16 g day by continuous infusion.

**Patients and methods:** Over an 8-month period, all critically ill patients treated by piperacillin-tazobactam for a suspected or documented sepsis without renal impairment were eligible. During the first three days of antimicrobial therapy, every patient underwent 24-hour creatinine clearance (CrCL) measurements and therapeutic drug monitoring at steady state. The main PK PD outcome investigated in this study was the rate of empirical target non-attainment using a theoretical target MIC of 16 mg L^−1^ for piperacillin and 2 mg L^−1^ for tazobactam. The secondary clinical outcome was the rate of therapeutic failure in microbiologically documented infections, defined as an impaired clinical response with a need for escalating antibiotics during treatment and or within 15 days after end-of-treatment.

**Results:** Over the study period, 60 patients were included in the primary pharmacological analysis and 45 in the secondary clinical analysis. Using a MIC of 16 mg L^−1^ for piperacillin, the rate of empirical target non-attainment in the overall population was 17%, with a strong association with CrCL (Fig. 1). Mean CrCL values ≥ 200 ml min over the three first days of antimicrobial therapy were statistically associated with empirical target non-attainment (OR 9.2 [2.2–38.5], p = 0.001). In microbiologically documented infections, mean CrCL values ≥ 200 ml min over the three first days of antimicrobial therapy were statistically associated with under-exposure < 4.MIC (OR 9.0 [2.0–40.6], p = 0.003). Under-exposure < 4.MIC was significantly associated with therapeutic failure (OR 10.7 [2.1–53.7], p = 0.003).

**Fig. 1:**
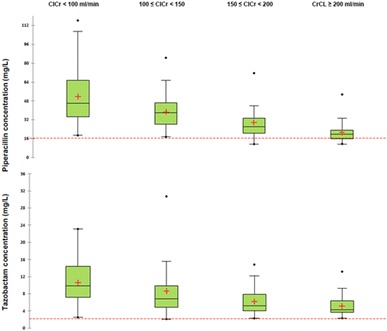
24-hour creatinine clearance (CrCL) measurements and therapeutic drug monitoring

**Conclusion:** Empirical administration of piperacillin-tazobactam 16 g day by continuous infusion should be appropriate for most critically ill patients without renal impairment, although patients with very high CrCL ≥ 200 ml min remain at risk of empirical or documented target non-attainment. These data emphasize the need of therapeutic drug monitoring in patients with ARC, especially when targeting less susceptible pathogens or surgical infections with limited penetration of antimicrobial agents.

### F-116 Targeting antifungal prophylaxis in liver transplantation: results of a retrospective cohort study

#### Terzi Nicolas^1^, Truche Anne-Sophie^1^, Ruping Florian^1^, Le Gall Fanny^1^, Bonadona Agnes^1^, Hamidfar Rebecca^1^, Minet Clémence^1^, Ara Somohano Claire^1^, Moustapha Ibrahim^1^, Schwebel Carole^1^

##### ^1^CHU Grenoble Alpes, La Tronche, France

###### **Correspondence:** Abid Sonia - NTerzi@chu-grenoble.fr

*Annals of Intensive Care* 2018, **8(Suppl 1):**F-116

**Introduction:** Invasive fungal infections are a major burden in solid organ transplantation, especially in patients receiving liver graft. However, their incidence has decreased thanks to the development of an antifungal prophylaxis in the post-transplantation period. In patients at high risk of invasive fungal infection (IFI), this strategy is recommended, whereas its benefit remains controversial in low-risk patients. However, there is no clear definition of these two patients groups. Our aim was to provide recent data on epidemiology, mortality and IFI risk factors in the early post-operative course in a population without any antifungal prophylaxis.

**Patients and methods:** This was a retrospective observational single center analysis of all consecutive patients receiving a liver transplantation between January 2010 and August 2016. The primary outcome was the occurrence of an IFI in the 90-day following transplantation. Qualitative variables were described as frequency (percentage) and quantitative variables as mean (standard deviation) when normality hypothesis was satisfied otherwise as median [interquartile range]. A multivariate logistic regression was conducted in order to identify IFI risk factors.

**Results:** 232 consecutive patients were included, with a median MELD score of 12 [5–21] and SAPS II score of 45 ± 14. Median numbers of transfusion during surgery was 5 units [1–9] and cold ischemia time was 399 min [339–472]. Biliary complications were the most frequent and occurred in 68 patients (29.3%). Seventeen patients (7.3%) developed a proven IFI during the considered period (Fig. 1). Candida spp. was the only pathogen identified. There was no significant difference in mortality between the two groups (1 17 with IFI vs. 2 215 without IFI, p = 0.13), and no death could be imputed to IFI occurrence. Multivariate analysis showed that biliary complications was the only factor associated with a significant higher IFI risk (OR 8.27, 95% CI [2.8–28.1], p = 0.003). The only factor associated with such a complication was transplantation in super urgent conditions.

**Conclusion:** IFI occur during post-operative course of liver transplantation in low risk patients not receiving antifungal prophylaxis. In a targeted preventive strategy, patients developing biliary complications appear eligible candidates for early post-operative antifungal prophylaxis.

### F-117 Early surgery and functional outcomes of critically ill patients with Staphylococcus aureus infective endocarditis

#### Abid Sonia^1^, Papin Grégory^1^, Mourvillier Bruno^1^, Lebut Jordane^1^, Smonig Roland^1^, Magalahes Eric^1^, Andremont Olivier^1^, Dupuis Claire^1^, Juguet William^1^, Neuville Mathilde^1^, Mariotte Eric^2^, Bouadma Lila^1^, Timsit Jean-François^1^, Sonneville Romain^1^

##### ^1^Hôpital Bichat, Paris, France; ^2^Hôpital Saint Louis, Paris, France

###### **Correspondence:** Abid Sonia - s.sonia.abid@gmail.com

*Annals of Intensive Care* 2018, **8(Suppl 1):**F-117

**Introduction:** Staphylococcus aureus is the most common cause of infective endocarditis (IE) and is associated with a severe prognosis. Patients frequently require ICU admission because of acute circulatory failure or neurological complications, or the need for emergent valvular surgery. The benefit of early surgery on functional outcomes remains uncertain. We aimed to identify risk factors for poor functional outcomes in critically ill patients with Staphylococcus aureus IE.

**Patients and methods:** We conducted a retrospective analysis of consecutive patients admitted to the medical ICU with acute left-sided defined Staphylococcus aureus IE according to the DUKE criteria, from January 2007 to December 2016. Factors associated with a poor functional outcome, defined by a score > 3 on the modified Rankin scale (i.e., severe disability or death) at 90 days, were identified by multivariate analysis. The model was built using clinically relevant variables associated with outcome by univariate logistic regression analysis (p < 0.2). Organ failures were defined by a score > 2 on each component of the SOFA score. Data are presented as median (interquartile) or numbers (percentage).

**Results:** A total of 110 consecutive patients were studied (age—62 [52–70] years, SOFA score—9 [5–13], GCS—15 [13–15]), of whom 78 (71%) patients underwent early valve replacement surgery, 2 [1–5] days after admission. Mechanical ventilation, renal replacement therapy, catecholamines were needed in 56 (51%), 22 (20%), 59 (65%) respectively. At 90 days, 73 (66%) patients had a poor functional outcome (i.e. 60 (55%) deaths and 13 (12%) severe disabilities). The only independent factor positively associated with a poor outcome was renal failure at ICU admission, (Odds ratio (OR) = 5.52, 95% confidence interval (95% CI), 1.56–19.56). By contrast, early surgery, performed within 48 h after admission, had a protective effect (OR 0.08, 95% CI 0.02–0.34) (Table 1). Septic shock, neurological complications and IE characteristics were not independently associated with functional outcome.
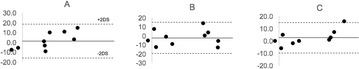

**Table:**
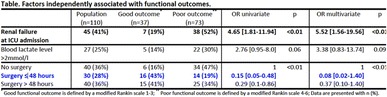

**Conclusion:** Our study suggests that about one-third of patients admitted to the ICU for Staphylococcus aureus IE have good functional outcomes at 90 days. The main factor at admission associated with poor neurological outcome is renal failure. Our study confirms the benefit of early valve replacement surgery on functional outcomes, even in the most severe patients.

### F-118 Retrospective study of the neurotoxicity induced by piperacillin and amoxicillin administration in critically ill patients

#### Grangé Steven^1^, Lagoutte Jennifer^1^, Grall Maximillien^1^, Lebouar Gurvan^1^, Lagache Laurie^1^, Lemaitre Caroline^1^, Surlemont Elisabeth^1^, Carpentier Dorothée^1^, Béduneau Gaétan^1^, Girault Christophe^1^, Misset Benoit^1^, LamoureuxFabien^1^, Tamion Fabienne^1^

##### ^1^CHU de Rouen, France

###### **Correspondence:** Grangé Steven - steven.grange@chu-rouen.fr

*Annals of Intensive Care* 2018, **8(Suppl 1):**F-118

**Introduction:** Central nervous system toxicity following administration of beta-lactam antibiotics is a potential cause of morbidity and mortality. Ensuring a reliable and safe drug dosing in critically ill patients can be difficult, due to variable and dynamic organ function. Indeed, overexposure to piperacillin and amoxicillin can lead to neurological disorders. Pharmacological therapeutic monitoring (PTM) of beta-lactams ensures the efficacy and safety of the treatment by measuring the residual serum concentration. There are currently no clear guidelines on a threshold for toxic concentrations for all beta-lactams and therapeutic drug monitoring in critically ill patients may be a useful intervention to avoid antibiotic-related toxicities. The aim of this study was to determine the therapeutic concentration values of the beta-lactam antibiotics as well as threshold values associated with toxicity.

**Patients and methods:** This study was carried out retrospectively during the period 01.01.2015 to 08.02.2017, in patients hospitalized in the medical ICU-CHU Rouen with PTM. The dosages of piperacillin and amoxicillin were performed in the Department of Pharmcology and Toxicology using a high performance liquid chromatography technique coupled with tandem mass spectrometry (HPLC MS).

**Results:** The number of beta-lactam antibiotics was 119. Of these 119 requests, half were for piperacillin (49.6%), and one-third were for amoxicillin (30.3%). The other dosages were mainly for cloxacillin, cefepime, cefotaxime and ceftriaxone. The results confirmed that serum concentrations of piperacillin (21.6 ± 4.0 vs 98.2 ± 16.8 mg L^−1^ < 0.0001) and amoxicillin (35.8 ± 13.0 vs 157 ± 72 mg L^−1^ < 0.0001) significantly were higher in patients with neurological disorders or wakefulness delays. The ROC curves allowed the predictive values associated with the presence of neurological disorders attributable to antibiotic treatment, corresponding to residual serum concentrations of piperacillin of 66 mg L^−1^ and amoxicillin of 160 mg L^−1^. A predictive value for neurological disorders of these concentrations is proposed for residual serum concentrations greater than 40 mg L^−1^ for both antibiotics (80% specificity and sensitivity).

**Conclusion:** Our results suggest that there is an association between a residual concentration of piperacillin and amoxicillin greater than 40 mg L^−1^ and the occurrence of neurological disorders. Pharmacological therapeutic monitoring of beta-lactams in critically ill patients may be a useful intervention to optimize the antibiotic regimens and to avoid antibiotic-related toxicities.

### F-119 Prognosis of severe infectious endocarditis, impact of preoperative neurological complications and evaluation of surgical tolerance: retrospective multicenter study

#### Gros Alexandre^1^, Séguy Benjamin^2^, Toussaint Aurélia^3^, Préau Sébastien^2^, Durocher Alain^3^, Coupez Elisabeth^4^, Souweine Bertrand^1^, Marest Delphine^5^, Reignier Jean^5^, Robin Guillaume^6^, Porte Lydie^6^, Lavie-BadieYoan^6^, Galinier Michel^6^, Coudroy Remi^7^, Robert René^7^, Urien Jean-Marie^8^, Martins Raphaël^8^, Leclercq Christophe^8^, Boyer Alexandre^2^

##### ^1^CH de la Côte Basque, Bayonne, France; ^2^CHU de Bordeaux, France; ^3^CHU de Lille, France; ^4^CHU Clermont-Ferrand, France; ^5^CHU de Nantes, France; ^6^CHU de Toulouse, France; ^7^CHU de Poitiers, France; ^8^CHU de Rennes, France

###### **Correspondence:** Gros Alexandre - alexandre.gros37@gmail.com

*Annals of Intensive Care* 2018, **8(Suppl 1):**F-119

**Introduction:** Neurological complications during severe infectious endocarditis (IE) interfere with surgeon’s decisions and IE prognosis by the risk of haemorrhagic transformation or increase in cerebral haemorrhage during extracorporeal circulation. The objective is to determine the factors associated with the IE 1 year survival.

**Patients and methods:** Observational, multicenter, retrospective cohort study performed in 6 French University Hospitals from 2010 to 2016. Severe (SOFA score > = 3) left-sided endocarditis defined by modified Duke criteria in patients admitted in cardiac or medical ICU, complicated by at least one neurological event (MRI or CT-scan diagnosis) were included. Factors associated with the risk of 1 year mortality were assessed by uni and multivariate analysis.

**Results:** Among the 152 patients, 124 experienced strokes, 57 cerebral haemorrhages, 28 mycotic aneurysms, 22 meningitis. 76 (50%) underwent valvular surgery (13 emergency, 43 urgent, 19 elective, and 1 unknown), with a mortality rate of 39% compared with 74% without surgery (OR 0.23–95CI 0.12–0.46–p < 0.0001). Factors associated with 1 year mortality in univariate analysis were—male gender (OR 2.43–95CI 1.24–4.76—p 0.01), chronic renal insufficiency (OR 3.96–95CI 1.85–8.49—p 0.0003), immunosuppression (OR 4.44–95CI 1.33–14.85–p 0.02), higher Euroscore 2 and SOFA (respectively p 0.02 and p 0.003), lower systolic ejection fraction (p 0.0003) and antibiotics duration (p < 0.0001). Protective factors were oral streptococcal infection (OR 0.17–95CI 0.05–0.57—p 0.0047), mycotic aneurysms (OR 0.42–95CI 0.19–0.97—p 0.04), valvular surgery (OR 0.23–95CI 0.12–0.46—p < 0.0001) and β-lactams-aminoglycosides combination (OR 0.48–95CI 0.24–0.93—p 0.03). In multivariate analysis, 1 year mortality independent factors were chronic renal insufficiency (OR 3.6–95CI 1.1–12.1–p 0.03) and antibiotics duration (p 0.002). In subgroup analysis 87% (41 47) of patients undergoing surgery did not present neurological worsening.

**Conclusion:** Chronic renal insufficiency and antibiotics duration are the only factors associated with 1 year prognosis of severe IE with neurological complications. Surgery, despite its low complications rate, did not impact their prognosis. A future propensity-matched cohort study will be needed to reduce confusion bias due to influence of neurological complications on surgeon’s decisions.

### F-120 Impact of resistant ICU-acquired Gram negative bacilli bloodstream infection on patient prognosis -Analysis of a large French ICU network

#### Bailly Sebastien^1^, Murris Juliette^2^, Lucet Jean-Christophe^3^, Lepape Alain^4^, L’Hériteau François^5^, Aupee Martine^6^, Bervas Caroline^7^, Boussat Sandrine^8^, Berger-Carbonne Anne^9^, Machut Anaïs^4^, Savey Anne^4^, Timist Jean-François^3^

##### ^1^CHU, Grenoble, France; ^2^INSERM IAME, Paris, France; ^3^CHU BICHAT, Paris, France; ^4^HCL Lyon, Lyon, France; ^5^AP HP, Paris, France; ^6^CHU Rennes, Rennes, France; ^7^CHU Bordeaux, Bordeaux, France; ^8^CHU Nancy, Nancy, France; ^9^Santé Publique, Paris, France

###### **Correspondence:** Bailly Sebastien - sebbailly@wanadoo.fr

*Annals of Intensive Care* 2018, **8(Suppl 1):**F-120

**Introduction:** Gram negative bacilli (GNB) bloodstream infection (BSI) is the main source of ICU-acquired bacteremia. The incidence of multidrug resistant GNB (R-GNB) isolates has increased significantly in France from 2005 to 2015. The impact of R strain on the prognosis of GNB-BSI patients has not been assessed.

**Patients and methods:** Data from a large French national ICU network were explored during a 5-year period (2011–2015). Patients with a GNB-BSI were included and were divided into two groups according to the resistance (R) profile (BSI due to a R isolate or not). The following resistances were considered—all GNB-BSI including Pseudomonas spp., Acinetobacter spp., Stenotrophomonas spp. and Enterobacteriacae (Eb) for which the following antimicrobial resistances were considered—ticarcillin and ceftazidime (cefta) (Pseudomonas (PA)), third generation cephalosporin (3GC) (Eb) and imipenem (all GNB). After variable selection using random forest and univariable mixed logistic regression models, a multivariable analyses using a mixed model with a random effect (center). Sub-group analyses were performed according to species (PA and Eb) and resistance for Eb.

**Results:** From 189,020 patients admitted in an annual median of 198 French ICUs, 6837 experienced an ICU-acquired (> 48 h.) BSI, 3462 (51%) BSI due to GNB, including 1345 (41%) BSI due to R isolates. PA was identified in 828 (24%) (MDR-PA BSIs 248 (30%)) and Eb in 2933 (86%) (MDR-Eb BSIs (1126 (38%)). The raw mortality rate was 18% in the overall population and 38% in the patient with GNB BSI. It was significantly higher for R GNB BSI (41 vs 36% for susceptible GNB BSI, p < 0.01). After adequate adjustment in a multivariate analysis, we showed that R-GNB BSI was significantly associated with mortality compared to susceptible strains (Fig. 1). By considering species sub-group, the effect was not significant for resistant Pseudomonas aeruginosa (p = 0.54) but remained significant when considering only Eb. Considering Eb resistance, the impact of 3GC R showed a trend to an increased mortality risk whatever there was no effect of IMI R (N = 45 (2%)) on prognosis. Limitation—The absence of information about antibiotic consumption may partly explain the remaining significant center random effect in the final models.

**Fig. 1:**
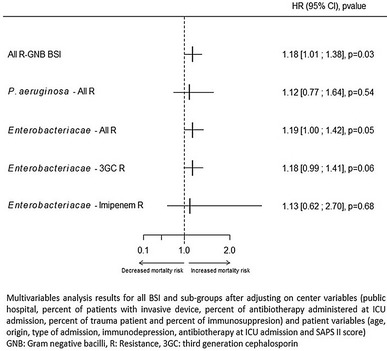
Multivariate analysis results for all BSI and sub-groups

**Conclusion:** in a large French database, after adequate adjustment on prognostic factors, Resistant BGN-BSI was associated with a higher ICU mortality than susceptible one. The effect was mainly due to Eb 3GC R.

## Posters

### P-01 Polytrauma: incidence and prognostic factors in 120 cases

#### Ferjani Wassim^1^, Romdhane Hayfa^1^, Ben Jdidia Bilel^1^, Azri Ines^1^, Saoudi Fedi^1^, Zaidi Bacem^2^, Barhoumi Mohamed Hafed^2^

##### ^1^CHU Habib Bourhuiba Sfax, Sfax, Tunisia; ^2^Kairouan, Tunisia

###### **Correspondence:** Ferjani Wassim - wassimferjani01@gmail.com

*Annals of Intensive Care* 2018, **8(Suppl 1):**P-01

**Introduction:** The polytrauma is by definition a life-threatening wounded in the short term. More than half of the preventable post-traumatic deaths are related to errors in the management strategy, lack of organization, or inexperience in the initial hospital structure. It is a rapidly evolving patient for whom the time factor is one of the pejorative components of its management. The objective of the study is to specify the socio-demographic characteristics of polytrauma patients and to identify the factors predictive of poor prognosis.

**Patients and methods:** This is a retrospective study of 120 polytrauma patients in the Anesthesia Reanimation department of the Ibn El Jazzar hospital in Kairouan during the year 2016.

**Results:** The average age of patients was 38 years. The majority of patients are male. In 80%, road accidents are the cause of polytrauma. The main lesions were—lesions of the musculoskeletal system in 92%, thoracic trauma in 90%, head trauma in 89%, maxillofacial trauma in 52.5%, trauma of the pelvis in 31%, abdominal trauma in 30.8% and spinal lesions in 19.1%. Gravity scores averaged 5.92 for RTS, 34 for ISS and 42 for IGSII. Surgical treatment was reported in 80% of patients. These were mainly orthopedic surgery in 47.5% and neurological surgery in 21.7% of cases. The use of intubation and ventilatory assistance was required in 57.5%. A secondary tracheotomy was performed in 16 patients. Mean duration of mechanical ventilation was 15 days. The length of stay in intensive care units was on average 10 days. The use of vasoactive drugs was necessary in 37% of patients. Complications were dominated by trophic disorders in 89% and mechanical complications of ventilation in 33% of cases. The mortality rate was 51.7%. On the balance of the multivariate study, the factors potentially influencing the mortality of polytrauma are + - the presence of severe head trauma (p = 0.001)—inhalation at intake (p = 0.037)—the presence of a state of shock at admission (p = 0.003)—the presence of initial hypoxemia (p = 0.042)—an initial hemoglobin level of less than 10 g (p = 0.036)—ISS score > 40 (p < 0.01) and IGSII > 45 (p = 0.034).

**Conclusion:** The management of polytrauma can not be improvised. The medical teams must be coordinated in pre-hospitalization. stabilizing this type of patient as quickly as possible, can improve the morbidity and mortality associated with early post-traumatic shock.

### P-02 Management of non-vital polytrauma patients in the Emergency Department: a single-center, retrospective study

#### Le Borgne Pierrick^1^, Bilger Luc^2^, Dascalu Elena^2^, Ugé Sarah^2^, Renfer Céline^2^, Kauffmann Philippe^2^, Quoirin Etienne^2^, Bilbault Pascal^2^

##### ^1^Hôpital de la Hautepierre, Strasbourg, France; ^2^CHU Strasbourg, Strasbourg, France

###### **Correspondence:** Le Borgne Pierrick - pierrick_med@yahoo.fr

*Annals of Intensive Care* 2018, **8(Suppl 1):**P-02

**Introduction:** Severe trauma remains a major issue worldwide. Management of these patients is mostly performed in ICUs. The radiological evaluation is essentialy based on performing a whole-body computed tomography (WBCT). However, less attention has been paid to the management of non-vital polytrauma patients in the Emergency Departments. The aim of this study was to evaluate the management of non-vital polytrauma patients who had a WBCT in our ED and to find predicting factors of severity at admission.

**Patients and methods:** Retrospective and monocentric study. We reviewed the chart of all patients who had a WBCT in 2014. We collected epidemiological, clinical and biological parameters and all therapeutic measures during the stay. A long-term survival follow-up was also performed. All patients admitted to the ICU were excluded.

**Results:** A total of 210 patients were included for statistical analysis and 64% (CI 95% [57.8–70.8]) of them had one or more lesion(s) in the WBCT. The mean ISS score was 10.1 ± 8.8. 42 severely injured patients (20% + CI 95% [14.6–25.4]) underwent urgent surgical procedures or were admitted to the ICU. The mean ISS score for these patients was 16.1 ± 10.8 compared to others 8.5 ± 7.5 in the remaining cohort (p < 0.0001). The mortality rate predicted by the TRISS model was 3.1% compared to 1.5% in the whole cohort (p < 0.0001). The average length of stay in the ED was 5.4 ± 2.9 h for the severely injured trauma patients versus 7.2 ± 4.6 h for the other patients of the study (p = 0.003), the average lenght of stay was 16.2 ± 18.9 days for the severely injured group versus 3.1 ± 6.4 days for the non-severely injured patients (p < 0.0001). In multivariate analysis, heart rate (> 100 min) and Vittel score (≥ 2 criterias) were related to the probability of belonging to the severely injured group (p = 0.03). The 24-hour mortality rate was 0.5% in the ED and the 30-day mortality rate was 1.5%.

**Conclusion:** The development of a network in the ED hosting non vital polytraumas remains crucial. Its primary goal will be to meet technical and time requirements and establish in-hospital triage algorithms based on clinical variables, in order to detect these patients at an early stage and offer them priority care in our overcrowded EDs.

### P-03 Epidemiology and prognosis of isolated cranial trauma caused by traffic accidents in ICU

#### Turki Olfa^1^, Chelly Hedi^1^, Regaieg Kais^1^, Bahloul Mabrouk^1^, Braidai Sabrine^1^, Ben Hmida Chokri^1^, Bouaziz Mounir^1^

##### ^1^CHU Habib Bourguiba, Sfax, Tunisia

###### **Correspondence:** Turki Olfa - olfa.turki.rea@gmail.com

*Annals of Intensive Care* 2018, **8(Suppl 1):**P-03

**Introduction:** The trauma of traffic accidents and particularly cranial trauma are, due to their frequency and severe consequences in both the short and long term, a real public health scourge on a global scale. Studies of the epidemiology of cranial trauma by traffic accidents and their prognosis are rare at least in underdeveloped or developing countries. In addition, the impact of extracranial lesions on cranial trauma prognosis has long been discussed. The purposes of our study were to examine the epidemiological aspects and to determine the factors correlated to the immediate and distant prognosis of isolated cranial trauma.

**Patients and methods:** Retrospective cohort spread over 4 years (from 2009 to 2012) and including 242 patients with isolated Cranial trauma by traffic accidents (mean age 30.9 years, sex ratio—7). We proposed to study the factors correlated with a poor prognosis in terms of death in hospital and Glasgow Outcome Scale (GOS) at 6 months unfavorable in dual analysis (univariate and then multivariate). For the GOS study, patients were divided into 2 groups—GOS favorable for patients with good recovery (GOS = 5), recovery with a light handicap (GOS = 4), GOS unfavorable for those having survived with a severe disability (GOS = 3), a vegetative or pauci-relational state (GOS = 2) and those who died (GOS class 1).

**Results:** Hospital mortality was 26% and the GOS at 6 months was distributed as follows: death (27.3%), vegetative state (2.9%), severe disability (11.2%), mild disability (28%) and good recovery (30.6%). The 6-month GOS was deemed unfavorable in 41.4% of the cases.

Various after effects were observed in survivors: physical (42%) dominated by headache (24.4%), sleep disorders (14.2%) and epilepsy (7.2%); memory disorders (27.8%) or concentration (16.5%) and finally emotional after effects (32.4%) with irritability (30.7%) and aggressiveness (9.7%).

In multivariate statistical analysis, independent predictors of mortality were arterial hypotension, hypoxia extradural hematoma (EDH),, acute subdural hematomas (SDH), diffuse axonal injury and ventilator associated pneumonia. Those correlated with an unfavorable GOS were an age ≥ 38 years, hypotension, cerebral edema, coma duration ≥ 5.5 days, EDH and H24 glucose ≥ 7.15 mmol/l.

**Conclusion:** Although the short-term prognosis of head trauma seems to be improved at present, the long-term consequences of cranial trauma remain fairly frequent, and often underestimated, which underlines the importance of their screening and their proper care.

### P-04 Prognostic factors in the polytrauma in the emergency Department of CHU Oran

#### Djebli Houria^1^, Benbernou Soumia^2^, Ghomari Nabil^1^, Azza Abdelkader^1^, Benterki Zoubir^1^

##### ^1^Laboratoire de recherche AVC, Oran, Algeria; ^2^Boulevard benzerjeb, Oran, Algeria

###### **Correspondence:** Djebli Houria - djeblihouria@yahoo.fr

*Annals of Intensive Care* 2018, **8(Suppl 1):**P-04

**Introduction:** Multiple trauma patients are mainly caused by road accidents (RA), which become the leading cause of death for men under the age of 40 in industrialized countries. This condition is common in Algeria because of the large number of RA. it is in this perspective that we wanted to carry out a study with the aim of knowing the clinical radiological characteristics of polytrauma patients and of knowing the risk factors for the occurrence of death.

**Patients and methods:** This is a retrospective and descriptive study of the observations of polytrauma patients admitted to the multifunctional intensive care unit of UMC CHU Oran between January 2014 and July 2016. Inclusion criteria: All injured patients presenting at least two lesions, one of which involves the vital prognosis. Exclusion Criteria: Patient with less than one hospitalized lesion in CHUOran Emergency Resuscitation Department. Data were entered, coded and analyzed using the statistical analysis software Epi Info ™ 7.2.0.1 version 27/06/2016 in English. A general description of the population was made (average age, distribution by sex, origin). The EXCEL 2016 was used after exporting data from the Epi Info software, the variable of interest is mortality. p value and Chi2 are calculated, with a significance level of 5%‬‬‬‬.

**Results:** Over the study period (31 months), 135 patients were enrolled in our study > 16 years of age, with an age range between 16 and 89 years. The mean age of the patients was 34.07 ± 15.38 years. The average age of the survivors (30.83 ± 13.08 years) was lower than the mean age of the deceased (36.81 ± 16.68). RA was the cause of the trauma in 76% of the cases followed by the fall found cat 17% of the patients. Prehospital care only concerned 4% of patients. The univariate analysis showed that the main factors of occurrence of death were age (p = 0.02), glasgow score (p = 0.0001) anisocoria (p = 0.0009), shock (p = 0.002) 26% of deaths were due to intracranial hypertension, haemorrhagic shock in 15% of patients and ARDS in 7% of polytrauma patients.

**Conclusion:** The management of polytrauma can not be improvised. The medical teams must be coordinated by an emergency physician in prehospital, a doctor anesthesiologist-resuscitator at the reception. Some systematic gestures such as preparation of the reception allow to optimize the management of the time.

### P-05 The prognostic factors of benign cranial trauma

#### Mayola Vanadiaku Ulrich^1^, Khaleq Khalid^2^

##### ^1^Hôpital Beausejour, Casablanca, Morocco; ^2^University Hassan II, Casablanca, Morocco

###### **Correspondence:** Mayola Vanadiaku Ulrich - ulrichvan2002@yahoo.fr

*Annals of Intensive Care* 2018, **8(Suppl 1):**P-05

**Introduction:** Benign cranial trauma is a major public health problem due to both its frequency and the health costs it creates. The aim of this study was to identify relevant clinical factors that could predict the achievement of brain CT and situations at risk for neurosurgical care and for which CT was a necessity.

**Patients and methods:** This is a 24 month prospective study, including 404 patients with benign traumatic brain injury (Glasgow coma score GCS ≥ 13), patients under 16 years of age and patients with GCS < 13 were excluded. Epidemiological, clinical, paraclinical, therapeutic and evolutionary parameters were studied. A multivariate and univariate statistical study was carried out to reveal the predictive factors of a CT anomaly and the predictive factors for the neurosurgical care. Data were entered and analyzed using SPSS 16.0 and Excel 2007 software.

**Results:** The average age of patients was 34 years with a predominance of male, and sex ratio of 4.05. The cause of the BTB was mainly represented by the accidents of the public road in 73.27% of the cases. 28.22% of the patients were asymptomatic, the most common symptomatology was dominated by the initial loss of consciousness (52.72%), headache (35.64%). The Glasgow Coma Score was distributed as follows—GCS 15 (54.95%), GCS 14 (35.9%) and 13 (9.15%). 38.9% of patients had clinical signs of trauma to the skulland or face. Brain CT was performed in 88.4% of patients, and 25.5% had abnormal CT. The use of neurosurgical care was of the order of 3.22%. In univariate analysis—the predictive factors for a CT abnormality were the Intoxication during the brain trauma, the GCS < 15, Signs of trauma in the skull face, the vomiting, the Initial loss of consciousness, the Comitial crisis and the predictive factors of neurosurgical care were the GCS < 15, the anisocoria, Headache, the vomiting, the amnesia, the Initial loss of consciousness, the Comitial crisis, the anormal CT, the Extradural hematoma or the Subdural hematomat In multivariate analysis-the predictive factors for a CT abnormality were the GCS < 15, the Initial loss of consciousness and the predictive factors for the use of neurosurgical care were the GCS < 15, Signs of trauma in the skull face, the amnesia, the Comitial crisis, the HSD.

**Conclusion:** an algorithm must be applied in collaboration between resuscitators and neurosurgeons to improve the quality of benign cranial trauma management.

### P-06 Prognostic value of hyperchloremia in patients with traumatic brain injury: a prospective observational study

#### Taghouti Sarah^1^, Jendoubi Ali^2^, Marzouk Mahmoud^1^, Charradi Nesrine^1^, Riahi Yosra^1^, Ghedira Salma^1^, Houissa Mohamed^1^

##### ^1^University Tunis El Manar, Tunis, Tunisie; ^2^Hôpital Charles Nicolle, Tunis, Tunisie

###### **Correspondence:** Taghouti Sarah - sarah_17_88@hotmail.fr

*Annals of Intensive Care* 2018, **8(Suppl 1):**P-06

**Introduction:** Background—Traumatic brain injuries (TBI) are a major public health problem. They are the leading cause of death among those aged less than 40 years. Hyperchloremia is a common electrolyte disturbance in patients with TBI. Hyperchloremia has been associated with increased morbidity and mortality in critically ill patients + however, its prognostic significance in TBI patients is poorly documented. The aim of this study is to describe the prevalence and outcomes of hyperchloremia in patients with TBI admitted to the intensive care unit.

**Patients and methods:** In a prospective design, we included 43 consecutive patients with TBI (37 males + median age—52 years) admitted to the ICU in Charles Nicolle Hospital of Tunis from Mars to September 2017. Adult patients (aged ≥ 18 years) with isolated TBI or associated with minor extra-cranial injuries (defined as all non-head Abbreviated Injury Scale < 3) were included. Hyperchloremia was defined as a chloride level > 110mEg/l. Clinical and laboratory variables were compared between survivors (n = 36) and non-survivors (n = 7). We assessed the association between hyperchloremia 24-h post-admission and 30-day mortality. p < 0.05 was taken to indicate statistical significance.

**Results:** The median SOFA score at T0 was 7 points and the median IGS 2 score was 32 points. The median ISS was 25 points. There were 11 cases of mild head injury, 12 moderate head injury and 20 severe head injury. The 30-day mortality was 16%. Hyperchloremia occurred in 15 patients (35%) and the incidence was significantly different between survivors and non-survivors (28 vs. 72%, respectively, p < 0.001). In addition to hyperchloremia (p = 0.018), other laboratory variables were associated with 30-day mortality—hypernatremia (p = 0.011) and hypoalbuminemia (p = 0.024).

**Conclusion:** Hyperchloremia 24-h post-admission was associated with 30-day mortality in patients with TBI. This index could be useful prognostic marker. Efforts should focus on the prevention of hypernatremia and hyperchloremia in this vulnerable group of critically ill patients.

### P-07 Child traumatic brain injury

#### Naili Amine^1^

##### ^1^Blida Rp, ALGÉRIE

###### **Correspondence:** Naili Amine - drnailiamine@yahoo.fr

*Annals of Intensive Care* 2018, **8(Suppl 1):**P-07

**Introduction:** Brain injury in children is common and mild in most cases, but it remains the leading cause of death and disability in children over 1 year of age worldwide. The peculiarity of the child is that he possesses not mature brain and that the consequences of injuries acquired by traumatic brain injury can lead to the loss of capacities, as well as the non-acquisition of function, but above all the risk impact on learning abilities. The objective of the study is to define the incidence rate of cranial trauma in children as well as the mortality and morbidity of this scourge which presents a major public health problem.

**Patients and methods:** It is a descriptive retrospective study of a series of 186 children hospitalized in neuro-resuscitation service during the period January 01 to December 31, 2016, including 22 children admitted for cranial trauma. Clinical, para-clinical, etiological and therapeutic data were collected from hospitalization records.

**Results:** In a series of 186 children hospitalized during the defined period, 22 children were admitted for cranial trauma, i.e. a frequency of 12%. The average age was 6 years [6 h of life-14 years], with a sex ration of 1 Among the 22 children, 12 had severe head trauma, a rate of 55% + whose causes are variable—6 road accidents, 3 domestic accidents, 2 traffic accidents, and 1 obstetric accident, admitted with a pediatric Glasgow score between 5 and 10, and all required mechanical ventilation of the 22 head trauma, 10 were operated for different lesions—3 extra-dural hematomas, 3 cranio-cerebral wounds, 2 sub-dural hematomas, 1 decompressive craniectomy, and 1 embarrure. 02 children had died following severe head trauma, i.e. a mortality rate of 9%, the morbidity rate of head trauma in the Tipaza wilaya was 1.1 100,000 children year, the average length of stay in intensive care units was 22 days, with several complications of decubitus, and functional due to the primary and secondary lesions of the cranial trauma.

**Conclusion:** The head trauma of the child is a public health problem, its functional prognosis can be dramatic when it is severe, its management must be early and multidisciplinary.

### P-08 Factors associated with lung contusions in critically ill trauma children

#### Turki Olfa^1^, Chaari Anis^1^, Bahloul Mabrouk^1^, Ammar Rania^1^, Chelly Hedi^1^, Ben Hmida Chokri^1^, Bouaziz Mounir^1^

##### ^1^CHU Habib Bourguiba, Sfax, Tunisia

###### **Correspondence:** Turki Olfa - olfa.turki.rea@gmail.com

*Annals of Intensive Care* 2018, **8(Suppl 1):**P-08

**Introduction:** The aim of the study was to identify factors predicting lung contusion in trauma children.

**Patients and methods:** Retrospective study conducted for a period of 8 years (January 01, 2005–December 31, 2012) in a medical surgical intensive care unit. All trauma patients younger than 15 years were included. Two groups were compared—those with lung contusions (C + group) and those without lung contusions (C − group).

**Results:** We included 330 patients. The mean (SD) age was 7.6 (4.3) years. Chest injury was diagnosed in 70 patients (21.2%). All our patients needed mechanical ventilation. Lung contusions were diagnosed in43 patients (13% of all patients and 61.4% of patients with chest trauma). In multivariate analysis, independent factors predicting lung contusion were road traffic accident (odds ratio [OR], 3.2 + 95% confidence interval [CI], 1.2–8.6 + p = 0.019), increased Pediatric Risk of Mortality (PRISM) score (OR, 1.1 + 95% CI 1.1–1.2 + p = 0.017), hepatic contusion (OR 4.8 + 95% CI 1.3–17.1 + p = 0.017), and pelvic ring fracture (OR, 3.5 + 95% CI 1.1–10.5 + p = 0.026). Death occurred in 46 patients (13.9%). Intensive care unit mortality was significantly higher in the C + group (OR, 2.5 + 95% CI 1.2–5.4 + p = 0.021). However, mortality was not differentbetween the 2 groups after adjusting for PRISM score (OR, 1.2 + 95% CI 0.5–2.9 + p = 0.752) or after adjusting for Injury Severity Score (OR, 0.7 + 95% CI 0.3–2.1 + p = 0.565).

**Conclusion:** Lung contusion is common in critically ill children with chest trauma. The diagnosis should be considered in patientswith road traffic accident, increased PRISM score, hepatic contusion, and pelvic ring fracture.

### P-09 Evaluation of bougie-assisted chest tube insertion technique for the drainage of post-traumatic pleural effusions

#### Ait Mamar Bouziane^1^, Bouchiha Nabil^2^, Lobo David^2^, Mounier Roman^2^, Martin Mathieu^2^, Attias Arié^2^, Cook Fabrice^2^, Dhonneur Gilles^2^

##### ^1^CHU Henri Mondor - APHP, Créteil, France; ^2^Créteil, France

###### **Correspondence:** Ait Mamar Bouziane - bouziane.ait-mamar@aphp.fr

*Annals of Intensive Care* 2018, **8(Suppl 1):**P-09

**Introduction:** Chest trauma is often associated with pleural effusion (hemothorax and or pneumothorax). Drainage of the pleural space by a chest tube is a common intervention in such situations. Blunt dissection technique with a Kelly clamp is preferred to classical trocar techniques to prevent severe complications, like perforation of thoracic or abdominal organs. Despite these precautions, malposition remains the most common complication of tube thoracostomy. We investigated a new technique of bougie-assisted chest tube insertion to prevent chest tube malposition after chest drainage of post traumatic pleural effusion.

**Patients and methods:** We performed a controlled before-and-after study to assess the ability of a bougie-assisted chest tube insertion technique, compared to a standard blunt dissection technique, to prevent chest tube malposition. For the bougie-assisted group, we used a disposable Eschmann-style bougie, commonly used to guide the endotracheal tube during difficult intubations. Technique consisted in blunt dissection until the parietal pleura is opened. Thoracostomy tube was preloaded onto the bougie and bougie was advanced alongside the finger, with apical or caudal direction after entering the chest cavity, depending on the type of pleural effusion. Thoracostomy tube was then advanced forward utilizing a Seldinger technique. The primary end point was optimal position of the chest tube. The tube position was blindly assessed on standard chest X-ray. In pneumothorax, optimal position was apical (above the aortic arch), and in hemothorax or mixed-effusion it was basal (2 cm above the diaphragm or lower).

**Results:** A total of 58 patients were enrolled (bougie-assisted—n = 30 + conventional—n = 28). Chest tubes were optimally position in 29 (96%) in bougie-assisted group and 12 (43%) in conventional group, OR 38.5, IC 95% = [4.6–325.16], p < 0.0001. Efficacy of chest drainage (defined on chest X-ray as the absence of visible pleural line for pneumothorax and as a clear costophrenic angle for hemothorax) was assessed in 29 (96%) in bougie-assisted group and in 15 (53%) in conventional group, OR 25.13, IC 95% = [3.00–210.93], p < 0.001. Average procedure time was 281 s (95% CI 120–360 s) for bougie-assisted group and 558 s (95% CI 318–900 s) for conventional group, p < 0.0001. No severe complication was observed in both groups.

**Conclusion:** Bougie-assisted chest tube insertion technique prevents chest tube malposition, is safe, effective and shortens procedure time for the post traumatic pleural effusion drainage.

### P-10 The Consommation of Antibiotics in plastic surgery

#### Khaleq Khalid^1^, Zaouit Maryam^2^, Derfoufi D^1^, Zerouali K^1^, Alharrar R^1^, Ezzoubi M^1^

##### ^1^Centre Hospitaliser Universitaire Ibn Rochd, Casablanca, Morocco

###### **Correspondence:** Khaleq Khalid - khaleq20@gmx.fr

*Annals of Intensive Care* 2018, **8(Suppl 1):**P-10

**Introduction:** Infectious complications determine the prognosis of burned patients. However, the emergence of bacterial resistance to antibiotics threatens treatment efficacy, which is due to an inadequate antibiotic consumption inqualitative and quantitative terms. The objective of this study was to describe the profil of consumptionand susceptibility to antibiotics. And, to explore the predictive factors for theemergence of MRB in the service of burns and plastic surgery.

**Patients and methods:** It is a retrospective study including 122 severe burnedpatients hospitalized for 2 years in the plastic surgery department of theuniversity hospital Ibn Rochd from January 2015 to December 2016. Bacterialecology was described, and the distribution of the seeds by group, by species andby period of time was detailed. The DDD 1000JH (daily defined dosage reportedin 1000 days of hospitalization) was used to assess the consumption of antibiotics. P correlation coefficients were calculated to explore the association betweenconsumption of antibiotics and the emergence of the BMR (Multiresistantbacteria), and identified predictors of this emergence.

**Results:** On 512 samples taken, 539 bacterial and fungal strains were identified, with a predominance of P. aeruginosa (21.15%), A. baumani i (13%) and S. aureu s (13%), the number of strains increased with the duration of the stay reaching itsmaximum from 28 days in hospital. The ceftazidine (212.7 DDD 1000DH), imipenem (139.7 DDD 1000DH), and amikacin (122.4 DDD 1000DH) were themost used antibiotics during our study, also + the profile of consumption increasedbetween 2015 and 2016.362 BMR were isolated + the EBLSE were at the top (24.5%) follow up of theCRPA (21.8%), followed by the IRPA (20.7%) follow-up of the CRAB (17.1%) then the IRAB (13.5%) and finally the MRSA with a portion of 2.2%. The profile of bacterial resistance has varied significantly for severalantibiotics bacteria pairs.

**Conclusion:** It remains difficult to show correlations between antibioticconsumption and bacterial resistance. However, these data are particularly usefulin the epidemiological surveillance of bacteria to better guide probabilisticantibiotic therapy.

### P-11 Obstetrical critical care in a subsaharan Intensive Care Unit (ICU)

#### Traoré Salah^1^, Bako Yves Paul^1^, Kabré Bamweleguedé Yvette^1^, Ki Kelan Bertille^1^, Bougouma Check Tidiane W. H.^1^, Simporé André^1^, Kaboré R Armel Flavien^1^, Sanou Joachim^1^, Ouédraogo Nazinigouba^1^

##### ^1^CHU Yalgado Ouédraogo, Ouagadougou, BURKINA FASO

###### **Correspondence:** Traoré Salah - seif_tis@yahoo.fr

*Annals of Intensive Care* 2018, **8(Suppl 1):**P-11

**Introduction:** Care delivery in severely ill obstetric patients remains problematic in developing countries, due to many factors such as poverty, sociocultural factors and paucity of research. The aim of this study is to describe the epidemiology, clinics and outcomes of the obstetric patients admitted in the ICU of a subsaharan country.

**Patients and methods:** It was a descriptive study including all obstetric patients admitted in the ICU during a 5 year period. We assessed their sociodemographic characteristics, clinical features and outcome.

**Results:** During the study period, among the 1686 patients admitted in the ICU, 174 were obstetric patients, representing 10.3% of the admissions. They were mainly young (mean age 27.7 ± 7.0 years), unschooled (53.3%) and unemployed (82.2%). Most of them were primiparous (29.3%) and had no clinical check during pregnancy (41.4%). A large number (89.8%) was admitted in ICU after delivery, 7% after abortion and 3.2% were still pregnant. Thirty-seven per cent (37%) of them had medical history of disease that can lead to obstetrical complication. The major causes of admission were severe sepsis (28.0%), eclampsia (21.0%) and hemorrhage (10.2%). Mortality was 39.5 and 67.7% of the deaths occurred within 72 h after admission. Infectious diseases were the major cause of mortality (56.5). Mortality rate of the neonates was 40.5%.

**Conclusion:** Mortality of critically ill obstetric patients admited in the ICU is high in our teaching hospital as in other developping countries and due to septic complications, éclampsia and hemorraege. Some initiatives most be taken to improve quality of obstetric critical care and identification of near miss cases in developping countries.

### P-12 Eclampsia in a third level Tunisian hospital: from January 2004 to December 2016

#### Khedher Mouna^1^, Ferhi Fehmi^1^, Nasr Amal^1^, Nasr Amal^1^, Gardabou Omarkais^1^, Helel Najet^1^, Chaabani Marwa^1^, Nasr Amal^1^, Rahma Herch^1^, Khalil Tarmiz^1^, Khaled Benjazia^1^

##### ^1^Farhat Hached University Teaching Hospital, Sousse, Tunisia

###### **Correspondence:** Khedher Mouna - mounakhedher85@gmail.com

*Annals of Intensive Care* 2018, **8(Suppl 1):**P-12

**Introduction:** Eclampsia is a rare but serious threat to maternal and fetal well-being. The aim of this study was to assess the incidence of eclampsia and its morbidity and mortality.

**Patients and methods:** We conducted a retrospective survey in a third level Tunisian university teaching hospital from January 2004 to December 2016. We included all patients with the diagnosis of eclampsia.

**Results:** In study period 129847 deliveries were registered. 83 women with eclampsia were identified hence the incidence of eclampsia was 6.39 per 10000 deliveries. The median gestational age at the time of eclampsia was 36 weeks. No maternal deaths due to eclampsia were recorded. The delivery mode was caesarean section in 100% of eclamptic patients. The recurrence of eclampsia despite magnesium sulfate prevention was observed in 6% of patients. Severe complications of eclampsia were recorded in 10.8% of patients—4 posterior reversible encephalopathy syndrome, 2 acute pulmonary edema, and 3 HELLP syndrome. 10.8% of new born were preterm. There were 5 stillbirths and 2 neonatal deaths.

**Conclusion:** The incidence of eclampsia was very high probably due to center effect. It’s essential to raise awareness among mothers in the community regarding early signs and symptoms of preeclampsia eclampsia and to design a better tracking system for antenatal care program.

### P-13 Study of maternal mortality in maternity level 3 from 1990 to 2015

#### Sellami Walid^1^, Rachdi Mohamed Amine^1^, Ben Mrad Ines^1^, Hajjej Zied^1^, Sammoud Walid^1^, Bousselmi Radhouene^1^, Yengui Olfa^1^, Gharssallah Hedi^1^, Labbene Iheb^1^, Ferjani Mustapha^1^

##### ^1^Hôpital militaire de Tunis, Tunisia

###### **Correspondence:** Sellami Walid - drsellamiwalid@yahoo.fr

*Annals of Intensive Care* 2018, **8(Suppl 1):**P-13

**Introduction:** To monitor maternal mortality which is an indicator of the quality of obstetrical care and anesthesia resuscitation, Our country worked to set up several programs targeting maternal and child health. The aim of this work was—To evaluate the maternal mortality rate in our department and its evolution. To Identify the cause of death and classify it depending on whether it is preventable or not. To spot the deficiencies either in the care management or the organization of the care system. To propose ways to improve our care and to fill the failures.

**Patients and methods:** It was a retrospective study about maternal death, performed at the Department of Gynecology and Obstetrics, over a 25-year period (from 1990 to 2015), that have reported 14 cases of maternal death according to the World Health Organization definition.

**Results:** The Maternal Mortality Rate (MMR) was 22.43 for every 100,000 live births. The average age of our patients was 32.3 years. The main risk factors for maternal mortality are unfavorable socioeconomic conditions, high-risk pregnancies, multiparity, primiparity and a poor follow-up of the pregnancy. The main causes of maternal death are represented by direct obstetric causes (80%) allocated as follows—postpartum hemorrhage (45%), pregnancy toxemia (14%), acute fat hepatic steatosis (7%), infection (7%) and complications of anesthesia (7%). Indirect obstetric causes were found in 20% of deaths. Death was considered avoidable in 78.58% of cases.

**Conclusion:** At the end of this work, we were able to pull several recommendations in order to reduce M.M.R. Health education. Facilitate access to care for the parturient, improve care and conditions of childbirth. Continuous training of the medical and paramedical staff.

### P-14 Impact of hyperoxemia on the prognosis of patients with septic shock subjected to pre-hospital invasive ventilation

#### Jouffroy Romain^1^, Saade Anastasia^1^, Castres Saint Martin Laure^1^, Philippe Pascal^1^, Idialisoa Rado^1^, Carli Pierre^1^, Vivien Benoit^1^

##### ^1^Hôpital Necker Enfants Malades, Paris, France

###### **Correspondence:** Jouffroy Romain - romain.jouffroy@gmail.com

*Annals of Intensive Care* 2018, **8(Suppl 1):**P-14

**Introduction:** Mechanical ventilation can help improve the prognosis of sepsis. While adequate delivery of oxygen to tissue is crucial, hyperoxemia may be deleterious. Invasive out-of-hospital ventilation is often promptly performed in life-threatening emergencies. We propose to determine whether the arterial oxygen pressure (PaO2) at intensive care unit (ICU) admission affects mortality at day 28 (D28) in patients with septic shock subjected to mechanical out-of-hospital ventilation.

**Patients and methods:** We performed a monocentric retrospective observational study on 77 patients with septic shock admitted to the ICU. PaO2 was measured at ICU admission in patients subjected to invasive ventilation before any hospital admission. The primary outcome was mortality at day 28 (D28).

**Results:** Forty-nine (64%) patients with septic shock were mechanically ventilated before any hospital admission and transferred to the ICU. The mean PaO2 at ICU admission was 153 ± 77 and 202 ± 82 mmHg for alive and deceased patients at D28, respectively. PaO2 was significantly associated with mortality at D28 (p = 0.04). Using a ROC curve, the corresponding AUC was 0.70 [0.54–0.85]. For a PaO2 > 150 mmHg, the OR for mortality at D28 was 4.20 [1.25–15.95] (p = 0.02), whereas for a PaO2 < 150 mmHg, the OR was 0.24 [0.06–0.80] (p = 0.02).

**Conclusion:** In this study, we report a significant association between hyperoxemia at ICU admission and mortality at D28 in patients with septic shock subjected to pre-hospital invasive mechanical ventilation. The adjustment of the PaO2 is a crucial prognosis factor in patients with septic shock subjected to invasive out-of-hospital ventilation to avoid the toxic effects of hyperoxemia. However, blood gazometry is hard to get in a prehospital setting. Consequently, alternative and feasible measures are needed, such as pulse oximetry, to improve the management of prehospital invasive ventilation.

### P-15 Benefits of control CT-scan 24 h after thrombolysis in acute pulmonary embolism

#### Jamoussi Amira^1^, Jarraya Fatma^1^, Ayed Samia^1^, Merhebene Takoua^1^, Hantous Saoussen^1^, Ben Khelil Jalila^1^, Besbes Mohamed^1^

##### ^1^Hôpital Abderrahmen Mami, Ariana, Tunisia

###### **Correspondence:** Jamoussi Amira - dr.amira.jamoussi@gmail.com

*Annals of Intensive Care* 2018, **8(Suppl 1):**P-15

**Introduction:** Nowadays, benefit of enhanced CT-scan in positive diagnosis of acute pulmonary embolism (PE) is well established. It also allows evaluation of PE’s burden on the right heart and shows several signs of acute cor pulmonale (ACP). Objectives -We aimed to assess benefits of control CT-scan 24 h after thrombolysis in acute PE.

**Patients and methods:** We retrospectively enrolled patients with confirmed PE whom have been thrombolysed between January 2014 and August 2017 and controled with an enhanced CT-scan 24 h after thrombolysis. Assessement criteria were: Qanadli obstruction index; Signs of ACP—right ventricle diameter left ventricle diameter (RVD LVD) and paradoxical interventricular septum (IVS). Non inclusion criteria were: lack of initial or control CT-scan.

**Results:** During the study period (3 years and 8 months) we admitted 1790 patients from whom 98 patients had acute PE (5.5%). Very severe patients that were thrombolysed as rescue therapy without initial CT-scan and those who died before control CT-scan were not included. We enrolled 33 patients—high risk mortality PE (n = 10, 30.3%) and intermediate high risk PE (n = 23, 69.7%). Mean age was 60 years and sex-ratio was 1.06. At admission, mean severity scores were 22.1 ± 9.4 for SAPS II and 7.6 ± 4.2 for APACHE II. Evolution criteria are listed in Table 1.

**Table 1:** Comparison of tomography assessment criteria before and after 24 after thrombolysis

	Initial CT-Scan (n = 33)	Control CT-Scan (n = 33)	p
OI % mean [extremes]	53.9 [20–85]	36.5 [0–75]	< 10^−3^
RVD/LVD > 1 n (%)	31 (93.9)	20 (60.6)	0.001
Paradoxical IVS n (%)	24 (72.7)	9 (27.3)	< 10^−3^

*OI* Obstruction Index, *RVD/LVD* right ventricle diameter/left ventricle diameter, *IVS* interventricular septum

**Conclusion:** Control CT-scan is highly useful 24 h after thrombolysis. It allows evaluation of response to pharmacological thrombolysis of acute PE and shows significative resolution of arterial obstruction degree and signs of ACP.

### P-16 Thromboembolic disease in medical intensive care: evaluation of risk factors, WELLS and GENEVE scores in a Moroccan department

#### Ezzouine Hanane^1^, Soussane Mehdi^1^, Eljadid Siham^1^, Benslama Abdellatif^1^

##### ^1^Centre Hospitalier Universitaire Ibn Rochd, Casablanca, Morocco

###### **Correspondence:** Ezzouine Hanane - ezzouinehanane@yahoo.fr

*Annals of Intensive Care* 2018, **8(Suppl 1):**P-16

**Introduction:** Venous thromboembolic disease (VTE) is a major cause of morbidity and mortality for patients admitted to ICU. Recognition of risk factors is an essential element of management. the objective of our study is to evaluate the risk factors of MTEV in a Moroccan medical resuscitation department, the WELLS and GENEVE scores and the specificities of our patients.

**Patients and methods:** We carried out a retrospective study in the medical resuscitation department of the Ibn Rochd hospital in Casablanca, Morocco for 1 year (January to December 2015). All patients admitted to the department were included during the study period and the parameters evaluated and analyzed were the epidemiological, clinical, risk factors for VTE, the WELLS and GENEVE scores.

**Results:** 354 patients were included in the study. Their mean age was 42.9 years with a male predominance of 53.4%. 29.7% of patients were diabetic and 33.05% of admissions were for neurological disease. APACHE II average was 10.47 and SAPS II mean at 21.77. The average length of hospitalization was 7.64 days. 47.5% required mechanical ventilatory assistance and the duration of intubation was on average 3.71 days. 29.7% vascular prosthesis.81.4% received thromboembolic drug therapy. Low-molecular-weight heparin was used 70.9%. The thromboembolic risk assessment by the WELLS score noted that 70.3% had a low risk 28.5% had an average risk. For the GENEVE score, 1.4% were at high risk and 69.8% were at medium risk. Overall patient outcome was favorable in 62, 7%. 8 patients developed deep venous thrombosis and had an average risk factor of 23.8 l. 3 patients developed pulmonary embolism had an average risk factor of 23.6.

**Conclusion:** The risk of thromboembolism in a medical resuscitation environment requires an evaluation for each patient admitted. Thromboembolic prevention must be adapted to each level of risk. In our series, the low risk according to the Wells score is predominant as well as the intermediate risk according to the Geneva score. The choice of thrombophylaxis is a major determinant in the management in medical resuscitation.

### P-17 Dare to decorate your Intensive Care Units during the Christmas holidays!

#### Beaudeau Lauren^1^, Lebut Jordane^1^, Henry Aline^1^, Jorge Sabrina^1^, Podeur Anaelle^1^, Dijoux Mathilde^1^, Roig Coralie^1^, Kaddour Jean Philippe^1^, Solbiac Sonia^1^, Partenet Frédérique^1^, Serrat Françoise^1^, Nicollet^1^, Martine, Joffredo Emilie^1^, Roucaud Nicolas^1^, Le Meur Matthieu^1^, Lau Nicolas^1^, Paulet Rémi^1^, Thyrault Martial^1^

##### ^1^CH2 V - site Longjumeau, Longjumeau, France

###### **Correspondence:** Beaudeau Lauren - lauren.beaudeau@gmail.com

*Annals of Intensive Care* 2018, **8(Suppl 1):**P-17

**Introduction:** In December 2016, after cancellation of the budget for a Christmas tree, the nurses and caregivers of the night team spontaneously made and hung Christmas decorations in our Intensive Care Unit to make patients and their families feel better. The context was difficult with controversies around secularity. The town of Paray Le Monial had been forced to remove a Nativity scene and the city of Melun had been criticized for setting one up. So we found it important to assess the perception of the approach by patients and relatives.

**Patients and methods:** Decorations -Hand-colored patterns about Christmas theme printed on A4 paper Decorations brought by the staff or already possessed by the unit—Christmas balls, garlands, silver starDecorations made with service equipment—Christmas tree consisting of inflated non-sterile gloves, cardboard, figurative Nativity scene without a recognizable figure in a cardboard box with cotton, bed sheet to simulate snow. Evaluation -All visitors and conscious patients received an anonymous single choice questionnaire with numerical scale and free fields from December 15th to December 31st, 2016.

**Results:** 59 answers were received, including-No negative opinion. 1 neutral answer by a person who had not noticed the decorations. 54 positive or extremely positive opinions. No answer without data. The comments pointed out the originality, the good idea, the warm comforting side. Some asked for more decorations. Others found them sober.

**Table:** 

Numeric scale	1	2	3	4	5	6	7	Total
Opinion	Shocked	Unpleasant	Inappropriate	No opinion	Why not	Good	Great	
Answers (%)	0	0	0	1 (1.7)	4 (6.8)	25 (42.4)	29 (49.1)	59
Free comments								
With (%)	0	0	0	1 (1.9)	4 (7.9)	21 (41.2)	25 (49)	51
Without (%)	0	0	0	0	0	4 (50)	4 (50)	8

**Discussion:** The results show the good perception of the spontaneous action by the patients and their relatives. There was no negative response, particularly offend persons. However, it is possible that relatives or patients with negative opinions did not dare to express themselves. The initiative demonstrated a good cohesion of the night paramedical team, encouraging the interns and the day teams to take part in the coloring of the decorations. The initiative was initially aimed at the wellbeing of the patients and their relatives. However it has enabled an activity similar to preventing psychosocial risks among the healthcare team, allowing them to adopt a positive attitude in their approach to care. In addition, no significant costs were incurred thanks to the use of cheap materials, mainly recycled cardboard and standard quality white paper.

**Conclusion:** The spontaneous decoration of our Intensive Care Unit by the night care team was very well received by the patients, their families and their relatives. The initiative also made possible to enhance team cohesion and to value it. The associated costs were negligible.

### P-18 Arrhythmias and blood pressure variability during physicians’ night shifts in intensive care unit

#### Khedher Mouna^1^, Ferhi Fehmi^1^, Gardabou Omarkais^1^, Gharbi Anissa^1^, Chrigui Noura^1^, Assidi Bassem^1^, Bouslama Mohamed Amine^1^, Khalil Tarmiz^1^, Khaled Benjazia^1^

##### ^1^Farhat Hached University Teaching Hospital, Sousse, Tunisia

###### **Correspondence:** Khedher Mouna - mounakhedher85@gmail.com

*Annals of Intensive Care* 2018, **8(Suppl 1):**P-18

**Introduction:** Shift work is associated with an increased rate of cardiovascular diseases and accidents. The work organization in intensive care units with fixed daily working hours and additional schedules during the guard when residents is called upon to complete 24 h of continuous work, deserve special attention. The aims of this study is to evaluate the effects of a 24 h intensivist’s night shift on 24 h electrocardiogram (ECG), heart rate variability and blood pressure (BP).

**Patients and methods:** Forty-five healthy intensivists from 3 Tunisian University Teaching Hospitals were included. Risk factors for cardiovascular disease, lifestyle, incidents, conflicts, stressful situations, sleep deficit, and resynchronization of circadian rhythms during the 24 h of guard were evaluated. Twenty-four hours ECG and BP monitoring were performed after reference electrocardiogram. Participants were instructed to fill out an event diary.

**Results:** The median age was 29.8 years (range 28–31.0) + the sex ratio was 1.8 1. Twenty-four hours ECG showed a higher rate of ventricular premature beats (VPB) during early morning hours and increased low-frequency normalized units during night shift. The most observed abnormalities were—sinus tachycardia, sinus bradycardia, sinus pause, atrial premature beats, supraventricular tachycardia, and premature ventricular contractions. BP monitoring revealed a greater diastolic BP throughout 24 h as well as during night-time. Systolic BP higher than 125 mmHg during sleep time was observed in 55% of participants. The frequency of arrhythmias and blood pressure variability are correlated with the increase in work stress and conflicts.

**Conclusion:** Our results highlight the extent incidence of arrhythmia and blood pressure variability during intensive care unit night’s shift probably due to the increased neuroendocrine stress response.

### P-19 Acute olanzapine poisoning in the intensive care unit: clinical features and pharmacokinetic data

#### Bérezel Ouissam^1^, Chevillard Lucie^1^, Gourlain Hervé^1^, Malissin Isabelle^1^, Deye Nicolas^1^, Voicu Sebastian^1^, Megarbane Bruno^1^

##### ^1^Hôpital Lariboisière, Paris, France

###### **Correspondence:** Bérezel Ouissam - ouissam.berezel@gmail.com

*Annals of Intensive Care* 2018, **8(Suppl 1):**P-19

**Introduction:** Olanzapine, an atypical 2nd generation antipsychotic drug, is increasingly prescribed due to an improved efficiency to control chronic psychosis with reduced tolerance in comparison to the 1st generation antipsychotic drugs. Whereas side-effects are well-reported, acute poisonings remain less documented.

**Patients and methods:** We conducted a retrospective single-center descriptive study including all patients admitted in the intensive care unit (ICU) of a University Hospital between 01 2000 and 06 2017 in relation to olanzapine overdose, defined by the onset of compatible features and the increase in plasma olanzapine concentrations. The usual demographic, clinical, toxicological and outcome data were collected. When available, non-compartmental pharmacokinetics was modeled and the different parameters determined using WinNonlin v. 5.1 software.

**Results:** Seventy-one patients (38 females (54%) 33 males (46%) + age—36 years [27 + 44] (median [percentiles 25 + 75] + 55% with past suicide attempts + 18% of chronic alcoholics) were included. Multidrug ingestion (3 co-ingestants [2 + 4] with mainly psychotropic drugs including benzodiazepines in 69%) included olanzapine (ingested dose—200 mg [110 + 333] and plasma concentration on admission—0.310 mg L^−1^ [0.185 + 0.820]. Features consisted in consciousness impairment (Glasgow coma score—4 [3 + 8]), myorelaxation (68%), myosis (39%) or mydriasis (10%), abolished (31%) or increased tendon reflexes (28%), QT widening (23%) and QRS enlargement on the ECG (10%). No seizure was observed. Management included mechanical ventilation (91%), catecholamine infusion (18%), gastrointestinal decontamination (10%) and hemodialysis (4%). Three patients (4%) who presented pre-hospital cardiac arrest died in the ICU. Pharmacokinetic analysis suggested prolonged olanzapine elimination at elevated concentrations, attributed to the reduction in liver metabolism and renal clearance.

**Conclusion:** Olanzapine poisoning may result in ICU admission with life-threatening central nervous depression, cardiac toxicity and fatality risk. Co-ingestions including benzodiazepines may have masked the presence of olanzapine-induced anticholinergic manifestations. Olanzapine elimination in overdose is altered contributing to the clinical severity of poisoned patients.

### P-20 Acute amitryptilin and clomipramine poisonings in the intensive care unit—clinical presnetation and pharmakinetic modelling

#### Meng Hélène^1^, Chevillard Lucie^1^, Mihoubi Mohamed El Amine^1^, Malissin Isabelle^1^, Nuzzo Alexandre^1^, Peron Nicolas^1^, Megarbane Bruno^1^

##### ^1^Hôpital Lariboisière, Paris, France

###### **Correspondence:** Meng Hélène - helene.meng@aphp.fr

*Annals of Intensive Care* 2018, **8(Suppl 1):**P-20

**Introduction:** Amitriptylin (AMI) and clomipramine (CLO), two tricyclic antidepressants, are responsible for severe poisonings with anticholinergic encephalopathy, seizures and membrane stabilizing effects. Their prescriptions have decreased since 30 years following the marketing of the serotonin reuptake inhibitor (SRI) antidepressants. Our objective was to report a series of AMI and CLO poisonings in the era of SRI supremacy with the description of the clinical manifestations and pharmacokinetic data.

**Patients and methods:** We conducted a retrospective single-center descriptive study including all AMI- and CLO-poisoned patients admitted in a University Hospital ICU in 2000–2016, evidenced by plasma AMI and CLO concentrations in the toxic ranges. Non-compartmental analysis of AMI and CLO pharmacokinetics was performed using WinNonlin v.5.1 software. Comparisons were performed using Chi-2 and Mann–Whitney tests as required.

**Results:** Sixty patients (age—49 years [42 + 61] (median [percentiles 25 + 75]) + 68% females 32% males) + 75% with depression) were included. AMI (dose—1.5 g [1.0 + 2.6] + plasma concentration—0.7 mg L^−1^ [0.4 + 1.4]) was responsible for 3 times more intoxications than CLO (dose—3.0 g [1.6 + 5.6] + plasma concentration—0.7 mg L^−1^ [0.5 + 1.4] with 72 vs. 28%). The patients presented consciousness impairment (Glasgow coma score—3 [3 + 9]), tonic–clonic seizures (12%), mydriasis (23%), QT lengthening (55%) and QRS enlargement (50%). AMI was responsible for a significantly deeper coma (p < 0.0001) but fewer seizures than CLO (p = 0.02). Three patients (5%) died. Based on a univariate analysis, factors associated with death were cardiac arrest onset (p = 0.003), elevated plasma lactate concentration (p = 0.005), low arterial pH (p = 0.007), reduced PaO2 FiO2 ratio (p = 0.007) and prothrombine ratio (p = 0.008), increased aspartate aminotransferases (p = 0.009), alanine aminotransferases (p = 0.01) and serum creatinine concentration (p = 0.01) as well as marked catecholamine infusion rate (p = 0.02). The pharmacokinetic study showed significant increase in AMI (43 h vs. 24 h) and CLO (55 h vs. 21 h) elimination half-lives in overdose compared to pharmacological conditions, highlighting the contribution of organ failure to the delayed elimination of both toxicants.

**Conclusion:** AMI and CLO poisonings did not disappear and are still responsible for significant morbidities and mortality. AMI was responsible for deeper coma with fewer seizures in comparison to CLO. AMI and CLO elimination half-lives were significantly prolonged in overdose due to organ failure.

### P-21 Investigation of the pharmacodynamic interaction involved in the respiratory depression attributed to diazepam buprenorphine combination

#### Vodovar Dominique^1^, Chevillard Lucie^1^, Pottier Géraldine^2^, Auvity Sylvain^2^, Caillé Fabien^2^, Risède Patricia^1^, Buvat Irène^2^, Tournier Nicolas^2^, Megarbane Bruno^4^

##### ^1^HU Henri Mondor, Paris, France; ^2^Université Paris-Sud, Orsay, France; ^4^Hôpital Lariboisière, Paris, France

###### **Correspondence:** Vodovar Dominique - dominique.vodovar@gmail.com

*Annals of Intensive Care* 2018, **8(Suppl 1):**P-21

**Introduction:** Severe poisonings and fatalities have been attributed to buprenorphine (BUP) despite its ceiling respiratory effects, mainly if abused in co-ingestion with benzodiazepines. We previously showed that diazepam (DZP) BUP combination induces severe respiratory depression in the rat, while each drug by itself does not. The objective of this study was to investigate the mechanisms involved in this drug–drug interaction using 11C-BUP PET imaging and diaphragmatic electromyography in the Sprague–Dawley rat.

**Patients and methods:** 11C-BUP was administered intravenously, 30 mg kg unlabeled BUP intraperitoneally and 20 mg kg DZP subcutaneously. PET acquisition started with 11C-BUP PET injection, 15 min after DZP or its vehicle (VEH + N = 5 group) administration. SUV normalized time activity curves (TACs) were generated and 11C-BUP binding potential [BPND, i.e. the ratio of the total receptor density (Bmax) on the equilibrium dissociation constant (KD)] were modeled in different brain regions using a simplified reference tissue model with cerebellum as reference region. dEM, implanted under anesthesia 7 days before the experiment, was recorded during 240 min in rats receiving VEH VEH, DZP VEH, VEH BUP or DZP BUP (N = 6 group). After filtering and half-wave rectification, the first 60 min AUC of diaphragm contraction and workload were determined and compared between the groups.

**Results:** TACs and 11C-BUP BPND were not different between the DZP BUP and the VEH BUP groups in all studied brain regions. Diaphragm contraction was significantly increased in the VEH BUP group in comparison to the DZP BUP group (p < 0.05). Diaphragm workload was significantly increased in the VEH BUP group in comparison to the DZP VEH and the DZP BUP group (p < 0.05 and p < 0.01 respectively).

**Discussion:** DZP did not affect the 11C-BUP brain distribution and brain binding suggesting that DZP does not affect BUP transport across the blood brain barrier and BUP receptors density affinity. BUP administration induced an increase in diaphragm contraction and workload. This increase was inhibited in the presence of DZP suggesting that DZP BUP combination-induced respiratory depression is mostly related to DZP.

**Conclusion:** Respiratory depression related to DZP BUP combination results from a pharmacodynamic drug–drug interaction.

### P-22 Is naloxone the best antidote to reverse tramadol-induced neuro-respiratory toxicity in overdose? An experimental investigation in the rat

#### Lagard Camille^1^, Malissin Isabelle^1^, Indja Wassila^1^, Risède Patricia^1^, Chevillard Lucie^1^, Megarbane Bruno^1^

##### ^1^Hôpital Lariboisière, Paris, France

###### **Correspondence:** Lagard Camille - camille.lagard@gmail.com

*Annals of Intensive Care* 2018, **8(Suppl 1):**P-22

**Introduction:** Since the banning of dextropropoxyphene from the market, overdoses and fatalities attributed to tramadol, a WHO step-2 opioid analgesic, have increased markedly. Tramadol overdose results not only in central nervous system (CNS) depression attributed to its opioid properties but also in seizures, possibly related to non-opioidergic pathways, thus questioning the efficiency of naloxone to reverse tramadol-induced CNS toxicity. Our objective was to investigate the most efficient antidote to reverse tramadol-induced seizures and respiratory depression in overdose.

**Patients and methods:** Sprague–Dawley rats overdosed with 75 mg kg intraperitoneal (IP) tramadol were randomized into four groups to receive solvent (control group), diazepam (1.77 mg kg IP), naloxone (2 mg kg intravenous bolus followed by 4 mg kg h infusion) and diazepam naloxone combination. Sedation depth, temperature, number of seizures and intensity, whole-body plethysmography parameters and electroencephalography activity were measured. For each parameter, we compared the areas under the curves using Mann–Whitney tests for two-by-two comparisons between the four groups. Regarding the effects of treatments on seizures, comparisons were performed using two-way analysis of variance followed by multiple comparison tests using Bonferroni’s correction.

**Results:** Naloxone reversed tramadol-induced respiratory depression (p < 0.05) but significantly increased seizures (p < 0.01) and prolonged their occurrence time. Diazepam abolished seizures but significantly deepened rat sedation (p < 0.05) without improving ventilation. Diazepam naloxone combination completely abolished seizures, significantly improved rat ventilation by reducing inspiratory time (p < 0.05) but did not worsen sedation. Based on the EEG study, tramadol-treated rats experienced electro-clinical seizures as soon as 5 min after the injection, characterized by spike-waves and polyspikes with progressive decreased frequencies and inter-critical phases of slow delta waves until the next crisis. After diazepam naloxone injection, EEG waveforms consisted in 8 Hz-alpha rhythms and slow-down theta rhythms of drowsiness. None of these treatments significantly modified rat temperature.

**Conclusion:** Diazepam naloxone combination is the most efficient antidote to reverse tramadol-induced CNS toxicity. Our experimental data greatly encourage administering this combination rather than naloxone alone as first-line antidote in tramadol-poisoned patients as an alternative to tracheal intubation.

### P-23 Acute Rubigine^®^ poisoning in the French Overseas Departments of America: clinical characteristics and prognostic factors

#### Resiere Dabor^1^, Florentin Jonathan^1^, Valentino Ruddy^1^, Brouste Yanick^1^, Gueye Papa^1^, Mehdaoui Hossein^1^

##### ^1^CHU de la Martinique, Fort-De-France, France

###### **Correspondence:** Resiere Dabor - dabor.resiere@chu-martinique.fr

*Annals of Intensive Care* 2018, **8(Suppl 1):**P-23

**Introduction:** Rubigine^®^ poisoning is a medical emergency that causes a major public health problem in underdeveloped countries, as it is frequently fatal. This poisoning is rare in France, but frequent in the French overseas departments (DOM). The Rubigine^®^, made of fluoride and used as a rust remover, is the main source of poisoning in the Caribbean. In Martinique, the exact incidence of this intoxication is unknown, as there is no national and regional register. It could represent up to 8–10% of severe acute poisoning. It was not until April 1994 that, following a prefectural order on the declaration, classification, packaging and labeling of substances, the composition of Rubigine^®^ was modified to significantly reduce the mortality induced by its ingestion. The objective of our study was to describe the clinical features and complications that can occur after ingestion of Rubigine^®^ as well as to determine the prognostic factors of death.

**Patients and methods:** We conducted a retrospective study over 16 years, from 2000 to 2016, including all patients admitted to emergency and intensive care units of the University Hospital Center (Martinique) for acute Rubigine^®^ poisoning. The usual demographic and clinical data were collected and comparisons between surviving and deceased patients were performed using a univariate analysis.

**Results:** Fifty-five patients (mean age—43 years (15–82) + sex ratio male female—1,1) were hospitalized at the University Hospital of Martinique. One-quarter of patients had no significant history. The average length of stay was 3.3 days (1–37). Forty percent of patients experienced hypocalcaemia after initial intravenous calcium supplementation. Complications included acute respiratory failure requiring invasive mechanical ventilation (22% of patients, duration of ventilation—2.8 days, (1–16)), renal failure (20%, of which 50% required extra-renal treatment, hemodynamic failure (16%), hepatic failure (15%), coagulation failure (15%), neurological failure (12%) and multi-visceral failure (10.9%). Three patients presented cardiogenic refractory shock requiring VA ECMO (5.5%) and another patient with digestive perforation (1.8%). The mortality was 10.9%, allowing the identification of prognostic factors of death.

**Conclusion:** Rubigine^®^ poisoning is responsible for significant morbidity and mortality, despite optimal management. However, its incidence seems to have decreased sharply in recent years thanks to the strong mobilization and awareness of the population following the implementation of an information system by the University Hospital ‘s clinical toxicology and toxico-vigilance unit, and different preventive measures introduced by the health authorities.

### P-24 Acute insulin overdose with suicidal attempt: management and complications

#### Khzouri Takoua^1^, M’Rad Aymen^1^, Foudhaili Nasreddine^1^, Mahdhaoui Soumaya^1^, Bachrouch Maissa^1^, Fatnassi Meriem^1^, Barghouth Manel^1^, Brahmi Nozha^1^

##### ^1^Centre Mahmou Yaacoub d’assistance médicale urgente, Tunis, Tunisia

###### **Correspondence:** Khzouri Takoua - takoua_kh2@yahoo.fr

*Annals of Intensive Care* 2018, **8(Suppl 1):**P-24

**Introduction:** Acute insulin overdose with suicidal attempt (AIOSA) is rare and understudied. The aim of this work was to provide epidemiolgical, cilnical, biological, therapeutic and pronostic features of this condition.

**Patients and methods:** We conducted a retrospective study of patients admitted for AIOSA with hypoglycemia in our intensive care unit during the period between January 2012 and December 2016.

**Results:** Sixty-two patients were included, they were 24 males and 38 females aged of 32 ± 13 years. Fifty percent of patients were diabetics, 30% were long term treated by insulin and 19% had a history of AIOSI. Intermediate-acting insulin (IAI) was found in 54% (n = 33), rapid-acting insulin (RAI) in 28% (n = 17), insulin glargine in 10% and mixed intoxications in 8%. Route of administration was subcutaneous in all cases. Median capillary glycaemia on the first medical contact was 44 mg dl [36 + 71]. The most common signs and symptoms were sweats (33%), dizziness (19%), palpitations (16%), coma (21%), paresthesia (12%) and nausea (12%). The most common electrolyte abnormality was hypokalemia (29%). The amount of insulin was similar in the group IAI (500 UI) and RAI (555 UI). However, there were a difference in term of delay of symptom onset which was of was of 198 ± 98 min in the IAI group versus 80 ± 40 min in the RAI group (p < 0.001), hypoglycemia duration—24 ± 11 h in the IAI group versus 20 ± h in the RAI group (p = 0.447) and finally the mean required dose of carbohydrates—254 ± 140 g in IAI group versus 250 ± 95 g in RAI group (p = 0.908). Outcome was favorable in 95% of cases (n = 49) who discharged hospital in 47 ± 13 h. Two patients kept neurological sequelae because of late management and one patient died.

**Conclusion:** Insulin intoxication is rare with some unusual presentations. Basis of treatment are carbohydrates administration and blood glucose monitoring. It is a potentially serious condition with low mortality and a risk of electrolyte disorders and severe neurological sequelae. Early care is the main prognosis factor.

### P-25 Delayed neuropsychiatric sequelae observed in outpatients victims of carbon monoxide poisoning

#### Khzouri Takoua^1^, Foudhaili Nasreddine^1^, Mahdhaoui Soumaya^1^, Bachrouch Maissa^1^, Ben Hamida Samia^1^, Fatnassi Meriem^1^, M’Rad Aymen^1^, Brahmi Nozha^1^, Essafi Fatma^1^, Chatbri Bassem^1^, Mhamdi Ammar^1^, BahriaWided^1^, Thabet Hafedh^1^

##### ^1^Centre Mahmou Yaacoub d’assistance médicale urgente, Tunis, Tunisie

###### **Correspondence:** Khzouri Takoua - takoua_kh2@yahoo.fr

*Annals of Intensive Care* 2018, **8(Suppl 1):**P-25

**Introduction:** Carbon monoxide (CO) poisoning can lead to late neurological sequelae. The prevalence of these sequelae is rarely reported. The present study aims to determine the prevalence of neuropsychiatric sequelae after CO poisoning in outpatients, to analyse their characteristics and to identify the main associated risk factors.

**Patients and methods:** Carbon monoxide (CO) poisoning can lead to late neurological sequelae. The prevalence of these sequelae is rarely reported. The present study aims to determine the prevalence of neuropsychiatric sequelae after CO poisoning in outpatients, to analyse their characteristics and to identify the main associated risk factors.

**Results:** Eighty-three patients were included. They were 17 males and 66 females aged of 33 ± 12 years [6–69]. They were healthy in 80% of cases. The mean serum HbCO level on admission was 15 ± 8% (5–40%). Hyperbaric oxygen therapy was required in 12.5% of patients (n = 10). The mean telephone contact delay was 16 ± 9 months (4–36). The prevalence of affected patient was of 26%. The mean onset time of symptoms was 5 ± 3 months (1–10). The main identified disorders were persistent headaches (39%), anxiety-depressive disorders (39%), irritability (22%) and cognitive disorders (22%). No identified risk factors for developing delayed neuropsychiatric sequelae following CO poisoning were found in this study.

**Conclusion:** Despite the high prevalence of neurological sequelae after CO poisoning, no identified risk factors has been found, particularly hyperbaric oxygen therapy. It is very important to inform patients about the risk of late sequelae and to prompt them to consult as soon as the first signs appear.

### P-26 Mechanisms of tramadol-induced seizures in overdose in the rat

#### Vodovar Dominique^1^, Lagard Camille^1^, Chevillard Lucie^1^, Jacques Callebert^1^, Liang Hao^1^, Risède Patricia^1^, Tournier Nicolas^1^, Megarbane Bruno^1^

##### ^1^Université Paris-Diderot, Paris, France

###### **Correspondence:** Vodovar Dominique - dominique.vodovar@gmail.com

*Annals of Intensive Care* 2018, **8(Suppl 1):**P-26

**Introduction:** Since dextropropoxyphene withdrawal from the market, overdoses and fatalities attributed to tramadol, a WHO step-2 opioid analgesic drug, have increased markedly. Besides central nervous system depression, tramadol overdose may result in seizures, usually included in the related serotonin syndrome. However, the serotoninergic mechanism of tramadol-induced seizures has been recently questioned. We investigated the effects of various specific pretreatments on tramadol-induced seizure onset and alterations in brain monoamines in the rat.

**Patients and methods:** Sprague–Dawley rats were randomized into five groups (N = 6 group) to be pretreated with various agonists antagonists before receiving 75 mg kg tramadol intraperitoneally—1.77 mg kg IP diazepam + 2 mg kg IV bolus followed by 4 mg kg h infusion naloxone + 10 mg kg IP cyproheptadine, and 15 mg kg IP fexofenadine. Seizure severity was graded according to the modified Racine Score (1). We measured neurotransmitter concentrations in the frontal cortex using high performance liquid chromatography coupled to flurorimetry or radioenzymatic assay, as required. We used positron emission tomography-computed tomography to investigate Interactions of tramadol with GABA-A receptors. The effects of treatments on seizures were compared using two-way analysis of variance followed by multiple comparison tests with Bonferroni’s correction. The Areas under the curves of the effects on monoamine concentrations and the binding potentials in the PET-imaging study were compared two-by-two using Mann–Whitney *U* tests.

**Results:** Diazepam abolished tramadol-induced seizures, by contrast to naloxone, cyproheptadine and fexofenadine pretreatments. Interestingly, despite seizure abolishment, diazepam significantly enhanced tramadol-induced increase in the brain serotonin (p < 0.01), histamine (p < 0.01), dopamine (p < 0.05) and norepinephrine (p < 0.05) while no significant modifications were observed with the other tested pretreatments. Based on Positron emission tomography imaging using 11C-flumazenil fixation in the rat brain, we demonstrated molecular interaction between tramadol and γ-aminobutyric acid (GABA)-A receptors not related to a competitive mechanism between tramadol and flumazenil on the benzodiazepine binding site. Our findings clearly ruled out the involvement of serotoninergic, opioidergic, histaminergic, dopaminergic and norepinephrinergic pathways in tramadol-induced seizures while strongly suggested tramadol-induced specific allosteric change in GABAA receptors that could contribute to seizures onset in overdose.

**Reference**

1. Racine RJ. Modification of seizure activity by electrical stimulation. Electroencephalogr Clin Neurophysiol 1972 + 32–269–299.

**Conclusion:** Tramadol-induced seizures in overdose are mainly related to the GABAergic pathway.

### P-27 Clinical and biological aspects of Heparin-induced thrombocytopenia in intensive care unit

#### Sellami Walid^1^, Dhaouadi Imen^2^, Hajjej Zied^2^, Ben Mrad Ines^2^, Yengui Olfa^2^, Sammoud Walid^2^, Bousselmi Radhouene^2^, Gharssallah Hedi^2^, Labbene Iheb^2^, Ferjani Mustapha^2^

##### ^1^Montefleury, Tunis, Tunisia; ^2^Hôpital Militaire, Tunis, Tunisia

###### **Correspondence:** Sellami Walid - drsellamiwalid@yahoo.fr

*Annals of Intensive Care* 2018, **8(Suppl 1):**P-27

**Introduction:** Heparin-Induced Thrombocytopenia (HIT) is a serious iatrogenic complication of heparinic treatments. The diagnosis of HIT is difficult in the resuscitation environment because thrombocytopenia is a frequent and multifactorial phenomenon. The aim of this work was to study the clinical and biological presentation of patients with HIT and the consequences attributable to HIT on the evolution of patients in terms of morbidity and mortality and to develop a diagnostic strategy for HIT for resuscitation patients.

**Patients and methods:** This was a retrospective, monocentric, descriptive and evaluative study conducted in our intensive care unit (ICU) over a period of 4 years 6 months. An anti-PF4 antibody test was performed in patients who developed thrombocytopenia or a 30% drop in their initial platelet kinetics and the clinical picture.

**Results:** The incidence of HIT was 1.71% in patients hospitalized in ICU. The clinicobiological severity scores, the reasons for admission to resuscitation were similar in both groups (HIT+ and HIT−) as well as the characteristics of the heparins used. The time of occurrence of thrombocytopenia was similar in the two groups. The diagnosis of HIT was more often the only plausible diagnosis in the HIT+ group. The 4T’s score was significantly higher in the HIT+ group. The evolution of the platelet count was similar in the two groups, in the decay phases as well as in the recuperation phase. HIT+ patients showed significantly more thrombosis than HIT− patients. There was no significant difference between the transfusion needs of HIT+ and HIT− patients. Mortality was identical in both groups, as was the length of stay in ICU.

**Conclusion:** HIT is a rare disease. There was no evidence of a predisposing factor for the occurrence of the disease in a uniform resuscitation population. The diagnosis of HIT is based on a cluster of arguments and not on an isolated event. Biological tools are indispensable, in a complementary way to the clinical picture.

### P-28 Pulmonary embolism in patients with sickle cell disease in intensive care unit: a challenging diagnosis

#### Jamoussi Amira^1^, Zayet Souheil^1^, Merhebene Takoua^1^, Ayed Samia^1^, Ben Khelil Jalila^1^, Besbes Mohamed^1^

##### ^1^Hôpital Abderrahmen Mami, Ariana, Tunisia

###### **Correspondence:** Jamoussi Amira - dr.amira.jamoussi@gmail.com

*Annals of Intensive Care* 2018, **8(Suppl 1):**P-28

**Introduction:** Pulmonary embolism (PE)’s occurence within sickle cell disease (SCD) is already reported and may be due to intrinsic hypercoagulability. However, PE’s diagnosis can be difficult because of clinical similarities with acute chest syndrome. In addition, SCD is a systemic disease that could be associated to chronic or acute pulmonary hypertension with unknown pathophysiology. Aim—To describe the clinical manifestations, diagnosis, ultrasound parameters, and management of PE in patients with SCD in intensive care unit.

**Patients and methods:** We conducted a retrospective and descriptive study, including SCD patients hospitalized in medical ICU of Abderrahmen Mami Hospital, diagnosed with PE during 11 years and 3 months period from January 2006 to March 2017.

**Results:** During the study period, a total of 26 patients with SCD were admitted. Among them, 20 presented with respiratory distress and chest pain and then benefited first of trans-thoracic echocardiography that often showed right ventricle dilation and systolic PAP > 30 mmHg (n = 11). All the 20 patients underwent enhanced CT-scan and the diagnosis of PE was finally retained in 6 cases (18.8%) and hence colliged.

The average age was 32.5 years ± 9.4 [20–42 years] with a sex-ratio = 1. The mean of APACH II score was 7. SCD were diagnosed at the age of 8.2 years ± 6.2 [3–15 years] with a regular follow up in 83%. The reason for admission was acute respiratory failure in all cases. Patients had clinical symptoms of pneumonia: pleuritic chest pain (n = 6), dyspnea (n = 5) and fever (n = 2). All patients had a chest X-ray showing an alveolo-interstitial syndrome in 4 cases (66.6%) and an associated pneumonia in 2 cases (3.33%). At enhanced CT-scan, PE was unilateral in all cases with an average Kanadly obstruction index of 27.9% [12.5–42%]. Echocardiographic findings are illustrated in Table 1. PE’s mortality risk was low or intermediate low so there was no need for thrombolysis in all cases. Heparin at curative dose was prescribed in all cases with a relay by oral anticoagulant. Mean ICU stay was 6 days [3–11 days]. A favorable outcome was achieved in all cases.

**Table 1:** Ultrasound parameters in transthoracic echocardiography in studied patients (n = 6/6)

Echocardiography parameters	Values
FEVG% median [extremes]	48.16 [44–45]
ITVsAo mean ± SD [extremes]	21.26 ± 1.76 [19.4–22.9]
SVD/SVG mean ± SD [extremes]	0.53 ± 0.42 [0.5–0.56]
PAPS mmHg median [extremes]	33.75 [30–45]
TAPSE mean ± SD [extremes]	2.33 ± 0.41 [2–2.8]
STDI mean ± SD [extremes]	21.86 ± 1.91 [20.3–24]

**Conclusion:** PE occurrence is considerable within SCD. PE’s diagnosis is challenging in ICU among SCD patients presenting clinical and echocardiographic signs that could mimic acute chest syndrome. Enhanced CT-scan is often necessary to confirm or to exclude PE.

### P-29 Acute pulmonary hypertension during acute chest syndrome in adult patients with sickle cell disease: Is there a hypercoagulability state?

#### Voiriot Guillaume^1^, Jutant Etienne-Marie^1^, Gerotziafas Grigorios^1^, Labbe Vincent^1^, Mokrani Hayat^1^, Fartoukh Muriel^1^

##### ^1^Hôpital Tenon, Paris, France

###### **Correspondence:** Voiriot Guillaume - guillaume.voiriot@aphp.fr

*Annals of Intensive Care* 2018, **8(Suppl 1):**P-29

**Introduction:** Acute chest syndrome (ACS) is the most severe complication of sickle cell disease and its evolution is unpredictable. Acute pulmonary hypertension (PH) in ACS is associated with an increased mortality, but its mechanism remains poorly known. Our hypothesis is that acute PH is associated with a biological state of hypercoagulability in ACS.

**Patients and methods:** In a prospective single center study, all consecutive SCD patients with ACS admitted to the intensive care unit (ICU) of Tenon hospital were included. Specialized haemostasis dosages were performed on ICU admission. A trans-thoracic echocardiogram was also performed on admission, and was repeated at steady state.

**Results:** Among 29 patients with ACS, 22 had a trans-thoracic echocardiogram and 6 had a high echocardiographic probability of acute PH, including 1 patient with bilateral pulmonary embolism and 1 patient who developed multiple organ failure and died. There were no significant clinical, biological or radiological differences between patients with a low-intermediate probability of acute PH and those with a high probability of acute PH+ their evolution was similar. The exploration of haemostasis did not show between-group differences, regarding each parameter of haemostasis. However, when using a hierarchical cluster analysis, distinct profiles of coagulation were evidenced, defining 3 biological classes. The subset of patients with a high echocardiographic probability of acute PH was more frequent in biological classes 2 and 3 which corresponded to hypercoagulability states. Acute PH was transient in patients (n = 17) with a repeated echocardiography at steady state.

**Conclusion:** Acute PH may likely occur in patients with ACS and a biological condition of hypercoagulability. Further studies are needed to confirm these findings.

### P-30 Prognostic role of modified Glasgow prognostic score in lung cancer patients admitted in intensive care

#### Gorham Julie^1^, Ameye Lieveke^1^, Paesmans Marianne^1^, Berghmans Thierry^1^, Sculier Jean-Paul^1^, Meert Anne-Pascale^1^

##### Institut Jules Bordet, Bruxelles, Belgium

###### **Correspondence:** Gorham Julie - julie.gorham@hotmail.com

*Annals of Intensive Care* 2018, **8(Suppl 1):**P-30

**Introduction:** The prognostic and predictive roles of the modified Glasgow Prognostic Score (mGPS), based on serum C-reactive protein and albumin levels, have been shown in patients with operable and inoperable cancers. Its value for hospitalization and death during hospitalization in a population of patients with lung cancer (LC) consulting at the emergency department has also been demonstrated previously. The aim of our retrospective study was to evaluate the prognostic role of mGPS in LC patients admitted to intensive care unit (ICU).

**Patients and methods:** We performed a retrospective study in a cancer hospital between September 1st 2008 and December 31st 2013. We included all patients with LC admitted into the ICU for a medical or surgical complication and for whom the data (CRP and albumin levels) used to calculate the mGPS were available.

**Results:** CRP and albumin values were available to calculate the mGPS in 148 patients with LC admitted in the ICU. The majority of patients were younger than 70 years (72.3%), were male (63.5%) and had non-small cell lung cancer (NSCLC) (79.1%). Cardiovascular (33.1%), respiratory (26.4%) and neurological complications (17.6%) were the three main causes of admissions. Hospital mortality was 26.4%. SAPS 2 score (OR 1.07 + 95% CI 1.03–1.11 + p < 0.001) and respiratory distress (OR 3.46 + 95% CI 1.41–8.51 + p 0.007) were the 2 independent predictors of hospital mortality. Presence of metastases (HR 2.25 + 95% CI 1.37–3.69 + p = 0.001) and mGPS (HR 1.30 + 95% CI 1.01–1.68 + p = 0.04) were the two independent predictors of survival after hospital discharge.

**Conclusion:** In lung cancer patients admitted into the ICU, the mGPS is an independent predictor of survival after hospital discharge but not for mortality during ICU stay. This inflammatory score could therefore be used as a long-term prognostic marker in this population of patients and would be more reflective of cancer, than reflecting the acute complication leading to ICU admission. Prospective and multicentric studies must be carried out to validate these results.

### P-31 Interest of FVIIa factor in postpartum haemorrhage: about 14 cases

#### Ferjani Wassim^1^, Ben Jdidia Bilel^1^, Saoudi Fedi^2^, Haddad Hayfa^2^, Azri Ines^2^, Romdhane Hayfa^2^, Zaidi Bacem^2^, Barhoumi Mohamed Hafed^3^

##### ^1^CHU Habib Bourhuiba sfax, Sfax, Tunisie; ^2^Monastir, Tunisie; ^3^Kairouan, Tunisie

###### **Correspondence:** Ferjani Wassim - wassimferjani01@gmail.com

*Annals of Intensive Care* 2018, **8(Suppl 1):**P-31

**Introduction:** Recombinant active factor VII is a pro-hemostatic treatment used in obstetric haemorrhage, but no study has made it possible to specify its exact place in the decision algorithm. The objective of our work is to evaluate the efficacy and the benefit risk ratio of recombinant factor VIIa in the treatment of severe postpartum hemorrhage.

**Patients and methods:** We conducted a prospective study at the IBN JAZZAR University Hospital in Kairouan during the period from January 1, 2015to December 31, 2016. In total, we collected 15 cases of recombinant factor VIIa in one postpartum haemorrhage.

**Results:** The mean age of our patients was 31 + 5.96 years. The rate of childbirth was 33.33%. The caesarean was the mode of delivery chosen for 10 patients. The causes of postpartum haemorrhage in our series were—uterine atony in 5 cases, uterine rupture and cervico-uterine tear 3 cases each, retroplacental hematoma and placenta accreta 1 cases for each two and placenta prævia in 2 cases. Our patients were treated in an intensive care unit and the average hospital stay was 4.1 days. Sulprostone was reported in 7 cases (46.6%), and all patients received a massive transfusion. The average time to administer rFVIIa was 6 h 40 min. The mean dose of factor VIla recombinant was 78.8 ± 9.24 μg kg. Five patients received a single dose, 8 patients received a second injection and 2 patients received 3 doses. Clinical efficacy—After a single injection, clinical efficacy with reduction in bleeding was observed in 8 patients, i.e. 60%. The most frequent complication was insufficiencyrenal in 7 cases including 2 requiring hemodialysis, CIVD in 4 cases, OAP in 2 cases, a multivisceral failure in 2 cases, a septic shock in 1 case and a mesenteric infarction in 1 case. The progression was favorable in 12 patients, while 3 patients died (21.9%).

**Table:** 

	Before	After	% of reduction
Hemoglobin	7.64	8.54	
Hematocrit	21.9		
Prothrombin	68	76.2	
Platelets	117,000	136,000	
Fibrinogen	1.11	2.78	
CG	6.98	3.84	44.98
CP	3.40	1.68	50.5
PFC	9.58	6.14	35.9

**Conclusion:** It is important that new studies be carried out and shared experiences around the world on this drug appear to be effective and prevent invasive actions in the therapeutic arsenal of postpartum heamorrhage.

### P-32 Early administration of tranexamic acid and fibrinogen concentrate reduces mortality in postpartum hemorrhage

#### Khedher Mouna^1^, Ferhi Fehmi^1^, Gardabou Omar Kais^1^, Nasr Amal^1^, Chrigui Noura^1^, Bouslema Med Amine^1^, Tarmiz Khalil^1^, Ben Jazia Khaled^1^

##### ^1^Research unit on maternal morbidity and mortality UR17SP08, Sousse, Tunisia

###### **Correspondence:** Khedher Mouna - mounakhedher85@gmail.com

*Annals of Intensive Care* 2018, **8(Suppl 1):**P-32

**Introduction:** Post-partum haemorrhage (PPH) is a life-threatening complication and remains a leading cause of maternal morbidity worldwide. The WOMAN trial* estabished that early administration of Tranexamic Acid (TA) reduces mortality due to the bleeding in women with PPH. Our study purpose was to determine the effects of early administration of TA and fibrinogen concentrate on death, hysterectomy and transfusion in women with severe PPH.

**Patients and methods:** This retrospective, monocentric study was performed in a third level Tunisian hospital providing healthcare for more than 10000 pregnant women per year. Were included in this study women with diagnosis of severe post partum haemorrhage after a vaginal or caesarean delivery from 2014 to 2016. Patients who received TA and fibrinogen concentrate were assessed in group 1(G1) and who not in group 2(G2).

**Results:** The incidence of severe PPH was 35/10000 deliveries. 166 women were retained for data analysis G1 (n = 109), G2 (n = 57). Anthropomorphic and obstetrics characteristics were not significantly different between the two groups. There was a significant difference between the two groups regarding to transfused units of red blood cells however, no difference in term of the use of frozen plasma and platelets concentrates was observed. Perioperative hemoglobin nadir was significantly higher in G 1. The frequency of hysterectomy and pelvic packing were higher in G2 (Table 1). No thromboembolic events and no haemorrhage related mortality were observed in the two groups.

**Table 1:** Anthropomorphic and obstetrics characteristics

	G1 (n = 109)	G2 (n = 57)	p value
PRBC units mean (extremes)	4.31 (2–15)	5.77 (2–13)	0.003
Platelets concentrates (n)	8.65 (0–20)	10.24 (0–24)	0.19
Frozen plasma concentrates (n)	8.04(0–30)	8.95 (0–26)	0.323
Hb nadir (g/dl)	7.31 ± 2.09	6.23 ± 1.56	0.003
Hysterectomy n(%)	23 (21.1)	28 (49.1)	0.003

**Conclusion:** In this retrospective study, early administration of tranexamic acid and fibrinogen reduces risk of hysterectomy transfusion. These encouraging results strongly support the need for a large, international, double-blind study to investigate the potential of the association “TA—Fibrinogen concentrate” to reduce maternal haemorrhage related morbidity and mortality.

### P-33 Outcome of hematologic patients with respiratory failure in a pediatric intensive care unit

#### Vanel Noemie^1^, Ughetto Fabrice^1^, Michel Fabrice^1^, Barlogis Vincent^1^, Picon Julie^1^

##### ^1^Hôpital de La Timone Marseille, Marseille, France

###### **Correspondence:** Vanel Noemie - Noemie.VANEL@ap-hm.fr

*Annals of Intensive Care* 2018, **8(Suppl 1):**P-33

**Introduction:** Immunodeficiency, acquired or congenital, is the first comorbidity associated with poor outcome in pediatric patients with acute respiratory distress syndrome 1(ARDS). The aim of this study was to describe outcome of pediatric patient with hematologic disease hospitalized in our intensive care unit for respiratory failure and to investigate the clinical variables associated with mortality.

**Patients and methods:** It was a retrospective monocentric descriptive study including all immunodeficient pediatric patient (malignant hemopathy, congenital immunodeficiency, bone marrow transplantation…) from hematology hospitalized in our 14 beds pediatric intensive care unit with the diagnosis of respiratory failure between January 2003 and February 2017.

**Results:** Fifty one patients were included corresponding to 53 admissions. Nighty percent of the patients met criteria for pediatric ARDS—67% were severe, 15% moderate and 2% mild. Extracorporeal circulation (ECC) was needed for 7 patients. Global mortality rate at PICU discharge was 47%. Twenty four patients (45%) received noninvasive ventilation (NIV). Height of them (33%) did not need invasive mechanical ventilation (IMV). In patients who received IMV, mortality rate was significantly higher if patients received before NIV (81 vs. 41%) p = 0.001. All patients who needed IMV after more than 24 h of NIV died (n = 3). Mortality was higher in children with griffon versus host disease (80 vs. 40% p = 0.04). Mortality of patients receiving ECC and renal replacement therapy (RTT) was respectively 71 and 80%.

**Conclusion:** In our study, most of the patients hospitalized for respiratory failure met criteria for pediatric ARDS. If NIV decrease IMV requirement, it could be associated with higher mortality rate in case of failure. This result support recent recommendation2 that immunodeficiency is not a sufficient criteria to delayed IMV.

### P-34 Influence of different non-invasive respiratory support devices on tidal volume (Vt): a bench study

#### Maraffi Tommaso^1^, Haudebourg Anne-Fleur^1^, Razazi Keyvan^1^, De Prost Nicolas^1^, Mekontso Dessap Armand^1^, Carteaux Guillaume^1^

##### ^1^Hôpital Henri Mondor, Créteil, France

###### **Correspondence:** Maraffi Tommaso - tommaso.maraffi@aphp.fr

*Annals of Intensive Care* 2018, **8(Suppl 1):**P-34

**Introduction:** Multiple devices are available for non-invasive respiratory support—mask noninvasive ventilation (M-NIV), helmet NIV (H-NIV), high flow nasal cannula (HFNC) and continuous positive airway pressure (CPAP). There are conflicting data about their respective effects during acute respiratory failure. Vt is a key determinant of both efficacy and safety during ventilatory support. We assess whether Vt is influenced by noninvasive ventilatory device.

**Patients and methods:** Six setups were tested using the RespiSim^®^ Manikin connected to an ASL 5000^®^ breathing simulator—no device + oxygen mask at 15 L min flow + HFNC at 60 L min + CPAP at 10 cm H_2_O+ M-NIV and H-NIV both with a PEEP of 5 cm H_2_O and a pressure support of 10 cm H_2_O. Three different respiratory mechanics were simulated—normal (compliance—60 ml cm H_2_O, resistance—5 cm H_2_O L sec), obstructive (compliance—60 ml cm H_2_O, resistance—20 cm H_2_O L sec) and restrictive (compliance—30 ml cm H_2_O and resistance—5 cm H_2_O L sec) + each mechanics was tested at three simulated efforts—low (inspiratory muscle pressure (Pmus)—5 cm H_2_O), moderate (inspiratory Pmus—10 cm H_2_O), and respiratory distress (inspiratory Pmus—20 cm H_2_O, expiratory Pmus—10 cm H_2_O). Flow and airway pressure were recorded at the ASL inlet and mouth pressure into the manikin mouth. We defined “device driving pressure” as the peak mouth pressure minus the tele-expiratory mouth pressure. Continuous data are reported as mean ± SD.

**Results:** As compared to the oxygen mask, Vt increased significantly with M-NIV and H-NIV whatever the simulated respiratory effort (599 ± 201 and 537 ± 217 vs. 307 ± 121 ml respectively with the moderate simulated effort, p < 0.001; Fig. 1). HFNC and CPAP were associated with a slight but non-significant decrease in Vt as compared to the oxygen mask. Overall, for a given respiratory effort, Vt was influenced by the “device driving pressure”, which tended to decrease when using HFNC and CPAP and markedly increased with M-NIV as compared to the oxygen mask. Therefore, Vt in M-NIV with a simulated low effort was significantly higher than Vt in CPAP and HFNC with a simulated moderate effort (456 ± 162 ml, 252 ± 95 ml, and 262 ± 90 ml respectively, p = 0.01 for both comparisons).

**Fig. 1:**
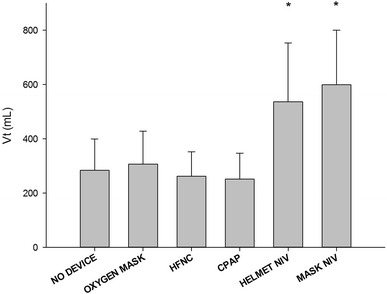
Tidal volume (Vt) per non-invasive respiratory support device

**Conclusion:** In our bench model, the Vt value was significantly influenced by the noninvasive ventilatory device. NIV was invariably associated with significantly higher Vt than with other devices, even when dividing by two the simulated inspiratory effort during NIV.

### P-35 Primary outcome for studies on intubation in intensive care unit-performance of different metrics: an ancillary study of the MACMAN trial

#### Lascarrou Jean-Baptiste^1^, Bailly Arthur^2^, Le Thuaut Aurelie^3^, Champigneulle Benoit^4^, Toufik Kamel^5^, Mercier Emmanuelle^6^, Ricard Jean-Damien^7^, Lemiale Virginie^8^, Colin Gwenhael^2^, Martin Maelle^3^, Helms Julie^9^, Reignier Jean^3^

##### ^1^CHU de Nantes, France; ^2^CHD Vendee, France; ^3^CHU Nantes, France; ^4^HEGP, France; ^5^CHR Orleans, France; ^6^CHU Tours, France; ^7^Hopital Louis Mourier, France; ^8^CHU Saint Louis, France; ^9^CHU Strasbourg, France

###### **Correspondence:** Lascarrou Jean-Baptiste - jeanbaptiste.lascarrou@chu-nantes.fr

*Annals of Intensive Care* 2018, **8(Suppl 1):**P-35

**Introduction:** In ICU, intubation is a high risk procedure associated with high morbidity. Despite procedure’s improvement with systematic application of fluid loading, early use of vasopressors and checklist use, morbidity remains high. Several recent trials has been conducted with different metrics choose as primary outcome. However any evidence exists to choose one more than another: time to intubation, first pass success, difficult intubation. First pass success sine hypoxia and hypotension (DASH-1A) has been highlighted recently and choose by the GAME program without any scientific evaluation. We conducted a post hoc analysis of the randomized clinical trial MacGrath Mac video laryngoscope or Macintosh laryngoscope for intubation in the intensive care unit (MACMAN) to determine the best metric to choose for primary outcome for the next intubation studies in ICU.

**Patients and methods:** MACMAN was a multicentre, open-label, randomized controlled superiority trial. Consecutive patients requiring intubation were randomly allocated to either the McGRATH MAC videolaryngoscope or the Macintosh laryngoscope, with stratification by centre and operator experience. An only inclusion criterion was—“Patients must be admitted to an ICU and require mechanical ventilation through an endotracheal tube”. Patients were excluded if—contraindication to orotracheal intubation (e.g., unstable spinal lesion) + insufficient time to include and randomize the patient (e.g., because of cardiac arrest) + age < 18 years + pregnant or breastfeeding woman + correctional facility inmate + patient under guardianship + patient without health insurance + refusal of the patient or next of kin to participate in the study + previous enrolment in a clinical randomized trial with intubation as the primary end point (including previous inclusion in the present trial). Post-hoc analysis was performed to assess association and prediction of life threatening complication (mild to moderate, severe, mild to severe) by different metric existing—time to intubation, first pass success, difficult intubation, first pass success sine hypoxia and hypotension. Each metric was compared with another one. Area under curve was built for every metric and all metrics were then compared.

**Results:** DASH-1A was superior to all others metrics included in the analysis for prediction of life threatning complications (all p < 0.001).

**Table:** 

	OR [IC95%]	AUC	Se	Sp
Failure of first pass	1.18 [0.43–3.22] p = 0.75	0.52 [0.40–0.63]	33.3 [13.3–59.0]	70.2 [64.9–75.1]
Bougie use at first pass	1.41 [0.31–6.47] p = 0.66	0.51 [0.44–0.59]	11.1 [1.4–34.7]	91.9 [88.4–94.6]
Duration of intubation > 3 min	1.01 [0.37–2.76] p = 0.99	0.50 [0.39–0.62]	33.3 [13.3–59.0]	66.9 [61.5–71.9]
DASH1-A	32.51 [4.27–247.39] p = 0.001	0.80 [0.74–0.86]	94.4 [72.7–99.9]	65.7 [60.3–70.8]

**Conclusion:** All metrics are not equal to predict severe life threatening complications during intubation in the ICU. In this context, we recommend adoption of definitive airway sine hypoxia or hypotension at first attempt (DASH-1A) as primary outcome for intubation studies in the ICU or as metric indicator tracked in quality improvement program.

### P-36 The place of non invasive ventilation (NIV) in the management of acute respiratory failure in the emergencies of Oran University Hospital Center

#### Benbernou Soumia^1^, Ghomari Nabil^1^, Djebli Houria^1^, Azza Abdelkader^1^, Iles Malik Sofiane^1^, Bouyacoub Khalida^1^

##### ^1^Laboratoire de recherche AVC, Oran, Algeria

###### **Correspondence:** Benbernou Soumia - gsoumia@hotmail.com

*Annals of Intensive Care* 2018, **8(Suppl 1):**P-36

**Introduction:** IntroductionAcute respiratory failure (ARF) is a common cause of emergency use and one of the major reasons for admission to intensive care unit. It associates a vital risk imposing immediate symptomatic treatments and an etiological approach. [1] Among the etiologies of the ARF, Acute Lung Edema (ALE), decompensation of chronic obstructive pulmonary disease (COPD), chest trauma and pneumonia are the most frequent @It is a life-threatening pathology with a high incidence of mortality, since mortality is reported to be 20–40% [4, 5] for ARF secondary to cardiogenic ALE. The prevalence of ARF in Algeria remains unknown + the TAHINA study showed that respiratory diseases were the leading cause of consultation in the hospital [2]. The The objective of this study is to estimate the frequency of use of the NIV and to determine the associated factors of failure of the NIV for the adult patients hospitalized for ARF in the emergency department of Oran hospital from January 2015 to November 2016. Prevalence of COPD was found in the 31.5% of tobacco subjects [3]. The number of patients hospitalized for chest trauma continues to increase, resulting in an increase in the number of patients admitted for ARF secondary to chest trauma.

**Patients and methods:** This is an observational and exhaustive study during the month of November, from the files of patients. The population-all subjects over 16 years hospitalized for an ARF at the reception and resuscitation units of the emergency department of Oran Hospital from January 2015 to November 2016.

**Results:** Ninety-seven patients were hospitalized for ARF during this period. NIV was used for 37 patients. 24 patients were acute lung edema. Univariate analysis showed that SPO2 was the only failure factor in this series. The failure rate of this technique was 32.4%.

**Conclusion:** NIV is a technique that should be used more in the emergency rooms, which would make it possible to use less intubation specially in indications where the level of proof in the literature is important.

### P-37 Prognosis of acute respiratory failure after bronchiectasis exacerbation

#### Mnif Karama^1^, Tilouche Nejla^1^, Lahmar Manel^1^, Sikali Habiba^1^, Gharbi Rim^1^, Fekih Hassen Mohamed^1^, Elatrous Souheil^1^

##### EPS Taher Sfar, Mahdia, Tunisia

###### **Correspondence:** Mnif Karama - Karamamnif@gmail.com

*Annals of Intensive Care* 2018, **8(Suppl 1):**P-37

**Introduction:** In Tunisia tuberculosis is the most common etiology of bronchiectasis. In our intensive care bronchiectasis exacerbation is the second pulmonary cause of admission after COPD exacerbation. Few studies have assessed prognosis of bronchiectasis exacerbation requiring mechanical ventilation. Objectives—to determine the prognosis of bronchiectasis exacerbation and factors associated with mortality in these patients.

**Patients and methods:** In this retrospective study we included all patients hospitalized between 2001 and 2016 in the Medical intensive care for exacerbation of bronchiectasis and who required invasive or noninvasive ventilation. We excluded COPD exacerbation and all other causes of exacerbation of chronic pulmonary disease. We analyzed demographic characteristics, etiology of exacerbation, comorbidities, the SAPSII score, arterial blood gases at admission, respiratory, hemodynamic and neurological parameters, use of noninvasive or invasive ventilation, nosocomial infection, duration of NIV, length of stay and mortality.

**Results:** During period study 160 patients (58% women with a SAPSII score 29 ± 15) were included. The etiology of exacerbation was bronchitis in 45% of cases and pneumonia in 40%. Only 3 patients have NIV at home and 6 patients have oxygen. *Pseudomonas aeruginosa* was isolated in 6 cases. Twenty percent of the patients had developed a nosocomial infection, Acinetobacter Baumanii and *Pseudomonas aeruginosa* were isolated in 60 and 25% respectively. NIV was used in 89 patients at admission and the rate of NIV failure was 29%. The duration of mechanical ventilation was 9 ± 8 days and the length of stay was 12 ± 10 days. The mortality was 12%. NIV and oxygen at home were prescribed for 21 patients. In univariate analysis survivors and non-survivors were comparable regarding baseline and clinical characteristics. Nosocomial infections (53 vs. 16%), and SPASSII score were significantly more elevated in non-survivors. Noninvasive ventilation was used in 50 versus 64% (p = 0.02) in non-survivors and survivor patients respectively. In multivariate logistic regression analysis, factors associated with mortality were: nosocomial infection (OR 5, 95% IC (1.7–14.42) p = 0.003) and the use of invasive ventilation (OR 2.87, 95% IC (1.04–7.87), p = 0.04).

**Conclusion:** Exacerbation of bronchiectasis that requiring mechanical ventilation is associated in this study with low mortality. Nosocomial infection and invasive ventilation were the predictive factors of mortality.

### P-38 High flow nasal oxygenotherapy for management acute hypercapnic respiratory failure in emergency departement

#### Jardin François^1^, Montini Anne Claire^1^, Labro Guislaine^1^, Winiszewksi Adrien^1^, Desmettre Thibaut^1^, Capellier Gilles^1^

##### ^1^CHU de Besançon, France

###### **Correspondence:** Jardin François - francois.jardin@wanadoo.fr

*Annals of Intensive Care* 2018, **8(Suppl 1):**P-38

**Introduction:** The indication of High-Flow nasal Oxygen (HFO) during Hypercapnic Acute Respiratory Failure is still discussed, although several studies have shown beneficial effects (sweep of dead space, positive expiratory pressure, comfort, inspired gases control). The purpose of this study is to determine the place of HFO to cure hypercapnic acute respiratory failure in an Emergency Department.

**Patients and methods:** Observational study including adults patients in ED treated for hypercapnic acute respiratory failure with HFO from 05 27 15 to 02 15 17. Collected parameters—epidemiology, clinical and biological parameters, management in the emergency department (ventilatory support), diagnosis and evolution.

**Results:** 103 patients were treated with HFO but only 21.35% of them suffering from hypercapnic failure (22 patients). Mean age—71.2 years old. Medical history—heart failure (36.4%) respiratory failure (59.1%) loss of autonomy (22.7%) progressive cancer (27.3%). Diagnosis was lung sepsis for 81.8% of patients. Non-infectious causes are cancerous, cardiac, inflammatory and mechanical. Limitation of active therapy was taken for 10 patients (45.5%), two of them were already considered in palliative care. Mean flow rate of HFO was 51 L min, with 47.8% of mean FiO_2_. 31.8% were treated by HFO because of contraindications, poor tolerance or no accept. 72.7% were dyspneic, 36.4% show distress respiratory signs, with 88% of mean oxygen saturation, 31 of mean respiratory rate, no hemodynamic instability. HFO was discontinued for 40.9% of patients and replace by non-invasive ventilation (NIV) for 88.8% of them. Two was treated by alternating NIV HFO. 81.8% were hospitalized in intensive care, 13.6% in critical care, and 1 in medecine service. The number of day of hospitalization was 12.8 days. 1 patient was intubated—27.3% placed under alternating HFO NIV—22.7% under NIV—22.7% under HFO. 45.5% of patients died.
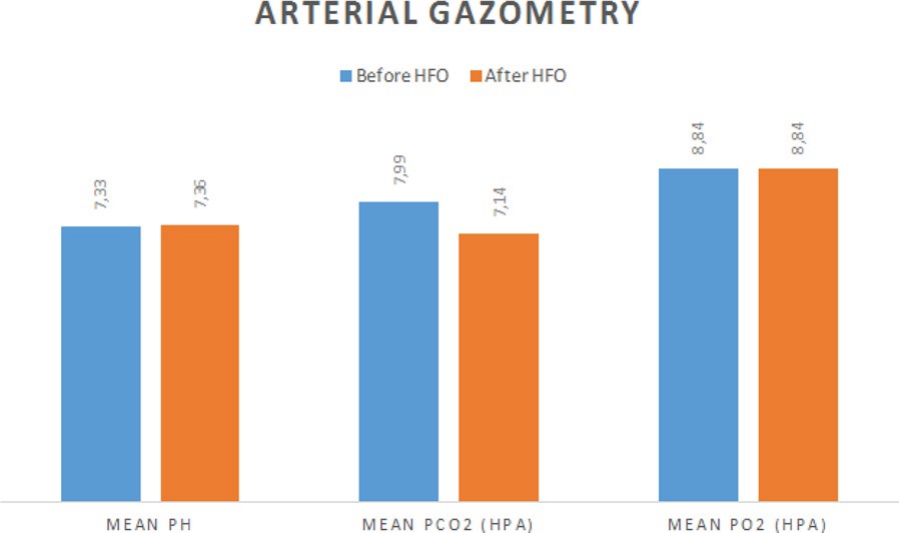


**Conclusion:** In emergency department, the management of hypercapnic acute respiratory failure with HFO is limited. Hypercapnia and acidosis remain moderate. Patients are old with comorbidities. The mortality rate is high but expected given the number of limitation of active therapy. HFO appears to be effective for a majority of patients, but half of them required NIV too. The NIV HFO association seems an interesting option. But our methodology is perfectible and would require a randomized control tria.

### P-39 Severe chronic obstructive pulmonary disease with chronic respiratory failure in intensive care unit: mortality and prognostic factors

#### Arnout Chloé^1^, Faure Morgane^1^, Novy Emmanuel^1^

##### CHU Nancy, Nancy, France

###### **Correspondence:** Arnout Chloé - arnout.chloe@gmail.com

*Annals of Intensive Care* 2018, **8(Suppl 1):**P-39

**Introduction:** Last decades, the number of patient with chronic respiratory failure due to chronic obstructive pulmonary disease (COPD) admitted in intensive care unit (ICU) increased. Data about their real prognosis in the ICU are lacking. The objective of this study was to evaluate mortality rate at 6 months and to identify prognostic factors of COPD patients with chronic respiratory failure, treated with long term oxygen therapy (LTOT), admitted in ICU.

**Patients and methods:** A retrospective cohort study was conducted in the French university hospital of Nancy during years 2014–2015 on all COPD patients treated with LTOT admitted in ICU. Only the first admission was analysed. Patients were included if they had spirometry, blood gas and oxygen flow in the year before admission in ICU. Other causes of chronic respiratory failure, and patients with tracheostomy before ICU admission were excluded of the cohort. Hospitalizations were selected using the International Classification of Diseases, 10 th revision (ICD-10).

**Results:** One hundred and thirteen patients were included, 37 (33%) died in the first 6 months after ICU admission. Mortality rate in ICU was 10%. Severity of COPD was—mean BODE score 7 ± 2.1, number of exacerbation per year requiring hospitalization 3 ± 1.4. LTOT was used for 3.7 ± 3.3 years. Acute respiratory failure was the main frequent cause—38% pneumonia, 34% acute exacerbation of COPD, 14% acute lung oedema. The Sequential Organ Failure Assessment score within the first 24 h of ICU admission was 11 ± 4. Need for mechanical ventilation was noted in 22% of cases and was associated to mortality with an Odds Ratio of 3.5 (CI 95% [1.4–9] p = 0.01). In presence of other organ failure, mortality rate tends to increase. Patients with median PaO2 FiO_2_ ratio > 196 on first blood gas had a reduced risk of death (OR 0.4 + CI 95% [0.1–0.9], p = 0.01).

**Conclusion:** This is the first study to assess mortality at 6 month of patients with severe COPD requiring LTOT admitted in ICU. Severity of hypoxemia and use of mechanical ventilation are two prognosis factor of mortality. The addition of another organ failure seems to increase the mortality rate. Severity of the chronic respiratory insufficiency less influenced short and long term outcome. This data have to be included in the global decision to admit a COPD patient with LTOT in ICU.

### P-40 Why asthmatics are again admitted to intensive care: a Tunisian series

#### Hammouda Zeineb^1^, Nouira Wiem^1^, Boukadida Sana^1^, Kheder Ahmed^1^, Alkortli Said^1^, Sik Ali Habiba^1^, Bousarsar Mohamed^1^, Elatrous Souheil^1^, Besbes Ouanes Lamia^1^

##### ^1^Medical ICU Monastir, Monastir, Tunisia

###### **Correspondence:** Hammouda Zeineb - zanoubia83@hotmail.com

*Annals of Intensive Care* 2018, **8(Suppl 1):**P-40

**Introduction:** The remarkable progress in the outpatient care of the asthmatic patient (development and access to inhaled drugs) has made the admission of these patients exceptional in the ICU. We have noticed a recent upsurge in asthmatic afmissions in the ICU, and are investigating whether this fact was related to modifiable factors (access to adapted drugs) or an increase in the severity of the disease.

**Patients and methods:** Retrospective, observational, three-center study conducted in three Tunisian medical ICU from January 2016 to July, 2017. Were included all consecutive patients admitted for severe acute asthma in three ICUs. Were assessed—patient’s demographic characteristics, asthma severity and its actual control based on Global Initiative for Asthma classification (GINA) 2016, clinical characteristics of the acute episode, length of ICU stay, ventilatory free days and mortality.

**Results:** Out of the1142 patients admitted within the study period, 34 (3%) had severe acute asthma. The mean age was 37 years (IQR32-50.5). Sex ratio was 1. Asthma was allergic in 84% with an average ancienty of 14.7 years. Over all asthma was not very severe with no prior ICU admission for acute severe asthma 38.2% were mechanically ventilated at least one time. Were classified severe and moderate persistant asthma respectively in 42.9(%) and 19(%). 57.1% were consideredpoorly controlled. Low educational level and socio-economic status are the main determinants of poor control-20% of analyzed patients didn’t have a social care, and thus no accesse to prescribed anti-asthmatics + 40% didn’t have a regular follow up and 23.5% were jobless. When admitted to the ICU-23 patients (67.6%) needed invasive mechanical ventilation, one patient received NIV. The mean length of stay was 8 days (IQR 3.5–12.5). Levels of auto PEEP and PIC pressure at ICU admission were respectively 6 (IQR 1–12) and47.5 (IQR 37, 25–58.5) cm H_2_O. Mortality rate was 3%.

**Conclusion:** This study suggests that Low educational level and socio-economic status (especially the lack of social care and joblessness) are the main determinants of poor control of asthma and may lead to the increase of rate of ICU admission for severe acute asthma requiring mechanical ventilation.

### P-41 Boussignac therapy: How can we control FiO_2_ decreases while minute ventilation increases?

#### Ilunga Ndaya Myriam^1^, Ebogo Titus^1^, Jacques Jean Marie^1^, Cuvelier Grégory^2^, Machayekhi Shahram^1^, Duprez Frédéric^3^

##### ^1^Centre Hospitalier Epicura Hornu, Boussu, Belgique; ^2^Ecole Condorcet, Tournai, Belgique; ^3^Baudour, Belgique

###### **Correspondence:** Ilunga Ndaya Myriam - myriam.ilunga@gmail.com

*Annals of Intensive Care* 2018, **8(Suppl 1):**P-41

**Introduction:** In emergency medicine, the Boussignac system (BS) is sometimes used to administer oxygen and continuous positive airway pressure (cpap). In this case, FiO_2_ value depends on the ratio between O_2_ flow and inspiratory flow (IF). In some cases, the FiO_2_ decreases due to the IF increase. The aim of this study was to test a modified Boussignac system in order to limit the FiO_2_ decreases during inspiratory flow rate increases.

**Patients and methods:** The study was conducted on bench with BS connected to a two compartment adult lung model (Dual Test Lung^®^) (DTL) controlled by a Maquet Servo I^®^ventilator. Three minute ventilation (MV—7.5 15 22.5 L min) with Ti Ttot = 0.33 were investigated. FiO_2_ and MV measurements were made using an iWorx^®^ GA207 gas analyzer. With a BS, two Peep were analyzed—3 and 10 cm H2O. The BS was supplied by an O_2_ flow. In order to increase the FiO_2_, we have evaluated the addition of a T piece connected to a nebulizer at the air-room admission of a BS. The aerosol was supplied by an O_2_ flow of 9 L min. The O_2_ flow was analyzed in continuous with a calibrated mass flow meter (Red Y Vogtlyn™).

**Results:** When MV increases, the FiO_2_ decreases (p < 0.05). When Peep increases, FiO_2_ increases too (p < 0.05). The addition of an aerosol (O_2_—9 L min) to a BS increases the FiO_2_ (p < 0.05). However, in this last case, the gap between both FiO_2_ decreases with increases MV (Fig. 1).

**Fig. 1:**
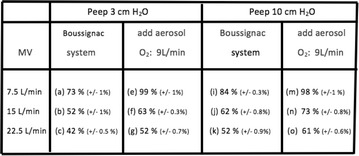
FiO_2_ differences between two peep values and with arid without add aerosol (supplied with O_2_ flow at 9 L/min). Means are expressed with SD

**Discussion:**

**Conclusion:** The addition of an aerosol connected to an O_2_ flow rate (9 L min) at the entry of a BS limits the FiO_2_ decreases during the MV increases.

### P-42 Distribution and antifungal susceptibility of Candida isolates in a Tunisian burn unit

#### Trabelsi Sonia^1^, Hanchi Majdi^1^, Bouchekoua Meriam^1^, Aloui Dorsaf^1^, Cheikhrouhou Sarra^1^, Khaled Samira^1^, Messadi Amen Allah^2^, Thabet Lamia^2^

##### ^1^Charles Nicolle Hospital, Tunis, Tunisie; ^2^Traumatology and great burned center, Tunis, Tunisie

###### **Correspondence:** Trabelsi Sonia - trabelsi.sonia@gmail.com

*Annals of Intensive Care* 2018, **8(Suppl 1):**P-42

**Introduction:** Burned patients are at high risk of yeast colonization and thus of invasive fungal infections, particularly to Candida (C.) spp., leading to an increase in morbidity and mortality. While pre-emptive antifungal therapy has improved survival, it may lead to an increase in antifungal resistance. The objectives of this work were to describe Candida species distribution and to determine the antifungal susceptibility of Candida isolates acquired in a burn unit.

**Patients and methods:** Our study is a retrospective review of 17 severely burned patients admitted to the burn unit of the Ben Arous Traumatology and Burns Center with one or more positive culture sites for Candida, during the 16-month period from May 2016 through August 2017. A total of 42 isolates were thus obtained. The susceptibility to 6 antifungal drugs (5-fluorocytosine, fluconazole, ketoconazole, micronazole, itraconazole, amphotericin B) was determined using the Fungitest^®^ broth dilution method for patients with infected normally sterile body sites or a Candida colonization index superior or equal to 0.4. Since echinocandin and anidulafungine were recently introduced in Tunisia, the susceptibility to these antifungal classes was tested for only one patient from our cohort.

**Results:** Nasal and buccal sites were the most colonized body sites (21.4% each), followed by axillary (11.9%) and rectal sites (9.5%) and urines (9.5%). *C. albicans* was the predominant species (45.2%), followed by *C. glabrata* (38.1%), *C. tropicalis* (7.1%) and *C. parapsilosis* (4.8%). Among the strains whose antifungal susceptibility was determined, majority of Candida isolates were susceptible to fluconazole (77.8%), which is the most frequently used molecule as a pre-emptive treatment in such cases in Tunisia due to its availability and its efficiency. On the other hand, 11.1% of the isolates were intermediate and 11.1% were resistant to this antifungal drug, mainly *C. glabrata* for both groups. As for the other tested azoles, high rates of intermediate strains were noticed (81.5% to itraconazole, 40.7% to ketoconazole and 33.3% to miconazole), mostly *C. glabrata*. Only one strain was resistant to amphotericin B, which is not usually used in these cases due to its nephrotoxicity and the frequency of kidney failure in burned patients.

**Conclusion:** Our study demonstrates that *C. albicans* is the most frequent species in burn unit-acquired candidiasis. No major antifungal resistance was observed, apart from high rates of intermediate strains (mainly *C. glabrata*) to azole class antifungal drugs.

### P-43 Bloodstream infections in burned patients

#### Mokline Amel^1^, Amri Helmi^1^, Khaled Ameni^1^, Gharsallah Lazheri^1^, Laajili Achref^1^, Rahmeni Imen^1^, Thabet Lamia^1^, Messadi Amen Allah^1^

##### ^1^Intensive Burn Care Department, Burn and Trauma Center, Tunis, Tunisie

###### **Correspondence:** Mokline Amel - dr.amelmokline@gmail.com

*Annals of Intensive Care* 2018, **8(Suppl 1):**P-43

**Introduction:** Infection, especially bacteremia, is a major cause of morbi-mortality in severely burned patients. Mortalityrelated to bacteremia in burn patients was about 30% [1]. We performed this study to determine the prevalence, the causative agents and outcomes of bacteremia in burned patients.

**Patients and methods:** Were reviewed patients admitted to intensive burn care unit in Tunis from the 1st of January 2016 to 31 of December 2016. Were included all patients who developed one or more cultures positive for bacteria. Demographic data of patients, species and number of cultures of bloodstream infections (BSI) were collected.

**Results:** During the study period, 320 patients were admitted, from which 35 developed 39 cases of BSI. The mean age was 40 ± 17 years. The mean body surface area burned was 38 ± 20%. BSIs were monomicrobial in 54% of cases. Gram-negative organisms caused 80% of these BSIs, Gram-positive organisms caused 18%. The organisms causing BSIs were *Pseudomonas aeruginosa* (30%), Acinetobacter baumannii (15%), Klebsiella P (15%) and Staphylococcus aureus (15%). The main factors significantly correlated to the occurence of BSIs are detailed in Table 1. The mean interval between admission and infection was 7 ± 6 days. The ICU stay was 24 ± 19 days. The mortality was 43%. The mortality was significantly correlated with the gravity of the bacteremia—60% in BSI with septic shock versus 40% in BSI without septic shock.

**Table 1:** Occurence of blood stream infections (BSIs)

Arterial catheterization		
Number	2.7 ± 1.7	p = 0.043
Duration	12.4 ± 8	p = 0.038
Venous catheterization		
Number	2.6 ± 1.9	p = 0.008
Duration	13.3 ± 8.6	p = 0.014

**Conclusion:** In our study, bloodstream infections occurred in 12% in severely burned patients. The main pathogens are *Pseudomonas aeruginosa*, Acinetobacter baumannii and Staphylococcus aureus. The mortality rate of these patients was 43%.

### P-44 Role of carbapenemase detection in optimization antimicrobial therapy in burns

#### Mokline Amel^1^, Khaled Ameni^1^, Gharsallah Lazheri^1^, Belhaj Salah Nada^1^, Rahmeni Imen^1^, Thabet Lamia^1^, Messadi Amen Allah^1^

##### ^1^Burn and Trauma Center, Tunis, Tunisia

###### **Correspondence:** Mokline Amel - dr.amelmokline@gmail.com

*Annals of Intensive Care* 2018, **8(Suppl 1):**P-44

**Introduction:** Carbapenems, the last line of therapy, are now frequently needed to treat nosocomial infections, and increasing resistance to this class of β-lactams limit antibiotic options in critically ill patients especially in burns. The objective of our study was to assess the impact of the detection of carbaménépases in optimizing treatments in burned.

**Patients and methods:** A prospective, monocentric study was carried out at the intensive care unit of burn in Tunisia over 6 months (March–August 2017). Were included all patients who have had a carbapenemase research. The sample was carried out by rectal swab. All samples were analyzed by polymerase chain reaction (PCR) methods for presence of carbapenemase.

**Results:** During the study period, 30 patients were included. The mean age was 30 ± 11 years. They were 24 men and 6 women. The average burned surface area was 41 ± 17%. Patients were transferred from another hospital structure in 87% of cases with a delay of 86 h. 90% of patients had a septic complication with a delay of 5 ± 3 days. Antibiotic treatment was empirical in 23 cases. The therapeutic failure rate was 60%. Results of carbame-nepases detected by PCR are detailed in Table 1. In the group of patients PCR (+), the antibiotic treatment was changed in 18 cases. The most association of antibiotics were—tigecycline in combination with colistin or in combination with fosfomycine and fosfomycin in combination with colistin. This leads to reduce therapeutic failure by 20%.

**Table 1:** Results of carbame-nepases detected by PCR

PCR	Positive +	Negative −	
	24 (80%)	6 (20%)	
Enzymes	OXA-48	VIM	NDM
	43.3%	70%	60%

**Conclusion:** Detection of carbapenemase in our study was higher (80%), allows us to identify regions with high risk of carbapenemase, improve therapeutic efficacy and strengthen infection control measures by isolation of all carbapenemase producing patient.

### P-45 Molecular characterization of carbapenemase-producing enterobacteriaceae in burn patients

#### Thabet Lamia^1^, Bouslah Zoubeir^1^, Maamar Beya^1^, Bourbiaa Yosra^1^, Messadi Amen Allah^1^

##### ^1^Hôpital de Ben Arous, Tunisia

###### **Correspondence:** Thabet Lamia - thabetlamia@gmail.com

*Annals of Intensive Care* 2018, **8(Suppl 1):**P-45

**Introduction:** The emergence of carbapenemase-producing Enterobacteriaceae (CPE) is an increasingly serious worldwide problem associated with high rates of therapeutic failure and mortality. Thus, early detection of CPE and rapid application of infection control measures is of paramount importance. We conducted a prospective study to characterize the molecular mechanisms and to determinate the antimicrobial susceptibility profiles of Enterobacteriaceae with decreased susceptibility to carbapenems isolated from burn patients.

**Patients and methods:** We examined 52 strains with reduced susceptibility to carbapenems among 327 Enterobacteriaceae clinical isolates collected between January 2017 and June 2017. Thirty-nine strains were selected for the study, one strain per species and per patient. The susceptibility of each strain was determined for a range of antibiotics involved, according to CA-SFM 2015 guidelines. Multiplex real-time PCR was performed with Cepheid’s GeneXpert Carba-R, allowing detection of the most prevalent carbapenemase gene families (blaVIM, blaNDM, blaIMP, blaOXA-48 and blaKPC). The 39 strains selected for molecular detection comprised 22 *Klebsiella pneumonia*e, 11 *Proteus mirabilis*, 3 *Klebsiella oxytoca*, 1 *Escherichia coli*, 1 *Enterobacter cloacae* and 1 *Providencia stuartii*, obtained from various biological samples.

**Results:** Of the 39 selected bacteria, 95% revealed ertapenem MIC > 1 mg l whereas only 30% showed imipenem MIC > 8 mg l. Twenty-eight bacteria (72%) expressed the blaNDM gene. The blaOXA-48 gene was found in 17 strains (44%), and 6 strains carried both genes. All *Proteus mirabilis* strains expressed the blaNDM gene. The antibiotics showing the highest resistant rates were cefotaxime (97%), piperacillin-tazobactam (87%), aztreonam (67%) and amikacin (57%). The most active agents were colistin (excepting with Proteus), fosfomycin and tigecycline with 98, 65 and 63% susceptibility, respectively.

**Conclusion:** The spread of CPE is an alarming problem in our center. The predominance of the blaNDM gene is reported for the first time in burn patients in Tunisia. Isolation of this resistance gene from *Proteus mirabilis* is particularly worrying. Detection of CPE by Xpert^®^ Carba-R was established in the center with a whole protocol of prevention.

### P-46 Epidemiological profile and antibiotic susceptibility of blood culture isolates from burn patients

#### Thabet Lamia^1^, Bouslah Zoubeir^1^, Bourbiaa Yosra^1^, Messadi Amen Allah^1^

##### ^1^Hôpital de Ben Arous, Tunisie

###### **Correspondence:** Thabet Lamia - thabetlamia@gmail.com

*Annals of Intensive Care* 2018, **8(Suppl 1):**P-46

**Introduction:** ICU-acquired bacteraemia is prevalent and poses a grave threat. Providing information about the main causative bacterial agents and determination of their susceptibility to antibiotics may improve empiric therapy and early detection of emerging antimicrobial resistance. The aim of this study was to investigate the species distribution and antibiotic susceptibility of isolated strains from blood culture in Burn Intensive Care Unit during a five-year period.

**Patients and methods:** From January 2012 to December 2016, a total of 2,182 non repetitive strains were isolated from blood cultures. Incubation of blood culture vials and the detection of bacterial growth were performed by the BACTEC system. All Isolated organisms were identified on the basis of standard microbiological techniques. Antibiotic susceptibility testing was carried out by the agar disk diffusion method, and susceptibility results were interpreted using clinical breakpoints according to CA-SFM 2013 and 2015 guidelines. Data were analyzed using the SIR-system. Minimum inhibitory concentrations of colistin, imipenem and vancomycin were determined using the Etest^®^ method (bioMérieux).

**Results:** Of the 2,182 strains isolated, the most frequently identified species were Staphylococcus aureus (21%), Acinetobacter baumannii (11%), *Klebsiella pneumonia*e (10%), and *Pseudomonas aeruginosa* (9%). The rate of methicillin-resistant Staphylococcus aureus (MRSA) was 70%. Resistance to tigecycline and linezolid was 15 and 1%, respectively. All strains were susceptible to glycopeptides. In addition, isolated Acinetobacter baumanii strains showed high rates of resistant to all tested antibiotics except colistin. Eighty per cent of these strains were resistant to ceftazidime and 94% to imipenem. Resistance to rifampicin was 15% in 2014, and has increased steadily to 67% by 2016. Similarly, high resistance rates were observed among *Klebsiella pneumonia*e and *Pseudomonas aeruginosa* to ceftazidime (70 and 39% respectively), ciprofloxacin (68 and 67%) and imipenem (15 and 72%).

**Conclusion:** This study investigated on the local distribution patterns of causative organisms of bacteraemia in burn patients and the corresponding antimicrobial susceptibility profiles. Multidrug-resistant pathogens, especially MRSA and Acinetobacter baumanii, were the most frequently isolated organisms. Hygiene measures and antimicrobial stewardship should be implemented to prevent the spreading of these resistant strains.

### P-47 Colonization with carbapenemase-producing gram-negative bacilli in burn patients in tunisia

#### Messadi Amen Allah^1^, Bouslah Zoubeir^1^, Khaled Ameni^1^, Maamar Beya^1^, Rahmani Imen^1^, Thabet Lamia^1^

##### ^1^Hôpital de Ben Arous, Tunisia

###### **Correspondence:** Messadi Amen Allah - amen933@gmail.com

*Annals of Intensive Care* 2018, **8(Suppl 1):**P-47

**Introduction:** Burn patients are at risk for multi-drug resistant (MDR) bacterial infections with high mortality rate. Therefore, monitoring the emergence of MDR pathogens in these patients is important. This study aimed to initiate the follow-up of digestive colonization with carbapenemase-producing Gram-negative bacilli in patients admitted to the Burn Intensive Care Unit.

**Patients and methods:** Our study was transversal and conducted over a period of 6 months (February 2017 to August 2017). Every patient admitted, especially when transferred from another hospital, was subjected for the screening. A double swab set was used to collect rectal swab specimens. One swab was used for classical bacteriology and MDR bacterial screening by chromogenic media, and the other swab was reserved for multiplex real-time PCR, which was performed by Cepheid’s Gene Xpert Carba-R allowing detection of the 5 most prevalent carbapenemase gene families—blaVIM, blaNDM, blaIMP, blaOxa-48 and blaKPC.

**Results:** Forty-four patients were screened on admission for carbapenemase-producing Gram-negative bacilli. Twenty-nine samples (65.9%) were positive by the Xpert Carba-R assay. Most of patients with positive samples were transferred from another hospital (76%), mainly from the southern hospitals, and one patient from Libya. The molecular characterization of detected carbapenemases showed the simultaneous presence of multiple target genes in most of samples—three genes (blaVIM + blaVDM + blaOxa-48) in 14 samples (48.2%), both blaVIM + blaNDM genes in 4 samples (13.8%), and both blaVIM + blaOxa-48 genes in 2 samples (6.8%). Furthermore, 9 samples were positive for only one target gene, as follow the blaVIM in 5 samples (17.2%) and the blaNDM gene in 4 samples (13.8%). The classical bacteriology and parallel screening of MDR bacteriaby chromogenic media detected the presence of carbapenem-resistant Gram-negative bacilli in all molecular positive samples.

**Conclusion:** Most of the patients included in this study were carriers of carbapenemases, 76% of them were transferred from another hospital. Our study describes the characterization of carbapenemases in burn patients and highlights their alarming spread. Thus, detection of colonization may guide the empirical antibiotic therapy. This emphasizes the importance of an isolation policy of transferred patients, and the need of a strategy to limit the spread of the MDR organisms.

### P-48 Frequency of *Pseudomonas aeruginosa* serotypes in burned patients and their resistance to antibiotics

#### Thabet Lamia^1^, Bourbiaa Yosra^1^, Bouslah Zoubeir^1^, Messadi Amen Allah^1^

##### ^1^Hôpital de Ben Arous, Tunisia

###### **Correspondence:** Thabet Lamia - thabetlamia@gmail.com

*Annals of Intensive Care* 2018, **8(Suppl 1):**P-48

**Introduction:** *Pseudomonas aeruginosa* is known opportunistic pathogen frequently causing serious infections in burned patients. Multidrug resistance in this pathogen is increasing throughout the world and is a major problem in the management of these pathogens. Analysis of serotype and resistance profile to antobiotics of *P. aeruginosa* help to establish a prompt control and prevention program. The aim of this study was to evaluate the frequency of antimicrobial resistance and the prevalence of *Pseudomonas aeruginosa* serotypes isolated in the burn unit.

**Patients and methods:** During a period of 4 years (from 01 01 2012 to 31 12 2015), 485 strains of *Pseudomonas aeruginosa* were isolated from burned patients. Conventional methods were used for identification. Antimicrobial susceptibility testing was performed with disk diffusion method and susceptibility data were interpreted according to breakpoints recommended by the French Society of Microbiology (FSM). Serotypes were identified by slide agglutination test using *P. aeruginosa* O antisera (Biorad). The imipenem-resistant strains have benefited from a research of carbapenemase production by the EDTA test.

**Results:** In our study period, 3464 bacterial isolates were found among which *Pseudomonas aeruginosa* was the second most frequent bacterium isolated from burned patients (14%) after Staphylococcus aureus (18%). The most frequent sites were—cutaneus infection (36%), blood culture (24%) and catheter (16%). The most prevalent serotypes were—O12 (72%), O1 (10%), O2 (6%), O11 (1%) and O6 (1%). The survey of antibiotic susceptibilily showed high pourcentage of resistance to the different antibiotics—21% of strains were resistant to ceftazidim, 69% to ticarcillin, 66% to ciprofloxacin, 62% to amikacin and 56% to imipenem. Among the 272 imipenem resistant strains, 34% were metallo-beta-lactamase producers. The antibiotic to which *P. aeruginosa* was the most susceptible was colistin (100%). Multidrugresistance was associated with O12 serotype in 67% of the cases.

**Conclusion:** The global frequency of serotypes O12, O1 and O2 was more than 88%. Multidrug resistance and carbapenemase being associated with serotype O12. Serotyping of the strains isolated from burned patients will help to guide the first antibiotherapy. The dissemination of carbapenemases strains must be contained by implementation of timely identification, strict isolation methods and better hygienic procedures.

### P-49 Pronostical factors in abdominal chirugical sepsis

#### Khaleq Khalid^1^, Hattabi Khalid^1^, Bensardi Fatima Zahra^1^, Bouhouri M. A^1^, Nciri A^1^, Hamoudi D^1^, Alharrar R^1^

##### ^1^Centre Hospitalier Universitaire Ibn Rochd, Casablanca, Morocco

###### **Correspondence:** Khaleq Khalid - khaleq20@gmx.fr

*Annals of Intensive Care* 2018, **8(Suppl 1):**P-49

**Introduction:** The abdominal sepsis is one of the most frequent digestive emergencies and one of the first causes of septic shock, involving the vital prognosis of the patients who are often elderly or carriers of subjacent pathologies. It’s management is multidisciplinary. The purpose of our study is to describe the epidemiological, clinical, bacteriological, evolutionary data of abdominal sepsis, to evaluate the predictive factors of the mortality.

**Patients and methods:** We realized a retrospective descriptive and analytical study spread over 3 years about 200 cases of abdominal sepsis. The adult patients with community or postoperative abdominal sepsis were included in our study and received medical and surgical care. The parameters studied are demographic, clinical, and radiological, per operative, bacteriological and evolutionary data. The statistical analysis was carried out using the SPSS software, the valuation of the prognostic factors made use of a varied and analysis.

**Results:** The incidence of the abdominal sepsis during the study represented 24%, it was divided into community sepsis (72%) and postoperative sepsis (28%). The manage was 50.80 ± 18.28 years old, with a sex ratio of 1.4. The clinical signs were dominated by the abdominal pain (66.5%), fever (63.5%), extra-abdominal signs (hemodynamic insufficiency (70%), renal failure (66%), hematological disorders%) And respiratory disorders (43.5%)…). The therapeutic management was based on per operative resuscitation, organ failure treatment, probabilistic antibiotic therapy and median laparotomy surgery. The main etiologies of abdominal sepsis were—digestive perforations (43.5%), purulent effusion (38%), intestinal necrosis (14%), cholecystitis (12.5%). The bacteriological profile was -predominance of BGN (82.7%) dominated by E. Coli (28%) followed by *Klebsiella pneumonia*e and Acinetobacter baumanii (13.46%), the mean duration of the hospitalization was 7.03 ± 6.65 days. The mortality rate was 57%. The main prognostic factors in our study in univariate analysis were—the advanced age, the diabetes, the organ failure, the increased gravity scores, the time to management, the use of catecholamines and the development of septic shock. The multivariate analysis showed a statistically significant association between the development of septic shock, the stercoral effusion, the peptic ulcer perforation, the operator and the therapeutic descalation.

**Conclusion:** The abdominal sepsis is a serious affection, with great mortality. The improvement of its prognosis is based on a revision of the medical and surgical protocols, and an adapted antibiotic therapy depending on the direct examination of the samples, also of the bacterial ecology of the service.

### P-50 Prognostic factors of postoperative peritonitis

#### Mayola Vanadiaku Ulrich^1^, Khaleq Khalid^2^, Majida Mghari^2^, Bouhouri Aziz^2^, Nsiri Afak^2^, Hamoudi Driss^2^, Al Harrar Rachid^2^

##### ^1^Hôpital Beausejour, Casablanca, Morocco; ^2^University Hassan II, Casablanca, Morocco

###### **Correspondence:** Mayola Vanadiaku Ulrich - ulrichvan2002@yahoo.fr

*Annals of Intensive Care* 2018, **8(Suppl 1):**P-50

**Introduction:** Postoperative peritonitis (PPO) is a serious complication of abdomino-pelvic surgery. It is a medical-surgical emergency, the prognosis of which depends on the speed and quality of management, the underlying terrain and etiology. The aim of our study is to elucidate the prognostic factors of postoperative peritonitis in a series of 100 patients.

**Patients and methods:** It is a descriptive analytical retrospective study, Four-year period (from junuary 2013 to December 2016), 100 observations of postoperative peritonitis were collected in the Surgical intensive care unit of the CHU Ibn Rochd Casablanca. The statistical analysis was carried out using the SPSS 20 software. The results are expressed with OR and 95% confidence intervals (95% CI). The results were considered significant when P is < 0.05.

**Results:** The average age of patients was 46 ± 14 years with a sex ratio of 1.94. The risk factors most frequently were-field factors, factors associated with initial surgery (with sub-mesocolic site, which was the largest provider of POP 53%, mainly dominated by the colorectal region 38%). Polyvisceral failure was present in 92% of patients. Diagnostic time was 6.55 days. Intraoperative bacterial sampling allowed the following bacteriological profile -Predominance of BGN (70%) dominated by *E. coli* (25%). After which *Klebsiella pneumonia* (18%), Acinetobacter baumanii (15%) and enterococci (18%). Multi-microbial character was found in 55% while 45% was mono-microbial. Candida accounted for 17% of isolated microorganisms. The mortality rate was 68%. The prognostic factors in the multivariate analysis were IRA (OR 28.013, 95% CI between 3.009 and 260.768, p = 0.003), hepatic failure (OR 10.889, 95% CI between 1.633 and 72.625, p = 0.014). The fungal infection (OR 12.649, 95% CI between1.224 and 130.767, p = 0.033) IGS2 score (OR 1.080, 95% CI between 1.015 and 1.150, p = 0.015) SOFA score (OR 1.911, 95% CI between 1.405 and 2.598, p < 0.001).

**Conclusion:** Postoperative peritonitis is a serious complication of abdominal surgery, diagnosis is often difficult. Support based on a multidisciplinary approach in which the anesthesiologist plays a central role. Only effective therapeutic management and reduces early mortality remains high in recent years, despite various advances in the field of surgery and resuscitation.

### P-51 Severe acute respiratory infections of viral origin in Moroccan medical ICU: incidence and prognostic factors

#### Dendane Tarek^1^, Cherkaoui Imad^2^, Elmassaoudi Imane^3^, Oumzil Hicham^2^, Belayachi Jihane^3^, Madani Naoufel^3^, Abouqal Redouane^3^, Abidi Khalid^3^, Zeggwagh Amine Ali^3^

##### ^1^Service de Réanimation Médicale, Rabat, Maroc; ^2^Ministère de la santé, Rabat, Maroc; ^3^Mohamed V University, Rabat, Maroc

###### **Correspondence:** Dendane Tarek - tdendane@hotmail.com

*Annals of Intensive Care* 2018, **8(Suppl 1):**P-51

**Introduction:** Severe acute respiratory infections (SARI) are common in critically ill patients. Viruses can be found in immuno-competent patients. However, the main problem for viral infections is the diagnosis, isolation of the pathogen is often difficult and the symptoms not specific. The aims of this study were to describing the epidemiological characteristics of viral respiratory infections, to identify factors predictive of a poor outcome.

**Patients and methods:** A retrospective study was conducted at the Medical Intensive Care Unit of the University Hospital Ibn Sina, Rabat-Morocco from July 2014 to August 2016. A total of 140 adult patients were admitted over the study period, and laboratory tested for influenza and other respiratory viruses by PCR. Several variables were collected and analyzed. They were included in the study that all patients developed SARI with or without identified viruses. To identify factors associated with mortality, two other groups were compared—survivors versus died.

**Results:** Of the 140 patients admitted to ICU during the study period (mean age of 49.5 ± 18.4 years, 80 males, 60 females). 28 patients had positive viral specimens at a viral respiratory infection prevalence rate of 20% (28 140) and an incidence rate of 3, 5%. The mean Apache II score was 15.5 ± 7.1. The main cause of admission was a COPD (27%). 14.3% of patients had septic shock at admission and 45.7% had severe sepsis. The use of mechanical ventilation was necessary in 42% of the cases. The chest radiograph was abnormal in 73.6% of the cases. The main isolated SARI viruses were influenza A virus (8.6%) and influenza B virus (2.1%) as well as others respiratory viruses (9.3%). The mortality of patients with viral SARI was 28.6%. The overall mortality rate was 37%. Mortality rates were higher in patients with influenza A virus (50%) or septic shock at admission (85%). Presence of septic shock (OR 30.3 + 95% CI 4.2–219.2), prior antibiotic consumption (OR 10.6 + 95% CI 2.1–53.0), high APACHE II score (OR 1.2 + 95% CI 1.1–1.4), were independently associated with an elevated risk of death.

**Conclusion:** The incidence of SARI remains low, but mortality is high. This mortality appears to be related to the presence of a septic shock at admission, a high APACHE II score, and to a preliminary antibiotic treatment.

### P-52 Identification of predictive elements of insufficiency Amikacin peak in patients with septic shock and severe sepsis

#### Chalvon Nellie^1^, Pierre Malika^1^, Bekkhoucha Hamza^1^, Cordonnier Aurélien^1^, Prieur Nathalie^1^, Mateu Philippe^1^

##### ^1^Hôpital de Charleville-Mezieres, Charleville-Mezieres, France

###### **Correspondence:** Chalvon Nellie - nellie390@hotmail.fr

*Annals of Intensive Care* 2018, **8(Suppl 1):**P-52

**Introduction:** In septic shock there are physiological changes with an increase in the volume of distribution, with implications for pharmacokinetics of antibiotics that make recommended doses potentially inadequate for target organisms with highest minimal inhibitory concentrations. To cover these bacteria, peak serum concentration (Cmax) target is 64–80 pg ml. Identification of predictive factors for insufficient Cmax, in common practice, would make it possible to target the patients at risk in order to optimize dosage of antibiotic to be administered. Objective of this study was to determine predictive factors of Amikacin’s Cmax insufficient independently of the dosage.

**Patients and methods:** This was a retrospective study carried out between August 2013 and November 2015 in ICU of our Hospital. All adult patient receiving an initial injection of Amikacin between 20 and 30 mg kg were included. Clinical data collected were—Amikacin dosage, Body Mass Index (BMI), Mechanical ventilation (MV), mean arterial pressure (MAP), use of Noradrenaline and continuous hemofiltration (CVVH). Biological elements were collected and for each, the last result in the 24 h prior to admission and that at the patient’s entry into ICU were added to analysis. A comparison of this clinical and biological variables was made between two groups—the first one with an ineffective Cmax of Amikacin (< 64 pg ml) and the second with an effective Cmax of Amikacin (> 64 pg ml).

**Results:** 191 patients were selected for statistical analysis. Median dosage was 22.5 mg kg for a median Cmax at 72.1 mg l. For 77 patients, Cmax was less than 64 mg l and in 53 patients, it was greater than 80 mg l. There was a statistically significant relationship between a Cmax greater than 64 mg l and MV, BMI, PCT measured before and after admission, albumin after admission, hemoglobinemia, hematocrit level after admission, the rate of urea after admission (Table 1). A low BMI was associated with Cmax < 64 mg l.

**Table 1:** Study of the association between Cmax and the studied parameters for an objective of Cmax greater than 64 mg/l

	Cmax < 64 mg/l N = 77/191		Cmax > 64 mg/l N = 114/191		p	
Dosage (mg/kg)	22.3 SD 2.6		22.74 SD 3.6		0.52	
BMI (kg/m^2^)	24.6 SD 7.8		29.4 SD 9.4		<0.001	
MAP ≤ 70 mmHg	16/64 (25%)		34/104 (32.7%)		0.29	
MAP > 70 mmHg	33/73 (45.2%)		53/113 (47%)		0.8	
Mechanical ventilation	42/66 (64%)		51/109 (46.7%)		0.03	
Noradrenaline	41/73 (56%)		57/113 (50%)		0.44	
CVVH	4/66 (6%)		7/108 (6.4%)		0.92	

	PRE.ADM	POST.ADM	PRE.ADM	POST.ADM		
Hémoglobinemia (g/dl)	11.7 SD 3.11	10.56 SD 2.1	12.3 SD 2.6	11.28 SD 2.7	0.15	*0.01*
Hématocrit (%)	35.9 SD 8.8	32.6 SD 6.4	37.9 SD 8.2	34.9 SD 7.9	0.12	*0.03*
Protidemia (g/l)	61 SD 13	55 SD 8	61 SD 12	56 SD 9	0.88	0.23
Albuminemia (g/l)	22.6 SD 6.1	21 SD 6.8	24 SD 5.9	24 SD 6.6	0.13	*0.007*
Urea (mmol/L)	10.8 SD 8	10.7 SD 7.4	8.6 SD 5.8	8.6 SD 5.4	0.26	*0.028*
Créatininemia (μmol/L)	134 SD 102	138 SD 106	126 SD 102	123 SD 100	0.64	0.33
Procalcitonin (μg/L)	17 SD 45	18.1 SD 1.5	7.33 SD 13.5	7.7 SD 6	0.04	*0.05*

PRE.ADM: biological data before admission; POST.ADM: biological data after admission; MAP: mean arterial pressure; CVVH: continuous veno-venous hemofiltration

**Discussion:** These results remain comparable to those found by Taccone in 2010, with dosages of 25 mg kg having only 70% of the peaks above 64 mg l + comparable also to Montmollin’s study in 2014.

**Conclusion:** MV, BMI, pre- and post-admission PCT, and albumin, hemoglobin after admission, hematocrit and urea after admission seems to be predictive criteria for insufficient amikacin’s Cmax independently of dosage. Our study was limited to one ICU, a heterogeneous recruitment, and that all samples have been taken at the right time.

### P-53 Are standard doses of ceftriaxone adapted in critically ill patients with augmented renal clearance?

#### Petit Laurent^1^, Carrié Cédric^1^, Foumenteze Cecile^1^, D’Houdain Nicolas^1^, Breilh Dominique^1^, Sztark François^1^

##### ^1^CHU PELLEGRIN, Bordeaux, France

###### **Correspondence:** Petit Laurent - laurent.petit@chu-bordeaux.fr

*Annals of Intensive Care* 2018, **8(Suppl 1):**P-53

**Introduction:** This study aimed to assess whether augmented renal clearance (ARC) impacts negatively on ceftriaxone pharmacokinetic pharmacodynamics (PK PD) target attainment in critically ill patients receiving 2 g day by intermittent infusion.

**Patients and methods:** Over an 8-month period, all critically ill patients treated by ceftriaxone for a first episode of sepsis without renal impairment were eligible. During the first 3 days of antimicrobial therapy, every patient underwent 24-hour creatinine clearance (CrCL) measurements and therapeutic drug monitoring at trough concentrations. The main outcome investigated in this study was the rate of empirical target non-attainment using a theoretical target MIC of 2 mg/L.

**Results:** Over the study period, 21 patients were included (63 samples analyzed for therapeutic drug monitoring). The rate of PK/PD target non-attainment was 62%, with a strong association with CrCL (p < 0.0001) (Table 1). There was no statistical association between PK/PD target non-attainment and therapeutic failure.

**Table 1:** Percentages of PK/PD target attainment for various MIC and CrCL for cefazolin administered continuously (100 mg/kg/day)

	CrCL < 150 ml/min N = 15	150 ≤ CrCL < 200 ml/min N = 22	CrCL ≥ 200 ml/min N = 26
Targeted MIC ≥ 0.5 µg/ml	100 [100–100]	100 [100–100]	100 [100–100]
Targeted MIC ≥ 1 µg/ml	93 [81–100]	86 [72–100]	92 [82–100]
Targeted MIC ≥ 2 µg/ml	80 [60–100]	41 [20–62]	12 [0–24]
Targeted MIC ≥ 4 µg/ml	20 [0–40]	5 [0–13]	0 [0–0]

*Results expressed as percentages [95% confidence interval]*

**Conclusion:** When targeting 100%fT > MIC of the less susceptible pathogens, patients with CrCL > 150 ml/min are at risk of sub-exposure in ceftriaxone (2 g day). These data emphasize the need of therapeutic drug monitoring in patients with ARC, especially when targeting less susceptible pathogens or surgical infections with limited penetration of antimicrobial agents.

### P-54 Prognosis factors in septic shock in ICU in Military Hospital Avicenna in Marrakech (Morocco)

#### Qamouss Youssef^1^, Aissaoui Younes^1^, Serghini Issam^1^

##### ^1^Hopital Militaire Avicenne, Marrakech, Morocco

###### **Correspondence:** Qamouss Youssef - yqamouss@yahoo.fr

*Annals of Intensive Care* 2018, **8(Suppl 1):**P-54

**Introduction:** The septic shock is a major concern of the intensive care unit in the world because of its frequency and especially of its mortality which remains high in spite of the progress made in the optimizing care. The aim of our work is to analyze the prognosis factors related to death among patients with septic shock in the ICU of the military hospital Avicenna of Marrakesh, and to focus on the physiopathological and therapeutic data of the septic shock in the light of last acquisitions in this field.

**Patients and methods:** We proceed to a prospective study including all patients with septic shock at admission to ICU or secondary, over a 3-year period (January 2014 - December 2016). Prognosis factors related to death in patients with septic shock were studied in univariate and multivariate analysis.

**Results:** Eighty-six cases of septic shock were collected from 1290 ICU admissions, the incidence is 3.33%, the mean age was 58 ± 14.7. The sites of infection most often involved were the abdomen and lung (50%), there was a predominance of Gram-negative bacilli, the number of organ failure is in average 2.6 ± 1.1. The overall mortality was 65.1%. Prognosis factors related to mortality retained after logistic regression are cardiovascular organ failure followed by neurological. Indeed, the number of patients with 3 or more failures was 22 (78%) in the group of patients who died. As the second factor influencing the high mortality found severity score 53.6 ± 14.7, age is also considered a prognosis factor since 21 of 28 patients were over 60 years. The average age of the deceased was 66 ± 7 years versus 51 ± 7 years in survivors (p < 0.001), yet the mortality according to the infectious agent was not found as factor influencing mortality (p = 0.75).

**Conclusion:** Septic shock is a frequent reason for hospitalization in ICU. The improvement of prognosis requires an early and adapted management of sepsis as well as increases efforts for control and prevention of nosocomial infection.

### P-55 Vitamin D deficiency in septic shock: a study of the prognosis

#### Chaari Anis^1^, El Bahr Mohamed^1^, Khalil Elsayed^1^, Elkomy Mustafa^1^, Bousselmi Kamel^1^, Alansari Mariam^1^

##### ^1^King Hamad university Hospital, Muharaq, Bahrain

###### **Correspondence:** Chaari Anis - anischaari2004@yahoo.fr

*Annals of Intensive Care* 2018, **8(Suppl 1):**P-55

**Introduction:** Vitamin D deficiency is common in critically-ill patients. In addition to its role in the regulation of phosphor-calcic metabolism, vitamin D is of paramount importance for the immune system. The aim of the current study is to assess the prognostic value of vitamin D deficiency in patients with septic shock.

**Patients and methods:** Retrospective study conducted over 6 months. All the adult patients with septic shock and vitamin D level screening performed within the first 24 h of admission were included in the study. We excluded patients with chronic kidney disease and those receiving vitamin D supply. Two groups were compared: those with a serum Vitamin D level < 10 ng/ml (G1) and those with higher level (G2).

**Results:** Thirty-three patients were included in the study. Median age was 70 [61–77.5] years. Sex-ratio was 1.2. Median APACHE II score was 19 [15–25]. Hypertension and diabetes were the common comorbidities (respectively 69.7 and 48.5%). Median serum vitamin D level was 15 [7.7–28] ng/ml. Low vitamin D was recorded in 11 patients (37.9%). Mechanical ventilation was required for 16 patients (48.5%). Nine patients (27.3%) required continuous renal replacement therapy. Seven patients died in the ICU (21.2%). The comparison of the two groups showed that patients with low vitamin D levels were significantly younger than the others (61 [52–68] versus 75.5 [66.8–80] years, p = 0.009). The ICU mortality was comparable between the two groups (18.2% in G1 and 22.7% in G2, p = 0.763).

**Conclusion:** Decreased vitamin D is common in patients with septic shock. Our study does not suggest that low vitamin D is associated with poor outcome.

### P-56 Sepsis-induced inhibition of malignant tumor growth is associated with quantitative and functional modulation of tumor-infiltrating macrophages

#### Mirouse Adrien^1^, Rousseau Christophe^2^, Alby-Laurent Fanny^2^, Toubiana Julie^2^, Mira Jean-Paul^1^, Chiche Jean-Daniel^1^, Pène Frédéric^1^

##### ^1^Hôpital Cochin, Paris, France; ^2^INSERM U1016, Paris, France

###### **Correspondence:** Mirouse Adrien - adrien.mirouse@gmail.com

*Annals of Intensive Care* 2018, **8(Suppl 1):**P-56

**Introduction:** Since immunity plays a central role in neoplasms surveillance, it is likely that sepsis induced immune dysfunctions may impact on the underlying malignancy. We developed a research project investigating the reciprocal relationships between bacterial sepsis and cancer. We reported that sepsis-induced immune suppression promoted tumor growth in post-septic mice inoculated with cancer. In a reverse cancer-then-sepsis model we observed that sepsis may conversely inhibit tumor growth. This study aimed at investigating the cellular and molecular mechanisms of sepsis-induced tumor inhibition, and most especially the role of monocytes macrophages and Toll-like receptor (Tlr) signaling.

**Patients and methods:** We used C57BL 6 J wild-type (WT), Tlr4-/-, Tlr2-/- and Myd88-/- mice. Mice were first subjected to tumor inoculation by subcutaneous injection of MCA205 fibrosarcoma cells. Fourteen days after, mice were subjected to polymicrobial sepsis induced by cecal ligation and puncture (CLP). Controls were cancer mice subjected to sham surgery. Alternatively, cancer mice were subjected to an i.p. challenge with Tlr agonist (LPS or heat-killed *Staphylococcus aureus* (HKSA)). The distribution of tumor-associated immune cells was assessed by FACS at days 1 and 7 following surgery. The activation status of tumor-infiltrating monocytes macrophages was assessed by FACS (MHCII, CD40, CD86, PDL1, PD1). F4/80 + cells were purified by FACS and we assessed cytokines production (rt-qPCR) and bacteria phagocytosis.

**Results:** We confirmed polymicrobial sepsis dampens tumor growth in WT mice. A similar CLP-induced tumor growth inhibition was observed in Tlr2-/- mice, but neither in Tlr4-/- nor Myd88-/- mice. A challenge with LPS resulted in a marked anti-tumoral effect, whereas a challenge with HKSA had no impact on tumor growth. Tumor-infiltrating immune cells analysis retrieved monocytes/macrophages predominance with two different subsets based on F4/80 expression (F4/80High and F4/80Low). Late-onset (day 7) tumors from CLP-operated mice displayed increased proportions of F4/80High. As compared to F4/80Low cells, F4/80High cells displayed a more immature status with a lower expression of CD40, MHCII and PDL1, and a higher phagocytic activity. Interestingly, F4/80High cells from CLP-operated mice exhibited a higher phagocytic activity than those from sham-operated mice.

**Conclusion:** Polymicrobial sepsis drives a potent antitumoral activity in cancer mice, which is associated with changes in the distribution and functions of tumor-associated monocytes macrophages subsets. Our results converge on a critical role of Tlr4 signaling, that should be further investigated.

### P-57 Critical sepsis and ILCs (CriSIs): a prospective study of circulating innate lymphoid cells in septic shock

#### Carvelli Julien^1^, Fraisse Megan^1^, Bourenne Jeremy^1^, Vely Frédéric^1^, Vivier Eric^1^, Gainnier Marc^1^

##### ^1^Réanimation des Urgences et Médicale, Marseille, France

###### **Correspondence:** Carvelli Julien - Julien.CARVELLI@ap-hm.fr

*Annals of Intensive Care* 2018, **8(Suppl 1):**P-57

**Introduction:** Immune response to bacterial infection is central in the pathophysiology of septic shock. It is essential to understand it thoroughly in order to identify new biological markers, new therapeutic targets whilst allowing a decrease in morbidity and mortality. After a cytokine storm syndrome, post-aggressive immunosuppression takes place. Within immunologic changes after a severe infection, innate lymphoid cells (ILCs) status remains unknown.

**Patients and methods:** We compared prospectively 9 patients hospitalized for septic shock in the medical intensive care unit (ICU) of the teaching hospital La Timone-Marseille to 10 control patients hospitalized in the same unit for another reason (cardiac arrest, trauma). Fresh blood samples were collected and analyzed. An extended lymphocyte phenotyping (B cells, T cells, NK cells, regulatory T cells and ILCs), was conducted as well as an evaluation of HLA-DR expression on circulating monocytes.

**Results:** There was a trend for more pronounced lymphopenia in septic patients but without statistically significant difference (620/mm3 [166–3987] versus 1300/mm3 [598–2246], p = 0.15). In addition, septic patients weren’t more susceptible to develop a secondary infection (2 secondary infections versus 4 in the control group, p = 0.4). However, septic patients exhibited a decrease in circulating ILC3 s (49 mL [5–124] versus 183 mL [38–1026], p = 0.01). An excess of ILC2 s was found to be associated with a higher risk to develop a secondary infection (314 mL [152–621] versus 104 mL [15–677], p = 0.05). Finally, immunosuppression was correlated with disease severity (SOFA < 8).

**Conclusion:** Post-agressive immunosuppression in ICU is not specific to sepsis. In septic shock, the low counts in circulating ILC3 s could be explained by ILC plasticity (conversion of these cells into ILC1 s), by migration from the blood or by an exacerbated apoptosis. ILC2 s expansion, associated with a higher risk of secondary infection, could be promoted by IL-33, released by tissue injuries. ILC2 s could activate regulatory T cells via IL-10. These preliminary results must be confirmed on a larger cohort.

### P-58 Measure of regulatory B cells level in peripheral blood of patients with severe sepsis or septic shock

#### Brault Clément^1^, Guignant Caroline^1^, Titeca Beauport Dimitri^1^, Marolleau Jean-Pierre^1^, Gubler Brigitte^1^, Maizel Julien^1^

##### ^1^CHU AMIENS PICARDIE, Amiens, France

###### **Correspondence:** Brault Clément - brault.clement@chu-amiens.fr

*Annals of Intensive Care* 2018, **8(Suppl 1):**P-58

**Introduction:** Sepsis is characterized by a dysregulation of the inflammatory response initiated by the infection. An extreme inflammatory state is responsible of severe shock, multiple organ failure and death. The compensatory anti-inflammatory response syndrome (CARS) includes immunological phenomenons which is aimed at reducing inflammation. Among them, regulatory B cells (Breg) are a lymphocyte subpopulation derived from transitional or marginal zone B cells. They play a suppressive role in the immune system by the secretion of negative regulatory cytokines such as interleukin-10 or by immune cell contact inhibition. The objective of this pilot study was to develop and test a protocol to determine the Breg level in septic patients.

**Patients and methods:** The level of Breg were measured on whole blood sample by flow cytometry the first day of hospitalisation in septic patients. B cells were identified on the single-parameter expression CD19 combined with scatter. The Breg were identified as subpopulation expressing CD24/hiCD38hi or CD24/hiCD27+ (see Fig. 1). The results were expressed as percentage of the parental lineage gate and absolute value per microliter. This protocol has been optimised in order to be able to transfer technic into clinical practice.

**Results:** We include 8 patients hospitalized in intensive care unit with severe sepsis or septic shock. The percentage of CD19 + CD24hiCD38hi was 6.6 ± 5.2% with a mean of 4.6 ± 2.3 cells microliter. The percentage of CD19 + CD24hiCD27 + was 9.1 ± 4.7% with a mean of 8.8 ± 6.4 cells microliter.

**Fig. 1:**
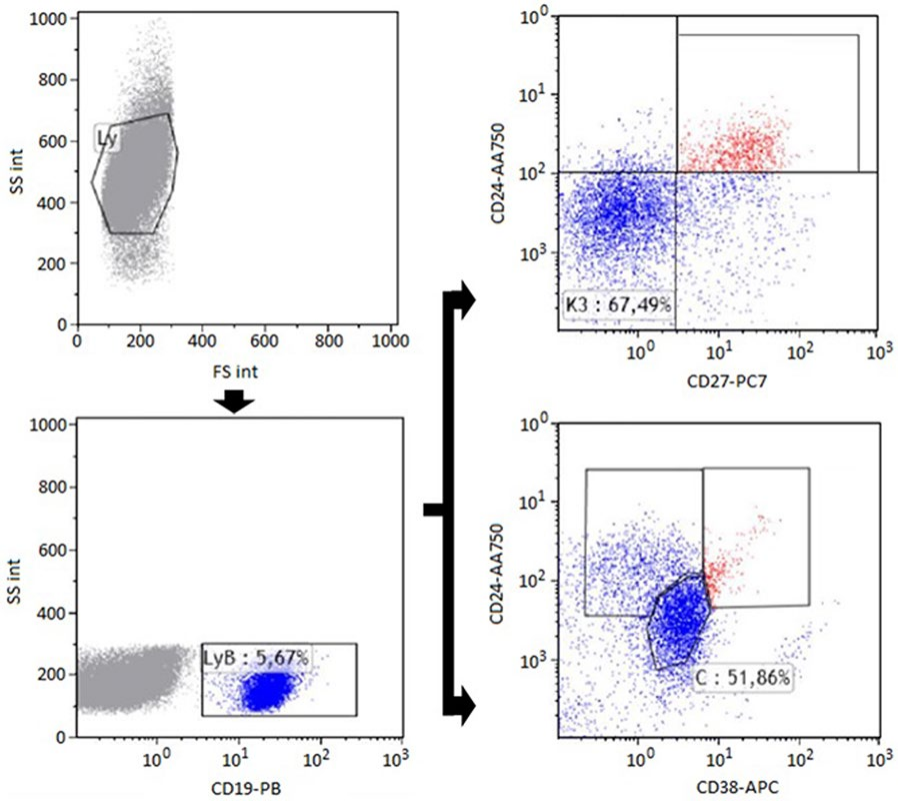
Breg levels in septic patients

**Discussion:** Many studies have confirmed a correlation between Breg level and the disease activity or the prediction of a therapeutic response in auto-immune or allergic diseases. In contrast, only two studies have evaluated Breg level in patients with sepsis. Pan et al. have shown a significant increase of Breg in neonatal sepsis patients compared to controls. While Wang et al. have shown a significant decrease of Breg in death group compared to survival group. For the moment, no study was interested in Breg for prediction of the occurrence of nosocomial infection.

**Conclusion:** We are able to measure and follow the evolution of Breg during severe sepsis or septic shock. Because Breg could inhibit body immune function, we wish to conduct a prospective study to evaluate the correlation between Breg level and the prognosis of patients with sepsis.

### P-59 Prognostic value of neutrophil-to-lymphocyte ratio in patients with septic shock: a prospective observational study

#### Jendoubi Ali^1^, Jerbi Salma^1^, Najar Nadim^1^, Abdelmalek Fares^1^, Mosbahi Boutheina^1^, Ghedira Salma^1^, Houissa Mohamed^1^

##### ^1^Charles Nicolle Hospital, Tunis, Tunisia

###### **Correspondence:** Jendoubi Ali - jendoubi_ali@yahoo.fr

*Annals of Intensive Care* 2018, **8(Suppl 1):**P-59

**Introduction:** The neutrophil/lymphocyte ratio (NLR) reflects an inflammatory state. The NLR has recently emerged as a prognostic marker in colorectal cancer patients, acute coronary syndrome and pulmonary embolism (Kayrak M, Heart Lung Circ 2014). The aim of this study was to assess the prognostic value of NLR in patients with septic shock.

**Patients and methods:** We performed a prospective observational study in septic shock ICU patients within 72 h of admission from January to July 2017 in Charles Nicolle Hospital of Tunis. Exclusion criteria were age < 18 years, pregnancy + oncohematological patients, recent blood transfusion, post-cardiac arrest and brain-death. NLR was measured soon after admission and 24h, 48h, and 72h after. Demographic, clinical and biochemical parameters, severity scores, life-support therapies (vasopressors, ventilation), and length of ICU stay were recorded. The primary endpoint was 28-day mortality.

**Results:** Sixty-five patients (50 males, median age, 48.5 years) with septic shock were included in the study. The 28-day mortality was 54%. The median SOFA score at T0 was 12 points and the median IGS2 score was 55 points. The sources of infection were as follows: the lungs (n = 27), the urinary tract (n = 14), the central nervous system (n = 4), the abdomen (n = 8), skin and soft tissue (n = 12). The parameters that were identified through univariate analysis to be associated with 28-day mortality were IGS2 score, lactate level, the NLR elevation at H24, H48 and H72. Median NLR levels were significantly higher in non-survivors (n = 35) than survivors (n = 30) at H24 (Median [IQR] 19.02 [8.3–36.3] vs. 8.37 [5.36–13.9], p = 0.005), H48 (17.38 [9.1–34.2] vs. 6.9 [3.7–13.1], p = 0.013) and H72 (12.44 [7.82–21.12] vs. 6.2 [4.7–9], p = 0.008).

**Conclusion:** Increased NLR levels were associated with higher mortality in patients with septic shock. Further investigations are required to confirm these findings.

### P-60 Comparison of automated and semi-quantitative assessment of sublingual microcirculation

#### De Fazio Chiara^1^, De Santis Paolo^2^, Vincent Jean-Louis^2^, Scolletta Sabino^1^, Franchi Federico^1^, Creteur Jacques^2^, Taccone Fabio Silvio^2^

##### ^1^Université de Sienne, Italy; ^2^Hôpital Erasme, Belgium

###### **Correspondence:** De Fazio Chiara - chiara.defazio@student.unife.it

*Annals of Intensive Care* 2018, **8(Suppl 1):**P-60

**Introduction:** The introduction of new handheld microscopes, such as the Incident Dark Field (IDF) tool, allows a better resolution and definition of sublingual microcirculation than older devices, but the analysis of microvascular abnormalities remains cumbersome. Automated softwares could rapidly quantify microcirculation.

**Patients and methods:** From our database of sublingual microcirculatory assessment by IDF (Braedius Medical, The Netherlands), 50 images were randomly selected (July 2017–October 2017). The proportion of small (< 20 µm) perfused vessels (PPV = [number of vessels with continuous flow number of all vessels] × 100) was used to define microcirculatory perfusion. PPV was also determined automatically by the IDF software (CCTools V2.0—Braedius Medical BV, The Netherlands) and compared to the semi-quantitative technique, according to the De Backer score. Vessels falsely detected (false positive = FP) or missed (false negative = FN) by the software were also reported.

**Results:** Median PPV was significantly lower in the semi-quantitative method than in the software analysis (91 [86–95] vs. 98 [95–100]%, p < 0.001). The correlation between the two methods was 0.79 (95% CI 0.66 to 0.88, p < 0.001) + the mean bias was 5% with limits of agreements of 1 to 12%. The FP rate was 2 [1–2]% and the FN rate 16 [12–22]%. Of the missed vessels by the software (FN), 40 [27–50]% of them were capillaries with intermittent or absent flow.

**Conclusion:** The assessment of microcirculatory perfusion using an automated software is significantly correlated with the semi-quantitative measurements, with an agreement within acceptable limits. The proportion of FN vessels remains still high. Future studies assessing microcirculation using software-based analysis should consider these limitations.

### P-61 Prognostic value of venous to arterial carbon dioxide difference in patients with refractory shock

#### Jaoued Oussema^1^, Nouira Hajer^1^, Kallel Mariem^1^, Tilouche Nejla^2^, Sikali Habiba^1^, Elatrous Souheil^1^, Fekih Hassen Mohamed^1^

##### ^1^Mahdia, Tunisia; ^2^EPS Taher Sfar, Mahdia, Tunisia

###### **Correspondence:** Jaoued Oussema - oussamajaoued@hotmail.com

*Annals of Intensive Care* 2018, **8(Suppl 1):**P-61

**Introduction:** The central venous-to-arterial CO2 difference (PcvaCO2) or central PCO2 gap is a marker of tissue hypoperfusion. The PCO2 gap depend on global CO2 production and cardiac output. Some studies used this parameter to guide treatment in patients with shock. The aim of this study was to investigate the prognostic value of central PCO2 gap at admission in patients with acute circulatory failure.

**Patients and methods:** We conducted a prospective study in the medical intensive care unit of Hospital Taher Sfar in Mahdia between March 2013 and April 2016. We included all patients with circulatory failure who required mechanical ventilation and who were monitored by The PICCO2 system. Refractory shock was defined by the need of high doses of catecholamine defined by more than 5 mg/h of norepinephrine. We considered that a central PCO2 gap > 6 mmHg is abnormal. The Central PCO2 gap, APACHE II, hemodynamic and biological parameters were recorded at ICU admission. Patients were classified into two groups: group I-survivors and Group II- non-survivors.

**Results:** Thirty-one patients (age 64 ± 11 and SAPS II score 48 ± 18) were included in this study. The mechanism of circulatory failure was septic shock in 64% of cases, hypovolemic shock in 16 cases and cardiogenic in 19 cases. Mean PCO2 gap was 6 ± 2. Group 1 (survivors) included 9 (29%) patients. There was no significant difference between the two groups regarding sex, comorbidities and APACHE II score. Patients of Group II were significantly older (71 ± 7 vs. 61 ± 11). The septic shock was more frequently observed in non-survivor group (1 patients versus 19 patients, p < 0.001). Nine patients (55%) had a central PCO2 gap > 6 mmHg in Group I versus 13 (41%) in Group II with no significant difference (p = 0.69). In multivariate analysis, PCO2 gap was not associated with mortality. The predictive factors of mortality were septic shock (0R = 2.02 95% IC (2.34–4.35) p = 0.003) and cardiac index (OR 4.45, 95% IC (3.89–8, 94) p = 0.001).

**Conclusion:** In this study, central PCO2 gap was not associated with mortality in patients with refractory shock.

### P-62 Continous heart rate variability monitoring in the ICU-definition of the system and parameters: the ReaSTOC project

#### L’Her Erwan^1^, Librati Audrey^1^, Bodenes Laetitia^1^, Pateau Victoire^1^

##### ^1^CHU de la Cavale Blanche, Brest, France

###### **Correspondence:** L’Her Erwan - erwan.lher@chu-brest.fr

*Annals of Intensive Care* 2018, **8(Suppl 1):**P-62

**Introduction:** The autonomic nervous system (ANS) is highly adaptable and allows the organism to maintain its balance when experiencing stress. Heart rate variability (HRV) is a mean to evaluate cardiac effects of ANS activity and a relation between HRV and outcome has been proposed in various types of patients. We evaluated the feasibility of a automated HRV monitoring, based on standard electrocardiography monitoring, and investigated the different parameters that should be recorded. This project is based on a prospective physiological tracing data-warehousing program (Rea STOC, clinicaltrials.gov # NCT02893462) that aims to record more than 1500 ICU patients over a 3-years period.

**Patients and methods:** Physiological tracings were recorded from the standard monitoring system (Intelliview MP70 Philips), using a dedicated network and extraction software (Synapse v1, LTSI INSERM U1099) that enables simultaneous recording of 5 different physiological curves, at their native resolution (500 Hz for ECG, 125 Hz for other). Raw data were subsequently stored on a dedicated local server, before anonymization and analysis. All consecutive patients were recorded for a 2-hours period during the 24-hours following ICU admission. All measurements were recorded with the patient laying supine, with a 30° bed head angulation. Physiological recordings were associated with metadata collection by a dedicated research assistant. HRV parameters were derived from electrocardiography monitoring using Kubios HRV premium (standard deviation of all normal RR intervals [SDNN], root-mean-square of difference of successive RR intervals [rMSSD], and RR triangular index) and frequency domain (power within low-frequency band [LF], and power within high-frequency band [HF]).

**Results:** For the first interim analysis of the ReaSTOC database, 93 files were analyzed, of which 55 male 38 female (mean age 63 ± 14 yr, survival rate 78%). Most admission were related to respiratory distress and 46 patients were under mechanical ventilation at the time of recordings, 9 patients were hemodynamically unstable. Data are provided within Fig. 1, the figure also illustrates the Poincare plot analysis—important HRV induces high SD1 and SD2 values.

**Fig. 1:**
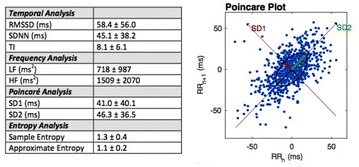
Analysis of ReaSTOC database

**Conclusion:** Routine continuous HRV monitoring is feasible in the ICU. Whether HRV monitoring derived from electrocardiography monitoring has the potential of detect physiological deterioration or response to therapy in ICU patients requires further investigation.

### P-63 Echocardiographic assessment of right ventricular systolic dysfunction during acute respiratory distress syndrome—advantages of the right ventricular longitudinal strain analysis

#### Doyen Denis^1^, Davy Vivien^1^, Robert Alexandre^1^, Hyvernat Hervé^1^, Dellamonica Jean^1^, Bernardin Gilles^1^

##### ^1^CHU L’Archet - Nice, France

###### **Correspondence:** Doyen Denis - doyen.d@chu-nice.fr

*Annals of Intensive Care* 2018, **8(Suppl 1):**P-63

**Introduction:** Acute Cor Pulmonale (ACP) is a frequent complication of acute respiratory distress syndrome (ARDS). It occurs in 22% of cases and might be associated with an increased mortality rate. It is defined by a ratio of telediastolic surfaces of right ventricular (RV) on left ventricular greater than 0.6 and a septal dyskinesia. However, systolic dysfunction defined by the guidelines of the American Society of Echocardiography has not been well studied in ARDS and in particular concerning the RV free wall longitudinal strain (RV-FW-LS). The aims of the present study were to identify the prevalence of RV systolic dysfunction and ACP in ARDS, and to evaluate the effects of inhaled nitric oxide (NOi) and prone positioning.

**Patients and methods:** We prospectively included patients to a mild to severe ARDS, and proceeded to standardization of ventilation and systematic echocardiography in semirecumbent position, with NOi and in prone position. Interpretation of examination was blinded to the investigator. We evaluated the presence of ACP, systolic dysfunction identified by classical cardiologic criteria (RV fractional area change, RV Tei index, Tricuspid annular plane systolic excursion, Velocity of the tricuspid annular systolic motion) and also by RV-FW-LS.

**Results:** Sixteen patients were included. Thirty-seven percent of patients were in severe ARDS. The prevalence of ACP was 25% while right ventricular systolic dysfunction was identified in 37.5% of patients with the classic cardiologic criteria and 68.7% with the impairment of RV-FW-LS which represented the most sensitive test for right ventricular dysfunction detection (Table 1). ACP was not systematically associated to a right ventricular systolic dysfunction (only 68.7% of ACP). RV-FW-LS was significantly improved by NOi (− 15.6 vs. − 18.2 + p = 0.001) and prone positioning (− 15.6 vs. − 23.5 + p = 0.005) in such a way that only 36.3% of patients had a systolic dysfunction in prone position.

**Table 1:** Comparison of strain sensitivity with other echocardiographic right ventricular dysfunction markers

	RV-FW-LS	ACP	RV Tei index	S velocity	TAPSE	RV FAC
Sensitivity	68.7%	25%	25%	26.7%	31.3%	31.3%
		*p* = 0.02	*p* = 0.02	*p* = 0.01	*p* = 0.01	*p* = 0.01

RV-FW-LS: right ventricular free wall longitudinal strain; ACP: acute cor pulmonale; RV Tei index: right ventricular Tei index; S velocity: velocity of the tricuspid annular systolic motion; TAPSE: tricuspid annular plane systolic excursion; RV FAC: right ventricular fractional area change

**Conclusion:** RV systolic dysfunction is much more detected with RV-FW-LS and classical cardiological criteria than with identifying ACP. RV-FW-LS represented the most sensitive test. The impact of morbi-mortality of RV systolic dysfunction screening in ARDS has to be evaluated in larger cohorts.

### P-64 Predictive factors of neurological complications during ECMO support

#### Prezman Pietri Maud^1^, Delmas Clément^1^, Conil Jean-Marie^1^, Bounes Fanny^1^

##### ^1^Hôpital Rangueil, Toulouse, France

###### **Correspondence:** Prezman Pietri Maud - maud.prezmanpietri@hotmail.fr

*Annals of Intensive Care* 2018, **8(Suppl 1):**P-64

**Introduction:** The use of Extra Corporeal Membrane Oxygenation (ECMO) is increasing. Brain complications may occur, resulting in an increased morbidity and mortality. The objective of our study was to analyze the incidence of neurological complications while receiving ECMO, the risk factors, and to describe morbidity and mortality in a large cohort of patients in intensive care unit.

**Patients and methods:** This was an observational, mono-centric, 2-year retrospective study in patients who received ECMO. Primary outcome was the occurrence of neurological complication until D7 after ECMO.

**Results:** One hundred and eight patients were included in the analysis. Twenty-seven patients (25%) presented a neurological complication. Of these, 16 died at D30. There were 13 ischemic sequelae (24.5%), 5 intracranial haemorrhages (9.4%), 4 cerebral edema (7.6%) and one other lesion (1.9%). The median time before occurence of a neurological complication was 8 days after the implementation of ECMO. Multivariate analysis revealed the presence of hyperlactatemia > 5.4 mmol L, neurological deficit at the beginning of the management, as well as the history of stroke before the ECMO implementation as predictive factors of neurological complication (OR 44.15, 95% CI 0.21–3.49).

**Conclusion:** The incidence of neurological complications under ECMO is about 25% and ischemic sequelae are the most frequent. History of stroke and low cerebral flow associated with ischemia–reperfusion seem to increase the occurrence of these complications and must lead to greater vigilance in these patients.

### P-65 Prognostic factors of ECLS for refractory cardiac arrest: experience of a University Hospital

#### Daou Oussama^1^, Piton Gaël^1^, Perrotti Andrea^1^, Winiszewski Hadrien^1^, Pili-Floury Sébastien^1^, Chocron Sidney^1^, Capellier Gilles^1^

##### ^1^CHU Besançon, France

###### **Correspondence:** Daou Oussama - oussama.daou@gmail.com

*Annals of Intensive Care* 2018, **8(Suppl 1):**P-65

**Introduction:** To describe the experience of a university hospital in ECLS implantation for refractory cardiac arrest and to identify prognostic factors associated with a good neurological outcome.

**Patients and methods:** Retrospective analysis of all patients who underwent ECLS for both in-hospital and out of hospital refractory cardiac arrest between February 2010 and March 2017. The primary end point was survival with a good neurological outcome (CPC 1–2) at ICU discharge.

**Results:** 113 patients, aged 55 years [46–63], 47% out-of-hospital cardiac arrest, were included in the analysis. No-flow duration was 0 min [0–3] and low-flow duration until ECLS was 84 min [55–122]. Eighteen patients (16%) survived at ICU discharge with a good neurological outcome. By multivariate logistic regression analysis, female sex, initial shockable rhythm, and pre-ECMO arterial blood pH ≥ 7.1 were independent predictors of survival with good neurological outcome. All of the patients presenting with CPC score of 1 or 2 at ICU discharge had a shockable rhythm and or pH ≥ 7.1 before ECLS implantation. 28% of the patients presenting with these criteria had a good neurological outcome at ICU discharge. All of the patients presenting with non-shockable rhythm and pH < 7.1 before ECLS implantation died in the ICU.

**Conclusion:** About one third of the patients presenting with shockable rhythm and or pH ≥ 7.1 before ECLS implantation had a good neurological outcome at ICU discharge. On the contrary, all of the patients presenting with both non-shockable rhythm and pH < 7.1 before ECLS implantation died in the ICU. These simple parameters might help to identify cardiac arrest patients which could benefit from ECLS implantation.

### P-66 Outcome and quality of life following implantation of left ventricle assist devices (LVAD) in cardiogenic shock patients with multiorgan failure (MOF)

#### Radjou Aguila^1^, Sonneville Romain^1^, Bouadma Lila^1^, Neuville Mathilde^1^, Mariotte Eric^1^, Ruckly Stephane^1^, Magalhaes Eric^1^, Lebut Jordane^1^, Andremont Olivier^1^, Dupuis Claire^1^, Smonig Roland^1^, Lermuzeaud Mathilde^1^, Nataf Patrick^1^, Lepage Laurent^1^, Dorent Richard^1^, Wolff Michel^1^, Mourvillier Bruno^1^, Timsit Jean François^1^

##### ^1^Hopital Bichat, Paris, France

###### **Correspondence:** Radjou Aguila - aguila.radjou@gmail.com

*Annals of Intensive Care* 2018, **8(Suppl 1):**P-66

**Introduction:** Heart transplantation (HT) is considered the “gold standard” therapy for patients with end-stage heart failure. However, only few patients can benefit from HT in emergency setting. We aimed to determine prognosis and quality of life of patients who underwent LVAD implantation with preoperative cardiogenic shock and multiorgan failure.

**Patients and methods:** We performed a retrospective chart review of 37 cardiogenic shock patients with MOF who underwent LVAD implantation, between October 2011 and September 2016. Primary end-point was all-cause mortality 1 year following LVAD implantation. We also analyzed quality of life by phone interviews between February 2017 and April 2017, using EUROQOL and Short-Form General Health Survey (SF-36) scales (scores range from 0 to 100, with higher scores indicating a better health care status). Results are presented as n (%) or median [interquartile range].

**Results:** Thirty-seven patients (age, 60 [45 + 66.5] years, SAPS II, 48 [34 + 62] + SOFA 8 [6–13]) were analyzed. Main cause of heart failure was ischemic cardiomyopathy (n = 18 (49%) patients). Fifteen (40.5%) patients had moderated or severe right heart failure. All patients were mechanically ventilated, 34 (92%) patients received vasopressor inotropic support, 21 (57%) patients received renal replacement therapy, and 22 patients (59%) received venoarterial ECMO support before LVAD implantation. LVAD implantation was performed after 10.5 [5.5 + 16] days of ICU stay. Overall, twenty-one (57%) patients were alive at ICU discharge. Duration of mechanical ventilation and duration of ICU stay were 9 [3.5 − 21] and 32 [22 − 58] days, respectively. Hospital survival and 1-year survival were 57% (21/37) and 54% (20/37), respectively. Seventeen (46%) patients underwent HT 264 [51 − 342] days after LVAD implantation, 2 (5%) patients were still waiting for HT at 1 year, and 2 (5%) patients received LVAD implantation as a destination therapy. The score on the EUROQOL scale was 67.5 [57.5 − 76.3]. Among the SF-36 items, lower score was observed on the role physical (RP) item (12.5 [0 + 50]), whereas higher scores were observed for other items (i.e., Physical Functioning (PF) item, 80 [64 + 95] and Social Functioning (SF) item, 75 [60.1 + 87.5]). Main qualitative complaint in survivors was incapacity to take showers.

**Conclusion:** Implantation of a LVAD in cardiogenic shock patients with MOF is associated with a 54%-survival rate at 1 year, with an acceptable quality of life in most of survivors.

### P-67 Extracorporeal cardiopulmonary resuscitation for refractory in-hospital cardiac arrest

#### Bourcier Simon^1^, Desnos Cyrielle^1^, Rigolet Marina^1^, Schmidt Matthieu^1^, Hékimian Guillaume^1^, Bréchot Nicolas^1^, Coutrot Maxime^1^, Lebreton Guillaume^1^, Besset Sébastien^1^, Nieszkowska Ania^1^, Leprince Pascal^1^, CombesAlain^1^, Luyt Charles-Edouard^1^

##### ^1^Hôpital Cochin, Paris, France

###### **Correspondence:** Bourcier Simon - simon.bourcier@aphp.fr

*Annals of Intensive Care* 2018, **8(Suppl 1):**P-67

**Introduction:** Despite recent improvement in cardiac arrest management, in-hospital cardiac arrest (IHCA) still suffers poor outcome. Whereas its usefulness for out-of-hospital cardiac arrest seems poor, extracorporeal cardiopulmonary resuscitation (e-CPR, i.e. veno-arterial extracorporeal membrane oxygenation (VA-ECMO) under cardiopulmonary resuscitation) could potentially be a life-saving strategy for refractory IHCA, but data are missing. We intended to describe the characteristics and outcomes of refractory IHCA patients treated with e-CPR in our institution over a 10-year period.

**Patients and methods:** Retrospective cohort study of data prospectively collected. All patients implanted with a VA-ECMO for refractory IHCA from 2007 to 2017 were included. VA-ECMO was implanted at the cardiac arrest site by trained cardiac surgeons from our circulatory support mobile unit. After ECMO implantation, patients were all referred and managed in our ICU. Survivors were all contacted by phone 1 year after hospital discharge to specify their vital status and 1-year CPC score.

**Results:** During the study period, 97 patients were treated with e-CPR for refractory IHCA (of cardiac origin for 80.4% of them). VA-ECMO was implanted in our ICU for 37% of them, in the catheterism lab for 19%, in the cardiology department for 11% and in another hospital for 23%. Survival rate at hospital discharge was 19.6%. Data regarding 93 patients are available at 1 year (4 patients are alive but with follow-up < 1 year). Among them, 14 (15%) survived, with a 1-year CPC score of 1 [1–2]. Deaths occurred early (time from ECMO to death, 2 [1–5] days), main causes being multiorgan failure (71%) and post anoxic encephalopathy (12%). Comparisons between 1-yr survivors and non-survivors are shown on Table 1. As compared to 1-yr non-survivors, 1-yr survivors had same no- and low-flow (from CPR start to VA-ECMO depart), their initial rhythm was more frequently shockable (69.2% versus 33.8%, respectively, p = 0.03) and their day-1 SOFA score was significantly lower (13 [10–14] versus 15 [12–17], respectively, p = 0.02).

**Table 1:** Characteristics and outcomes of 97 patients with intra-hospital cardiac arrest

Characteristic	All patients *(n* = *97)*	1-year survivors *(n* = 14)*	1-year non-Survivors *(n* = *79)*	***P***
Age (years)	50.9 (± 14.8)	45 (± 16.3)	51.7(± 14.6)	0.12
Male sex, n (%)	70 (72)	9 (64.3)	57 (72.2)	0.53
Body mass index (kg/m^2^)	26 [22.9–29.3]	26.1 [23.9–27.6]	26 [22.8–29.4]	0.70
McCabe & Jackson score for comorbidity	1 [0–2]	1 [1–2]	1 [0–2]	0.70
SAPS II	79 [65–93]	64 [53–79]	82 [69–94]	*< 0.01*
SOFA score	15 [12–17]	13 [10–14]	15 [12–17]	*0.02*
Cause of CA, n (%)				0.01
Myocardial infarction	41 (42.3)	7 (50)	31 (39.2)	
Acute decompensation of chronic cardiomyopathy	20 (20.6)	1 (7.1)	18 (22.8)	
Myocarditis	6 (6.2)	2 (14.3)	4 (5.1)	
Pulmonary embolism	9 (9.3)	0 (0)	9 (11.4)	
No etiology found	16 (16.5)	1 (7.1)	15 (19)	
Miscellaneous	5 (5)	3 (21)	2 (2.5)	
Shockable rhythm	39 (40)	9 (69.2)	26 (33.8)	*0.03*
Attempted defibrillation	46 (47)	10 (76.9)	32 (41.6)	*0.04*
No flow (min)	0 [0–0]	0 [0–0]	0 [0–0]	0.76
Low flow (min)	43 [30–60]	37 [30–59]	43 [30–76]	0.49
Therapeutic hypothermia	55 (57)	10 (71.4)	42 (53.2)	0.32
ECMO duration, days	3 [1–7]	11 [5.5–18.7]	2 [1–5]	< 0.01
MV duration	3 [1–9.3]	27 [6.2–37.5]	2 [1–7.5]	< 0.01

*4 patients are alive but with a follow-up < 1 year and thus were not included in this analysis

**Conclusion:** The use of e-CPR to treat refractory IHCA is associated with a 15% 1-yr survival rate. Most of deaths occurred during the first days, due to multiorgan failure. Further data are needed to precisely identify factors associated with outcome, in order to select patients who could beneficiate from this technique.

### P-68 Incidence of high lung volume in critical care unit

#### Vallat Thibaud^1^, Heshmati Nassim^2^

##### ^1^CHU de Mulhouse, France; ^2^CHU de Strasbourg, France

###### **Correspondence:** Vallat Thibaud - thibaud.vallat@gmail.com

*Annals of Intensive Care* 2018, **8(Suppl 1):**P-68

**Introduction:** The decrease of lung volume is a keystone for the management of patients under mechanical ventilation in intensive care units. This procedure has not only led to a reduction of morbimortality in ARDS but also in all patients mechanicaly ventilated in intensive care units as well as in major surjery. Nevertheless, the incidence of high volume (VT) on morbimortality is extremely variable (about 8 to 70%). Our main objective is to assess the incidence of high volume ventilation (> 8 ml/kg predicted body weight, PBW) in our hospital intensive care units. Moreover we were interested in determining the risk factors of high volume ventilation.

**Patients and methods:** We conducted a retrospective observational study from January to March 2015 in three intensive care units of a tertiary university hospital. All patients ventilated under sedation in VAC mode during the 24 h after admission were included in the study.

**Results:** Of the 502 patients admissions during the period, 71 one of them (14%) have no height mentioned in their medical file and were exluded. Among the 351 patients considered, 110 (31.3%) were ventilated with high VT (Fig. 1). 98% of patients had a positive expiratory pressure ≥ 4 cmH2O. In multivariable analysis, height (smaller) and weight (lower) are the only associated factors with a high volume ventilation (p < 0.0001 and p = 0.015, respectively).

**Fig. 1:**
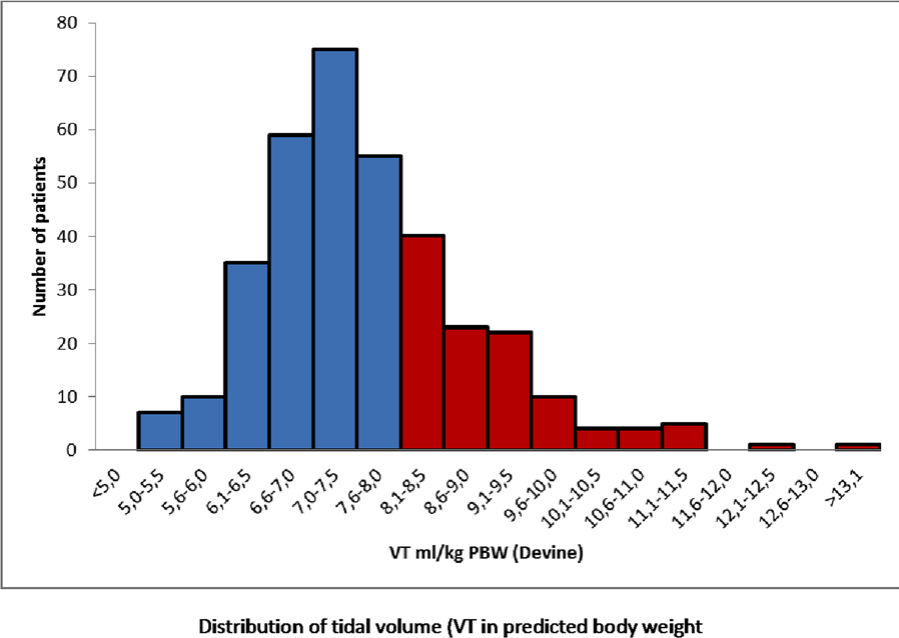
Distribution of tidal volume (VT) in predicted body weight

**Discussion:** The observed incidence on high VT patients is higher than that reported in most papers in literature (Jaber et al. 18%, Hess et al. 8%). Nevertheless, both studies were conducted in operating room with higher VT cut-off (10 ml/kg). Walkey and al showed that 34% of patients in ARDS were ventilated with VT › 8 ml/kg of PBW. Moreover, the same associated factors (smaller height and lower weight) have found in the study. Older studies revealed higher BMI as factor to high volume ventilation. This difference could be explained by the use of predicted body weight.

**Conclusion:** Although the growing literature and the recommandations aim to reduce the lung volume between 6 to 8 ml/kg of PBW, still one third of the patients in intensive care units are ventilated with too high lung volume.

### P-69 Accuracy of automated P0.1 measurements performed by ICU ventilators

#### Beloncle François^1^, Olivier Pierre-Yves^1^, Vuillermoz Alice^1^, Richard Jean-Christophe M.^2^, Piquilloud Lise^3^, Mercat Alain^1^

##### ^1^CHU d’Angers, Angers, France; ^2^Hospitalier Annecy genevois, Annecy, France; ^3^CHU de Vaudois, Lausanne, Suisse

###### **Correspondence:** Beloncle François - francois.beloncle@univ-angers.fr

*Annals of Intensive Care* 2018, **8(Suppl 1):**P-69

**Introduction:** Occlusion pressure at 100 ms (P0.1), defined as the negative pressure measured 100 ms after the initiation of an inspiratory effort performed against a closed respiratory circuit, has been shown to correlate with the respiratory effort and the central respiratory drive. P0.1 measurement is proposed on all modern ventilators. However, the reliability of its automated measurement by the different ICU ventilators has never been studied. This study aimed at assessing the accuracy of the ventilator automated P0.1 measurements.

**Patients and methods:** To simulate spontaneous ventilation, a chamber of a two-chamber Michigan test lung (Michigan Instruments, Grand Rapids, USA) was connected to a driving ventilator set in volume controlled ventilation. The other chamber was connected to the tested ventilator by a double limb circuit with an active humidifier. The compliance and resistance of the lung model were 80 ml cm H_2_O and 5 cmH20 ml s, respectively. Pressure and flow transducers were inserted between the test lung and the ventilator circuit. Signals were acquired using an analog–digital converter (MP150, Biopac Systems^®^, Goleta, USA). Four commercialized ventilators (Evita XL Dräger^®^, Lübeck, Germany, Servo u, Maquet^®^, Solna, Sweden, PB 980, Covidien^®^, Carlsbad, USA, Engstrom Carestation, GE^®^, Madison, USA) were tested in pressure support mode. Each ventilator was assessed for three levels of inspiratory effort (P0.1ref of 2, 6 and 10 cmH20 measured by occluding the test lung with a hermetic plug). Automated P0.1 measurements displayed on the ventilator screen (P0.1vent) were recorded for five different respiratory cycles at each inspiratory effort level.

**Results:** Visual analysis of pressure–time and flow-time curves revealed that airway occlusions were performed during automated P0.1 measurements in all the tested ventilators except in the servo u. P0.1vent values underestimated P0.1ref values in 3 among the 4 tested ventilators (Fig. 1). Variations of P0.1vent correlated well with variations of P0.1ref.

**Fig. 1:**
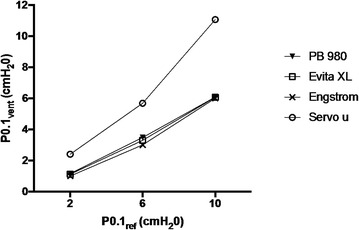
Analysis of ventilator measurements

**Conclusion:** Accuracy of absolute values of P0.1vent varies according to the ventilator model, limiting the use of P0.1 threshold values to help in decision making in clinical practice. However, variations of P0.1vent are reliable in all the tested ventilator.

### P-70 What is the effect on FiO_2_ of mouth closed breathing versus mouth open breathing with nasal cannula? A bench study

#### Bahar Nabila^1^, Machayekhi Shahram^1^, Cuvelier Grégory^2^, Duprez Frédéric^3^

##### ^1^ICU Hornu, Boussu, Belgium; ^2^Ecole Condorcet, Tournai, Belgium; ^3^Baudour, Belgium

###### **Correspondence:** Bahar Nabila - nabila006@hotmail.com

*Annals of Intensive Care* 2018, **8(Suppl 1):**P-70

**Introduction:** Nasal cannula (NC) are used for O2 administration during low and middle hypoxia. Sometimes, the patients receive O2 through NC breathing with their mouth open or mouth closed. During this time, their SpO2 values can vary. The purpose of this bench study was to determine the impact of closed mouth breathing or open mouth breathing on FiO_2_.

**Patients and methods:** The study was conducted on bench with NC connected to a two compartment adult lung model (Dual Test Lung^®^) (DTL) controlled by a Maquet Servo I^®^ventilator. One O2 flow rate (OFR) (4.5 L/min) and three minute ventilation (MV—7, 12, 20 L/min) with Ti Ttot = 0.33 were investigated. FiO_2_ and MV measurements were made using an iWorx^®^ acquisition system (GA207 gas analyzer) and LabScribe II^®^ software. Compliance of DTL was set to—0.07 L cm/H2O and resistance to—5 cmH2O/L/sec. In order to simulate the anatomic naso-buccal area, we have used a T piece connected to a 15 cm long corrugated tube ISO 22 mm (CT22) at the level of inflow of the DTL. The NC was introduced at the entry of the CT22. Three different situations were analyzed—a totally open mouth (TMO), a half open mouth (HOM) and a closed mouth (CM). To simulate HOM, we have used a flow restrictor piece which includes an aperture of 10 mm in the center of this piece.

**Results:** When MV increases, FiO_2_ decreases (p < 0.05). FiO_2_ was highly with a half open mouth regardless MV (p < 0.05). With totally open mouth, the FiO_2_ decreases strongly (p < 0.01). If we consider half open mouth and totally open mouth, the gap of FiO_2_ value decreases with MV increases (Fig. 1).

**Fig. 1:**
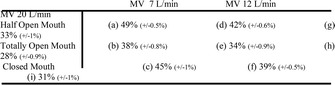
FiO_2_’s obtained for an O_2_ of 4.5 L/min. Means are expressed with SD

**Conclusion:** In oxygen therapy with NC, the FiO_2_ is influenced at the same time by the MV and the opening of the mouth. If the MV increases, the FiO_2_ decreases. In our bench study, the FiO_2_ is lower when the mouth is totally open and higher when the mouth is half open.

### P-71 What is the effect on FiO_2_ when the prongs of the nasal cannula overlap a nostril and the cheek?

#### Mandianga Jean-Michel^1^, Cuvelier Grégory^2^, Machayekhi Shahram^1^, Duprez Frédéric^3^

##### ^1^ICU Hornu, Boussu, Belgium; ^2^Ecole Condorcet, Tournai, Belgium; ^3^Baudour, Belgium

###### **Correspondence:** Mandianga Jean-Michel - mandianga@skynet.be

*Annals of Intensive Care* 2018, **8(Suppl 1):**P-71

**Introduction:** Nasal cannula (NC) are used for O2 administration to treat hypoxia. Sometimes, the prongs of the NC slip on the face and can be found overlapping a nostril and the cheek. Moreover, the patient’s mouth can be open or closed during this time. The purpose of the bench study was to determine the impact of these situations on the FiO_2_.

**Patients and methods:** The study was conducted on bench with NC connected to a two compartment adult lung model (Dual Test Lung^®^) (DTL) controlled by a Maquet Servo I^®^ventilator. One O2 flow rate (OFR—4 L/min) and a four-minute ventilation (MV—7, 10, 14, 17 L/min) with Ti Ttot = 0.33 were investigated. FiO_2_ and MV measurements were made using an iWorx^®^ acquisition system (GA207 gas analyzer) and LabScribe II^®^ software. Compliance of DTL was set to—0.07 L/cmH2O and resistance to—5 cmH2O/L/sec. In order to simulate the anatomic naso-buccal area, we used a T piece connected to a 15 cm long corrugated tube ISO 22 mm (CT22) at the level of inflow of the DTL. To simulate closed mouth, we blocked an extremity of T piece, while the open mouth, was simulated by remove this obstruction. Four different approaches were analyzed—a closed mouth (CM) with either a NC overlap on one nostril or not. A totally open mouth (TMO) with either a NC overlap on one nostril or not.

**Results:** When the MV increases, the FiO_2_ decreases. When the mouth opens, the FiO_2_ decreases. When the prongs are overlapping one nostril the FiO_2_ decreases slightly (mean 5 ± 2% in absolute value). Statistical differences were found between closed and open mouth and between overlap on one nostril and not (p < .05), except between TMO and CM at two MV (14 and 17 L/min) when NC overlap on one nostril (Fig. 1).

**Fig. 1:**
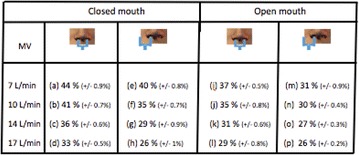
FiO_2_ differences between closed mouth and open mouth when the prongs are overlapping one nostril (O_2_ flow 4 L/min). Means are expressed with SD

**Conclusion:** When the prongs of NC are not correctly placed in the nostrils, the FiO_2_ decreases, but this impact is limited in our bench study. The impact of MV increases and mouth opening on the FiO_2_ values is also important.

### P-72 Surgical tracheotomy in neuro-reanimation

#### Naili Amine^1^

##### ^1^Blida Rp, Algeria

###### **Correspondence:** Naili Amine - drnailiamine@yahoo.fr

*Annals of Intensive Care* 2018, **8(Suppl 1):**P-72

**Introduction:** The weaning of mechanical ventilation is an essential and delicate phase in the management of a resuscitation patient. The neurosurgical patient presents a number of specific problems, such as impaired control ventilatory control, coughing or the pharyngo-laryngeal intersection. However, it often allows short-term ventilatory withdrawal in the neurosurgical patient, probably largely by the simple fact that it authorizes the definitive cessation of sedation. The objective of the study and demonstrate the place of tracheotomy in neuro-resuscitation patients, and prevent its complications.

**Patients and methods:** A retrospective descriptive study of 597 patients hospitalized in the neuro-resuscitation unit during the period 1 January to 31 December 2016, of which 113 patients benefited from surgical tracheotomy, is a frequency of 19% of all inpatients during this period. Clinical, para-clinical, etiological, and therapeutic data were collected from hospitalization records.

**Results:** In a series of 597 hospitalized patients, during the defined period, 113 patients had surgical tracheotomy, a frequency of 19%, in the literature two studies or the data were extremely variable, with 29% in the study Namen versus 2.9% in the Coplin study. Of the 113 tracheotomies, 6 were performed by neurosurgeons, and 107 by resuscitators at a frequency of 95%. The tracheotomy was performed on average 7 days after the intubation of the patients, after verification of the impossibility of the extubation of the latter either for central affection of the ventilatory controls, or reached the mixed nerves and disorders of the laryngo-pharyngeal intersection and according to expert recommendations in 2017—Tracheotomy should not be performed in the intensive care unit before the fourth day of mechanical ventilation. Different pathologies that patients suffered and required tracheotomy were: post-operative complications of brain tumors (brain stem and mixed nerves) with 52 patients, a rate of 46%, vascular pathologies (stroke and CVT)), with 32 patients (28%), traumatic pathologies, with 29 patients (26%). 21 cases, 19%, 4 cases of secondary bleeding of the orifice, 2 cases of tracheal stenosis, and 1 case of tracheomalacia. The decan made after pharyngolaryngeal neurological examination, and according to SFAR 2017 recommendations experts suggest that a multidisciplinary decanulation protocol available in resuscitation departments.

**Conclusion:** Tracheotomy in neuro-resuscitation has its place, especially in view of the different complications specific to this type of patient, but no study has demonstrated its improvement in vital prognosis. Post-tracheotomy complications can be considerably reduced if the protocols and expert recommendations are applied.

### P-73 Ultasonographic detection of patient-ventilator asynchronies during non-invasive ventilation. A study in healthy volunteers

#### Vivier Emmanuel^1^, Carteaux Guillaume^2^, Haudebourg Anne Fleur^2^, Mekontso Dessap Armand^2^

##### ^1^CH saint Joseph, Lyon, France; ^2^CHU Henri Mondor, Créteil, France

###### **Correspondence:** Vivier Emmanuel - evivier@hotmail.com

*Annals of Intensive Care* 2018, **8(Suppl 1):**P-73

**Introduction:** Noninvasive ventilation (NIV) in intensive care (ICU) is associated with the occurrence of frequent asynchronies related to the leaks around the interface, mainly auto-triggering and delayed cycling. Their detection requires a respiratory muscles activity monitoring. Diaphragmatic ultrasonography is a simple imaging technique available at bedside to assess diaphragm motion. Whether diaphragmatic ultrasonography would allow detecting asynchronies due to leaks during NIV is unknown. The aim of this study was to assess two methods of diaphragmatic ultrasonography (excursion and thickening), coupled with the airway pressure signal to detect patient-ventilator asynchronies during NIV.

**Patients and methods:** Nine healthy subjects were placed under NIV and subjected to intentional inspiratory and expiratory leakage on the ventilator circuit to generate delayed cycling and auto-triggering, respectively. The flow, airway pressure and diaphragmatic electromyogram were collected in order to identify the asynchronies generated by the leaks. In the meantime, an ultrasound recording of the excursion of the right diaphragm and of the thickening of the right diaphragmatic zone of apposition were performed and combined with the display of airway pressure on the ultrasound screen. These records were analyzed a posteriori to define the diagnostic performance [including sensitivity (Se), specificity (Spe), positive predictive value (Ppv), and negative predictive value (Npv)] of the excursion and the thickening to detect asynchronies.

**Results:** The experimental setup generated a median of 34 asynchronies per subject (interquartile range 24–44). Auto-triggering was correctly identified by continuous recording of both excursion (Se = 93%, Spe = 99%, Ppv = 93%, and Npv = 99%, Fig. 1A) and thickening (Se = 94%, Spe = 99%, Ppv = 95%, Npv = 99% + Fig. 1C). Delayed cycling was detected with a slightly lower performance by diaphragm excursion (Se = 85%, Spe = 98%, Ppv = 84%, Npv = 98% + Fig. 1B) and thickening (Se = 89%, Spe = 98%, Ppv = 86%, Npv = 99% + Fig D).

**Fig. 1:**
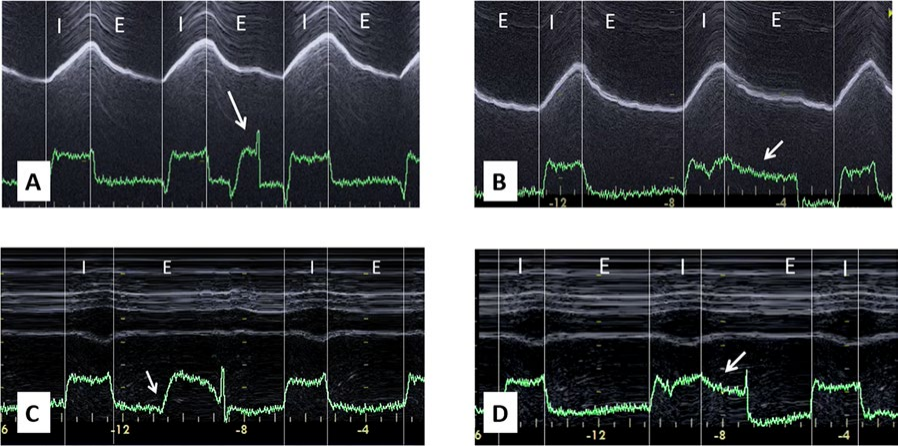
Diaphragmatic ultrasonography results

**Discussion:** These encouraging results may be tempered by a variable effectiveness of the technique from one subject to another, in particular concerning the excursion. Moreover, their generalization to critically ill patients may depend on several factors including echogenicity, stability and amplitude of the ultrasound signal in this population.

**Conclusion:** Ultrasound is a simple clinical tool available at the bedside to detect delayed cycling and auto-triggering associated with NIV leaks, provided that the airway pressure curve is displayed on the screen of the ultrasound machine. Further studies are needed to assess its usefulness in detecting other types of asynchronies and its feasibility in critically-ill patients.

### P-74 Single center experience of ECCO2R

#### Winiszewski Hadrien^1^, Aptel Francois^1^, Piton Gaël^1^, Capellier Gilles^1^

##### ^1^CHRU Besançon, Besancon, France

###### **Correspondence:** Winiszewski Hadrien - hadrien51@hotmail.com

*Annals of Intensive Care* 2018, **8(Suppl 1):**P-74

**Introduction:** Although extra-corporeal CO2 removal (ECCO2R) is not recommended, strong rational supports the concept. We aimed to describe our single-center experience of ECCO2R in the setting of mild to moderate acute respiratory distress syndrome (ARDS) and chronic obstructive pulmonary disease (COPD).

**Patients and methods:** We performed a retrospective case note review of patients admitted to our tertiary regional intensive care unit (ICU) and commenced on ECCO2R from November 2015 to August 2017. Demographic data, physiologic data (including pH and partial pressure of carbon dioxide in arterial blood [PaCO2]) before ECCO2R starting, and at day 1 were recorded.

**Results:** Twenty one patients received ECCO2R. Thirteen were managed with Hemolung^®^ device, seven with Prismalung^®^ and one with ILA^®^. Indication for ECCO2R were COPD exacerbation (n = 11), mild to moderate ARDS (n = 8), uncontrolled hypercapnia due to pneumonia (n = 1), and hypercapnia due to bronchial compression by mediastinal adenopathy (n = 1). Before starting ECCO2R, median minute ventilation, pH and PaCO2 were respectively 7.2 [6.5, 8.5] L/min, 7.32 [7.23, 7.34] and 8.3 [7.2, 9.8] kPa. After 24 h of treatment, 15 patients were in pressure support ventilation mode. At that time, median minute ventilation, pH and PaCO2 were respectively 8.9 [7.9, 10] L/min, 7.44 [7.40, 7.46] and 5.2 [4.5, 6.9] kPa. Median duration of ECCO2R treatment was 7 days [5, 10]. Prone positioning was performed in 10 patients, without any complications. Four of 11 patients receiving ECCO2R for COPD exacerbation were extubated during extra-corporeal treatment. Device thrombosis occurred in 3 patients, all receiving treatment by Hemolung^®^. Hemolysis was documented in 10 patients treated by Hemolung^®^, and one patient treated by Prismalung^®^. Seventeen patients were discharged from ICU alive.

**Discussion:** Unexpected increase of minute ventilation after beginning of ECCO2R therapy may be related to early weaning of sedation, and pressure support ventilation mode.

**Conclusion:** Our observational cohort shows that ECCO2R therapy is effective to reduce PaCO2 and improve pH in the settings of mild ARDS and COPD exacerbation. However, early weaning of sedation and pressure support ventilation might limit the decrease of respiratory rate and tidal volume.

### P-75 Clinical spectrum of patients with diaphragmatic paralysis and relationship between age and right hemi-diaphragm motion in Duchenne muscular dystrophy

#### Fayssoil Abdallah^1^, Chauffaut Cendrine^2^, Lamothe Laure^1^, Meng Paris^1^, Ogna Adam^1^, Stojkovic Tanya^3^, Mompoint Dominique^1^, Prigent Helene^1^, Clair Bernard^1^, Behin Anthony^3^, Laforet Pascal^1^, Bassez^3^, Guillaume, Carlier Robert^1^, Orlikowski David^1^, Chevret Sylvie^2^, Eymard Bruno^3^, Lofaso Frederic^1^, Annane Djillali^1^

##### ^1^CHU Raymond Poincaré, Garches, France; ^2^SBIM Hopital Saint Louis, Paris, France; ^3^CHU Pitié Salpetriere, Paris, France

###### **Correspondence:** Fayssoil Abdallah - fayssoil2000@yahoo.fr

*Annals of Intensive Care* 2018, **8(Suppl 1):**P-75

**Introduction:** Duchenne muscular dystrophy (DMD) is an X-linked recessive genetic disorder, caused by mutations in the DMD gene. Respiratory failure is classical in the natural history of this disease. Little is known about the diaphragm echographic pattern and the spectrum of patients with diaphragmatic paralysis in this disease. We aimed to assess the relationship between age and diaphragmatic motion and thickening fraction (TF) and to characterize the spectrum of patients with diaphragmatic paralysis.

**Patients and methods:** We included retrospectively DMD patients who experienced diaphragmatic echography and spirometry in our institution. Diaphragmatic paralysis was defined as a diaphragm with TF < 20%.

**Results:** 38 DMD patients were included in this study. All DMD patients were wheelchair bound. DMD patients had severe respiratory insufficiency with a median VC at 11% of predicted value [8–19]. 87.5% of patients were on home mechanical ventilation (HMV) and 28% were invasively ventilated. Right diaphragmatic motion at deep inspiration was severely altered with a median of 8.4 mm [4–13]. Right TF of the diaphragm was severely altered with a median of 13.3% [7.7–27.2]. 66.6% of patients disclosed a paralyzed diaphragm pattern with a right TF < 20%. The age was inversely correlated with TF of the diaphragm (r = -0.56, p 0.016) and with the right diaphragm inspiration motion (r = − 0.68 p < 0.0001). Patients with diaphragm paralysis were older with median age at 32.5 years [31–39.5], with severe respiratory impairment (median sitting CV = 6%) and median cumulated annual HMV duration at 12.5 years.

**Conclusion:** In DMD, age is inversely correlated with diaphragm function. Diaphragm paralysis is frequent in older adult non-ambulant DMD.

### P-76 A model to predict short-term prognosis of acute exacerbation of COPD patients admitted to a Tunisian ICU

#### Ouanes Islem^1^, Bouker Nouha^1^, Tlili Meriem^1^, Hammouda Zaineb^1^, Hraiech Kmar^1^, Zorgati Hend^1^, Boukadida Sana^1^, Nouira Wiem^1^, Ouni Amal^1^, Dachraoui Fahmi^1^, Abroug Fekri^1^, Ouanes-Besbes Lamia^1^

##### ^1^CHU Fattouma Bourguiba, Monastir, Tunisia

###### **Correspondence:** Ouanes Islem - ouanes.islem@gmail.com

*Annals of Intensive Care* 2018, **8(Suppl 1):**P-76

**Introduction:** AECOPD is a major cause of ICU admission. Short-term prognosis has been substantially improved in the recent years but a number of patients are frequently readmitted. The phenotype of acute exacerbation leading to death in ICU and the impact of exacerbation frequency is not well studied. The aim of the present study is to identify independent risk factors and to develop a probability model predicting ICU death in a cohort of AECOPD admitted to ICU.

**Patients and methods:** In a prospectively collected database including consecutive patients admitted between 2000 and 2015 for AECOPD. We compared patient’s episodes characteristics according to the end of ICU stay outcome. Statistics—We developed a multivariable model for ICU death. Our assessment of model performance was goodness of fit as evaluated by the Hosmer–Lemeshow test (p > 0.05 indicate better fit). We assessed discrimination using the area under the curve (AUC) of the receiver operating characteristics (ROC) curve.

**Results:** During the study period 662 episodes fulfilled criteria of COPD exacerbation in 498 patients, 161 of them were admitted more than one time (median = 3, IQR 2–4). The rate of ICU death was 12.2% (81 patients). In univariate comparisons, parameters with a p-value < 0.2, were: age, cardiovascular comorbidities, SAPS II score, first ventilatory mode, NIV failure, VAP occurrence and the following ABG parameters at ICU admission—pH, HCO3—and PaO2. In multivariate logistic regression, parameters independently related to ICU death were: cardiovascular comorbidities (OR 2.21 + 95% CI 1.10–4.44), intubation at admission (OR 79.3 + 95% CI 10.4–604.2), NIV failure (OR 200 + 95% CI 26.3–1000), VAP occurrence (OR 11.5, 95% CI 5.46–23.8), pH at ICU admission (OR 0.24 per 0.01 decrease), 95 CI 0.01–8.77) and HCO3—at ICU admission (OR 0.93 per 1 mmol/l increase + 95% CI 0.88–0.98). The final probability model included the previous identified factors in addition to age and SAPS II score, this model exhibited good calibration (Hosmer–Lemeshow X^2^, p = .98) and good discrimination (ROC-AUC, 0.94 + 95% confidence interval, 0.92–0.96) (Fig. 1).

**Fig. 1:**
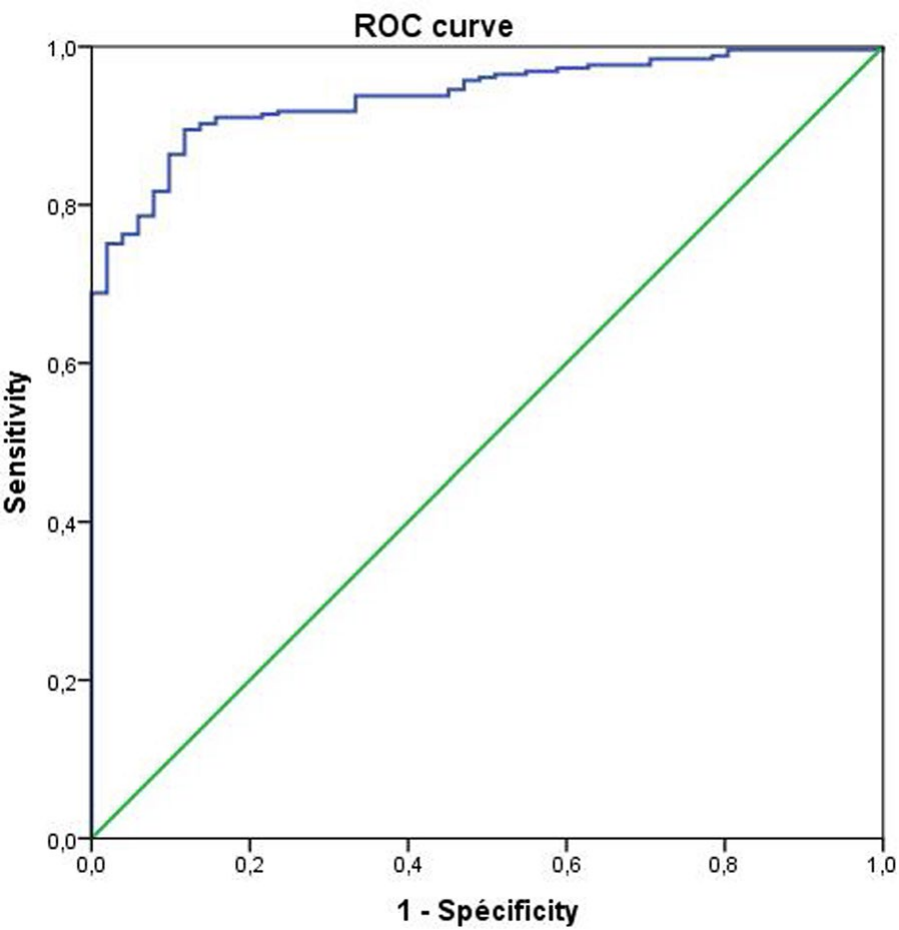
ROC curve

**Conclusion:** Our cohort study identified risk factors of ICU death, mainly collected at admission among patients with AECOPD. The proposed probability model has a good performance in predicting the short-term prognosis. Further evaluation in other cohorts is needed.

### P-77 Prevalence and distribution of precarity features of patient admitted to an ICU located in a high poverty rate territory: a pilot study from the Saint-Denis general hospital

#### Benais Morgan^1^, Preda Gabriel^1^, Ferreira Luis^1^, Laine Laurent^1^, Memain Nathalie^1^, Verdiere Bruno^1^, Da Silva Daniel^1^, De Montmollin Etienne^1^

##### ^1^Hôpital Delafontaine, Saint Denis, France

###### **Correspondence:** Benais Morgan - morgan.benais@gmail.com

*Annals of Intensive Care* 2018, **8(Suppl 1):**P-77

**Introduction:** Precarity is a complex notion including several components, and its definition is still debated. It is more subtle than financial poverty alone, and can increase population’s health insecurity. We hypothesize that patients with precarity features may have different epidemiologic characteristics and ICU outcomes than the general population. The aim of this study was to describe precarity features and outcomes of patients admitted to an ICU located in a high poverty rate territory.

**Patients and methods:** We conducted a prospective single-center observational study of all patients admitted to ICU of the Saint-Denis general hospital, from February to July 2017. Precarity features were classified in 4 categories—absence of health insurance, lack of incomes or minimum allowances, homelessness or social home or hotel, and social isolation (no social link, or associations or neighbors). Others social data were collected (speaking fluent French and education level) as well as usual clinical ICU data.

**Results:** Among 241 patients included, precarity features were found in 83 patients (35.3%). Income precarity was the most common, followed by accommodation precarity, health precarity and relation precarity (Table 1). Precarity was not associated with hospital mortality (37 vs. 25.7%, p = 0.20). All types of precarity were significantly associated with each other. Precarious patients were younger (63 vs. 40 years-old, p < .01) and had less comorbidities. We found no differences concerning hospital or ICU length of stay (12 vs. 11 days, p = 0.53) or concerning education level between precarious patients and the others.

**Table 1:** Precarity features

Variable—n (%) or median [IQR]	Total n = 241
Clinical data	
Age	56.4 [35–69.7]
Male sex	135 (66)
Body Mass Index	24.5 [21.5–28.7]
SAPS II	39 [22–52]
Precarity features	
A least one precarity feature	83 (35.3)
Income precarity	64 (27.4)
Accommodation precarity	23 (9.6)
Health precarity	22 (9.2)
Relation precarity	19 (7.9)
Number of precarity features	
0	152 (65.2)
1	51 (21.9)
2	20 (8.6)
3	5 (2.1)
4	5 (2.1)
Other social features	
Neither speaks nor understands French	23 (9.6)
Low education level	164 (79.6)

**Conclusion:** Our pilot study shows that precarity features are indeed very frequent and are often cumulated. With respect to the small patient sample, precarity does not seems to be associated with hospital mortality or length of stay. Further investigations with larger patient samples and multicenter designs are warranted to investigate properly the impact of precarity on ICU management and outcomes.

### P-78 Determinants of intra-hospital mortality of patients over 85 years hospitalized in intensive care unit—experience of a general hospital in a Paris suburban area

#### Andre Valentin^1^, Ayed Soufia^1^, Barchazs Jean^1^, Bouguerba Abdelaziz^1^, Boukary Madgid^1^, Yaacoubi Sondes^1^, Bornstain Caroline^1^, Vincent François^1^

##### ^1^Groupe Hospitalier Intercommunal Le Raincy Montfermeil, Montfermeil, France

###### **Correspondence:** Andre Valentin - valentinandre90@gmail.com

*Annals of Intensive Care* 2018, **8(Suppl 1):**P-78

**Introduction:** Few data are available on very–very old patients admitted in the intensive care units (ICU). We report our experience.

**Patients and methods:** Patients aged ≥ 85 admitted into ICU between 1st August 2011 and 1st August, 2017, were included. We collected data about year of admission, place of residence, provenance, SAPS II, type of patient, presence of cancer or orthopedic emergency, organ support in ICU, final diagnosis in ICU, location of discharge, and intra-hospital mortality (IHM). IHM was the variable of interest. Results are reported as medians and interquartile ranges or percentages. Categorical variables were compared using the Chi square test and Fisher’s exact test when appropriate, and continuous variables were compared using the nonparametric Wilcoxon test or the Mann–Whitney test.

**Results:** 325 patients were included (10% of ICU admissions, age—87.7 [86.4–89.6], 74 ≥ 90 years, 55.7% males, SAPS II, 47[38–66], 74.8% living at home before hospital admission, 84% medical). Patients were referred from emergencies (37%) or wards, except geriatrics (39.2%, geriatrics: 1.9%). Main final in-ICU diagnoses were: acute respiratory failure of COPD (16.6%) or *de novo* (14.2%), septic shock (13.5%), pulmonary edema (12.3%), and digestive disease, mainly bleeding (10.5%). Patients were admitted for surveillance without organ support in 22.2%. Decision on withholding or withdrawing of cares (WWC) was decided in the ICU for 78. 89.7% died in the hospital. Among all patients, 111 (34.2%) died in the ICU and 175 (53.8%) in the hospital (post ICU deaths: 36.6%). IHM was not higher among those over 90 years than those younger (p = 0.74). 156 (72.9%) were discharged to general or specialized wards other than geriatrics (24). In multivariate analysis, if age is forced, vasopressors and SAPS II were predictors of IHM. When forced in the model, very old age was not associated with outcome and did not modified the model. If WWC (confusing factor) was not included, only SAPS II and use of vasopressors were predictive of IHM. The age still does not come out even if forced. If we include the type of patient in the model, emergency surgery seems protective (p = 0.009 vs. medical or elective surgery).

**Conclusion:** In our experience only SAPS II and use of vasopressors in the ICU can predict IHM in VVOP. We cannot determine if discharge to geriatrics was beneficial.

### P-79 Management of very elderly people in ICU over 14 years: a retrospective study

#### Danin Pierre-Eric^1^, Carreira Serge^2^, Fosse Jean-Philippe^3^, Hyvernat Hervé^1^

##### ^1^CHU de Nice, France; ^2^Hôpital Sainte Camille, Bry Marne, France; ^3^Clinique Les Sources, Nice, France

###### **Correspondence:** Danin Pierre-Eric - danin.pe@chu-nice.fr

*Annals of Intensive Care* 2018, **8(Suppl 1):**P-79

**Introduction:** Population aging is a global and expanding phenomenon. Elderly people are particularly vulnerable, and often need health care. This demographic evolution also affects Intensive Care Units, and 80 years old patient are now frequently admitted—it corresponds to 15% of admission in France. Indeed we have analyzed the change in management of this very elderly people (80 years old and more) over the past 14 years in a French medical intensive care unit in a provincial University Hospital.

**Patients and methods:** A retrospective cohort study was conducted using medical intensive care unit registry for demographic, physiological and diagnostic data from January 2003 to December 2016. Characteristics and treatment intensity during medical ICU stay were specified, and short term and long term mortality were also recorded.

**Results:** A total of 6608 admissions, including 689 octogenarians and older, were registered during the period. The proportion of very elderly people gradually increase from 4 to 11%. Intensity of treatment (organ support) increased from 1.14 from 1.31 per patient between the primary and the second part of the period, notably linked to mechanical ventilation (56 vs. 66%, p < 0.01) and vasopressor infusion (35 vs. 46%, p < 0.01). Even if severity score increased (SAPS 2 increase from 52.7 to 60.5, p < 0.01), the ICU mortality remains constant (34 vs. 32.8%). However, we were surprised to observe an increase in 1 year mortality (62 to 74%, p < 0.01).

**Conclusion:** Between 2003 and 2016, proportion of admission of very elderly people has increased two fold in our ICU. Although treatment intensity increases for more severe patients, ICU mortality remains the same. Nevertheless, absence of beneficial effect after 1 year remains questioning. Could ICU to ward transfers and care course after hospital be optimized?

### P-80 The fate of very old patients in the ICU

#### Michel Philippe^1^, Gelée Bruno^1^, Plantefève Gaétan^2^, Fadel Fouad^1^, Ehrmann Stephan^3^

##### ^1^CHRD, Pontoise, France; ^2^CH Victor Dupouy, Argenteuil, France; ^3^CHU de Tours, France

###### **Correspondence:** Michel Philippe - philippemich@gmail.com

*Annals of Intensive Care* 2018, **8(Suppl 1):**P-80

**Introduction:** Very old patients (over 85 years) are increasingly present in french intensive care units (ICU). However the potential benefit for patients in terms of recovery and discharge versus delayed death in the ICU environment remains debated. We aimed to describe their stay in the ICU and the mortality in the unit.

**Patients and methods:** Multicentric observational study. All patients over 85 years old hospitalized in the ICU or attendant intermediate care units were included in seven French participating centers from 2015 to 2016.

**Results:** Of the 239 patients included, 48 (20%) were over 89 years. 62 (25%) were admit exclusively to intermediate care units. 107 (41%) lived alone at home, 40% in couple or with ther family, 21 (9%) in retirement home and 16 (7%) in nursing home. 40 (17%) had a simplified ALD score lower than 4 indicating good functional independence. The more frequent diagnosis were acute pulmonary oedema and exacerbated COPD. The mean simplified acute physiology score (SAPS II) was 53 ± 21. The treatment were were invasive mechanical ventilation 84 (36%), only with noninvasive ventilation 50 (21%), vasopressor agents 79 (33%) and 4 (2%) with renal replacement therapy. The average length of stay was 5.9 ± 6.1 days. After adjustment on SAPSII (without age), those invasive treatments were not associated with mortality no more than age. Global mortality rate was 28%. 53 (22%) were subject of a procedure for limiting therapeutics, among which 35 (66%) died in the unit versus 17% for the other patients. The decision of therapeutic limitation was associated with severity of illness as measured by the SAPSII (p = 0.01) but not with age. Frequency of therapeutic limitation were similar in ICU and intermediate care units.

**Discussion:** The mortality rate is lower than the older studies (S de Rooij 2005—38%). Unlike the study of P. Biston (2013) which covers only the most serious cases, the mortality for any type of gravity remains reasonable. The procedure for limiting care were frequent especially for the most severe pathologies but all the patients who a decision of limiting care were stated were not dead.

**Conclusion:** The patients over 85 years old admit in french ICU are very chosen. Any major treatment appear to enhance mortality.

### P-81 Clinical characteristics and long-term outcomes of elderly survivors of intensive care unit (ICU)

#### Ben Saida Imen^1^, Ennouri Emna^1^, Zarrougui Wafa^1^, Sma Nesrine^1^, Limam Manel^1^, Fraj Nesrine^1^, Ayachi Jihene^1^, Khedher Ahmed^1^, Azouzi Abdelbaki^1^, Boussarsar Mohamed^1^

##### ^1^Farhat Hached University Hospital, Sousse, Tunisia

###### **Correspondence:** Ben Saida Imen - imen.bensaida@yahoo.com

*Annals of Intensive Care* 2018, **8(Suppl 1):**P-81

**Introduction:** Due to advancements in medical technology and management of illnesses, an increasing proportion of critically ill patients are elderly. Few information is available on the prognosis of these patients after ICU discharge. The aim of this study was to analyze the clinical characteristics and long-term outcomes of elderly admitted to ICU.

**Patients and methods:** Monocentric, observational prospective study was performed. All elderly survivors (aged ≥ 65 years) after an ICU stay in a medical Tunisian ICU between January 2014 and December 2015 were included. Data collected were: clinical features at admission, acute management procedures, functional characteristics and vital parameters (Blood Pressure, Heart Rate, ABG’s) at ICU discharge. Patients were followed during 1 year via phone calls. A multivariate regression analysis was used to identify risk factors for one-year mortality.

**Results:** During the two-years study period, 102 elderly patients were discharged alive. 60 (58.8%) were male. Clinical features of elderly survivors were: Mean age, 74.9 ± 6.54 years, median of Charlson index, 2 [0–7], Chronic respiratory disease, 70 (68.6%), hypertension, 46 (45.1%) and diabetes 39 (38.2%). The most common reason for admission was acute respiratory failure in 80 (78.43%) patients and mean SAPS II was 34.9 ± 9.7. 45 (44.1%) patients required invasive ventilatory support, 43 (42.2%) vasoactive drugs and 3 (2.9%) received renal replacement therapy. The median of ICU length of stay was 6 [1–64] days. The follow up was possible for 80 (78.4%) patients. Mortality rate at 1 year was 37.5%. Predictors of one-year fatal outcome in univariate analysis were as follows—SAPS II (p = 0.009), Heart rate at discharge (p = 0.039), decline in functional status (p = 0.000), World health organization (WHO) performance status at discharge (p = 0.000) and readmission within 1 month (p = 0.000). Multivariate regression showed that SAPS II (OR, 1.11 + 95% CI [1.02–1.20] + p = 0.01), WHO Performance status at discharge (OR, 3.64 + 95% CI [1.92–6.9] + p = 0.000) and heart rate (OR, 1.06 + 95% CI [1.01–1.12] + p = 0.01) were independent risk factors of one-year mortality.

**Conclusion:** This study suggests that age and comorbidities should not be exclusion criteria for ICU admission. In the long-term only SAPS II, performance status and heart rate were significantly associated with one-year mortality in the elderly ICU survivors.

### P-82 Triage and admissions at hospital resuscitation room

#### Ghomari Wahiba Imene^1^, Boumlik Reda^1^, Bradai Senouci^1^

##### ^1^CHU Abdelkader Hassani, Sidi Bel Abbes, Algeria

###### **Correspondence:** Ghomari Wahiba Imene - ghomari_wahiba@yahoo.fr

*Annals of Intensive Care* 2018, **8(Suppl 1):**P-82

**Introduction:** Triage is an act performed at the entrance of emergency departments (ED’s), it allows the classification of patients in different categories according to the seriousness and the priorities of treatment. Vital emergencies are geared towards resuscitation room. In our ED, triage is not codified and is «done» in most cases by an unqualified staff. The aim of this work is to show the impact of absence of triage on the functionning of the resuscitation room.

**Patients and methods:** It’s a prospective study, conducted in the ED of a university hospital, over 6 months, including all patients over 15 years old, admitted at the resuscitation room. Epidemiological and clinical data of patients, their CCMU classification (Classification Clinique des Malades aux Urgences) have been specified, as well as their outcomes.

**Results:** We collected 366 patients. The average age was 51.24 years old (15–97 years), for a sex ratio of 1.57. Forty patients (10.1%) arrived «standing» at the ED. Patients CCMU 1 and 2 represented 41.3% of these admissions. The systolic blood pressure was under 90 mmHg in 25% of cases, the Glasgow coma scale < 8.15 in 18% of cases, and the SpO2 < 90% in 23% of cases. Mortality was 5.5%. The other patients were admitted at the intensive care unit (36%), at the short stay hospitalization unit (33.6%), at the operating room (13.4%), or transferred to other departments (9.2%).

**Discussion:** The patients CCMU 1 and 2 arrived by ambulance, «lying» , were considered as severe. The proximity of the resuscitation room of consultation rooms allows it to be used sometimes in flows’ management and as a place of triage. The patients transferred straight to services didn’t show signs of vital distress motiving their initial admission at resuscitation room or even at ED. Those admitted at the short stay hospitalization unit were steady, but needed complementary examinations, specialized expert advice, or were waiting for a downstream bed.

**Conclusion:** A triage system must be introduced at the entrance of our emergency departments. The staff involved in that sorting must be identified, and disposing of a triage scale in order to figure out the degree of priority associated to patients conditions, and direct the ones needing urgent care towards the resuscitation room.

### P-83 Why oncology patients come to emergency department near the end of life?

#### Ghomari Wahiba Imene^1^, Boumlik Reda^2^

##### ^1^Sidi Bel Abbes, Algérie; ^2^CHU Abdelkader Hassani, Sidi Bel Abbes, Algérie

###### **Correspondence:** Ghomari Wahiba Imene - ghomari_wahiba@yahoo.fr

*Annals of Intensive Care* 2018, **8(Suppl 1):**P-83

**Introduction:** Beside patients presenting real emergency, emergency departments (ED’s) receive oncology patients near the end of life, developing different symptoms. However, taking care of these patients collides with constraints of an emergency department, related to time and space.

**Patients and methods:** This survey, over 18 months, concerned patients with terminal neoplasic diseases, admitted at the resuscitation room of an ED in university hospital. Patients under 15 years old have been excluded.

**Results:** One hundred patients were included, with average age of 59.5 years old (25–84) and sex ratio of 1.12. These patients were brought to emergencies by their family in 83% of cases. Reasons for admissions were varied, severe deterioration of their general condition (41%), alteration of consciousness (28%), respiratory distress (13%), convulsive seizures (12%). Therapeutic interventions were cardio-pulmonary resuscitation (2%), fluid volume expansion (91%), mechanical ventilation (15%), administration of vasopressors (23%) and anticonvulsants (12%). Mortality at the resuscitation room was 13%. Thirty eight patients were admitted at the intensive care unit, equally at the short stay hospitalization unit (SSU) of ED. Two patients returned home at the request of their family.

**Discussion:** These results show that ED’s remain the last resort in front of oncology patient who is deteriorating, the occurrence of complications, and sometimes, the psychological exhaustion or family’s obstinacy.

**Conclusion:** Emergencies departments continue admitting patients with terminal cancer, but are not organized for medium and long term care. The creation of a palliative care unit and the organization of home-based care will allow the prevention and treatment of complications as well as a psychological care, thus improving the living quality of these patients and their relatives.

### P-84 Refusal of intensive care admission: assessment of a Tunisian ICU practices

#### Merhabene Takoua^1^, Blel Farah^1^, Jamoussi Amira^1^, Ayed Samia^1^, Ben Khelil Jalila^1^, Besbes Mohamed^1^

##### ^1^Hôpital Abderrahman Mami, Ariana, Tunisia

###### **Correspondence:** Merhabene Takoua - takouamg@yahoo.fr

*Annals of Intensive Care* 2018, **8(Suppl 1):**P-84

**Introduction:** Need of intensive care exceeds its availability in several countries. As a consequence, rationing intensive care unit (ICU) beds is common and often leads to admission refusal. Purpose- To report refusal determinants and characteristics of patients associated with decisions to deny ICU admission.

**Patients and methods:** This study was performed at the ICU of Abderrahman Mami Hospital, a 22-bed ICU in Ariana, Tunisia. It was a prospective study enrolled between 1st January and 31th December 2016. No predefined admission criteria were determined. Decisions to admit are based on a combination of patient-related factors, severity of illness and bed availability. All consecutive patients referred for admission to ICU during the study period were included. 2 groups were defined GI—Admitted patients and GII—refused patients. The reasons for refusal were categorized as follows: too well to benefit, too sick to benefit, patient or family refusal, necessity of other exploration not available in our institution and unit too busy.

**Results:** During the study period, ICU admission was requested for 1081 patients of whom 590 were admitted (54%). Of the 491 patients refused, only 41 were admitted to ICU later. Refusal of ICU admission came in 23% of cases from the emergency room and wards of our hospital, in 65% from other hospitals of whom 30% without ICU. Reasons of refusal were no beds availability (76.2%), too sick to benefit from ICU (13%), too well (9.4%) and necessity of other exploration (1.4%). No differences in demographic characteristic between the two groups were noted. Among the refused patients, when compared with admitted patients, we found higher proportions of hematologic malignancies (p < 0.01) and cardiocirculatory arrest (p = 0.01). On the other hand, admitted patients were more likely to have cardio-respiratory comorbidities (437/590 vs. 297/491, p = 0.01) and more need to mechanical ventilatory support (404 590 vs. 166 491, p = 0.016).

**Conclusion:** Our study confirms that ICU refusal rate still high. It depends on both ICU organization and patient characteristics.

### P-85 Acute heart failure syndroms in intensive care: clinical features, management and outcome

#### Jamoussi Amira^1^, Ajili Achraf^1^, Merhebene Takoua^1^, Ayed Samia^1^, Ben Khelil Jalila^1^, Besbes Mohamed^1^

##### ^1^Hôpital Abderrahmen Mami, Ariana, Tunisie

###### **Correspondence:** Jamoussi Amira - dr.amira.jamoussi@gmail.com

*Annals of Intensive Care* 2018, **8(Suppl 1):**P-85

**Introduction:** Classification of acute heart failure (AHF) into 5 clinical scenari (CS) was first proposed to facilitate early management (1). A decade after implementation of this approach, epidemiological and evolutive data based on this classification are interesting to investigate. That is why we aim to describe frequencies, management and mortality of each AHF syndrom in intensive care.

**Patients and methods:** A prospective study of patients > 18 years with AHF admitted to the medical intensive care unit (ICU) of Abderrahmen Mami hospital from January 2017 to August 2017 was conducted. Patients were classified according to the 5 clinical scenari (1). Clinical, therapeutic and outcome findings were recorded.

**Results:** During the study period (8 months), we admitted 291 patients in ICU from whom 43 (14.8%) presented with AHF and then enrolled. The median age was of 63 ± 18.6 years and sex-ratio 1.05. A medical history of COPD (20.9%), hypertension (46.5%), diabetes (20.9%), ischemic cardiopathy (28%) and valvular cardiopathy (16.3%) were noticed. At admission, severity assessement scores were: median APACHE II 18.5 ± 10.5 and median SAPS II 30 ± 17.6. Clinical and evolutive characteristics according to clinical scenari are listed in Table 1.

**Table 1:** Evaluative characteristics according to clinical scenarios

	CS 1 (n = 12; 27.9%)	CS 2 (n = 14; 32.5%)	CS 3 (n = 4; 9.3%)	CS 4 (n = 5; 11.6%)	CS 5 (n = 8; 18.6%)
Age (med ± DS), years	66.5 ± 13.9	72.5 ± 17.8	49.5 ± 27.6	62 ± 11.6	50 ± 22.2
SAPS II (med ± DS)	36 ± 21.9	30 ± 11.9	39 ± 6.9	28 ± 7.8	23.5 ± 25.2
APACHE II (med ± DS)	18.5 ± 10.5	19 ± 7.06	18 ± 2.06	14 ± 2.28	16 ± 10.7
Hypertension history : n (%)	6 (50)	11 (78)	2 (50)	0	1 (12.5)
PaO_2_/FiO_2_ (med ± DS), mmHg	170 ± 68.8	161.6 ± 71.8	159.1 ± 124.6	168.5 ± 13.6	190 ± 64.5
Need IMV n (%)	4 (33.3)	4 (28.6)	2 (50)	2 (40)	5 (62.5)
NIV only n (%)	8 (66.6)	10 (71.4)	2 (50)	3 (60)	3 (37.5)
MV length (med ± DS), days	12 ± 57.9	9 ± 12.2	10 ± 8.5	9 ± 3	59 ± 64.5
LOS (med ± DS), days	8 ± 5.63	3 ± 21.5	1 ± 2.5	8 ± 4.6	2 ± 3.9
Mortality n (%)	3 (25)	3 (21.4)	3 (75)	2 (40)	4 (50)

CS: clinical scenario; IMV: invasive mechanical ventilation; MV: mechanical ventilation; LOS: length of stay

**Conclusion:** CS 2 and CS 1 are the most frequent AHF syndroms in ICU and also have the best outcome.

### P-86 Recurrent arrhythmia after resuscitation from ischemic ventricular fibrillation: analysis of the PROCAT registry

#### Bellut Hugo^1^, Guillemet Lucie^1^, Bougouin Wulfran^1^, Charpentier Julien^1^, Ben Hadj Salem Omar^1^, Llitjos Jean-François^1^, Paul Marine^1^, Valade Sandrine^1^, Spagnolo Shirley^1^, Chiche Jean-Daniel^1^, Marijon Eloi^1^, Pène Frédéric^1^, Varenne Olivier^1^, Mira Jean-Paul^1^, Dumas Florence^1^, Cariou Alain^1^

##### ^1^Hôpital Cochin, Paris, France

###### **Correspondence:** Bellut Hugo - hbellut@gmail.com

*Annals of Intensive Care* 2018, **8(Suppl 1):**P-86

**Introduction:** In cardiac arrest patients resuscitated from an ischemic ventricular fibrillation or tachycardia (VF/VT), both incidence and risk factors of recurrent severe arrhythmia are unclear. Whether it is useful to give a prophylactic anti-arrhythmic (AA) treatment during the first hours and days is debated, particularly when a successful coronary reperfusion was provided. We aimed to evaluate the incidence of severe arrhythmia in patients resuscitated from an ischemic VF VT and to identify risk factors for developing arrhythmia during their ICU stay.

**Patients and methods:** The PROCAT registry captures all data from patients admitted in a tertiary hospital center after a resuscitated cardiac arrest (CA). We selected patients with an initial VF VT caused by an acute coronary syndrome (ACS) and who were successfully treated with early percutaneous coronary intervention (PCI) on admission. The primary endpoint was the recurrence of major arrhythmia between ICU admission and ICU discharge. All arrhythmias resulting in CA recurrence and or severe arterial hypotension requiring infusion of vasopressors were classified as major arrhythmias. Multivariate logistic regression identified factors associated with the occurrence of major arrhythmias.

**Results:** Between 01/2007 and 12/2016, 256 consecutive CA patients were included in the analysis. All patients underwent a successful PCI of the infarct-related artery on hospital arrival. The only drug used as a prophylactic AA treatment was amiodarone, which was employed in 104/256 patients (41%). Overall, 29/256 patients (11.3%) had a major arrhythmia recurrence during their ICU stay. A large majority of these major arrhythmia recurrences (79.3%) occurred during the first 24 h. Characteristics of patients with and without major arrhythmia recurrence are described in the Table 1. In multivariate analysis, public place location (OR 0.3 [0.1–0.9], p = 0.029) and male gender (OR 0.3 [0.1–0.9], p = 0.036) were both associated with a lower risk of major arrhythmia recurrence during the ICU stay. Prophylactic AA treatment was not associated with a lower risk of recurrences of major arrhythmias (OR 0.9 [0.3–3.1], p = 0.870).

**Table 1:** Characteristics of patients who presented or not major arrhythmia recurrence

	Overall N = 256	Major arrhythmia N = 29	No major arrhythmia N = 227	*p* value
Age > 59 years; n (%)	130 (51)	16(55)	114 (50)	0.62
Male gender; n (%)	205 (80)	20 (69)	185 (82)	0.14
Coronaropathy; n (%)	35(14)	3 (11)	32 (14)	0.78
Public place; n (%)	124 (48)	7 (24)	117 (51)	0.006
Bystander CPR; n (%)	180 (72)	22 (76)	158 (71)	0.62
Number of defibrillation > 2; n (%)	117 (46)	16 (57)	101 (45)	0.23
Time between cardiac arrest and ROSC > 20 min; n (%)	117 (48)	15 (56)	102 (47)	0.40
Norepinephrine > 1 mg; n (%)	106 (43)	16 (59)	90 (41)	0.08
Amiodarone; n (%)	104 (41)	17 (59)	87 (39)	0.07
Shock after resuscitation; n (%)	133 (52)	17 (59)	116 (51)	0.56
Dobutamine and/or norepinephrine in ICU; n (%)	144 (52)	19 (66)	125 (55)	0.33

**Conclusion:** Despite an early coronary reperfusion, more than 10% of our post-cardiac arrest patients experienced a recurrent severe arrhythmia during the post-resuscitation period, mostly during the first 24 h in the ICU. This proportion is much higher than what is reported in common acute coronary syndrome (without cardiac arrest) and further studies are needed to explore protective strategies.

### P-87 Kinetics of anaerobic metabolic markers during the first hours of septic shock

#### Zriouel Siham^1^, Farkas Jean-Christophe^2^

##### ^1^Hôpital Saint Antoine, Paris, France; ^2^Polyclinique de Saint-André, Reims, France

###### **Correspondence:** Zriouel Siham - zriouelsiham@yahoo.fr

*Annals of Intensive Care* 2018, **8(Suppl 1):**P-87

**Introduction:** During symptomatic treatment of septic shock, markers of anaerobic metabolism may be used in a goal-oriented strategy. The recent International Guidelines for Management of Sepsis and Septic Shock 2016 suggested guiding resuscitation to normalize lactate as a marker of tissue hypoperfusion. The purpose of this study was to evaluate the kinetics of lactate and other markers during the first three hours and to compare their levels between survivors and non survivors.

**Patients and methods:** We conducted a prospective, observational, single-center study of patients admitted to a general ICU from the May to August 2016. Inclusion criteria were patients age ≥ 18, intubated and under mechanical ventilation with septic shock as defined by the Third International Consensus Conference. Simultaneous sampling of arterial and central venous blood gas were collected at H0 and H3 to obtain lactate (mmol/l), and ScvO2 (%). Delta PCO2 (mmHg) and delta PCO2/CavO2 (mmHg/ml) were computed by our Patient Data Management System and presented as a chart with additional hemodynamic data for clinical decision support. Comparisons of values between groups were made by Mann–Whitney U test as appropriate. p < 0.05 was considered statistically significant. All reported p values are two-sided. Statistical analysis was performed using Systat ver. 11.0.

**Results:** We studied 19 intubated septic shock patients aged 71 ± 10 years, SAPS II 68 ± 23, SOFA 11 ± 3.6. Community pneumonia and peritonitis were the major sources of infection. ICU mortality rate was 58%. All patients received norepinephrine (0.62 ± 0.5 µg/kg/min), two patients received Dobutamine (5.5 ± 0.2 µg/kg/min). The evolution of markers is summarized in Table 1. At H0 and H3, arterial lactate levels were higher in non-survivors than in survivors, but did not decrease at H3 in both groups. At H0 there was no statistical difference concerning ScvO2, delta PCO2 and delta PCO2/CavO2. After three hours of resuscitation, delta PCO2 and delta PCO2/CavO2 ratio decreased and ScvO2 increased in survivors. Survivors had lower delta PCO2 and delta PCO2/CavO2 ratio than non survivors.

**Table 1:** Kinetics of anaerobic markers

	H0				H3			
	All	Survivors	Non survivors	p	All	Survivors	Non survivors	p
Lactate	5.2 ± 4.2	3 ± 1.1	7.7 ± 5	< 0.001	5.5 ± 3.9	3.4 ± 1.6	7.9 ± 4.5	0.01
ScvO2	66.2 ± 13.8	70.6 ± 6.2	61.3 ± 18	ns	71 ± 9.8	75.6 ± 8.3	66 ± 9.7	ns
ΔPCO2	8.4 ± 5	6.6 ± 3.6	10.2 ± 5.5	ns	6.9 ± 4.7	4.6 ± 2.4	9.4 ± 5.5	0.03
ΔPCO2/CavO2	2.6 ± 1.7	1.7 ± 1.1	3.2 ± 2	ns	2 ± 1.4	1.4 ± 0.7	2.6 ± 1.7	0.02

**Conclusion:** Although high lactate level is a key signal of anaerobic metabolism, it did not decrease during the first three hours in this group of severe septic shock patients. Instead of using lactate, delta PCO2 and delta PCO2/CavO2 kinetics could be integrated in a goal-oriented strategy for septic shock resuscitation.

### P-88 Oesophageal Doppler can predict fluid responsiveness through end-expiratory and end-inspiratory occlusion tests

#### Depret François^1^, Jozwiak Mathieu^1^, Teboul Jean-Louis^1^, Alphonsine Jean-Emmanuel^1^, Richard Christian^1^, Monnet Xavier^1^

##### ^1^CHU Bicêtre, Le Kremlin-Bicêtre, France

###### **Correspondence:** Depret François - francois.depret@aphp.fr

*Annals of Intensive Care* 2018, **8(Suppl 1):**P-88

**Introduction:** To assess whether, in patients under mechanical ventilation, fluid responsiveness is predicted by the effects of short respiratory holds on cardiac index estimated by oesophageal Doppler (CIDoppler).

**Patients and methods:** In 28 patients, before infusing 500 mL of saline, we measured CIDoppler before and during the 5 last seconds of successive 15-second end-inspiratory occlusion (EIO) and end-expiratory occlusion (EEO), separated by 1 min. Patients in whom volume expansion increased cardiac index (transpulmonary thermodilution) > 15% were defined as “fluid responders”.

**Results:** EEO increased CIDoppler more in responders than in non-responders (8 ± 2 vs. 3 ± 1%, respectively, p < 0.0001) and EIO decreased CIDoppler more in responders than in non-responders (-9 ± 5 vs. -4 ± 2%, respectively, p = 0.0002). Thus, when adding the absolute values of changes in CI observed during both occlusions, CIDoppler changed by 18 ± 5% in responders and 7 ± 2% in non-responders. Fluid responsiveness was predicted by the EEO-induced change in CIDoppler with an area under the receiver operating characteristic (ROC) curve of 1.00 (95% confidence interval—0.88–1.00) and a threshold value of 4% increase in CIDoppler. It was predicted by the sum of absolute values of changes in CIDoppler during both occlusions with a similar area under the ROC curve (0.99 (0.86–1.00)) and with a threshold of 9% change in CIDoppler, which is more compatible with oesophageal Doppler precision. In this case, the sensitivity was 100(77–100)% and the specificity was 93(66–100)%.

**Conclusion:** If consecutive EIO and EEO change CIDoppler > 9% in total, it is very likely that volume expansion will be efficient in terms of cardiac output.

### P-89 The measurement of cardiac output using a signal morphology-based form of impedance cardiography (Physioflow^®^) in intensive care unit: comparison with the trans thoracic echocardiography. Study REACSHOCK

#### Marc Julien^1^, Brault Clement^1^, Zerbib Yoann^1^, Kontar Loay^1^, Soupison Thierry^1^, De Cagny Bertrand^1^, Lepretre Pierre Marie^1^, Maizel Julien^1^, Slama Michel^1^

##### ^1^CHU d’Amiens, France

###### **Correspondence:** Marc Julien - julienmarc88@gmail.com

*Annals of Intensive Care* 2018, **8(Suppl 1):**P-89

**Introduction:** In the Intensive care units, the cardiac output (CO) is one of the main hemodynamic parameters required to manage patients in shock. The Physioflow^®^ is a new non-invasive method using the waveform analysis of the thoracic impedance signal (TI) to assess CO. In hemodynamicaly unstable patients, no studies have evaluated the level of agreement between the CO estimated by transthoracic echocardiography (CO-TTE) and that measured using the waveform analysis of thoracic Impedance Physioflow^®^ (CO-TI). The objective of this study was to evaluate the ability of CO-TI relative to CO-TTE to estimate the absolute CO value and detect the expected variation CO (V-CO) in critically ill patients.

**Patients and methods:** Fourteen patients sedated and mechanically ventilated, in shock under catecholamines and monitorred with TTE and TI Physioflow^®^ were included. Hemodynamic datas, stroke volume (SV) and CO with two monitoring were performed at baseline 2 min before passive leg raising (PLR), 30 s after PLR and 2 min after volume expansion (VE) of 500 ml of saline solution. Responders were defined by an increase > 12% of cardiac output (V-CO) after PLR.

**Results:** Fourteen pairs of TTE and TI measurements were compared. The median (IQR) age was 69 years (55–76), IGS2 was 54 (40–69). Only 3 patients were responders after PLR. There was a significant correlation between the CO-TTE and CO-TI measurements (r = 0.807, p < 0.0001). The median bias was 0.8 L/min and the limits of agreement (LOAs) were −3.5 and 1.9 L/min. There was a significant correlation between V-CO-TTE and V-CO-TI (r = 0.77, p = 0.0022) (Fig. 1). The median bias was—5.2% and the LOAs for V-CO were respectively -26.5 and +16.5%.

**Fig. 1:**
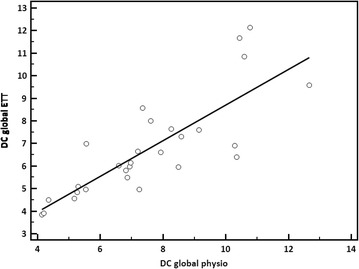
Cardiac output

**Conclusion:** The CO measured with PhysioFlow^®^, a signal morphology-based impedance cardiography, is correlated to the CO measured with TTE. However, the high LOA observed in this preliminary study underline the necessity to remain careful and wait for further inclusions.

### P-90 Hemodynamic parameters of septic shock patients treated with prior BB therapy

#### De Roquetaillade Charles^1^, Jean-Francois Llitjos^1^, Jamme Matthieu^1^, Charpentier Julien^1^, Cariou Alain^1^, Chiche Jean-Daniel^1^, Mira Jean-Paul^1^, Pène Frédérique^1^

##### ^1^Hopital Cochin, Paris, France

###### **Correspondence:** De Roquetaillade Charles - deroquetaillade.charles@gmail.com

*Annals of Intensive Care* 2018, **8(Suppl 1):**P-90

**Introduction:** Impact of Beta-Blockers (BB) on hemodynamics in sepsis remains a complex issue in critically ill. BB may worsen the early circulatoryfailure by limiting the adaptive cardiac and vasopressive response. Recent data suggests that BB could exert a protective role insepsis by improving cardiovascular dynamics. We aimed to investigate hemodynamic parameters and outcomes of septic shockpatients with respect to prior BB treatment.

**Patients and methods:** We conducted a 9-year (2008–2016) retrospective study. All adult patients diagnosed for septic shock within the first 48 h were included. Septic shock was defined as an infection associated with circulatory failure requiring vasopressors. BB treatment was stopped at ICU admission. Qualitative and quantitative parameters were compared using the Chi square or Mann–Whitney tests.

**Results:** Among 938 patients admitted for septic shock, 230 (24.5%) patients were previously treated with BB. These patients weresignificantly older (72.9 [61.5–80] vs. 66.9 [56–78] years, p < 0.01) and had more comorbidities including congestive heart failure (35% vs. 18%, p < 0.01), cirrhosis (15 vs. 9%, p < 0.01) and chronic renal insufficiency (18 vs. 12%, p = 0.01) (Table 1). With respect to hemodynamic parameters at ICU admission, patients with prior BB displayed lower heart rates (81[82–111] vs. 107[89–122] bpm, p < 0.01). However, we found no differences in arterial lactate levels and oxygen content (1.75 [0.9–3.4] vs. 1.8[0.8–4] mmol/L, p = 0.97 and 13.8, [11.5–15.9] vs. 13.5, [11.3–15.8] mLO2/dL, p = 0.34). Regarding hemodynamic resuscitation during the first 24 h, patients previously treated with BB received less fluid (2.95 [0.5–3.4] vs. 3.4 [2–5] L, p < 0.01) but required less amounts of norepinephrine (23.2 [5.1–57] vs. 22.4 [5.2–60] mg, p = 0.95). Duration ofmechanical ventilation was lower in patients with prior BB treatment (4 [2–9] vs. 5.5 [2–11] days, p = 0.05). Despite imbalancedunderlying characteristics in terms of demographics and comorbidities, in-ICU mortality rates were similar between patients (35.7 vs. 37%, p = 0.75).

**Table 1:** Hemodynamic parameters of septic shock patients treated with prior BB therapy

Baseline characteristics	Total (n = 938)	ß-blockers (n = 230)	no ß-blockers (n = 708)	*P*
Age, mean (SD)	69 (58–79)	72.9 (61.5–80)	66.9 (56–78)	<.001
Male, n (%)	598 (63.8%)	152 (66.1%)	446 (63%)	0.43
Prior congestive heart Failure, n (%)	210 (22.4%)	81 (35%)	129 (18.2%)	<.001
Cirrhosis, n (%)	99 (10.6%)	36 (15.7%)	63 (8.9%)	0.004
Diabetes, n (%)	172 (18.3%)	51 (22.2%)	121 (17.1%)	0.077
Obesity, n (%)	88 (9.4%)	23 (10%)	65 (9.2%)	0.696
Immunosuppression, n (%)	355 (37.8%)	84 (36.5%)	271 (38.3%)	0.696
Chronic obstructive pulmonary disease, n (%)	149 (15.9%)	33 (14.3%)	116 (16.4%)	0.533
Prior kidney failure, n (%)	127 (13.5%)	42 (18.3%)	85 (12%)	0.019
SOFA J0, mean (SD)	9 (6–13)	9 (6–12)	9 (6–13)	0.242
IGS2, mean (SD)	67 (52–84)	68 (54–85)	67 (51–84)	0.245
Heart rate on admission, mean (SD)	103.5 (87–120)	81.5 (82–111)	107 (89–122)	<.001
Creatinin, mean (SD)	142.5 (89–222)	165.5 (108–245)	135.5 (82–208)	<.000
Bilirubin, mean (SD)	13 (8–27)	14 (8.75–28.25)	12 (8–27)	0.065
TP, mean (SD)	59 (44–72)	56.5 (41–70)	60 (45–73)	0.101
Leucocyte count, mean (SD)	12 (5.4–20)	13.4 (6.0–19.8)	11.8 (5.0–20.6)	0.576
Arterial pH, mean (SD)	7.34 (7.24–7.42)	7.35 (7.25–7.42)	7.34 (7.23–7.42)	0.354
Arterial lactate, mean (SD)	1.8 (0.9–3.9)	1.75 (0.9–3.4)	1.8 (0.8–4.0)	0.974
Hemoglobin, mean (SD)	11 (9.1–12.5)	10.9 (9.3–12.4)	10.9 (9.1–12.5)	0.618
CaO2, mean (IC)	1357 (1146–1588)	1385 (1159–1598)	1355 (1139–1587)	0.347
*Outcome*				
Norepinephrine dose on day 1, mean (IC)	22.58 (5.18–59)	23.2 (5.1–57.0)	22.4 (5.2–60.5)	0.95
Use of positive inotropic drugs, n (%)	51 (5.4%)	9 (3.9%)	42 (5.9%)	0.248
Epinephrine use, n (%)	75 (8.0%)	19 (8.3%)	56 (8.1%)	0.89
Fluid requirement on day 1, mean (IC)	3250 (1900–4900)	2950 (1500–4500)	3400 (2000–5000)	0.001
Fluid balance on day 1, mean (IC)	2100 (700–3950)	1790 (500–3430)	2200 (800–4070)	0.027
Protidemia at 24h, mean (IC)	58 (50–66)	59 (51.7–68)	57 (50–66)	0.015
Urine Output on day 1, mean (IC)	870 (350–1430)	835 (352–1400)	900 (340–1600)	0.442
Mechanical ventilation, n (%)	789 (84.1%)	188 (81.7%)	601 (84.9%)	0.112
Days of mechanical Ventilation, mean (SD)	5 (2–10.5)	4 (2–9)	5.5 (2–11)	0.055
In-hospital mortality, n (%)	344 (36.7%)	82 (35.7%)	262 (37%)	0.753

**Conclusion:** Prior BB treatment have limited impact on the severity of acute circulatory failure in septic shock and is not associated with increased mortality despite the underlying frailty of patients.

### P-91 Cardiac output measurement in critically ill patient with atrial fibrillation: a comparison between transpulmonary thermodilution and transthoracic echocardiography

#### Rouget Antoine^1^, Castanera Jeremy^2^

##### ^1^CHU de rangueil, Toulouse, France; ^2^CHU de Toulouse, France

###### **Correspondence:** Rouget Antoine - rouget.a@chu-toulouse.fr

*Annals of Intensive Care* 2018, **8(Suppl 1):**P-91

**Introduction:** Cardiac output monitoring is a key component in the management of critically ill patients. Cardiac output estimated by transthoracic echocardiography is documented in patient with atrial fibrillation, but a large part of transpulmonary thermodilution validation studies excluded this specific population. The objective of this study was to evaluate cardiac output mesurement and trend ability by transpulmonary thermodilution relative to transthoracic echocardiography in critically ill mechanically ventilated patients with atrial fibrillation.

**Patients and methods:** Thirty mechanically ventilated patients requiring hemodynamic assessment were included in a prospective observational study. Cardiac output was mesured simultaneously with transpulmonary thermodilution and transthoracic echocardiography.

**Results:** Seventy-four pairs of cardiac output measurements were compared. The two measurements were significantly correlated (r = 0.769 et p < 0.0001). The mean bias was -0.7 l/min, the limits of agreement were -2.9 and +1.6 l/min, and the percentage error was 39.81%. Thirty-four pairs of cardiac output variation measurements were compared. There was no significant correlation between cardiac output variation measurements by transpulmonary thermodilution and transthoracic echocardiography. The mean bias was −0.19 l/min and the limits of agreement were −1.82 and +1.43 l/min. With a 15% exclusion zone, the four-quadrant plot had a concordance rate of 78.6%. The polar plot had a mean polar angle of 14.2° with 95% confidence interval between − 14.2° and 42.7°.

**Conclusion:** In critically ill mechanically ventilated patients with atrial fibrillation, cardiac output measurements with transpulmonary thermodilution and transthoracic echocardiography are not interchangeable.

### P-92 Diagnostic capability of a novel miniaturized imaging system in patients assessed using basic critical care echocardiography for cardiopulmonary compromise

#### Goudelin Marine^1^, Dalmay François^1^, Gonzalez Céline^1^, Lafon Thomas^1^, Daix Thomas^1^, François Bruno^1^, Vignon Philippe^1^

##### ^1^CHU Dupuytren, Limoges, France

###### **Correspondence:** Goudelin Marine - marinegoudelin@gmail.com

*Annals of Intensive Care* 2018, **8(Suppl 1):**P-92

**Introduction:** Basic critical care echocardiography (CCE) relies on transthoracic echocardiography (TTE). We sought to assess the diagnostic capacity of a next-generation micro-digital broadband beamformer in patients with cardiopulmonary compromise.

**Patients and methods:** All patients with acute circulatory respiratory failure underwent two basic TTE assessments using successively a next-generation micro-digital broadband beamformer (64 elements, 1–4 MHz) incorporated in a sector phased array probe with two-dimensional, M-mode and color Doppler mapping capacities which was connected to a touchscreen interface (Lumify, Philips), and using a compact full-feature imaging system (80 elements, 1–5 MHz + Cx50, Philips). TTE examinations were independently performed in random order by two intensivists with expertise in CCE, within a 10-min time frame without therapeutic intervention. Imaging quality was graded from 0 (no image in the corresponding view) to 3 (clear identification of 100% of endocardial boarders). The concordance of qualitative data was assessed using the Kappa test and agreement of two-dimensional measurements (left ventricular end-diastolic diameter [LVEDD], ratio of right ventricular (RV) and LV end-diastolic diameters [RVEDD LVEDD] + end-expiratory inferior vena cava diameter [DexpIVC]) was evaluated using intraclass coefficient correlation (ICC).

**Results:** Thirty consecutive patients were studied, without any exclusion for absence of TTE images (age, 56 ± 19 years, SAPSII, 45 ± 18, 70% ventilated, 60% under catecholamines, lactate, 3.9 ± 3.3 mmol l). The proportion of echocardiographic views eligible for interpretation and mean duration of TTE examinations were similar with the miniaturized and full-feature systems (79 vs. 77%, 7.1 ± 2.7 vs. 7.9 ± 4.1 min, p = 0.31). Two-dimensional imaging quality grade was lower with the miniaturized system (1.78 ± 0.69 vs. 1.91 ± 0.74, p = 0.007). Agreement between responses to binary clinical questions and therapeutic proposals were good-to-excellent as reflected by Kappa values ranging between 0.76 [IC95% 0.33–1.0] and 1.0 [IC95% 1.0–1.0] and between 0.81 [IC95% 0.48–1.0] and 1.0 [IC95% 1.0–1.0], respectively. Four acute and massive valvular regurgitations (mitral, n = 3, aortic, n = 1) were accurately depicted by the miniaturized system. Concordance of two-dimensional measurements was also good-to-excellent (Table 1).

**Table 1:** Comparison

Two-dimensional measurements	Feasibility of measurements miniaturized/full-feature system	Miniaturized system	Full-feature system	ICC (95% CIs)
LVEDD, mm	94/94%	49.8 ± 8.9	49.9 ± 8.5	0.97 (0.93–0.99)
RVEDD/LVEDD	75/75%	0.77 ± 0.17	0.79 ± 0.16	0.78 (0.41–0.93)
D_exp_IVC, mm	63/63%	22.1 ± 5.6	21.9 ± 5.4	0.94 (0.76–0.98)

**Conclusion:** For basic CCE use, next-generation micro-digital broadband beamformer appears providing reliable information with good-to-excellent diagnostic capability, accurate two-dimensional measurements, and adapted therapeutic suggestions. These preliminary data require further confirmation.

### P-93 Outcome of Acute kidney Injury in very old ICU patients

#### Lagrange Sophie^1^, Vivier Emmanuel^1^, Bourdin Gaël^1^, Rosselli Sylvène^1^, Putegnat Jean-Baptiste^1^, Doroszewsky Fanny^1^, Villar Emmanuel^1^, Pommier Christian^1^

##### ^1^CH St Joseph St Luc, Lyon, France

###### **Correspondence:** Lagrange Sophie - sophielagrange@gmail.com

*Annals of Intensive Care* 2018, **8(Suppl 1):**P-93

**Introduction:** Acute kidney injury (AKI) in very old patients (over 80 years) admitted in intensive care unit (ICU) is a frequent issue and is known to be associated with a severe prognosis. We aimed at describing the clinical characteristics and prognosis of such a population. The objective of the study was dual: first to evaluate the short and long term mortality of these patients, second to determine the factors associated with a poor outcome.

**Patients and methods:** We conducted a descriptive, retrospective and monocentric study based on the hospital records of patients over 80 years with AKI admitted in our ICU between January 2013 and December 2016. The patients were selected according to the KDIGO criteria (1). Survivals at the discharge from hospital, at day 90 and at 1 year were assessed. The factors associated with mortality at 1 year were scrutinized.

**Results:** After excluding 30 patients for an initial therapeutic limitation, the data of 171 remaining patients were reviewed. The patients were 84 years old (interquartile range, IQR 81–86) and were predominantly male gender (61%). SAPS II and SOFA score at admission were 62 (IQR 57–74) and 8 (IQR 5–10) respectively. 57% of the patients needed for mechanical ventilation and 69% of them needed for catecholamine use. Septic (44%), prerenal (38%), iatrogenic (17%) and cardiogenic injury (10%) were the leading cause of AKI. Dialysis was performed in 42% of patients. The overall mortality at the discharge from ICU, at day 90 and at 1 year was 39, 52 and 66% respectively (Fig. 1). Neither were the age, the comorbidities, the etiology of AKI nor the need for dialysis associated to a significant increase in mortality. A stepwise Cox regression analysis revealed SAPS II and blood lactate level at ICU admission as independent risk factors associated with 1 year mortality.

**Fig. 1:**
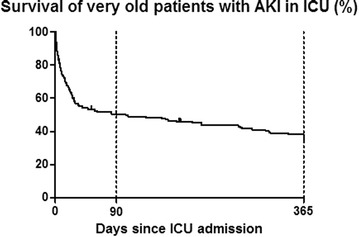
Survival of very old patients with AKI in ICU (%)

**Conclusion:** AKI at admission in ICU is associated with a high mortality at 1 year in an elderly population. Main long term prognostic factors are linked to the initial severity at ICU admission.

**Reference**

1. Stevens PE, Levin A, Kidney Disease—Improving Global Outcomes Chronic Kidney Disease Guideline Development Work Group Members. Evaluation and management of chronic kidney disease—synopsis of the kidney disease—improving global outcomes 2012 clinical practice guideline. Ann Intern Med. 4 juin 2013;158(11):825–30.

### P-94 Prognostic factors of death for the 80 years of age and over at admission to the icu

#### Leclercq Marion^1^, Kanagaratnam Lukshe^1^, Drame Moustapha^1^, Rouget David^1^, Cousson Joël^1^

##### ^1^CHU Reims, Reims, France

###### **Correspondence:** Leclercq Marion - ma.l.ice@hotmail.fr

*Annals of Intensive Care* 2018, **8(Suppl 1):**P-94

**Introduction:** The proportion of elderly around the world doesn’t stop growing and increases the consumption in health care. However, lots of studies report the impact of the age on the decision to admit a patient to the ICU despite no triage recommendation exists. The primary objective was to determine prognostic factors of death for the 80 years and over at admission to the ICU and secondly to evaluate their functional prognostic at short and medium term after their exit.

**Patients and methods:** Prospective and observational study conducted in our ICU 30 beds unit from August 2015 to February 2017. Patients of 80 years and over were listed. The dying patients arriving after a pre-hospital resuscitation for whom no therapeutic plan has been initiated and those admitted for an organ donation were excluded. The primary outcome was the duration between the admission and the potential death during the follow-up. The secondary outcomes were the necessity to entry an healthcare institution or the loss of one autonomy point on the ADL French scale after the hospitalization.

**Results:** 107 patients of the 1763 admissions were included. The IGS II and SOFA average scores were respectively 70.23 ± 22.47 and 9.02 ± 3.79. The most common diagnosis were a septic shock (23 patients), a cardiopulmonary arrest (21 patients), a cardiogenic shock (9 patients) and a pulmonary oedema or a lung infection (8 patients each). 75 patients (70.03%) died during the follow up—50 at the ICU, 13 during ward and 12 during re-education or after their home return. From a multivariate analysis (Table 1), anisocoria, cardiopulmonary arrest and acute kidney injury (AKI > 0) seem to be independent risk factors of death. 32 patients were alive at the end of the follow up. 11 recovered their previous autonomy, 8 needed a place in a specialized institution. All the other lost a part of autonomy 3 months after their home return with the average loss of one point on the ADL autonomy French scale.

**Table 1:** Independent risk factors of death for ICU patients 80 years of age and over

	HR	IC 95%	p
Anisocoria	2.21	1.01–4.85	0.04
Acute renal injury	1.71	0.98–2.98	0.005
Cardiopulmonary arrest	2.50	1.46–4.26	0.0008

**Conclusion:** Anisocoria, AKI and cardiopulmonary arrest seem to be independent risk factors of death for those patients. Concerning the survivors, a stay at the ICU lead to an increased dependency. Other studies have to be led to evaluate which of our patients could have get the best benefit of their stay to prevent from a misuse of the structure.

### P-95 Acute kidney failure in septic schock: comparison of RIFLE, AKIN and KDIGO criteria to diagnosis AKI and predict mortality

#### Jamoussi Amira^1^, Ktari Mariem^1^, Merhebene Takoua^1^, Ayed Samia^1^, Ben Khelil Jalila^1^, Besbes Mohamed^1^

##### ^1^Hôpital Abderrahmen Mami, Ariana, Tunisia

###### **Correspondence:** Jamoussi Amira - dr.amira.jamoussi@gmail.com

*Annals of Intensive Care* 2018, **8(Suppl 1):**P-95

**Introduction:** Since 2004, various criteria have been proposed to define and grade acute kidney injury (AKI). Nevertheless, fixed criteria for assessing septic shock related AKI have not yet been established (1). Aim—To assess the power of the different methods of AKI classification (RIFLE, AKIN and KDIGO) to diagnosis AKI and to predict mortality in a cohort of patients with septic schock.

**Patients and methods:** A retrospective study of patients > 18 years with septic shock admitted to the intensive care unit (ICU) of Abderrahmen Mami hospital from January 2016 to December 2016 was conducted. Patients with history of chronic renal failure were not included. Plasma creatinine levels and diuresis were measured daily. Patients were then classified according to RIFLE, AKIN, KDIGO criteria.

**Results:** During the study period (12 months), we enrolled 38 patients. The median age was of 62 ± 20 years and sex-ratio 1.53. At admission, the median seric creatinin clearance was of 70.5 ± 56.5 µmol/l, median diuresis (first 6 h) was of 250 ± 330 ml and lactates 3.6 ± 3.1 mmol/l. Severity assessement scores—median APACHE II 22.5 ± 7.7 and median SOFA 9 ± 3. The percent of AKI rate according to the different criteria was 68.4% for RIFLE, 71.1% for AKIN and 73.7% for KDIGO. There was a very good agreement between the 3 classifications criteria to stadify AKI severity: RIFLE and AKIN, Kappa = 0.938; RIFLE and KDIGO: Kappa = 0.872; AKIN and KDIGO: Kappa = 0.934. Median length of stay was of 7 ± 6.6 days. Global mortality was of 76.3% (n = 29). Mortality according to AKI occurence was compared for each classification. It was significant for KDIGO (p = 0.036) but not for RIFLE (p = 0.108) or AKIN (p = 0.088). Each severity stage defined by the 3 classifications was related to a higher risk of ICU mortality—RIFLE (p = 0.030), AKIN (p = 0.004) and KDIGO (p = 0.021).

**Conclusion:** A high rate of patients with septic schock developed AKI, which can be classified according to different criteria. KDIGO criteria were the most sensitive to diagnosis AKI and to predict mortality in patients with septic schock.

### P-96 Post-partum acute kidney injury: sorting placental and non-placental thrombotic microangiopathies using the trajectory of biomarkers

#### Meybodi Fleuria^1^, Jamme Matthieu^2^, Vassilis Tsatsaris^1^, François Provot^1^, Mercédès Jourdain^1^, Anne-Sophie Ducloy-Bouthors^1^, Yahsou Delmas^1^, Pierre Perez^1^, Julien Darmian^1^, Alain Wynckel^1^, Jean-Michel Rebibou^1^, Jérome Lambert^1^, Véronique Frémeaux-Bacchi^1^, Paul Coppo^1^, Cédric Rafat^1^, Luc Frimat^1^, Alexandre Hertig^1^

##### ^1^Hôpital Tenon, Paris, France

###### **Correspondence:** Meybodi Fleuria - f.meybodi@hotmail.fr

*Annals of Intensive Care* 2018, **8(Suppl 1):**P-96

**Introduction:** Context—Among the severe complications of preeclampsia, acute kidney injury (AKI) poses a dilemma if features of thrombotic microangiopathy (TMA) are present. Although a HELLP syndrome is considerably more frequent, ruling out a flare of atypical haemolytic and uremic syndrome (HUS) is then of utmost importance. Objective—To improve the differential diagnosis procedure in cases of post-partum AKI.

**Patients and methods:** A hundred and five cases of post-partum AKI, admitted in the last 5 years (2011–2015) in French ICU from 9 different regions, were analysed. Initial and final diagnosis, renal features, haemostasis and TMA parameters were all analysed, paying a special attention to their dynamics within the first days following the delivery.

**Results:** The main circumstances of AKI were severe preeclampsia (n = 40), post-partum haemorrhage (PPH, n = 20) and primitive TMA (n = 14, including 10 atypical HUS and 4 thrombotic thrombocytopenic purpura). Among the thirteen cases of renal cortical necrosis, 11 were associated with preeclampsia. Congruence between the initial and the final diagnosis was low (63%). Thus, none of the women referred to our centers for a suspicion of non-placental TMA has received a final diagnosis of non-placental TMA (and instead had a PE or a PPH). Conversely, all women with a final diagnosis of non-placental TMA were referred for a suspicion of PE-related TMA, or with a PPH which polluted the diagnosis. Tranexamic acid was largely used in the context of PPH (82%), at a dose up to 5 grams total. Taking into account the final diagnosis, we subjectively concluded that plasma exchanges and eculizumab were abusively indicated in 5 and 2 cases, respectively, of typical HELLP syndrome. Plasma exchanges were in itiated in all 14 cases, a mean 84 h following the admission. Dynamics of hemoglobin, haptoglobin, and liver enzymes were poorly discriminant. The dynamic pattern of LDH and of platelets, in contrast, was statistically different between primitive TMA-related AKI and other groups—at day 3, platelets increased in preeclamptic women, and in other circumstances, but not in patients with primitive TMA. A classification and regression tree (CART) independently confirmed the usefulness of platelets and LDH trajectory in the diagnostic algorithm (Fig. 1).

**Fig. 1:**
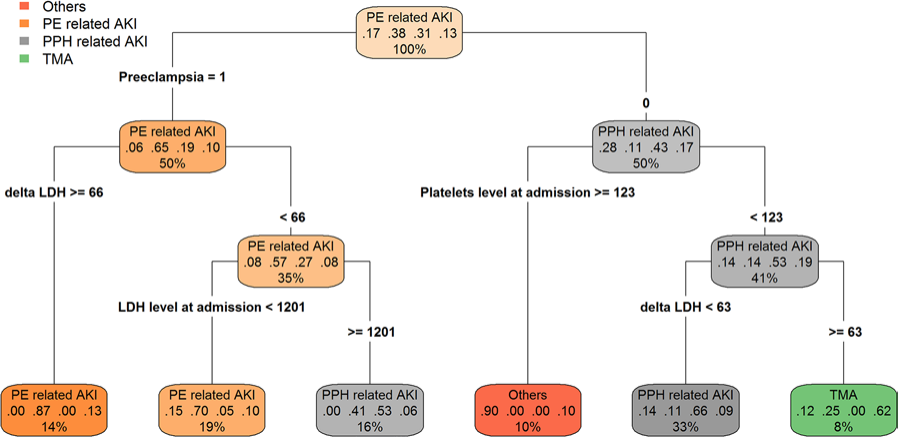
Analysis of post-partum AKI cases

**Conclusion:** The trajectory of LDH and platelet count is useful to identify the cause of post-partum AKI, and the clinician may reasonably take therapeutic decisions at day 3 post-delivery.

### P-97 Continuous veno-venous hemofiltration and filter’s lifespan: comparing systemic enoxaparin anticoagulation versus regional citrate anticoagulation

#### Mathais Quentin^1^, Cungi Pierre Julien^2^, D’Aranda Erwan^1^, Nguyen Cédric^1^, Laurent Matthieu^1^, Schmitt Johan^1^, Prunet Bertrand^1^, Meaudre Eric^1^

##### ^1^CHU de Toulon, France; ^2^HIA Sainte Anne, France

###### **Correspondence:** Mathais Quentin - quentin.mathais@orange.fr

*Annals of Intensive Care* 2018, **8(Suppl 1):**P-97

**Introduction:** Continuous veno-venous hemofiltration (CVVHF) is a common practice in intensive care units (ICU). Because it is continuous, the choice of anticoagulation is essential—regional anticoagulation with citrate or systemic with unfractionned heparin or low molecular weight heparin (LMWH). Filter’s lifespan is a major issue regarding filtration’s effectiveness and cost. In this study, we compared the filter’s lifespan between LMWH and citrate anticoagulation.

**Patients and methods:** A monocentric retrospective study was led from January to October 2016. All the CVVHF sessions during this period were included. Prismaflex© monitors (Hospal) were used. Practioners were free to choose between citrate or LMWH defining 2 groups. We aimed a post filter ionized calcemia between 0.25 to 0.5 mmol/L in citrate group + and a post filter anti Xa activity between 0.2 to 0.5 UI/mL in LMWH.

**Results:** 95 CVVHF sessions were included—64 with LMWH anticoagulation, and 31 with citrate. Patients were 62 years old on average, primarly males (65%), with an initial average SAPS II score of 73. ICU mortality was 51%. Patients’ hemostasis was measured before each CVVHF session, without any significant difference between the 2 groups. Global filter’s lifespan was 47 h + 54 h in citrate group versus 44H in LMWH, without significant difference (p = 0.236) (Fig. 1). No serious side effect, especially hemorrhage in the LMWH group, was reported. Filtration efficiency, represented by the urea reduction ratio during the first CVVHF session, was similar, 30% ± 20% in LMWH group versus 20% ± 20% in citrate group (p = 0.235).

**Fig. 1:**
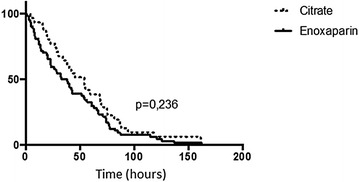
Filter’s lifespan

**Conclusion:** Both anticoagulation—systemic with LMWH or regional with citrate can be used in ICU. Both methods enable long and comparable filter lifespan, with similar filtration efficiency and without serious adverse events. Our results need to be confirmed by a randomized propective study.

### P-98 Evaluation of criteria associated with renal replacement therapy (RTT) for acute renal failure (ARF) in intensive care unit (ICU) during the post-partum

#### Malacrino Dominique^1^, Satre Buisson Lea^2^, Garabedian Charles^2^, Ducloy-Bouthors Anne-Sophie^2^, Robriquet Laurent^2^, Jourdain Merce^2^

##### ^1^CH de Bethune, France; ^2^CHRU Lille, Lille, France

###### **Correspondence:** Malacrino Dominique - dominique.malacrino@gmail.com

*Annals of Intensive Care* 2018, **8(Suppl 1):**P-98

**Introduction:** ARF during the post-partum period is a rare complication. The main etiologies are post-partum haemorrhage (PPH) and thrombotic microangiopathy (TMA). RRT may be required. The aim of this study was to identify variables associated with RRT in this population admitted in ICU.

**Patients and methods:** We conducted a study using retrospectively collected data in a cohort of patient with post-partum ARF according to the KDIGO criteria and requiring ICU in the University hospital of Lille from 2008 until 2016. Two groups were compared—RRT and non RRT patients. Demographic and obstetrical data as well as data during ICU stay and patients’ outcome were collected. Etiologies of ARF, KDIGO stage, anuria, hemolysis parameters and biological data at ICU admission were studied. Comparisons were made using a Chi-two or Fisher Exact test or a Mann–Whitney U test. Odds Ratio (OR) for the statistically different criteria were studied.

**Results:** Twenty-two patients requiring RRT were compared to the 49 patients without RRT. The two main etiologies of ARF were TMA (50.7%) and PPH (35.2%). Vaginal delivery was significantly more frequent in the RRT group compared with caesarian delivery (p = 0.04). Use of RTT was significantly increased after PPH compared the others etiology of ARF (p = 0.03). In the RRT group, the ICU length of stay was longer (p < 0.0001) and IGS II score was higher (p < 0.0001). Higher KDIGO score was observed in RRT patients (in the RRT group—KDIGO 1 = 0, 2 = 9%, 3 = 91%, and without RRT—KDIGO 1 = 38.8%, 2 = 36%, 3 = 36%, p < 0.0001). Anuria 24 h after ICU admission was more frequent in cases of RTT (86.3% versus 20.3%, p < 0.0001). Hemolysis was greater in RRT patients with lower haptoglobin (p = 0.02) and increased Lactate Deshydrogenase (LDH) (p = 0.04). The association with RRT requirement was stronger with the duration of anuria, with an OR at 6 h at 31.3 [8.1–121.7] and at 24 h at 72.2 [8.3–622]. A lower haptoglobin was associated with a higher risk of RTT (OR 3.7 [1.2–11.7]), as well as PPH (OR 3.1 [1.1–8.8]) and vaginal delivery (OR 2.9 [1.02–8.1]).

**Conclusion:** Hemolysis parameters and anuria seemed useful criteria to identify patients at higher risk of RRT early during their ICU admission.

### P-99 Renal Replacement Therapy in ICU of Moroccan University Hospital

#### Qamouss Youssef^1^, Aissaoui Younes^1^, Boughalem Mohamed^1^

##### ^1^Hopital Militaire Avicenne, Marrakech, Morocco

###### **Correspondence:** Qamouss Youssef - yqamouss@yahoo.fr

*Annals of Intensive Care* 2018, **8(Suppl 1):**P-99

**Introduction:** Renal replacement therapy (RRT) has three aims—restoring homeostasis, ensuring survival and preserving the potential for renal recovery. The main indication of RRT in ICU is acute renal failure, correlated with a very important rate of mortality despite the progress made in its management.

**Patients and methods:** The objective of this work is to take stock of the indications and the objectives of the RRT in ICU. Through a prospective study, we report a serie of 39 cases, collected at the multipurpose resuscitation unit of the Avicenna military hospital in Marrakech between September 2015 and September 2016.

**Results:** The average age of our patients is 65, 10 ± 24, 89 years with extremes ranging from 34 to 90 years and a male predominance (77%). The main reasons for admission were hemodynamic distress in 43.5% of cases, followed by septic shock in 30.7% of cases, neurological and respiratory distress were noted in 20.5 and 5.3% of cases, respectively. RRT indications were severe acidosis in 27% of patients, followed by 21% hyperkalaemia, acute pulmonary edema in 13%, hemodynamic instability in patients with chronic renal failure in 12%, acute renal failure in 8%, and hyperuriaemia in 5% of cases. The technique chosen is conventional intermittent hemodialysis with a synthetic membrane. The main duration of the sessions was 2 h 06 min ± 45 mn. Vascular access was a right internal jugular catheter in 49.7% of patients and left in 2.5%, right femoral catheter in 34.8% of patients and left in 2.4%, arteriovenous fistula (FAV) and a tunneled catheter in 7.9 and 2.7% of patients. Mortality was 61, 55%, chronicity progressed in 10.25% of cases and total or partial recovery of normal renal function in 28% of cases.

**Conclusion:** we have a high rate of mortality in our ICU that’s why we will focus on prevention of risk of renal failure in our patients.

### P-100 Outcome of continuous veno-venous hemofiltration vesus hemodialysis on adult traumatic intracranial hemorrhage with acute kidney injury: a nationwide population-based retrospective study in Taiwan

#### Chia-Chao Wu^1^, Tseng Min Feng^1^

##### ^1^Tri-Service General Hospital, Taipei, Taiwan

###### **Correspondence:** Chia-Chao Wu - wucc@mail.ndmctsgh.edu.tw

*Annals of Intensive Care* 2018, **8(Suppl 1):**P-100

**Introduction:** There is limited information on the outcome of acute kidney injury (AKI) in patients with traumatic intracranial hemorrhage (tICH). TICH patient with AKI was related high mortality rate. The aim of this study is to estimate the outcome using different renal replacement therapy on the survival rate and rate of long term renal-replacement therapy in adult tICH patient.

**Patients and methods:** We retrospectively identified a total of 310 tICH patients with AKI who required glycerol or mannitol therapy admitted to the intensive care unit during a 10-year period ending DEC 2010 from the National Health Insurance Research Database. Demographic data, severity of tICH, medication, level of care, type of head surgery were collected. All patients subjects were older than > 18 years. We also excluded patients diagnosed with tICH before the cohort entry date, hemodialysis before tICH, chronic kidney disease cancer coagulation defects purpura and other hemorrhagic conditions, mortality mechanical ventilation ischemic heart disease before tracking. The primary outcome was overall survival at day 30. The secondary outcome was the rate of long term HD therapy.

**Results:** A total of 310 patients were enrolled. The Kaplan–Meier estimates of mortality at day 30 did not differ significantly between the continuous veno-venous hemofiltration (CVVH) and hemodialysis (HD) strategies + 5 deaths occurred among 134 patients receiving CVVH-strategy group and 9 deaths occurred among 176 patients receiving HD-strategy group (adjusted hazard ratio: 0.782, 95% CI 0.239 to 2.558; p = 0.685). The rate of long term HD was higher in the HD-strategy group than in the CVVH-strategy group (15.34 vs. 7.46%, p = 0.021) especially in injury severity score ≥ 16 group (Table 1).

**Discussion:** In our study, tICH patient with AKI receving CVVH may have effect on renal blood flow protection or cytokine removal which lower the rate of long term HD.

**Conclusion:** These clinical data provides readers interventions to improve outcomes in this population and future study are needed to confirm the result. This study highlights the importance different renal replecement therapy in the tICH with AKI population (Table 1).

**Table 1:** Factors of long-term HD stratified by variables listed in the table by using Cox regression

Hemodialysis methods	CVVH			HD			Ratio	Adjusted HR	95% CI	95% CI	p
Strarified	Event	PYs	Rate (per 10^5^ PYs)	Event	PYs	Rate (per 10^5^ PYs)					
Total	10	815.23	1226.65	27	963.11	2803.42	0.438	0.368	0.158	0.858	0.021
Gender											
Male	6	593.66	1010.68	23	831.41	2766.38	0.365	0.307	0.139	1.154	0.452
Female	4	221.57	1805.30	4	131.70	3037.21	0.594	0.500	0.215	1.116	0.334
ISSg 16											
Without	6	615.15	975.37	19	792.98	2396.03	0.407	0.315	0.067	1.479	0.143
With	4	200.08	1999.20	8	170.13	4702.29	0.425	0.358	0.155	0.839	0.020
Head surgery											
Without	8	754.32	1060.56	25	889.15	2811.67	0.377	0.317	0.136	1.740	0.206
With	2	60.91	3283.53	2	73.96	2704.16	1.214	0.921	0.438	2.381	0.595
Tracking time											
f 30 days	0	1.02	0.00	4	8.84	45,248.87	0.000	0.000	–	–	0.842
>31 days, <1 year	4	41.03	9748.96	5	351.28	1423.37	6.849	5.760	0.868	17.643	0.297
g 1 year	6	733.19	818.34	18	602.99	2985.12	0.274	0.231	0.101	0.498	0.001

PYs: person-years; adjusted HR: adjusted Hazard ratio: adjusted for gender, age, comorbities, medicine, season, urbanization, level of care, and insured premium; CI: confidence interval CVVH: continuous veno-venous hemofiltration HD: hemodialys

### P-101 Digestive surgical emergencies of elder patients

#### Khaleq Khalid^1^, Hattabi Khalid^1^, Bensardi Fatima Zahra^1^, Bouhouri M. A^1^, Nciri A^1^, Hamoudi D^1^, Alharrar R^1^

##### ^1^CHU Ibn Rochd, Casablanca, Morocco

###### **Correspondence:** Khaleq Khalid - khaleq20@gmx.fr

*Annals of Intensive Care* 2018, **8(Suppl 1):**P-101

**Introduction:** The frequency of surgical procedures of the elderly people increases with longevity of the population. In an emergency, the doctor has little time to evaluate the patient’s condition, which leaves place for approximate diagnostics that lead to a certain reluctance in operating the elderly. The objective of our study is to determine predictive factors of mortality for emergency of digestive surgical pathology of the elderly people.

**Patients and methods:** 100 patients aged of 65 years or more underwent emergency surgery for a digestive abdominal affection between 1st January 2014 and 31 December 2016 within the service of emergencies of surgical visceral P35 and having stayed in the Surgical Resuscitation. Several data have been the subject of univariate and multivariate statistical analysis using the SPSS software.

**Results:** The average age was 73.24 years old. 54 (54%) were men and 46 (46%) were women, 67% had comorbidities dominated by hypertension 40% followed by diabetes 28%, The main reason of consultation was the suspicion of an occlusive syndrome (41%). The most frequent surgical indications were acute peritonitis (35%), followed by intestinal occlusion (18%), overall mortality was 58%, the main cause of death was septic shock in 57% of cases, By using multivariate statistical analysis we have deduced several prognostic factors significantly related to mortality with a p value < 0.05: Age ≥ 76ans «OR 15.686/IC 95% : 1,285–191,521» , Time to surgical takeover > 6 h «OR 10,980/IC 95% : 1,263–95,464» , Albuminemia ≤ 30 g/l «OR 8,065/IC 95% : 1,019–63,827» , Surgeon experience (junior) «OR 12,1546/IC 95% :1,440–102,446» , Vasoactive Drug Administrations «OR 0.004/IC 95% : 0.001–0.063» , SAPS II «OR 1,290/IC 95% :1,107–1,503» , POSSUM SO Score ≥ 30 «OR 4,555/IC 95% : 1,148–18,064» SP > 20 «OR 3,355/IC 95% : 1,057–10,651» .

**Conclusion:** The eldery subject, known for his fragility and his multimorbidity, has to consult urgently for a surgical digestive pathology, and due to the high mortality, it is necessary to give more interest to this age category. The ideal would be to have a geriatric service within the hospital that can train specialist doctors in gerontology, and above all to establish health structures that could ensure the proper care of the elderly subject.

### P-102 Postoperative mortality and morbidity in digestive surgery

#### Rghioui Kawtar^1^, Azime Meryem^1^, El Allam Wafaa^1^, Cherkab Rachid^1^, El Kettani Chafik^1^, Barrou Lahoucine^1^

##### ^1^CHU Ibn Rochd, Casablanca, Morocco

###### **Correspondence:** Rghioui Kawtar - rghioui.kawtar@gmail.com

*Annals of Intensive Care* 2018, **8(Suppl 1):**P-102

**Introduction:** The combined progress of abdominal surgery and anesthesia lead to more frequent surgical indications, including for fragile patients or serious pathologiesPostoperative morbidity and mortality is an element that requires evaluation and analysis in surgical resuscitation. Although pathological processes and new therapeutic approaches in surgery are currently well known, data on risk factors for morbidity and mortality are less available. The aim of our work is to evaluate the post-operative morbidity and mortality rate and to identify the main predictive factors.

**Patients and methods:** A retrospective-cohort, unicentric study that included all consecutive patients hospitalized in the surgical resuscitation department after abdominal surgery regardless of the operated organ, during 3 years. The structured sheet of data collection included more than 100 items on all perioperative data concerning the patient, the disease, and the operating surgeons. Postoperative mortality and morbidity were defined as in-hospital death and complications. A first descriptive analysis of the various parameters collected was carried out A bivariate analysis was then performed to study the factors affecting morbidity and mortality in digestive surgery The comparison was made using the student’s t test for quantitative variables and the Chi square for the qualitative variables. A difference is considered significant when p < 0.05 (5%).

**Results:** Among 360 patients, the in-hospital death rate was 15.08% and the overall morbidity rate was 41.61%, the mean age was 55.54 ± 15, 10 years with extreme ages of 18 years and 85 years with sex ratio of 1.01. Five factors were incriminated in post: operative mortality notably—renal failure p = 0.002, duration of stay p = 0.001, parenteral nutrition p = 0.047, long duration of intubation p = 0.001, perioperative blood transfusion p = 0.001. Three factors influencing morbidity were found: duration of stay p = 0.003, Parenteral nutrition p = 0.018, long duration of intubation p = 0.0001.

**Conclusion:** Knowledge of the true frequency of both mortality and morbidity is crucial in planning health care and research and identifying risk factors.

### P-103 Gastric Ultrasound in ICU patient’s Bowel Management

#### Malki Khalil^1^, Housni Brahim^1^, Aziz Mohammed^1^

##### ^1^CHU Mohammed VI Oujda, Oujda, Morocco

###### **Correspondence:** Malki Khalil - khalil.malki@gmail.com

*Annals of Intensive Care* 2018, **8(Suppl 1):**P-103

**Introduction:** Tools to quantify and assess bowl management in critically ill are still very limited and often over-looked. With the primary concern of optimizing patients to preserve life, the problem of bowel care has been given less priority. The aim of this study was to use ultrasonographic measurements of gastric emptying in the critically ill as a tool of measurement of the impact of different specific factors of ICU stay on bowl emptying.

**Patients and methods:** This is a prospective study conducted in an Intensive care unit for 1 months. It included 32 patients. Ultrasonic imaging of antral sections was undertaken every 15 min for the first 1 h and every 30 min thereafter until total emptying. Correlation analyses were calculated, applying an adjusted significance level (Pb < .0125) to correct for multiple testing.

**Results:** All our patients were above the age of 18. The median of age was years old 31. 19 of our patients were male and 13 were female. The total emptying median time was 273 ± 21 min. Significant correlation was observed between length of stay and delay in bowl emptying. Mechanical ventilation had also significant relation with slower bowl progression and gastric emptying. Patients in septic shock had tendencies to earlier delayed bowl emptying compare to others patients included in our study.

**Conclusion:** The study we conducted is a pilot study. Further studies should be conducted and unltrasonografic gastric assessment could be standardized in protocols to assess clinical decision making and improve nutrition and bowl management in ICU patients.

### P-104 Administration of drugs by nasogastric tube among critically ill patients: evaluation of ICU nurses

#### Piton Gaël^1^, Marceau Sarah^1^, Pili-Floury Sébastien^1^, Vettoretti Lucie^1^, Martinez Benjamin^1^, Krouk Y^1^, Capellier Gilles^1^, Clairet Anne-Laure^1^

##### ^1^CHU de Besançon, France

###### **Correspondence:** Piton Gaël - gpiton@chu-besancon.fr

*Annals of Intensive Care* 2018, **8(Suppl 1):**P-104

**Introduction:** Enteral nutrition, via a feeding tube, is often used in Intensive care units (ICU) to supply artificial nutrition to critically ill patients. The feeding tube is also commonly used to administrate drug therapy as well. However, there is a lack of knowledge of the nurses about this way of administration. This could be a potential source of medicine-related illness. The purpose of this study was first, to evaluate the nurse’s knowledge on enteral drug administration, and second, to observe nurses and to evaluate the adequacy of their practices with guidelines, and to report medication-administration errors.

**Patients and methods:** This prospective study using the observation technique was conducted in 2 ICU (one medical and one surgical). First, a knowledge and practice questionnaire regarding drug administration trough enteral feeding tube was filled by each intensivist nurse. Secondly, Pharmacist performed observations of nurses during preparation and administration of medications. These practices were compared with the original medical prescription and with the data available in the literature.

**Results:** 60 questionnaires were returned. 48 nurses evaluated their knowledge as medium and 9 as inadequate. There was a lack of knowledge on the type of drugs which can be used by feeding tube (65 wrong responses). 30 nurses and 71 different drugs were observed during the drug administration phase. No administration totally complied with our institutional protocol, particularly the crush of tablets. When a tablet was crushed, in 31% an alternative formulation (in syrup for example) existed.

**Conclusion:** The correct administration of drugs in feeding tubes is important and represents a challenge in ICU. Firstly, crushed tablets is the most frequent cause of obstruction of feeding tubes which have to be changed + secondly, crushed tablets destroys the controlled release of enteric coated dosage forms, resulting in a higher or a lower initial blood level. We have to train nurses for drug administration by feeding tube. On their daily ward, the pharmacist should improve the choice of medication’s forms.

### P-105 Early outcome of acute variceal hemorrage (avh) in cirrhotic patients: preliminary study

#### Khedher Sana^1^, Cyrine Abdennebi^1^, Wafa Azaza^1^

##### ^1^Tunis, Tunisia

###### **Correspondence:** Khedher Sana - sanakhedher@hotmail.fr

*Annals of Intensive Care* 2018, **8(Suppl 1):**P-105

**Introduction:** Acute variceal hemorrhage (AVH) is a severe complication of portal hypertension. In addition, the variceal bleeding is still the most common lethal complication of cirrhosis. The most effective modality of treating is based on resuscitation combined with the endoscopic variceal band ligation. The purpose of this preliminary study was to find the factors associated with poor prognosis of AVH in cirrhotic patients.

**Patients and methods:** This is a retrospective study, spread over 12 months between January 2016 and December 2016. Are included all consecutive patients with liver cirrhosis hospitalized for variceal bleeding. We exploited the medical records to identify the clinical, biological and endoscopic parameters.

**Results:** A total of 30 patients hospitalized for AVH occurred during the study period. The mean age at admission was 60 years, and 12 are female. Cirrhosis was post viral in 30% of cases. Patients were classified as Child–Pugh C in 40% of cases. The median presenting model for end stage liver disease (MELD) and CLIF SOFA Were respectively 17 and 8.4. Twelve (12) patients received beta-blockers and 15 have required at least one endoscopic variceal band ligation at the time of the bleeding episode. In the acute phase, pharmacological treatment based on vasopressor (sandostatin)) was instituted in all cases and combining with antibiotic prophylaxis (C3G or fluoroquinolone) in 26 cases. In 8 cases the endoscopy was made within 12 h, Active bleeding at endoscopy was observed in 20 patients. Esophageal avarices (OV) were grade I (2 patients) grade II (10 patients) and grade III (18 patients). The eradication of varices was obtained in 23 patients (76.6% percentage of the cases). The variceal bleeding recurred in 9 of patients (30%of cases) and 6 patients died which 4 within the first 5 days. Spontaneous Bacterial Peritonitis (p 0.010), hepatic encephalopathy (p 0.041) and the hemodynamic instability with schok (p 0.01) are correlated with early mortality at 5 days. Hepatic encephalopathy (p 0.028) and bacteremia (p 0.05) are corrolated with 6 week motality. Non selective beta-blocker (p 0.013) and primary use of band ligation when indicated (p 0.008) are protective factors and parameters of good outcome.

**Conclusion:** Despite developing of endoscopic tools and respect of actual therapeutic guidelines in AVH, the outcome is still poor. The prognosis appears to be dependent on the clinical condition at admission and primary prevention.

### P-106 Outcomes in ICU of patients with acute occlusive arterial mesenteric ischemia benefiting from open revascularization in an intestinal stroke center

#### Da Costa Ines^1^, Corcos Olivier^1^, Castier Yves^1^, Pellenc Quentin^1^, Roussel Arnaud^1^, Huguet Audrey^1^, Maggiori Léon^1^, Tanaka Sebastien^1^, Augustin Pascal^1^, Lortat-Jacob Brice^1^, Paugam Catherine^1^, Montravers^1^, Philippe, Tran Dinh Alexy^1^

##### ^1^Hôpital Beaujon, Paris, France

###### **Correspondence:** Da Costa Ines - inesdacosta123@gmail.com

*Annals of Intensive Care* 2018, **8(Suppl 1):**P-106

**Introduction:** The French intestinal stroke center based on a multimodal and multidisciplinary management has been developed to improve survival and intestinal viability. Open surgical revascularization was decided for patients unsuitable for radiological revascularization and or suspected of intestinal necrosis. We aimed to study the prognosis of patients suffering from AOAMI in ICU and who have benefited from open revascularization.

**Patients and methods:** Single-center, observational and prospective study was carried out in a surgical ICU of a tertiary center. Patients with AOAMI managed in our intestinal stroke center from 01 2016 to 09 2017 and who underwent open revascularization were included.

**Results:** Data of 15 patients were collected. Patients’ characteristics are described in Table 1. All patients had abdominal computed tomography angiography at the diagnosis, and 11 patients (73%) presented signs of intestinal injury. Thrombosis was the main mechanism of superior mesenteric artery (SMA) occlusion (14 patients, 93%). All patients received antiplatelet therapy, curative unfractionated heparin therapy and digestive decontamination. Open revascularization was performed by SMA endarterectomy (4 patients, 27%), SMA surgical bypass (8 patients, 53%), retrograde open mesenteric stenting (3 patients, 20%) and coeliac artery bypass (2 patients, 13%). Three patients (20%) underwent a radiologic endovascular revascularization attempt before open repair. Small bowel resection (100 cm [IQR 35–250]) was achieved in 8 patients (53%). Four patients (27%) had peritonitis. Six patients (40%) had one or more relaparotomy (2 [IQR 1.3–2.8]), usually for hemodynamic instability (83%). Only one patient died in ICU (7%). ICU lenght of stay was 7 days [IQR 4.5–14.5] and duration of mechanical ventilation was 3 days [IQR 0–7]. Overall, haemodynamic failure was present in 10 patients (67%). Median duration of vasoactive support was 2 days [IQR 0–4]). Severe acute respiratory distress syndrome was observed in 2 patients (13%) and acute kidney injury in 9 patients (60%, including 2 patients who received renal-replacement therapy, 13%). Enteral feeding was initiated in 6 patients (40%) with a delay of 3.5 days [IQR 3–7]. Parenteral nutrition was administered in 13 patients (87%), including 8 patients (53%) without enteral feeding. Five patients (33%) were discharged with small bowel syndrome.

**Table 1:** Patients’ characteristics

Patients’ characteristics	
Age, median [IQR]	65 [55–74]
Sex (male/female), n	9/6
Past medical history, n (%)	
Chronic mesenteric ischemia	4 (27)
Smoking	10 (67)
Coronary artery disease	4 (27)
Chronic hypertension	7 (47)
Lower limb arteriopathy obliterans	4 (27)
Hypercholesterolemia	6 (40)
Immunosuppression	1 (7)
Thrombophilia	2 (13)
SAPS-II, median [IQR]	43 [29–56]
SOFA, median [IQR]	
On the day of admission	5 [4–7]
Day 1	4 [2–7.8]
Day 3	2 [1–6]

**Conclusion:** ICU patients who underwent open revascularization to treat AOAMI as part of a multimodal and multidisciplinary management in a dedicated intestinal stroke center have low mortality and intestinal resection rates. Larger studies are needed to confirm these results.

### P-107 Late intestinal transit in critical care: Toward an improved definition criterion?

#### Sztrymf Benjamin^1^, Prat Dominique^1^, Messika Jonathan^2^, Jacobs Frédéric^1^, Hamzaoui Olfa^1^, Trouiller Pierre^1^, Demars Nadege^1^, Millerux Maude^1^, Ricard Jean Damien^2^

##### ^1^Hôpital saint Antoine, Clamart, France; ^2^Hôpital Louis Mourier, Colombes, France

###### **Correspondence:** Sztrymf Benjamin - benjamin.sztrymf@aphp.fr

*Annals of Intensive Care* 2018, **8(Suppl 1):**P-107

**Introduction:** Precise consequences of late transit in ICU remain elusive. We have previously shown that defining late transit by the absence of stool within 6 days after admission was not relevant because it did not identify a group of patients with specific outcome [1]. To further improve this definition, we investigated the differences in outcome among patients according to their bowel movements frequency.

**Patients and methods:** Preliminary results of a prospective, two centers, observational study. All patients admitted to ICU, with a length of stay (LOS) of at least 72 h were eligible and included with the following exceptions—abdominal surgery, bowel infection or any baseline condition known to alter transit time. Patients were compared according to stool frequency—less than 25%, between 25 and 50%, between 50 and 75% or more than 75% of ICU days. We also tested the former constipation definition of more than 6 days after admission without stool passage. We registered demographic data, time spent under mechanical ventilation (MV), ICU LOS, ventilation associated pneumoniae (VAP) and vital status at discharge.

**Results:** Over 10 months, 421 patients were screened and 141 (33.5%) were included, age 64.6 ± 16.7 years, mean SAPS II 45 ± 17, 71 (50.3%) mechanically ventilated. The most frequent exclusion criteria were LOS < 72 h (n = 140). 68% of the patients had stool less than 50% of ICU days. Patients with fewer bowel movements were more likely to be mechanically ventilated, without association with time spent under MV. There was a link between the time to first stool after admission and the stool frequency during ICU (p < 0.0001). No difference was evidenced regarding SAPS II, ICU LOS, VAP occurrence and survival. When testing the delay of 6 days to first stool as a diagnostic criterion, we found that constipated patients spent more time under MV (9[10.8] vs. 6[8.8] p = 0.004), had a higher LOS (12[11] vs. 6[5] p < 0.0001) and more VAP (0[0] vs. 0[1] p = 0.03), but no link with survival.

**Table:** Outcomes according to frequency of stool passage

	<25% (n = 42)	25–50% (n = 54)	50–75% (n = 33)	>75% (n = 12)
Age (years)	65.9 [25.1]	64.1 [21.8]	67.5 [25.4]	75.4 [7.4]
SAPS II	39 [18]	42 [17]	42 [32]	46 [20]
Time between admission and first stool (days)	6.5 [4]	3 [2]	2 [3]	1 [2]
MV n(%)	27 (64.3)	31 (57.4)	10 (30.3)	3 (25)
Time spent under MV (days)	5 [7.5]	8 [12]	14.5 [14]	7 [6]
LOS (days)	7 [6]	7.5 [9]	7 [9.5]	5.5 [5]
VAP n(%)	4 (9.5)	6 (11.1)	2 (6.1)	2 (16.7)
Death n(%)	4 (9.5)	3 (5.5)	5 (15.2)	1 (8.3)

**Discussion:** This study is limited by the number of patients leading to an imbalance between subgroups therefore limiting the comparison.

**Conclusion:** These preliminary results do not plead for an improvement of the late transit definition based on the frequency of stool. Further data is warranted to better define this condition, and the management to provide.

### P-108 Epidemiology and prognostic factors of infective endocarditis in intensive care unit

#### Hajjej Zied^1^, Dahmani Salma^1^, Sellami Walid^1^, Samoud Walid^1^, Daiki Mayssa^1^, Ferjani Mustapha^1^

##### ^1^Hôpital militaire de Tunis, Tunisie

###### **Correspondence:** Hajjej Zied - hajjej_zied@hotmail.com

*Annals of Intensive Care* 2018, **8(Suppl 1):**P-108

**Introduction:** Infective endocarditis (IE) treated in intensive care unit is the severe forms of this disease. Clinical and epidemiological data concerning this pathology in intensive care are uncommon. The aim of our study was to analyze the clinical and epidemiological profile as well as factors associated with the mortality of IE in intensive care units.

**Patients and methods:** This was a retrospective, observational study, including patients admitted from 1 January 2008 to 31 December 2016 in the medical surgical intensive care unit at the Tunisian military hospital with a confirmed diagnosis of Endocarditis.

**Results:** 35 patients were included for the study. Concerning the site of infection, 77.2% occurs on native valve and 11.4% on prosthetic valve. Endocarditis occurs on the left-sided valve in 87.7% of cases and on the right-sided valve in 14.3% of cases. Blood cultures remained negative in 20% of cases. The most common germs were staphylococci (46.4%), with a predominance of *S. aureus*. In the second place, streptococci (17.8%) and enterococci (17.8%) were equally divided. In multivariate analysis, the factors associated with hospital mortality were—advanced age, high EuroSCORE II, extended length of stay, A high-admission SOFA score, a cardiogenic or septic shock, and a cerebral embolic complication, and the presence of a double mito-aortic involvement. On the other hand, delayed surgery had a protective effect that significantly reduced mortality.

**Conclusion:** Mortality of IE in ICU remains high. The search for predictive factors of mortality will allow an optimal and an early management. Like prospective studies carried out outside resuscitation unit and which have allowed a large recruitment of this rare disease, similar studies should be carried out in ICU in order to confirm our results.

### P-109 Broncho-alveolar alpha amylase for the diagnosis of aspiration pneumonia in out hospital cardiac arrest

#### Bourenne Jeremy^1^, Belloni Axel^1^, Fraisse Megan^1^, Carvelli Julien^1^, Lambert Dominque^1^, Fromonot Julien^2^, Hraeich Sami^3^, Gainnier Marc^1^

##### ^1^Hôpital de la Timone, Marseille, France; ^2^Laboratoire de Biochimie, Marseille, France; ^3^Réanimation DRIS, Marseille, France

###### **Correspondence:** Bourenne Jeremy - jeremy.bourenne@ap-hm.fr

*Annals of Intensive Care* 2018, **8(Suppl 1):**P-109

**Introduction:** Aspiration pneumonia (AP) is a common complication of out hospital cardiac arrest (OHCA). Recent data confirmed an improved survival of patient admitted to the Intensive Care Unit with a targeted temperature management (TTM). The diagnosis of AP is then challenging due to immunologic effects and the absence of fever that results from TTM. Alpha amylase is a marker only present in salivary secretions. The aim of our study is to evaluate the performance of broncho-alveolar amylase for early prediction of AP following OHCA with successful resuscitation treated by TTM in the ICU.

**Patients and methods:** All patients admitted for OHCA underwent non-bronchoscopic BAL (Combicath PRODIMED) in ICU. Aspirate was collected after two instillations of 20 ml of isotonic saline. Amylase measurement was realized in the biochemistry laboratory on the first sample. The second sample and the extremity of the Combicath were sent to the microbiology laboratory for culture. All patients had 24 h of TTM between 35–36 °C with deep sedation and neuromuscular blocking. Initiation of antibiotic therapy was left to the clinician’s appreciation. Diagnosis of AP was independently confirmed by investigators, following the American Thoracic Criteria.

**Results:** The following results are an intermediate analysis of 41 out of 150 patients planned. Among the 41 patients, 5 have died within 48 h, but only 2 of them without aspiration pneumonia symptoms were excluded. The average age was 53 years ± 14, the average SOFA score at admission was 9 ± 3. The principal diagnosis for OHCA was acute coronary syndrome (26%). An alpha amylase level superior or equal to 4 UI L was associated with the occurrence of AP with a sensitivity of 69.2 (0.05–0.83) and a specificity of 73.3 (0.48–0.89). The area under the Receiver Operating Characteristic (ROC) curve was 0.788. The negative predictive value was only 58%.
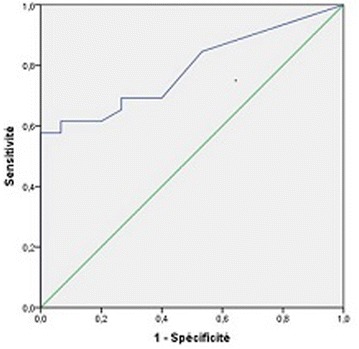


**Discussion:** Level of alpha amylase observed here is lower than in a study conducted by Weiss and al.. Indeed, a cut-off of 125 UI L was found, but all the samples were collected by bronchoscopic BAL. Non bronchoscopic BAL is a rapid, non invasive and inexpensive technique for bronchial alpha amylase measurement. The use of this diagnostic tool can avoid the prescription of unnecessary antibiotics.

**Conclusion:** A bronchial amylase concentration above 4 UI L after non bronchoscopic BAL is associated with a high risk of AP in OHCA with TTM.

### P-110 Mucoid capsular character of *Klebsiella pneumonia*e: factor of severity?

#### Serie Mathieu^1^, Fauvet Thomas^1^, Tarantola Arnaud^1^, Merlet Audrey^1^, Marot Benoit^1^, Colot Julien^1^, Goarant Cyrille^1^

##### ^1^CHT, Nouméa, France

###### **Correspondence:** Serie Mathieu - mathieu.serie@cht.nc

*Annals of Intensive Care* 2018, **8(Suppl 1):**P-110

**Introduction:** Since the 1980s, a hyper mucoid phenotype of Klebsiella Pneumoniae (Kp) has emerged in Asia and has been associated with complicated bacteremia and septic metastases (liver, CNS, muscles). In New Caledonia, Kp is a major cause of infection and one third of the strains responsible for bacteremia have this hyper mucoid phenotype. The objective of this study was to compare the clinical severity of Klebsella pneumoniae bloodstream infections according to their hyper mucoid character (KpHM) or not (Kpc).

**Patients and methods:** We carried out an observational retrospective study including successively all patients with blood culture positive to *K. pneumoniae* analyzed at Gaston Bourret hospital, Nouméa, between 2013 and 2015. The hyper mucoid character of the strains was defined by the use of the string test followed by molecular analysis to determine the capsular serotype. After a double seizure and anonymization of the data, a bi- and multivariate analysis was carried out according to the mucoid nature or not of the Kp strain and the clinico-biological characteristics of the patients.

**Results:** Fifty-five bacteremic patients were included in the study, 27.3% of the strains isolated were hyper mucoids. Infected populations are comparable and have a high incidence of diabetes in both groups (43.6%). Hypermucoid strains accounted for two thirds of community infections whereas the non-mucoid profile was predominantly found in nosocomial infections (72.5 vs. 33.4%, p = 0.01) There was no significant difference in mortality (KpHM 46.7 vs. Kpc 15%, p = 0.07) but patients with a KpHM strain had a longer hospital stay (73.5 days versus 50.7 days), a higher number of infectious metastases (OR 7.06, CI 95% 1.25–39.64 + p = 0.026), and a longer persistence of positive blood cultures despite the implementation of a suitable treatment (OR 1.41, CI 95 1.01–1.96 + p = 0.045).

**Discussion:** Community-acquired or nosocomial KP bacteraemias represent severe infections where resuscitation is required in half of the cases. This probably explains why there is no difference in mortality between the two groups. However, it can be assumed that the trend of excess mortality in the KpHM group is not significant due to insufficient sample size.

**Conclusion:** Due to their persistent and metastatic nature, bacteremia with KpHM should be considered severe and have to be closely monitored. Prolonged antibiotic therapy should be discussed.

### P-111 Procalcitonin guided antibiotic therapy during severe acute exacerbation of chronic obstructive pulmonary disease requiring mechanical ventilation: a before after study

#### Nakaa Sabrine^1^, Tilouche Nejla^1^, Sikali Habiba^1^, Jaoued Oussema^1^, Gharbi Rim^1^, Fekih Hassen Mohamed^1^, Elatrous Souheil^1^

##### ^1^EPS Taher Sfar, Mahdia, Tunisia

###### **Correspondence:** Nakaa Sabrine - nakaasabrine89@hotmail.com

*Annals of Intensive Care* 2018, **8(Suppl 1):**P-111

**Introduction:** Antibiotic therapy during acute exacerbation of COPD (AECOPD) still controversial and not well supported by clinical evidence. In fact half of these episodes are caused by viruses even during severe episodes with need to ventilator support. Procalcitonin is effective to guide antibiotic therapy during acute exacerbation of COPD without compromising patients’ outcome, its efficacy in the intensive care setting still not well evaluated.

**Patients and methods:** We have conducted in a 10 bed ICU a before after study. During the first period (January 2012-December 2013) patients with AECOPD were included retrospectively and treated with antibiotics according to Anthonisen criteria (control group). In the second period (January 2014–May 2017) antibiotics were prescribed only if the Procalcitonin level was greater than 0.25 ng ml (procalcitonin group).

**Results:** Ninety-two patients were included, 41 in the Procalcitonin group and 52 in the control group. Antibiotics were administered at ICU admission in 7 patients (17%) in the procalcitonin group and in 27 (52%) patients in the control group, p = 0.001. Only 10% of sputum cultures were positive at ICU admission. Time to recovery was similar between the two groups [7 IQR (3–14), 6 IQR (3–10), p = 0.48]. Other patients’ outcomes did not differ between the control group and the procalcitonin group with respectively: the mortality (9.5 vs. 19%, p = 0.17), the incidence of PAVM (11 vs. 17%, p = 0.44) and NIV failure (42 vs. 46%, p = 0.69). Readmission to the hospital at day 90 was significantly higher in the control group (38 vs. 17%, p = 0.03).

**Conclusion:** Using Procalcitonin to guide antibiotic therapy during severe AECOPD can reduce the use of antibiotics without compromising patients’ outcomes.

### P-112 Are standard doses of cefazolin adapted for methicillin-susceptible staphylococcus aureus respiratory infections in critically ill patients with augmented renal clearance?

#### Petit Laurent^1^, Carrié Cédric^1^, Hisz Quentin^1^, D’Houdain Nicolas^1^, Breilh Dominique^1^, Sztark François^1^

##### ^1^CHU PELLEGRIN, Bordeaux, France

###### **Correspondence:** Petit Laurent - laurent.petit@chu-bordeaux.fr

*Annals of Intensive Care* 2018, **8(Suppl 1):**P-112

**Introduction:** In methicillin-susceptible staphylococcus aureus (MSSA) infections, several studies have previously suggested that a pronounced inoculum effect could significantly increase MIC for cefazolin, potentially leading to higher therapeutic failure rates. Although recommended dosing regimen (100 mg kg day continuously) may be suitable for MSSA infections despite high inoculum effect, evidence in patients with augmented renal clearance (ARC) is still limited. Our study aimed to assess whether ARC impacts negatively on cefazolin pharmacokinetic pharmacodynamics (PK/PD) target attainment and clinical outcome in critically ill patients.

**Patients and methods:** Over an 8-month period, all critically ill patients treated by cefazolin for a documented respiratory infection without renal impairment were eligible. Patients who underwent an empiric antimicrobial treatment > 48 h before introduction of cefazolin were not included. During the first three days of antimicrobial therapy, every patient underwent 24-hour creatinine clearance (CrCL) measurements and therapeutic drug monitoring at steady state. The main outcome investigated in this study was the rate of PK PD target non-attainment defined by an unbound concentration < 16 µg ml (MIC value for inoculum > 10^7^ UFC ml). The secondary outcome was the rate of therapeutic failure, defined as an impaired clinical response with a need for escalating antibiotics during treatment and or within 15 days after end-of-treatment.

**Results:** Over the study period, 11 patients were included (33 samples analyzed for therapeutic drug monitoring). In pharmacological analysis, the rate of PK PD target non-attainment was 64%, with a strong association with CrCL (p = 0.001) (Table 1). In clinical analysis, the rate of therapeutic failure was 36% (4 11), with a strong association with inoculum effect (p = 0.02). There was a strong association between therapeutic failure, CrCL > 200 ml min and PK PD target non-attainment adjusted on the inoculum effect (p = 0.04).

**Table 1:** Percentages of PK/PD target attainment for various MIC and CrCL for cefazolin administered continuously (100 mg/kg/day)

	CrCL < 150 ml/min N = 15	150 ≤ CrCL < 200 ml/min N = 22	CrCL ≥ 200 ml/min N = 26
Targeted MIC ≥ 0.5 µg/ml	100 [100–100]	100 [100–100]	100 [100–100]
Targeted MIC ≥ 1 µg/ml	93 [81–100]	86 [72–100]	92 [82–100]
Targeted MIC ≥ 2 µg/ml	80 [60–100]	41 [20–62]	12 [0–24]
Targeted MIC ≥ 4 µg/ml	20 [0–40]	5 [0–13]	0 [0–0]

Results expressed as percentages [95% confidence interval]

**Conclusion:** The recommended doses of cefazolin are probably insufficient in ARC patients with MSSA respiratory infections with high inoculum effect.

### P-113 Better use of Vancomycin for a more effective effect

#### Mootien Joy^1^, Vallat Thibaud^1^, Guillard Daniel^1,^ Kuteifan Khaldoun^1^, Guiot Philippe^1^, Compagnat Fanny^1^

##### ^1^CHU de Mulhouse, France

###### **Correspondence:** Mootien Joy - mootieny@ghrmsa.fr

*Annals of Intensive Care* 2018, **8(Suppl 1):**P-113

**Introduction:** Vancomycin has long been used as the standard therapy of infections due to Methicillin-resistant Staphylococcus aureus (MRSA). The side effects of this drug as well as the increasing resistance and its pharmacodynamics effects have fostered the development of newly active drugs. Nevertheless it is still widely prescribed and it stands as the mostly used comparator in randomized study. An assessment of our medical practice regarding its use may enhance compliance to guidelines so as to promote a better use of Vancomycin.

**Patients and methods:** In our 1600 bed hospital, the incidence rate of MRSA fell from 0.38 to 0.24 per 1000 patient days from 2007 to 2012 whereas the current proportion of MRSA isolates is about 11%. Vancomycin is the most prescribed empirical or targeted antibiotic therapy covering MRSA in our medical intensive care unit of 20 beds even if a shift towards the use of linezolid in nosocomial pneumoniae has been noticed during the last 3 years. Key points regarding the proper use of Vancomycin have been implemented in our antibiotic stewardship program. Moreover courses concerning this topic are provided to our junior doctors. A retrospective review of the quality of antibiotic use has been carried out in 45 courses of Vancomycin therapy and the following criteria have been assessed—indication, dosing schedules, serum levels of Vancomycin, duration of antibiotic therapy and the overall degree of conformity of the prescription.

**Results:** Regarding indication, conformity was observed in42 cases (93%). The dosing schedule was appropriate in 32 cases (71%) only. Of the remaining 13 cases (29%), all of them were not adjusted to the serum concentration and in 2 cases (4%) the general dosing recommendation was not respected. The loading dose was inappropriate in 23 cases (51%) and the proper follow up of serum levels of vancomycin has not been carried out in 20 cases (44%). The duration of antibiotic therapy was in compliance with the protocol in 42 cases (93%) and a slight longer duration was observed in 3 cases (7%). Finally the overall degree of conformity of the prescription was observed in 7 cases (16%) only.

**Conclusion:** Despite the arrival of newly active drugs toward MRSA, Vancomycin may still stand as an appropriate choice if a better use for a more effective and a less toxic effect is observed.

### P-114 Yarrowia lipolytica Fungemia in severe polytrauma-patients requiring intensive care admission A Fortuitous Association!

#### Turki Olfa^1^, Chtara Kamilia^1^, Regaieg Kais^1^, Bahloul Mabrouk^1^, Ben Hmida Chokri^1^, Bouaziz Mounir^1^

##### ^1^CHU HABIB BOURGUIBA, Sfax, Tunisia

###### **Correspondence:** Turki Olfa - olfa.turki.rea@gmail.com

*Annals of Intensive Care* 2018, **8(Suppl 1):**P-114

**Introduction:** Yarrowia lipolytica known as Candida lipolytica is a ubiquitous and opportunistic yeast. Yarrowia lipolytica Fungemia in severe polytrauma-patients has, to the best of our knowledge, never been reported in this specific condition. Between October 2012 and June 2014, we collected 32 *Y. lipolytica* isolates from blood samples in 32 trauma—patients admitted to ICU. The aim of this study was to describe clinical and epidemiological data related to this atypical Fungemia. Moreover, we performed univariate and multivariate analyses to identify the risk factors associated with poor prognosis in the studied population.

**Patients and methods:** Between October 2012 and June 2014, we collected 32 *Y. lipolytica* isolates from blood samples in 32 trauma—patients admitted to ICU. All the patients with positive diagnosis of Yarrowia lipolytica Fungemia were prospectively included in this study.

**Results:** During our period of study, 55 episodes of *Y. lipolytica*-septicemia were diagnosed. Thirty two patients (68%) were admitted for post-traumatic injury and they were all included. The median age of the patients was 39 years (range 18–82 years). The mean SAPSSII score on admission was at 38 ± 11 (median 37.7). Mean SOFA score on ICU admission was at 7.47 ± 3 (median 7). All of the 32 patients had a poly-trauma. The mean duration of onset of candidemia in ICU was 20 ± 13 days (median 18 days). Treatment was based on intravenous amphotericin B (1 mg kg day) in 20 patients (62%), and Fluconazole (400 mg day) in 13 patients (40.6%). Central venous catheters were removed in all cases. The mean delay of antifungual treatment was at 4.9 ± 4 days (median 4 days). The mean antifungual treatment duration was at 13.4 ± 5 days (median 14 days). The evolution of all population groups was marked by the death of 12 patients (37.5%). The comparison between survivors and deceased patients showed that the factors associated with poor outcome were—age, SOFA score the day of candidemia, high SAPSS II score on ICU admission, renal failure and the mean delay of antifungual treatment.

**Conclusion:** Our study suggests that *Y. lipolytica* Fungemia can be observed in poly-trauma patients. It was associated with a poor outcome. The cause of this fungemia is vasucular catheter-associated candidemia. As a consequence, extreme vigilance in the use of medical implants, particularly in intensive care units, is strongly recommended.

### **P-115** Antifungal therapies for suspected *Candida* sp. infections in the ICU: Do they match the guidelines and are the guidelines easy to read?

#### Cadier Gaspard^1^, Accoceberry Isabelle^1^, Ricard Claire^1^, Blanchard Elodie^1^, Vargas Frederic^1^, Hilbert Gilles^1^, Gruson Didier^1^, Boyer Alexandre^1^

##### ^1^CHU Bordeaux, Bordeaux, France

###### **Correspondence:** Cadier Gaspard - gaspardcadier@live.fr

*Annals of Intensive Care* 2018, **8(Suppl 1):**P-115

**Introduction:** Four guidelines are suitable to help physicians decide whether (and which) antifungal therapies should be given to ICU patients suspected of *Candida* sp. infections. The primary objective of the study was to assess the validity of antifungal prescriptions according to each guideline. The secondary objective was to compare the way they are interpreted in the clinical decision making.

**Patients and methods:** We conducted a single-ICU (29 beds) retrospective observational cohort study including all prescriptions of classe III antifungal therapies for suspected or proven *Candida* sp. infections. Therapies included Voriconazole, l-amphoB, echinocandins. The guidelines were IDSA 2016, ESCMID 2012, ATS 2010, SFAR SRLF 2004. Each clinical picture including this treatment, whatever its prophylactic, preemptive, empirical or targeted nature, was presented by the leading investigator to a panel of ICU physician, infectious disease physician, pharmacist, biologist (n = 4). At the first round, they had to independently state whether the antifungal therapy matched with each guideline. Discordances were only known by the leading investigator who applied, via DELPHI method, a second round of interpretation where each investigator was only aware of absence of consensus obtained from one or several guidelines. A third round was necessary in case of persistent discordance and open discussion allowed reaching consensus. The primay endpoint was the overall agreement rate of each guideline. The secondary endpoints were the conensus obtained at 1st, 2nd or 3rd round by each guideline.

**Results:** 3% of ICU admitted patients have received class III antifungal therapies. 42 clinical pictures were analysed. Patients SAPS II was 68 ± 20, septic shock occurred in 71%, ICU mortality rate was 55%. Echinocandins, IA, VZ respectively represented 93.5, 2% of therapies. Indications were prophylactic (7%), empirical (69%), targeted (24%). Most were initiated in ICU (79%). Main results are presented in Table 1.

**Table 1:** Comparison of the four guidelines for the use of antifungal therapy

N = 42	IDSA	ESCMID	ATS	SFAR/SRLF
Applicable first round	34 (81%)	25 (59%)	7 (17%)	26 (62%)
Overall agreement rate (if applicable)	31 (86%)	28 (87%)	9 (100%)	8 (23%)
Consensus rate				
1st round	35 (83%)	27 (64%)	39 (93%)	27 (64%)
2nd round	1 (2%)	3 (7%)	1 (2%)	4 (9%)
3rd round	6 (14%)	12 (29%)	2 (5%)	11 (26%)

**Discussion:** In the SFAR SRLF guideline, the limitation of the echinocandins use to the benefit of ampho deoxycholate explains most of the poor agreement or consensus rate between investigators. The IDSA ESCMID guideline are more helpful to guide indications of empirical treatment which mainly explains their higher rate of both applicability and agreement rate. The rates of agreement do not reflect whether the choice between different class III antifungal therapies is the best or not.

**Conclusion:** The IDSA guideline seems to take a broader spectrum of clinical situations into account, particularly in guiding more precisely indications of empirical treatments. ESCMID or IDSA reach more often consensus at the first reading.

### P-116 Characteristics and prognosis of diaphragmatic paralysis after cardiac surgery in children

#### Denamur Sophie^1^, Roullet Renoleau Nicolas^1^, Soulé Nathalie^1^, Neville Paul^1^, Fakhri Nadine^1^, Chantreuil Julie^1^

##### ^1^CHU de Tours, France

###### **Correspondence:** Denamur Sophie - sophie.denamur.chrutours@gmail.com

*Annals of Intensive Care* 2018, **8(Suppl 1):**P-116

**Introduction:** Diaphragmatic paralysis due to phrenic nerve injury is a known, and potential serious side effect of cardiac surgery in children. We analyzed prevalence, demographics, clinical impact, and management of this paralysis.

**Patients and methods:** We identified retrospectively 20 cases of diaphragmatic paralysis in children, in 1500 cardiac surgeries, between 2006 and 2016, in a University hospital pediatric ICU.

**Results:** The prevalence of diaphragmatic paralysis was 1.3% (20 1500). Median age at cardiac surgery, for children with diaphragmatic paralysis, was 6.1 months (range 4 days to 5 years). The vast majority of operations were open-heart surgery (85%). Arterial switch operation (50%, 10 20), ventricular and or auricular septal defect closure (20%, 4 20), and Blalock-Taussig Shunt (10%, 2 20) were the three top leading causes of diaphragmatic paralysis. The most commonly used approach was median sternotomy. Only two cases of thoracotomy approach have been reported. The diagnosis was suspected for 32% on clinical signs (6 19), and was discovered during a chest X-ray examination for 68% (13 19). Diaphragmatic paralysis was confirmed for all cases with chest ultrasound.39% of patients (7 18) were receiving mechanical ventilation at the moment of the diagnosis. The paralysed hemidiaphragm was left sided in 52% (10 19), and right sided in 48% (9 19). There was no bilateral diaphragmatic paralysis. Hemi-diaphragmatic plication was performed in 30% of the patients (6 20), and median time from cardiac surgery to surgical plication was 21 days (range 8–38 days). Indications for plication were failure to wean from ventilator (100%, 6 6), and respiratory distress (33%, 2 6). Plicatured patients were remarkably younger (median age at cardiac surgery—23 days, range 4–82 days) than non-plicatured patients (7.7 months, range 5 days-5 years). The median ventilation time after plication was 3 days (range 2–6 days). All patients were asymptomatic after diaphragmatic plication. Two patients died (10%). Cause of death was independant from surgical plication (cardiogenic shock, septic shock).

**Conclusion:** Diaphragmatic paralysis is a rare but serious complication of cardiac surgery in children. It commonly occurs after open-heart surgery, and specifically after arterial switch operation. Plicatured patients were younger than non-plicatured patients and needed more frequently a ventilatory support. A closer monitoring may be required for young patients and mechanically ventilated patients. Indeed, both are more likely to be treated by a diaphragmatic plication, reducing mechanical ventilation and intensive care duration.

### P-117 Assessment of Esophageal pressure reliability to estimate pleural pressure in critically ill children

#### Mortamet Guillaume^1^, Nardi Nicolas^2^, Jouvet Philippe^2^, Essouri Sandrine^2^, Emeriaud Guillaume^2^

##### ^1^CHU Grenoble, Grenoble, France; ^2^CHU Sainte-Justine, Montréal, CANADA

###### **Correspondence:** Mortamet Guillaume - mortam@hotmail.fr

*Annals of Intensive Care* 2018, **8(Suppl 1):**P-117

**Introduction:** Despite data demonstrating usefulness of Esophageal pressure (PES) measurement in critically ill patients, several confounding factors that can affect the accuracy of PES measurement have been highlighted in the literature. This study aims to determine whether the values of PES measured in critically ill children are consistent with expectations based on fundamental principles of lung and chest wall mechanics. To reach this goal, we wanted to validate the reliability of PES-based method using a direct PES measurement when compared to direct PPL measurement in situ.

**Patients and methods:** A prospective study. Consecutive children aged between 7 days and 18-year-old admitted to the PICU, intubated and mechanically ventilated were eligible and they reached inclusion if they had at least one chest tube. PPL was directly measured by a pressure transducer connected through a needle inserted into the existing chest tube. PES was measured by both a specific probe (Gaeltec probe) and by the feeding tube after mobilization (PES-FT).

**Results:** 12 patients (median age 3 months (interquartile + 1–4)) were included and exploitable signals were finally available in 9 patients, who were included in the analysis. Most of patients (n = 11) were admitted after cardiac surgery and 5 had a spontaneous breathing activity. Median PES measured by Gaeltec probe and by feeding tube was 2.3 (interquartile + 1.8–5.4) and 7.9 (2.8–9.5) cm H_2_O, respectively. Median PPL measured into the chest tube was 4.0 (0.6–9.6) cm H_2_O. Bland–Altman plots are represented in the figure.

**Conclusion:** Both PPL measured into the chest tube, PES measured by the Gaeltec probe or by the feeding tube are reproducible methods. However, PES measurements does not seem to give a reliable estimation of PPL. Variations observed with these 3 methods are greater than variations considered as reasonable in clinical practice. Future studies are therefore needed before considering esophageal pressure and transpulmonary pressure for management of mechanical ventilation (including PEEP titration) in critically ill children.

### P-118 Management of apnea in bronchiolitis on the pediatric intensive care unit, what is the place of caffeine treatment? A retrospective study

#### Brossier David^1^, Heuze Nicolas^1^, Goyer Isabelle^1^, Jokic Mikael^1^, Villedieu Florence^1^, Letouze Nolwenn^2^

##### ^1^CHU de Caen, Caen, France

###### **Correspondence:** Brossier David - brossier-d@chu-caen.fr

*Annals of Intensive Care* 2018, **8(Suppl 1):**P-118

**Introduction:** Bronchiolitis is a viral infection of the lower respiratory tract affecting children under 2 years of age. Three to 11% of bronchiolitis episodes require hospitalisation in the paediatric intensive care unit (PICU). Apnea is one of the most commonly encountered complications (1.6 to 5%). Considering the established use of caffeine in the treatment of apnea of prematurity, some PICU teams have extrapolated this data to apnea in bronchiolitis and consider caffeine as a part of its management. However, for there is a lack of data regarding the use of caffeine in bronchiolitis related apnea, there is no recommendation or standard of care across PICUs worldwide. The objective of this study was to describe the management of bronchiolitis related apnea in the PICU and explore the effect of caffeine on clinical outcomes as well as its potential adverse effects in this indication.

**Patients and methods:** We conducted a retrospective study in the PICU of the level 3 university hospital of Caen between January 1st, 2009 and December 31st, 2016. All children under 1 year old with a diagnosis of bronchiolitis related apnea admitted in the PICU were included. Patients were allocated in one of the two groups (control or caffeine group) depending on the administration of caffeine.

**Results:** Fifty four infants were included. Caffeine treatment was administered in 49 patients (91%). Patients’ characteristics were similar between groups. Overall median age was 24 days [18–38] and median weight was 3260 g [2810–3810]. Respiratory syncytial virus was identified in 38 infants (70%). An initial caffeine citrate loading dose of 20 mg kg was usually administered, followed by a 5 mg kg day maintenance dose, for a median treatment duration of 3 days [1–6]. Therapeutic management (invasive and non-invasive ventilation, nutrition support) and clinical outcomes (death, length of stay) were similar between groups. There was no difference in potential caffeine adverse effects between groups or within the caffeine exposed group pre and post-caffeine administration.

**Conclusion:** Caffeine treatment of bronchiolitis related apnea seems to be a standard practice in our PICU. Our study failed to show any influence of caffeine on clinical outcomes in this indication when compared with a small number of patients. Further studies are needed to assess the efficacy and safety of caffeine treatment in this indication as well as the appropriate treatment regimen as pharmacokinetic data suggest that higher dose could be of great interest in this non-prematurely born population.

### P-119 Incidence and Risk Factors of Catheter-Associated Thrombosis in Critically Ill Children

#### Fareh Mohamed Karim^1^, Malouch Abir^1^, Marzouk Mahmoud^1^, Nasri Oussama^1^, Tounsi Mariem^1^, Ben Khalifa Sonia^1^

##### ^1^Tunis, Tunisia

###### **Correspondence:** Fareh Mohamed Karim - fareh.karim@gmail.com

*Annals of Intensive Care* 2018, **8(Suppl 1):**P-119

**Introduction:** During the last decade, many authors have raised awareness concerning the increasing rate of venous thromboembolism (VTE) in critically ill children [1]. The presence of central venous catheter (CVC) is one of the most important risk factor for venous thrombosis in children [2]. The purpose of this study was to analyze incidence and risk factors for catheter-related thrombosis in children admitted in our Pediatric Intensive Care Unit (PICU).

**Patients and methods:** All children aged less than 2 years, admitted in the PICU from January 2016 to June 2017, and receiving at least one tunneled CVC, were included in our retrospective study. Those with venous thrombosis unrelated to CVC placement were excluded. Catheter-associated venous thrombosis (CAVT) was confirmed using Doppler ultrasonography. Demographic data (age, gender, weight), underlying diagnosis, catheter type and characteristics (number of lumen, diameter, insertion site), indication of CVC placement and occurrence or not of an infection, were collected and analyzed using IBM SPSS Statistics for Windows (Version 22.0. Armonk, NY—IBM Corp). Pearson linear correlation coefficient was performed to measure correlation between catheter-associated venous thrombosis and risk factors. P-Value < 0.05 was considered significant.

**Results:** Seventy six children were included. Mean age was 14 days (0–540). During PICU stay, 51 (67.1%) children developed an infection, while 17 (22.36%) of them developed a CVC-related infection. Incidence rate of CAVT was 12 (15.79%). The mean diagnostic delay duration was 26 days (13–51). There was a significant correlation between CAVT and catheter infection (p < 0.05). This correlation has not been founded with regard to other risk factors (Table 1).

**Table 1:** Correlation between CAVT and risk factors

	Total (n = 76)	CAVT + (n = 12)	CAVT—(n = 64)	p
Age (days)	14 (0–540)	15(0–120)	14.15 (0–540)	
Gender				
Male	47 (61.84%)	7 (58.3%)	40 (62.5%)	NS
Female	29 (38.16%)	5 (41.67%)	24 (31.57%)	
Occurrence of an infection	51 (67.10%)	12 (100%)	39 (60.93%)	*0.006*
Germs KP ESBL	24 (31.57%)	6 (50%)	18 (28.12%)	NS
Candida *spp*	20 (26.31%)	12 (100%)	8 (12.5%)	
Insertion site Internal jugular vein	72 (94.73%)	12 (100%)	60 (93.75%)	NS
Others	4 (5.27%)	0	4 (6.25%)	
CVC size 2.7	33 (43.42%)	6 (50%)	27 (42.18%)	NS
4.2	42 (55.26%)	6 (50%)	36 (56.25%)	
Reason for admission				NS
Esophagus atresia	32 (42.10%)	4 (33.3%)	28 (43.75%)	
Small intestine atresia	17 (22.36%)	2 (16.7%)	15 (23.43%)	
Others	13 (17.10%)	6 (50%)	7 (10.93%)	
Indication of CVC insertion				NS
Parenteral nutrition	47 (61.84%)	7 (58.30%)	40 (62.5%)	
Vasopressive drugs	45 (59.21%)	9 (75%)	36 (56.25%)	
Transfusion	13 (17.10%)	4 (33.30%)	9 (14.06%)	

CAVT: Catheter-associated venous thrombosis; NS: Nonsignificant

**Conclusion:** We found higher incidence of CAVT in this trial, compared to previous studies, with a significant correlation to catheter infections. This might be explained by the higher rate of catheter infectious complication during hospital stay in our PICU.

**References**

1. Raffini L. and al. Dramatic increase in venous thromboembolism in children’s hospitals in the United States from 2001 to 2007. Pediatrics 2009
2. Monagle P. and al. Antithrombotic therapy in neonates and children. Chest 2012.

### P-120 Acute renal neonatal failure: What is the prognosis?

#### Batouche Djamila-Djahida^1^, Zerhouni Amel^1^, Tabeliouna Kheira^1^, Boudjahfa Samir^1^

##### ^1^Oran, Algeria

###### **Correspondence:** Batouche Djamila-Djahida - khedidjabatouche@yahoo.fr

*Annals of Intensive Care* 2018, **8(Suppl 1):**P-120

**Introduction:** Studies of neonatal acute renal failure have been fragmented in ORAN and still remain a problem in taking care of in our institution due to a frequent lack of technical platforms. Our objective is to analyze the etiologic and prognostic characteristics of newborns (NB) admitted for acute renal failure (ARF) at the Oran University Hospital.

**Patients and methods:** Retrospective study over a period of (1997–2015), in the pediatric intensive care unit department at the Oran University Hospital gathering the neonatal ARF diagnosed on average 1.5 days of life in the pediatric departments. The parameters studied—age of newborn, renal function at admission, RIFLE criterion, causes of acute renal failure, management, the biological monitoring of renal function, prognosis.

**Results:** We took care of 21 cases. The age at admission varied between 2 and 28 days of life. The majority of newborns were born at term. At admission, renal function was disrupted with a RIFLE (R) criterion in 04 cases, (I) in 10 cases and (F) in 07 cases. A context of perinatal suffering was found in 08 cases. The causes of ARF were infectious (08 cases), renal vein thrombosis (1 case), perinatal asphyxia (02 cases), urinary tract obstruction (06 cases), aminoglycoside nephrotoxicity (2 cases) hypovolemia on loss syndrome of salt (01 cases), abdominal hyperpressure on omphalocele (1 case). Peritoneal dialysis was indicated 10 times. A Lifting of surgical obstacle in 7 cases. The evolution was marked by a death rate in 7 newborns, total reversibility of renal function in 6 newborns, and in 8 cases a partial recovery of renal function was noted.

**Discussion:** Multiple factors may contribute to the occurrence of (ARF in newborns) including infection, hypovolemia and perinatal asphyxia. Sequellar renal failure is attributed to renal malformation. Acute renal failure of septic origin is dependent on a high mortality rate in newborns.

**Conclusion:** The mortality rate remains high in our study justifying pediatric multidisciplinary management and improvement of technical platforms. Monitoring of renal function should be regular in order to slow down any progression of chronic renal failure in this type of patients with a Newborn acute renal failure.

**Reference**

- Gupta BD., Sharma P., Renalfailure in asphyxiated neonates. Indian Pediatr 2005 + 42-928 34.

### P-121 Near Infrared Light in peripheral venous access in pediatric anesthesiology, Pediatric Intensive Care Unit (PICU) and Emergency Department

#### Delarue Gérald^1^

##### ^1^Armentières, France

###### **Correspondence:** Delarue Gérald - avicenne2008@hotmail.fr

*Annals of Intensive Care* 2018, **8(Suppl 1):**P-121

**Introduction:** The peripheral vein installation is an invasive procedure that is difficult in anesthesia, ICU and pediatric emergencies. A device using infrared light is available to professionals in Pediatric Intensive Care, it is the Accuvein*. The main objective of this study is to describe the indications, conditions of use and the success rate of Accuvein in PICU. The secondary objective is to quantify the team’s satisfaction and to explore correlations between success and difficulty sensed before using the Accuvein.

**Patients and methods:** Between January 1 and March 31, 2016, we studied the use of Accuvein with 26 children admitted to PICU to Regional and Universitary Hospital of Lille. Were included Children aged 1 month to 17 years required the installation of a peripheral vein and caregivers sought to make use of Accuvein*.

**Results:** A total of 26 children admitted to PICU were included in the study and the average age was 39 months, the median age was 14 months. Twelve children were younger than 1 year (46%), 8 between 1 and 2 year of age (31%), 6 more than 3 years (23%). Among 26 patientsenrolled, there were 15 (58%) peripheral venous access success and 11 failures (42%) under Accuvein. The average satisfaction rating of the assistance provide by Accuvein vas 5.24 10.

**Conclusion:** Despite having a limited group of 26 patients, our study has collected a lot of data. Clinical assessment of PICU operators is adapted to the score DIVA (p = 0.019). This study shows that the use of accuvein did not ensure the installation of peripheral vein, but still there are limits and a part of failure although the implementation of this resort tool in case of difficulty to locate veins. The number of puncture attempts did not seem t obe reduced with the use of accuvein.

### P-122 HLA-DR expression in neonates after cardiac surgery under cardiopulmonary bypass

#### Chenouard Alexis^1^, Cottron Nicolas^1^, Bourgoin Pierre^1^, Joram Nicolas^1^, Braudeau Cécile^1^, Josien Régis^1^, Roquilly Antoine^1^, Asehnoune Karim^1^

##### ^1^Hopital Mère Enfant, Nantes, France

###### **Correspondence:** Chenouard Alexis - alexis.chenouard@chu-nantes.fr

*Annals of Intensive Care* 2018, **8(Suppl 1):**P-122

**Introduction:** The diminished monocyte human leukocyte antigen-DR (HLA-DR) expression on cell surface is proposed as a reflection of immunosuppression in critically ill patients. To date, HLA-DR has been assessed in adults as a predictor of septic complications after various injuries, notably after cardiac surgery under cardiopulmonary bypass (CPB). However, to our knowledge, no study has specifically focused on HLA-DR expression among neonates undergoing cardiac surgery under CPB, which have a high risk of nosocomial infection (NI). In this pilot study, we investigated the kinetic of monocyte HLA-DR expression in this population and described the relationship between monocyte HLA-DR expression and the subsequent development of NI.

**Patients and methods:** Blood samples were collected from neonates preoperatively at line insertion, and 1, 2, 3 and 4 days after the end of CPB. The number of HLA-DR molecules per monocyte (AB c) was determined by flow cytometry on whole blood using a Quantibrite phycoerythrin fluorescence quantitation kit (BD Biosciences). NI were defined based on the Center of Disease Control (CDC) and National Nosocomial Infections Surveillance criteria and were prospectively recorded during the PICU stay or within 30 days after surgery.

**Results:** Nine patients were included with a median RACHS-1 operative risk score at 4 [3–6], a median age of 12 days [6–23] and a median weight of 2.9 kg [2.0–4.0]. The median duration of CPB was 164 min [54–272]. There was a significant reduction in HLA-DR expression for the first two postoperative days, as compared to preoperatively (Kruskal–Wallis test, p = 0.004). Moreover, three patients (33%) displayed an episode of NI 3 days, 4 days and 8 days after CPB. Interestingly, we observed that these 3 patients had a lower HLA-DR expression at day 4, as compared to patients without NI (4257 AB c [2220–5895] vs. 14947 AB c [9858–16960] + Mann Witney test, p = 0.04).
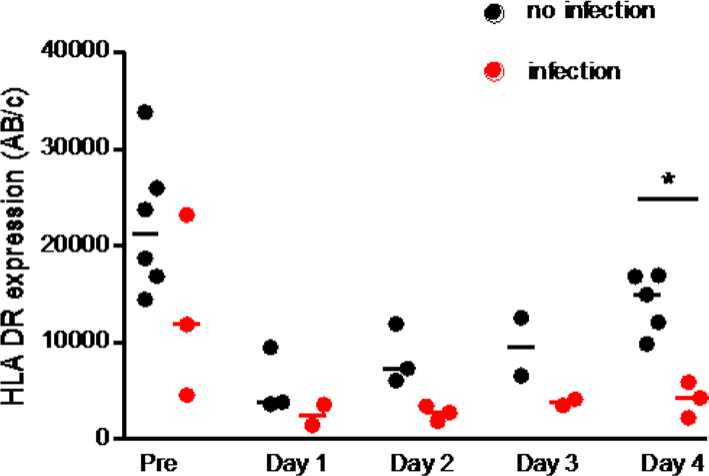


**Conclusion:** We report here that neonates had a reduction in HLA-DR expression after CPB, and those with prolonged decreased HLA-DR in the early postoperative period (Day 4) could represent a subpopulation at greatly increased risk of later NI. If confirmed in a larger cohort of patients, our findings could indicate that HLA-DR may be a useful biomarker of immunosuppression after CPB in neonates.

### P-123 Non-traumatic hemorrhagic stroke (NTHS) in comatose children: epidemiological features and clinical presentation

#### Vergnaud Estelle^1^, Terzi Eleonora^1^, Baugnon Thomas^1^, Meyer Philippe^1^, Orliaguet Gilles^1^

##### ^1^Hôpital Necker, Paris, France

###### **Correspondence:** Vergnaud Estelle - estelle.vergnaud@gmail.com

*Annals of Intensive Care* 2018, **8(Suppl 1):**P-123

**Introduction:** There are few studies describing the epidemiological features and clinical presentation of non-traumatic hemorrhagic stroke (NTHS) (1, 2). This retrospective observational study aims at describing epidemiological aspects and clinical presentation of NTHS in pediatric comatose patients.

**Patients and methods:** All the patients admitted over a 10 year-period (2005 to 2015) into our service for NTHS with a Glasgow score (GCS) ≤ 8 or a GCS drop > 4 points in less than 6 h were included. Data collected were: demographic data, clinical signs and symptoms and Glasgow Outcome Scale (GOS). Data are median (range) or number (%).

**Results:** Thirty-eight patients, 121 months (82–142) of age, with a sex ratio (M F) of 1.53, were included. The etiologies of NTHS were—rupture of cerebral arteriovenous malformation (58%) or aneurysm (8%) or cavernoma (5.2%), brain tumor (18.4%), factor V deficiency (2.6%), Moya–Moya disease (2.6%) and no cause in 5.2%. Warning signs were—headaches and vomiting (65.8%), convulsions (34.2%), motor deficit (34.2%), transient loss of consciousness (47.4%). GCS was 7 (6–9) on prehospital scene and 3 (3–4) on hospital admission. Clinical signs prevalence is reported in Table 1. The time between warning signs and coma onset was < 3 h in 16% and < 6 h in 60%. Overall mortality was 15.8% and no prehospital death was reported. All patients with back (or reverse) flow on transcranial Doppler on hospital admission have died. The GOS score was 3 (3–3) at discharge (M0) and 4 (3–5) at 6 months (M6).

**Table 1:** Clinical signs of non-traumatic hemorrhagic stroke (NTHS) in children

Clinical signs (%)	Prehospital	In hospital
Coma	57.9	100
Pupils size		
Anisocoria	28.4	21
Bilateral mydriasis	5.2	18.4
Generalized convulsions	21.0	18.4
Motor deficit	42.1	55.2
Circulatory failure	23.6	39.5
Respiratory failure	21.0	100

**Conclusion:** The diagnosis of NTHS should be suspected from warning signs, including sudden occurrence of headaches, vomiting, convulsions, motor deficit or transient loss of consciousness, in the lack of obvious head trauma. The observed overall mortality was significantly lower than in previous series (3), and may be related to an aggressive on site resuscitation, and a transport of patients in a neurosurgical center as soon as possible (3).

**References**

1. Yock-Corrales A et al. Acute childhood arterial ischemic and hemorrhagic stroke in the emergency department. Ann Emerg Med 2011 + 58—156–163.
2. Warren L. Childhood hemorrhagic stroke—an important but understudied problem. J Child Neurol 2011 + 26—1174–1185.
3. Meyer PG et al. Emergency management of deeply comatose children with acute rupture of a cerebral arteriovenous malformation. Can J Anaesth 2000 + 47—758.

### P-124 Individual’s impediments to weaning from mechanical ventilation: a prospective, single center study

#### Sztrymf Benjamin^1^, Dupont Julie^1^, Kudela Agathe^1^, Delval Paul^1^, Sabri Sami^1^, Tadbiri Sarah^1^, Prat Dominique^1^, Jacobs Frederic^1^, Hamzaoui Olfa^1^, Demars Nadege^1^, Millereux Maude^1^, Trouiller Pierre^1^

##### ^1^Hôpital Henry Mourier, Clamart, France

###### **Correspondence:** Sztrymf Benjamin - benjamin.sztrymf@aphp.fr

*Annals of Intensive Care* 2018, **8(Suppl 1):**P-124

**Introduction:** Weaning from the ventilation is a crucial moment in the ICU stay. Because of the risks of mechanical ventilation (MV), such as ventilator-associated pneumoniae, it is recommended to begin the weaning process as soon as weaning criteria occurs [1]. However, extubation is also a hazardous period, with 5 to 15% of subsequent respiratory failure requiring reintubation, harboring a dismal prognosis [2]. International guidelines display the criteria triggering the extubation. Nevertheless, the physician in charge eventually takes the decision to extubate. In this regard, there could be variations from an individual to another. The main goal of our study was to identify the perceived impediments to MV weaning among physicians, from intubation to extubation.

**Patients and methods:** Prospective single center study in a 12 bed university ICU. All patients admitted between February and May 2017 and undergoing MV were included. We daily registered the existence of the criteria recommending a spontaneous breathing trial (SBT), the occurrence of a SBT, the items recommending postponing extubation, and the occurrence of an extubation. The estimated reasons for all the aforementioned decisions were asked to the physician in charge.

**Results:** 25 patients were included, gathering 298 days of MV and 42 SBT. The average duration of MV was 8.25 ± 9.4 days. There was one extubation failure requiring reintubation. There were 9 SBT failures. In 9 cases, SBT was a success but did not lead to extubation because of hypotonia, weak cough, subsequent respiratory failure, hemorrhagic bronchial secretions, hemodynamic instability, absence of weaning criteria, drowsiness (all the aforementioned n = 1), post SBT hypercapnia (n = 2). 23 out of the 42 SBT (55%) were done while one or several weaning criteria were absent. Impediments to weaning trials were different according to the time lag since ICU admission, with fluid overload, muscular weakness and persistent need for assist control ventilation settings being the most frequent reasons advocated after 7 days (figure). No objective assessment of muscular or cough strength was performed at any time, neither was monitored the RR Vt, vital capacity or inspiratory pressure.
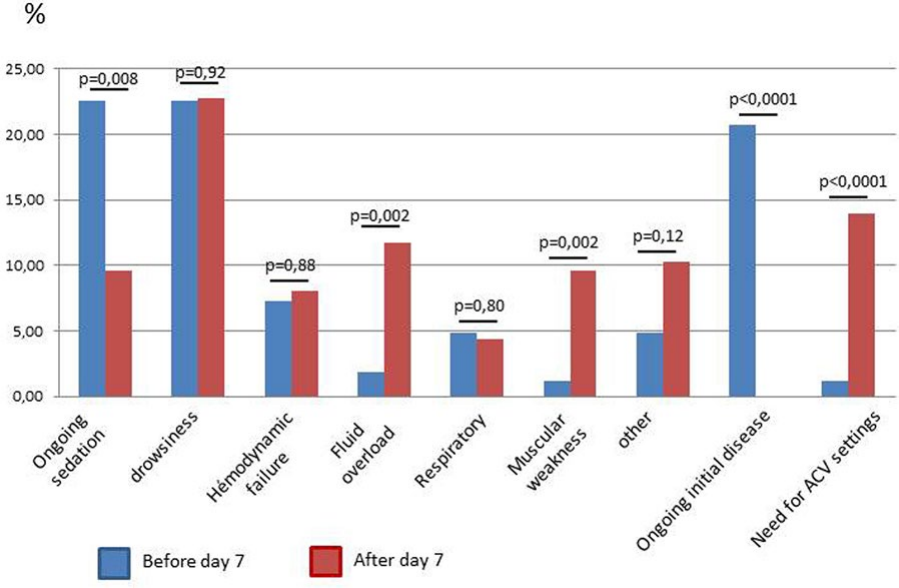


**Conclusion:** Reasons for postponing a weaning trial or an extubation depended on the time lag from intubation, and were not always supported by objective criteria. Weaning procedure may improve our practice in the field.

### P-125 Tracheotomy in ventilatory weaning

#### Khaleq Khalid^1^, Mghari Majida^1^, Makhchoune Marouane^1^, Bouhouri M. A^1^, Hamoudi D^1^, Nciri A^1^, Alharrar R^1^

##### ^1^CHU Ibn Rochd, Casablanca, Maroc

###### **Correspondence:** Khaleq Khalid - khaleq20@gmx.fr

*Annals of Intensive Care* 2018, **8(Suppl 1):**P-125

**Introduction:** Tracheotomy is a commonly performed procedure in intesive care units, when patient need long term mecanical ventilatory, or in failure of ventilatory weaning.

**Patients and methods:** Our study is a retrospective one in a 2 years period, starting in January 2015 to December 2016, collecting data of 100 patients admitted in department of surgical intensive care unit, 2 groups of 50 patients, Group «T» for tracheotomy and Group «I» for tracheal intubation.

**Results:** Mean age of our patients was the same between Group «T» and «I» wich was 36 years old, we had 75 men and 15 women, 91% of them were in ICU for neurological and neurosurgical diseases and 9% for respiratory diseases. Acute phisiology score SAPS II was around 27 on both groups. Tracheotomy was performed around the 5th and 6th day in the ICU, with a range between 2nd and 9th day. As early complications we had 1 case of pneumothorax, 2 cases of subcutaneous emphysema and 5 cases of local infection. Late complications was essentially ICUAP intensice care unit acquired pneumoniae—60% in Group «T» and 68% in Group «I» p = 0.405, wich had increased the need of artificial ventilation by 6 days in Group «T» and 10 days in Group «I». First attempt of ventilatory weaning was successful in 17 cases in Group «T» and 14 cases in Group «I». Finally we had successful ventilatory weaning in 28 patients in Group «T» and in 24 patients in Group «I». The mean period of artificial ventilation was 16 days in Group «T» and 20 days in Group «I». Total stay was arround 32 days in Group «T» and 36 days in Group «I» .

**Conclusion:** In our study, it appears that tracheotomy can give best ventilatory weaning conditions than tracheal intubation.

### P-126 With the same O2 flow, FiO_2_ decreases during non-invasive ventilation (NIV) with a home ventilator (HV) versus nasal normobaric long-term oxygen therapy (LTOT)

#### Goutorbe Philippe^1^, Cardinale Mickael^2^, Castagna Olivier^1^, N Guyen Cedric^1^, Cungi Pierre-Julien^1^, Esnault Pierre^1^

##### ^1^HIA Ste Anne, Toulon, France; ^2^IRBA, Toulon, France

###### **Correspondence:** Goutorbe Philippe - philippegoutorbe@me.com

*Annals of Intensive Care* 2018, **8(Suppl 1):**P-126

**Introduction:** Non-invasive ventilation should be considered in selected patients with chronic obstructive pulmonary disease (COPD) (GOLD 2017). Home ventilators are sometimes used in intermediate care (because of cost effectiveness or to adapt the patient before home return). Bench and rare clinical studies showed that FiO_2_ decreases with occurrence of leaks but none of the studies compares FiO_2_ during normobaric LTOT with FiO_2_ during NIV. We hypothesize that NIV with home ventilators induces a loss of FiO_2_ compared to normobaric oxygen therapy.

**Patients and methods:** To compare–Pharyngeal FiO_2_ with nasal O2 supply -And FiO_2_ during NIV at the same O2 flow with a home ventilatorIn ICU and intermediate care patients, we measured consecutively-Pharyngeal FiO_2_ (G5 Philips side stream FiO_2_) with O2 supply through a nasal cannula at basal flow (flow needed by patient). In order to compare FiO_2_ we measured FiO_2_ with 75, 50 and 25% of basal Flow -FiO_2_ during a 30 min NIV trial (pressure support ventilation + Vivo 50, Breas), using a facial mask (Comfort Gel Philips) with O2 flow set as the basal flow above.

**Results:** We performed 15 sequences in 9 patients. During NIV FiO_2_ decreased from 34.55% (± 10.92) to 25.44% (± 1.35) p < 0.0001 (Wilcoxon test) Fig. 1. During the 30 min NIV trial, we also correlated FiO_2_ with each corresponding pharyngeal FiO_2_ with the different normobaric O2 flows-Time with FiO_2_ corresponding to 100–75, 75–50, 50–25 and below 25% of the basal flow were respectively 0, 48, 45 and 7%. To sum up, with NIV the resulting FiO_2_ was corresponding to a 50% O2 withdrawal for half of the time.

**Fig. 1:**
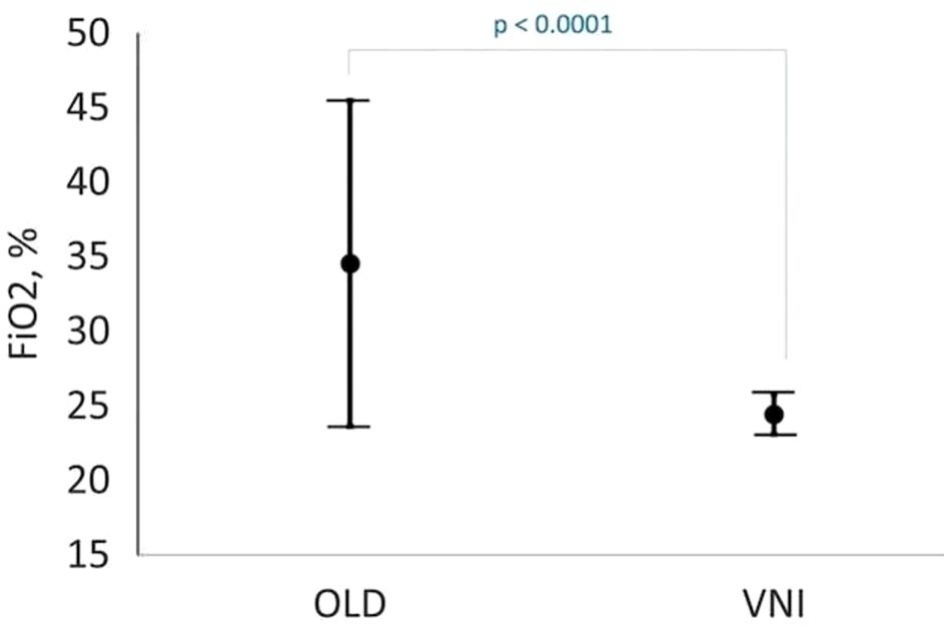
Comparison of FiO_2_ (%) with oxygen standard or noninvasive ventilation

**Conclusion:** Compared to normobaric LTOT the FiO_2_ is lower during NIV with the same O2 flow. Compensation for intentional and non-intentional leaks and so an increase of air flow despite a constant O2 input might explain this. In intermediate care the use of HV for NIV may be interesting alternative in which case the clinician must keep in mind that the FiO_2_ decreases compared to standard oxygen therapy. Concerning home usage we hypothesize that this partial removal of O2 treatment could contribute to the poor results of NIV in chronic COPD.

### P-127 Prolonged ICU stays and difficult-to-wean-patients: first year of experience in a french Post ICU Rehabilitation Center

#### Morawiec Elise^1^, Delemazure Julie^1^, Dres Martin^1^, Mayaux Julien^1^, Dangers Laurence^1^, Similowski Thomas^1^, Demoule Alexandre^1^

##### ^1^Hôpital Pitié Salpêtrière, Paris, France

###### **Correspondence:** Morawiec Elise - elisemorawiec@gmail.com

*Annals of Intensive Care* 2018, **8(Suppl 1):**P-127

**Introduction:** In February 2016, we opened a 12 beds-post ICU rehabilitation center (Service de Rééducation Post Réanimation, «SRPR»), dedicated to weaning from mechanical ventilation and global post ICU rehabilitation. Objectives—description of the characteristics and main outcomes of the patients admitted over the first year of activity.

**Patients and methods:** retrospective analysis of data extracted from the medical files.

**Results:** 96 patients were admitted 100 times in the unit over its 1st year, from 34 different ICUs (median duration of stay in the ICU 38.5 days (IQR 29–61)). 86% were ventilated (11% with NIV). 85% had a tracheostomy. 64% had ICU acquired weakness + 5% were able to walk. An underlying chronic respiratory disease was present in 53% of cases. 18% were obese. Difficult weaning was found to have one or several respiratory components in 77% of cases (including 20 post surgery diaphragmatic paralysis), cardiac in 34%, neurologic in 18%. Significant complications occured in 60% of cases. Median duration of stay was 19.5 (12–29.5) days. Ten patients died in the unit, 13 patients were re-transferred in the ICU, where 6 of them died. Over half of the patients were discharged at home, in a rehabilitation unit (SSR) or in a hospital ward awaiting a rehabilitation bed. The remaining 24%, that still needed some form of medical or surgical care were discharged in the ward (Fig 1). In intention to treat, successful weaning from invasive ventilation was obtained in 70% of patients. Of the patients discharged alive from the unit after completing the rehabilitation program (n = 74), 60% were completely weaned from mechanical ventilation, 35% were discharged with NIV or CPAP + 4 patients (5%) were considered not weanable from invasive ventilation + decanulation of tracheostomy was obtained in 82% of cases + 87% of the patients could walk.

**Fig. 1:**
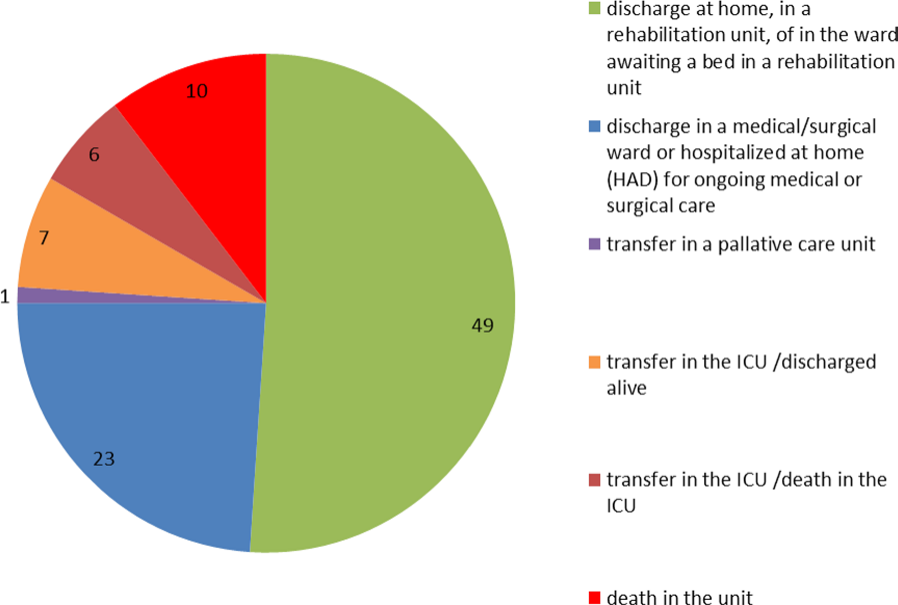
Outcome of patients

**Conclusion:** SRPRs offer a new concept of care for difficult to wean patients, with promising Results.

### P-128 Outcome of patients classified in group 3 of the WIND classification

#### Beduneau Gaëtan^1^, Pham Tài^2^, Schortgen Frédérique^3^, Piquillioud Lise^1^, Zogheib Elie^4^, Jonas Maud^1^, Runge Isabelle^1^, Grelon Fabien^1^, Guitard Pierre Gildas^1^, Terzi Nicolas^1^, Grangé Steven^1^, Frat Jean Pierre^1^, Barberet Guillaume^1^, Mercat Alain^1^, Richard Jean-Christophe^1^, Brochard Laurent^1^

##### ^1^CHU Rouen, Rouen, France; ^2^Hôpital Saint Michael, Toronto, CANADA; ^3^CHIC Créteil, Créteil, France; ^4^CHU Amiens, Amiens, France

###### **Correspondence:** Beduneau Gaëtan - gaetan.beduneau@chu-rouen.fr

*Annals of Intensive Care* 2018, **8(Suppl 1):**P-128

**Introduction:** Scarce data about patients with prolonged weaning from the mechanical ventilation are available in the literature. Patients without successful weaning 7 days after their first weaning attempt were classified in the group 3 of the Weaning according New Definition (WIND) classification (1). We here describe specific data concerning weaning and hospital evolution of group 3 patients included in this prospective cohort.

**Patients and methods:** Among the 2709 patients included in the WIND study, 235 were classified in the group 3. Additional data concerning comorbidities, cause of weaning failure and hospital evolution were collected for 213 (91%) of these 235 patients.

**Results:** These 213 patients had median [interquartile range] duration of invasive mechanical ventilation of 18 [15–28] days and 3 [2–4] separation attempts. Etiology of ICU hospitalization was medical in 171 (80%). They had a COPD in 49 (23%), hearth disease in 33 (15%) and immusoppression in 31 (15%). We noticed a mean SAPS II of 53 ± 18, a mean SOFA D1 of 8 ± 4 and D3 of 7 ± 4. Tracheostomy for weaning was performing in 55 (26%). At the end of their follow-up, 144 (68%) were still alive—44 (21%) were still tracheostomized, 4 still intubated and ventilated, 8 (4%) treated with VNI and 88 (41%) were extubated (or decannulated) and breathed without assistance. Among the 44 patients still tracheostomized at the end of the follow-up, 14 (32%) were still ventilated (permanently for 8 of them, and partially for 6) and 30 (68%) had spontaneous breathing through their tracheostomy. These patients had a total ICU length of stay of 30 [20–42] days. The destination at discharge from the ICU is known for only 49 of the 144 survivors—24 (49%) in medical ward, 15 (31%) in intermediate care units, 4 (8%) in sub acute care, 3 (6%) in ICU and 3 in surgical ward.

**Conclusion:** A third of the 213 patients of the WIND study classified in group 3 and with available additional data died in hospital in 2 months following intubation. At the end of the follow-up, 41% had spontaneous breathing without assistance, and 21% were still tracheostomized. Among these tracheostomized patients, one third still required mechanical ventilation. (1)—Béduneau, G., Pham, T. and co(2017). Epidemiology of weaning outcome according to a new definition. The WIND study. AJRCCM, 195(6), 772–783.

### P-129 Impact of anemia in long term prognosis of patients with COPD exacerbations

#### Ouanes Islem^1^, Hraiech Kmar^1^, Adhieb Ali^1^, Bouker Nouha^1^, Tlili Meriem^1^, Hammouda Zaineb^1^, Boukadida Sana^1^, Ouni Amal^1^, Nouira Wiem^1^, Zorgati Hend^1^, Dachraoui Fahmi^1^, Ouanes-Besbes Lamia^1^, Abroug Fekri^1^

##### ^1^CHU Fattouma Bourguiba, Monastir, Tunisia

###### **Correspondence:** Ouanes Islem - ouanes.islem@gmail.com

*Annals of Intensive Care* 2018, **8(Suppl 1):**P-129

**Introduction:** COPD patients have often polyglobulia because of associated hypoxemia especially in patients at the stage of chronic respiratory failure. We recently reported that anemia was present in 36% of patients with severe AECOPD admitted to ICU without impact on short-term prognosis. The aim of the present study was to assess the long-term impact of haemoglobin (Hb) levels on outcomes of AECOPD patients.

**Patients and methods:** In a prospectively collected database including consecutive patients admitted between 2007 and 2015 for AECOPD in our ICU. Long-term status of patients following the first ICU admission (surviving or deceased) has been verified by consulting the civil status registers. Anemia was defined according to WHO criteria—Hb < 13 g dl in males + Hb < 12 g dl in females. Long-term survival was assessed by Kaplan–Meier curve.

**Results:** The cohort included 213 patients (median age 67, median pH 7.30, 84.5% males, NIV as first ventilator mode in 87.4%). Anemia was observed in 77 of the 213 patients (36.2%) with median haemoglobin levels at 10.8 and 14.5 g dl, in patients with and without anemia, respectively. Anemia was associated with significantly lower 2 years survival (Log Rank p = 0.043) (Fig. 1). The final model included age, SAPS II score, comorbidities, home oxygen therapy, initial ventilatory mode, NIV failure and haemoglobin levels. Multivariate analysis identified age (OR 1.05 per year + IC 95% 1.01–1.10 + p = 0.028), home oxygen therapy prior to exacerbation (OR 2.38 + IC 95% 1.09–5.12 + p = 0.029), intubation at ICU admission (OR 6.97 + IC 95% 2.31–21.03 + p < 0.001), NIV failure (OR 11.5 + IC 95% 2.75–47.6 + p < 0.001), and haemoglobin (OR 1.20 per decrease of 1 g dl + IC 95% 1.01–1.44 + p = 0.034) as independently associated factors with 2 years mortality .

**Fig. 1:**
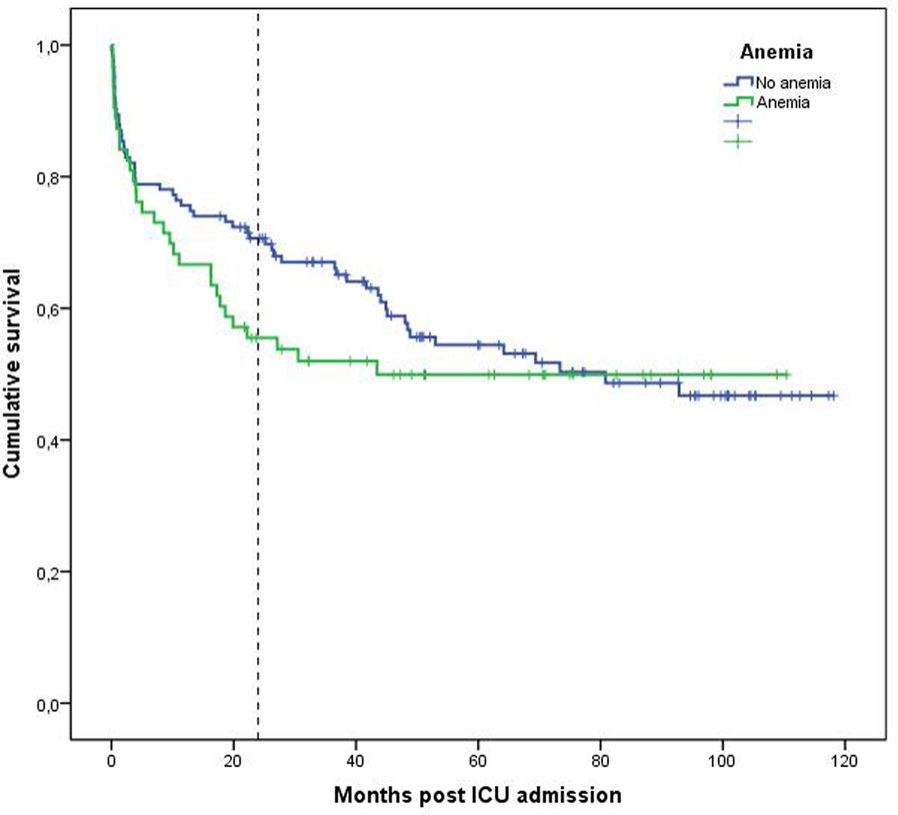
Kaplan Meier curves showing survival of COPD patients with anemia (green) or not (blue).

**Conclusion:** Our study suggests that anemia is associated with 2-year outcome in patients admitted for AECOPD.

### P-130 Tracheostomy in intensive care patients, a common practice at risk of complications

#### Hypolite Lucie-Marie^1^, Cravoisy-Popovic Aurélie^1^, Gibot Sébastien^1^, Conrad Marie^1^, Lemarié Jérémie^1^, Bollaert Pierre-Edouard^1^, Beaumann Cédric^2^, Rousseau Hélène^1^

##### ^1^CHRU Nancy, Nancy, France

###### **Correspondence:** Hypolite Lucie-Marie - lmhypolite@yahoo.fr

*Annals of Intensive Care* 2018, **8(Suppl 1):**P-130

**Introduction:** Tracheostomy is a common procedure in Intensive Care Unit (ICU) patients requiring prolonged mechanical ventilation and hastens weaning from ventilatory support. Tracheostomy improves patient comfort compared with standard intubation without survival benefits. However, this procedure is associated with a non-negligible morbi-mortality rarely described in the literature. The objective of our study was to describe the characteristics of the tracheostomized population and complications secondary to tracheostomy.

**Patients and methods:** Our observational, retrospective study included all tracheostomized patients from January 1, 2005 to December 31, 2015, admitted to the medical ICU department of Central Hospital of CHRU Nancy’s. We investigated the presence of risk factors associated with tracheostomy complications, mechanical ventilation duration, length of stay in ICU and intra-hospital mortality.

**Results:** Our study identified 295 patients who underwent a tracheostomy for the following indications—ventilatory comfort in chronic neuromuscular disorders (n = 141), mechanical ventilation withdrawal in intensive care polyneuropathy (n = 140) and upper airway obstruction (n = 14). The median age of the population was 62 years (Q1 49 & Q3 72), the median BMI was 24.8 kg m^2^ (Q1 21.7 & Q3 27.8) and the median IGS2 score was 51 (38 & Q3 65). The median cannula size of tracheostomy was 8 mm. The procedures were percutaneously performed in 73.6% of the cases with systematic tracheal fibroscopy, and more than a third of the cases (38.7%) were performed by medical resident. The complications rate was 21.7%, including early complications in the first week in 52.8% of the cases. 32.6% of the complications were serious. No risk factors for complications were identified. 141 patients were decannulated with a median time of 31 days (Q1 17 & Q3 63). 8.1% of patients had otolaryngologist follow-up. The overall mortality of the studied population was 30.1% including mortality related to tracheostomy in 2 patients. The tracheostomy for extended mechanical ventilation was significantly associated with an increase of mechanical ventilation duration before tracheostomy (p < 0.0001), duration of mechanical ventilation (p < 0.0001), length of stay in intensive care unit (p < 0.0001) and mortality rate (p = 0.0003).

**Table:** Characteristics of tracheostomized patients and complications presented according to indication of tracheostomy

Indication of tracheostomy	Neuromuscular pathology	Extended mechanical ventilation	Obstruction airways
Number of patients	141	140	14
Median age (years)	57	66	56.5
Median BMI (kg/m^2^)	24.7	24.85	22.6
Median IGS2 score	48	53	35
Timing of tracheotomy			
Early (≤ 7 days)	13.9% (n = 24)	2.9% (n = 4)	92.9% (n = 13)
Late (> 7 days)	86.1% (n = 117)	97.1% (n = 136)	7.1% (n = 1)
Tracheotomy procedure			
Percutaneous	87.2% (n = 123)	66.4% (n = 93)	7% (n = 1)
Surgical	12.3% (n = 18)	33.6% (n = 47)	93% (n = 13)
Operator experience			
Interne/Assistant	39.0% (n = 55)/34.0% (n = 48)	22.9% (n = 32)/35.7% (n = 50)	0% (n = 0)/14.2% (n = 2)
Hospital practitioner	20.6% (n = 29)	36.4% (n = 51)	85.8% (n = 12)
Tracheal tube size (mm)	8	8	8
Median mechanical ventilation duration before tracheotomy (days)	14	22	1
Median mechanical ventilation duration (days)	20	37	2.5
Number of complications	21.3% (n = 30)	22.1% (n = 31)	21.4% (n = 3)
Median time before decanulation (days)	30	32.5	32
Median length of stay in ICU (days)	25	39.5	5
Mortality in ICU	6.4% (n = 9)	24.3% (n = 34)	14.3% (n = 2)
Mortality in hospital	18.4% (n = 26)	38.6% (n = 54)	14.3% (n = 2)

**Conclusion:** Tracheostomy procedure, whatever the indications, remains a high risk procedure in ICU patients. Rigorous care, daily clinical monitoring and regular otolaryngologist follow-up may reduce the incidence and consequences of complications of tracheostomy.

### P-131 Echocardiography-Doppler evaluation during weaning process

#### Trifi Ahlem^1^, Ben Ismail Khaoula^1^, Abdellatif Sami^1^, Daly Foued^1^, Touil Yosr^1^, Trabelsi Insaf^1^, Nasri Rochdi^1^, Ben Lakhal Salah^1^

##### ^1^Tunis, Tunisie

###### **Correspondence:** Trifi Ahlem - trifiahlem2@gmail.com

*Annals of Intensive Care* 2018, **8(Suppl 1):**P-131

**Introduction:** The weaning of invasive ventilation is a determining factor in critical patient’s outcome. Screening of patients at risk of failure is a fundamental step in the weaning process. We aimed to study the hemodynamic changes during the weaning process and to identify the predictive signs of cardiovascular failure by using the transthoracic echocardiography doppler (TTE-doppler).

**Patients and methods:** a six-month descriptive and analytical prospective study that included patients ventilated for at least 48 h and considered ready for weaning. TTE-doppler was performed after 30 min of the pressure support ventilation mode with Positive End Expiratory Pressure (PSV-PEEP) and the spontaneous breathing trial with the T-tube test. The left ventricular ejection fraction (LVEF), cardiac output (CO), maximum velocity of the mitral waves E and A and the tissue mitral annular wave E’, deceleration time of mitral E wave (DTE), difference between the pulmonary A and mitral A times [(Ap-Am) time], the Flow propagation velocity of mitral flow (Vp) and its derived ratio E Vp were measured and compared between weaning success (n = 31) versus failure (n = 22) and between PSV-PEEP (n = 53) versus T-Tube test (n = 37).

**Results:** 53 patients were studied and 22 weaning failures were retained (16 in the PSV-PEEP mode and 6 in the T-tube test). Comparison of baseline TTE-Doppler parameters (PSV-PEEP) revealed that the failure group had—a more altered LVEF (49 vs. 62%, p = 0.03), a shortened DTE (154 vs. 199 ms, p = 0.01), a higher E E’ (9.44 vs. 7.07, p = 0.04), a higher d (Ap-Am) (27 vs. 2.25 ms, p < 10–3) and a higher E Vp 2.8 vs. 1.2, p = 0.009). The right ventricular functions and IVC aspect were similar. The changes during the T-tube test appear on the attached table. E A > 2, DTE < 150 ms, E E’ > 8, d (Am-Ap) > 10 ms, E Vp > 2.5 were significantly associated with the weaning pulmonary oedema in univariate analysis but not in logistic regression. Their simultaneous presence was significantly related to the weaning pulmonary oedema (OR 18.52, 95% CI [4.08–52], p = 0.01).

**Table:** TTE-Doppler assessment between PSV-PEEP and T-Tube test

	PSV-PEEP (n = 53)	T-Tube test (n = 37)	p
HR (bpm)			
Success weaning Failure weaning	101 ± 19 126 ± 21	108 ± 26 134 + 23	0.05 0.02
SAP (mmHg) (mm Hg)			
Success Failure	135 ± 21 152 ± 18	146 ± 10 166 ± 21	0.014 0.029
LVEF (%)			
Success Failure	62 ± 7 49 ± 10	64 ± 5 48 + 13	0.12 0.33
SV (ml)			
Success Failure	74 ± 24 69 ± 28	75 ± 17 68 + 21	0.78 0.8
CO (l/mn)			
Success Failure	6.5 ± 2.9 6.8 ± 2.5	6.9 ± 1.8 7.1 ± 2	0.009 0.01
E/A			
Success Failure	1.40 ± 0.57 1.61 ± 0.72	1.42 ± 0.46 1.64 ± 0.7	0.14 0.08
DTE (ms) Success Failure	199 ± 73 154 ± 57	198 ± 66 133 ± 53	0.5 <10^−3^
E/E′			
Success Failure	7.07 ± 3.5 9.44 ± 4.8	6.8 ± 4 12.8 ± 4.5	0.65 0.007
(Ap-Am) time (ms), mean [min–max]			
Success Failure	2.25 [− 20 to 17] 27 [15 to 42]	6.7 [− 14 to 9] 48 [19 to 66]	0.22 0.001
Vp MF (cm/s)			
Success Failure	55 ± 16 46 ± 13	54 ± 9 43 ± 15	0.9 0.18
E/Vp MF			
Success Failure	1.2 ± 0.5 2.8 ± 0.6	1.4 ± 0.8 3.2 ± 0.5	0.17 0.025
CI-IVC (%)			
Success Failure	17 ± 13 14 ± 11	16 ± 8 13 ± 6	0.82 0.16
PSP			
Success Failure	35 ± 10 43 ± 7	34 ± 2 45 ± 5	0.33 0.12

PSV-PEEP: pressure support ventilation mode with Positive End Expiratory Pressure; HR: heart rate; SAP: systolic arterial pressure; LVEF: left ventricular ejection fraction; SV: stroke volume; CO: cardiac output; E/A: ratio of maximal mitral E wave and A wave velocities; DTE: deceleration time of mitral E wave; E/E′: ratio of the maximum velocity of the mitral wave E and the tissue mitral annular wave E′; (Ap-Am) time: difference between the pulmonary A and mitral A times; Vp MF: the Flow propagation velocity of mitral flow (Vp); CI-IVC: collapsibility index of inferior vena cava; PSP: Pulmonary systolic pressure

**Conclusion:** TTE-doppler has a valuable contribution into the weaning process + it detects the functional and hemodynamic changes induced by spontaneous breathing tests and helps to identify patients at high risk of weaning failure from cardiac origin.

### P-132 Calcium measurements in ICU patients. Are current practices clinically relevant?

#### Dreyfus Aliénor^1^, Trouiller Pierre^1^, Jacobs Frédéric^1^

##### ^1^CHU Antoine Béclère, Clamart, France

###### **Correspondence:** Dreyfus Aliénor - alienordreyfus@gmail.com

*Annals of Intensive Care* 2018, **8(Suppl 1):**P-132

**Introduction:** Ionized calcium (iCa) and not total calcium (totCa) is the physologically relevant component of blood calcium. Hypoalbuminemia, acidemia, acute elevation of free fatty acids concentrations may result in poor correlation of total calcium with direct measurements of iCa. Thus the type of calcium measurement (iCa, totCa with or without adjustment for albumin or total protein) may lead to misinterpretation of calcium disorders. We report the results of a practice study in critically ill patients.

**Patients and methods:** Online survey about calcium measurement in ICU patients consisting of eleven questions regarding frequency and indication of measurement, type of measurement (totCa or iCa), adjustment of totCa results.

**Results:** 59 answers, mainly anesthesia critical care or critical care residents (residents 72.9%, senior physicians 27.1%).76.3% of the responders systematically measured calcium at the time of ICU admission. Subsequent systematic measuremnts during ICU stay were performed in 71.2% of cases. In first intention, iCa was tested in only 36.2% of cases and totCa in 63.8%.69.2% of totCa values were adjusted, mainly on albumin (albumin 59.6%, total protein 28.8%).

**Conclusion:** Among the responders of this sample, calcemia measurement is frequent. Practices are heterogeneous. Total calcemia remains the most frequent method. One third of totCa results are not adjusted. Furthermore, robust data concerning the reliability of totCa adjustment are lacking in this specific population. Complementary studies are probably needed to determine this latter aspect. Despite its widespread use, usfulness of routine calcium measurement is not warranted and may lead to misinterpretation of the concentration of calcium and hazardous diagnosis of dyscalcemia.

### P-133 Central diabete insipide in our departement of neuroreanimation

#### Triki Hadjer^1^, Naili Amine^1^, Bouali Djouheur^1^

##### ^1^EHS Neuro, Tipasa, Algeria

###### **Correspondence:** Triki Hadjer - batchhadjer26@gmail.com

*Annals of Intensive Care* 2018, **8(Suppl 1):**P-133

**Introduction:** Central diabetes insipidus (CDI) is defined by the inability to retain free water, it is due to an insufficient release of the anti-diuretic hormone by the hypothalamus. CDI is one of the complications to look for after neurosurgery, or cranial trauma, it can be transient in 30% of cases, and definitive in 2–10% of cases.

**Patients and methods:** It is about a retrospective study of 530 patients hospitalized in our department from June 1st, 2015 to January 31, 2016, of which 25 patients had central diabetes insipidus, which means a frequency of 5%. Clinical, para-clinical, etiological and therapeutic data were collected from the hospitalization records.

**Results:** In a series of 530 patients hospitalized during the defined period, the number of patients with central diabetes insipidus was 25, which means an incidence rate of 5%, the average age was 46 years. Trans-sphenoidal pituitary surgery was the leading cause of central diabetes insipidus with a rate of 64%. With hormone therapy and good rehydration, diabetes was transient in 24 patients, a rate of 96%, and definitive in 1 patient or a rate of 4%.

**Conclusion:** The incidence of central diabetes insipidus in neuro-intensive care unit is certainly not negligible, it is for this reason that it must be diagnosed early in order to allow a better multidisciplinary assistance.

### P-134 Prognostic factors of acute renal failure in post-operative sepsis

#### Mayola Vanadiaku Ulrich^1^, Khaleq Khalid^2^, Soufi Aziz^2^, Bouhouri Aziz^2^, Nsiri Afak^2^, Hamoudi Driss^2^, Al Harrar Rachid^2^

##### ^1^Hôpital Beausejour, Casablanca, Morocco; ^2^University Hassan II, Casablanca, Morocco

###### **Correspondence:** Mayola Vanadiaku Ulrich - ulrichvan2002@yahoo.fr

*Annals of Intensive Care* 2018, **8(Suppl 1):**P-134

**Introduction:** Acute renal failure complicating surgery has a particularly harmful prognosis, with a mortality of 40% to 90%. This high mortality rate is attributed to patient-related factors, the severity of the disease and the type of surgery, but not to the acute renal failure itself. The aim of our study is to elucidate the prognostic factors of acute renal failure in the postoperative sepsis in a series of 100 patients.

**Patients and methods:** It is a retrospective analytical descriptive study spread over a period of 4 years (from January 2013 to December 2016), 100 observations of postoperative peritonitis were collected in the service of resuscitation of surgical emergencies of CHU Ibn Rochdof Casablanca. The statistical analysis was carried out using the SPSS 20 software. The results are expressed with OR and 95% confidence intervals (CI at 95%). The results were considered significant when P is < 0.05.

**Results:** The mean age of the patients was 46 ± 14 years with a sex ratio of 1.94 (66 M 34). Renal failure was the most frequent failure after hemodynamic failure, 11 patients were oliguric, 4 anuriques and 37 patients had a preserved diuresis, patients were divided according to the RIFLE (R 49%, I 26%, F 25%) and AKIN (I 49%, II 26%, III 25%). The predictive factors of acute renal failure ARI were studied in univariate and multivariate analysis, 2 factors were retained including catecholamines—OR 3.362 + CI at 95% between 1.202 and 3.397 + p = 0.021 + the surgical site—OR 0.367 + CI at 95% between 0.145 and 0.929 + p = 0.034.

**Conclusion:** Acute renal failure is an independent factor of mortality in the post-operative sepsis, but remains that its presence is a pejorative prognostic factor.

### P-135 Metabolic and hemodynamic effects of molar sodium lactate in critically ill patients

#### Piton Gaël^1^, Granperrin Mathilde^1^, Mahr Nicolas^1^, Winiszewski Hadrien^1^, Capellier Gilles^1^

##### ^1^CHU Besançon, France

###### **Correspondence:** Piton Gaël - gpiton@chu-besancon.fr

*Annals of Intensive Care* 2018, **8(Suppl 1):**P-135

**Introduction:** Molar sodium lactate is an hypertonic crystalloid fluid. Recent prospective studies including few patients have suggested the interest of hypertonic sodium lactate for vascular filling among critically ill patients and for the treatment of intracranial hypertension. The metabolic effects of sodium lactate in the context of the ICU have been poorly documented. We aimed to describe hemodynamic and metabolic effects of an infusion of molar sodium lactate administered in critically ill patients.

**Patients and methods:** This was a retrospective study performed in a large University Hospital. All patients receiving the molecule were included in the analysis. Indication for sodium lactate, dose, and modality of administration were collected. We also collected clinical and biological variables before sodium lactate infusion, after 6 h (H6), and after 24 h (H24). An analysis of the evolution of these variables at H6 and H24 was performed.

**Results:** Between January 2016 and May 2017, 104 patients, aged 64 years, 66% males, SOFA score 9 [7–12], received an infusion of molar sodium lactate (250 ml [250–250]). Main indications for sodium lactate were hyperchloremic metabolic acidosis (68%), vascular filling (15%), mixed acidosis (12%), and intracranial hypertension (5%). 84% of the patients presented with a chloride sodium ratio > = 0.76 at basal time. Sodium lactate was associated with a significant increase of mean arterial pressure at H6 (p = 0.009) and H24 (p = 0.003), a decrease of catecholamine dose (p = 0.002) and heart rate (p = 0.05) at H24, and an increase of diuresis in the 6 h period following initiation of the treatment (p = 0.02). We observed an increase of pH, bicarbonate, base excess, and sodium, at H6 and H24 (all p < 0.0001). Plasma lactate concentration was increased at H6 (p < 0.0001), but was not different from basal value at H24 (p = 0.99). There were no significant variation of plasma chloride. Chloride sodium ratio was significantly reduced. Plasma sodium > = 150 mmol l and pH > = 7.50 at H24 were observed in 5% of the patients.

**Conclusion:** This retrospective study reports the largest number of critically ill patients having received sodium lactate. Hemodynamic effects observed in this study are concordant with the data of the literature. The metabolic effects observed in this study, with rapid increase of pH, bicarbonate, and base excess, strongly suggest the potential interest of sodium lactate among critically ill patients presenting with acidosis and increased chloride sodium ratio.

### P-136 Euglycemic ketoacidosis, a common and underecognized complication of continuous renal replacement therapy

#### Moulin Thibaut^1^, Coutrot Maxime^1^, Hékimian Guillaume^1^, Bréchot Nicolas^1^, Schmidt Matthieu^1^, Besset Sébastien^1^, Nieszkowska Ania^1^, Franchineau Guillaume^1^, Bourcier Simon^1^, Bourron Olivier^1^, Luyt Charles-Edouard^1^, Combes Alain^1^

##### ^1^Hôpital Pitié Salpêtrière, Paris, France

###### **Correspondence:** Moulin Thibaut - tibo.moulin@yahoo.fr

*Annals of Intensive Care* 2018, **8(Suppl 1):**P-136

**Introduction:** Acute kidney injury (AKI) is a frequent and severe condition in intensive care unit patients that may require renal replacement therapy, most frequently continuous renal replacement therapy (CRRT). Although hypoglycemia is a well-known complication of CRRT using glucose free solutions, euglycemic ketoacidosis (EKA) has never been described in this setting.

**Patients and methods:** All anuric patients with glucose free CRRT solution induced EKA (February 2017–May 2017) were prospectively included and evaluated. Ketoacidosis was deemed possible when non-lactic metabolic acidosis did not improve in patients on CRRT. Because all patients were anuric, we measured ketonemia and used urinary test strip in the effluent fluid. EKA diagnosis was retained when arterial serum bicarbonate was < 20 mEg/l despite CRRT, in the absence of lactic acidosis and in the presence of ketones in the serum or CRRT effluent fluid.

**Results:** Eighteen patients (15% of our patients under CRRT in this period) developed EKA during CRRT using glucose free solution (Phoxilium^®^). Time between CVVHDF initiation and ketonemia detection was 2 (1–3) days. Patient characteristics are presented in the Table 1. Half of them had for a medical history of diabetes (5 insulin-dependent). Only 3 patients were receiving insulin and most of them had low glucose or food intake. Increasing glucose intake and insulin infusion resolved ketonemia in all cases.

**Table 1:** Characteristics of the 18 patients who developed EKA under CRRT using glucose free solution

Characteristics	n (%) or median (IQR)
Age	64 (58–72)
Male	12 (67%)
BMI (kg/m^2^)	25.1 (23.4–33.9)
Diabetes	9 (50%)
Insulin-dependant diabetes	5 (28%)
Severe chronic kidney disease	0
SAPS II	82 (51–86)
SOFA admission	12 (10–14)
Reason for ICU admission	
Cardiac surgery	8 (44%)
Heart transplantation	2 (11%)
Septic shock	2 (11%)
Cardiogenic shock	10 (55%)
Cardiac arrest	1 (5%)
ARDS	1 (5%)
Mean arterial pressure at admission	75 (67–88)
Mechanical ventilation at admission	12 (67%)
Veno-venous ECMO	1 (5%)
Veno-arterial ECMO	9 (50%)
Time from ICU admission to ketonemia detection, days	3 (2–7)
Time from CRRT initiation to ketonemia detection, days	2 (1–3)
Dialysate and reinfusion flow rate (ml/kg/h)	29 (26–33)
Creatinine level the day of ketonemia detection, µmol/L	135 (98–159)
Anuria during hemodiafiltration	18 (100%)
ICU mortality	11 (61%)

	At ketoacidosis detection	After glucose and insulin infusion
pH	7.36 (7.32–7.39)	7.44 (7.32–7.49)
Bicarbonates level (mmol/l)	17.3 (14–18.9)	21 (19.1–21.9)
pCO_2_ (mmHg)	31.9 (26.9–35)	30 (28.1–38)
Lactate (mmol/l)	1.7 (1.3–2)	1.6 (1.3–2.4)
Insulin infusion (IU/h)	0 (0–0)	3 (2–5)
Glucose infusion (g/h)	1.05 (1.05–2.10)	2 (1–12)
Enteral nutrition (kcal/h)	0 (0–31)	21 (0–62)
Glycemia (mmol/l)	6.2 (5–7)	6.5 (5–8)
Ketonemia (mmol/l)	2 (1.7–3.1)	0(0–0.025)
Ketones in the effluent liquid (g/l)	0.15 (0.025–0.8)	0 (0–0)

**Discussion:** We describe for the first time the occurrence of euglycemic ketoacidosis in critically ill patients under CRRT using glucose-free replacement solution. Common features of the patients were multiple organ failure with anuria, normal glycemia without insulin infusion and low glucose infusion or food intake. Critical illness-induced insulin resistance and starvation could altogether contribute to ketoacidosis even if acidosis is unusual in starvation ketosis. By removing substantial amounts of glucose from the blood, CRRT with glucose free solution could worsen this condition, mask hyperglycemia and induce euglycemic ketoacidosis.

**Conclusion:** In critically ill patients on CRRT using glucose free solution, euglycemic ketoacidosis is common and should be detected, especially in patients with low glucose intake, no insulin infusion and unexplained metabolic acidosis. Importantly, the diagnosis can be missed in anuric patients with normal blood glucose and in the absence of known diabetes. Since, CVVHDF-induced ketoacidosis may contribute to persistent acidemia and its adverse effects, serum or CRRT effluent fluid ketone level should be measured in this setting.

### P-137 Epidemiology, clinical features and management of severe hypercalcemia in critically ill patients

#### Mousseaux Cyril^1^, Ghrenassia Etienne^2^, Kerhuel Lionel^2^, Ardisson Fanny^2^, Ekpe Kenneth^2^, Lemiale Virginie^2^, Mariotte Eric^2^, Schlemmer Benoit^2^, Azoulay Elie^2^, Zafrani Lara^2^

##### ^1^Paris, France; ^2^Hôpital Saint Louis, Paris, France

###### **Correspondence:** Mousseaux Cyril - mousseaux.cyril@gmail.com

*Annals of Intensive Care* 2018, **8(Suppl 1):**P-137

**Introduction:** Hypercalcemia (HCM) affects about 3% of patients admitted in the ICU, and is associated with mortality. However, detailed clinical features, the causes, mechanisms and biochemical features related to hypercalcemia have never been described.

**Patients and methods:** This retrospective study was performed in a 12-beds medical ICU between January 2007 and January 2017. All patients hospitalized with a severe HCM (defined by a calcemia above 3 mmol liter) at admission were included. Univariate and univariable prognosis analyses were made with Fisher exact test (Software R v3.2.2).

**Results:** We included 78 patients (female n = 40 (51.2%)) aged of 58 [39.5–67] years. Main reasons for admission were hypercalcemia (n = 55 (70.5%)), followed by acute encephalopathy (n = 9 (11.5%)). Median SAPS II and SOFA scores were 32 [22.25–39.5] and 3 [1–5] respectively. Main causes of HCM were hematological malignancies (n = 46 (59%)), solid tumors (n = 15 (19%)), iatrogenic events (n = 9 (9%)) and endocrinopathies (n = 5 (6%)). Median calcium levels at admission, at day 3 and at ICU discharge were 3.68 [3.43–4.21], 2.75 [2.55–3.12] and 2.56 [2.15–3.14] mmol L respectively. More than half of the patients (n = 46 (59%)) recovered from HCM 5 days after ICU admission. Acute kidney injury occurred in 65 (83%) patients and 19 (29.2%) patients required dialysis. Neurological complications concerned 27 (34.6%) patients, mainly delirium (n = 23, 29.4%). Digestive events occurred in 29 (37.1%) patients. Cardiovascular events concerned 49 (63%) patients and consisted in de novo hypertension in 18 (23%) patients, and EKG disturbances in 24 (30%) patients. During ICU stay, 9 (11.5%) patients required mechanical ventilation and 6 (7.6%) patients required vasopressors. Volume resuscitation with crystalloids was the first treatment in 73 (93.5%) patients, 63 (80.7%) received bisphosphonates and 48 (61.5%) received corticosteroids. Respective ICU and hospital mortality were 11.5 and 21.7%. There was no correlation between the degree of HCM and ICU mortality (p = 0.1). ICU and hospital mortality were associated with the underlying disease (hematological malignancies (p = 0.04)).

**Conclusion:** HCM is associated with high mortality rates. The increased mortality is a consequence of the main mechanism, mainly underlying malignancy rather than HCM per se. The course of HCM may be complicated by organ failures that are most of the time reversible with early ICU management.

### P-138 The Farnseoid X receptor regulation is implicated in the prognostic of septic uremic mice

#### Caillard Pauline^1^, Mbaya Eleonore^1^, Kamel Said^1^, Massy Ziad^1^, Maizel Julien^1^

##### ^1^CHU Amiens-Picardie, Amiens, France

###### **Correspondence:** Caillard Pauline - paulinecaillard@orange.fr

*Annals of Intensive Care* 2018, **8(Suppl 1):**P-138

**Introduction:** Sepsis is one of the leading cause of death among patients with chronic kidney disease (CKD). The mechanisms of this higher mortality remain poorly understood. Sepsis and chronic kidney disease are both conditions associated with a higher plasmatic concentration of bile acids. The Farnesoid X receptor (FXR) is a key regulator of the bile acid metabolism and has recently been involved in the regulation of the inflammasome during sepsis. We explored the role of FXR in the prognostic of sepsis in an animal model of CKD.

**Patients and methods:** Sepsis was provoked by the injection of 0.5 mg kg of LPS 6 weeks after the creation of CKD. The CKD was created by unilateral nephrectomy associated with contralateral thermocauterisation. The mice (C57BL6 J) were randomly assigned to one of the following groups—sham placebo, CKD placebo, sham LPS or CKD LPS. A fifth group of CKD LPS mice received a treatment with sevelamer (a bile acid sequestrant) during 6 weeks. Survival of the animals, serum biochemistry and molecular biology in the kidney were performed after sacrifice.

**Results:** Whereas the sham LPS animals survived, all CKD LPS animals died during the 72 h following the injection of LPS. The plasmatic urea, IL1beta and TNFa concentrations increased with the creation of CKD (CKD placebo versus sham placebo animals) and with the creation of sepsis (CKD LPS versus sham LPS groups). Whereas the expression of FXR RNA did not changed with the injection of LPS in the sham animals (sham LPS versus sham placebo), the FXR RNA decreased with the creation of sepsis in the CKD animals (CKD LPS versus CKD placebo groups). The CKD animals treated with sevelamer 6 weeks before the administration of LPS (CKD sev LPS group) had a lower plasmatic concentration of Il1b, TNFa and increased the RNA expression of FXR in the kidney compared to the CKD LPS group. Also, the treatment with sevelamer improved the survival of the CKD LPS animals.
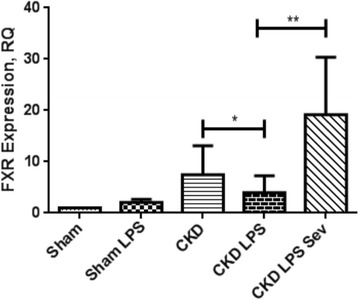


**Conclusion:** our study demonstrates a relation between FXR and the prognostic of sepsis in CKD animals. The exact link and the potential therapeutic interest of targeting FXR and bile acids metabolism in CKD patients remain to be studied.

### P-139 Estimation of 24-Hour electrolyte Excretion Using Urine Samples in ICU

#### Sboui Ghada^1^, Beji Olfa^1^, Atig Rabia^1^, Baroudi Jamila^1^, Ben Rejeb Mohamed^1^, Bouslama Ali^1^, Hmouda Houssem^1^

##### ^1^Hôpital Sahloul, Sousse, Tunisie

###### **Correspondence:** Sboui Ghada - ghadasb@hotmail.com

*Annals of Intensive Care* 2018, **8(Suppl 1):**P-139

**Introduction:** Dysnatraemia, dyskalaemia and hypomagnesemia are frequent metabolic disorders in intensive care, and their causes represent a major concern for the intensivist, especially in urgent conditions. In the diagnostic approach, we often use the urine analysis. Although measurement of 24-hour urine electrolyte excretion (24-HU) is considered the most reliable method, the great burden and difficulty in collecting complete 24-hour urine has prompted the search for more practical methods, such as spot urine analysis. The aim of the present study was to compare electrolyte excretion in urine samples collected over different time periods, in comparison with a 24-hour urine sample collection considered as the gold standard method.

**Patients and methods:** This prospective and descriptive study included 30 patients admitted in a tunisian medical ICU, between September and December 2015. Baseline characteristics, medications and laboratory data including electrolytes and renal function parameters were obtained from all patients. Multiple urine specimens for analyzing Na + K + Mg + Urea + Ca + phosphate + Creatinine + proteins and uric acid were obtained from 2-hour, 12-hour and 24-hour urine samples during day and night time, and results were compared with those obtained from the gold standard method (24-hour urine collection). Correlation analysis was performed using the spearman test.

**Results:** Significant correlation was found for all biochemistry parameters between 24-hour urine results and those obtained from 2-hour and 12-hour samples regardless of day or night sampling. A comparative analysis for sodium and potassium is shown in Fig. 1.

**Fig. 1:**
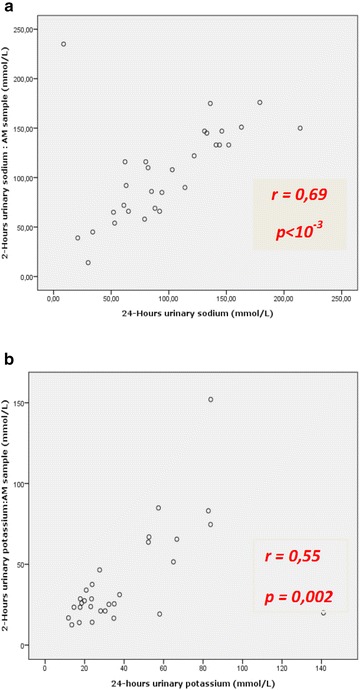
Correlation between 24-hour urine excretion and 2-hour urine excretion for sodium (**a**) and potassium (**b**)

**Conclusion:** Determination of electrolyte excretion from urine samples taken over 2 different time periods, 2 and12 h, provides a reliable estimation of 24-hour urine electrolyte excretion. It appears practical for early understanding of the mechanism of electrolyte imbalance. However, further studies are warranted to confirm the usefulness of this approach.

### P-140 Use of the procalcitonin assay in an adult emergencies department: retrospective experience of a general hospital of the suburb of Paris

#### Zamparini Emmanuelle^1^, Benmerjaa Hichem^1^, Soletchnik Mickael^1^, Khezam Noredine^1^, Safrano Geoffroy^1^, Vincent François^1^

##### ^1^CHI Le Raincy Montfermeil, Montfermeil, France

###### **Correspondence:** Zamparini Emmanuelle - manou.zamparini@yahoo.fr

*Annals of Intensive Care* 2018, **8(Suppl 1):**P-140

**Introduction:** The procalcitonin dosage (PCT) is expensive and its place in the care of adult patients consulting in the emergencies department (ED) is poorly-defined.

**Patients and methods:** All consecutive adult patients consulting in the ED in whom a PCT assay was performed between 1st January 2016 and 13th August 2017 were included. Results are reported as medians and interquartile range or numbers. In case of dosage of PCT < 0.10 ng mL or C-Reactive Protein (CRP) < 3 mg dL it were considered as 0.

**Results:** 322 patients were included (55265 consultations in the adult ED during this period + PCT assay—0.6%). 55.6% males aged of 60.9 years (40.0–77.4). Most of patients were coming from home (90.1%). Reason for consultation were considered as suspicion of infectious disease in only 149 (45.9%). The body temperature measured at ED admission was of 36.9 °C (36.6–37.5). PCT assay was associated with a CRP one in 94.9%. A blood count was performed in 88.8%. White blood cells were 10.20 G L (7.85–13.4). Other markers of infectious were poorly recorded (fibrinemia in34 (11.5% + 5.5 g L [4.6–6.6] + Immature forms on blood count—11.5%). Only 172 (53.6%) had blood cultures in the ED (1 patient [1–2]) and 163 (50.3%) other(s) microbiological sample(s), mainly urinary (84 patients [25.9% + among them 33% considered as positive]). 5% of blood cultures were positives, mainly for gram negatives (90%). Final diagnosis in the ED was considered as infectious disease (ID) in only 142 patients (44.1%, including 11 sepsis and 5 septic shocks). 175 (44.6%) was considered as non-infected (NID) and final diagnosis remains unprecise in 8 (1.6%). PCT values was of 0.42 (0–1.38) in the ID vs. 0 (0–1.37) in the NID (p < 0.001), WBC was of 10.68 in the ID vs. 9.75 in the NID (p < 0.013) and CRP was of 88 (22.5–185.5) in the ID vs. 21 (5–76) in the NID (p < 0.001). No correlation was observed between the PCT value and admission to dechocage room admission.

**Table:** Values of biomarkers in the diagnosis of infection in the ED (compared at those without non- final diagnosis of infection)

	OR	95% CI	*p*
CRP	1.004	1.001–1.008	0.011
PCT	1.608	1.127–2.292	0.009
WBC	1.023	0.973–10.75	0.377

**Conclusion:** PCT assay seems often wrongly prescribed in adult patients attending ED. It also seems of little diagnostic interest.

### P-141 Nonurgent visits to Emergency Department: estimation with a predictive model

#### Persico Nicolas^1^, Noel Guilhem^2^, Viudes Gilles^2^, Roch Antoine^1^

##### ^1^Hôpital Nord, Marseille, France; ^2^GIP e-Santé ORU PACA, Hyères, France

###### **Correspondence:** Persico Nicolas - nicolas.persico@ap-hm.fr

*Annals of Intensive Care* 2018, **8(Suppl 1):**P-141

**Introduction:** Nonurgent visits to Emergency Departments (ED) could contribute to ED overcrowding, whose deleterious consequences for patients are now well known. The literature shows considerable variability concerning methods and definitions for categorizing ED visits nonurgent. In French ED, characteristics of each visit are daily collected in a file called Summary of ED Visit to monitor activity. The main objective of this study was to develop and validate a predictive model of a nonurgent visit from the available data, at the national level, in the Summaries of ED Visits.

**Patients and methods:** We conducted a descriptive prospective and monocentric study in May 2017. Patients aged 16 years or older admitted to the ED were included except for patients who left without beeing seen or escaped. First ED visits were categorized nonurgent or urgent by the emergency physicians at the end of the stay from a consensual definition. A panel of 23 experts defined a nonurgent ED visit as a consultation which could have been deferred for 24 h to a general practitioner. Then a predicitive model was developed from variables of the Summary of ED Visit by multivariate logistic regression during a first period (15 days). During the validation’s period (15 days), categorization of nonurgent ED visits by the model was compared to categorization of emergency physicians.

**Results:** 3865 patients were included. Mean age (standard deviation) was 45.2 (21.0) years. Number of ED visits analyzed was 4090 (220 patients consulted 2 times or more). Emergency physicians categorized 44.6% Confidence Interval 95% (CI 95%) [43.1% + 46.1%] of ED visits as non urgent. Table 1 shows variables included in the model during the development’s period (n = 2039 visits). On the validation’s period (n = 2051 visits), 49.4% of the visits were categorized non urgent by the model versus 45.8% by the emergency physicians, beeing a surrestimation of 7.9%. Model’s performances were—sensitivity = 71.4% CI 95% [68.4% + 74.2%], specificity = 69.1% CI 95% [66.3% + 71.8%], positive predictive value = 66.2% CI 95% [63.2% + 69.0%], negative predictive value = 74.1% CI 95% [71.3% + 76.7%] and Area Under Curve = 0.64 CI 95% [0.61–0.66] with p < 0.001.

**Table 1:** Variables included in the predictive model of nonurgent visits (period of development, n = 2039)

	Univariate analysis			Multivariate analysis		
	OR	CI 95%	*p*	OR adjusted	CI 95%	*p*
Age (years)	0.98	(0.98–0.99)	<0.001	0.99	(0.99–1.00)	0.004
Sex						
Male*						
Female	1.40	(1.17–1.67)	<0.001	1.33	(1.08–1.63)	0.007
ED visit(s)						
1*						
≥ 2	1.37	(0.89–2.12)	<0.001	1.78	(1.08–2.95)	0.025
Provenance						
Public road/work*						
Home	2.09	(1.57–2.78)	<0.001	1.65	(1.17–2.32)	0.004
Transport						
Paramedics*						
Self referral	2.67	(2.22–3.21)	<0.001	1.58	(1.27–1.98)	<0.001
CIMU						
1/2*						
3	5.30	(2.73–10.31)		3.69	(1.74–7.81)	
4	10.11	(5.20–19.65)		5.57	(2.62–11.84)	
5	16.80	(8.47–33.32)	<0.001	8.07	(3.71–17.55)	0.001
Main diagnosis						
Trauma*						
Medical	0.83	(0.68–1.01)	0.064	1.67	(1.31–2.13)	<0.001
CCMU						
1/2	7.95	(6.12–10.34)	<0.001	3.89	(2.86–5.27)	<0.001
3/4/5*						
Orientation						
Hospitalization*						
Discharge	8.10	(6.11–10.74)	<0.001	3.47	(2.50–4.82)	<0.001

OR = odd ratio; CI 95% = confidence interval 95%; ED = Emergency Department CIMU = «Classification Infirmière des Malades aux Urgences», French nurse’s classification for severity of patients at the admission. Level 1 is the more serious and level 5 is the less serious; CCMU = «Classification Clinique des Malades aux Urgences», French emergency physician’s classification for severity of patients at the end of the stay. Level 5 is the more serious and level 1 is the less serious

*Modality of reference

**Conclusion:** This model could be used to estimate the proportion of nonurgent visits in French ED in order to implement measures to better manage nonurgent patients.

### P-142 Patients referred to the emergency department in Paris following the use of recreational drugs: clinical presentation and outcome

#### Hemmaz Kathia^1^, Galichon Bertrand^1^, Plaisance Patrick^1^, Labat Laurence^1^, Megarbane Bruno^1^

##### ^1^Hôpital La Riboisière, Paris, France

###### **Correspondence:** Hemmaz Kathia - kathia.hemmaz@aphp.fr

*Annals of Intensive Care* 2018, **8(Suppl 1):**P-142

**Introduction:** New psychoactive substances (NPS) have emerged on the recreational scene for 10 years. Data regarding the exact NPS toxicity are still limited. In 2013, the European Drug Emergencies Network (Euro-DEN) reported that only 5.6% of patients referred to the emergency department (ED) in relation to drug use did use at least one NPS. Our objectives were 1—to identify drugs responsible for ED presentation and 2—to describe features, outcome and management on ED presentation with drug toxicity.

**Patients and methods:** We prospectively collected data on all recreational drug toxicity presentations to the ED in a University Hospital in Paris (belonging to Euro-DEN) during 30 months (October 2014–June 2017). Recreational drug was defined as “a psychoactive compound that was taken for the purpose of recreational activities rather than for medical or work purposes or for self-harm”. Isolated ethanol intoxication, drug withdrawal and secondary complications of chronic drug use were excluded. Information on the drugs involved in the presentations was on the basis of patient self-reporting.

**Results:** 703 patients (532 males 171 females, age 32 years [26 + 41] + median [percentiles 25 + 75]) were included. About 60% were transported by ambulances or fire workers. The most involved drugs were cocaine (27%), cannabis (25%), MDMA (17%) and crack (10%). NPS were rarely declared (3%). Concomitant ethanol drinking was reported (48%). Although serious clinical features were not seen in most presentations and 51% were medically discharged from the ER (length of stay 5.3 h), patients presented agitation (24%), acute anxiety (21%), acute psychosis (16%), palpitations (10%), seizures (8%) and or hypertension (8%). Glasgow coma score was 15 [14 + 15] on admission and 15 [13 + 15] at the worst moment. When performed, serum lactate concentration was 1.5 mmol L [0.9 + 2.2]. Identification of the involved drug was obtained in 60% of the cases, based on qualitative screening. Management was mainly supportive and included sedation (13%), naloxone (10%) and flumazenil (1%). Tracheal intubation was required in 3 patients (0.4%). One cardiac arrest but no death occurred in the ED. Forty-three patients (6%) were transferred to the intensive care unit.

**Conclusion:** Our dataset provides an interesting insight into the drugs involved in and clinical pattern of toxicity outcome of acute recreational drug toxicity presentations at the ED, despite possible under-declaration and coding. Classical recreational drugs were more common (77%) followed by prescription drugs (20%) and NPS (3%).

### P-143 Report from the Paris supervised injection site: Is it this facility, the recent harm reduction approach towards drug use, safe?

#### Dusouchet Thomas^1^, Avril Elizabeth^1^, Plaisance Patrick^2^, Megarbane Bruno^2^

##### ^1^Gaia Association, Paris, France; ^2^Hôpital Lariboisière, Paris, France

###### **Correspondence:** Dusouchet Thomas - thomas.dusouchet@gaia-paris.fr

*Annals of Intensive Care* 2018, **8(Suppl 1):**P-143

**Introduction:** One year ago, the first French supervised injection site (SIS) was opened in Paris. SIS is a legally-sanctioned, medically-supervised facility designed to reduce nuisance from public drug use, providing hygienic and stress-free environment allowing intravenous injection and inhalation of illicit recreational drugs. A collaboration was set up between the SIS and the neighboring hospital to manage the medical complications resulting from drug use. Our objective was to describe the drug user profiles, the drug used and events requiring medical intervention, transfer to the emergency department and admission to the ICU.

**Patients and methods:** We conducted a prospective observational study including all patients who visited the SIS during the last 12 months. The collection of the usual anonymous demographic, medical and toxicological data was performed by the care-givers and social workers in charge of the drug users. Data were declarative and no analytical confirmation was available except for the patients admitted in the ICU.

**Results Discussion** During 10 months, 818 drug users [F M sex ratio 0.13 + median age 37.8 years (21–69) + patients without resources (40%), without medical insurance (27%), unstable housing homelessness (52%)] visited the SIS for drug injection or inhalation, representing 50,060 drug use including 14,587 inhalations and 35,473 injections by 180 drug users day. Drug users had no addictology (48%) or socio-medical (27%) follow-up. They were infected by hepatitis virus C (44%) and or HIV (5%). They declared to continue injecting in the public space (52%), sharing material (13%), and needles syringes (47%). The injected inhaled drugs in the SIS were Skenan^®^ (morphine, 42.6%), crack (43% including 1 3 injections), methadone (6.3%), buprenorphine (6.1%), heroine (1.2%), and cocaine (0.89%). These drugs were self-administered by polydrug users declaring concomitantly consuming crack (72.0%), illicit morphine (68.5%), cocaine (34.8%), ethanol (33.7%), cannabis (33.4%), heroin (29.9%), illicit methadone (20.4%), benzodiazepines (13.6%) and illicit buprenorphine (9.5%). Forty-five patients required a paramedical intervention in the SIS resulting in 17 calls to the emergency department and 15 hospital admissions including 2 transfers to the ICU in relation to opioid overdose. No cardiac arrest and no death occurred.

**Conclusion:** SIS visit for recreational drug self-administration rapidly becomes popular among drug users. Illicit morphine (Skenan^®^) represents the first self-administered drug. SIS seems safe, highlighting the effective collaboration set up with the neighboring hospital.

### P-144 Chronic digitalis poisoning treated with anti-digoxin Fab: a critical analysis of treatment indications and patient outcome

#### Guélou Calypso^1^, Bourgogne Emmanuel^1^, Chevillard Lucie^1^, Deye Nicolas^1^, Voicu Sebastian^1^, Megarbane Bruno^2^

##### ^1^Paris, France; ^2^Hôpital Lariboisière Hospital, Paris, France

###### **Correspondence:** Guélou Calypso - calypso.guelou@gmail.com

*Annals of Intensive Care* 2018, **8(Suppl 1):**P-144

**Introduction:** Incidence of digoxin poisoning has decreased, mainly including accidental chronic digoxin overdose to date. Indications, regimen dosage and usefulness of anti-digoxin Fab fragments to reverse chronic overdose remain controversial. Our objectives were to describe the management of digoxin-overdosed patients and evaluate outcome according to the treatment strategy.

**Patients and methods:** We conducted a retrospective observational study including all digoxin-intoxicated patients admitted to the ICU in a University Hospital from 2000 to 2016. The usual demographic, clinical, toxicological and outcome data were collected. Treatments including the dose regimen of anti-digoxin Fab fragments were decided by the physicians in charge. We analyzed the indications and modalities of the antidote administration and determined the predictive factors of death.

**Results:** Forty-nine patients (13 males 36 females + age—83 years [73 + 88] (median [percentiles 25 + 75], with past cardiac history (100%), renal (71%) and liver failure (4%) + digoxine dose—250 µg day [125 + 250] + plasma digoxin—4.10 nmol L [3.33 + 5.68]) were included. On ICU admission, patients presented nausea vomiting (53%), diarrhea (41%), confusion (33%), visual disturbances (18%), dizziness (16%), consciousness impairment (12%), agitation (12%), delirium (4%) and headaches (2%). Serum creatinine was 145 µmol L [105 + 226] and serum potassium 4.6 mmol L [4.1 + 5.3]. ECG showed slowed atrial fibrillation (47%), type I (20%), II (18%), III (8%) atrioventricular block and ventricular arrhythmia (2%). Patient management included anti-digoxin Fab (57%) with molar (31% + dose—160 mg [80 + 160] or semi-molar (27% + dose—80 mg [80 + 80]) dose regimen and mechanical ventilation (33% + duration—3 days [2 + 6]), catecholamine (47%) and hemodialysis (18%). The main observed complications were aspiration pneumonia (14%) and hospital-acquired infections (29%). Eight patients (16%) died in the ICU, mainly in relation to multi-organ failure. Based on an univariate analysis, only the presence of neuro-respiratory (p = 0.02), renal (p = 0.02) and liver (p = 0.05) failure on admission was predictive of death. Survival did not differ according to the serum digoxin concentration on admission or the Fab fragment dose administered (molar versus semi-molar).

**Conclusion:** Despite optimal management in the ICU, digoxin overdose is still responsible for life-threatening morbidities and an elevated mortality rate. If possible, the early anti-digoxin Fab administration at semi-molar dose regimen seems an efficient strategy to avoid organ failure while waiting the time for curative neutralization as usually recommended in the textbooks.

### P-145 Intensive care unit admission following the use of a new psychoactive substance (NPS): a case series in a Paris University Hospital

#### Haloua Noémie^1^, Malissin Isabelle^1^, Peron Nicolas^1^, Nuzzo Alexandre^1^, Voicu Sebastian^1^, Deye Nicolas^1^, Megarbane Bruno^2^

##### ^1^Paris, France; ^2^Hôpital Lariboisière, Paris-Diderot University, Paris, France

###### **Correspondence:** Haloua Noémie - noemie.haloua@aphp.fr

*Annals of Intensive Care* 2018, **8(Suppl 1):**P-145

**Introduction:** The new psychoactive substances (NPS) are increasingly used in the recearional scene while severe poisonings may occur requiring admission to the intensive care unit (ICU). The synthetic cathinones are becoming popular amphetamine-like products representing a major health concern. To the best of our knowledge, no ICU series of NPS-poisoned patients have been reported. Our objective was to describe the clinical features, management and outcome of NPS-poisoned patients admitted to the ICU. When available, pharmacokinetic data have been obtained.

**Patients and methods:** Retrospective single center descriptive study including all NPS-poisoned patients admitted in one University Hospital ICU during 2011–2017. NPS use was based on the patient’s declaration and confirmed analytically if possible. The usual demographic, clinical, toxicological and outcome data were collected from the medical records.

**Results:** Twenty-four NPS-poisoned patients (23 males 1 female + age—34 years [18] (median [IQR] + chronic ethanol (77%) and drug (59%) consumers + HIV-infected (58%) and depressive (25%) patients) were admitted to the ICU. The main declared compounds were methylenedioxypyrovalerone (MDPV + N = 9), 4-methylethcathinone (4-MEC + N = 6), 3-methyl methcathinone (3-MMC + N = 3) and 4-methyl methcathinone (4-MMC + N = 3), more frequently used in drug mixtures sold as bath salts or in poly-intoxication with conventional illegal drugs (mainly cocaine and gamma-hydroxybutyrate). NPS was used in a recreational (71%), Chemsex (29%) or solitary practice (29%). Binge (63%) and intravenous (50%) self-administration was remarkable. Patients presented acute encephalopathy with psychomotor agitation (46%), confusion (38% + Glasgow coma score—14 [9]), hallucinations (33%), anxiety (17%), seizures (17%), myoclonus (13%) and stereotypes (13%). ECG typically showed sinus tachycardia (70%), QRS QT abnormalities (13%) and atrio-ventricular block (4%). Acute cardiac ischemia (17%) and dysfunction (13%), disseminated intravascular coagulation (8%) and multiorgan failure (17%) developed. Management was supportive including mechanical ventilation (25%, length—24 h [21]). One NBOMe-poisoned patient died in relation to post-anoxic encephalopathy due to hypoxic pre-hospital cardiac arrest resulting from sustained seizures.

**Conclusion:** NPS and mainly synthetic cathinones may be responsible for severe features resulting in ICU admission and organ failure. Management is supportive. Improving our knowledge on the clinical risks of NPS use may be helpful to better inform the users.

### P-146 Poisonings with the new-generation anticonvulsive drugs admitted to the intensive care unit: a case series with pharmacokinetic data

#### Kandas Sylvie^1^, Chevillard Lucie^1^, Malissin Isabelle^1^, Gourlain Hervé^1^, Soichot Marion^1^, Megarbane Bruno^1^

##### ^1^Hôpital Lariboisière, Paris, France

###### **Correspondence:** Kandas Sylvie - sylvie.kandas@gmail.com

*Annals of Intensive Care* 2018, **8(Suppl 1):**P-146

**Introduction:** New-generation anticonvulsive drugs (NGAD) have been recently marketed due to improved efficiency and safety in comparison to the 1st generation. However, few data regarding NGAD poisoning have been published to date. Our objective was to report the clinical and biological toxicity attributed to NGAD overdose and, if available, describe NGAD pharmacokinetics.

**Patients and methods:** We conducted a retrospective single-center descriptive study including all NGAD-poisoned patients admitted to the intensive care unit (ICU) of a University Hospital over 10 years (2007–2017). The usual demographic, clinical, toxicological, and outcome data were collected from the patients’ records. Pharmacokinetics modeling and parameters were obtained using WinNonlin v.5.1 software.

**Results:** Twenty-one patients (9 males 12 females + age—44 years [33 + 51] (median [percentiles 25, 75] + depressive patients, 76%) were included in the study. The involved NGAD were lamotrigine (N = 10, 48%), levetiracetam (N = 5, 24%), topiramate (N = 4, 19%), lacosamide (N = 1, 5%) and oxcarbazepine (N = 1, 5%). The intoxication resulted from a multi-drug ingestion (N = 20, 95%). On admission, patients presented consciousness impairment (33% + Glasgow coma score—11 [3 + 15]), hypotension (systolic blood pressure—104 mmHg [99 + 119]) with gastrointestinal troubles (N = 6, 28%), seizures (N = 4, 19%), vertigo (N = 3, 14%), fatigue (N = 2, 9%), agitation (N = 2, 9%), headaches (N = 1, 5%), diplopia (N = 1, 5%), nystagmus (N = 1, 5%) and myoclonus (N = 1, 5%). Biological disturbances included increased plasma lactate concentrations (2.9 mmol L [1.5 + 5.9]), moderate hypoxemia (PaO2 FiO_2_—242 mmHg [165 + 398]), mild hypokalemia (3.2 mmol L [2.6 + 3.7]), mild increased alanine aminotransferase (49 IU L [28 + 99]) and rhabdomyolysis (creatine phosphokinase—271 IU L [65 + 653]). Management included mechanical ventilation (N = 16, 76%), catecholamine infusion (N = 8, 38%) and 8.4% sodium bicarbonate infusion (N = 5, 24%). A lancosamide-intoxicated patient died in relation to membrane-stabilizing effects resulting in acute cardiac failure. The pharmacokinetic analysis showed limited alterations in the parameters (half-life and clearance) in comparison to the pharmacological situation, highlighting the important consequences of poisoning-induced organ failure.

**Conclusion:** Despite marked safety at pharmacological doses, NGAD overdose may result in life-threatening poisoning requiring ICU admission and even fatality. The pharmacokinetic parameters seem more related to blood volume and organ function rather than the ingested NGAD dose.

### P-147 Epidemiology and resistance of multidrug resistant bacteria (MDRB) at the military hospital Avicenna in Marrakech (morocco)

#### Qamouss Youssef^1^, Aissaoui Younes^1^, Boughalem Mohamed^1^

##### ^1^Hopital Militaire Avicenne, Marrakech, Maroc

###### **Correspondence:** Qamouss Youssef - yqamouss@yahoo.fr

*Annals of Intensive Care* 2018, **8(Suppl 1):**P-147

**Introduction:** The bacterial multiresistance is a big medical problem nowdays because of morbidness and mortality whitch it procreates principally in the circle of intensive care department.

**Patients and methods:** It is about a retrospective descriptive study of a multidrug resistant bacteria identified from the registers of bacteriology of the department of microbiology in the central laboratory of analysis of the military hospital Avicenna. The objective of this study was to represent the epidemiological contour and resistance of multidrug resistant bacteria during years of 2015 and 2016.

**Results:** The results show that among the 9042 bacteriological samples treated for during years of 2015 and 2016, 1356 were positive and 215(16%) concerned MDRB. Extended spectrum betalactamase producing enterobacteriaceae was the most often isolated MDRB (65.11%), followed by Acinetobacter Baumanni resistant to Imipenem (26.04%), Staphylococcus aureus resistant to meticillin (7%), and finally *Pseudomonas aeruginosa* resistant to Ceftazidim (2%). According to types of samples, 44, 18% of MDRB has been identified among cytobacteriological examination of urine, followed by distal protected samples (22.8%). Intensive care department was at the origin of the majority of identified MDRB (43, 29%). The sharing out according to sex showed that 66% of subjects were of male sex. Extended spectrum beta lactamase producing Enterobacteriaceae they were mainly represented by *Escherechia coli* (44.20%), *Klebsiella pneumonia* (32.60%), then *Enterobacter cloacae* (21%) witch 5 bacterial stocks among this one were Carbapenemase producing Enterobacteriaceae. The study of resistance of Extended spectrum betalactamase producing Enterobacteriaceae revealed a rate of resistance of 86.95% to ciprofloxacin, 89.13% to Norfloxacin, 80.43% to the association Trimethoprim-sulfamethoxazole, 2.17% to Imipenem, 10, 86% to Amikacin, and 50% to Gentamicin, while all bacterial stocks were sensitive to Colistin. Concerning Acinetobacter baumanni Imipenem resistant, and *P. aeruginosa* Ceftazidim resistant, resistance was respectively, 77 and 10%, while all bacterial stocks were sensitive to Colistin. No Staphylococcus aureus Methicillin resistant was resistant to glycopeptides.

**Conclusion:** In the light of these results, specific control measures are particularly recommended against the main MDRB found, in the most affected departments. The rationalization of the prescription of antibiotics and the implantation of a monitoring system for MDRB are measures which their urgent implementation is strongly recommended in order to limit the emergence of new MDRB stocks in our hospital departments.

### P-148 Risk factors for bronchial colonization with extended spectrum beta-lactamase producing enterobacteriaceae among fecal carriers

#### Kugener Luc^1^, Roisin Sandrine^1^, Kohnen Michel^1^, Coussement Julien^1^, Brasseur Alexandre^1^, Creteur Jacques^1^, Jacobs Frederique^1^, Grimaldi David^1^

##### ^1^Hopital Erasme, Bruxelles, Belgique

###### **Correspondence:** Kugener Luc - lkugener@gmail.com

*Annals of Intensive Care* 2018, **8(Suppl 1):**P-148

**Introduction:** Extended spectrum beta lactamase-producing enterobacteria (E-ESBL) have spread across Europe and the world over the last 20 years. They are growing in importance as a source of nosocomial infections, especially in the intensive care unit (ICU). This leads to increased consumption of carbapenems. It is unclear whether patients colonised by E-ESBL in the digestive tract should be empirically treated by carbapenems when they develop intensive care unit-acquired pneumonia. The goal of our study was to identify risk factors associated with lower airway colonisation by E-BLSE among digestive carriers.

**Patients and methods:** We conducted a retrospective monocentric study in the ICU of a tertiary care university hospital from January 2012 until June 2014. Screening for E-ESBL by rectal swab and respiratory cultures was systematically done at admission then twice weekly until discharge. We included all patients with a positive rectal swab during their ICU stay. Exclusion criteria were an ICU stay of less than 48 h and known respiratory E-ESBL colonisation prior to ICU-admission.

**Results:** 243 patients were included. Median age was 63 years (48–71) and 64% were male. Median Apache II score was 20 (15–27) and median ICU length of stay was 8 days (5–14). 65.8% of patients were mechanically ventilated and we observed a 31% in-hospital mortality rate. Most patients were colonised by rectal ESBL at admission (median delay between ICU admission and colonisation 1 day (0–4.5)). The ESBL species were mostly E. Coli (151 isolates, 62%) followed by Klebsiella (48 isolates, 20%) and Enterobacter sp. (37 isolates, 15%). 19 patients acquired tracheal E-ESBL colonisation (7.8%) and 5 of them developed a pneumonia caused by E-ESBL. In univariate analysis, mechanical ventilation, higher APACHE-2 score and non-Coli species were associated with tracheal ESBL colonization (p < 0.05). In exploratory multivariate analysis, mechanical ventilation and non *E. coli* sp. were associated with tracheal ESBL colonisation. Interestingly, none of the fecal carriers of *E. coli* ESBL without mechanical ventilation developed tracheal colonization.

**Conclusion:** Bronchial colonization by E-ESBL is rare among fecal carriers in the ICU. Mechanical ventilation and non-*E. coli* E-ESBL seem to be the main risk factors for tracheal colonization. Among carriers of *E. coli* ESBL not treated by mechanical ventilation and developing ICU-acquired pneumonia it seems reasonable not to cover them with carbapenems. Further studies are necessary to confirm this finding.

### P-149 The consumption of antibiotics in a Moroccan medical intensive care unit and Clinical Relevance of the ESKAPE Pathogens

#### Ezzouine Hanane^1^, Eljadid Siham^2^, Kerdoud Ouassime^2^, Benslama Abdellatif^2^

##### ^1^CHU IBN Rchod, Casablanca, Maroc; ^2^UNIVERSITY HASSAN II, Casablanca, Maroc

###### **Correspondence:** Ezzouine Hanane - ezzouinehanane@yahoo.fr

*Annals of Intensive Care* 2018, **8(Suppl 1):**P-149

**Introduction:** The emergence of bacterial resistances to antibiotics is clearly increasing, especially in the intensive care units. It is mainly due to excessive or inappropriate use of antibiotics. Hence the importance of monitoring consumption and evaluating prescribing practices. Furthermore, the ESKAPE pathogens are increasing relevance to antimicrobial chemotherapy. We aimed in this study to make an inventory of the consumption of the antibiotics in our unit especially for the ESKAPE pathogens and to define the epidemiological and clinical characteristics of the patients.

**Patients and methods:** we carried out a retrospective descriptive and analytical study, over a year involving all patients hospitalized in the medical intensive care unit of university teaching hospital Ibn Rushd Casablanca during the 2015. Data collection was based on a mining record.

**Results:** 190 patients received antibiotics. Its prescription incidence was 53.37%. The average length of hospitalisation was 18.45 days. Antibiotic therapy was started before admission in 42.10% of patients. After admission, 53.37% of patients received antibiotic therapy. Curative antibiotic therapy was probabilistic in 90.5% of cases based on clinical, biological, radiological and ecological arguments. 6.3% of initial prescriptions were directed to bacteriological results and 3.2% of initial prescriptions were prophylactic. The infection was predominantly respiratory infections (62.10%), but nosocomial infection accounted for 37.89%. Third generation cephalosporins represented the most frequently prescribed molecules (73.68% of cases) followed by quinolones and aminoglycosides. 41.6% of patients received monotherapy, while 58.4% of patients received a combination of antibiotics. The mean duration of curative antibiotic therapy was 5.67 days. The most frequently isolated seeds in the samples were Acinetobacter baumannii, *Pseudomonas aeruginosa* and *Klebsiella pneumonia*e. Progression with antibiotics was favorable in 57.9% of patients. The prognosis in univariate analysis depended on the severity scores on admission (APACHE II, SAPS II, SOFA), intubation mechanical ventilation, bladder sampling, antibiotic pre-treatment, nosocomial infection and nature of the germ—ESKAPE pathogens. In multi-variate analysis of APACHE II score, SOFA score, pathogens and nosocomial infection were associated with significant excess mortality.

**Conclusion:** A constant reassessment of the prescribing practices regains its interest as well as the optimization of the prescription modalities. Restrictions on the use of certain molecules, the use of written protocols, the use of a computer tool and the establishment of a system of performance indicators would be effective strategies for improvement.

### P-150 Impact of the transfer of an ICU to new buildings on local bacterial ecology

#### Ouanes Islem^1^, Kotti Amine^1^, Hraiech Kmar^1^, Tlili Meriem^1^, Kadri Yosr^1^, Hammouda Zaineb^1^, Nouira Wiem^1^, Bouker Nouha^1^, Zorgati Hend^1^, Ouni Amal^1^, Boukadida Sana^1^, Dachraoui Fahmi^1^, Ouanes-Besbes Lamia^1^, Mastouri Maha^1^, Abroug Fekri^1^

##### ^1^CHU Fattouma Bourguiba, Monastir, Tunisie

###### **Correspondence:** Ouanes Islem - ouanes.islem@gmail.com

*Annals of Intensive Care* 2018, **8(Suppl 1):**P-150

**Introduction:** Assessment of local bacterial ecology is of paramount importance for the prevention and management of infections in intensive care units, especially with the emergence of multidrug resistant (MDR) bacteria causing a real problem worldwide. The aim of this study was to describe the patterns of bacterial ecology and resistance in our ICU before and after transfer to new buildings.

**Patients and methods:** This was a cohort study including consecutive positive clinical samples from patients admitted to our ICU before and after transfer to new buildings (March 2016), the first period (P1) from 1st March 2015 to 1st March 2016 and the second one (P2) from 1st March 2016 to 1st March 2017. Positive samples and patients characteristics were prospectively collected from the Microbiology Unit and the ICU databases respectively. The number of positive samples for different micro-organisms was reported to the number of patients (density) for each period and compared Spearman’s correlation coefficient test.

**Results:** During the first and the second study periods 204 and 244 patients were respectively admitted in the ICU. Total micro-organisms density was 48 and 69.5 for 100 patients for the first and the second period, respectively (p < 0.05). Acinetobacter spp and Pseudomonas Aeroginosa were the predominant isolated microorganisms with a respective density of 13.2 and 11.2 isolates for 100 patients. Figure 1 summarizes the patterns of bacterial ecology and resistance in our ICU before and after transfer to new buildings, showing a significant decrease in Pseudomonas aeroginosa resistance for ticarcillin and ceftazidim, whereas Acinetobacter resistant to carbapenems and Enterobacteriacae ESBL significantly increased.

**Fig. 1:**
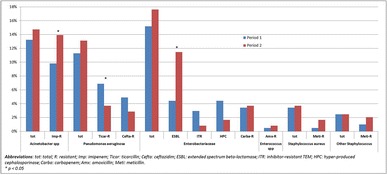
Patterns of bacterial ecology and resistance in our ICU before and after transfer to new buildings

**Conclusion:** Our study suggests that transfer of ICU to the new buildings was associated with a decrease of Pseudomonas aeroginosa resistance, whereas Acinetobacter spp resistance and ESBL Enterobacteriacae incidence increased.

### P-151 Systematic AMRB screening’s impact on empiric antimicrobial therapy in critical care

#### Boujelben Mohamed Ahmed^1^, Kallel Hela^1^, Fathallah Ines^1^, Sakis Dorra^1^, Habacha Sahar^1^, Tobich Mariem^1^, Ben Marzouk Rihem^1^, Anan Sonia^1^, Ben Salah Nawel^1^, Kouraichi Nadia^1^

##### ^1^Hôpital Régional de Ben Arous, Ben Arous, Tunisie

###### **Correspondence:** Boujelben Mohamed Ahmed - m.ahmedboujelben@gmail.com

*Annals of Intensive Care* 2018, **8(Suppl 1):**P-151

**Introduction:** Infections caused by antimicrobial-resistant bacteria (AMRB) are one of the main issues in the spectrum of critically ill patients as they are associated with higher mortality, morbidity, and length of stay. Thus, an appropriate initial antimicrobial therapy is decisive for better patient outcomes. The aim of the study is to determine the adequacy of first-line antibiotic therapy guided by weekly AMRB screenings.

**Patients and methods:** A 9 months prospective study was conducted in 6-bed MICU. Were included all patients with more than 48H of ICU stay. An AMRB screening was conducted upon admission and on weekly basis for all the patients. The choice of antibiotherapy if indicated, was guided by the most recent colonization results. If the patient has received at least one active in vitro antibiotic against the isolated bacteria, the empiric antibiotherapy was considered appropriate.

**Results:** 98 patients were included in the study. Mean age and SAPS II were respectively 56 ± 20 years and 41 ± 20. The median length of stay was 8 days. 11 (11%) patients were colonized by AMRB upon admission. The most frequent isolated microorganisms were—*Escherichia coli* 5 (55%) and *Klebsiella pneumonia* 4 (45%). Were assessed 57 hospital-acquired infections (HAI)—32 (56%) in AMRB colonized patients and 25 (44%) in uncolonized ones. The antibiotherapy was considered appropriate in 21 infections (65%). Out of the 20 colonized patients, 15 (75%) developed HAI. 11 (73%) patients had a concordant colonization body site to the infection. Of the 57 nosocomial infections, ventilator-associated pneumonias and central venous catheter infections were the most frequent, both at 38% (n = 22 and n = 22) + followed by urinary tract infections 21% (n = 10) and infective endocarditis 2% (n = 1). The commonest isolated pathogens were—enterobacteriaceae 22 (37%), *Pseudomonas aeruginosa* 20 (34%) and Acinetobacter baumannii 15(26%).

**Conclusion:** The study demonstrated the benefits of conducting systematic AMRB screenings in critically ill patients, as well as the emerging trend of hospital-acquired infections in our MICU.

### P-152 Performance of Screening for Multi-drug Resistant Bacteria in the Prediction of Nosocomial Infections

#### Trifi Ahlem^1^, Mhajba Mohamed Walid^1^, Zribi Meriem^1^, Daly Foued^1^, Touil Yosr^1^, Nasri Rochdi^1^, Abdellatif Sami^1^, Fandri Chadlia^1^, Ben Lakhal Salah^1^

##### ^1^Hopital La Rabta Jebbari 1007 Tunis, Tunis, Tunisie

###### **Correspondence:** Trifi Ahlem - trifiahlem2@gmail.com

*Annals of Intensive Care* 2018, **8(Suppl 1):**P-152

**Introduction:** Nosocomial infection (NI) is due to the decrease of the patient’s immune defences and its colonization by pathogenic or potentially pathogenic bacteria. In presence of infectious signs, the knowledge of multi—drug resistant (MDR) bacteria colonization should be included in the choice of anti-infective therapy. We aimed to study the performance of MDR bacteria screening in the prediction of NI caused by these pathogens.

**Patients and methods:** a 4-month prospective study carried out in collaboration with the bacteriology department. All adult patients older than 18 years and hospitalized in our unit with a stay exceeding 48 h were included. MDR bacteria screening consisted of performing a series of swabs on admission and renewed weekly. The sites were—nasal, cutaneous, rectal and urinary. Additional bacteriological samples were taken in presence of infectious signs. The group of true positives were those with a positive test (at least one MDR bacteria isolated in the series of samples) and having a documented NI to MDR bacteria.

**Results:** 112 screening tests were performed in 40 patients with a median age to 47 years, and 1.1 of sex ratio. 71 screening tests were positive isolating 142 MDR resulting in MDR bacteria colonized group (n = 71) versus non MDR bacteria colonized group (n = 41). The naso-pharyngeal was the most colonized site (57 MDR bacteria) followed by the rectal site (42). Overall, the isolates were—extended spectrum betalactamase productrice-Enterobacteria (43%), imipenem resistant-Acinetobacter baumanii (30%), and multi resistant-Pseudomonas aeroguinosa (19%). 50 NI were documented including 35 caused by MDR bacteria and distributed as follows—ventilator acquired pneumonia—VAP (n = 19), bacteraemia (n = 6), VAP with bacteraemia (n = 4), catheter related infection—CRI (n = 3), CRI with VAP (n = 2) and catheter-related bacteraemia—CRB (n = 1). The performance of MDR bacteria-screening in predicting NI was poor with 71% of sensitivity, 58% of specificity, 41.5% of negative predictive value (NPV), and 83% of positive predictive value (PPV). Nevertheless, the performance of the nasal swab in the prediction of VAP was better with 85% of sensitivity and 92.3% of NPV.

**Conclusion:** MDR bacteria-screening is useful as it allows to identifying the MDR bacteria-carriers and helps for a rational use of antibiotics in severe NI. However, its diagnostic contribution in the occurrence of NI is poor except the interest of the nasal swab in the prediction of VAP owing to its good NPV.

### P-153 Risk factors for subsequent infection among patients with extended-spectrum beta-lactamase-producing Enterobacteriaceae colonization: a prospective study

#### Van Der Meersch Guillaume^1^, Le Flécher Arnaud^1^, Tandjaoui-Lambiotte Yacine^1^, Saniez Thibault^1^, Martin Olivier^1^, Oziel Johanna^1^, Karoubi Philippe^1^, Blanchard Florian^1^, Popoff Benjamin^2^, Billard-Pomares Typhaine^1^, Zahar Jean-Ralph^1^, Cohen Yves^1^

##### ^1^CHU Avicenne, Bobigny, France; ^2^Rouen DAR, Rouen, France

###### **Correspondence:** Van Der Meersch Guillaume - guillaume.vandermeersch@aphp.fr

*Annals of Intensive Care* 2018, **8(Suppl 1):**P-153

**Introduction:** Intestinal carriage of extended-spectrum beta-lactamase-producing Enterobacteriaceae (ESBL-PE) is a common issue in ICU patients, and may be associated with subsequent infection. While ESBL-PE related infections rarely occur in patients without prior colonization, infections occurring among colonized patients may be due to other less resistant pathogens. In this view, it would be interesting to be able to predict, which colonized patients will actually develop ESBL-PE related infections, so as to control the increasing use of carbapenems that may jeopardize their efficacy in a near future. The aim of this study was to identify risk factors for subsequent infection among ESBL-PE colonized patients.

**Patients and methods:** We performed a prospective study in a 16-bed ICU of a French universitary hospital. All patients with ESBL-PE colonization were considered. Rectal swabs were performed at admission, thereafter on a weekly basis and at ICU discharge. The following data were collected—age, sex, severity scores, admission category (medical or surgical), underlying diseases, antibiotic exposure within 3 months before ICU admission, invasive procedures, type of ESBL-PE, and outcome. The number of infected patients without prior colonization was also recorded. Colonized patients with and without subsequent infection were first compared by univariate analyses. Then, we performed a multivariate logistic regression model to identify independent risk factors for ESBL-PE infection.

**Results:** A total of 291 colonized patients were screened from September 2015 to August 2017. Among them, 233 (80.0%) were colonized at ICU admission. *Escherichia coli* (199, 68.0%), *Klebsiella pneumonia* (60, 20.6%) and *Enterobacter cloacae* (40, 13.7%) accounted for 89.0% of species recovered from rectal swabs. Only 7 (2.4%) patients developed ESBL-PE infection without prior colonization. Among 284 prior colonized patients, 27 (9.5%) developed subsequent infections. Independent risk factors for subsequent infection among colonized patients were—prior hospitalization abroad (OR 8.94 [1.17–84.67] p = 0.03), presence of urinary catheter (OR 4.41 [1.16–18.03] p = 0.03) and prior exposure to third-generation cephalorins (OR-3.15 [1.09–10.41] p = 0.04).

**Conclusion:** We were able to identify three simple risk factors that may be considered in clinical practice to reduce carbapenems use as first line antibiotic therapy in case of severe sepsis or septic shock in patients with known ESBL-PE colonization. Our results have to be confirmed by further multicenter prospective evaluations.

### P-154 Screening for esbl enterobacteriacee by rectal swab: Is it worth?

#### Baudel Jean-Luc^1^, Tankovic Jacques^1^, Dahoumane Redouane^1^

##### ^1^Hôpital Saint-Antoine, Paris, France

###### **Correspondence:** Baudel Jean-Luc - jean-luc.baudel@aphp.fr

*Annals of Intensive Care* 2018, **8(Suppl 1):**P-154

**Introduction:** Over the last decade, the incidence of multidrug-resistant Gram-negative bacteria (MDR-GNB), notably Enterobacteriaceae producing extended-spectrumβ-lactamases (ESBL) and carbapenemases, has increased steadily. The question as to when hygiene screening cultures (HS) should be performed to identify MDR-GNB colonized patients is still debated. In our medical ICU we perform a targeted screening of high-risk patients. The aim of this study was to evaluate the relevance of this screening policy.

**Patients and methods:** We retrospectively analyzed (from January 2014 to April 2017) all cases of isolation of ESBL or carbapenemase producing Enterobacteriaceae in a previously naive patient during his her stay in our ICU, either by HS or clinical samples. Except for patient coming from known endemic area unit, hygiene screening was performed, when the clinician in charge of the patient felt it necessary. Hygiene screening cultures were done by plating rectal swabs on bioMerieux ESBL and Chromid Carbasmart plates and incubating the plates for 18–24 h.

**Results:** During the analysis period, 166 naive patients were detected positive by either hygiene screening or clinical samples or both. OXA-48 producing strains was found in 2 patients, and were detected by both clinical samples and hygiene samples. Among the164 patients found during their ICU stay to carry ESBL producing strains, no hygiene screening had been performed in 64 of them (39.0%). Among the 100 patients detected as ESBL positive 44 of them were BLSE positive only by rectal swab + 32 were positive by both hygiene screening and clinical cultures and 24 patients were hygiene screening negative, clinical culture-positive. In 12 of these latter 24 cases, clinical cultures became positive at least 5 days after hygiene sampling, making data difficult to interpret. The remaining 12 cases were more likely to be “real” false-negatives hygiene samples, because clinical samples (urines 6, respiratory samples, 4, blood cultures 2) were positive either before (1 to 7 days, 6 cases), concomitantly (3 cases) or not more than 3 days (1, 2 and 3 days, 3 cases) after hygiene screening.

**Conclusion:** For almost 40% of the new ESBL positive patients, no hygiene sample (rectal swab) had been performed. This raises questions about the fiability of targeted screening. Hygiene samples are negative in approximately 10% of patients with positive clinical samples. The sensitivity of surveillance screening could be improved, by the use of molecular techniques.

### P-155 Physiological determinants of the respiratory variations of the inferior vena cava diameter in ventilated critically ill patients

#### Jozwiak Mathieu^1^, Mercado Pablo^1^, Teboul Jean-Louis^1^, Depret François^1^, Richard Christian^1^, Monnet Xavier^1^

##### ^1^CHU Bicêtre, Le Kremlin-Bicêtre, France

###### **Correspondence:** Jozwiak Mathieu - mathieu.jozwiak@aphp.fr

*Annals of Intensive Care* 2018, **8(Suppl 1):**P-155

**Introduction:** The respiratory variations of the inferior vena cava (IVC) diameter allow reliably predicting fluid responsiveness in critically ill patients under mechanical ventilation. Two mechanisms could be involved to explain this phenomenon—a higher compliance of the IVC and or a higher respiratory variations of the IVC backward pressure, in other words, the central venous pressure (CVP), when hypovolemia. We aimed at determining the respective weight of these phenomenon and the physiological determinants of the respiratory variations of the IVC diameter.

**Patients and methods:** In 21 mechanically ventilated patients (tidal volume—6.2 ± 1.0 mL kg of predicted body weight) haemodynamic, respiratory and the intra-abdominal pressure (IAP) signals were continuously computerised. CVP, IAP and the IVC diameter (transthoracic echocardiography) were recorded during 15-second end-inspiratory and end-expiratory occlusions separated by 10 s, before and after the infusion of 500-mL of saline. Patients in whom fluid administration induced an increase in cardiac index (PICCO-2) > 15% were defined as “responders”. The respiratory variations of the IVC diameter, CVP and IAP were calculated as the (end-inspiratory—end-expiratory values) mean value. The compliance of the IVC was estimated by the ratio (end-expiratory—end-inspiratory IVC diameter) (end-expiratory—end-inspiratory CVP).

**Results:** Fluid administration increased cardiac index by more than 15% (3.09 ± 1.12 to 3.90 ± 1.42 L min m^2^, p = 0.0003) in 8 patients. The respiratory variations of the IVC diameter predicted fluid responsiveness (area under the ROC curve—0.822 (95% CI 0.595–0.952), p < 0.05). Before fluid administration, the ratio of changes in IVC diameter over changes in CVP was not different between responders and non-responders (0.73 ± 0.34 vs. 0.88 ± 1.25 mm mmHg, p = 0.75). Before fluid administration, the respiratory variations of the CVP tended to be higher in responders than in non-responders (34 ± 27 vs. 18 ± 8%, p = 0.07). The respiratory variations of the IVC diameter were associated with the respiratory variations of CVP (r = 0.56, p = 0.01) but not with the respiratory variations of IAP (r = -0.15, p = 0.52).

**Conclusion:** The respiratory variations of the IVC diameter were not explained by a higher IVC compliance but rather by higher respiratory variations of the CVP in responders than in non-responders. Interestingly, it seems that IAP, the IVC extramural pressure, was not involved in the respiratory variations of the IVC diameter. Inclusions are ongoing.

### P-156 Computerized simulation accelerates the learning curve for basic critical care transthoracic echocardiography

#### Pegot Benjamin^1^, Dalmay François^1^, Jean-Michel Vanessa^1^, Baudrier Fabien^1^, Depays Faustine^1^, Charpentier Mathieu^1^, Le Mée Pauline^1^, Chatenet Emeline^1^, Dargere Marine^1^, Danthu Clément^1^, Lanfranco Luca^1^, DubosMaria^1^, Langlais Marie-Line^1^, Gauthier François^1^, Le Roy Julie^1^, Guilbault Pierre^1^, Rezig Schéhérazade^1^, Ravry Céline^1^, Bocher Simon^1^, Renaudeau François^1^, Le Mao Raphaël^1^, Saint-Paul Aude^1^, Durand Bénédicte^1^, Tran Van Nho Jessica^1^, Geslain Marie^1^

##### ^1^CHU Dupuytren, Limoges, France

###### **Correspondence:** Pegot Benjamin - b.pegot@gmail.com

*Annals of Intensive Care* 2018, **8(Suppl 1):**P-156

**Introduction:** Basic critical care echocardiography (CCE) should be mastered by intensivists and relies on transthoracic echocardiography (TTE). Specific curricula have been previously validated. Potential additional value of computerized simulation on the learning curve of basic CCE remains unknown.

**Patients and methods:** During 2 years, residents of two Intensive Care Units (ICUs) novice in ultrasound participated in this bi-center study. All residents underwent the same training incorporating theoretical courses (6 h) and hands-on sessions (2 h). Unlike residents in ICU B (control group), all residents trained in ICU A (simulation group) underwent two 2-h sessions of computerized simulation of TTE during the first month of training (Vimedics, CAE Healthcare). Trainees performed and interpreted TTE at bedside and filled a standardized CRF. Each trainee was evaluated using a predefined scoring system (practical, diagnostic, technical, interpretation skills) in two patients with cardiopulmonary compromise at the end of the first (M1), third (M3) and sixth (M6) months. A score of > 48 54 (90% of maximal score) defined competency.

**Results:** 26 residents participated in the study (simulation group—n = 14 + control group—n = 12), with a similar intergroup profile (specialty, year of residency) and performed 965 TTE. In total, 156 evaluations were performed in 106 patients with similar characteristics in both ICUs (age—53 ± 19 years + SAPSII—55 ± 19 + 80% ventilated + 60% under catecholamines). Mean evaluation score was higher in the simulation group at M1 (41.5 ± 4.9 vs. 32.3 ± 3.7—p = 0.0004) and M3 (45.8 ± 2.8 vs. 42.3 ± 3.7—p = 0.0223), but not at M6 (49.7 ± 1.2 vs. 50.0 ± 2.7—p = 0.6410) (Figure). Specifically, the practical and technical skills were improved at M1 and M3, but neither the diagnostic nor the interpretation skills. Mean number of TTE performed was similar in both groups. Less supervised TTE were required to obtain the competency score in the simulation group (35 ± 3 vs. 39 ± 3—p < 0.0487). A score of 47 54 predicted concordance of both interpretation of TTE and suggested therapeutic change between referees and tutors with a sensitivity of 83% [IC95% 67–91] and a specificity of 63% [IC95% 39–81] (AUC-0.79 + p < 0.0001).
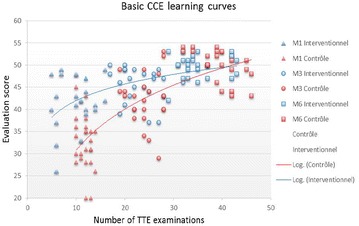


**Conclusion:** Computerized simulation accelerates the initial learning curve of training in basic level CCE and reduces the number of examinations required to reach the competency score. Nevertheless, it failed to increase the mean evaluation score at M6 and both the diagnostic and interpretation skills at M3 and M6.

### P-157 Early high-dose erythropoietin therapy and post-cardiac arrest kidney injury: a post hoc analysis of a randomized clinical trial

#### Guillemet Lucie^1^, Jamme Matthieu^1^, Geri Guillaume^1^, Deye Nicolas^1^, Vivien Benoit^1^, Varenne Olivier^1^, Pène Frédéric^1^, Barat Florence^1^, Treluyer Jean-Marc^1^, Hermine Olivier^1^, Carli Pierre^1^, Coste Joël^1^, Cariou Alain^1^

##### ^1^CH Victor Dupouy, Paris, France

###### **Correspondence:** Guillemet Lucie - lucie.guillemet@aphp.fr

*Annals of Intensive Care* 2018, **8(Suppl 1):**P-157

**Introduction:** After resuscitation of an out-of-hospital cardiac arrest (OHCA), acute renal injury (AKI) is a frequent complication, which contributes to the high mortality observed in this setting. Experimental data suggest a renal protective effect in treating OHCA patients with high-dose of erythropoietin analogues. We aimed to evaluate the efficacy of erythropoietin treatment on renal outcome of OHCA patients.

**Patients and methods:** We did a post hoc exploratory analysis on patients admitted in the medical intensive care unit at Cochin Hospital (Paris, France) and who were included in the EPO-ACR-02 trial. In this trial, OHCA patients still comatose after a witnessed OHCA of presumed cardiac origin were randomized. In the intervention group, patients received 5 intravenous injections (40,000 units each) spaced 12 h apart during the first 48 h, started as soon as possible after resuscitation. In the control group, patients received standard care without Epo. The main endpoint of the present analysis was the proportion of patients with AKI defined on Kidney Disease Improving Global Outcomes (KDIGO) criteria at the 48th hour. Secondary end-points included the distribution of patients in each KDIGO level within the first 48 h among the two groups, the occurrence of AKI through day-28 or ICU discharge, the presence of AKI at the 48th hour using only the creatinine criteria of the KDIGO classification, the hematopoietic effects of Epo through day-28 or ICU discharge, the day-28 glomerular filtration rate estimated with the MDRD equation, and side effects.

**Results:** In total, 162 patients were included in the primary analysis. Baseline characteristics were similar in the 2 groups. At the 48th hour, 52.8% of the patients in the intervention group had an AKI, as compared with 54.4% of the patients in the control group (p = 0.74). During the hospitalization in ICU, there was no significant difference between the two groups regarding the proportion of patients with AKI through ICU discharge. In the intervention group, 28% of the patients had a glomerular filtration rate lower than 75 mL min 1.73 m^2^ compared to 14.8% in the control group (p = 0.25) at day-28. We found no significant difference between the two groups neither on hematopoietic effects of Epo or serious adverse events.
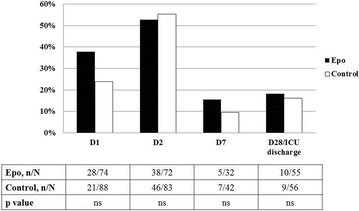


**Conclusion:** In patients resuscitated from an OHCA of presumed cardiac cause, early administration of erythropoietin compared to standard therapy did not confer any renal protective effect.

### P-158 Is Transthoracic Echocardiography (TTE) able to predict a poor hemodynamic tolerance (PT) to intermittent hemodialysis (IH)?

#### Salvetti Marie^1^, Augustin Pascal^1^, Laborne François-Xavier^2^, Allyn Jérôme^3^, Allou Nicolas^3^, Montravers Philippe^1^, Tashk Parvine^1^

##### ^1^Hôpital Bichat, Paris, France; ^2^SAMU 91, Corbeil-Essonness, France; ^3^Hôpital Félix Guyon, Saint Denis Réunion, France

###### **Correspondence:** Salvetti Marie - marie.salvetti@gmail.com

*Annals of Intensive Care* 2018, **8(Suppl 1):**P-158

**Introduction:** IH can be poorly tolerated from the hemodynamic standpoint despite the observance of recommendations that minimize these side effects in intensive care. Each episode of hypotension is deleterious and can harm the recovery of renal function or other organs. TTE is a non-invasive hemodynamic assesment tool, already assessed in this indication with inconclusive results. The aim of the study is to define echocardiographic criteria for PT to IH.

**Patients and methods:** Prospective observational monocentric study in an intensive care unit. All sessions of IH were included and several sessions could be included per patient. The patients having an hemodynamic failure with a SOFA score > 3 were excluded. A TTE was performed before the session. The PT was defined by—hypotension, arrhythmia episode, need for volume replacement, to increase the dose of catecholamines, or to suspend the session. The clinical and echocardiographic parameters, selected according to the current literature, were compared for the tolerance to IH with Mann-Withney tests or Fisher exact tests.

**Results:** We included 41 sessions of IH in 9 months, 46% of which under mechanical ventilation. Only 15% of the patients had bad echogenicity. In 56% of the cases, the patient had a heart disease. The median SOFA score was 8 and the median planned depletion was 2 L. On these 3 parameters we found no differences between tolerant and intolerant patients to IH. There were 17 sessions of IH (46%) associated with PT. The following echocardiographic parameters were not significantly different in the 2 groups—altered left ventricular ejection fraction, inferior vena cava collapsibility, ratio E A, ratio E Ea. The increase of left ventricular outflow tract velocity time integral (LVOT VTI) after leg raising was significantly higher in intolerant patients (0.8 vs. 10.3%, p = 0.02, see Figure below).
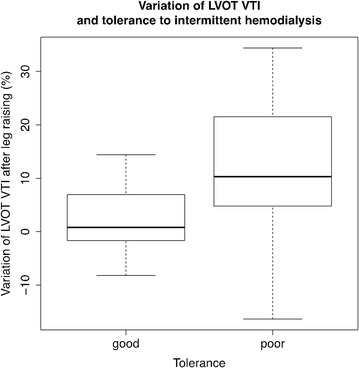


**Discussion:** The variation of the LVOT VTI is a criterion of preload dependence used to predict the response to volume expansion. Until now, no study had shown an association between this criterion and the PT to IH. In practice, anticipate a PT to IH would involve a better adjustment of the parameters of the IH, in particular the volume of depletion, to reduce its deleterious side effects.

**Conclusion:** The increase of LVOT VTI after leg raising measured before IH is significantly higher in patients having a PT to the session in our small cohort. It could be a predictive criteria of PT, that TTE would allow us to anticipate.

### P-159 Is it possible to predict the hemodynamic profile of ventilated patients with septic shock using front-line hemodynamic parameters?

#### Fedou Anne-Laure^1^, Repessé Xavier^2^, Riu Béatrice^3^, Gonzalez Céline^1^, Aubry Alix^2^, Jacob Christophe^2^, Bouferrache Koceila^2^, Kontar Loay^4^, Gwenaël Prat^5^, Goudelin Marine^1^, Evrard Bruno^1^, Jabot^6^, Julien, Daix Thomas^1^, Vandroux David^6^, François Bruno^1^, Silva Stein^3^, Maizel Julien^4^, Slama Michel^4^, Géri Guillaume^2^, Vieillard-Baron Antoine^2^, Vignon Philippe^1^

##### ^1^CHU Dupuytren, Limoges, France; ^2^Hôpita Ambroise Parée, Paris, France; ^3^CHU de Toulouse, France; ^4^CHU d’Amiens, France; ^5^CHU de Brest, France; ^6^CHU Félix Guyon, Saint Denis, France

###### **Correspondence:** Fedou Anne-Laure - anne-laure.fedou@chu-limoges.fr

*Annals of Intensive Care* 2018, **8(Suppl 1):**P-159

**Introduction:** The Surviving Sepsis Campaign 2016 recommends that hemodynamic assessment be serially performed during the initial management of septic shock patients. Front-line hemodynamic and metabolic parameters—blood pressure (BP), heart rate (HR), central venous pressure (CVP), mixed central venous oxygen saturation (ScvO2) and lactate—are widely used to guide early resuscitation. We sought to describe hemodynamic profiles in previously described cohorts of ventilated patients with septic shock assessed within 12 h of ICU admission using transesophageal echocardiography (TEE) and evaluate the diagnostic value of front-line hemodynamic parameters.

**Patients and methods:** In each patient, cardiac index (CI), left ventricular ejection fraction (LVEF), respiratory variations of the superior vena cava (deltaSVC) and of the subaortic Doppler velocities (deltaVmaxAo), and the ratio of end-diastolic areas of both the right and left ventricle in the long axis view of the heart (RVEDA LVEDA) were measured. A LVEF < 40% defined LV systolic dysfunction, a CI < 3 l min m^2^ defined low cardiac output, and a RVEDA LVEDA ratio > 0.6 (± associated with a paradoxical septal motion in the short axis of the heart) defined RV dysfunction (± acute cor pulmonale). The preload-dependence was evaluated using deltaSVC or deltaVmaxAo. Front-line hemodynamic and metabolic parameters were recorded at the time of TEE assessment.

**Results:** LVEF and CI could be simultaneously measured in 388 of 410 patients (95%). 74 patients (19%) had a low CI related to LV systolic dysfunction (lactate—4.36 ± 3.49 mmol l), 141 patients (36%) had a low CI and a preserved LVEF related to a RV dysfunction or to a sustained preload-dependence (lactate—3.58 ± 3.28 mmol l), 146 patients (38%) had preserved CI and LVEF (lactate—3.38 ± 3.32 mmol l) including only 23 patients (6%) with a hyperkinetic profile (high CI and LVEF > 70%), and 27 patients (7%) had preserved CI but altered LVEF (lactate—3.49 ± 2.95 mmol l) due to a marked tachycardia. None of the front-line hemodynamic parameters was discriminatory to identify the circulatory profile identified by TEE assessment (Table).

**Table:** 

	EF < 40%	EF ≥ 40%
CI ≥ 3 l/min/m²		
n (%)	27 (7%)	146 (38%)
mBP (mmHg)/HR (bpm)	82 ± 15/119 ± 26	76 ± 16/114 ± 20
CVP (mmHg)/ScVO2 (%)	10 ± 4/76 ± 13	10 ± 4/80 ± 10
CI < 3 l/min/m²		
n (%)	74 (19%)	141 (36%)
mBP (mmHg)/HR (bpm)	78 ± 16/108 ± 26	77 ± 17/96 ± 23
CVP (mmHg)/ScVO2 (%)	11 ± 5/76 ± 11	10 ± 5/76 ± 11

**Conclusion:** Front-line hemodynamic and metabolic parameters fail to discriminate between the distinct hemodynamic profiles identified during TEE assessment of ventilated patients with septic shock.

### P-160 The effects of fluid administration on arterial load in critically ill patients

#### Jozwiak Mathieu^1^, Millasseau Sandrine^1^, Richard Christian^1^, Monnet Xavier^1^, Mercado Pablo^1^, Depret François^1^, Alphonsine Jean-Emmanuel^1^, Teboul Jean-Louis^1^, Chemla Denis^1^

##### ^1^CHU Bicêtre, Le Kremlin-Bicêtre, France

###### **Correspondence:** Jozwiak Mathieu - mathieu.jozwiak@aphp.fr

*Annals of Intensive Care* 2018, **8(Suppl 1):**P-160

**Introduction:** Aortic end-systolic pressure (ESP) is considered as a reliable index of left ventricular afterload. Recently, the effective arterial elastance (Ea), i.e., the ratio of ESP over stroke volume (SV), has also been proposed as a reliable afterload index. Our aim was to document peripheral estimates of Ea (EapSAP) at the bedside in critically ill patients, and to investigate the haemodynamic mechanisms responsible for Ea changes after fluid administration (FA).

**Patients and methods:** In the validation study, carotid tonometry (Complior) was prospectively performed on 50 haemodynamically stable spontaneously breathing patients equipped with an arterial femoral (n = 21) or radial (n = 29) catheter. Ea was defined as the (0.9 × cSAP) SV ratio, where cSAP was the central systolic arterial pressure directly measured from the calibrated carotid waveform. EapSAP was calculated as the (0.9 x peripheral systolic arterial pressure) SV ratio. SV was obtained by transpulmonary thermodilution or transthoracic echocardiography. In the clinical study, we included 30 patients with invasive haemodynamic monitoring (PiCCO-2), in whom FA was planned.

**Results:** In the validation study, the Complior allowed estimating Ea in all patients (Ea = 1.73 ± 0.62 mmHg mL). The (EapSAP—Ea) bias was smaller at the femoral than radial artery level (0.08 ± 0.08 vs. 0.18 ± 0.16 mmHg mL, p < 0.05) and was strongly related to the systolic pressure amplification between the carotid and peripheral artery (r = 0.89, p < 0.05). Ea was more strongly related to SV (r = − 0.80) than to ESP (r = 0.48) (each p < 0.05). The four-quadrant plot analysis indicated that 47 patients (94%) exhibited a concordant low Ea high SV pattern or high Ea low SV pattern, while only 33 patients (66%) exhibited concordant high Ea high ESP pattern or low Ea low ESP pattern (p < 0.05). There was a negative relationship between changes in EapSAP and changes in SV in the whole population, in fluid responders (cardiac index increases > 15% after FA), in pressure responders (mean arterial pressure increases > 10% after FA) and in non-responders, while no consistent relationship between EapSAP and ESP changes was documented.

**Conclusion:** Ea may be reliably estimated at bedside by using the (0.9 x femoralSAP) SV ratio. Ea value and Ea changes induced by FA were related to SV rather than to ESP. Thus, Ea should be considered as an index reflecting SV rather than left ventricular afterload in critically ill patients.

### P-161 Pulmonary ischemia–reperfusion in a rat model: impact of cardiopulmonary bypass on pulmonary endothelial dysfunction and systemic inflammation

#### Selim Jean^1^, Hamzaoui Mouad^1^, Boukhalfa Ines^1^, Djerada Zoubir^1^, Chevalier Laurence^1^, Gentil Damien^1^, Besnier Emmanuel^1^, Dumesnil Anais^1^, Renet Sylvanie^1^, Mulder Paul^1^, Doguet Fabien^1^, Richard Vincent^1^, Veber Benoit^1^, Baste Jean-Marc^1^, Tamion Fabienne^1^

##### ^1^CHU de Rouen, France

###### **Correspondence:** Selim Jean - sjean85@msn.com

*Annals of Intensive Care* 2018, **8(Suppl 1):**P-161

**Introduction:** Lung transplant (LT) morbidity remains actualy high. Ischemia–reperfusion (IR) of the graft is one of the main causes of these early complications. The routine use of Cardiopulmonary Bypass (CPB) during LT is currently considered deleterious. The objective of this study is to evaluate the impact of CPB on pulmonary endothelial dysfunction and systemic inflammation after pulmonary IR in a rat model.

**Patients and methods:** This study included a Sham group (n = 11), a CPB group (n = 9), an IR group (n = 8) and a CPB-IR group (n = 11). Rats were exposed to 45 min of CEC, 30 min of left pulmonary ischemia and 15 min of reperfusion. Fonctional endothelial dysfunction was evaluated by measurement of the pulmonary artery reactivity. Systemic inflammation was evaluated by the plasma assay of IL-1 beta, IL-10 and TNF-alpha. The endothelial glycocalyx was evaluated by plasma assay syndecan-1 and electron microscopy. The statistics were performed using an ANOVA test, p < 0.05.

**Results:** We showed that CPB associated with IR induce an endothelial vasorelaxation dysfunction mainly mediated by nitric oxyde (NO). CPB increased significantly the effects of IR on systemic inflammation with increased plasma levels of IL-1 beta (CPB-IR 594.8 ± 113.0 vs. IR 109.9 ± 29, p < 0.05) and IL-10 (CPB-IR 234.8 ± 16.0 vs. IR 149.8 ± 15.4 pg mL, p < 0.01). The level of TNF-alpha increased significantly in all groups compared to the Sham group. CPB increased the effects of IR on glycocalyx degradation with syndecan-1 (CPB-IR 22.1 ± 0.7 ug mL vs. IR 18.3 ± 0.7 ug mL, p < 0.001).

**Conclusion:** CPB and pulmonary IR combination would increase pulmonary endothelial dysfunction and systemic inflammation. The glycocalyx degradation appear to be one important mechanism. The use of CPB routinely during LT may therefore be deleterious, further studies in humans need to be conducted to confirm these data.

### P-162 Veno-arterial PCO2 gradients can predict an increase in oxygen consumption following fluid challenge

#### Mongkolpun Wasineenart^1^, Gardette Mickaël^1^, Levy Sophie^1^, Dominguez-Curell Claudia^1^, Vincent Jean-Louis^1^, Jacques Creteur^1^

##### ^1^Hôpital Erasme, Bruxelles, Belgique

###### **Correspondence:** Mongkolpun Wasineenart - wasineenart.mongkolpun@yahoo.com

*Annals of Intensive Care* 2018, **8(Suppl 1):**P-162

**Introduction:** During circulatory shock, the goal of increasing cardiac output is to correct tissue hypoxia, which can be manifested by an increase in oxygen consumption (VO2) associated with an increase in oxygen delivery. We hypothesized that, in patients in circulatory shock, veno-arterial CO2 gradients (Pv-aCO2) could be a good predictor of an increase in VO2 in fluid responders.

**Patients and methods:** We included patients with circulatory shock who received a fluid challenge. Circulatory shock was defined by the association of vasopressor requirements to maintain mean arterial pressure (MAP) and a blood lactate concentration ≥ 2 mmol L. We measured cardiac index (CI) and arterial and central venous blood gases and arterial lactate before and after a volume expansion (500 ml of Plasmalyte^®^). Cardiac index (CI) was measured using a pulse contour analysis method (PiCCO + Pulsion, Munich, Germany). CI responders were the patients in whom CI increased (ΔCI) by > 15%. In those patients, VO2 responders were those in whom VO2 increased (ΔVO2) by > 15%. Receiver operating characteristic (ROC) curves were performed. The data was presented as median (25th percentile-75th percentile). A p < 0.05 was considered as statistically significant.

**Results:** We studied 66 patients in circulatory shock (42 of septic origin), including 29 (43%) responders. VO2 increased concomitantly 14 (48%) of these. Before volume expansion, CI and ScvO2 were similar in VO2 responders and non-responders (CI 2.0 (1.8–2.7) vs. 1.8 (1.2–2.3) (L min m^2^), p = 0.6, ScvO2 63 (56–66) vs. 56 (47–65), p = 0.1, respectively) but lactate and Pv-aCO2 were higher in VO2 responders than in VO2 non responders (lactate 3.5 (2.8–9.1) (mmol L) vs. 2.5 (2–5) (mmol L), p = 0.02, Pv-aCO2 9 (8–15) vs. 7 (6–8), p = 0.03, respectively). A Δ + VO2 > 15% was not predicted by baseline ScvO2 but by a baseline Pv-aCO2 ≥ 8 mm Hg (sensitivity 85%, specificity 72%, area under the ROC curve (AUC) = 0.75), by a high baseline lactate ≥ 3 mmol L (sensitivity 71% specificity 75%) and the combination of both (sensitivity 70%, specificity 81%, AUC = 0.8).

**Conclusion:** In circulatory shock, unlike ScvO2, Pv-a CO2 difference and blood lactate concentrations are good predictors of an increase in oxygen consumption in the fluid responders.

### P-163 Continuous photoplethysmographic heart rate variability monitoring as an indicator of outcome?

#### L’Her Erwan^1^, Bodenes Laetitia^1^, Librati Audrey^1^, Pateau Victoire^1^

##### ^1^CHU de la Cavale Blanche, Brest, France

###### **Correspondence:** L’Her Erwan - erwan.lher@chu-brest.fr

*Annals of Intensive Care* 2018, **8(Suppl 1):**P-163

**Introduction:** The autonomic nervous system (ANS) is highly adaptable and allows the organism to maintain its balance when experiencing stress. Heart rate variability (HRV) is a mean to evaluate cardiac effects of ANS activity and a relation between HRV and outcome has been proposed in various types of patients. While electrocardiographic HRV assessment seems to be the gold standard, we evaluated the feasibility of an automated HRV monitoring based on standard photoplethysmographic monitoring. This project is based on a prospective physiological tracing data-warehousing program (Rea STOC, clinicaltrials.gov # NCT02893462) that aims to record more than 1500 ICU patients over a 3-years period.

**Patients and methods:** Physiological tracings were recorded from the standard monitoring system (Intelliview MP70 Philips), using a dedicated network and extraction software (Synapse v1, LTSI INSERM U1099) that enables photoplethysmographic recordings from oximetry monitoring at a native resolution of 125 Hz. Raw data were subsequently stored on a dedicated local server, before anonymization and analysis. All consecutive patients were recorded for a 2-hours period during the 24-hours following ICU admission. All measurements were recorded with the patient laying supine, with a 30° bed head angulation. Physiological recordings were associated with metadata collection by a dedicated research assistant.HRV parameters defined in a previous study were derived using Kubios HRV premium (standard deviation of all normal RR intervals [SDNN], root-mean-square of difference of successive RR intervals [rMSSD], and RR triangular index) and frequency domain (power within low-frequency band [LF], and power within high-frequency band [HF]).

**Results:** 110 patients’ tracings were analyzed (72 male 38 female + age 61.1 ± 13.8 yr). Temporal analysis depicted a significant difference in terms of survival according to the triangular index (TI) value (Table 1). ROC curve analysis enabled to define a TI cut-off = 10.96 (Se 50% + Sp 74%). A better prognosis at the median Day-6 ICU stay (Alive + Discharge) tended to be associated to lower entropy indexes values.

**Table 1:** Outcomes

	Outcome	*p value*	Prognosis at Day 6	*p value*		
	*Survival* (n = 88)	*Died* (n = 22)		*Positive* (n = 53)	*Negative* (n = 57)	
*Temporal analysis*						
RMSSD (ms)			0.17			0.88
SDNN (ms)	89.67 ± 9.80	108.81 ± 22.56	0.39	96.11 ± 12.83	91.07 ± 12.80	0.78
TI	9.98 ± 0.98	19.77 ± 6.10	0.0076	10.02 ± 1.17	13.71 ± 2.63	0.21
*Frequency analysis*						
LF (ms^2^)	25655.4 ± 10244.03	5164.41 ± 1427.26	0.32	34086.26 ± 16795.48	9907.38 ± 2391.95	0.14
HF (ms^2^)	31030 ± 12369.23	4781.49 ± 1302.04	0.29	34242.26 ± 18924.49	17912.60 ± 7713.44	0.45
*Poincaré analysis*						
SD1 (ms)	75 ± 7.45	60.07 ± 10.04	0.34	71.36 ± 10.27	72.62 ± 7.06	0.92
SD2 (ms)	115.17 ± 13.21	77.95 ± 13.63	0.17	114.55 ± 19.02	101.38 ± 11.80	0.55
*Entropy analysis*						
Sample entropy	1.22 ± 0.05	1.32 ± 0.10	0.42	1.17 ± 0.07	1.32 ± 0.07	0.14
Approximate entropy	1.22 ± 0.03	1.32 ± 0.06	0.11	1.01 ± 0.03	1.1 ± 0.04	0.09

**Conclusion:** HRV monitoring using plethysmography is feasible and has the potential to detect physiological deterioration and predict patients’ outcome. The benefits of using plethysmography instead the ECG gold standard should be the more important versatility of the technique.

### P-164 Management of diabetic ketoacidosis in the intensive pediatric care service of the EHS canastel ORAN. Algeria

#### Zerhouni Amel^1^, Tabet Nabil^1^, Addou Zakaria^1^, Belahbiche K^1^, Moussati M^1^, Mir Souad^1^, Douah A^1^, Youbi H^1^, Aouffen Nabil^1^

##### ^1^Oran, Algeria

###### **Correspondence:** Zerhouni Amel - sebianezeramel@yahoo.com

*Annals of Intensive Care* 2018, **8(Suppl 1):**P-164

**Introduction:** Diabetic ketoacidosis is an acute complication of diabetes, defined as metabolic acidosis with a high anionic gap, associating hyperglycemia > 16 mmol l (3 g l), positive ketonuria, or superior or equal ketonuria to ++, it is a medical emergency which can occur in a known diabetic patient, or not. OBJECTIVE—To describe the clinical therapeutic and prognostic aspects of diabetic ketoacidosis in the intensive Pediatric care unit at the EHS Canastel Oran, Algeria.

**Patients and methods:** Retrospective study carried out over a period of 2 years. From January 1, 2015 to January 1, 2017, in the intensive pediatric care service. The data was entered and analyzed using Excel 2007.

**Results:** 16 cases were retained on 450 hospitalizations per year, 95% of cases had no history with diabetes, 5% occurred in known diabetics with insulin, but are not followed medically. Our patients were aged from 10 months to 15 years, but the average age of these patients was 6 years and 5 months, with a slight female predominance, coma was preceeded by 75% of cases polydipsy polyuria syndrome and 12% weight loss, triggered by an infectious syndrome including 31% of ENT cases, 12% of respiratory infections and 12% of cases with digestive infections characterized by fever, abdominal pain, vomiting. The delay between diagnosis and admission to ICE was 24–7 days. At admission 80% of patients were scored at 11 15 on the Glasgow scale, with presence of the cough reflex, and 20% were scored at < 7 15 requiring tracheal intubation and mechanical ventilation of 48 h with signs of dehydration and ionic disorders, namely hypokalemia and hyper-natremia, blood glucose at admission varies between 3.5 and 5 g l with glycosuria at +++ and ketonesuria between ++ and ++++ in only 18% of the patients had metabolic acidosis, a cerebral computed tomography (CT) performed in 13% of cases found a slight cerebral edema. Therapeutic management was the rehydration, correction of metabolic disorders and introduction of insulin into SAP, with monitoring and subcutaneous relaying due to ketonuria negativity. The outcome was favorable for all patients.

**Conclusion:** Diabetic ketoacidosis is a major complication of diabetes which can be avoided by a good prevention campaign and systematic screening of any child suspected of diabetes, recognition of risk situations such as infections and clinical manifestations in order not to delay the management.

### P-165 Management of viper bit in pediatric resuscitation at the oran childhood hospital in algeria

#### Tabet Aoul Nabil^1^, Addou Zakaria^1^, Douah Ali^1^, Youbi Houari^1^, Moussati Mohammed^1^, Belhabich Kamel^1^, Zerhouni Amel^1^, Abada Sanaa^1^, Mir Souad^1^, Aouffen Nabil^1^

##### ^1^EHS pédiatrique de Canastel, Oran, Algeria

###### **Correspondence:** Tabet Aoul Nabil - tabetrea@yahoo.fr

*Annals of Intensive Care* 2018, **8(Suppl 1):**P-165

**Introduction:** Envenomations by snake bite are infrequent in children in our region in western Algeria. The objective of our study is to describe the epidemiological, diagnostic, therapeutic and evolutionary characteristics of snake bites in children admitted to pediatric intensive care.

**Patients and methods:** It is a retrospective study on patient file carried out between January 2013 and August 2017 at the level of the versatile pediatric resuscitation of canastel, Oran in Algeria. The parameters analyzed were—age, sex, time of bite, time to take, duration of admission to resuscitation, clinical, therapeutic and severity of envenomation.

**Results:** Ten cases of snake bites were admitted during this period, boys accounted for 100% of the study population. The average age was 3.5 years [11 months-6.5 years]. All bites occurred during the day (40% in the morning and 60% in the afternoon). The average time to admission to intensive care was 10.2 h. The site of the bite was in the upper limb in 80% and the limb inferior in 20% of the cases. According to the classification of the envenomation—60% of the cases were of severity of grade 2–3 and 40% of grade 0–1. All grade 2–3 envenomations had received antivenom serotherapy and antibiotic therapy, and only one case had an aponeurotomy. Changes were favorable in all children with an average hospital stay of 5 days.

**Conclusion:** Snakebite is a therapeutic emergency. Initial clinical evaluation and early administration of antivenom serum in severe forms had led to a favorable outcome for child victims of envenomation.

### P-166 The scorpion envenomation at the intensive care pediatric service of Canastel, Oran, Algeria

#### Zerhouni Amel^1^, Tabet Nabil^1^, Addou Zakaria^1^, Belahbiche K^1^, Moussati M^1^, Mir S^1^, Douah A^1^, Youbi H^1^, Aouffen Nabil^1^

##### ^1^Oran, Algeria

###### **Correspondence:** Zerhouni Amel - sebianezeramel@yahoo.com

*Annals of Intensive Care* 2018, **8(Suppl 1):**P-166

**Introduction:** Scorpion sting is a public health problem world wide with a global distribution of 1500 species. In Algeria, scorpionic envenomation occupies a prominent place in declarations. In 2016, 43000 cases were reported. The objective of our study is to describe the epidemiological, diagnostic, therapeutic and evolutionary characteristics of the scorpion sting in children.

**Patients and methods:** Retrospective study of 07 cases of scorpionic envenomation hospitalized in the pediatric resuscitation department of the EHS Canastel ORAN conducted during the year 2016The inclusion criteria were the presence of traces with at least one locoregional or general clinical signs. The parameters studied—age, sex, city of origin, time of bite, time of management, initial first aid, time limit for admission to pediatric intensive care, and severity criteria.

**Results:** 57% of these cases were boys and 43% girls. The mediane age 6.5 90% of the punctures occurred during the day, the site of the injection was the lower limb in 80% of the cases and 20 There were 9 bites scorpion cases in the west of Algeria and exactly in Oran and Tiaret.100 of the cases was the upper limb. The delay of the management was from 1 to 3 h for 30 100 of the cases who were classified in the third classed according to the clinical signs of gravity. The type of the scorpion was not identified. We can classify all the patients that we received in our service into three classes −43% in class I, with local signs such as pruritus, redness, abnormalities and local pain. EVA 6–7, calmed by the infusion of 15 mg kg iv of paracetamol and application of xylocaine cream at the site of the sting. They were left under surveillance for 8 h. In class II, we had 30 100 of the cases with neurovegetative signs such as tachycardia at 120b min, diarrhea, hyperthermia at 39 C in addition to local signs that they were treated symptomatically and left under surveillance for 24 h. In Class III, 27 100 of the cases with cardiogenic shock, acute respiratory distress and agitation requiring sedation, tracheal intubation and 48-hour mechanical ventilation. Benefiting by 72 h of hospitalization. The evolution during hospitalization for all our patients was favorable.

**Conclusion:** Scorpionic envenomation is a therapeutic emergency that requires rapid management to avoid complicationsThe severity of the scorpionic envenomation rests mainly on the type of scorpion and the dey of the management.

### P-167 Psychological impact on children following their hospitalization in pediatric intensive care, Oran, Algeria

#### Tabet Aoul Nabil^1^, Douah Ali^1^, Addou Zakaria^1^, Youbi Houari^1^, Zerhouni Amel^1^, Belhabich Kamel^1^, Moussati Mohammed^1^, Mir Souad^1^, Abada Sanaa^1^, Aouffen Nabil^1^

##### ^1^EHS pédiatrique de Canastel, Oran, Algeria

###### **Correspondence:** Tabet Aoul Nabil - tabetrea@yahoo.fr

*Annals of Intensive Care* 2018, **8(Suppl 1):**P-167

**Introduction:** The residence of children in intensive care is most often due to the existence of one or more organ dysfunction which requires heavy treatment (intubation, ventilation, drainage, venous tract) and this in a hostile environment which amplifies the aggression organic. The main objective of our work is to study the consequences of hospitalization of children in pediatric resuscitation.

**Patients and methods:** This is a descriptive prospective study on the outpatient consultation file of Canastel’s EHS Multipurpose Resuscitation. We studied 18 files and assessed memory, perception of contact and nuisance factors felt by sick children.

**Results:** Out of 18 children seen in post resuscitation. The sex ratio is 1.25. The average age of children is 4 years (2 months–14 years). The average hospital stay is 13 days. The average GOS (Glasgow out scale) is 4. (2–5). The average duration of ventilation is 8 days. 60% of children had central vascular access. Three children describe a total memory of the stay, 2 some memory and 2 none. Three children have a good perception about the staff, one child dissatisfied and three others indifferent. The nuisance factors described by the children are pain (3), cold (2), noise (3), hunger (1) and light (1).

**Conclusion:** Consequences of psychological trauma, insufficiently evaluated especially by the staff, which result in the appearance of psychological disorders (nightmares and anxiety) with sometimes even severe post-traumatic neurosis. Hence the need to adapt the environment and mainly noise and respect for sleep.

### P-168 Cefotaxime dosing regimen optimization in critically ill children

#### Béranger Agathe^1^, Oualha Mehdi^2^, Urien Saïk^2^, Genuini Mathieu^2^, Renolleau Sylvain^2^, Aboura Radia^2^, Hirt Déborah^2^, Heilbronner Claire^2^, Tréluyer Jean-Marc^2^, Benaboud Sihem^2^

##### ^1^CHU de Montrouge, France; ^2^Hôpital Tarnier-Cochin-Necker, Université Paris Descartes, Paris, France

###### **Correspondence:** Béranger Agathe - agathe.beranger@gmail.com

*Annals of Intensive Care* 2018, **8(Suppl 1):**P-168

**Introduction:** Pharmacokinetic parameters are modified in critically ill children. This could result in a low CTX exposure, with subtherapeutic CTX concentrations. Our objectives were to build a pediatric population pharmacokinetic model for cefotaxime (CTX) and its metabolite desacetylcefotaxime (D-CTX), in order to optimize individual dosing regimen.

**Patients and methods:** All children aged less than 18 years, weighing more than 2.5 kg, and receiving intermittent CTX infusions were included. CTX and D-CTX were quantified by high performance liquid chromatography. Pharmacokinetics were described using the non-linear mixed effect modelling software MONOLIX. Monte Carlo simulations were used to optimize dosing regimen, in order to maintain serum concentration above the target concentration (defined at 2 mg L^−1^) 100% of the time during the dosing interval.

**Results:** We included 49 children with a median (range) post natal age of 23.7 (0.2–229) months, and median body weight (range) of 10.9 (2.5–68) kg. A one compartment model with first-order elimination adequately described the data. Median (range) values for CTX clearance, D-CTX clearance and volume of distribution were respectively 0.97 (0.3–7.1) L h^−1^, 3.2 (0.6–16.3) L h^−1^, and 3.3 (0.85–20) L. Body weight and post natal age had significant impact on pharmacokinetic. CTX calculated residual concentrations were low, and no patient succeeds to attain the target, leading to a low CTX exposure. Unlike with intermittent administration, continuous infusion provided a probability of target attainment of 100%, regardless of age and weight.
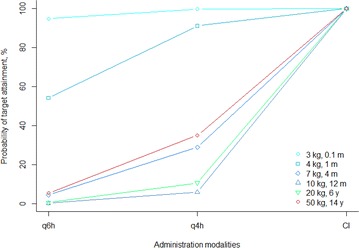


**Conclusion:** Standard intermittent CTX dosing regimens in critically ill children are not adequate to reach the target, and leads to subtherapeutic CTX concentrations. Population pharmacokinetic modelling and developmental approach enabled to perform simulations, which showed that continuous infusion was the most adapted dosing regimen, for children over 1 month of age.

### P-169 Norepinephrine in pediatric septic shock

#### Denante Solène^1^, Michel Fabrice^1^

##### ^1^Hôpital de la Timone, Marseille, France

###### **Correspondence:** Denante Solène - solene.denante@gmail.com

*Annals of Intensive Care* 2018, **8(Suppl 1):**P-169

**Introduction:** Use of norepinephrine in pediatric septic shock is still debated. Furthermore, high doses can be associated with serious side effects. Few data are available in pediatric population. The main objective of our study was to determine if there was a dose of norepinephrine beyond which its hemodynamic effects are maximum (refractory dose) and or harmful.

**Patients and methods:** We led a retrospective, observational study in a pediatric intensive care unit and a a pediatric and neonatal intensive care unit. All children diagnosed for septic shock treated with norepinephrine between January 2006 and December 2015 were included. For each patient, were collected—demographic data, organ dysfunctions, site of infection, fluid resuscitation, delay of adapted antibiotherapy, associated treatments, others vasoactive supports. Concerning norepinephrine, we collected the initial dose, the maximal dose, the first-line use, the delay of use, the total duration of use and the duration of maximal dose. Hemodynamical and respiratory parameters and associated treatments were collected at the introduction and at the maximal dose of norepinephrine.

**Results:** One hundred and ninety one patients were included, including 44.5% of preterm neonates. Norepinephrine was used as first-line treatment in 144 patients (75.4%). The global mortality rate was 28.8%, 35.3% in preterm neonates. We could not determine a refractory dose. Mortality increased to 46.2% when norepinephrine dose reached 0.75 µg kg per minute. The maximal dose of norepipnehrine was independently associated with mortality (OR 1.4, [95% confidence interval 1.1–2.0], p = 0.02). Others independent factors of mortality were—the neurologic dysfunction (odds ratio 2.7, [95% confidence interval 3.5–4.3], p = 0.03), the respiratory dysfunction (OR 4.4, [95% confidence interval 1.1–6.8], p = 0.03), the acid lactic serum at the introduction of norepinephrine (OR 1.2, [95% confidence interval 1.0–1.3], p = 0.04) and the pH at the introduction of norepinephrine (OR 3.7 [95% confidence interval 1.4–6.6], p < 0.001).
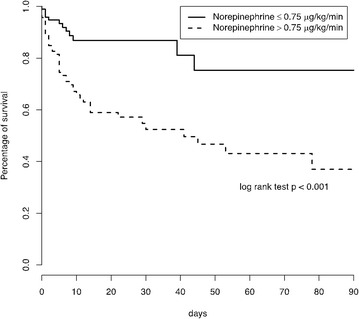


**Conclusion:** Mortality rates were in accordance with previous pediatric septic shock studies, suggesting that norepinephrine is an efficient treatment for pediatric septic shock. However, high doses of norepinephrine could have probably harmful effects.

### P-170 Population pharmacokinetics of enoxaparin in pediatric liver transplantation patients

#### Oualha Mehdi^1^, Chardot Christophe^1^, Debray Dominique^1^, Lesage Fabrice^1^, Harroche Annie^1^, Renolleau Sylvain^1^, Tréluyer Jean-Marc^2^, Urien Saïk^2^

##### ^1^Hôpital Necker Enfants-Malades, Université Paris Descartes, Paris, France; ^2^Université Paris Descartes, Paris, France

###### **Correspondence:** Oualha Mehdi - mehdi.oualha@aphp.fr

*Annals of Intensive Care* 2018, **8(Suppl 1):**P-170

**Introduction:** Preventing post liver transplantation (LT) hepatic artery and portal vein thrombosis is challenging and includes enoxaparin administration. Enoxaparin pharmacokinetics (PK) has not been investigated in children following LT. Between-subject variability and critical illness may alter PK, leading to the risk of subtherapeutic exposure.

**Patients and methods:** Clinical, biological and kinetic data were retrospectively collected in a single Pediatric Intensive Care Unit center from January 2013 to July 2015. We described an enoxaparin PK model in 22 children the first week following the LT. Anti-Xa activity time-courses were analyzed using a non linear mixed effects approach with Monolix version 2016R.

**Results:** Anti-Xa activity time-courses were well described by a one-compartment open model with first order absorption and elimination. Body weight prior the surgery (BWPREOP) and the related postoperative variation (BW(t)) were the main covariates explaining CL and V between subject variabilities. Parameter estimates were CLi = CLTYP*(BWPREOP 70)3 4 + Vi = VTYP*(BW(t) 70)1 + where typical clearance (CLTYP) and typical volume of distribution (VTYP) were 1.26 L h^−1^ and 16.9 L, respectively. Standard dosing regimens of 50 IU kg 12 h were insufficient to reach the target range of anti-Xa activity of 0.2 to 0.4 IU mL. Specifically, 7 children (32%) did never attain the target range during the whole period of treatment and all children were at least once under dosed. According to the final results, we simulated individualized dosing regimens within 4 h following the first administration. More than 100 IU kg 12 h are suggested to reach the target range of anti-Xa activity of 0.2 to 0.4 IU mL from the first day.
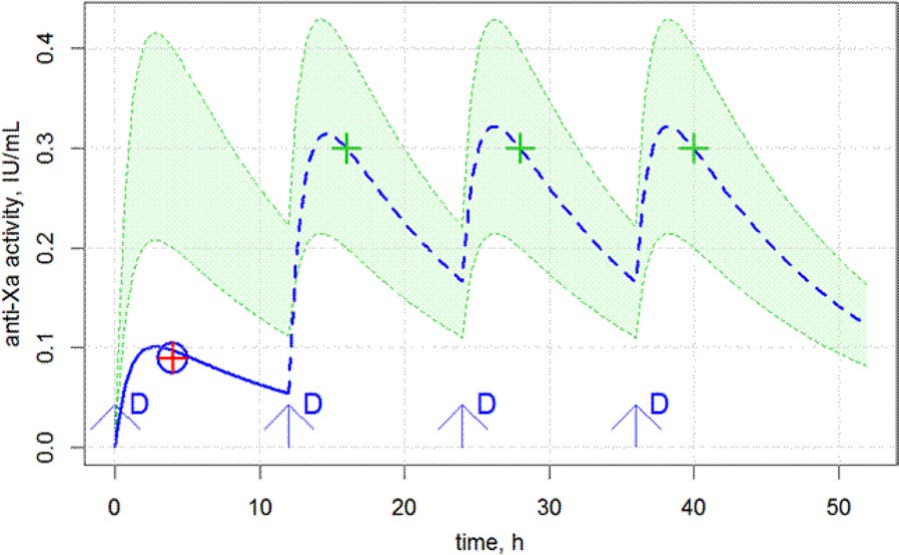


**Conclusion:** Standard enoxaparin dosing regimens is not appropriate to reach the target in pediatric liver transplantation patients. Enoxaparin PK modeling should help the physician to achieve the target range from the initial dose and during the maintenance doses. Higher dosing regimens, especially in youngest children are suggested to achieve the prophylactic target range.

### P-171 Pharmacokinetic analysis of unfractionated heparin in critically ill children during extracorporeal membrane oxygenation: Do we achieve the target?

#### Grimaud Marion^1^, Urien Saïk^2^, Borgel Delphine^1^, Amar Géraldine^1^, Gaudin Régis^1^, Burgos Paula^1^, Renolleau Sylvain^1^, Tréluyer Jean-Marc^2^, Oualha Mehdi^1^

##### ^1^Hôpital Necker Enfants-Malades, Université Paris Descartes, Paris, France; ^2^Université Paris Descartes, Paris, France

###### **Correspondence:** Grimaud Marion - marion.grimaud@aphp.fr

*Annals of Intensive Care* 2018, **8(Suppl 1):**P-171

**Introduction:** Preventing thrombosis in children under extracorporeal membrane oxygenation (ECMO) requiring unfractionated heparin administration. Unfractionated heparin pharmacokinetics (PK) has not been well investigated in children under ECMO. We described the unfractionated heparin dosing regimens and resulting anti-Xa activities in children with ECMO.

**Patients and methods:** This is a single center retrospective study from March 2015 to September 2016. Were included children (< 18 years old age) who were under ECMO for refractory hemodynamic failure related to (i) myocarditis or (ii) septic shock. Anti-Xa activity time-courses were analyzed using a non linear mixed effects approach with Monolix version 2016R.

**Results:** A total of 12 children were included (septic shock, n = 6 + myocarditis + n = 6 with a median age of 42 months (0–197), a median weight of 17.7 kg (4.9–69) and median admission PELOD-2 score of 11 (6–21). Bleeding occurred in 2 children and thrombosis in 4. An initial bolus of unfractionated heparin ranging from 30 to 85 IU kg was infused and then continued by continuous perfusion with an initial dosing ranging from 15 IU kg h to 215 IU kg h. A total of 214 Anti-Xa activity measurements were performed between 1 h to 189 h after the start of the treatment. Dose changes occurred for all children with total of 59 dose changes. Anti-Xa activity was in the target (0.4 to 0.6 IU mL) in 96 samples (45%), < 0.4 IU mL in 81 samples (38%) and > 0.6 IU mL in 37 samples (17%). All children observed at least one time an anti-Xa activity out of the target (0.4 to 0.6 IU mL). Anti-Xa activity time-courses were well described by a one-compartment open model with first order elimination. Body weight (BW) was the main covariate explaining the between subject variability of clearance (CL) and volume of distribution (V. Parameter estimates were CLi = CLTYP*(BW 70)3 4 + Vi = VTYP*(BW 70)1 + where typical clearance (CLTYP) and typical volume of distribution (VTYP) were 2.42 L h^−1^ and 5.1 L, respectively.
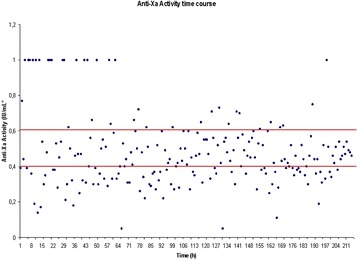


**Conclusion:** In children with ECMO, unfractionated heparin standard dosing regimen and empirical adjustments are not appropriate to reach and maintain the target of anti-Xa activity. Unfractionated heparin PK modeling may help the physician to achieve the target range from the initial dose and during the maintenance doses.

### P-172 Hemophagocytic syndrome and infection: about 12 cases

#### Bouzid Salma^1^, Daoud Fatma^1^, Aydi Zohra^1^, Hajjam Najah^1^, Rachdi Imene^1^, Zoubeidi Hana^1^, Ben Dhaou Basma^1^, Boussema Fatma^1^

##### ^1^Hôpital Habib Thameur, Tunis, Tunisia

###### **Correspondence:** Bouzid Salma - salmabz87@gmail.com

*Annals of Intensive Care* 2018, **8(Suppl 1):**P-172

**Introduction:** Hemophagocytosic syndrome is a rare but potentially fatal disease, defined by clinical, biological and histological criteria. It raises various affections, posing the problem of etiologic diagnosis. In this work we will focus on hemophagocytosic syndromes exclusively of infectious origin.

**Patients and methods:** This is a retrospective study involving 12 patients with an infectious induced hemophagocytic syndrome, hospitalized in the internal medicine department of Habib Thameur Hospital of Tunis during the period from January 2014 to September 2017. The objective of this work is to determine their clinico-biological, etiological and evolutionary aspects.

**Results:** Among the twelve cases, there were as many men as women. Mean age was 47.2 years. An immunosuppression factor was found in 41.7% cases. Clinically, all patients had fever. A deterioration of the general condition was observed in 9 cases. Tumor syndrome was found in 50% of patients. Biologically, cytopenia was noted in 11 cases—bicytopenia in 7 cases and pancytopenia in 4 cases. Hepatic cytolysis was observed in 10 patients, hyperferritinemia in 11 cases and hypertriglyceridemia in 11 cases. Cytological examination of bone marrow confirmed hemophagocytosis in 9 cases (3 patients had refused sternal puncture). The causal infection was of bacterial origin in 58% of cases—three cases of tuberculosis, one case of brucellosis, one case of rickettsial disease, one gram-negative bacilli septicemia and one staphylococcal septicemia. The viral origin was found in 5 patients—2 cases of EBV infection, one case of CMV infection, one HSV infection in one case and one case of HIV infection associated with candidiasis. A specific etiological treatment was initiated in all patients, with a favorable outcome in 83% of cases. However, we deplore two deaths. They were immunocompromised patients.

**Conclusion:** The infectious etiologies of hemophagocytic syndrome are numerous. The severity of this pathology requires a rigorous etiological investigation in order to determine the responsible infectious agent and propose a specific treatment of the causal infection, the only guarantee of a favorable evolution.

### P-173 Plasma exchange in the intensive care unit: a 14 year retrospective audit

#### Daubin Delphine^1^, Kanouni Tarik^1^, Platon Laura^1^, Buzancais Aurele^1^, Besnard Noemie^1^, Brunot Vincent^1^, Latry Pascal^1^, Jung Boris^1^, Klouche Kada^1^

##### ^1^CHU Lapeyronie, Montpeller, France

###### **Correspondence:** Daubin Delphine - d-daubin@chu-montpellier.fr

*Annals of Intensive Care* 2018, **8(Suppl 1):**P-173

**Introduction:** Plasma exchange (PE) may be indicated in the critical care setting as primary or adjunctive therapy for hematologic, neurologic, renal, and autoimmune disorders. However, little is know about its indication prescription and adverse effects. We therefore reviewed the current use of PE in our ICU.

**Patients and methods:** All patients aged more than 18 y.o who were admitted to our tertiary 20 bed ICU and received PE therapy between 2002 and 2016 entered the study. Data was collected from identified patient medical records.

**Results:** Seventy-eight patients with median age at 50.5 [39–64] y.o, sex-ratio 1.1 underwent 551 PE procedures. At ICU admission, median SAPS II was at 36 [27–46] and SOFA score at 6 [4–9]. More than half of patients (56%) were in mechanical ventilation, 46% received constrictive drugs and 40% were in renal replacement therapy. Indications of PE were—Thrombotic Thrombocytopenic Purpura (TPP) 43 (55%), Neurologic diseases (acute polynevritis 7, Myasthenia Gravis 4, ADEM 4, others 2) 17 (22%), Autoimmunes diseases (vasculitis 5, cryoglobulinemia 4, dermato-polymyositis 2, others 4) 15 (19%) and 3 patients for other indications. The median number of treatments patient was 5 [3–9]. The median treatment duration time was at 120(87–180) minutes. Femoral vein was used as vascular access (93%) and most of PE procedures (78%) were performed with citrate anticoagulation. Median exchange volume was at 3500 [2796–4190] ml and renal replacement fluid was fresh frozen plasma (FFP) in 64%, 50% FFP and 50% human albumin 4% in 22% and human albumin only in 14% of procedures. Adverse effects were observed in less than 20% of procedures and 10% were lifethreathening including cardiac arrest, heart rhythm disorders, cerebral oedema and hemolysis. Other remaining complications were secondary infections 48%, hemorrhage 23%, and pulmonary oedema 14% in all patients. Twenty patients deceaded (ICU mortality 26%). ICU and hospital lenght of stay was at 19 ± 21 and 47 ± 33 days respectively. 2 3 of survived patients still underwent PE after their ICU discharge + 9 totally recovered whereas 49 (63%) were on partial remission.

**Conclusion:** PE is a routine and feasible technique in ICU. This study showed that TPP was the most commonly indication of its use and that outcome was fair. Adverse effects frequently occured but most of them were not severe. Further studies would benefit form larger cohort to improve indications, delay of initiation and practice of this treatment.

### P-174 Outcome of acute myeloid leukemia admitted to ICU according to cytogenetic characteristics

#### Bredin Swann^1^

##### ^1^Assistance publique, Hôpitaux de Paris, Paris, France

###### **Correspondence:** Bredin Swann - swann18b@gmail.com

*Annals of Intensive Care* 2018, **8(Suppl 1):**P-174

**Introduction:** Karyotype analysis is recognized as the main determinant of relapse risk and long term mortality in AML patients. However it is not known if cytogenetic characteristics are associated with life threatening inaugural complications and or ICU outcomes.

**Patients and methods:** TRIAL-OH is a prospective, multicenter observational study that included 1011 patients with hematological malignancies who required ICU admission in 2010–2011 in 17 French and Belgian centers (1). Newly diagnosed AML patients of this cohort were included in the present study. Karyotype analysis of all patients were collected and divided into 4 groups according to the MRC classification (favorable + intermediate and adverse). The main endpoint was hospital mortality. Using uni and multivariate analysis, we analysed the influence of karyotype group on outcomes.

**Results:** 144 patients were included. The karyotype was t(15 + 17) in 24 (17%) patients, inv(16) or t(8 + 21) in 13 (9%) patients, intermediate in 77 (54%) and adverse in 30 + 21%. 57 (39%) were male, the median age was 58 (45–68) years. AML was diagnosed during ICU stay or the day before hospital admission for all patients. Median time between hospital admission and ICU admission was 5 (1–18) days. Reason for ICU admission was acute respiratory failure (91 + 63%), shock (51 + 35%), acute kidney failure (37 + 25%). During ICU stay respiratory complications were related to infiltration by AML (29 + 20%), leucostasis (20 + 14%), hemoptysis (12 + 8%). 23 (15%) patients had tumor lysis syndrome. The median SOFA score at admission was 5 (4–9).45 (31%) patients died during ICU stay and 59 (41%) patients died during hospital stay. Multivariate analysis revealed that factors associated with hospital mortality were performans status before ICU admission (OR 1.44 (1–2.11)), Charlson co-morbidity score (OR 1.19 (0.98–1.45) and SOFA score at admission (1.18 (1.08–1.32). Karyotype group were not associated with hospital mortality in this cohort with OR 1.55 (0.26–8.34) for t(15 + 17) or inv(16) or t(8 + 21), OR 1.60 (0.56–5.06) for intermediate and 1.94 (0.56–7.12) for adverse group.

**Conclusion:** For newly diagnosed AML admitted to the ICU, cytogenetic characteristics doesn’t seems to influence the pattern of inaugural complications or hospital mortality. In this cohort, performans status, Charlson index and severity of acute disease were the main determinants of hospital mortality.

### P-175 The clinical picture of thrombotic microangiopathy in patients older than 60yo

#### Schmidt Julien^1^, Zafrani Lara^1^, Ardisson Fanny^1^, Ekpe Kenneth^1^, Ghrenassia Etienne^1^, Kerhuel Lionel^1^, Lemiale Virginie^1^, Stepanian Alain^1^, Azoulay Elie^1^, Mariotte Eric^1^

##### ^1^Hôpital Saint Louis, Paris, France

###### **Correspondence:** Schmidt Julien - j.schmidt@hotmail.fr

*Annals of Intensive Care* 2018, **8(Suppl 1):**P-175

**Introduction:** Thrombotic microangiopathy (TMA) syndrome encompasses a wide spectrum of pathologies characterized by the formation of thrombi in microcirculation, including thrombotic thrombocytopenic purpura (TTP), hemolytic and uremic syndrome (HUS), complement-mediated TMA (aHUS), and secondary TMAs. Ageing along with increasing awareness from clinicians have led to more frequent TMA-diagnosis among elderly. This study focus on their characteristics and outcomes.

**Patients and methods:** All TMA patients admitted to our ICU from 2006 to 2017 were enrolled. Clinical, biological and follow-up data were collected. Data are presented as numbers (%) and medians [IQR]. Elderly (≥ 60yo) were compared to non-elderly using non-parametric statistical tests.

**Results:** 154 TMA-patients aged 45yo [32–56] required ICU, including 26 (17%) elderly and 128 (83%) non-elderly. Elderly presented more frequently with a history of hypertension, diabetes mellitus, dyslipidemia, arteriopathy, tobacco use, cancer (respectively 65 vs. 26, p < 0.01 + 23 vs. 5%, p = 0.01 + 42 vs. 12%, p < 0.01 + 31 vs. 3%, p < 0.01 + 58 vs. 31%, p = 0.01 + 31 vs. 12.5%, p = 0.03). Baseline hemoglobin was 8 [6–9] g dl, LDH 1889 [1196–2713] U l, platelets count 17 [10–41] G l and SOFA 6 [5–8]. Organ involvement at admission included neurological (75%), renal (70%), cardiac (66%), digestive (42%). At admission, delirium was found in 27% of elderly versus 11% (p = 0.03), altered vigilance in 46 versus 21% (p = 0.007), stroke in 8 versus 1% (p = 0.02), digestive hemorrhage in 19 versus 5% (p = 0.02). Renal replacement therapy was required in 46% of elderly versus 27% (p = 0.047). Frequency of TTP, HUS and aHUS was similar beetween groups. TMA was more frequently associated with ongoing cancer and drug use in elderly (39 vs. 8%, p < 0.01 and 23 vs. 6%, p < 0.01, respectively). Gastro-intestinal bleeding during ICU occurred more often among elderly (35 vs. 15% (p = 0.02)). ICU mortality rate was higher (19 vs. 7%, p = 0.049). No difference was found concerning plasma-exchange therapy, steroids use, and rescue treatments for refractory-TTP.

**Discussion:** Increased complication and mortality rates in the elderly group might be ascribed to more cardiovascular morbidity in this population. The association between TMA and ongoing cancer suggests a routine oncological workup among elderly.

**Conclusion:** This study reports the clinical picture of TMAs in patients over-60-crowd. Increasing awareness of TMA-related complications may improve outcome.

### P-176 Antibiotics management of septic neutropenic patients in intensive care unit: How new French guidelines change clinical practice?

#### Jean-Michel Vanessa^1^, Tonnelier Jean-Marie^1^, Couturaud Francis^1^, L’Her Erwan^1^

##### ^1^CHRU La Cavale Blanche Hospital, Occidental Bretagne University, Brest, France

###### **Correspondence:** Jean-Michel Vanessa - vanessa.jean-michel@chu-brest.fr

*Annals of Intensive Care* 2018, **8(Suppl 1):**P-176

**Introduction:** Neutropenia, defined by an absolute count of polymorphonuclear neutrophils less than < 1500 mm3, exposes patients to infectious complications that can lead to sepsis or septic shock. The mortality risk is higher. The French guidelines published in 2016 were formulated to homogenize the clinical practices and improve survival.

**Patients and methods:** We performed a monocentric retrospective study including all consecutive patients admitted to the medical ICU of a tertiary hospital to a neutropenia with sepsis or septic shock, between the 30th of December 2012 and the 30th of December 2016. The study protocol was approved by the local Ethics Committee (2017.CE15) and published on clinical trial (NCT03217721).

**Results:** 2235 patients were admitted in ICU during this period. 130 patients (17%) presented a neutropenia with sepsis or septic shock. Among these patients, 92% had hematologic malignancies and 5% had solid tumour. 78 patients (60%) was treated empirically with antipseudomonal beta-lactam or carbapenem and aminoglycoside. 129 (99%) skin or suspected catheter-related infections were treated with anti-MRSA (methicillin-resistant staphylococcus aureus), vancomycin or linezolid. Adequate antibiotics as described in guidelines was performed to 76 patients (58%). 76 patients (58%) received aminoglycoside (58 patients received initial dose in ICU, 18 patients complement dose) and 100 (77%) received anti-MRSA with antipseudomonal beta-lactam or cabapenem. 71 patients (55%) had microbiologically documented infections with, 59% of bacteria (16% of gram-posit cocci, 1% of gram-negative cocci, 39% of gram-negative bacillus), 8% of fungi and 3% of viral infection (Table 3). Among of them, 10% (13) of ESBL, 2% (2) of MRSA and 2% (2) of emerging highly resistant bacteria (BHRe). The ICU-mortality rate was 29% (37 130) with 28% of 28-day mortality (36 130). The curves of the cumulative incidence of death risk between D0 and D28 were no different according to adequate empirical antibiotic treatment as like French guidelines (Fig 1). By multivariate analysis, independent factors of adequate antibiotic treatment were septic shock (OR, 0.38 + 95% CI 0.15–0.96) and febrile neutropenia > 7 days (OR, 0.42 + 95% CI 0.20–0.88) at ICU admission.

**Fig. 1:**
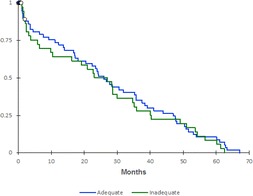
Kaplan–Meier survival between admission and 28-day according to adequate empirical antibiotic therapy guidelines (log rank, p = 0.53)

**Conclusion:** According to the usual clinical practice, septic neutropenic patients was already treated empirically by bitherapy including antipseudomonal or anti-MRSA if there is a skin or suspected catheter-related infection. Adjunction of aminoglycoside in empirically antibiotics for these patients with severe infection may be recommended.

### P-177 Outcome of allogeneic stem cell transplant recipients admitted to ICU: results of a wide admission policy

#### Gournay Viviane^1^, Dumas Guillaume^1^, Joffre Jérémie^1^, Baudel Jean-Luc^1^, Bourcier Simon^1^, Pichereau Claire^1^, Ait-Oufella Hafid^1^, Guidet Bertrand^1^, Maury Eric^1^, Bigé Naïke^1^

##### ^1^Hôpital Saint-Antoine, Paris, France

###### **Correspondence:** Gournay Viviane - vivianegournay@hotmail.fr

*Annals of Intensive Care* 2018, **8(Suppl 1):**P-177

**Introduction:** Prognosis of allogeneic hematopoietic stem cell transplant (HSCT) recipients admitted to ICU has improved with advances in HSCT procedures and critical care management, but also with evolution in ICU triage policy. Our aim was to describe the outcome of HSCT recipients admitted to ICU according to a wide admission policy.

**Patients and methods:** Retrospective multicenter study including all consecutive allogeneic hematopoietic stem cell transplant (HSCT) recipients admitted to Saint-Antoine hospital medical ICU, Paris, France from to January 2005 to April 2017. Admissions were identified through a systematic review of ICU database using ICD—10 codes Z948 and T860. Data were extracted from medical charts. Qualitative and quantitative values are expressed as number and percentage, and median and interquartile range, respectively. Comparisons between groups were performed using Fisher’s exact test and Mann–Whitney test for qualitative and quantitative variables, respectively. A p-value < 0.05 was considered to be significant.

**Results:** One hundred seventeen patients—77 men (65.8%), median age 53 [36–63] years—were included in the study. Underlying hematological malignancies were: acute myeloid leukemia (n = 51, 43.6%), myelodysplastic/myeloproliferative neoplasms (n = 22, 18.8%), acute lymphoid leukemia (n = 22, 18.8%), lymphoma (n = 11, 9.4%), other 11 (9.4%). Complete remission was achieved before HSCT in 61 (56%) patients. Forty-nine (46.2%) patients underwent myeloablative conditioning regimen and 23 (21.1%) received haploidentical grafts. Twenty-eight (23.9%) patients experienced disease relapse after HSCT and 48 (41%) graft versus host disease prior ICU admission. Median SAPS II was 49 [26–66] and SOFA score at day one 9 [6–11]. The ICU, hospital and 90-day mortality rates were respectively 38.5, 54.9 and 57.5%. In univariate analysis, factors associated with 90-day mortality were: SAPS II (p = 0.0011), invasive mechanical ventilation (p < 0.0001), vasopressors (p = 0.0012) and renal replacement therapy (p = 0.045). Mechanical ventilation was the only independent factor of 90-day mortality (OR 2.5–16.4], p < 0.0001) with mortality rate reaching 76.9% and even 86.7% among patients with uncontrolled hematological disease.

**Conclusion:** Prognosis of unselected HSCT recipients admitted to ICU remains poor, particurlaly among those receiving mechanical ventilation, and even more if hematological disease is not controlled. These results suggest that the implementation of an ICU triage policy determined both by intensivits and hematologists would be helpful to identify good candidates for ICU admission.

### P-178 Etiology and prognosis of acute respiratory failure in patients with primary malignant brain tumors admitted to the intensive care unit

#### Decavèle Maxens^1^, Weiss Nicolas^2^, Rivals Isabelle^3^, Prodanovic Hélène1, Lemasle Léa^1^, Idbaih Ahmed^4^, Mayaux Julien^1^, Similowski Thomas^1^, Demoule Alexandre^1^

##### ^1^Hôpital Pitié-Salpêtrière Charles Foix, Paris, France; ^2^IHU, Paris, France; ^3^PSL Research University, Paris, France; ^4^Inserm U 1127, Hôpitaux U, Paris, France

###### **Correspondence:** Decavèle Maxens - maxencesar@hotmail.fr

*Annals of Intensive Care* 2018, **8(Suppl 1):**P-178

**Introduction:** Acute respiratory failure (ARF) is a common event in patients with primary malignant brain tumors (PMBT). Even if many factors (corticosteroid therapy, swallowing disorders) suggest a specific etiologic spectrum, few data are available regarding its precipitating factors. Our first aim was to compare the causes of ARF between patients with PMBT and those with other type solid tumors. Our second aim was to identify, among PMBT, the factors influencing survival in ICU.

**Patients and methods:** Bicentric case–control study from March 1996 to May 2014. Patients with PMBT (cases, primary central nervous system lymphoma included) admitted for ARF were compared to patients with other kind of solid tumors (controls). The reason for admission “ARF” as well as the causes of ARF was determined by three experienced respiratory physicians and were required for inclusion: a respiratory rate > 20 cycles/min and a PaO2/FiO_2_ < 300 for patients in spontaneous breathing and only a PaO2/FiO_2_ < 300 for patients under mechanical ventilation. In both groups were excluded patients with metastatic solid tumors, benign tumors or tumors with more than 5 years of complete remission, recent post-operative patients, and patients with other immunodeficiency.

**Results:** A total of 84 cases and 133 controls were included. Main patients’ characteristics are reported in the Table 1. Acute infectious pneumonia was the leading cause of ARF in both groups but was more frequent among cases (77 vs. 36%, p < 0.001). Cardiogenic pulmonary edema and exacerbation of chronic respiratory diseases were more frequents in controls (10 vs. 37%, p < 0.001). Pulmonary embolism was similar between the two groups (10 vs. 4%, p = 0.143). Among acute infectious pneumonia, pneumocystis pneumonia (PCP) and aspiration pneumonia were more frequent in cases (19 vs. 2%, p < 0.001 and 19 vs. 8%, p < 0.001 respectively). In multivariate analysis cancer progression (OR-7.25 95%IC [1.13–46.45], p = 0.034), need for intubation (OR-7.01 95%IC [1.29–38.54], p = 0.022) and respiratory rate (OR-1.17 95% CI [1.05–1.30], p = 0.003) independently predicted ICU mortality of PMBT patients.

**Table 1:** Main comparative characteristics and outcomes between cases and controls

Variables	Case n = 84	Control n =133	*p-value*
General characteristics			
Age	58 [50–66]	72 [62–78]	< 0.001
Gender (male), *n(%)*	62 (74)	79 (59)	0.030
Obstructive respiratory disease, *n(%)*	8 (9)	36 (27)	0.002
Restrictive respiratory disease, *n(%)*	2 (2)	13 (10)	0.019
Chronic heart failure, *n(%)*	6 (7)	44 (33)	< 0.001
Corticosteroid therapy, *n(%)*	69 (84)	4 (3)	< 0.001
Performance Status 3–4, *n(%)*	59 (70)	39 (41)	< 0.001
Severity at admission			
PaO_2_/FiO_2_	161 [101–233]	166 [120–232]	0.113
Respiratory rate, *cycles/min*	30 [24–35]	27 [23–33]	0.472
SAPS II	46 [32–59]	39 [29–53]	0.534
Invasive mechanical ventilation, *n(%)*	33 (39)	52 (39)	0.978
Mortality			
ICU mortality, *n(%)*	20 (24)	32 (24)	0.966
Hospital mortality, *n(%)*	39 (46)	65 (49)	0.687

Case: patients with primary malignant brain tumors; Controls: patients with solid tumors of other type; SAPS II, Simplified Acute Physiology Score II; SOFA, Sequential Organ Failure Assessment

Continuous variables are expressed as median (first quartile–third quartile) and categorical data are expressed as number (%)

**Conclusion:** In PMBT patients, the causes of ARF differ significantly from other cancer patients. Up to 30% of the admissions was related to preventable causes (pulmonary embolism, PCP) and a curable cause was identified in the majority of cases. Our results suggest that PMBT alone is not a relevant criterion for ICU recusal.

### P-179 Drug Poisoning in the Emergency Department CHU Oran

#### Benbernou Soumia^1^, Ghomari Nabil^1^, Djebli Houria^1^, Amirat Norreddine^1^, Azza Abdelkader^1^, Iles Sofiane^1^, Bouyacoub Khalida^1^

##### ^1^Centre Hospitalier et Universitaires d’Oran, Oran, Algérie

###### **Correspondence:** Benbernou Soumia - gsoumia@hotmail.com

*Annals of Intensive Care* 2018, **8(Suppl 1):**P-179

**Introduction:** Drug intoxication is a common problem encountered in emergency departments. Poisoning remains a major cause of hospitalization for young people, and that of the elderly is constantly increasing. Objectives 1. Determine the epidemiological characteristics of addicted patients 2. Know the clinical manifestations of poisoning.

**Patients and methods:** A retrospective study of cases of acute poisoning recorded at the University Hospital Center CHUOran between January 2013 and December 2015 was carried out. Seizure on data processing by epi-info version 3.5

**Results:** 808 cases of acute poisoning, with an age ranging from 16 to 90 years. Female patients predominated with 67%. People between the ages of 16 and 25 are the people most affected by poisoning. The nature of poisoning is varied. In this series, analgesics were found to be the leading cause of acute intoxication, with 81 cases, 31% followed by psychotropic drugs (24%), benzodiazepines (21%), neuroleptics (6%), antiepileptics%) and antihistamines (9%). The majority of acute intoxications were managed within an average time of 2.51 ± 3.03 h with an interval between 0.5 and 48 h. In 74% of cases the poisoning was asymptomatic, there were digestive manifestations in 10% of patients, 7% Neurological, 6% Cardiovascular and 3% respiratory. We deplore 1 death in this series secondary to many drug poisoning.

**Conclusion:** Acute drug poisoning is a common reason for admission to the emergency department of Oran University Hospital. The large number of drug families offered for sale, as well as the heterogeneity of the symptomatology in case of overdose, make the diagnosis difficult, especially since the drug in question is often unspecified and the toxicological analysis is not exhaustive.

### P-180 Cyproheptadin poisoning

#### Sedghiani Ines^1^, Benjazia Amira^1^, Mrad Aymen^1^, Fredj Hana^1^, Brahmi Nozha^1^

##### ^1^Hopital Camu, Tunis, Tunisia

###### **Correspondence:** Sedghiani Ines - SEDGHIANIINES@yahoo.fr

*Annals of Intensive Care* 2018, **8(Suppl 1):**P-180

**Introduction:** Cyproheptadin (Periactin) is an histamine H1 receptor and serotonin antagonist. It has also an anticholinergic effects. It is used to treat allergic type symptoms and as an appetite stimulator.

Cyproheptadine poisoning has been rarely reported.

That’s why we conduct this study to identify the epidemiological characteristics and clinical outcomes of cyproheptadine poisonings and to define the association between the ingested dose and clinical manifestations.

**Patients and methods:** Retrospective study was performed between January 2008 and APRIL 2017. Epidemiological data clinical data and outcome were reviewed.

**Results:** A total of 56 patients (3 M 53F) were reviewed with a mean age of 22.43 [14–42]. All patients had no psychiatric backgroud. All cases were self inflicted. Mean ingested dose was 94 mg [61–750]. The median delay of symptoms onset was 3 h [1–14]. Neurological symptoms included delirium in 20% of cases agitation and hallucination. Forty two percent of patients had peripheral anti-cholinergic symptoms. Gastric lavage was performed in 5.5% of cases. Six patients were admitted to ICU and required mechanical ventilation (MV) for neurological impairement. Mean duration of (MV) was 17 h ± 4. The outcome was favorable in all cases. Length of hospital stay was21 h [6–74]. There is no correlation between coma and assumed ingested dose.

**Conclusion:** Cyproheptadine over dose causes mainly neurological effects resulting from antichlinergic syndrom. Outcome is generally favorable.

### P-181 Fatal carbon monoxide intoxication in south of Tunisia: epidemiological and medical-legal study

#### Zribi Malek^1^, Ben Amar Wiem^1^, Bennour Walid^1^, Feki Nihel^1^, Bardaa Sami^1^, Hammami Zouheir^2−1^, Maatoug Samir^1^

##### ^1^University Hospital, Sfax, Tunisia

###### **Correspondence:** Zribi Malek - ahmedchakroun2023@gmail.com

*Annals of Intensive Care* 2018, **8(Suppl 1):**P-181

**Introduction:** Carbon monoxide intoxication is a public health problem in Tunisia and around the world. Currently, it is unclear the impact of this type of poisoning in our country for lack of declarations. We propose in our work to study the epidemiological characteristics of fatal carbon monoxide intoxications collected in the forensic Pathology department of the university hospital in Sfax, Tunisia, to describe the different steps used in forensic diagnosis of fatal carbon monoxide intoxication and to propose preventive measures to reduce the rate of these intoxications.

**Patients and methods:** It is a retrospective study of 29 cases of fatal carbon monoxide intoxications collected in the Forensic Pathology Department of the University Hospital in Sfax, Tunisia during 06 years (1 January 2011 to 31 December 2016). Commemoratives were collected from medical and police records. A forensic autopsy and a toxicological analysis were carried out in all cases.

**Results:** Fatal carbon monoxide intoxication is the leading cause of toxic death in Sfax during the period of our study. We notice a decrease in the incidence of this type of intoxication. The average age of deaths was 36 years and 2 months with male predominance. The peak frequency of intoxication was in cold season. The most frequent form of intoxication was accidental. The source of carbon monoxide was mainly the defective water heater often placed in poorly ventilated areas. The classic carmine red-color of lividity was found in the majority of cases. Myocardial distress, favored by hypoxia, has been reported in two subjects with a pathological coronary artery. The mean HbCO level was 51.59%. However, account must be taken of the survival time and the time elapsed between death and dosing of HbCO. The incidence of fatal carbon monoxide intoxication has decreased since 2011 and the victim profile has not changed too much.

**Conclusion:** The fatal carbon monoxide intoxication is still persists as a public health problem in Tunisia. The reduction of its frequency requires the implementation of a well-structured prevention plan based on epidemiological data from a national registry. The identification of these data requires mandatory reporting of this type of intoxication in Tunisia.

### P-182 What do you Know about Olanzapine poisoning? A descriptive study

#### Barghouth Manel^1^, Bachrouch Mayssa^1^, Fatnassi Meriam^1^, Khzouri Takoua^1^, Foudhaili Nasreddine^1^, Mrad Aymen^1^, Brahmi Nozha^1^

##### ^1^Centre Mahmoud Yaakoub d’assistance médicale urgente, Tunis, Tunisia

###### **Correspondence:** Barghouth Manel - manelbarghouth@outlook.fr

*Annals of Intensive Care* 2018, **8(Suppl 1):**P-182

**Introduction:** Olanzapine is an atypical antipsychotic drug frequently prescribed in the treatement of bipolar disorder and schizophrenia. Acute poisoning with this molecule is rarely reported. Through this study we aimed to evaluate the incidence and describe the different clinical features of acute olanzapine poisoning.

**Patients and methods:** Retrospective analysis of all cases of olanzapine intoxication admitted in 12-bed teaching ICU between January 2013 and Decembre 2017. Inclusion criteria were patient age ≥ 14 year, acute olanzapine intoxication, the intoxication severity was assessed by the Poisoning Severity Score (PSS) of the European Association of Poison Centres and Clinical Toxicologists. The evaluation of electrocardiograms was performed in the first day of hospitalization. The durations of QRS and QTc was measured and arrhythmias and conduction disorders was identified.

**Results:** 24 patients were included, the mean age was 35 ± 11 years. They were 11 males and 13 females. Long term treatment with olanzapine was noted in 19 patients (79%) who suffered from psychiatic desease. The supposed ingestion dose ranged from 25 to 600 mg. The mean consulting time was 5 ± 4 h after the ingestion. Olanzapine was co-ingested with others drugs in 16 patients (66%). Co-ingested drugs were—benzodiazepine (n = 5), levomepromazine (n = 5), serotonin recapture inhibitor (n = 3), amitriptilyne (n = 2) and biperiden (n = 1). The PSS was moderate in 9 cases (37.5%), severe in 14 cases (58%) and fatal in 1 case. The main clinical signs were tachycardia and miosis in 62% of cases each of them (n = 15), agitation in 33% of cases (n = 8). ECG abnormalities has been detected such as prolonged QTc in 5 cases with a mean duration of 460 ± 28 ms. In the group of monointoxication (8 patients) the PSS was moderate in 3 cases (37.5%), severe in 4 cases (50%) and fatal in one case. The Coma Glosgow Scale was < 12 in 14 cases. Mechanical ventilation was required in 58% of cases (n = 14%) with a mean duration of 23 ± 8 heures. The mean duration of ICU stay was of 50 ± 30 h. Twenty three patients recovered during the hospitalisation, one patient died with severe poisoning.

**Conclusion:** As showed in this study, acute olazapine poisoning could be severe, and lead to death sometimes.

### P-183 Acute selective serotonin reuptake inhibitors (SSRIs) poisoning

#### Bachrouch Maissa^1^, Manel Barghouth^2^, Foudhaili Nassreddine^2^, Khzouri Takoua^2^, Fatnassi Mariem^2^, Mahdhaoui Soumaya^2^, Mrad Aymen^2^, Nozha Brahmi^2^

##### ^1^Nabeul, Tunisia; ^2^Hopital de tunis, Tunis, Tunisia

###### **Correspondence:** Bachrouch Maissa - maissa_bachrouch@yahoo.fr

*Annals of Intensive Care* 2018, **8(Suppl 1):**P-183

**Introduction:** Selective serotonin reuptake inhibitors (SSRIs) have been considered for their low toxicity comparatively to antidepressant agents. The present study aims to describe clinical features and prognosis of poisoning SSRIs.

**Patients and methods:** A retrospective study of patients admitted to our 12-bed teaching ICU for acute SSRIs poisoning over a period of 8 years from January 2009 to December 2016. SSRIs poisoning was retained on a history of over dose ingestion, clinical signs and positive urine samples for SSRIs.

**Results:** Thirty seven patients were collected, the middle age was 32 ± 15 years with a female predominance (86.5%). A psychiatric history with depressive syndrome was noted in 67.6% and a history of suicide in 13.5%. Paroxetine was the main invoked drug (n = 20), followed by sertaline (n = 8), then fluoxetine (n = 6), venlafaxine (n = 1) citalopram (n = 1). The mean supposed ingestion dose was 342.9 mg. Intoxication was pure in 13 cases and associated with other drugs in 24 cases—benzodiazepines (n = 17), klippal (n = 2), amisulpride (n = 1), Non-steroidal anti-inflammatory drug (n = 1), prazin (n = 1) and promethazine (n = 1). Neurological examination found drowsiness and mydriasis in 46% of cases (n = 17), coma in 13.5% (n = 5), agitation (n = 5), tremor (n = 5), hyperreflexia (n = 2), hypersudation (n = 1), fever (n = 1) and diarrhea in one patient. The QT was lengthened in five cases. Treatment was symptomatic. Five patients (13.5%) required mechanical ventilation with average of ventilation duration of 21.2 h. All patients discharged alive the ICU.

**Conclusion:** SSRIs poisoning is mainly manifested by serotonergic syndrome. Evolution is favorable in the majority of cases. Mechanical ventilation could be required.

### P-184 Hemodynamic profile of shocks induced by dihydropyridine calcium channel blocker poisoning

#### Khzouri Takoua^1^, Mrad Aymen^1^, Fatnassi Meriem^1^, Barghouth Manel^1^, Foudhaili Nasreddine^1^, Barbouch Mayssa^1^, Mahdhaoui Soumaya^1^, Ben Hamida Samia^1^, Brahmi Nozha^1^

##### ^1^10, Rue Abulkassem Chabbi, Tunis, Tunisia

###### **Correspondence:** Khzouri Takoua - takoua_kh2@yahoo.fr

*Annals of Intensive Care* 2018, **8(Suppl 1):**P-184

**Introduction:** Acute Calcium Channel Blockers (CCB) poisoning remains infrequent despite their increasing use. In our country, dihydropiridines are the most prescribed ones. Very few works have studied the hemodynamic profile of acute dihydropyridines poisoning either by invasive means (right cardiac catheterization, trans-pulmonary thermodilution) or non-invasive (cardiac ultrasound). In this perspective, we carried out this study whose main objective was to illustrate the different hemodynamic profiles of shocks induced by dihydropyridine CCB Poisoning.

**Patients and methods:** It was an observational retrospective study spread over 12 months from 1st January 2016 to 31th December 2016 in a teaching toxicological ICU, including all patients admitted for acute dihydropyridine CCB poisoning, who presented a shock and underwent right hemodynamic exploration.

**Results:** During the study period, CCB poisoning accounted for 3.15% (n = 24) of all the acute poisoinings requiring hospitalization in our Intensive Care Unit. Among them, 21 had taken dihydropyridine which represents 87.5%. Four women aged of 29 [21, 47] were eligible. All the exposures were single-drug. The dihydropyridines involved were amlodipine in 2 cases with a median value of supposed ingested dose (SID) of 297.5 mg and nicardipine in the other two ones, the median SID was 1000 mg. The delay of consultation was of 2.25 ± 1 h after ingestion. Gastrointestinal decontamination was performed in one patient with activated charcoal. The 4 patients developed a shock within 3 h, treated by initial vascular filling on average 1000 ml of crystalloids, noradrenaline alone in 3 cases and with a combination of dobutamine in one patient. Other adjuvant treatments (high dose insulin, calcium salts) have been used in all patients. Their hemodynamic profile evaluation by right-handed catheterization Swan-Ganz was in favour of vasoplegia in 3 cases with median values of systemic vascular resistances (SVR) of 224 dynes.s.cm-5, of cardiac Output (CO) of 14 (L min), and of the arteriovenous oxygen difference of 4.76. The fourth patient’s shock had mixed nature with SVR of 507 dynes.s.cm-5 and CO of 3.7 (L min). All patients were discharged from the ICU with a mean length of stay of 14 days.

**Conclusion:** The dihydropyridine calcium channel blockers poisoning exposes to the shock risk due to several mechanisms. The clinician must be warned to look for signs of severity and understand its mechanisms by using the hemodynamic study in order to improve its management.

### P-185 Management of voluntary drug poisoning at Oran University Hospital

#### Goulmane Mourad^1^, Alachaher Djamel^1^, Djebli Houria^1^

##### ^1^CHU d’Oran, Oran, Algeria

###### **Correspondence:** Goulmane Mourad - m.goulmane@hotmail.com

*Annals of Intensive Care* 2018, **8(Suppl 1):**P-185

**Introduction:** Voluntary drug intoxication (VDI) continues to be a major health problem in many developed and developing countries. In Algeria, this has become a worrying concern. Awareness-raising is launched to prevent the public from these dangers. VDI are intentional or rarely accidental and can be individual or collective and affect all age groups. The VDI represents the first reason for hospitalization in the Emergency Department University hospital of Oran.

**Patients and methods:** In Algeria there is no national or regional register of voluntary intoxication. Knowledge of the causes of drug poisoning should therefore be extrapolated from foreign studies. To draw up an assessment of the IMVs, a retrospective study was carried out over the 2 years (2015–2016). This survey consisted of collecting data on the nature of the drug, age, sex, major toxidromes, severe IMVs requiring hospitalization in ICU, mortality, E.T.C scores and GLASGO scores.

**Results:** 1650 cases of acute poisoning were collected, with a predominance in patients aged between 16 and 25, a percentage of 31.77%. In addition, most patients were female with 60.98%, a sex ratio of 1.56 with p < 0.05. The main toxidromes were—opioid syndrome in 27% of cases and anticholinergic syndrome in 23% of cases. ETC with a score of > 9% accounted for 93% of patients. Severe VDI requiring resuscitation hospitalization were 8%.

**Conclusion:** Acute poisoning remains high and steady in the Oran region and the under-25 age group represents the most affected category. Awareness campaigns must be launched throughout the year to better conserve and store medicines, phytosanitary products and other chemicals. Improved socio-economic conditions would help to reduce voluntary intoxication.

**Reference**

1. Staikowsky, F., Uzan, D., Grillon, N., Pevirieri, F., Hafi, A., Michard, F. 1995. Voluntary drug poisoning received in an emergency department. Medical Press. Vol 24 (28), pp. 1296–1300. SRLF.

### P-186 Epidemiological profile of scorpionic envenomations treated at the Mahres Resuscitation Department

#### Kaaniche Fatma^1^, Allala Rania^1^

##### ^1^Sfax, Tunisia

###### **Correspondence:** Kaaniche Fatma - fatma_kaaniche@yahoo.fr

*Annals of Intensive Care* 2018, **8(Suppl 1):**P-186

**Introduction:** Scorpionic envenomation is unevenly distributed throughout the world and is particularly frequent in some regions of the world, notably North Africa. The purpose of this work is to describe the epidemiological profile of the scorpionic envenomations admitted to the resuscitation department of Mahres.

**Patients and methods:** A prospective study conducted at the Mahres intensive care unit over a period of 6 months (15 02 2017 until 15 08 2017), including all patients admitted for scorpion envenomation.

**Results:** We collected 102 cases of patients admitted to the resuscitation department of Mahres from 15 02 2017 to 15 08 2017, including 10 cases of scorpionic envenomations, i.e. 0.098%. The median age was 43 years with extremes ranging from 20 to 78 years. The sex ratio was 4. Scorpion stings occurred at night in 80% of patients, 60% in the first half of the night (between 6 pm and 11–59 pm) and 20% in the second half of the night (0 to 5 h). Venom inoculation points were in the lower limbs in 80% of cases, followed by upper limbs (20%). The color of the incriminated scorpion was yellow in 80%, black in 10% and unspecified in 10% of the cases. For admission classes, there were 10% Class I, 80% Class II and 10% Class III. The traditional therapeutic gestures practiced by the patients or their entourage were the laying scarification (50%) and the suction (10%). All patients received anti-scorpion serum, an analgesic, serum and tetanus vaccine. The progression was favorable in all cases after an average hospital stay of 4 ± 1 days.

**Conclusion:** Scorpionic envenomations are indeed a reality in Mahres with a non-negligible frequency despite under-reporting of cases treated by traditional medicine or in other hospitals. They mostly affect young people and the associated clinical manifestations often remain benign.

### P-187 Severe poisoning by drugs and or illicit substances in pediatric intensive care Oran Algeria

#### Tabet Aoul Nabil^1^, Addou Zakaria^1^, Douah Ali^1^, Youbi Houari^1^, Moussati Mohammed^1^, Belhabich Kamel^1^, Abada Sanaa^1^, Mir Souad^1^, Zerhouni Amel^1^, Aouffen Nabil^1^

##### ^1^EHS pédiatrique de Canastel, Oran, Algeria

###### **Correspondence:** Tabet Aoul Nabil - tabetrea@yahoo.fr

*Annals of Intensive Care* 2018, **8(Suppl 1):**P-187

**Introduction:** Severe pediatric poisoning is defined by the need for intensive care monitoring due to the nature, quantity of the substance and or clinical manifestations. It is one of the frequent reasons for admission to emergency and resuscitation. The purpose of this work is to identify poisoning in children admitted to pediatric intensive care units in order to assess the frequency, identify the products involved, and the clinical and evolutionary aspects.

**Patients and methods:** This is a descriptive study over a 24-month period in the Canastel Oran multi-purpose pediatric intensive care unit from July 2016 to July 2017. We included all children aged 0–15 years admitted for ingestion and inhalation of products toxic.

**Results:** 39 children admitted to pediatric intensive care, mean age was 5 years, 70% under 6 years with extremes of 02 months and 15 years, a female predominance of 60% was observed with a slight predominance of accidental poisoning (56%) Compared to voluntary poisoning (44%). In 87% the toxic is ingested orally. The most frequent toxicants were drugs with 19 cases (35%), mostly antidepressants and antiepileptics, followed by organophosphates with 10 cases (26%), CO 5 cases (12%), petroleum products and plants with 5 cases (12%). The main clinical signs were neurological signs (76%) with predominance of coma and convulsions in 19 cases (43%), respiratory distress was present in 6 cases (15%) and digestive signs 8 cases (20%). For therapeutic management gastric lavage, charcoal and antidotes were the most frequent treatments. The evolution was marked by a mortality of 2% or a death secondary to a poly-medicinal intoxication voluntary in a girl of 10 years. Mechanical ventilation in 5 cases (12%) and an average hospital stay of 2 days.

**Conclusion:** Acute poisoning is a medical emergency that may require resuscitation. Young children are most exposed with drugs are the most frequently incriminated. We propose, as a preventive measure, companions of information on the dangers of toxic products and especially of medicines by the surveillance of the child and the regulation of certain products.

### P-188 Serum neuron specific enolase as predictor of outcome after cardiac arrest

#### Grangé Steven^1^, Beignot Devalmont Edouard^1^, Misset Benoit^1^, Artaud-Macari Elise^1^, Beduneau Gaetan^1^, Beuzelin Marion^2^, Carpentier Dorothée^1^, Girault Christophe^1^, Hobeika Sinad^1^, Lemaitre Caroline^1^, Tamion Fabienne^1^

##### ^1^CHU de Rouen, France; ^2^Hôpital de Dieppe, France

###### **Correspondence:** Grangé Steven - stevengrange@gmail.com

*Annals of Intensive Care* 2018, **8(Suppl 1):**P-188

**Introduction:** The place of neuron specific enolase (NSE) dosing remains uncertain as an indicator of neurological prognosis after a cardiac arrest, the threshold value for predicting an unfavorable evolution being variable from one study to another. Our objective was to determine a NSE cut-off value predictive of poor neurological outcome after a cardiac arrest.

**Patients and methods:** We realized a monocentric prospective trial in a medical ICU of a French University hospital from January 1st 2016 to December 31th 2016. All patients over 18 years old hospitalized for a cardiac arrest in medical ICU were included. Patients who died during the first 24 h or admitted for cardiac arrest with a neurological cause were excluded. Serum NSE values (Elecsys NSE test, Cobas^®^ analyzer) were assessed at H24 and H72 after cardiac arrest. Somatosensory evoked potentials were recorded between H24 and H72. The primary endpoint was neurological outcome at 6 month using the cerebral performance category scale (CPCS). CPCS 1 or 2 was considered as favorable outcome and CPCS higher than 2 as poor outcome. Data were collected using cardiologic or neurologic consultations report, or by phone call to the patient. Using a ROC curve we determined the NSE value at H24 with higher specificity and acceptable sensitivity.

**Results:** We included 48 patients. Average age was 62 years old. No-flow time and low-flow time were respectively 3.7 and 17 min. Hypothermia was performed in 30 (63%) patients. 27 patients (56%) died in the ICU. The 30-day and 6-months survival rates were respectively 44 and 42% with a favorable outcome of 35% at 6 months. On the ROC curve we found a cut-off value of 41 ng ml with specificity of 0.95 CI 95% (0.75–0.99) and a sensibility of 0.64 CI 95%(0.44–0.81). Area under curve was 0.828 CI 95% (0.713–0.942). 9 out of the 10 patients with a rising NSE between H24 and H72 had an unfavorable outcome. Among patients with NSE > 41 ng ml, the cortical N20 responses were bilaterally present in 4 of them.

**Conclusion:** In our study NSE value over than 41 ng ml at H24 was predictive of poor neurological outcome after cardiac arrest. NSE may prove to be a useful marker in patients with present N20 responses, possibly limiting the duration of hospitalization by introducing therapeutic limitation or withdrawal of support.

### P-189 Physicians assessment of prognosis in ICU patients with brain damage

#### Laradh Mehdi^1^, Clerc-Urmes Isabelle^1^, Cravoisy-Popovic Aurélie^1^, Mahjoub Khaoula^1^, Labroca Pierre^1^, Lemarié Jérémie^1^, Conrad Marie^1^, Nace Lionel^1^, Gibot Sébastien^1^, Bollaert Pierre-Edouard^1^

##### ^1^CHRU de Nancy, Vandoeuvre, France

###### **Correspondence:** Laradh Mehdi - m.laradh2@chru-nancy.fr

*Annals of Intensive Care* 2018, **8(Suppl 1):**P-189

**Introduction:** Outcome prediction in ICU patients with severe brain damage is a difficult task with observed heterogeneity in physicians estimation. The aim of the survey was to evaluate the prognostic estimates and treatment recommendation of intensivists in real patients with various causes of severe brain damage.

**Patients and methods:** A web anonymous survey including a summarized clinical report of four patients who stayed in the ICU was submitted to French intensivists. Patient 1 presented with prolonged hypoglycemic coma, patient 2 with intracerebral hemorrhage, patient 3 with central and extra pontine myelinolysis, patient 4 with a brainstem hemorrhage. All these patients received full treatment in the ICU and had a 6-month follow-up. Physicians were provided with the four clinical vignettes including clinical history, brain imaging and other relevant exams (CSF, EEG,…), evolution of symptoms within the first days of the ICU stay. They had to estimate 6-month outcome using modified Rankin scale (mRS) where a score from 0 to 3 was considered as a good outcome and 4 to 6 as a poor outcome. They had to provide a recommendation about care among the following—full treatment, care limitation, care withdrawal.

**Results:** 109 physicians completed the survey. There were 37 (33.9%) female. 48 (44%) respondents were residents and 20 (18.3%) had a > 10-year of experience. Patients 1 and 2 had a good 6-month outcome with mRS 1 and mRS 2 respectively while patients 3 and 4 had a poor outcome, both with mRS 5. Correct prognosis estimations were 36 (33%), 31 (28.4%), 97 (89%) and 100 (91.7%) in patients 1 to 4 respectively. Care limitation or withdrawal was recommended by 70 (64.2%), 40 (36.7%), 73 (67%) and 73 (67%) respondents in patients 1 to 4 respectively. Of interest, care withdrawal was recommended by 2 (1.8%), 4 (3.7%), 19 (17.4%) and 25 (22.9%) respondents in patients 1 to 4 respectively. Univariate analysis did not display any factor related with a good prediction of prognosis.

**Conclusion:** In this study, overall predictions were pessimistic with important variations among respondents. Although decisions to withdraw life sustaining care were relatively low with regard to estimated prognosis, both inappropriate care limitation leading to self-fulfilling prophecies and unreasonable prolonged life supportive care could result from these estimations.

### P-190 Factors related to the refusal of organ donation

#### Si Larbi Anne-Gaëlle^1^, Gaudin Virginie^1^, Quere Regis^2^, Charpentier Julien^2^, Zuber Benjamin^3^, Breynaert Sophie^2^, Hayon Jan^4^

##### ^1^Hôpital Foch, Suresnes, France; ^2^Paris, France; ^3^Versailles, France; ^4^Hôpital de Poissy, France

###### **Correspondence:** Si Larbi Anne-Gaëlle - agsl17@club-internet.fr

*Annals of Intensive Care* 2018, **8(Suppl 1):**P-190

**Introduction:** Organ harvesting is a national priority because of the shortage of organs, responsible each year for the lengthening of transplant waiting lists. Among the identified potential donors, the main cause of non-harvesting is the refusal of organ donation (OD), which exceeds 30% in France and 40% in Paris area.

**Patients and methods:** In a network of 6 hopitals, each procedure on a potential donor by the donor co-ordinator is recorded in a report. After selection of the reports with interviews with relatives about OD between 2012 and 2015, the data in the reports were collected and a multivariate logistic regression was performed to identify the factors associated with the refusal.

**Results:** 571 reports with interviews about OD was found. The overall opposition rate is 43.3%. Among the 42 children (7.3% of cases) the opposition rate is 47.6%. Among adults, 147 (27.8%) expressed their will about OD during their lifetime, with an opposition rate of 43.5% and for the 382 (72.2%) of them who never expressed their will, the opposition rate is 42.9%. The factors associated with opposition in multivariate analysis are presented in Table 1. When the deceased had never expressed their will, the reasons given by the relatives to justify the refusal are specified in 87.5% of the reports. These are religious grounds (20%), cultural grounds (12%), respect for physical integrity (15%). In 18% of the cases, relatives believe that the deceased would have been opposed, and in 16% of the cases, they choose to refuse because they do not know the deceased’s opinion.

**Table 1:** Factors associated with opposition in multivariate analysis

Factor tested	Hazard ratio	p	Opposition rate	Frequency of the factor
History of ethylism	0.31	0.002	24%	12%
History of psychiatric disease	0.54	0.09	26%	12%
Foreign patient or family	2.73	0.03	68%	11%
Practice of a religion: yes	1		58%	32%
Practice of a religion: no	0.04	< 0.001	27%	24%
Practice of a religion: not specified	0.08	< 0.001	41%	43%
Organ donation first addressed by relatives	0.2	0.004	13%	8%
Presence of a spouse	2.43	< 0.001	49%	52%
Presence of child/children	1.48	0.07	47%	52%
Disagreement between relatives	2.56	0.03	68%	12%
Interview during the night (20:00 to 8:00)	1.68	0.06	51%	19%

**Discussion:** French law is based on presumed consent. Despite this, it is noted that when patients had never expressed their opinion about OD (and therefore had not refused it), the opposition rate reached 43.5% and was comparable to the patients who had expressed themselves.

**Conclusion:** In our study, factors related to refusal of OD are mainly related to the characteristics of the deceased (religion, culture, history of ethylism) and those of relatives (disagreement, presence of a spouse), but little to the way of doing the interview. However, there is a trend for less opposition when the interview is conducted during the day (between 8–00 and 20–00). On the other hand, when relatives first address the issue of OD, the opposition rate is lower.

### P-191 Identification of power relations between medical and paramedical staff during withdrawal or limitation of care decision: a questionnaire study

#### Piazza Sara^1^, De Montmollin Etienne^1^

##### ^1^Centre hospitalier de Saint-Denis, Saint Denis, France

###### **Correspondence:** Piazza Sara - piazza.sar@gmail.com

*Annals of Intensive Care* 2018, **8(Suppl 1):**P-191

**Introduction:** French intensive care society guidelines and the Claes-Leonnetti Law recommend that intensive care teams organize collegiate and multidisciplinary discussions regarding limitation and withdrawal of care decisions. These moments, coined ethical staffs in our unit, require freedom and safety of speech, which can be difficult to obtain when people are caught in hierarchical and or power relations. We sought to assess the representations, perceptions and opinions of ICU personnel regarding ethical staffs.

**Patients and methods:** A questionnaire, developed by the ICU psychologist, was distributed to the entire unit (secretaries, nurses, nursing auxiliaries, doctors) over a period of 6 months. This 30-question questionnaire covered session organization and power relations between participants.

**Results:** Among the 86 questionnaires distributed in the ICU, 76 were retrieved and analyzed. Medical function was associated by respondents with roles linked with power (leading, knowledge, decision, explanation) whereas paramedical function was associated with roles linked with care (perception, account, spokesperson) (Fig. 1). Regarding representations of decision making, nurses were considered as decision makers in 48 cases (63%) and doctors in 76 cases (100%).

**Fig. 1:**
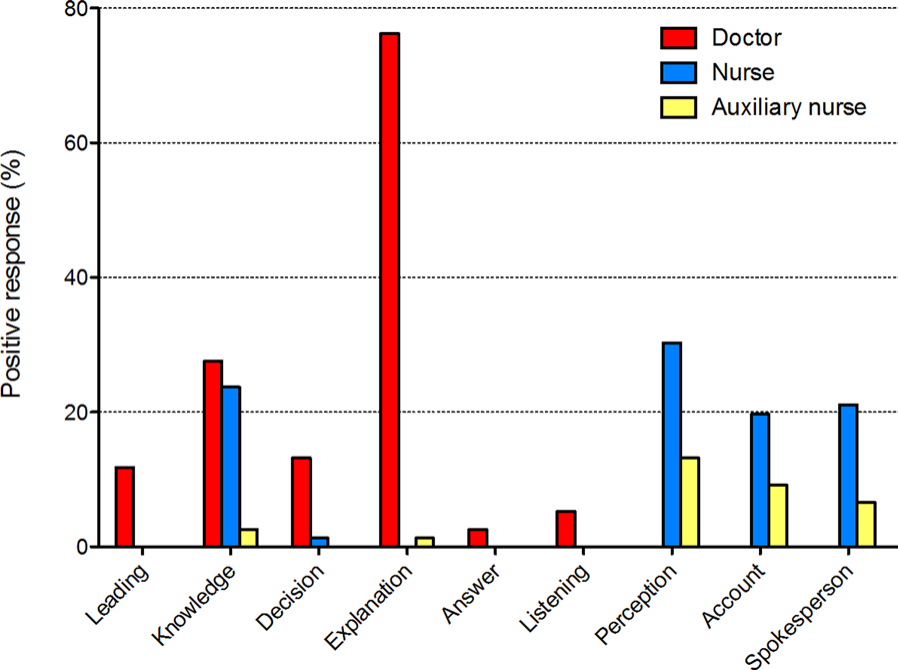
Positive responses according to medical function from doctors (red), nurses (blue) or auxiliary nurse (yellow)

**Discussion:** Although ethical staffs are presented as a place where each opinion counts, stereotypes representation appear in the different roles assigned—on one side doctors are in charge of explanation and decision, and on the other side, nurses are taking care of patient’s feelings and assume a role of spokesperson. These stereotypes correspond to gender stereotypes assigning women to positions of care, empathy and relationship, and men to more intellectual and leading skills. These gender stereotypes attest a hierarchy internalized by each one, as highlighted by social sciences and gender studies.

**Conclusion:** Our results highlight the existence of a global idea, shared by the majority—doctors are decision makers and therefore are in a power relation regarding paramedical staff. This hierarchical relationship persists in this moment wished egalitarian (each opinion would count equally). These is a linkage between professional power relations and gender power relations, which show an association between doctor and masculine “qualities” and caretakers and feminine “qualities”. These power relations are rarely acknowledged but could have a significant impact on the decision process of these meetings, and should be further investigated.

### P-192 Evaluation of the decision-making process leading to non-admission of patient to the intensive care unit: ETICAR

#### Leiva Rojas Uriel^1^, Meunier-Beillard Nicolas^2^, Georgiev Stefan^1^, Fischbach Georges^1^, Cosic Olga^1^, Feissel Marc^1^, Kalakhy Rachid^1^, Barrot Loic^1^, Badie Julio^1^, Toitot Amaury^1^, Ecarnot Fiona^3^, DargentAuguste^2^, Rigaud Jean-Philippe^4^, Quenot Jean-Pierre^2^

##### ^1^HNFC, Trevenans, France; ^2^CHU de Dijon, France; ^3^CHU de Besançon, France; ^4^Ch de Dieppe, France

###### **Correspondence:** Leiva Rojas Uriel - Uriel.LEIVAROJAS@hnfc.fr

*Annals of Intensive Care* 2018, **8(Suppl 1):**P-192

**Introduction:** In daily practice, admission to the intensive care unit (ICU) usually does not raise any major ethical problems. Difficulties arise mainly in acute situations requiring intensive care that have not been anticipated and therefore, not adequately prepared and discussed. We hypothesized that non-admission of a patient to the ICU must occur in the following circumstances—(1) with the patient’s agreement, expressed either directly or through advance directives (AD), or as relayed by a surrogate or the family + (2) according to a collegial decision-making process (if the patient is decisionally incapacitated) + and (3) after seeking the opinion of an external consultant. The decision-making process must be documented in the patient’s medical file.

**Patients and methods:** Prospective, observational study in two hospitals (one large university hospital, one regional non-acamedic hospital) over a period of 2 months. Inclusion criteria were—patients aged ≥ 18 years presenting with failure of at least 1 organ that was directly life-threatening and requiring life-sustaining therapies. Complete data collection was performed for each patient.

**Results:** A total of 31 patients were included (61% from the emergency department and 39% from medical wards). The decision not to admit the patient to the ICU was taken—(1) during night duty for 19 patients (61%) + (2) by a senior physician in 100%, and (3) after clinical examination in 25 (80%). The main reasons justifying the decision not to admit to the ICU were—(1) metastatic cancer in 8 patients (26%) + (2) total loss of autonomy in 8 (26%) + (3) severe cognitive impairment in 4 (13%) + (4) premorbid state in 1 (3%) + (5) chronic organ failure for 2 (5%) + and (6) presence of AD (written or oral) specifying that the patient did not wish to be admitted to the ICU in 8 (26%). This study raises several points concerning the decision-making process for patients requiring intensive care. First, collegiality is observed in almost all situations of non-admission (97%). Second, an outside consultant was contacted in around 30% of cases. Third, 26% of patients had AD. Fourth, the family or entourage were consulted in less than 40% of cases and finally, in around 75% of cases, the decision-making process was documented.

**Conclusion:** This study shows that in emergency situations, it is more difficult to take adequately structured decisions regarding ICU admission than, for example, decisions regarding limitation or withdrawal of treatment in the ICU.

### P-193 Long-term survival and quality of life in patients discharged at home from the ICU with tracheostomy

#### Sboui Ghada^1^, Beji Olfa^1^, Atig Rabia^1^, Bouhamed Chafia^2^, Mani Radhouene^3^, Hmouda Houssem^1^

##### ^1^Medical Intensive Care Unit, Sousse, Tunisia; ^2^Emergency Department, Kairouan, Tunisia; ^3^Oral and maxillofacial Department, Sousse, Tunisia

###### **Correspondence:** Sboui Ghada - ghadasb@hotmail.com

*Annals of Intensive Care* 2018, **8(Suppl 1):**P-193

**Introduction:** As known, tracheostomy is performed to improve quality of life (QOL) in patients requiring prolonged mechanical ventilation. It is indicated to facilitate care of critically ill patients, in order to minimize risks of oro-tracheal intubation, and enhance recovery, allowing early discharge from ICU with home ventilation. We aimed by this study to evaluate long-term survival and QOL in Tunisian patients discharged from the ICU with tracheostomy, as well as related burden assumed by their relatives.

**Patients and methods:** Patients who were admitted to the ICU between 2015 and 2017 were eligible for inclusion in this retrospective cohort if they had a tracheostomy during their ICU stay, and were discharged at home with a tracheostomy canula. For survivors, we used the Short Form Health survey (SF12) to assess their QOL at home. We estimated the degree of autonomy using the ADL scale. To assess burden assumed by caregivers (family members most of the time) we used the short version of Zarit burden interview. Exclusion criteria were refusal of the interview or unavailability on the phone call.

**Results:** Fourteen patients were discharged at home with a tracheostomy canula. Only twelve responded to the phone call. Four patients died 1 month later. Amoung the 8 survivors, the removal of the tracheostomy canula was successful in 3 patients after a mean duration of 30 days. Main findings are summarized in Table 1.

**Table 1:** Main findings

Variables	Value, Range/(%)
Age (years)	58.9 [20–81]
Gender	
Men	4 (50%)
Women	4 (50%)
CHARLSON Comorbidity Index	2 [1–3]
APACHE II	17.5 [9–22]
Time to tracheostomy (days)	12 [7–18]
Indications of tracheostomy	
Difficult weaning	6 (75%)
Neurological cause	2 (25%)
Time to ICU discharge (days)	32.5 [19–91]
ADL scale (/6)	3.5 [0–6]
SF 12 scale (%)	
Physical health scale	40.27 [23.17–53.57]
Mental health scale	41.71 [32.95–49.76]
Mini-Zarit scale (/7)	6 [0–7]

**Conclusion:** Tracheostomy shows good acceptance and acceptable QOL. It allowed shorter length of stay in the ICU and long-term survival after discharge from the ICU, and should be encouraged for Tunisian patients. In contrast, the QOL of patients’ relatives was more affected, with significant burden and work load.

### P-194 Psychological support for intensive care survivors

#### Longueville Virginie^1^, Peigne Vincent^1^, Thouret Jean-Marc^1^

##### ^1^Centre Hospitalier Métropole Savoie, Chambéry, France

###### **Correspondence:** Longueville Virginie - virginie.longueville@ch-metropole-savoie.fr

*Annals of Intensive Care* 2018, **8(Suppl 1):**P-194

**Introduction:** Intensive care survivors present often some psychological disorders linked with experience memory loss or nightmares. The use of patient diaries has been developed and implemented by clinical staff to improve the quality of life after intensive care. Patients received their diaries at ICU discharge. This study was conducted in order to understand the potential benefits for patients the diary on prevalence anxiety, depression and post traumatic disorders during recovery.

**Patients and methods:** A structured interview study was administered to adult critical illness survivors who received ≥ 48 h of mechanical ventilation in a medical and surgical Intensive care unit. After 6 months, this patients answered at two questionnaire—Hospital Anxiety and Depression Scale (HAD) and a screening instrument for PTSD (QSPT).

**Results:** From the survivors at 6 months, 36 patients answered the questionnaires. We have two groups—18 patients had a diary and 18 patients no diary. But these group are so low currently to compare results. Despite the diary, 33% had a QSPT score > 51, indicating a higher post traumatic disorders. 9 patients (25%) presented a anxiety score > 11 and 11 patients (30.5%) had a depression score > 11. These results underline the need of psychological support after the stay.

**Conclusion:** Many survivors of intensive care unit reported a high level of psychological distress. It seems important offer at this patient a psychological support after an intensive care unit stay. Most patients needs return in intensive care unit to understand some elements of hospitalization. Actually, this support lack to screening and treatment this psychological morbidity.

### P-195 Prevalence and description of the complications following a percutaneous coronary intervention for a myocardial infarction in non-cardiac critically ill patients: a retrospective single-center study

#### Roué Morgan^1^, Desnos Cyrielle^1^, Voiriot Guillaume^1^, Blayau Clarisse^1^, Djibre Michel^1^, Fulgencio Jean Pierre^1^, Garnier Marc^1^, Fartoukh Muriel^1^, Labbe Vincent^1^

##### ^1^Hôpital Tenon, Paris, France

###### **Correspondence:** Roué Morgan - roue.morgan@gmail.com

*Annals of Intensive Care* 2018, **8(Suppl 1):**P-195

**Introduction:** Type 1 myocardial infarction (MI) is an emergency, which immediate invasive strategy by a percutaneous coronary intervention (PCI) is based on guidelines for cardiologic patients. Conversely, the invasive strategy remains uncertain for patients hospitalized in the intensive care unit (ICU) for a primary non-cardiac disease with MI as a complication, given the ischemic and hemorrhagic risks. We aimed to assess the prevalence of—and describe the major adverse cardiac and hemorrhagic events occurring in the ICU after an invasive strategy by PCI in this context.

**Patients and methods:** We conducted a retrospective single-center 5-year (2012–2017) study. All the consecutive ICU patients with a suspected MI undergoing a coronarography were screened. Patients treated with an invasive strategy (PCI performed within 7 days of MI) were included. Patients hospitalized in ICU for cardiac disease were excluded. The major adverse cardiac events (MACE) were defined as post-procedure events occurring in the ICU, including death from cardiovascular causes, MI recurrence, need for emergent revascularization and stroke. The major adverse hemorrhagic events (MAHE) were defined as post-procedure events occurring in the ICU, according to the Bleeding Academic Research Consortium.

**Results:** 60 ICU patients suspected of MI underwent a coronarography. 29 patients (48%) had significant coronary lesions. Twelve patients were excluded—tri-truncular coronary involvement (n = 12), delayed procedure (n = 4), cardiogenic shock (n = 1). 17 patients were included (11 men, 70 years [IQR 25–75–68–77], 9 patients mechanically ventilated, 9 patients with sepsis septic shock, median SOFA Score at the time of MI 8 [3–10]). A PCI was performed during the first day after diagnosis of MI in 9 patients (53%) (median time—0 day [IQR 25–75–0–2]). A MACE occurred in 3 patients (18%), including stroke (n = 2) and MI recurrence without revascularization (n = 1). No patients deceased from cardiovascular causes in the ICU, neither at 6 months post-procedure (Table 1). A MAHE occurred in 3 patients (18%), 2 of whom had a MACE. Altogether, the prevalence of major adverse cardiovascular events combining MACE and MAHE was 0.24 (95% CI 0.01–0.46). There was no difference between septic and non septic patients regarding the prevalence of MACE or MAHE.

**Table 1:** Comparison of major adverse events in patients with sepsis or septic shock versus the others

Major adverse events (in the ICU)	Overall n = 17	Sepsis /septic shock n = 9	No Sepsis/septic shock n = 8	p
MACE				
Death from cardiovascular causes	0	0	0	–
Myocardial infarction recurrence	1 (6)	1 (11)	0	0.3
Emergent revascularization	0	0	0	–
Stroke	2 (12)	1 (11)	1 (12)	0.9
MAHE	3 (18)	2 (22)	1 (12)	0.6
Death from all causes	4 (23)	3 (33)	1 (12)	0.3

MACE: major adverse cardiac events; MAHE: major adverse hemorrhagic events

**Conclusion:** The prevalence of adverse cardiovascular events after an invasive strategy by PCI is high in non-cardiac critically ill patients with MI. Larger studies are needed to determine which patients may benefit from this procedure.

### P-196 Effects of Cyclosporine A on immune alteration after out-of-hospital cardiac arrest

#### Jahandiez Vincent^1^, Cour Martin^1^, Bochaton Thomas^2^, Monneret Guillaume^3^, Argaud Laurent^1^

##### ^1^Médecine Intensive Réanimation, Lyon, France; ^2^Laboratoire INSERM-U1060, Lyon, France; ^3^Hôpital Edouard Herriot, Lyon, France

###### **Correspondence:** Jahandiez Vincent - vincent.jahandiez01@chu-lyon.fr

*Annals of Intensive Care* 2018, **8(Suppl 1):**P-196

**Introduction:** Resuscitated cardiac arrest (CA) lead to immune alteration including lymphopenia, decreased monocyte HLA-DR (mHLA-DR) expression and dysregulated production of cytokines. In a recent multicenter randomized clinical trial, we tested the hypothesis that Cyclosprine A (CsA) would limit organ failures following out-of-hospital cardiac arrest (OHCA). In a substudy, we aimed to determine the influence of CsA on OHCA-induced immune dysfunction.

**Patients and methods:** This study is a predefined substudy of the randomized CYRUS trial (Cyclosporine in CA resuscitation). Patients with non-shockable OHCA randomly received either an intravenous bolus injection of CsA (2.5 mg kg) at the onset of advanced cardiovascular life support (CsA group) or no additional intervention (Control group). Patients from the coordinating center were sampled at admission (D0) and at 24 h (D1). Complete blood count, CD4 + lymphocytes count and mHLA-DR were evaluated by flow cytometry. Serum levels of IL-1, IL-6, IL-8, IL-10 and TNF&#945 + were measured by ELISA test on frozen samples.

**Results:** A total of 33 patients were sampled—17 patients from the CsA group and 16 from the Control group. The characteristics of the patients, including resuscitation data, were also similar between the two groups at admission. The severity of organ failure as assessed by the SOFA score at admission was similar between groups. All patients but one died before hospital discharge. At each follow-up time point (i.e. D0 and D1), we observed in both groups a similar increase in neutrophils and a marked decreased in mHLA DR as compared to normal values. At Day 1, total and CD4 + lymphocytes counts were significantly higher in CsA group as compared to Control group—1.3 [0.8–1.8] G l and 557 [385–874] cells µl versus 0.8 [0.4–1.2] G l and 233 [157–615] cells µl, respectively (p < 0.05). IL-1, IL-6, IL-8 and TNFα levels did not significantly differ between the two groups at D0 and D1 (p = ns). Interestingly, level of IL 10, a pleiotropic interleukin linked with lymphocytes response, was significantly lower at admission in CsA group compared to Control group (p < 0.05).

**Conclusion:** In patients presenting with nonshockable cardiac rhythm after OHCA, CsA administration prevented both CA induced lymphopenia and early systemic release of IL-10.

### P-197 Administration of testosterone gel in critical ill patients

#### Bachoumas Konstantinos^1^

##### ^1^CHU de Clermont-Ferrand, France

###### **Correspondence:** Bachoumas Konstantinos - cbachoumas@gmail.com

*Annals of Intensive Care* 2018, **8(Suppl 1):**P-197

**Introduction:** Critically ill patients experience major insults that lead to increased protein catabolism and a significant loss of lean body mass with an impact on weaning from the ventilator and muscle recovery. In critically ill patients, severe and persistent testosterone deficiency is very common after ICU admission. Administration of testosterone may induce skeletal muscle fiber hypertrophy and decreases protein breakdown. The aim of this work is to assess testosterone levels in critical ill patients and to evaluate the safety of testosterone gel administration.

**Patients and methods:** This is a single center study realized in a university ICU of 10 beds. Total testosterone levels were measured in critical ill men with at least one organ dysfunction with SOFA > 3. The study drug was Androgel, a formulation of 1% testosterone in an alcohol-water gel, approved by the ANSM for treatment of hypogonadism in men. AndroGel was applied to the abdomen, shoulders or upper arms once a day at the same time to dry and intact skin during ICU stay. The daily dose was 75 mg in men and 25 mg in women daily. Patients with history of prostate or breast cancer or PSA > 4 ng ml were excluded.

**Results:** Total testosterone levels were measured in 19 men. Median length of stay at the time of measurement was 7 days in ICU and 11 days in the hospital. Plasma testosterone levels were low in all but 1 patient. Median testosterone level was 58 ng dl (normal values 270–780 ng dl). Testosterone levels were not correlated with score SOFA or length of ICU stay. We found a moderate positif correlation between testosterone levels and length of hospital stay (r = 0.5 =). Testosterone gel was administered in 12 men and in 6 women. In these 18 patients, the median score SOFA was 10, ICU death occurred in 3 patients (22% ICU mortality), median length of ventilation was 13 days and median length of stay in ICU 30 days. All patients received mechanical ventilation and vasoactive treatment. 9 patients needed renal replacement therapy. AndroGel was well tolerated. No ischemic cardiovascular events were described. There was no application site reaction or acne. Median length of testosterone gel administration was 12 days.

**Conclusion:** Critical ill patients have low testosterone levels. Testosterone gel may be safely administered during the acute phase in ICU. Randomized clinical trials are needed to evaluate the impact of testosterone gel on physical performance.

### P-198 Clinical and epidemiological aspects of stroke at the University hospital of Oran Algeria

#### Goulmane Mourad^1^, Alachaher Djamel^1^, Djebli Houria^1^

##### ^1^CHU Benaouda Benzerdje, Oran, Algeria

###### **Correspondence:** Goulmane Mourad - m.goulmane@hotmail.com

*Annals of Intensive Care* 2018, **8(Suppl 1):**P-198

**Introduction:** Stroke is the leading cause of physical disability and the second leading cause of death worldwide. Two thirds of all strokes occur in developing countries and is increasingly a public health problem. The aim of this study was to evaluate the epidemiology of strokes in Oran, Algeria in order to create a stroke registry.

**Patients and methods:** A cross-sectional study was conducted on all patients admitted for stroke at the Oran CHU between January 2014 and September 2017. Sociodemographic data, modifiable and no-modifiable risk factors, type of stroke, degree of disability, Severity scores (GLASGOW and NIHSS) were studied. The SPSS 20 software, Log Rank test, was used for data analysis and statistical testing as well as kaplan–meier for survival studies.

**Results:** A total of 2305 stroke patients were enumerated, aged 20–99 years (mean ± SD = 68.33 ± 12.99), 74.2% had an ischemic stroke and 15% had a haemorrhagic stroke. 49% of the patients were men and 51% of the women. High blood pressure, diabetes, emboligenous heart disease and smoking were the most common risk factors. Intra-hospital mortality was 15.5% and the overall survival rate at 28 days was 77%.

**Conclusion:** This epidemiological study demonstrates that strokes at Oran Hospital may be similar to other locations. However, it seems necessary and useful to design a continuous patient registration system.

### P-199 Hyperosmolar states: prognosis and overview in a Moroccan intensive care unit

#### Ezzouine Hanane^1^, Benkhaldoun Mohammed^2^, Sghier Zineb^2^, Benslama Abdellatif^2^

##### ^1^CHU IBN Rchod, Casablanca, Morocco; ^2^University Hassan II, Casablanca, Morocco

###### **Correspondence:** Ezzouine Hanane - ezzouinehanane@yahoo.fr

*Annals of Intensive Care* 2018, **8(Suppl 1):**P-199

**Introduction:** The prevalence of hyperosmolar states and the relationship with mortality nevertheless remain unquantified and not objectively demonstrated. The aim of this work is to determine whether hyperosmolarity is a prognosis factor, and to assess the impact of hyperosmolarity on the evolution of patients.

**Patients and methods:** This is a retrospective descriptive and analytical study performed at the medical intensive care unit at the university teaching hospital IBN RUSHD in Casablanca on the cases admitted during 1 year. We noted epidemiological, clinical, biological and evolutionary parameters of all the patients and divided them into two groups according to their osmolar states, the first non-hyperosmolar group with plasma osmolarity of less than 320 mOsm L, called the control group and the second hyperosmolar group, plasma osmolarity greater than or equal to 320 mOsm L.

**Results:** 371 patients were included. The first group comprised 293 patients (79%) and the second comprised 78 patients (21%). The two groups did not differ significantly about sex and age. Hyperosmolar patients had more diabets 42.3%. Patients in the two groups did not show significant differences in clinical outcomes, including APACHE II and SAPS II scores. Significant differences are reported between the two groups, in natremia, creatinemia, liver transaminases. The plasma osmolarity was significantly different between the two groups with a mean in the control group of 269.77 ± 12.73 mol L while in the hyperosmolar group it was 346.95 ± 35.85 mosmol L (p = 0.046). The prevalence of hyperosmolar states in the study was 21% with 60% mortality. In the control group 41% were intubated-ventilated + 21.2% received vasoactive drugs and 68.6% received antibiotic therapy. In the control group + 7%of the patients were complicated by nosocomial infection, 9.9% by septic shock and 1% diseased by thromboembolic complications. The deceased subgroup used intubation artificial ventilation in 89.36%, vasoactive drugs in 68%, and antibiotic therapy in 89.36%. In the surviving subgroup, 6.45% only contracted the nosocomial infection. In the subgroup died 51.1% are of mixed hyperosmolar type + 82.4% hyperglycemic hyperuremic + 55.6% hyperglycemic hypernatremic type.

**Conclusion:** Hyperosmolar states are an independent a prognosis factor. Intubation and ventilation, vasoactive drugs and antibiotic therapy increases considerably in hyperosmolar states. Furthermore, it induced serious complications as nosocomial infections and septic shocks that further aggravate the prognosis even within hyperosmolar states.

### P-200 Incidence of exertional stroke in the Caribbean + clinical presentation and prognostic factors

#### Resiere Dabor^1^, Barret Morgane^2^, Florentin Jonathan^2^, Megarbane Bruno^3^, Mehdaoui Hossein^2^

##### ^1^Hôpital Pierre Zobda-Guitman, Fort-De-France, Martinique; ^2^CHU de la Martinique, Fort-De-France, Martinique; ^3^Hopital Lariboisière, Paris, France

###### **Correspondence:** Resiere Dabor - dabor.resiere@chu-martinique.fr

*Annals of Intensive Care* 2018, **8(Suppl 1):**P-200

**Introduction:** Hyperthermia represents a major life-threatening medical emergency, and is also one of the leading causes of death in young athletes worldwide. Its incidence is rare and little understood, but its mortality is on the rise. The objective of this study was to describe the population of patients admitted for exertional hyperthermia in Martinique and Guadeloupe and to determine the prognostic factors.

**Patients and methods:** Retrospective and prospective study, including all patients admitted for exertional hyperthermia in both emergency and resuscitation services in Martinique and Guadeloupe from January 2006 to June 2017. Results were expressed as mean ± SD or %.

**Results:** In 10 years, 55 patients were observed (age—79 ± 18, 44 men and 11 women), the main antecedents of which were—2 hypertension, 2 chronic OH, 2 psychoses, 1 stress hyperthermia. 10 (18%) of the patients had seizures initially. The pre-hospital management was < 30 min. Nevertheless, 20 (36%) patients were admitted to ICU due to organ failure (neurologic 74%, hemodynamic 2%, liver 4%). The progression was favorable, 4 deaths, including 3 fulminant hepatitis and multi-visceral failure. The average length of stay in intensive care units was 5 days (± 6).

**Conclusion:** Despite considerable preventive measures, stress hyperthermia represents a major problem within the military, soldiers and other athletes, with a mortality rate about 10% in most published series. The most effective method is immersion in ice water. There is an urgent need to provide the region with a clear preventive policy, including a relief action plan, training for doctors, athletes and other health professionals at risk of hyperthermia.

**Reference**

1. Nichols AW. Heat-related illness in sports and exercise. Curr Rev Musculoskelet Med. 21 sept 2014;7(4):355–65.

### P-201 Epidemiologic, comparative and prospective study on children population between Lille and Mayotte from 2016 to 2017

#### Chapoutot Anne-Gaëlle^1^, Leteurtre Stéphane^1^, Chamouine Abdourahim^2^

##### ^1^CHRU Lille, Lille, France; ^2^CH de Mayotte, Mamoudzou, France

###### **Correspondence:** Chapoutot Anne-Gaëlle - annegaelle.chapoutot@gmail.com

*Annals of Intensive Care* 2018, **8(Suppl 1):**P-201

**Introduction:** The setting up of intermediate care units (IteCUs) has been authorized in France since 2006. These units aim at receiving patients who need close monitoring, or present a risk of a potential organ failure without requiring substitution. The terms of the structural and staff organizations, clearly defined in 2006 by two Decrees and a Circular of application, have been described by Leclerc et al. (2008). The University Hospital of Lille is a pediatric center including several IteCUs in its pediatric hematology or gastrology departments, and more recently in its pediatric surgical department. Moreover, there are 4–6 IteCU extra-beds within the 12–14 bed pediatric intensive care unit (IveCU). The Hospital of Mayotte has no pediatric IveCU but a polyvalent one for adults, which receives children when necessary, as well as a 4 bed IteCU. The aim of this study was to describe prospectively the pediatric population which was admitted in the IteCUs of Lille and Mayotte over a one-year period from June 2016 to May 2017.

**Patients and methods:** In this twin-center, prospective and observational study, data were collected for each patient admitted during the test period in IteCUs of both Lille and Mayotte pediatric hospitals—general information about the patient, characteristics of each stay, severity scores on admission, type of treatments implemented, the report of the stay and patient’s evolution. A standard declaration was made with an authorization granted by the local Commission on Informatics and Liberty (French Commission Informatique et Liberté, CIL).

**Results:** During the course of the study, about 450 children were admitted in each center. The collected data allow to describe and compare both populations in terms of severity of each patient’s condition.

**Conclusion:** This study based on a very large cohort has permitted to compare the population of a regional hospital with that of a university hospital and to demonstrate that a health-care provision including a pediatric intensive care unit is needed on Mayotte Island.

### P-202 Accuracy of very low tidal volume delivery from ICU ventilators. A bench study with implications for ventilator setting in ARDS patients under ECMO

#### Moro Barbara^1^, Guerin Claude^1^, Baboi Loredana^1^, Yonis Hodane^1^, Lissonde Floriane^1^, Riad Zakaria^1^, Aublanc Mylène^1^, Louf-Durier Aurore^1^, Richard Jean-Christophe^1^

##### ^1^Hôpital de la Croix Rousse, Lyon, France

###### **Correspondence:** Moro Barbara - barbara.moro@chu-lyon.fr

*Annals of Intensive Care* 2018, **8(Suppl 1):**P-202

**Introduction:** In ARDS patients under ECMO common ventilator strategy aims at resting the lung by lowering tidal volume (VT) in the 1–4 ml kg predicted body weight range found in the literature analysis. We tested on the bench the not previously explored hypothesis that VT was not delivered in the 10% accuracy by most of ICU ventilators in this low range.

**Patients and methods:** Pneumatic test lung set at 20 ml/cmH2O compliance and 20 cm H_2_O/L/s resistance was attached to any of 5 ICU ventilators (V 500 (Drager), Carescape R 860 (GE Healthcare), Servo U (Maquet), PB980 (Covidien) and G5 (Hamilton)) equipped with Heated humidifier (Fisher-Paykel MR 850) set off and adult ventilator circuit (RT 380 EVAQUA Fisher Paykel). Each ICU ventilator was set in BTPS condition, at PEEP 12 cm H_2_O and FIO2 0.21. Airway pressure and airflow (Hans-Rudolph pneumotachograph) were measured (Biopac M150) proximal to the lung model. For each ventilator a series of VT ranging from 100 to 280 ml was delivered for 10 breaths each, at 30 then at 15 breaths/min respiratory rate (RR). The relationship of VT measured to VT set was assessed by linear regression over the 5 ICU ventilators for each circuit—RR combination. In each model, the change from the mean effect was assessed for each ventilator. For each model we obtained the mean effect of the 5 ventilators then we compared the effect of each ventilator to the mean effect.

**Results:** For each combination of f and circuit, the mean slope was significantly lower than 1 indicating that, on average, the set VT was under delivered (Table). There were differences in change in slope from the mean across the ventilators with interaction between ventilators and combinations. As an example, for the adult circuit f 15, Carestation, PB980 and Servo U performed better than G5 and V500. Across the combinations, V500 had consistent negative (greater underestimation than average) slopes and Servo U consistent positive (lower underestimation than average) slopes whilst the slope sign in the three others changed direction.

**Table:** Results of the four linear regression models

	Adult RR 15		Adult RR 30		Neonat RR 15		Neonat RR 30	
	Intercept (ml)	Slope (ml/ml)	Intercept (ml)	Slope (ml/ml)	Intercept (ml)	Slope (ml/ml)	Intercept (ml)	Slope (ml/ml)
Mean	0.77 [− 0.08;1.63]	0.85 [0.85;0.86]^†^	1.90 [0.91;2.89]*	0.85 [0.85;0.86]^†^	− 7.74 [− 8.63; − 6.85]*	0.83 [0.83;0.83]^†^	− 7.72 [− 8.43; − 7.01]*	0.83 [0.83;0.83]^†^
Carestation 860	2.23 [0.51;3.94]**	0.04 [0.03;0.05]^††^	5.64 [3.66;7.63]**	0.02 [0.01;0.03]^††^	5.77 [3.99;7.54]**	− 0.09 [− 0.10; − 0.08]^††^	5.98 [4.56;7.41]**	− 0.08 [− 0.09; − 0.07]^††^
G5	3.82 [2.11;5.53]**	− 0.08 [− 0.08;0.00]^††^	8.12 [6.14;10.11]**	− 0.09 [− 0.10; − 0.08]^††^	− 6.94 [− 8.71; − 5.16]**	0.05 [0.04;0.06]^††^	− 7.12 [− 8.54; − 5.70]**	0.04 [0.04;0.05]^††^
PB980	− 5.70 [− 7.41; 3.99]**	0.01 [0.01;0.00]	− 8.77 [− 10.76; − 6.78]**	− 0.02 [− 0.03; − 0.01]^††^	0.74 [− 1.04;2.51]	0.04 [0.04;0.05]^††^	− 0.37 [− 1.80;1.05]	0.04 [0.04;0.05]^††^
Servo-U	3.63 [1.92;5.34]**	0.04 [0.04;0.00]^††^	3.32 [1.33;5.31]**	0.07 [0.06;0.08]^††^	6.61 [4.83;8.39]**	0.01 [0.00;0.02]^††^	7.57 [6.15;8.99]**	0.01 [0.00;0.02]^††^
V500	− 3.98 [− 5.70; − 2.27]**	− 0.02 [− 0.03; − 0.01]^††^	− 8.32 [− 10.30; − 6.33]**	− 0.01 [− 0.00;0.02]	− 6.17 [− 7.95; − 4.40]**	− 0.02 [− 0.03; − 0.01]^††^	− 6.06 [− 7.49; − 4.64]**	− 0.02 [− 0.03; − 0.01]^††^

*p < 0.05 versus. 0 ** p < 0.05 versus. Mean intercept ^†^p < 0.05 versus. 1 ^††^p < 0.05 versus. Mean slope

**Conclusion:** Very low VT were properly delivered by common ICU ventilators on this present bench study.

### P-203 Respiratory mechanic using a simulator of artificial ventilation in virtual ARDS patients with the protocols of the ARMA and Express studies

#### Roze Hadrien^1^, Dubois Rémi^1^

##### ^1^IHU LIRYC, Bordeaux, France

###### **Correspondence:** Roze Hadrien - hadrien.roze@chu-bordeaux.fr

*Annals of Intensive Care* 2018, **8(Suppl 1):**P-203

**Introduction:** Simulation in intensive care is an innovative method for teaching. Respiratory settings are responsible for some morbi-mortality of our patients. For this reason we develop a simulator of artificial ventilation (SimVA) and virtual patients. Mathematical model resolved differential equations of chest and lung movements in order to match with a clinical data base. The goal of this study was to evaluate and compare virtual patients respiratory mechanic with the results of 4 different protocols of ventilation from 2 large randomised controlled trial—Arma (1) and Express (2).

**Patients and methods:** Virtual patients had ARDS, and were defined by different thoracic and pulmonar compliance, total resistance, lung volumes, pressure–volume relation, and pressure and volume recruitment coefficients. Ventilatory protocols were High versus Low VT (Arma study) and Max versus Min distension according to PEP (Express study). Each virtual patient was titrated on the simulator with the 4 protocols. Respiratory frequency was set around 28 cycles minute and adapted to protocols. Respiratory mechanic after titration was recorded and compared to results of the 2 studies.

**Results:** Results are summarised in the table—the difference between virtual and real patients were not significant.

**Table:** 

Protocols	Arma high V_T_		Arma low V_T_		Express distension min		Express distension max	
Patients	Real	Virtual	Real	Virtual	Real	Virtual	Real	Virtual
V_T_, ml	11.8 (6.2)	12 (0)	6.2 (0.9)	6 (0)	6.1 (0.4)	6 (0)	6.1 (0.3)	6 (0)
Pplat cmH_2_O	33 (9)	34.1 (3.6)	25.0 (7.0)	24.0 (2.0)	21.1 (4.7)	22 (0.8)	27.5 (2.4)	28 (0.2)
PEP cmH_2_O	8.6 (3.6)	7.9 (0.7)	9.4 (3.6)	9.0 (0)	7.1 (1.8)	7.1 (0.8)	14.6 (3.2)	14.7 (4.3)
PEPtot cmH_2_O	Na	8.5 (0.9)	Na	10.0 (1.0)	8.4 (1.9)	8 (0.8)	15.8 (2.9)	15.7 (4.4)
RF cycle/min	16.0 (6.0)	14.5 (1.0)	29.0 (7.0)	28.0 (1.0)	27.8 (5.4)	28.1 (1.2)	28.2 (5.4)	28.1 (1.2)
Vm L/min	12.6 (4.5)	13.3 (1.7)	12.9 (3.6)	13.0 (2.0)	11.2 (2.8)	13.2 (1.3)	11.3 (2.7)	13.2 (1.8)
Ctp ml/cmH_2_O	Na	33.7 (3.4)	Na	32.0 (4.0)	33.7 (14.3)	31 (1.6)	37.2 (22.7)	36.2 (7.9)

Ctp: tharacopulmanar compliance (ml/cmH_2_O)

**Discussion:** Inspiratory plateau pressure and thoraco-pulmonary compliance were able to change according to PEP or VT settings within the same range as the 2 large RCT studies. Mathematical model of recruitment was adapted to create many different results while PEP was titrated according to respiratory mechanics with the Express protocol.

**Conclusion:** Simulation of artificial ventilation with a software can be realistic and might be an interesting pedagogical tool to teach interactively and repetidly ventilatory settings and respiratory mechanics interactions in ARDS without any risk for the patient in our units.1. N Engl J Med. 2000 + 342-1301-82. JAMA. 2008 + 299-646-655.

### P-204 Tidal expiratory flow limitation in ARDS patients. A reassessment

#### Yonis Hodane^1^, Baboi Loredana^1^, Riad Zakaria^1^, Lissonde Floriane^1^, Louf-Durier Aurore^1^, Aublanc Mylene^1^, Mezidi Mehdi^1^, Richard Jean-Christophe^1^, Guerin Claude^1^

##### ^1^Hôpital de la Croix Rousse, Lyon, France

###### **Correspondence:** Yonis Hodane - Hodane.yonis@chu-lyon.fr

*Annals of Intensive Care* 2018, **8(Suppl 1):**P-204

**Introduction:** Expiratory flow limitation (EFL) has previously been investigated in ARDS patients on zero PEEP by using negative expiratory pressure (NEP) technique on tidal breath. In ARDS patients with EFL PEEP 10 improved oxygenation from intrinsic PEEP homogenization rather than lung recruitment. The NEP technique is no longer available. As EFL should reflect airway closure it is important to assess it. We described a new technique to assess EFL.

**Patients and methods:** Thirty-nine ARDS patients (6 mild, 32 moderate, 1 severe) were investigated at PEEP5 and15. They were intubated, mechanically ventilated (Evita XL) in volume controlled mode (tidal volume 5 ± 1 ml kg predicted body weight) in the semi-recumbent position. Airway pressure and flow measured proximal the endotracheal tube were continuously recorded (Biopac150). We measured respiratory mechanics by the occlusion technique at each PEEP and recruited lung volume between PEEP15 and 5 by using low flow inflation method associated with measurement in change in end-expiratory lung volume. For the latter, patient was manually disconnected at the end of baseline tidal inflation downstream pneumotachograph to atmosphere til zero flow, then reconnected at previous settings. EFL was assessed offline by superimposing flow-volume loops of disconnected and baseline breath. EFL was defined if no change in flow occurred over all or part of the disconnected expiration as compared to the baseline breath and no EFL (NFL) if any increase in flow during the expiration was present (Fig. 1). The percentage of the tidal volume involved in EFL was measured.

**Fig. 1:**
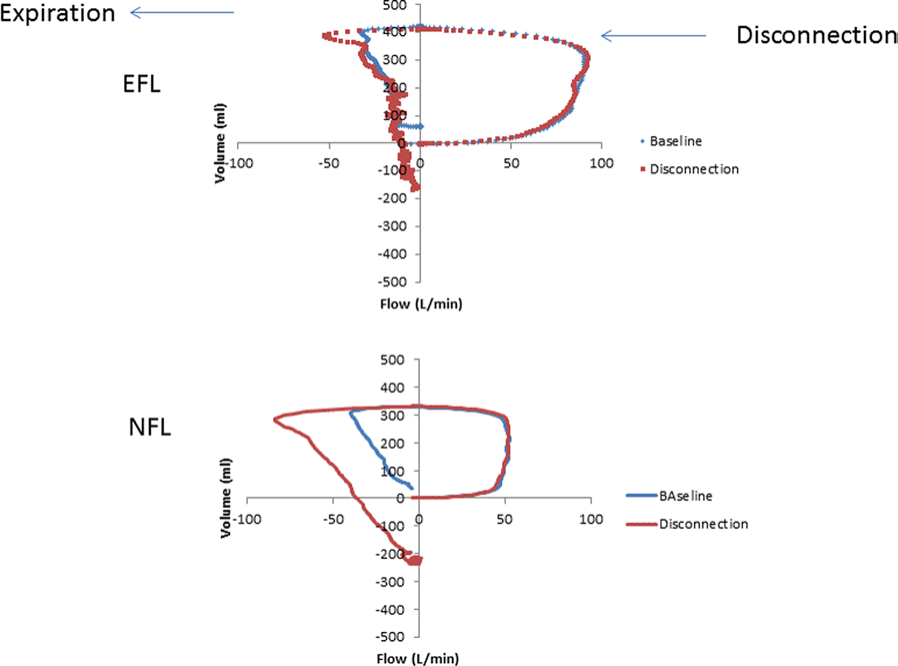
The flow signal during disconnection in case of expiratory flow limitation (at the top) or no expiratory flow limitation (at the bottom)

**Results:** EFL was present in 7 patients (18%) over 80% of the tidal expiration. Patients with EFL had significant higher body mass index (34 ± 7 vs. 28 ± 6 kg m^2^, p < 0.05) and totalPEEP at PEEP5 (7 ± 1 vs. 6 ± 1cmH2O, p < 0.05) than NFL patients and tended to be more hypoxemic. At PEEP15 EFL patients had a significant better compliance (50 ± 21 vs. 30 ± 12 ml cm H_2_O, p < 0.05) with no change in recruited lung volume (393 ± 271 vs. 347 ± 203 ml) and tended to be more hypoxemic than NFL patients. Mortality at ICU discharge was 57% in EFL versus 28% in NFL (p = 0.19).

**Conclusion:** Measurement of EFL is feasible without the NEP technique. At higher PEEP ARDS patients with EFL markedly improved compliance of the respiratory system not related to lung recruitment. Further studies are required to better understand EFL in ARDS patients and to assess its impact on patient outcome.

### P-205 Functional and phenotypic alterations of monocytes in pneumonia-related acute respiratory distress syndrome

#### Bendib Le Lan Ines^1^, Schlemmer Frederic^1^, Maitre Bernard^1^, Ferchiou Asma^1^, Hüe Sophie^1^, Carteaux Guillaume^1^, Razazi Keyvan^1^, Mekontso Dessap Armand^1^, Godot Véronique^1^, De Prost Nicolas^1^

##### ^1^CHU Henri Mondor, Assistance Publique-Hôpitaux de Paris, Créteil, France

###### **Correspondence:** Bendib Le Lan Ines - ines.bendib@aphp.fr

*Annals of Intensive Care* 2018, **8(Suppl 1):**P-205

**Introduction:** Sepsis-induced immune alterations encompass a wide range of immune cells alterations. Among these defects, a decreased HLA-DR expression on circulating monocytes was associated with a higher risk of nosocomial infections and higher mortality. Such immune alterations have also been observed in lung cells obtained from deceased patients. We aimed to compare phenotypic and functional characteristics of monocytes from ARDS and controls in blood and lung compartments.

**Patients and methods:** This is a prospective single center study. Patients with pneumonia and moderate severe ARDS admitted to the ICU (February-May 2017) were included. Immunosuppressed patients were excluded. The control group consisted in ARDS-free patients having no lung infection or immunosuppression status. Monocytes from peripheral blood and broncho-alveolar lavage (BAL) fluid were collected within 48 h of intubation and phenotyped (HLA-DR and PD-L1 expressions). Phagocytosis and TNFa intracellular synthesis, after *E. coli* lipopolysaccharide priming, were assessed in vitro. All analyzes were performed using flow cytometry. Results were expressed as positive cells ratio and mean florescence intensity.

**Results:** Six ARDS patients and five controls were included. All patient had septic shock upon ICU admission. SAPS II and SOFA scores were 61 [44–71] and 12 [9–14], the PaO2 FiO_2_ ratio was 97 [67–125] mmHg. Five patients had a bacterial pneumonia and one had a viral pneumonia. HLA-DR and PD-L1 expressions were higher on alveolar than on blood monocytes in both ARDS patients and controls (Figure). Yet, HLA-DR expression on alveolar monocytes was higher in controls compared to ARDS patients (p = 0.004). Circulating monocytes had a higher phagocytic activity than alveolar monocytes (p < 0.001), but no significant difference was observed between ARDS patients and controls. An LPS challenge increased the phagocytic activity of monocytes in controls (p = 0.03) but not in ARDS monocytes (p = 0.88). TNF-α intracellular synthesis was increased after LPS exposure in circulating and alveolar monocytes of controls (p < 0.05) but only tended to do so in ARDS (p = 0.08).
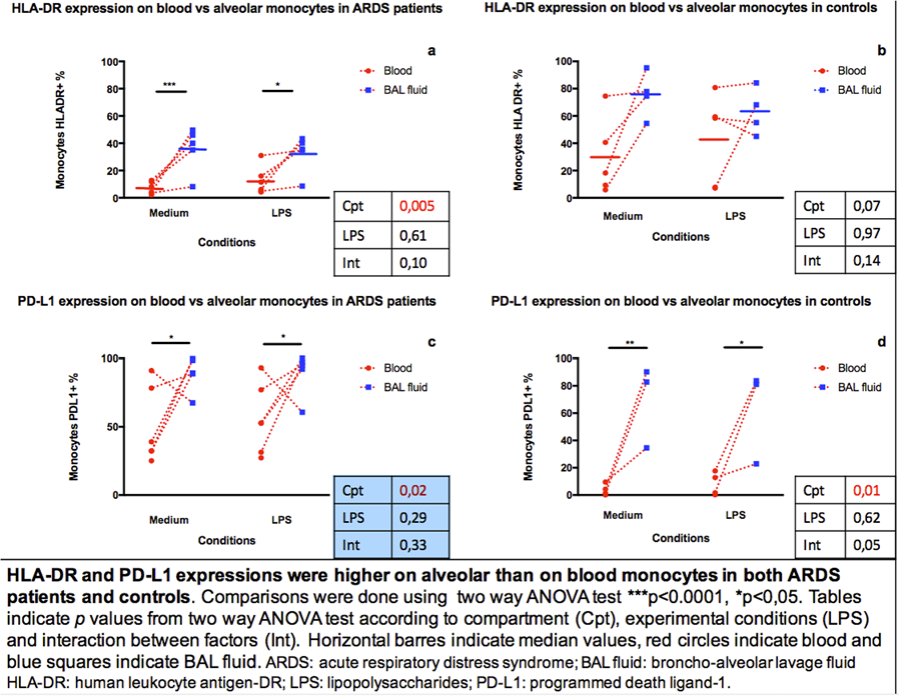


**Conclusion:** Differences in the phenotype of alveolar and circulating monocytes were observed in ARDS but also in controls, suggesting a physiological lung blood gradient in the expression of these biomarkers. Yet, HLA-DR expression on alveolar monocytes was lower in ARDS than in controls, consistent with sepsis-induced immunosuppression at the alveolar level. Functional differences observed between ARDS and controls suggested a tolerogenic profile of ARDS monocytes.

### P-206 Comparison of two strategies of oropharyngeal and tracheal suctioning in mechanically ventilated patients (AMYLASPI): a pilot study

#### Griton Marion^1^, Bedel Aurélie^1^, Clouzeau Benjamin^1^, Vargas Frederic^1^, Hilbert Gilles^1^, Gruson Didier^1^, Boyer Alexandre^1^

##### ^1^CHU Bordeaux, Bordeaux, France

###### **Correspondence:** Griton Marion - marion.griton@chu-bordeaux.fr

*Annals of Intensive Care* 2018, **8(Suppl 1):**P-206

**Introduction:** Despite their recommendation in the prevention of ventilator-associated pneumonia, oral care is not still clearly standardized. It generally includes a time for oropharyngeal and tracheal suctions which can induce a cough reflex in non-paralyzed patients leading to the mobilization of the endotracheal tube and a consecutively increased risk of tracheal microaspirations. During the oral care procedure, drainage of subglottic secretions at particular times before oro-tracheal suctions is expected to reduce microaspiration. The aim of this study is to assess whether this “optimized” oral care including subglottic drainage can reduce microaspirations.

**Patients and methods:** This is an open prospective study, including ICU ventilated patients. Two procedures have been compared in two randomized cross-over consecutive periods of one day each (3 oral cares a day)—on 1 day, they received routine oral care (oral care (O) then tracheal suction (T)) and on the other day they received optimized oral care (subglottic suction (SG1) then O then SG2 then T). The amylase enzymatic activity has been measured in O, T, SG1 and SG2 suctions as a surrogate for the oropharyngeal content. If present in T suctions, it defines microaspiration. Since the amylase O content is not similar from a patient to another, the primary outcome was the median amylaseT O ratio after routine versus optimized oral care.

**Results:** After informed consent, 24 patients were included. 21 were analyzed due to incomplete follow-up in 3 patients. Patients (SAPSII 51 ± 16) were ventilated since 4.5 ± 4.3 days for a majority of respiratory indications. At day 1, 10 and 11 patients received routine oral or optimized oral car respectively without significant baseline difference. A trend in the reduction (− 35%) of Amylase T O median ratio was observed after optimized versus routine oral care (2.3% [0.6–6 vs. 1.5% [0.7–16], p = 0.16.

**Conclusion:** Despite protection of trachea by the cuff of the endotracheal tube, amylase has been found in tracheal suctions (which represents the last step of oral care). In this pilot study with a limited sample of patients, a trend in the reduction of microaspirations was observed when subglottic suctions were interleaved between oral and tracheal suctions. An increased sample power could show more significant results, but we cannot eliminate that this weak effect could also be due to the inability of subglottic suctions to prevent microaspiration of the oral content. The study has been founded by Teleflex.

### P-207 Safety and feasibility of extra corporeal carbon dioxide removal (ECCO2R) in ARDS and non ARDS patients

#### Monet Clément^1^, Monnin Marion^1^, De Jong Audrey^1^, Prades Albert^1^, Conseil Matthieu^1^, Chanques Gerald^1^, Carr Julie^1^, Belafia Fouad^1^, Jaber Samir^1^

##### ^1^Hôpital Saint Eloi, Montpellier, France

###### **Correspondence:** Monet Clément - clemen.t@hotmail.fr

*Annals of Intensive Care* 2018, **8(Suppl 1):**P-207

**Introduction:** Although necessary, mechanical ventilation can lead to ventilator-induced lung injury (VILI) even when using protective ventilation strategies that combine low tidal volume (Vt)(6 ml kg predicted body weight) and plateau pressure (Pplat) <= 30cmH20. Lower positive pressures and tidal volumes could enhance lung protection + the limiting factor being carbon dioxide accumulation and hypercapnic acidosis. Extra corporeal carbon dioxide removal (ECCO2R) intervenes by maintaining ph and PCO2 within physiological ranges. This combination is called ultra-protective ventilation. We report our experience with ECCO2R in ARDS and non ARDS patients with a focus on feasibility and safety.

**Patients and methods:** From June 2014 to July 2017 all patients who have undergone ECCO2R in our ICU were included consecutively and prospectively. Venovenous ECCO2R was used through a dual lumen venous catheter (femoral or jugular).

**Results:** Nineteen patients underwent ECCO2R for a total of 21 sessions. ECCO2R was implemented through a dual lumen venous catheter (femoral or jugular) with 3 different devices—Hemolung Respiratory Assist System^®^ (ALung) (n = 2), iLA activve^®^ (Novalung) (n = 5) and Prismalung^®^ (Prismaflex system) (n = 12). Sessions were 3 (IQR 2.5–4.0) days long. Catheter diameters were 13 Fr (n = 8), 15 Fr (n = 6), 24 Fr (n = 4) and 17 Fr (n = 1). Thirteen patients suffered from ARDS and 6 had non ARDS indications for ECCO2R, including ultra-protective ventilation. Tidal volume decreased during ECCO2R from 5.3 (IQR 4.3–5.8) to 3.4 (IQR 2.6–4.1) ml kg of predicted body weight (p < .001) while ECCO2R allowed maintaining of pH and PCO2 within acceptable range (Fig. 1). Driving pressure decreased from 17 (IQR 11–21) to 8 (IQR 6–14) cm H_2_O (p < .001). The main adverse effect was thrombocytopenia (8 patients). Six selected patients had no anticoagulation during ECCO2R because of high bleeding risk.

**Fig. 1:**
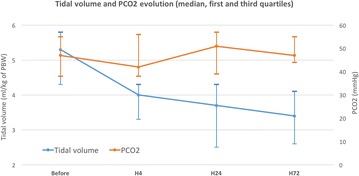
Evolution of tidal volume (blue line) and PaCO_2_ (orange line) over the time

**Discussion:** Ultra-protective ventilation was achieved with a decrease of tidal volumes (Vt < 4 ml kg) and positive pressures. Few data on ECCO2R are available in patients at high risk of hemorrhagic complications, we report here a subgroup of 6 patients who underwent efficiently ECCO2R without anticoagulation. Six patients underwent ECCO2R for non ARDS indications, 3 of them had no structural damages to the lungs which has never been reported and ECCOR allowed implementing ultra-protective ventilation with no major adverse effect.

**Conclusion:** We report our experience on ECCO2R for ARDS and non ARDS indications. Ultra-protective ventilation (Vt < 4 ml kg) was safe and feasible.

### P-208 The impact of general practitioners consultation on ARDS complicating community acquired pneumonia

#### Donval Ulysse^1^, Tadie Jean-Marc^1^, Maamar Adel^1^, Berneau Pauline^1^, Benezit François^1^, Le Tulzo Yves^1^, Gacouin Arnaud^1^

##### ^1^Bloc Hôpital, Rennes, France

###### **Correspondence:** Donval Ulysse - ulysse.donval@gmail.com

*Annals of Intensive Care* 2018, **8(Suppl 1):**P-208

**Introduction:** Community-acquired pneumonia (CAP) is a potentially severe infection that results in numerous general practitioner (GP) visits and hospital admissions each year. CAP is also the most frequent single cause of acute respiratory distress syndrome (ARDS). Risk factors for development of ARDS in the course of CAP are not clearly defined although prognostic factors associated with mortality have been extensively studied. GP visits, as an early diagnosis and earlier access to antibiotics prescription could significantly affect the course of CAP. The aim of the present study was to evaluate the impact of general practitioners consultation on ARDS complicating CAP admitted to our ICU.

**Patients and methods:** We retrospectively reviewed the medical records of all patients aged over 18 years admitted between October 1, 2006 and December 31, 2015, for ARDS complicating community acquired pneumonia with a PaO2 FiO_2_ ratio < 200 mmHg after at least 12 h of lung protective mechanical ventilation (MV). Ventilatory modalities for ARDS had been protocolized over the study period as our ICU was recruiting patients for two consecutive multicenter trials (Acurasys and Proseva). Consequently, the protective ventilatory strategy used in these two clinical trials was applied to every patient with ARDS. Patients were divided into two groups according to whether or not they visited a GP before ICU admission.

**Results:** 216 patients were admitted for ARDS complicating CAP. 114 patients (53%) had visited a GP before admission in ICU (GP +) and 102 did not (GP-). Analysis of demographic data, respiratory microbiology patterns, ARDS severity at admission did not show any differences between the two groups. SOFA score at admission was significantly higher in GP—compared to GP + patients (9.5 (8–13) vs. 8.5 (7–10) respectively + p = 0.003) although respiratory SOFA scores were not different (4 (3–4) vs. 4 (3–4) respectively + p = 0.31). 71 (70%) GP—(70%) and 60 (53%) GP + patients presented septic shock at ICU admission (p = 0.01). Multivariate analysis found that GP consultation (0.54 [0.29–0.98] + p = 0.04) with antibiotics prescription (0.46 [0.22–0.99] + p = 0.04) were associated with decreased mortality at day 28 (Fig. 1).

**Fig. 1:**
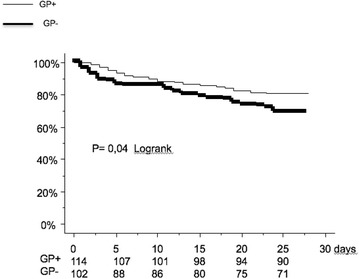
Kaplan–Meier survival analysis of 28-day survival according to whether or not ARDS patients visited a GP before ICU admission. p = 0.04, log-rank test

**Conclusion:** In patients admitted to our ICU for ARDS complicating community acquired pneumonia, GP visits prior to ICU admission was associated with a better outcome. The beneficial effect may be due to earlier antibiotic prescription which could significantly lowered severe infection and septic shock.

### P-209 Alveolar recruitment during ards—monitoring the end-expiratory lung volume by CRF inview^®^

#### Hajjej Zied^1^, Sellami Walid^1^, Cherni Hayet^1^, Yengui Olfa^1^, Bousselmi Radhouene^1^, Labbene Iheb^1^, Ferjani Mustapha^1^

##### ^1^Hôpital Militaire de Tunis, Tunisia

###### **Correspondence:** Hajjej Zied - hajjej_zied@hotmail.com

*Annals of Intensive Care* 2018, **8(Suppl 1):**P-209

**Introduction:** Optimal PEEP level during ARDS remains controversial because of its beneficial and adverse effects. The optimal level of recruitment and its effect on oxygenation are not well defined and no technique is currently validated. The aim of our study was to evaluate the correlation between the recruited pulmonary volume estimated by a new technique (CRF INview^®^) and the evolution of PaO2 as well as the respiratory and hemodynamic tolerance of the application of an increasing levels of PEEP .

**Patients and methods:** A prospective, monocentric study that will last 3 years (January 2015-January 2018), taking place in the intensive care unit at the military teaching hospital of Tunis and including patients if they met standard criteria for ARDS (Berlin criteria). The main criterion for judgment was the correlation between the recruited pulmonary volume estimated by a new technique (CRF INview^®^) and the evolution of the PaO2 after application of three increasing levels of PEEP (5–10–15). The other secondary criteria were the respiratory and hemodynamic tolerance of the application of increasing levels of PEEP measured by the PiCCO^®^ technique.

**Results:** Until 31 03 2016, ten patients were included and analyzed for the study. There was a significant difference between the volumes recruited at the three PEEP levels (p = 0.002). The recruitment evaluated was not correlated with PaO2. There is a significant decrease in cardiac index and PAM caused by the increase in PEEP.

**Conclusion:** Preliminary results from our study suggest that the estimated recruited lung volume estimated by CRF INview^®^ technology appears to be poorly correlated with measured PaO2. The hemodynamic repercussions observed should also be considered in order to propose an optimal strategy for the optimal adjustment of PEEP.

### P-210 Evaluation of carbapenems prescriptions in a medical intensive care unit

#### Jeannin Benedicte^1^, Pajot Olivier^1^, Contou Damien^1^, Thirion Marina^1^, Tirolien Joanna^1^, Zylberfajn Cecile^1^, Mentec Herve^1^, Plantefeve Gaetan^2^

##### ^1^Hôpital Antoine Beclere, Paris, France; ^2^Hôpital Victor Dupouy, Argenteuil, France

###### **Correspondence:** Jeannin Benedicte - bene.jeannin@hotmail.fr

*Annals of Intensive Care* 2018, **8(Suppl 1):**P-210

**Introduction:** The use of carbapenems increases in France. Their use in French hospitals tripled between 2000 and 2015. This use increases the risk of emergence of new bacterial resistance, potentially leading to therapeutic impasses. The main objective of this study is to evaluate the compliance of carbapenems prescriptions in a medical intensive care unit (ICU). The secondary objective is to evaluate the impact of these prescriptions about outcome.

**Patients and methods:** This non-interventional, retrospective, monocentric study includes all patients who received carbapenems in a medical ICU between January 2011 and December 2015. We excluded patients with more than 20% missing data. The carbapenem requirements were classified according to the recommendations of the Society of Infectious Pathology of French Language, according to their compliance, by targeting the indication (criteria A) and the reassessment at 48–72 h or on receipt of antibiogram (criteria B).

**Results:** 3236 patients were admitted in ICU during the period. 1633 patients received antibiotics, of whom 226 received carbapenems. We analyzed 270 prescriptions of carbapenems (248 prescriptions of imipenem and 22 prescriptions of meropenem). 238 (88%) prescriptions were compliant with the indication of carbapenems and 239 (89%) were compliant with the re-evaluation. 191 (85%) patients received carbapenems according to the recommendations. A compliant prescription had no impact on hospital or ICU length of stay and no impact on duration of mechanical ventilation but seemed associated with increase mortality (p = 0.04).

**Discussion:** The high rate of compliant prescriptions can be explained by the broad indications of carbapenems in the ICU, especially in patients with septic shock. The increase mortality of patients with a compliant prescription is probably due to the severity of the infections. In order to achieve 100% compliance, we could suggested regularly updating the knowledge of carbapenems prescriptions, collaborating with bacteriology and infectiology teams, and establishing a computerized or paper prescription with feedback control.

**Conclusion:** The prescription of carbapenems appears most often in accordance with the recommendations in this ICU. However, there is a need for improvement.

### P-211 Early sepsis markers in diabetic ketoacidosis—leukocytes, neutrophils-to-lymphocytes count ratio, temperature or procalcitonin? A retrospective study

#### Blanchard Florian^1^, Van Der Meersch Guillaume^1^, Charbit Judith^1^, Popoff Benjamin^2^, Cohen Régis^3^, Bonnet Nicolas^1^, Ahmed Pasem^1^, Bihan Hélène^1^, Cohen Yves^1^

##### ^1^CHU Avicenne, Bobigny, France; ^2^CHU Rouen, Paris, France; ^3^CHU Delafontaine, St Denis, Paris, France

###### **Correspondence:** Blanchard Florian - fblancha@gmail.com

*Annals of Intensive Care* 2018, **8(Suppl 1):**P-211

**Introduction:** Bacterial infections are frequent triggers for diabetic ketoacidosis and a significant increase in morbimortality is observed in case of delayed antibiotic treatment. However the unnecessary administration of antimicrobial therapy can also lead to bacterial resistance. Early sepsis markers are thus particularly useful for patients admitted in ICU for diabetic ketoacidosis.

**Patients and methods:** We retrospectively studied cases of patients admitted in ICU at Avicenne French Universitary hospital for ketoacidosis defined by pH < 7.3 and glycemia > 13.75 mmol L. Clinical and biological data were analyzed at admission (D0) and on day 2 (D2).

**Results:** Between 2010 and 2016, among 93 patients admitted for diabetic ketoacidosis, 69 were included. Twelve out of 69 were infected (5 urosepsis, 5 pneumonia, 2 others). Demographic data and comorbidities did not significantly differ between the infected and non infected group (IG and NIG). Antibiotics were administered to 33 patients—12 12 (100%) in the infected group versus 21 57 (36.8%) in the non infected group. On D0, there was no difference for—pH, temperature, leukocytes, neutrophils-to-lymphocytes count ratio and PCT (Table 1). On D2, temperature, leukocytes, neutrophils-to-lymphocytes count ratio and PCT were significantly higher in the IG. In the IG, the biological markers did not vary between D0 and D2, whereas in the NIG, leukocytes (p < 0.0001), PNN (p < 0.0001) and neutrophils-to-lymphocytes count ratio (p < 0.0001) significantly decreased. Surprisingly average PCT levels seem to be particularly high in the NIG on DO as well as on D2.

**Table 1:** Infected and non infected patients at admission (D0) and day 2 (D2)

Variable:mean (range)	D0		IG vs NIG on D0	D2		IG vs NIG in D2
	Infected patients (n = 12)	Non infected patients (n =57)	p value*	Infected patients (n = 12)	Non-infected patients n = (57)	p value*
pH	7.06 [6.95–7.17]	7.10 [7.07–7.13]	0.48	7.37 [7.31–7.43]	7.41 [7,40–7,43]	0.18
Temperature, °C	36.9 [30.0–39.9]	35.9 [32.9–38.7]	0.67	38.2 [36.8–39.0]	37.1 [35.5 –38.5]	0.002
Leukacytes, G/L	17.8 [6.9–30.3]	19.2 [4.2–57.0]	0.57	14.3 [7.1–31.5]	8.8 [3.0–17.7]	0.016
Neutrophils-to-lymphocyts ratio	12.6 [2.1–31.1]	20.0 [0.9–129.3]	0.08	18.0 [4.4–42.2]	5.32 [0.4–21.1]	0.004
Neutrophils polynuclears, G/L	15.02 [6.07–26.36]	16.28 [2.65–44.46]	0.56	12.05 [6.75–24.57]	6.29 [1.12–16.46]	0.003
Procalitonine, µg/L	23.62 [0.64–141.68]	1.51 [0.04–17.01]	0.11	26.95 [0.63–116.00]	0.89 [0.02–4.68]	0.033

Values of p < 0.05 are statistically significant

**Conclusion:** At admission, PCT as well as other usual markers do not appear to be useful to differentiate infected from non infected patients admitted for ketoacidosis. However, on day 2, two different patterns can be drawn and help detecting non-infected patients and thus reduce exposure to antibiotics. These results should be confirmed by a prospective study, including a larger number of patients.

### P-212 Are ventilator associated events relevant for the detection of ventilator associated lower respiratory tract infections?

#### Pouly Olivier^1^, Rouze Anahita^2^, Voisin Benoit^2^, Poissy Julien^2^, Wallet Frederic^2^, Nseir Saad^2^

##### ^1^Ronchin, France; ^2^CHRU Lille, Lille, France

###### **Correspondence:** Pouly Olivier - olivier.pouly@hotmail.fr

*Annals of Intensive Care* 2018, **8(Suppl 1):**P-212

**Introduction:** In 2013, The Centers for Disease Control and Prevention (CDC) introduced a new surveillance paradigm for ventilated patients. Ventilator-associated events (VAE), reflecting worsening oxygenation, are defined as a persistent and significant increase in FiO_2_ or PEEP level after a period of stability on the ventilator. VAE definition includes ventilator-associated conditions (VAC), infection-related ventilator-associated complications (IVAC) and probable ventilator-associated pneumonia (PVAP). The relevance of VAE for ventilator-associated pneumonia (VAP) is low. However, the correlation between the three VAC, IVAC, and PVAP, and the onset of ventilator-associated low respiratory tract infection (VALRTI), including ventilator-associated tracheobronchitis (VAT) and pneumonia (VAP), has never been studied yet. We aimed to investigate the concordance between the onset of three VAE tiers and VALRTI, and their impact on outcomes.

**Patients and methods:** We performed a retrospective analysis of prospectively collected data from patients requiring mechanical ventilation for more than 5 days in a 50-bed mixed ICU of a tertiary university teaching hospital, between January 1 and December 31, 2016. VAT and VAP episodes were assessed by prospective surveillance of nosocomial infections, according to the American Thoracic Society criteria. VAE were identified retrospectively, according to current CDC definitions. The agreement between VAC, IVAC, PVAP and VALRTI was assessed by k statistic. The impact of VAE and VALRTI on duration of mechanical ventilation, ICU and hospital length of stay and mortality was also assessed for the first episode of VAT and VAP.

**Results:** We included 545 patients (7927 ventilator days). 126 VAP (15.9 per 1000 ventilator-days), 80 VAT (10.1 per 1000 ventilator-days) and 115 VAE (14.5 per 1000 ventilator-days) were diagnosed. There was no agreement between VAT and VAE and the agreement was poor between VAP and VAC (k = 0.12, 95% CI 0.03–0.20), VAP and IVAC (k = 0.23, 95% CI 0.14–0.32) or VAP and PVAP (k = 0.30, 95% CI 0.22–0.40). Patients who developed VAT, VAP or VAE had significantly longer duration of mechanical ventilation, ICU and hospital length of stay, compared to patients who did not, with similar mortality rates.

**Conclusion:** VAE are not relevant for VAT diagnosis and have low agreement with VAP, despite their negative impact on ventilation duration, ICU and hospital length of stay (Fig. 1).

**Fig. 1:**
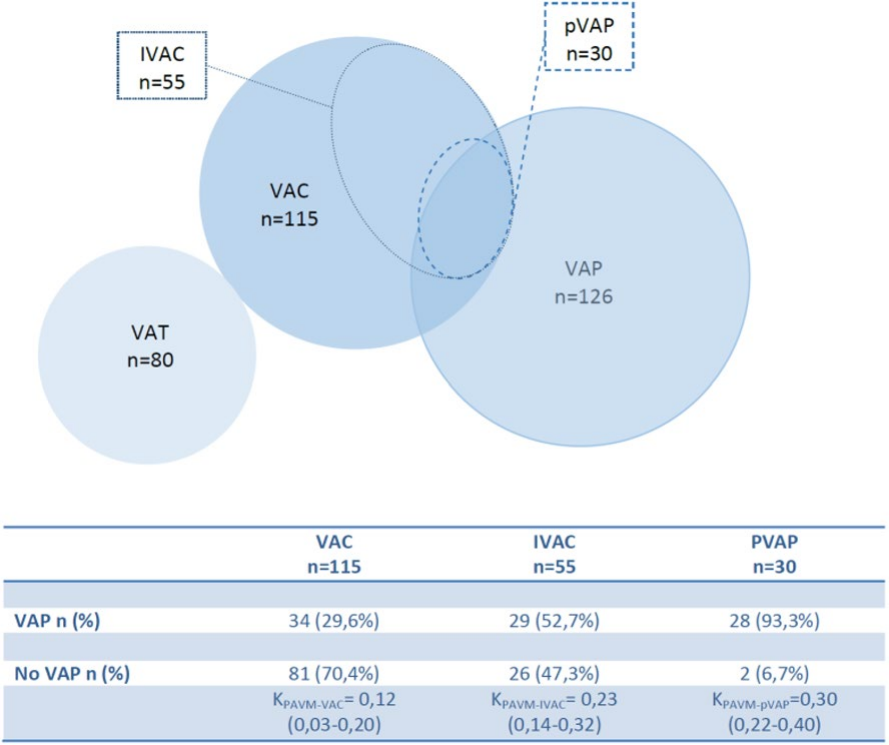
Agreement between VAE and LRTI diagnostic

### P-213 The impact of an antimicrobial stewardship program on management of severe post-operative pneumonia after major lung resection

#### Kernéis Solen^1^, Blanc Kim^1^, Caliez Julien^1^, Canouï Etienne^1^, Loubinoux Julien^1^, Gauzit Rémy^1^, Peretout Jean-Baptiste^1^, Nguyen Yen-Lan^1^, Lefebvre Aurélie^1^, Regnard Jean-François^1^, Alifano Marco^1^, Rabbat Antoine^1^

##### ^1^Hôpital Cochin, Paris, France

###### **Correspondence:** Kernéis Solen - solen.kerneis@aphp.fr

*Annals of Intensive Care* 2018, **8(Suppl 1):**P-213

**Introduction:** Post-Operative Pneumonia (POP) is a frequent and severe complication of major lung resection surgery. In 2002, we changed our surgical antibioprophylaxis protocol from cefamandole to amoxicillin-clavulanate and observed a significant decrease of POP incidence and mortality. In 2012, we additionally implemented in the respiratory intensive care unit (RICU) an antimicrobial stewardship program based on a local antimicrobial guideline and a weekly multidisciplinary review of all antibiotic therapies by RICU physicians, infectious diseases specialists and microbiologists. Our objectives were to describe our current epidemiology of severe POP and to assess the quality of antibiotic prescriptions.

**Patients and methods:** All patients with severe POP occurring within 30 days after lung resection between January 2013 and December 2015 were included. We collected data on clinical presentation, results of microbiological investigations, antibiotic regimen and outcomes. The quality of antibiotic use was assessed using indicators previously validated in the literature.

**Results:** Over 1555 patients who underwent major lung resection in our center, 91 matched criteria for severe POP and were included. Most were males (n = 71, 78%). The median age was 69 years (minimum—42 + maximum—84). Most patients had chronic obstructive pulmonary disease (n = 59, 65%) and 18 (20%) a history of non-pulmonary cancer. The resection consisted in lobectomy in 59% (n = 54). The median length of stay in RICU was 12 days (1 + 90), and 30-day mortality was 9% (n = 8). Respiratory microbiological samples were obtained in all patients, in most cases invasively per bronchoscopy (74%). Microorganisms were cultured at a significant level in 66 (73%) patients. Predominant species were Enterobacteriacae (58%), Haemophilus influenzae (14%), Staphylococcus aureus (11%) and *Pseudomonas aeruginosa* (10%). Microorganisms were sensitive to third generation cephalosporins in 52 (79%) and to piperacillin-tazobactam in 57 (86%). In patients treated empirically, antibiotics were prescribed according to the guideline in 80% (69 86). In documented POP, empiric antibiotics were active against documented micro-organisms in 53 62 (86%), and were correctly changed to pathogen-directed therapy in 46 62 (74%). The median duration of antibiotics was of 7 days (2 + 21).

**Conclusion:** Ten years after implementation of amoxicillin-clavulanate as surgical antibioprophylaxis, the proportion of Enterobacteriacae increased. The 30-day postoperative mortality rate remained below 10%. We report high adherence to the guideline for the choice of empirical therapy and treatment duration. The rate of de-escalation to pathogen-directed therapy could however be improved considering the high rate of bacteriologically-documented POP.

### P-214 Impact of an antibiotic stewardship program on the epidemiology of infection and colonization with *Pseudomonas aeruginosa* in ICU

#### Adrien Vladimir^1^, Rollin Nathalie^2^, Sy Oumar^2^, Iordache Laura^2^, Tebano Gianpietro^2^, Pitsch Aurélia^2^, Vinsonneau Christophe, Mehran Monchi^2^, Jochmans Sebastien^2^, Diamantis Sylvain^2^

##### ^1^10 rue Riquet, Paris, France; ^2^CH de Melun, France

###### **Correspondence:** Adrien Vladimir - vladimir.adrien@free.fr

*Annals of Intensive Care* 2018, **8(Suppl 1):**P-214

**Introduction:** We describe the epidemiology of patients that had a microbiological sample positive with *Pseudomonas aeruginosa* (PA) during their stay in ICU and the impact of an antimicrobial stewardship program, based on the interaction with an infectious disease’s specialist within the unit, aiming in reducing the use of some antibiotic classes (C3G, carbapenems and fluoroquinolones).

**Patients and methods:** Retrospective analysis of medical files in the ICU unit of a general hospital over a 8-year period (2007–2014), of all hospitalized patients that had a positive sample with PA within their stay. Comparison between both periods, 2007–2010 (P1) and 2011–2014 (P2) between which an antibiotic stewardship has been implemented.

**Results:** Among the 5 442 patients, 274 (5%) cases had a positive sample with PA, for which 95 (35%) cases were colonized whereas 179 (65%) cases were infected with PA. Half of the infections were ventilator-associated pneumonias (VAP), which concerned 92 (34%) cases. The average length of stay (ALOS) and the mortality rates were higher among infected patients (respectively 48 vs. 16 days and 47 vs. 20%, p < 0.001) or colonized (respectively 29 vs. 16 days and 36 vs. 20%, p < 0.001). Nevertheless, median age of the cases was higher (72.5 vs. 65 years, p < 0.001). During P2, the number of cases reduced significantly from 136 (7%) cases among 1939 patients, to 146 (4.2%) cases among 3503 patients (RR = 0.8, CI 95% 0.71–0.89). The antibiotic resistance of PA has reduced between both periods from 42% to 11% (p < 0.01) for ceftazidim, from 74% to 18% (p < 0.01) for cirpofloxacin and from 38% to 18% (p < 0.01) for imipenem. Nevertheless, among the cases, the P2 period did not change the risk of developing an infection (RR = 0.77, CI 95% 0.58–1.01), a VAP (RR = 0.99, CI 95% 0.76–1.28), a septicemia (RR = 1, CI 95% 0.66–1.51) or the mortality rates (RR = 1.04, CI 95% 0.81–1.33).

**Conclusion:** Colonization and infection with PA are risk factors of increased mortality rates and ALOS in ICU. An antibiotic stewardship program allows to reduce the incidence of patients having a positive sample with PA, and the antibiotic resistance of PA strains, without reducing the infection rate of these patients.

### P-215 Impact of a local care protocol on the duration of antibiotic therapy in community-acquired peritonitis: 10 years of experience

#### Cadiet Julien^1^, Dumont Romain^1^, Cinotti Raphaël^1^, Mirallie Eric^1^, Asehnoune Karim^1^

##### ^1^CHU de Nantes, Nantes, France

###### **Correspondence:** Cadiet Julien - julien.cadiet@gmail.com

*Annals of Intensive Care* 2018, **8(Suppl 1):**P-215

**Introduction:** The use of antibiotics is a major public health, economic and ecological challenge. In 2011, a French national warning plan was created to manage the use of antibiotics. It advocates monitoring of the prescription of antibiotics and the implementation of measures to assess professional practices. The great majority of guidelines concerning the duration of antibiotic therapy in community-acquired peritonitis are based on studies with low level of evidence. The objective of this study is to evaluate the implementation of a Standardized Operational Report (SOR) with a local antibiotic protocol in the management of community-acquired peritonitis at our institution.

**Patients and methods:** This is a monocentric, prospective cohort study—before and after the establishment of the SOR. The primary endpoint is duration of antibiotic therapy. Secondary endpoints are length of hospitalization, infectious complications, mortality, and changes in local bacterial ecology. We have also evaluated retrospectively these different criteria on cohort was constituted since 2005.

**Results:** A total of 205 patients were enrolled from January 2011 to June 2013 and 231 patients from May 2014 to May 2016. The duration of antibiotic therapy was decreased by 7 to 3 days in localized peritonitis (p < 0.0001) and 9 to 7 days in generalized peritonitis (p < 0.0001) (Figure). However, the compliance to the protocol was only 66%, which leads to an increase in the duration of antibiotic therapy and hospital stay when not used (p < 0.0001). The hospital stay decreased from 7 to 4 days in the localized peritonitis (p < 0.0001). Amoxicillin clavulanic acid (AMC) is the most used antibiotic with an efficiency of 77%. There was no impact on morbidity and mortality when AMC was inadequate. The bacterial ecology was not modified, the rate of extended-spectrum beta-lactamase (ESBL) producing enterobacteria (ESBLE) was 3%.
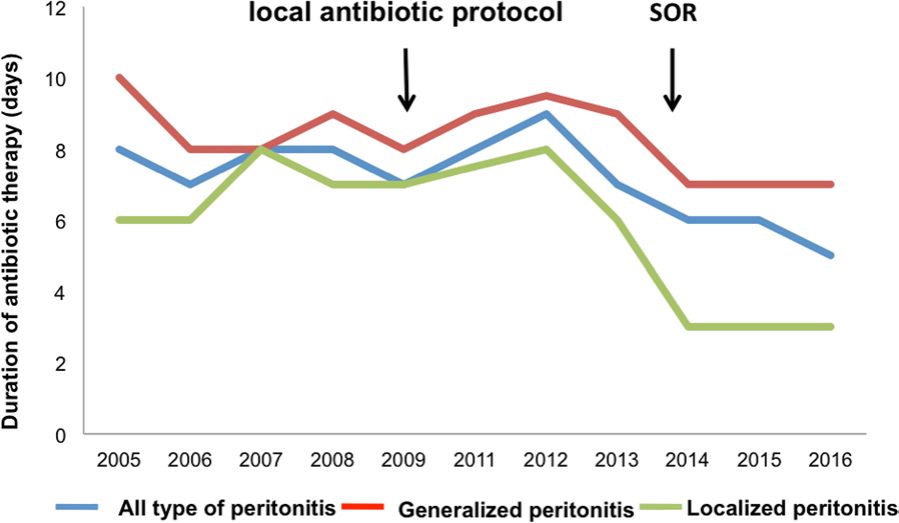


**Conclusion:** The use of a standardized antibiotic protocol reduced antibiotic therapy duration and hospital stay, particularly in localized peritonitis despite incomplete compliance to the protocol. To achieve full compliance, we need to continue the training of different physicians and continue the spread of the protocol.

### P-216 Severe meningitis-Epidemiological data and prognostic factors

#### Ezzouine Hanane^1^, Mountassir Chorouk^2^, Sghier Zineb^2^, Benslama Abdellatif^2^

##### ^1^CHU IBN Rchod, Casablanca, Morocco; ^2^University Hassan II, Casablanca, Morocco

###### **Correspondence:** Ezzouine Hanane - ezzouinehanane@yahoo.fr

*Annals of Intensive Care* 2018, **8(Suppl 1):**P-216

**Introduction:** Bacterial meningitis is an important public health problem because of its frequency and severity. They remain a major cause of mortality and morbidity in developing countries. The aim of our work is to establish the epidemiological characteristics and the prognostic factors .

**Patients and methods:** we did a retrospective descriptive and analytical study and we included all the patients admitted for severe meningitis for 1 year in the medical intensive care unit of the university teaching hospital Ibn Rushd at Casablanca-Morocco.

**Results:** 70 patients were included. The incidence of severe meningtis was 12.61%, the mean age was 44 years old and the sex ratio H F was 1,06.24,3% were pneumococcal meningitis and 20% were tuberculosis In univariate analysis, factors influencing mortality significantly—The male sex Patients with pulmonary tuberculosis as an antecedent.• A low glasgow score at admission. The presence of a neurological deficit on clinical examination. Gravity scores—APACHE II and SAPS II. For lumbar puncture data, there is the proteinuria, glycorrhaphy Resuscitation measures—drug intake and intubationIn multivariate analysis, the factors of pejorative evolution—The male sex Presence of meningeal syndrome. High proteinorachia. Taking vasoactive drugs. The SAPS II score.

**Conclusion:** According to this work, many factors influence the prognosis of acute meningitis in our population such as severity general scores, hemodynamic state and initial lumbar puncture data. We will need more investigations and prospective multicentric study to have more discrimination parameters.

### P-217 Antibiotics’ consommatiion in surgical resuscitation

#### Khaleq Khalid^1^, Hanane Toumi^1^, Derfoufi Sanaa^1^, Zerouali Khalid^1^, Bouhouri M. A^1^, Alharrar R^1^

##### ^1^CHU Ibn Rochd, Morocco

###### **Correspondence:** Khaleq Khalid - khaleq20@gmx.fr

*Annals of Intensive Care* 2018, **8(Suppl 1):**P-217

**Introduction:** The emergence of ATB-resistant bacteria has become an important public health problem, particularly in resuscitation environments, surveillance and monitoring of ATB consumption is essential to combat this threat ecologically and economically. The aim of this work was to evaluate the consumption of ATB in surgical resuscitation, to establish the cost, and to list the risk factors for bacterial resistance.

**Patients and methods:** It is a retrospective analytical study spread over 1 year, studying 225 patients who have received antibiotic therapy, the data on the consumption of ATB were collected from the patient’s medical records, the delivered doses were converted into DDD, according to The WHO standards and the end result is expressed in DDD 1000 days of hospitalization. The statistical analysis was carried out by the SPSS 16 software.

**Results:** In our study, the mean age was 43.15 ± 5.5, with male predominance + Sex ratio 1.71, traumatic pathology is the most common reason for admission, pneumopathy was the most frequent infection. Overall ATB consumption was 1236.11 DDD 1000DH, dominated by the class of Betalactamins (cephalosporins 166.89 DDD 1000DH, carbapenemes 179.68 DDD 1000DH), the direct cost of ATBs rises to 0.33 million dirhams, these are accounting for a large part of the pharmaceutical budget of the Ibn Rochd University Hospital. Bacteria found in order of frequency were Acinetobacter baummanii, beta-lactamase-producing enterobacteria, *S. aureus* and *P. aeruginosa*. Acinetobacter baumannii showed the highest resistance rate. Several risk factors for bacterial resistance were studied, notably the correlation between the use of ATB and the emergence of resistant strains, only piperacillin-tazobactam was associated with the emergence of resistant strains of EBLSE, as well as 2 other factors that were retained as significantly related to bacterial resistance by multivariate analysis—duration of hospitalization and perfusion of albumin.

**Discussion:** Despite the limited number of studies done on ATB consumption, it seemed that our results were similar to other national and foreign studies, the consumption of ATBs is increased in hospital giving rise to the appearance of many multi-resistant bacteria.

**Conclusion:** In conclusion, resistance to antibiotics is a serious threat to public health both nationally and globally. It is therefore crucial to implement measures to counter this phenomenon + this is only possible through the proper use of ATBs and gaits to prevent nosocomial infections.

### P-218 Traumatic brain injury in older adults: risk factors of ventilator-associated pneumonia

#### Sellami Walid^1^, Hajjej Zied^2^, Sammoud Walid^2^, Ben Mrad Ines^2^, Gharssallah Hedi^2^, Labbene Iheb^2^, Ferjani Mustapha^2^

##### ^1^Montefleury, Tunis, Tunisia; ^2^Hôpital Militaire de Tunis, Tunisia

###### **Correspondence:** Sellami Walid - drsellamiwalid@yahoo.fr

*Annals of Intensive Care* 2018, **8(Suppl 1):**P-218

**Introduction:** Ventilaor-associated pneumonia (VAP), the leading cause of infection in resuscitation, is also the main respiratory complication in cranial trauma. The aim of this study is to determine the specific risk factors for the occurrence of VAP in this type of patient in an intensive care unit.

**Patients and methods:** We performed a retrospective study in our intensive care unit for an 18-month period (January 2016, June 2017). All patients admitted for cranial trauma were included in the study and ventilated more than 48 h in intensive care. VAP is defined as late as of the 5th day of occurrence. The quantitative and qualitative variables studied were recorded at admission and during hospitalization. A univariate and multivariate analysis using the Fischer and Mann–Whitney tests was performed. p is said to be significant if it is < 0.05.

**Results:** Our study included 50 traumatic brain injury in older adults, of whom 22 (44%) had one or more episodes of VAP during their resuscitation. Late VAP accounted for almost 2 3 of the cases (33 patients). Four independent variables were significantly related to the occurrence of VAP—advanced age (p = 0.04), Glasgow score (GCS) at admission < 8 (p = 0.01), diabetes (p = 0, 03), and the use of Proton Pump Inhibitors for the prevention of stress ulcers (p = 0.04). The duration of intubation (18 ± 6 vs. 11 ± 3 days) and on intensive care (28 ± 16 vs. 15 ± 3 days) are significantly longer in the case of VAP. Mortality was significantly higher in VAP—45 versus 28% (p = 0.01). The majority of early VAP were due to both Strepococcus ppneumoniae and Haemophilus influenzae. The ecology of late VAP was dominated by *Klebsiella pneumonia*e, *Pseudomonas aeruginosa* and acinetobacter baumanii.

**Conclusion:** Of the four independent risk factors found in our study, glycemic balance and rapid airway safety by orotracheal intubation in the case of initial GCS < 8 represent the 2 relevant prevention axes of VAP in traumatic brain injury in older adults.

### P-219 Nosocomial urinary tract infections in an intensive care unit: resistance profile

#### Lazraq Mohamed^1^, Bensaid Abdelhak^1^, Miloudi Youssef^1^, Elharrar Najib^1^

##### ^1^Hospital 20 aout, Casablanca, Morocco

###### **Correspondence:** Lazraq Mohamed - mohamed_lz@hotmail.com

*Annals of Intensive Care* 2018, **8(Suppl 1):**P-219

**Introduction:** Nosocomial urinary tract infection is the leading cause of nosocomial infection and the second cause in intensive care unit. Unfortunately, it is accompanied by a significant increase in bacterial resistance to antibiotics, leading to an increase in morbidity and mortality in intensive care units.

**Patients and methods:** This is a retrospective study carried out in our intensive care unit, covering all patients hospitalized between January 2010 and June 2014 and having contracted a nosocomial urinary infection. Patients whose hospital stay was less than 48 h and those with a nosocomial urinary tract infection acquired in another service were excluded.

**Results:** The study of resistance of the germs responsible for nosocomial urinary tract infection showed that-Escherichia-coli was resistant to Third generation cephalosporins in 21% of cases, at imipenem in 8% of cases, and without resistance to ertapenem and amikacin. Pseudomonas was resistant to ceftazidime in 61% of cases, to imipenem in 64% of cases and to amikacin in 44% of cases. Acinetobacter baumannii was resistant to imipenem in 87% of cases and to amikacin in 85% of cases. Enterococcus faecalis had no resistance to vancomycin and ampicillin. Staphylococcus aureus was resistant to methicillin in 25% of cases and without any resistance to vancomycin. Mortality directly associated with nosocomial urinary tract infection was 11%.

**Conclusion:** The comparison with previous studies has shown a significant increase in the bacterial resistance responsible for nosocomial urinary tract infection, which is of interest in monitoring the ecology of intensive care units and the resistance profile as well as the improvement of the management of antibiotics.

### P-220 Nosocomial enterococcus infections—outcome comparison according to susceptibility to vancomycin and predictive mortality factors

#### Jamoussi Amira^1^, Doghri Hamdi Hemdene^1^, Ayed Samia^1^, Merhebene Takoua^1^, Ben Khelil Jalila^1^, Besbes Mohamed^1^

##### ^1^Hôpital Abderrahmen Mami, Ariana, Tunisia

###### **Correspondence:** Jamoussi Amira - dr.amira.jamoussi@gmail.com

*Annals of Intensive Care* 2018, **8(Suppl 1):**P-220

**Introduction:** Nosocomial enterococcus infections are a constant concern in intensive care units due to their increasing frequency and the emergence of resistant strains to vancomycin. The aim of our study was to compare outcome findings of patients with nosocomial enterococcus infections according to their sensibility to vancomycin, and then to investigate predictive factors of mortality.

**Patients and methods:** It was a retrospective descriptive study, including all hospitalized patients in intensive care, between January 1st, 2013 and April 1st, 2017, with nosocomial enterococcus infections. We recorded demographic and clinical findings, severity scores IGS II, APACHE II, initial SOFA and SOFA at the time of infection, microbiological, therapeutic and outcome data. Patients infected with vancomycin-susceptible enterococcus (VSE) were compared to those having vancomycin-resistant enterococcus (VRE) + then we searched for independent risk factors for VRE. Finally, a multivariate logistic regression was conducted to investigate independent predictive mortality factors.

**Results:** During the study period (4 years and 3 months), 35 patients presented a nosocomial enterococcus infection with a median age of 63 years [25–87] and a sex-ratio of 2.18. At admission, 31 patients (83.3%) had respiratory distress. The median scores of IGS II, APACHE II, initial SOFA and SOFA at the time of infection were respectively 45 + 16 + 6 and 6. The infection sites were—urinary infection (n = 17, 48.6%), bacteremia (n = 15, 42.9%) and central line associated infection (n = 3, 8.5%).16 patients had a VRE nosocomial infections and 19 VSE. A septic shock complicated enterococcus infection in 13 cases including 7 cases of VRE and 6 cases of VSE (p = 0.458). VRE nosocomial infections were significantly related to arterial (p = 0.006) and venous (p = 0.009) femoral catheterization, to a duration of venous femoral catheterization > 3 days (p = 0.035) and to *E. faecium* species (p < 10–3). No independent risk factor of VRE was found. The median duration of hospitalization was 34 days and the overall mortality rate was 68.6%. Multivariate analysis identified 2 independent predictive factors of attributable mortality—patients in coma (OR 30.44 + IC 95% = 1.99–465.39 + p = 0.014) and the occurrence of septic shock (OR 14.4 + IC 95% = 1.45–137.72 + p = 0.023).

**Conclusion:** Attributable mortality to nosocomial enterococcus infections was high and independent of the susceptibility of the strain to vancomycin. Mortality was independently associated to septic schock occurrence and neurologic dysfonction.

### P-221 Ventilator-Associated Pneumonia-incidence, risk factors and mortality

#### Ayed Samia^1^, Aouadi Walid^2^, Amara Yesser^2^, Ounalli Karim^2^, Kammoun Mohamed Hedi^2^

##### ^1^Ariana, Tunisia; ^2^Tunis, Tunisia

###### **Correspondence:** Ayed Samia - samia.ayed@yahoo.fr

*Annals of Intensive Care* 2018, **8(Suppl 1):**P-221

**Introduction:** Ventilator-Associated Pneumonia (VAP) is defined by a lung infection contracted 48 h after the putting under mechanically assisted breathing. Risk factors predisposing to the development of VAP among mechanically ventilated patients are many. Some are related to the patient as age, history of COPD, presence of an altered state of consciousness + others are related to care providing.

**Patients and methods:** A prospective nested case control study was conducted from Marsh 2014 through April 2015. All ICU patients mechanically ventilated for more than 48 h with endotracheal intubation or tracheostomy were included. Cases of community-acquired pneumonia, non-mechanical ventilated hospital-acquired pneumonia, end-life patients and those aged less than 18 years were excluded. The included patients with VAP and those without VAP were matched based on the age, the severity score and the comorbidities. For all patients included, preventive measures as assessed by the recent guidelines for preventing VAP were applied after an education period of all medical and paramedical staff of the ICU. The collected data are—age, comorbidities, admission severity scores, time to onset of VAP, prior antibiotic therapy at the onset of VAP, need for tracheostomy, duration of mechanical ventilation, length of stay in ICU and become.

**Results:** During the study period, 71 patients were mechanically ventilated. VAP was observed in 42% of cases. VAP was observed in 27 cases with an incidence of 38% and incidence density of 21 per 1000 patient-days of mechanical ventilation (MV). In univariate analysis, significant difference was found between the group with VAP and the group without VAP regarding admission for poly trauma, acute respiratory failure, the concept of prior antibiotic therapy, the need tracheostomy, the number of days alive without antibiotics and without MV, the duration of mechanical ventilation, length of stay and mortality. Multivariate analysis showed that prior antibiotic therapy and the use of tracheotomy were independent factors for developing VAP. Prolonged duration of mechanical ventilation was an independent predictor of mortality in multivariate analysis with OR 1.773 + 95% [1.202 to 2.616], p = 0.04. The occurrence of VAP was not an independent predictor to mortality.

**Conclusion:** The incidence of VAP found in our study is similar to that found in the literature. An active strategy of rationalizing the prescription of antibiotics in intensive care units and a well-defined protocol of weaning from mechanical ventilation may reduce the incidence of VAP and over-all morbidity and mortality.

### P-222 Epidemiology of nosocomial infections in a Tunisian medical intensive care unit

#### Ayed Samia^1^, Ben Ismail Khaoula^1^, Jamoussi Amira^1^, Merhebene Takoua^1^, Ghariani Asma^1^, Slim Leila^1^, Ben Khelil Jalila^1^, Besbes Mohamed^1^

##### ^1^Hôpital Abderrahmen Mami, Ariana, Tunisia

###### **Correspondence:** Ayed Samia - samia.ayed@yahoo.fr

*Annals of Intensive Care* 2018, **8(Suppl 1):**P-222

**Introduction:** Intensivists are often threatened by the spectre of nosocomial infection (NI) that burdens morbimortality. That’s why knowing the epidemiology of NI represents the first step for control of this complication. We aimed to describe epidemiology of pathogens and types of NI.

**Patients and methods:** This was a retrospective study conducted within a 22-bed medical ICU of a Tunisian teaching hospital (301 beds) carried from January 1st to December 31, 2016. We recorded demographic patients’ characteristics which ICU’s stay was complicated with at least one NI. Then we described pathogens and microbiological specificities of NI.

**Results:** During this period, 59 patients out of a total of 590 admitted to ICU developed NI (incidence 10%). The mean age was of 59.9 years ± 17.3 and the sex-ratio was of 3.2. Most frequent comorbidities were—COPD (57.6%), hypertension (37.3%) and diabetes (27.1%). The main reason for ICU admission was acute respiratory failure (69.5%), Shock (15.3%) and coma (10.2%). SAPS II and SOFA scores were respectively (50.31 ± 29.94) and (11.76 ± 6.5). Almost half of them (47.5%) have got antibiotics shortly before ICU admission—Amoxicillin-clavulanate (25.4%) and quinolones (8.5%). We recorded 91 episodes of nosocomial infections—pneumonia (n = 45, 49.5%), bacteremia (n = 26, 28.6%), catheter related infections CRI (n = 13, 14.3%) and urinary infections (n = 7, 7.6%). Pathogens isolated were largely dominated by non-fermentent Gram—negative bacilli (n = 55, 60.4%)—Acinetobacter Baumanii (n = 39, 42.8%) with 100% resistance to imipenem and tygecycline, *Pseudomonas aeruginosa* (n = 14, 15.4%) with 33.3% resistance to ceftazidim and stenotrophomonas maltophila (n = 2). Other Gram—negative bacilli were enterobacteries (n = 11), which were wide-spectrum betalactamase secreting (n = 6) and carbapenemase (n = 1). Gram-positive cocci were the second highest (n = 22, 24.2%)—coagulase negative Staphylococcus (n = 12) which were resistant to methicilline (75%), enterococcus (n = 7) which were resistant to vancomycin (n = 2, 28.6%), Staphylococcus aureus sensitive to methicilline (n = 2) and streptococcus (n = 1). Candida was incriminated in 2 cases of CRI. Pathogens distribution according to NI localizations is shown in Fig. 1.

**Fig. 1:**
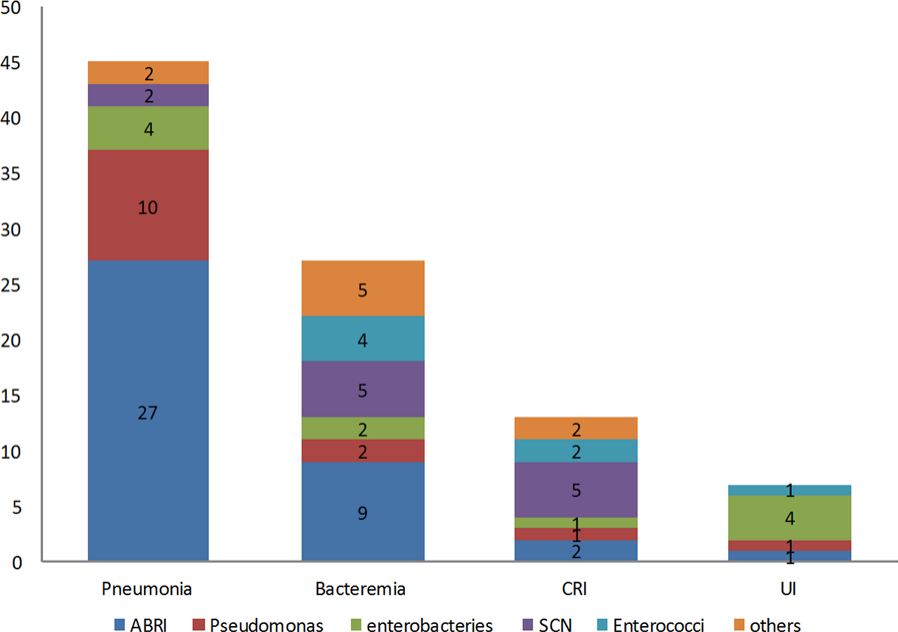
Pathogens distribution according to localization of nosocomial infection

**Conclusion:** Incidence of NI remains high in ICU. ABRI was the most frequent pathogen. More efforts in hygiene precautions should be deployed to limit multi-drug resistant pathogens propagation.

### P-223 Hyperoxemia impact on the mortality in patients with ventilator-associated pneumonia

#### Six Sophie^1^, Rouze Anahita^1^, Pouly Olivier^1^, Poissy Julien^1^, Wallet Frederic^1^, Preau Sebastien^1^, Nseir Saad^1^

##### ^1^CHRU LILLE, Lille, France

###### **Correspondence:** Six Sophie - sixsophie@hotmail.com

*Annals of Intensive Care* 2018, **8(Suppl 1):**P-223

**Introduction:** Hyperoxemia is common in critically ill patients. Hyperoxic acute lung injury (HALI), reduced bacterial clearance, atelectasis and higher mortality rates were reported in mechanically ventilated patients with hyperoxemia. The aim of our study was to determine the relationship between hyperoxemia and mortality in patients with ventilator-associated pneumonia (VAP).

**Patients and methods:** This retrospective observational single center study was performed in a 50-bed mixed intensive care unit (ICU) during a 1-year period, from January 2016 to January 2017. All patients with VAP were included. VAP was defined using clinical, radiological and quantitative microbiological criteria. Hyperoxemia was defined as peripheral capillary oxygen saturation—SpO2 ≥ 98%. SpO2 was hourly collected in all study patients during the whole period of mechanical ventilation. The daily percentage of time spent with hyperoxemia was calculated as the number of hours with hyperoxemia divided by 24.

**Results:** Among the 547 patients receiving invasive mechanical ventilation (MV) > 48 h during the study period, the incidence rate of VAP was 11.7 VAP per 1000 ventilator-days. 93 patients developed VAP and were all included in this study. 30 (32%) VAP patients died in the ICU. The mean daily time spent with hyperoxemia was 45%. No significant difference was found in mean percentage of time spent with hyperoxemia between survivors and nonsurvivors at ICU admission, before, after or at the VAP diagnosis. Age, and sequential organ dysfunction assessment (SOFA) at the day of VAP occurrence were independently associated with ICU mortality (OR 1.04 [1.007–1.075] per year, p = 0.02 + 1.19 [1.064–1.339] per point, p = 0.003 + respectively). No significant impact was found of time spent with hyperoxemia before VAP occurrence, on MV free days, or ICU length of stay (Fig. 1).

**Fig. 1:**
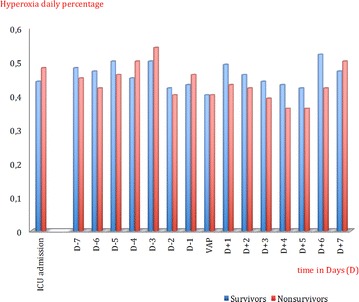
Duration of hyperoxia (in %) over the time in survivors (blue bars) and in non-survivors (red bars)

**Discussion:** Several potential explanations could be provided for the absence of negative impact on mortality of hyperoxemia. First, the definition used for hyperoxemia could be debated, as no consensus exists. However, the definition used in our study was rather stringent and the mean daily time spent with hyperoxemia was in line with that reported by studies. Second, the impact of hyperoxemia on mortality could have been confounded by a large number of patients included with pulmonary lesions at admission. Third, the number of included patients was small.

**Conclusion:** Our study found no significant impact of hyperoxemia at ICU admission, or during ICU stay, on ICU mortality in VAP patient.

### P-224 Acquired pneumonia in mechanical ventilation at the ICU of Oran

#### Azza Abdelkader^1^, Benbernou Soumia^2^, Mokhtari Djebli Houria^2^

##### ^1^Institut National de l’enseignement en sciences medicales, Oran, Algeria; ^2^Medecine faculty of oran, Oran, Algeria

###### **Correspondence:** Azza Abdelkader - geopardy2006@hotmail.com

*Annals of Intensive Care* 2018, **8(Suppl 1):**P-224

**Introduction:** Ventilation is one of the first gestures performed in ICU, sometimes the patients under machine are complicated by lung infections that occur during hospitalization. According to a published study, ventilator aquired pneumonia PAV constitute nearly half of nosocomial infections and are the leading cause of death in intensive care units.

**Patients and methods:** Retrospective study spread over 2 years from January 2014 to December 2015 carried out in the resuscitation unit of the medical and surgical emergency department of CHUOran. Data utilization with Whonet software. The criteria of vap retained are those of CTINILS (May 2007).

**Results:** 128 patients collected during this period. 228 distal protected specimens were performed in patients suspected of VAP. The diagnosis of this infection was made. In 55 of them with other diagnostic criteria (43%) which represents an incidence density of 8.2 per 1,000 days.80% of PAVM are due to gram negative bacilli. The first germ involved in our series and pseudomonas (30%) followed by klebsielles (15%) followed by acintobacter baumanii (8%) enterobacteries representing the rest.12% lung infection with Gram-positive cocci (principally sensitive methicillin) pseudomonas was imipenem resistant in 18.4%, baumanii was imipenem resistant in 55%.

**Conclusion:** The resistance profile of the recovered germs (baumanii and pseudomonas) encourages the utmost rigor in the management of these patients, prevention is better attitude to adopt.

### P-225 Epidemiology and risk factors of ventilator associated pneumonia in a moroccan hospital

#### Qamouss Youssef^1^, Aissaoui Younes^1^

##### ^1^Hopital Militaire Avicenne, Marrakech, Morocco

###### **Correspondence:** Qamouss Youssef - yqamouss@yahoo.fr

*Annals of Intensive Care* 2018, **8(Suppl 1):**P-225

**Introduction:** The ventilator associated pneumonia (VAP) appear in the second rank of the infections acquired in hospital after the urinary infections. The diagnosis is based on a beam of clinical, biological, radiological and bacteriological arguments. This work consisted of an epidemiologic analysis of the VAP and aimed at evaluating of it the frequency, the risk factors, the antibioresistance of the isolated bacteria and the mortality factors.

**Patients and methods:** This retrospective study related to 906 patients hospitalized in ICU during a period of 3 years from January 2014 to December 2016. The study included all patients over 18 years and ventilated more than 48 h and developing VAP.

**Results:** BGN predominant and represent 80.16% of identified germs, the Acinetobacter baumanni leads with 33.88%, followed by klebsiella pneumonia (13.22%), followed by *Pseudomonas aeruginosa* (9.92%), followed by *E. coli* (6.61%), followed by *Enterobacter cloacae* (3.31%) and citrobacter frendi (1.65%). The cocci gram positive (CGP) constitue 18.19% of isolated germs of witch 14.88% Staphylococcus aureus, 0.83% of non aureus staphylococci, 2.48% Streptococcus sp. The polymicrobism was found in 33% cases. The isolated germs were multiresistants. In this study, we find a very high mortality and a major additional morbidity of the NP by prolongation of hospitalization, of mechanical ventilation and a major additional cost.

**Conclusion:** It appears in the light of this work that a strategy of prevention based on the strict application of hygiene measurements, the maintenance of the material of ventilation and the respect of care procedures prove to be urgent in our context.

### P-226 Pharmacokinetic evaluation of single daily dose of amikacin in burn patients

#### Mokline Amel^1^, Ben Salem Mouna^1^, Rahmeni Imen^1^, Gharsallah Lazheri^1^, Harzallah Ines^1^, Messadi Amen Allah^1^

##### ^1^Burn and Trauma Center, Tunis, Tunisia

###### **Correspondence:** Mokline Amel - dr.amelmokline@gmail.com

*Annals of Intensive Care* 2018, **8(Suppl 1):**P-226

**Introduction:** Burns induce modification of distribution volume, increased clearance of drugs and decrease of protein binding. Amikacin pharmacokinetics (PK) was altered with subthera-peutic serum concentrations. The aim of our study was to assess the PK of amikacin in burns after a loading dose given once a day according to this equation—Dose(mg kg) = 30*Pi(30*0,4*Dp1) + (30*0,4* Dp2). Threshold for therapeutic efficacy was a ratio of ≥ 8 between the concentration achieved 1 h after beginning the infusion (C peak) and the minimal inhibitory concentration (MIC) of the isolated pathogen.

**Patients and methods:** This study was conducted in burn center in Tunis. Patients with documented and or suspected infections were included. Were excluded pregnant women and patients with renal failure. Enrolled patients received amikacin at a loading dose in 1 h infusion. Blood samples for PK analysis were assessed during 5 days (total duration of amikacin)—immediately after the end of the first infusion (T1) and 30 min after (T2) at day 1. For the 2nd, 3rd, 4th and 5th day, blood samples were taken before the infusion (T0), at the end (T1) and 30 min after the end of it (T2).

**Results:** 18 burned patients were included. The mean age was 40 ± 15 years with a body weight of 78 ± 15 kg. The mean dose of amikacin was 26 mg kg day [20–35 mg kg day]. A peak between 60 and 80 μg/ml was reached in 55% of cases, corresponding to 8 times the MIC, break-points for Enterobactericeae and Pseudomonas Aeuroginosa. Total volume of distribution was 0.55 L kg (0.28–1.32) L kg, half-life time (t1 2) was 4.70 h [1.42–8 h] and the amikacin clearance was 6.4 L h. A correlation was found between Cpeak At day 1 and Cpeak at day 5 (r = 0.45).

**Conclusion:** Our study shows that an early achievement of an optimal Cpeak MIC ratio of amikacin was reached in half of cases with a correlation between Cpeak in the beginning and at the end of treatment. So, initial Cpeak was useful tu adjuste AMK therapy in burns and predicts treatment efficacy. *Pi—ideal weight + Dp1—admission weight—ideal weight + Dp2—actuel weight—admission weight.

