# Correction to: P-42 Distribution and antifungal susceptibility of Candida isolates in a Tunisian burn unit

**DOI:** 10.1186/s13613-019-0555-2

**Published:** 2019-07-04

**Authors:** Trabelsi Sonia, Hanachi Majdi, Bouchekoua Meriam, Aloui Dorsaf, Cheikhrouhou Sarra, Khaled Samira, Messadi Amen Allah, Thabet Lamia

**Affiliations:** 1 Charles Nicolle Hospital, Tunis, Tunisie; 2 Traumatology and Great Burned Center, Tunis, Tunisie

## Correction to: Ann. Intensive Care 2018, 8(Suppl 1):13 10.1186/s13613-017-0345-7

After publication of this supplement [1], it was brought to our attention that in abstract P-42 an author’s name has been erroneously spelled. It should be read “Hanachi Majdi” instead of “Hanchi Majdi”.

Introduction: Burned patients are at high risk of yeast colonization and thus of invasive fungal infections, particularly to Candida (C.) spp., leading to an increase in morbidity and mortality. While pre-emptive antifungal therapy has improved survival, it may lead to an increase in antifungal resistance. The objectives of this work were to describe Candida species distribution and to determine the antifungal susceptibility of Candida isolates acquired in a burn unit.

Patients and methods: Our study is a retrospective review of 17 severely burned patients admitted to the burn unit of the Ben Arous Traumatology and Burns Center with one or more positive culture sites for Candida, during the 16-month period from May 2016 through August 2017. A total of 42 isolates were thus obtained. The susceptibility to 6 antifungal drugs (5-fluorocytosine, fluconazole, ketoconazole, miconazole, itraconazole and amphotericin B) was determined using the Fungitest® broth dilution method for patients with infected normally sterile body sites or a Candida colonization index superior or equal to 0.4. Since echinocandin and anidulafungine were recently introduced in Tunisia, the susceptibility to these antifungal classes was tested for only one patient from our cohort.

Results: Nasal and buccal sites were the most colonized body sites (21.4% each), followed by axillary (11.9%) and rectal sites (9.5%) and urines (9.5%). *C. albicans* was the predominant species (45.2%), followed by *C. glabrata* (38.1%), *C. tropicalis* (7.1%) and *C. parapsilosis* (4.8%). Among the strains whose antifungal susceptibility was determined, majority of Candida isolates were susceptible to fluconazole (77.8%), which is the most frequently used molecule as a pre-emptive treatment in such cases in Tunisia due to its availability and its efficiency. On the other hand, 11.1% of the isolates were intermediate and 11.1% were resistant to this antifungal drug, mainly *C. glabrata* for both groups. As for the other tested azoles, high rates of intermediate strains were noticed (81.5% to itraconazole, 40.7% to ketoconazole and 33.3% to miconazole), mostly *C. glabrata*. Only one strain was resistant to amphotericin B, which is not usually used in these cases due to its nephrotoxicity and the frequency of kidney failure in burned patients.

Conclusion: Our study demonstrates that *C. albicans* is the most frequent species in burn unit-acquired candidiasis. No major antifungal resistance was observed, apart from high rates of intermediate strains (mainly *C. glabrata*) to azole class antifungal drugs.
